# Global burden of hematologic malignancies and evolution patterns over the past 30 years

**DOI:** 10.1038/s41408-023-00853-3

**Published:** 2023-05-17

**Authors:** Nan Zhang, Jinxian Wu, Qian Wang, Yuxing Liang, Xinqi Li, Guopeng Chen, Linlu Ma, Xiaoyan Liu, Fuling Zhou

**Affiliations:** 1 Department of Hematology, Zhongnan Hospital of Wuhan University, Wuhan, Hubei China; 2 School of Nursing, Wuhan University, Wuhan, Hubei China

**Keywords:** Haematological cancer, Epidemiology, Risk factors

## Abstract

Hematologic malignancies are among the most common cancers, and understanding their incidence and death is crucial for targeting prevention, clinical practice improvement, and research resources appropriately. Here, we investigated detailed information on hematological malignancies for the period 1990–2019 from the Global Burden of Disease study. The age-standardized incidence rate (ASIR), the age-standardized death rate (ASDR), and the corresponding estimated annual percentage changes (EAPC) were calculated to assess temporal trends in 204 countries and territories over the past 30 years. Globally, incident cases of hematologic malignancies have been increasing since 1990, reaching 1343.85 thousand in 2019, but the ASDR for all types of hematologic malignancies has been declining. The ASDR for leukemia, multiple myeloma, non-Hodgkin lymphoma, and Hodgkin lymphoma were 4.26, 1.42, 3.19, and 0.34 per 100,000 population in 2019, respectively, with Hodgkin lymphoma showing the most significant decline. However, the trend varies by gender, age, region, and the country’s economic situation. The burden of hematologic malignancies is generally higher in men, and this gender gap decreases after peaking at a given age. The regions with the largest increasing trend in the ASIR of leukemia, multiple myeloma, non-Hodgkin lymphoma, and Hodgkin lymphoma were Central Europe, Eastern Europe, East Asia, and Caribbean, respectively. In addition, the proportion of deaths attributed to high body-mass index continued to rise across regions, especially in regions with high socio-demographic indices (SDI). Meanwhile, the burden of leukemia from occupational exposure to benzene and formaldehyde was more widespread in areas with low SDI. Thus, hematologic malignancies remain the leading cause of the global tumor burden, with growing absolute numbers but sharp among several age-standardized measures over the past three decades. The results of the study will inform analysis of trends in the global burden of disease for specific hematologic malignancies and develop appropriate policies for these modifiable risks.

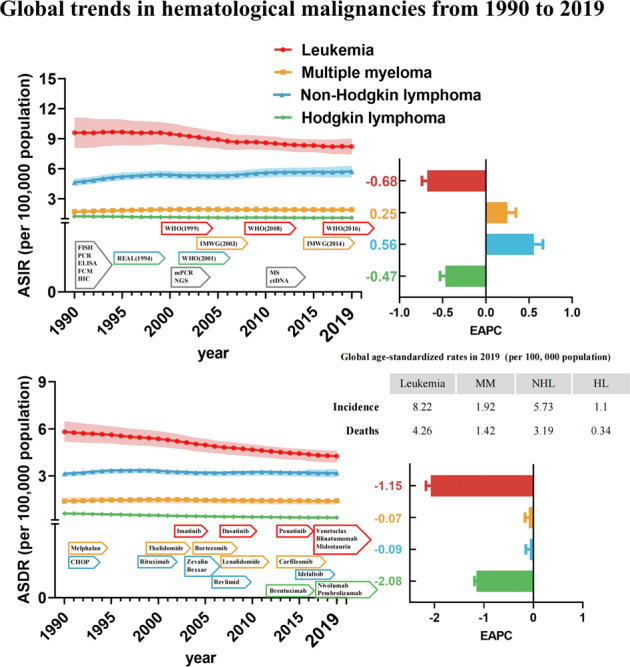

## Introduction

Health systems are challenged by a rapidly aging population, which is causing an increase in the burden of hematologic malignancies [1, 2]. The epidemiological transitions and demographic changes have led to increased prioritization of malignant tumors around the world [3]. Evidence regarding the disease incidence, disease prevalence, and disease burden associated with hematologic malignancies worldwide is limited. Only several local studies have reported the burden of individual diseases [4, 5]. We have previously reported on the clinical and basic mechanisms of hematologic malignancies [6, 7]. However, a more comprehensive and accurate understanding of the magnitude and trends of all hematological malignancies is not yet available, and evidence-based epidemiological studies are needed to become essential for healthcare decision-making and planning.

Hematological malignancies are myeloid and lymphatic tumors caused by disruption of normal hematopoietic function [8]. They are classified into several common subtypes, generally consisting of leukemia, multiple myeloma (MM), non-Hodgkin lymphoma (NHL), and Hodgkin lymphoma (HL) [9]. As the number of cancer cases increases, the spectrum of hematologic malignancies is also changing. For example, the incidence of leukemia is declining globally, but it is still rising in developed regions (such as France, Spain, Slovenia, and Cyprus). Countries and regions differ in the types of hematologic malignancies on account of differences resulting from different socioeconomic development stages [10]. Although survival rates for patients with hematological malignancies have improved dramatically over the past few decades, knowing the specific patterns and time trends in morbidity and mortality from hematologic malignancies remains a priority, which can help to develop more targeted prevention strategies.

The Global Burden of Disease (GBD) project, providing the best possible comparable estimates of ill health, injury, and risk factors, is an important achievement of the long-term collaboration between governments around the world [11]. A key benefit of this resource is that it provides insights into the epidemiological dynamics of hematological malignancies. In this study, we extracted epidemiological data on the incidence and death of hematologic malignancies based on subtype, sex, and age groups from GBD. The disease burden of hematological malignancies was further assessed by determining time trends of hematologic malignancies generated by specific aetiologies at global, regional, and country levels between 1990 and 2019. The results of our study can be an important extension of previous studies and contribute to the development of hematological malignancies prevention strategies in different countries.

## Methods

### Data sources

The GBD study provides comprehensive estimates of the incidence, deaths, and prevalence of diseases for each country and territory. The detailed procedures for collecting, processing, and generating this dataset have been extensively reported in GBD Study 2019 [12, 13]. As a whole, all available information is utilized in the GBD study, including survey data, surveillance data, published literature, as well as hospital and clinical information. The Bayesian meta-regression tool DisMod-MR 2.1 was utilized to synthesize available resources of data and generate internally consistent projections of incidence and deaths, providing a 95% uncertainty interval (95% UI). Studies on the epidemiology of hematological malignancies benefited from regional statistical analyses of morbidity and mortality. The complete datasets of hematological malignancies are accessible from the Global Health Data Exchange (http://ghdx.healthdata.org/).

### Data collection

We collected information from 204 countries and territories, including incidence, death, sex, age, and age-standardized rate (ASR) of hematological malignancies from 1990 to 2019. Specifically, we obtained information on the morbidity and mortality of four major hematologic malignancies, namely leukemia, MM, NHL, and HL, among which leukemia includes acute myeloid leukemia (AML), chronic myeloid leukemia (CML), acute lymphoid leukemia (ALL), chronic lymphoid leukemia (CLL), and other leukemia. The socio-demographic indices (SDI) are a comprehensive predictor that describes the socio-demographic level of a country and is strongly correlated with the outcome of human health development [14]. Following the classification of these countries and territories by their SDI, five distinct regions were defined: high, high-middle, middle, low-middle, and low. Furthermore, 21 geographic regions in 204 countries were defined around the world based on similar geography to facilitate data follow-up at the regional level (Table S1) [14, 15].

### Case definition

The GBD 2019 study adopted the International Classification of Diseases (ICD) definition of hematologic malignancies by the World Health Organization (WHO), which refers to cancers originating from blood-forming cells. Incidence included causes coded as AML (C92.0–C92.02, C92.3–C92.62, C93.0–C93.02, C94.0–C94.02, C94.2–C94.22), ALL (C91.0–C91.02), CML (C92.1–C92.12), CLL (C91.1–C91.12), MM (C88–C90.32), NHL (C82–C85.29, C85.7–C86.6, C96–C96.9), HL (C81–C81.49, C81.7–C81.79, C81.9–C81.99, Z85.71–Z85.72). Death included causes coded as AML (C92.0, C92.3–C92.6, C93.0, C94.0, C94.2, C94.4–C94.5), ALL (C91.0), CML (C92.1), CLL (C91.1), other leukemia (C91.2–C91.9, C92.2, C92.7–C92.9, C93.1–C93.9, C94.1, C94.3, C94.6–C95.9), MM (C88–C90.9), NHL (C82–C86.6, C96–C96.9), HL (C81–C81.9). Unspecified hematologic malignancies were not taken into account as the ICD-10 classification lacks the ability to differentiate between disease subtypes within each hematologic malignancy category.

### Risk factor

GBD risk factors are estimated using a comparative risk assessment framework that involves six steps. The first step involves identifying risk-outcome pairs that have convincing or plausible evidence according to World Cancer Research Fund criteria. Only these pairs are included in the estimation of GBD risk factors. The second step is estimating relative risk (RR) as a function of exposure for each risk-outcome pair. The third step is determining the distribution of exposure for each risk factor by age, sex, location, and year. The fourth step is determining the theoretical minimum risk exposure level (TMREL). The fifth step involves estimating the population attributable fraction (PAF) and attributable burden, using the RR for each risk-outcome pair, exposure levels, and TMREL. This information is used to model the PAF and then multiplied by cancer deaths to generate the deaths attributable to that risk factor. The sixth step involves estimating the PAF and attributable burden for the combination of risk factors. The details of each of these steps and the underlying methodology are published elsewhere [16]. Eighty-seven risk factors were included in this GBD iteration, in which the contribution of the risk attributable to occupational exposure to formaldehyde and benzene to leukemia mortality was nonzero. Data on occupational exposure to carcinogens were collected from the International Labour Organization (ILO), which provided information on the proportion of the population ever exposed to benzene/formaldehyde at work or through their occupation based on population distributions across 17 economic activities.

Where individual-level survey data were available, the mean body-mass index (BMI) was calculated using weight and height [17]. High BMI for adults (aged over 20 years) was defined as a BMI greater than or equal to 25 kg/m^2^. High BMI for children (aged 1–19 years) was defined as being overweight or obese based on International Obesity Task Force (IOTF) standards. For individuals aged over 20 years, we considered them to be overweight if their BMI was greater than or equal to 25 kg/m^2^, and obese if their BMI was greater than or equal to 30 kg/m^2^. For individuals aged 1 to 19 years, we used monthly IOTF cutoffs [2] to determine overweight and obese status when age in months was available [18]. The individual and combined age-standardized summary exposure values (SEVs) for each area-associated risk factor are provided in Table S2.

### Statistical analysis

To quantify trends associated with the incidence and death of hematologic malignancies in various regions, we used the ASR and estimated annual percentage change (EAPC) [19–21]. The standardization of data is necessary when comparing several populations with varying age structures or when comparing the same population over time, as the age profile changes [13]. The age-standardized incidence rate (ASIR) and age-standardized death rate (ASDR) per one hundred thousand population were computed by summing the products of age-specific rates (where *α*_*k*_ = distribution of the selected reference standard population in the k age groups, and *β*_*k*_ = age-specific rate). Based on the age distribution, a weighted average of rates was calculated, the formulae are described below:

$${{{\mathrm{ASR}}}} = \frac{{\mathop {\sum}\nolimits_{k = 1}^n {\alpha _\kappa \beta _\kappa } }}{{\mathop {\sum}\nolimits_{k = 1}^n {\beta _k} }} \times 100,000$$

The EAPC and their 95% confidence intervals (CIs) are a comprehensive measure of ASR trends over a specific period, with lower bounds above 0 points to an upward trend, whereas upper bounds below 0 points to a downward trend. We use a log-linear regression to calculate (where y = ln[ASR], and x = calendar year), as follows:

$${{{\mathrm{y}}}} = \alpha + \beta {{{\mathrm{x}}}} + \varepsilon$$

$${{{\mathrm{EAPC}}}} = 100\% \times (e^\beta - 1)$$

In addition, correlations between EAPC and ASR in 1990, EAPC and SDI in 2019, and ASR and SDI were analyzed for all hematologic malignancies and their subtypes. The global incidence and death of leukemia, MM, NHL, and HL were mapped by the country, including the ASR (in 2019), the percentage change in cases, and the EAPC from 1990 to 2019. Pearson correlation analysis was performed to estimate the ρ indices and p-value of the correlation. To clarify the combined effect of the GBD risk factors, we calculated a scalar describing the proportion of prevalence attributable to high BMI, occupational exposure to benzene and formaldehyde. All data visualization and statistical analysis were performed using GraphPad Prism (version 8.02) and R software (version 3.5.2).

### Patient and public involvement

GBD Study is an international scientific collaboration. In designing the study, we did not consider involving patients since we used secondary data from the GBD Study 2019, and the research question did not relate directly to the management of patients with hematologic malignancies. Researchers did not involve patients in designing the study, collecting and analyzing data, interpreting results, or drafting the manuscript.

## Results

### Global burden of hematologic malignancies

**Fig. 1:**
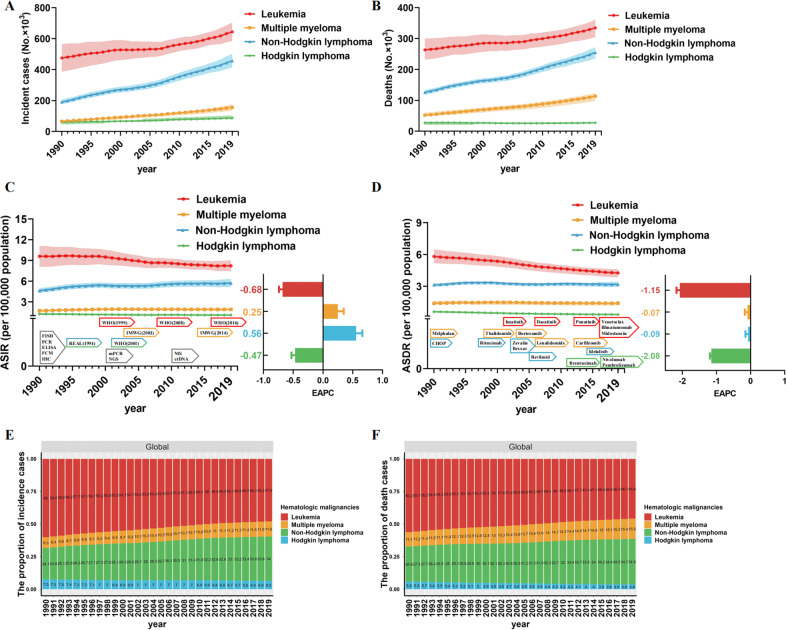
Global trends in incidence and death for hematological malignancies from 1990 to 2019. **A** The new cases of hematological malignancies from 1990 to 2019. **B** The number of deaths due to hematological malignancies from 1990 to 2019. **C** ASIR and EAPC in hematological malignancies over the last 30 years. **D** ASDR and EAPC in hematological malignancies over the last 30 years. **E** The proportion of new cases of hematological malignancies from 1990 to 2019. **F** The proportion of deaths of hematological malignancies from 1990 to 2019. *ASIR* age-standardized incidence rate, *ASDR* age-standardized death rate, *EAPC* estimated annual percentage change, *FISH* fluorescence in situ hybridization, *mPCR* multiplex polymerase chain reaction, *ELISA* enzyme-linked immunosorbent assay, *FCM* flow cytometry, *IHC* immunohistochemical, *NGS* Next-generation sequencing, *MS* mass spectrum, *ctDNA* circulating tumor DNA, *WHO* World Health Organization, *IMWG* International Myeloma Working Group, *REAL* Revised European American Lymphoma, *CHOP* cyclophosphamide, doxorubicin, vincristine, and prednisone.

Globally, the incident cases, deaths, and their change trends with hematologic malignancies from 1990 and 2019 are presented in Table 1. In 2019, the incident cases of leukemia, MM, NHL, and HL increased to 643.58 thousand, 155.69 thousand, 457.08 thousand, and 87.51 thousand, respectively, while the number of deaths increased to 334.59 thousand, 113.47 thousand, 254.61 thousand, and 27.55 thousand, respectively (Fig. 1A, B). Although the number was almost two to three times when since 1990, the ASDR for all hematologic malignancies is on a declining trend, while the ASIR is relatively stable. According to 2019 data, the hematologic malignancies with the highest incidence and mortality rate is leukemia (Fig. 1C, D). The global incidence trends of four major hematologic malignancies were essentially different during 1990–2019. The ASIR of leukemia and HL were 8.22 and 1.1 per 100,000 population in 2019, respectively, and the EAPC exhibited a decreasing tendency (−0.68 and −0.47). The ASIR of MM and NHL was 1.92 and 5.73 per 100,000 population in 2019, respectively, and the EAPC illustrated an increasing trend (0.25 and 0.56). However, the ASDR of leukemia, MM, NHL, and HL were 4.26, 1.42, 3.19, and 0.34 per 100,000 population in 2019, respectively, with a significant downward trend in EAPC (−1.15, −0.07, −0.09 and −2.08).

**Table 1:** The global incident cases, deaths, and their change trends of hematologic malignancies from 1990 to 2019.

Types of hematologic malignancies	Incidence (95% UI)				1990–2019	Deaths (95% UI)				1990–2019
	1990		2019			1990		2019		
	Incident cases No. × 10^3^	ASR per 100,000	Incident cases No. × 10^3^	ASR per 100,000	EAPC No. (95%CI)	Deaths No. × 10^3^	ASR per 100,000	Deaths No. × 10^3^	ASR per 100,000	EAPC No. (95%CI)
Leukemia	474.92 (388.56 to 560.55)	9.6 (8.14 to 11.02)	643.58 (586.98 to 699.73)	8.22 (7.5 to 8.94)	−0.68 (−0.74 to −0.62)	263.26 (233.66 to 298.7)	5.82 (5.25 to 6.44)	334.59 (306.82 to 360.21)	4.26 (3.91 to 4.58)	−1.15 (−1.19 to −1.11)
Acute myeloid leukemia	63.82 (56.66 to 75.53)	1.42 (1.28 to 1.64)	124.33 (108.51 to 135.85)	1.58 (1.38 to 1.73)	0.47 (0.41 to 0.54)	48.19 (43.33 to 56.61)	1.1 (1 to 1.27)	94.06 (82.65 to 101.44)	1.19 (1.04 to 1.28)	0.35 (0.3 to 0.39)
Acute lymphoid leukemia	66.81 (52.91 to 88.25)	1.23 (0.99 to 1.58)	153.32 (128.58 to 170.55)	1.96 (1.64 to 2.17)	1.61 (1.52 to 1.7)	41.24 (32.02 to 56.59)	0.76 (0.61 to 1.02)	47.65 (39.37 to 53)	0.63 (0.52 to 0.7)	−0.57 (−0.65 to −0.49)
Chronic myeloid leukemia	42.7 (34.52 to 56.19)	0.96 (0.82 to 1.19)	65.8 (59.45 to 74.13)	0.83 (0.75 to 0.94)	−1.04 (−1.31 to −0.77)	31.07 (25 to 41.33)	0.71 (0.6 to 0.89)	29.93 (27.01 to 33.48)	0.38 (0.34 to 0.42)	−2.55 (−2.68 to −2.41)
Chronic lymphoid leukemia	40.54 (37.12 to 42.75)	1.09 (1 to 1.14)	103.47 (93.46 to 118.94)	1.28 (1.16 to 1.48)	0.47 (0.33 to 0.6)	21.55 (19.81 to 23.03)	0.62 (0.56 to 0.66)	44.61 (40.39 to 50.07)	0.57 (0.51 to 0.64)	−0.34 (−0.42 to −0.26)
Other leukemia	261.06 (170.27 to 329.46)	4.9 (3.39 to 6.03)	196.66 (170.77 to 224.15)	2.57 (2.23 to 2.92)	−2.43 (−2.52 to −2.34)	121.21 (91.37 to 142.96)	2.62 (2.08 to 3.02)	118.35 (102.75 to 132.39)	1.51 (1.31 to 1.69)	−2.09 (−2.18 to −2)
Multiple myeloma	65.94 (60.78 to 74.06)	1.73 (1.59 to 1.93)	155.69 (136.59 to 172.58)	1.92 (1.68 to 2.12)	0.25 (0.15 to 0.35)	51.86 (47.71 to 58.98)	1.4 (1.28 to 1.58)	113.47 (99.53 to 121.74)	1.42 (1.24 to 1.52)	−0.07 (−0.15 to 0.01)
Non-Hodgkin lymphoma	190.73 (179.03 to 203.62)	4.65 (4.37 to 4.93)	457.08 (416.89 to 498.78)	5.73 (5.21 to 6.25)	0.56 (0.45 to 0.66)	126.08 (119.77 to 131.98)	3.15 (3 to 3.29)	254.61 (237.71 to 270.35)	3.19 (2.98 to 3.39)	−0.09 (−0.17 to −0.02)
Hodgkin lymphoma	59.69 (48.25 to 64.24)	1.26 (1.02 to 1.35)	87.51 (77.94 to 101.43)	1.1 (0.98 to 1.27)	−0.47 (−0.53 to −0.41)	27.6 (21.66 to 30.23)	0.61 (0.48 to 0.66)	27.55 (23.68 to 31.81)	0.34 (0.29 to 0.4)	−2.08 (−2.17 to −1.98)

*ASR* age-standardized rate, *EAPC* estimated annual percentage change, *UI* uncertainty interval, *CI* confidence interval.

Moreover, we analyzed global trends in the proportion of new cases and deaths associated with all hematologic malignancies. Leukemia has always accounted for the largest proportion of new cases, but from 1990 to 2019 the proportion has been declining (47.9% in 2019), and leukemia has been less than half of the hematological malignancies since 2012. Compared to that, the proportion of new cases of MM and lymphoma increased (Fig. 1E). In terms of deaths, the trend in the proportion is similar to that of new cases (45.8% in 2019), but since 2007 leukemia has accounted for less than half of the deaths of hematological malignancies (Fig. 1F). For specific leukemia subtypes, the incident cases of AML, ALL, CML, and CLL increased to 124.33 thousand, 153.32 thousand, 65.8 thousand, and 103.47 thousand in 2019, respectively, while the proportion of other subtypes of leukemia decreased significantly due to the precision of classification in the past three decades (Fig. S1A, B). The number of deaths from AML, ALL, and CLL increased except for CML (Fig. S1C, D). From 1990 to 2019, CML was the type of leukemia with a decreased AISR (EAPC = −1.04) (Fig. S1E), while AML was the type of leukemia with an increased ADSR (EAPC = 0.35) (Fig. S1F).

### Global burden of hematologic malignancies by gender and age

**Fig. 2:**
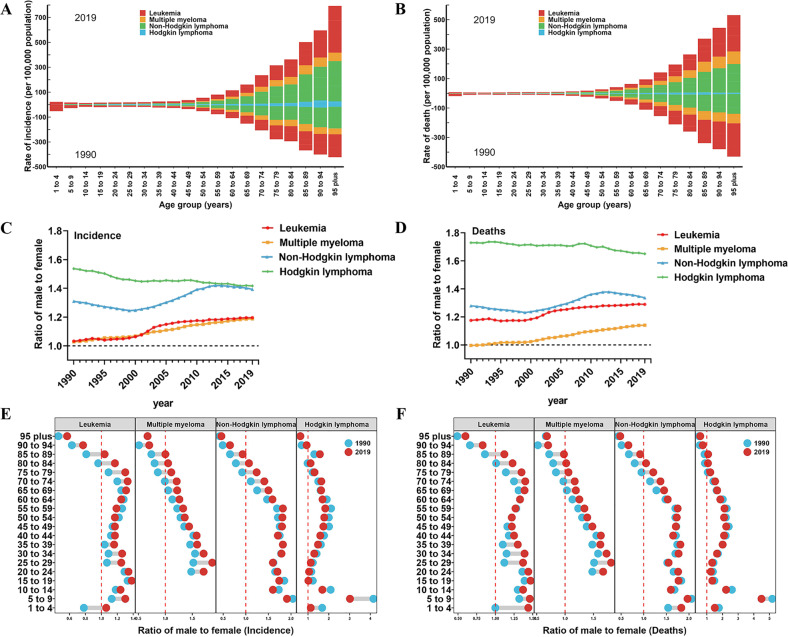
Global incidence and death of hematological malignancies by age and sex. **A** Global hematological malignancies incidence rates by age for both sexes combined in 1990 and 2019. **B** Global hematological malignancies deaths by age for both sexes combined in 1990 and 2019. For each group, the below column shows case data in 1990 and the above column shows data in 2019. **C** The sex ratio of hematological malignancies incident cases from 1990 to 2019 by four causes. **D** The sex ratio of hematological malignancies deaths from 1990 to 2019 by four causes. **E** The ratio of male to female in global hematological malignancies incident cases by age in 1990 and 2019. **F** The ratio of male to female in global hematological malignancies deaths by age in 1990 and 2019.

The age distribution of the population can provide evidence for primary prevention by reflecting the health burden of different age groups. People over the age of 70 are at high risk of developing hematologic malignancies, about 80% of all age groups (Fig. 2A, B). Among all age groups, the highest incidence rates of leukemia, MM, NHL, and HL were 95 plus group, 85 to 89 group, 95 plus group, and 90 to 94 group, respectively, with rates of 116.18, 23.55, 103.62, and 10.29 per 100,000 population in 2019 (Fig. 2A). The highest death rates of leukemia, MM, NHL, and HL were 95 plus group, 90 to 94 group, 95 plus group, and 85 to 89 group, respectively, with rates of 75.81, 24.71, 62.18, and 1.96 per 100,000 population in 2019 (Fig. 2B). In terms of gender, the incidence and death of hematologic malignancies are generally higher in males than in females globally (Fig. S2). The male-to-female ratio for leukemia, MM, and NHL continued to increase from 1990 to 2019 (Fig. 2C, D). The increasing male-to-female ratio may be caused by hormonal, genetic, and environmental factors and requires further research. We show the contribution of different age groups and different genders to hematologic malignancies in 1990 and 2019, not all age groups are more male than female. In most age groups of 1 to 4 and above 75, the proportion of females is larger than males (Fig. 2E, F).

For specific leukemia subtypes, the highest incidence rates of AML, ALL, CML, and CLL in 2019 were 95 plus group, 70 to 74 group, 90 to 94 group, and 95 plus group, respectively, with rates of 56.31, 4.80, 14.69, and 25.76 per 100,000 population (Fig. S3A). The highest death rates of AML, ALL, CML, and CLL were 90 to 94 group, 85 to 89 group, 95 plus group, and 95 plus group, respectively, with rates of 13.24, 2.03, 5.19, and 24.61 per 100,000 population in 2019 (Fig. S3B). Regarding gender, all subtypes of leukemia were male with higher morbidity and mortality (Fig. S3C, D), but there were slight differences between different age groups (Fig. S4).

### Leukemia burden in different regions and countries

**Fig. 3:**
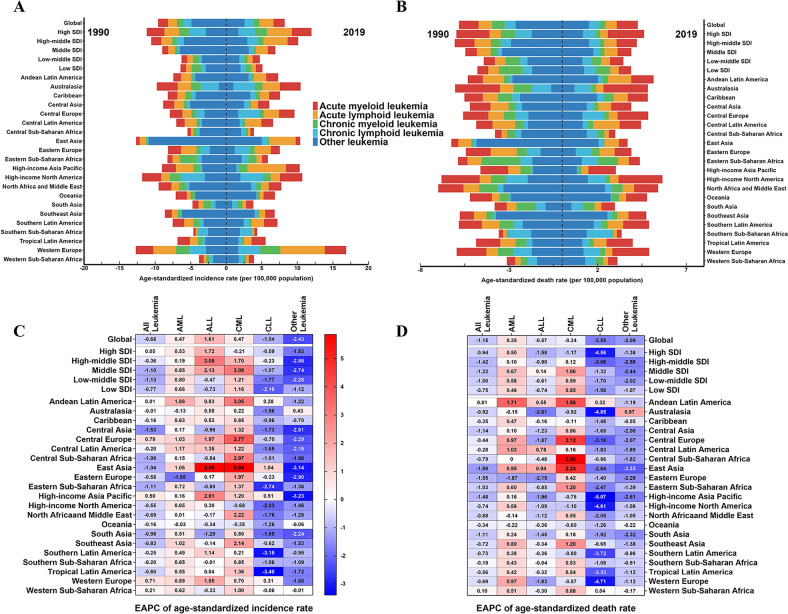
The global trends in leukemia by five subtypes and regions. **A** The ASIR of leukemia at a regional level in 1990 and 2019. **B** The ASDR of leukemia at a regional level in 1990 and 2019. **C** The EAPC in ASIR of leukemia from 1990 to 2019, by subtypes and by regions, for both sexes, combined. **D** The EAPC in ASDR of leukemia from 1990 to 2019, by subtypes and by regions, for both sexes, combined. Blue indicates a downward trend and Red indicates an upward trend. *AML* acute myeloid leukemia, *ALL* acute lymphoid leukemia, *CML* chronic myeloid leukemia, *CLL* chronic lymphoid leukemia, *ASIR* age-standardized incidence rate, *ASDR* age-standardized death rate, *EAPC* estimated annual percentage change.

**Fig. 4:**
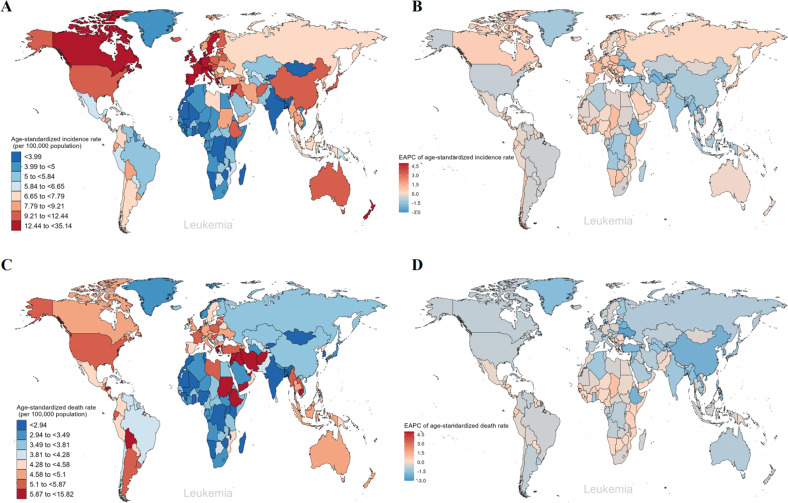
The global trends of leukemia by countries and territories. **A** The ASIR of leukemia in 2019. **B** The EAPC in ASIR of leukemia from 1990 to 2019. **C** The ASDR of leukemia in 2019. **D** The EAPC in ASDR of leukemia from 1990 to 2019. *ASIR* age-standardized incidence rate, *ASDR* age-standardized death rate, *EAPC* estimated annual percentage change.

With regard to the SDI regions, incident cases of leukemia increased across the five SDI regions. However, the ASIR decreased in the low, low-middle, middle, and high-middle SDI regions from 1990 to 2019. The highest ASIR was in the high SDI regions and was on the rise, with an EAPC of 0.05 (Fig. S5A and Table S3). Of the leukemia subtypes, AML, ALL, CML, and CLL have the highest ASIR in the high SDI regions (Fig. S5B–F). For geographical regions, the highest ASIR of leukemia in 2019 appeared in Western Europe (16.87 per 100,000 population), North America (10.69 per 100,000 population), and Australasia (10.46 per 100,000 population). The regions with the lowest ASIR were South Asia (3.81 per 100,000 population), Central Sub-Saharan Africa (3.89 per 100,000 population), and Western Sub-Saharan Africa (3.9 per 100,000 population) (Fig. 3A and Table S3). The regions with the largest increases in the ASIR were Central Europe (EAPC = 0.79), Western Europe (EAPC = 0.71), and Asia Pacific (EAPC = 0.5). In contrast, the regions with the largest declines were Central Asia (EAPC = −1.53), Eastern Sub-Saharan Africa (EAPC = −1.11), and Central Sub-Saharan Africa (EAPC = −1.09) (Fig. 3C and Table S3). Among the 204 countries, Qatar, United Arab Emirates, and Cyprus had the most dramatic increases in leukemia incident cases (percent change: 300% to 500%), while the Republic of Moldova, Georgia, and the Democratic People’s Republic of Korea had the most significant declines (percent change: −60% to −40%) (Fig. S7A and Table S7). Extremely high levels of ASIR were observed in San Marino, far higher than in any other country (35.14 per 100,000 population) (Fig. 4A). On the other hand, the lowest ASIR was found in Palau, with 2.97 per 100,000 population in 2019 (Table S7). In addition, the Cyprus (EAPC = 3.01) and Republic of Moldova (EAPC = −2.37) have the most significant increasing and decreasing trends respectively in the ASIR of leukemia (Fig. 4B).

Notably, the leukemia of ASDR showed a significant decline over three decades in all five SDI regions, most notably in the high-middle SDI regions, with an EAPC of −1.42 (Fig. S6A and Table S3). Of the leukemia subtypes, the ASDR of AML and CLL was the highest in the high SDI regions, the ASDR of CML was the highest in the low SDI regions, and the ASDR of ALL changed from the highest in high-middle SDI to middle SDI from 1990 to 2019 (Fig. S6B–F). For geographical regions, the highest ASDR of leukemia in 2019 was observed in North America (5.65 per 100,000 population), North Africa and the Middle East (5.41 per 100,000 population), and Andean Latin America (5.15 per 100,000 population). The regions with the lowest ASDR were South Asia (2.98 per 100,000 population), Central Sub-Saharan Africa (2.99 per 100,000 population), and Asia Pacific (3.02 per 100,000 population) (Fig. 3B and Table S3). Almost all regions showed a downward trend in ASDR, with the largest declines in East Asia (EAPC = −1.99), Eastern Europe (EAPC = −1.55), and the Asia Pacific (EAPC = −1.48) (Fig. 3D and Table S3). Among the 204 countries, the United Arab Emirates and Qatar had the most pronounced increases in leukemia deaths (percent change: 350% to 500%), while the Republic of Moldova, Ukraine, and Georgia had the most significant declines (percent change: −50% to −30%) (Fig. S7B and Table S7). The two countries with higher ASDR were found to be the Syrian Arab Republic and Afghanistan, with rates of 10 to 20 per 100,000 population in 2019 (Fig. 4C). Lesotho (EAPC = 2.01) and Ukraine (EAPC = −2.48) have the most significant increasing and decreasing trends respectively in the ASDR of leukemia (Fig. 4D).

For the correlation analysis of EAPC influencing factors in leukemia, we found that EAPC (in ASIR) was not correlated with ASIR (in 1990) and SDI (in 2019), but EAPC (in ASDR) was significantly negatively connected with ASDR in 1990 (*P* < 0.001, *ρ* = −0.457). It is also significantly negatively correlated with SDI (in 2019) (*P* < 0.001, ρ = −0.368). Interestingly, ASIR demonstrated a clear positive correlation with SDI levels (*P* < 0.001, *ρ* = 0.781) (Fig. S8). Although the data show a trend towards a higher incidence of leukemia and lower mortality in regions with higher economic levels, it is also possible that this is due to improved reporting or earlier diagnosis.

### Multiple myeloma burden in different regions and countries

**Fig. 5:**
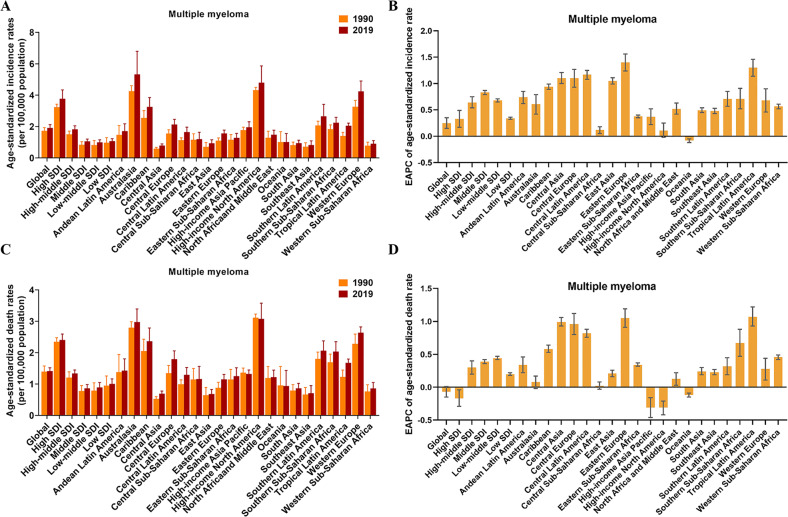
The global trends in multiple myeloma by regions. **A** The ASIR of multiple myeloma at a regional level in 1990 and 2019. **B** The EAPC in ASIR of multiple myeloma from 1990 to 2019 by region, for both sexes, combined. **C** The ASDR of multiple myeloma at a regional level in 1990 and 2019. **D** The EAPC in ASDR of multiple myeloma from 1990 to 2019 by region, for both sexes, combined. *ASIR* age-standardized incidence rate, *ASDR* age-standardized death rate, *EAPC* estimated annual percentage change.

**Fig. 6:**
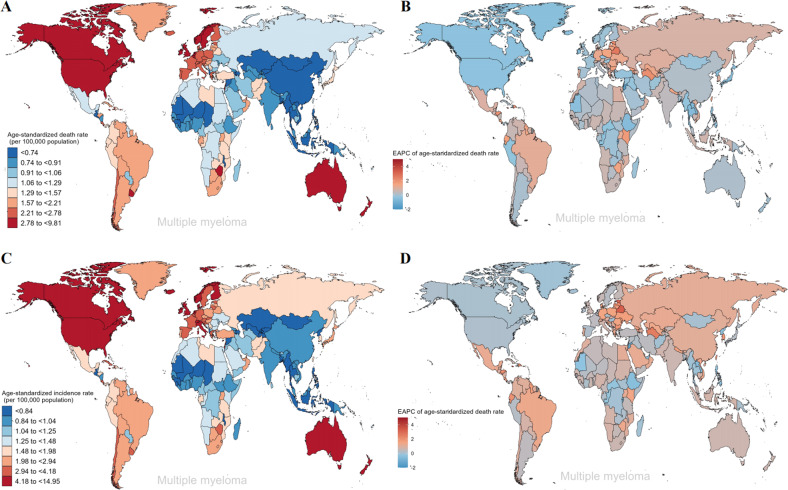
The global trends of multiple myeloma by countries and territories. **A** The ASIR of multiple myeloma in 2019. **B** The EAPC in ASIR of multiple myeloma from 1990 to 2019. **C** The ASDR of multiple myeloma in 2019. **D** The EAPC in ASDR of multiple myeloma from 1990 to 2019. *ASIR* age-standardized incidence rate, *ASDR* age-standardized death rate, *EAPC* estimated annual percentage change.

During the period from 1990 to 2019, the number of MM cases increased across the five SDI regions. As for ASIR, ASIR in high SDI regions remained the highest, while that in low-middle SDI and low SDI regions were consistently low (Figs. 5A and S9A). The ASIR of MM increased across all SDI regions with the largest increase in middle SDI regions (EAPC = 0.83), while the ASIR was a slow increase in high SDI regions (EAPC = 0.33) (Fig. 5B and Table S4). For geographical regions, the highest ASIR of MM in 2019 was observed in Australasia (5.33 per 100,000 population), North America (4.8 per 100,000 population), and Western Europe (4.24 per 100,000 population). The regions with the lowest ASIR were Central Asia (0.8 per 100,000 population), Southeast Asia (0.82 per 100,000 population), and Western Sub-Saharan Africa (0.91 per 100,000 population) (Fig. 5A and Table S4). The regions with the largest increases in the ASIR were Eastern Europe (EAPC = 1.4), Tropical Latin America (EAPC = 1.3), and Central Latin America (EAPC = 1.17). In contrast, the region with the largest decline was Oceania (EAPC = −0.08) (Fig. 5B and Table S4). Among the 204 countries, Qatar and the United Arab Emirates had the most significant increases in MM incident cases (percent change: 800% to 1000%), while Tokelau and Niue had the most significant declines (percent change: −1% to −10%) (Fig. S10A and Table S7). Extremely high levels of ASIR were observed in Monaco, far higher than in other countries (14.95 per 100,000 population) (Fig. 6A). On the other hand, the lowest ASIR was found in Kyrgyzstan, with 0.62 per 100,000 population in 2019 (Table S7). In addition, Jamaica (EAPC = 4.15) and Northern Mariana Islands (EAPC = −1.29) have the most significant increasing and decreasing trends respectively in the ASIR of MM (Fig. 6B).

Although regions with high SDI also consistently had the highest ASDR from 1990 to 2019, the overall trend was downward, with an EAPC of −0.17 (Figs. 5C, D and S9B). And the increasing trend in ASDR was primarily concentrated in low SDI (EAPC = 0.2), low-middle SDI (EAPC = 0.44), middle SDI (EAPC = 0.39), and high-middle SDI (EAPC = 0.3) regions over this period (Fig. 5D). For geographical regions, the highest ASDR of MM in 2019 was observed in North America (3.07 per 100,000 population), Australasia (2.97 per 100,000 population), and Western Europe (2.64 per 100,000 population). The regions with the lowest ASDR were East Asia (0.68 per 100,000 population), Central Asia (0.69 per 100,000 population), and Southeast Asia (0.71 per 100,000 population) (Fig. 5C and Table S4). Almost all regions demonstrated an increasing tendency in ASDR, with the largest increases in Tropical Latin America (EAPC = 1.07), Eastern Europe (EAPC = 1.05), and Central Asia (EAPC = 0.99) (Fig. 5D and Table S4). Among the 204 countries, the United Arab Emirates and Qatar had the most pronounced increases in MM deaths (percent change: 650% to 900%), while Tokelau and Niue had the most significant declines (percent change: −15% to −9%) (Fig. S10B and Table S7). The two countries with higher ASDR were found to be Monaco and Barbados, with rates of 6 to 10 per 100,000 population in 2019 (Fig. 6C). Jamaica (EAPC = 3.96) and Jordan (EAPC = −1.61) have the most significant increasing and decreasing trends respectively in the ASDR of MM (Fig. 6D).

For the correlation analysis of EAPC influencing factors in MM, the EAPC (in ASIR) was positively correlated with the SDI (in 2019) (*P* = 0.007, *ρ* = 0.19), but not the ASIR (in 1990) (*P* = 0.273, *ρ* = −0.077). Although EAPC (in ASDR) was not significantly associated with ASDR (in 1990) and SDI (in 2019), we found that both ASIR and ASDR were higher in regions with higher SDI levels (Fig. S11), suggesting that areas with higher SDI levels should better perform preventive healthcare and promotion for MM.

### Lymphoma burden in different regions and countries

**Fig. 7:**
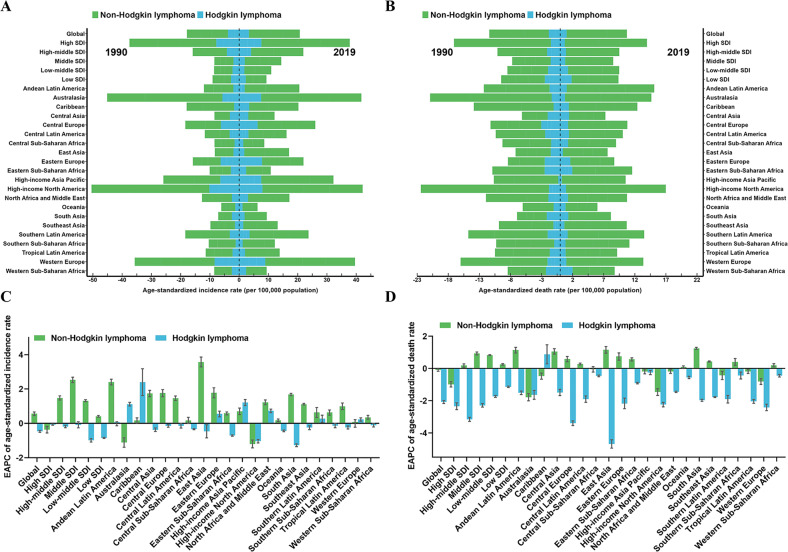
The global trends in lymphoma by regions. **A** The ASIR of non-Hodgkin lymphoma and Hodgkin lymphoma at a regional level in 1990 and 2019. **B** The ASDR of non-Hodgkin lymphoma and Hodgkin lymphoma at a regional level in 1990 and 2019. **C** The EAPC in ASIR of non-Hodgkin lymphoma and Hodgkin lymphoma from 1990 to 2019 by regions, for both sexes, combined. **D** The EAPC in ASDR of non-Hodgkin lymphoma and Hodgkin lymphoma from 1990 to 2019 by regions, for both sexes, combined. *ASIR* age-standardized incidence rate, *ASDR* age-standardized death rate, *EAPC* estimated annual percentage change.

**Fig. 8:**
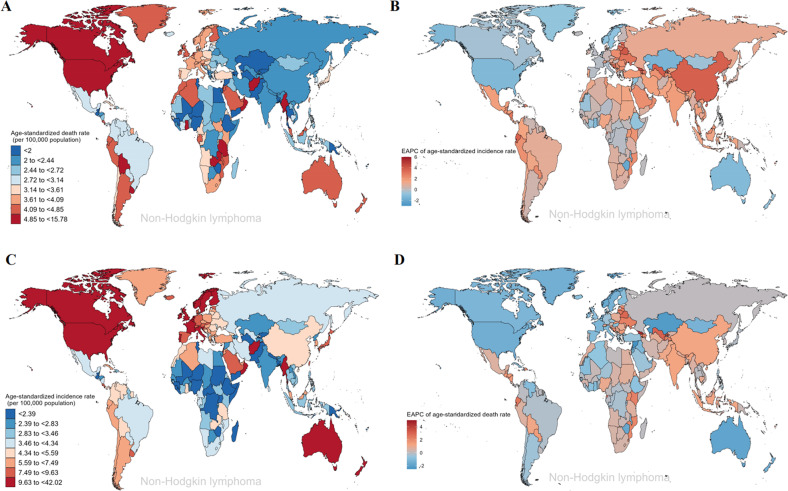
The global trends of Non-Hodgkin lymphoma by countries and territories. **A** The ASIR of Non-Hodgkin lymphoma in 2019. **B** The EAPC in ASIR of Non-Hodgkin lymphoma from 1990 to 2019. **C** The ASDR of Non-Hodgkin lymphoma in 2019. **D** The EAPC in ASDR of Non-Hodgkin lymphoma from 1990 to 2019. *ASIR* age-standardized incidence rate, *ASDR* age-standardized death rate, *EAPC* estimated annual percentage change.

Lymphoma mainly consists of NHL and HL, with NHL being consistently 3–5 times more common than HL. For SDI regions, ASIR in high SDI and high-middle SDI regions remained the highest in NHL and HL (Fig. S12A, B). The ASIR in high SDI regions was 9.93 per 100,000 population for NHL and 2.51 per 100,000 population for HL (Fig. 7A). For geographical regions, the highest and lowest ASIR of NHL in 2019 were observed in Australasia (11.31 per 100,000 population) and Oceania (1.71 per 100,000 population) (Fig. 7A and Table S5), the highest and lowest ASIR of HL in 2019 were observed in Western Europe (2.96 per 100,000 population) and Oceania (0.39 per 100,000 population) (Fig. 7A and Table S6). Except for North America, Australasia, and Western Europe, the ASIR of NHL showed an increasing trend in all regions, with the largest increases in East Asia (EAPC = 3.57), Andean Latin America (EAPC = 2.41), and Eastern Europe (EAPC = 1.77) (Fig. 7C). The regions with the largest increasing trend in the ASIR of HL were the Caribbean (EAPC = 2.4), Asia Pacific (EAPC = 1.21), and Australasia (EAPC = 1.11) (Fig. 7C). Among the 204 countries, Qatar and the United Arab Emirates had the most pronounced increases in NHL incident cases (percent change: 700% to 1000%), while Malawi and Zimbabwe had the most modest increases (percent change: 5% to 10%) (Fig. S13A and Table S7). The Qatar and Republic of Korea had the most pronounced increases in HL incident cases (percent change: 700% to 1000%), while Georgia and Hungary had the most significant declines (percent change: −50% to −30%) (Fig. S14A and Table S7). The two countries with higher ASIR of all lymphoma types were found to be Monaco and San Marino (Figs. 8A and S14B). In addition, Georgia (EAPC = 4.7) and Zimbabwe (EAPC = −2.0) have the most significant increasing and decreasing trends respectively in the ASIR of NHL (Fig. 8B), Cuba (EAPC = 5.86) and Equatorial Guinea (EAPC = −2.42) has the most significant increasing and decreasing trends respectively in the ASIR of HL (Fig. S14C).

Regarding the death status of lymphoma by SDI regions, high SDI regions consistently have the highest ASDR of NHL from 1990 to 2019, and low SDI regions consistently have the highest ASDR of HL (Fig. S12C, D). For geographical regions, the highest and lowest ASDR of NHL in 2019 was observed in North America (5.27 per 100,000 population) and Oceania (1.7 per 100,000 population) (Fig. 7B and Table S5), the highest and lowest ASDR of HL in 2019 were appeared in Western Sub-Saharan Africa (0.66 per 100,000 population) and Asia Pacific (0.08 per 100,000 population) (Fig. 7B and Table S6). The regions with the largest increasing trend in the ASDR of NHL were South Asia (EAPC = 1.25), East Asia (EAPC = 1.16), and Andean Latin America (EAPC = 1.14) (Fig. 7D). Except for the Caribbean (EAPC = 0.88), the ASDR of HL demonstrated a decreasing tendency in all regions, with the largest decreases in East Asia (EAPC = −4.68), Central Europe (EAPC = −3.4), and Western Europe (EAPC = −2.42) (Fig. 7D). Among the 204 countries, Qatar and the United Arab Emirates had the most significant increases in all lymphoma types deaths (Figs. S13B, S14D, and Table S7). The two countries with higher ASDR of NHL were found to be Monaco and Afghanistan, with rates of 14 to 16 per 100,000 population in 2019 (Fig. 8C). The two countries with higher ASDR of HL were found to be Pakistan and Nigeria, with rates of 1.1 to 1.3 per 100,000 population in 2019 (Fig. S14E). In addition, Georgia (EAPC = 4.54) and Syrian Arab Republic (EAPC = −2.46) has the most significant increasing and decreasing trends respectively in the ASDR of NHL (Fig. 8D), Cuba (EAPC = 3.96) and China (EAPC = −4.77) has the most significant increasing and decreasing trends respectively in the ASDR of HL (Fig. S14F). For Qatar and the United Arab Emirates, which had the largest fluctuations in the number of cases, further analysis showed that the huge changes in population structure were an important reason for the fluctuations in the number of cases (Fig. S21).

For the correlation analysis of EAPC influencing factors in lymphoma, the EAPC was negatively connected with ASIR (*P* < 0.001, *ρ* = −0.342) and ASDR (*P* < 0.001, *ρ* = −0.444) in NHL (Fig. S15), the EAPC was negatively associated with ASDR (*P* < 0.001, *ρ* = −0.417) and SDI (in 2019) (*P* < 0.001, *ρ* = −0.431) in HL (Fig. S16). In addition, we found that in areas with higher SDI levels, ASIR and ASDR in NHL were higher, ASIR in HL was higher, and only ASDR in HL showed a decrease, suggesting that areas with higher SDI levels should better perform preventive healthcare and promotion for NHL.

### Contribution of high BMI burden to death from hematologic malignancies

Leukemia was the leading cause of death for hematologic malignancies at the global and regional scales, followed by NHL, MM, and HL, accordingly for 45.8%, 34.9%, 15.5%, and 3.8% of total deaths in 2019, respectively (Fig. S17A). High BMI is the main cause of diverse metabolic disorders, the public health issues associated with high BMI have been recognized for a long time and are becoming increasingly important [22–25]. Our analysis found that the proportion of mortality risk attributable to high BMI in 2019 was significantly higher in all regions than in 1990. The proportion of deaths attributed to high BMI had a strong positive correlation with SDI levels, and the proportion of leukemia, MM, and lymphoma in low SDI regions was 2.4%, 3.7%, and 2.9%, respectively, in 2019, lower than in other SDI regions (Fig. S17B). Meanwhile, the region with high SDI consistently had the highest risk of death attributable to high BMI from 1990 to 2019 (Fig. S17C, E). Regarding the effect of gender and age, the proportion of deaths attributed to high BMI was higher in females with leukemia and MM in all regions (Fig. S19A). Globally, the highest risk of death from hematologic malignancies (including leukemia, MM, and NHL) attributed to high BMI in 2019 was in the 50 to 69 age group (Fig. S19B). Table S8 shows the details of the risk of death attributed to high BMI at the national level, Congo males (15.9%), Canadian males (15.2%), and Colombia males (17.6%) account for the highest percentages of leukemia, MM, and NHL respectively, in 2019.

### Contribution of occupational carcinogens burden to death from leukemia

Exposure to benzene and formaldehyde in occupational carcinogens is a known risk factor for leukemia [26]. Globally, the proportion of leukemia deaths attributable to occupational exposure to benzene and formaldehyde in 2019 was 0.56% and 0.18%, respectively. The proportion of deaths attributed to occupational carcinogens was always the highest in middle SDI regions, and the proportion of deaths from occupational benzene exposure changed from the lowest in the low SDI regions to high SDI regions from 1990 to 2019 (Fig. S18A, B). For all leukemia subtypes, the largest risk of death attributed to occupational exposure to benzene and formaldehyde globally in 2019 was CML. The regions with the highest proportion of leukemia deaths attributable to occupational carcinogens in 2019 were Andean Latin America and Central Latin America (Fig. S18C). Regarding the effect of gender, the proportion of deaths attributable to occupational exposure to benzene in CML and CLL was predominant in males, while AML and ALL were predominant in females, and occupational exposure to formaldehyde was more common in males with all types of leukemia (Fig. S20). Ireland and Dominica are at high risk of occupational exposure to benzene and formaldehyde, the detailed death risk caused by occupational carcinogens for all types of leukemia at the national level is shown in Table S9.

## Discussion

The findings presented in this report provide a systematic understanding of the global burden of hematologic malignancies by region and country from 1990 to 2019. As predicted by previous studies, we found that hematologic malignancies are one of the major causes of the global tumor burden, with leukemia having the highest burden of all types [27]. Globally, the incident cases of hematologic malignancies have been increasing since 1990, reaching 1343.85 thousand in 2019, but the mortality rate for all types of hematologic malignancies has been declining, reflecting years of relentless endeavors for prevention, early detection, and treatment of hematologic malignancies.

The predominance of males in hematologic malignancies has been observed throughout the world, with male-to-female ASIR ratios of 1.3:1 for leukemia, 1.4:1 for MM, 1.6:1 for NHL, and 1.5:1 for HL. Notably, the gender differences varied by age. The male-to-female ratios of hematologic malignancies continue to decline from certain ages until females are dominant, with the peak age for leukemia approaching 70 to 74 years, MM at 25 to 29 years, and lymphoma at 55 to 59 years. Consistent with findings in the literature, the gender difference was evident in incident cases and ASIR for leukemia, MM, and lymphoma [28–30]. During the period from 1990 to 2019, the male-to-female ratio for leukemia, MM, and NHL continued to increase (Fig. 2C, D). This trend may be attributed to various factors, including differences in hormonal and genetic factors between males and females, as well as differences in environmental exposures and lifestyle factors that may increase the risk of developing these malignancies in males compared to females [31]. However, the burden and trend of hematologic malignancies varied among regions. For overall hematologic malignancies, the trends in ASDR remained or decreased stable in high and high-middle SDI regions, although other SDI regions had an increasing trend. This is mainly due to imbalances in socio-economic development, while inadequate investments in healthcare and poor health awareness exacerbate the burden in low SDI regions, and can be improved through global collaborative partnerships [32, 33]. However, ASIR for all types of hematologic malignancies in the high SDI regions has remained the highest over the past three decades.

During the past study period, advances in laboratory techniques have had a significant impact on the diagnosis and management of hematologic malignancies [34, 35]. With the development of new technologies, such as next-generation sequencing, flow cytometry, and molecular genetics, it is now possible to identify specific genetic mutations and biomarkers that can be used to classify and prognosticate hematologic malignancies [36]. This 30-year time series shows significant hematologic malignancies and specific temporal features, and below we consider possible contributing factors to changes in morbidity and mortality. Changes in diagnostic criteria or definitions have a certain impact on the increase or decrease of incidence (Fig. 1C). For example, before 1999, the diagnosis of CML was based on clinical features and the presence of the Philadelphia chromosome. The discovery of the BCR-ABL1 fusion gene provided a more specific diagnostic marker for CML [37]. More cases previously classified as leukemia solely based on morphological diagnosis were excluded without immunophenotypic typing and genetic testing. The new criteria require confirmation of the diagnosis with specific biomarkers. For example, cytoplasmic CD3 or TdT for T-cell ALL, and cytoplasmic CD10 or CD19 for B-cell ALL [38]. This makes the diagnosis of ALL more accurate and specific, reducing the inclusion of cases with overlapping features, such as Burkitt’s lymphoma previously classified as ALL. In 2008, the WHO introduced the concept of recurrent genetic abnormalities as the main diagnostic criteria, leading to the emergence of new subtypes and the reclassification of some previously defined subtypes [39]. This change resulted in an increase in the diagnosis of certain subtypes (such as AML with NPM1 and FLT3-ITD mutations), while other subtypes (such as AML with bone marrow proliferative abnormalities and treatment-related myeloid neoplasms) gained new recognition [40]. Similarly, in 2016, WHO further improved the diagnostic criteria for other subtypes, which have more or less impact on the incidence of leukemia [41]. For MM, the International Myeloma Working Group (IMWG) updated the diagnostic criteria for MM in 2003 to include the use of new laboratory tests such as serum-free light chain assay, and imaging techniques such as magnetic resonance imaging (MRI) and positron emission tomography (PET) to better detect bone lesions [42]. In 2014, IMWG updated the diagnostic criteria for MM again. Before this, patients had to exhibit end-organ damage to be diagnosed [43]. They also introduced a new category called “smoldering MM” for patients with malignant tumor biomarkers but no end-organ damage [44]. The updated criteria allow for earlier detection and identification of patients who may benefit from early treatment, but may also overestimate the incidence and mortality rates of this disease (with over 140,000 cases of MM diagnosed annually since 2015) [45, 46]. Similarly, for lymphoma, the inclusion of immunophenotypic and molecular criteria in the Revised European American Lymphoma (REAL) and WHO classifications may lead to the identification of previously unrecognized lymphoma subtypes, resulting in an overestimation of incidence rates [47, 48].

According to the disease-specific survival rates reported in the Nordic countries [49], the 5-year survival rate for MM patients increased from 30% in 1990 to 60% in 2019, while the 5-year survival rate for AML increased from 10% in 1990 to 35% in 2019, which is due to significant advances in the treatment of hematological malignancies, including the development of new targeted therapies, immunotherapies, and more effective chemotherapy regimens (Fig. 1D). However, in our study, except for leukemia, the mortality rates of other hematologic tumors appear to be stable, which may be due to changes in the world’s population structure over the past 30 years. Unlike survival rates, the population mortality rate is an important indicator for measuring population health status and can help determine trends and patterns in mortality rates.

The age-standardized is a useful measure for comparing disease burden across populations of different sizes, as it adjusts for differences in age distribution [50]. However, in countries with very small populations, the reliability of age-standardized morbidity rates may be limited due to small sample sizes and the potential for random variation. As we found consistently high rates in small countries such as Monaco and San Marino, we again used crude incidence rates to reduce the size effect. We found that the exclusion of population age stratification differences did not significantly affect the overall results (Fig. S22). Nonetheless, available data on the incidence of hematological malignancies in small countries are still limited, and GBD provides the reference available. Therefore, caution should be exercised when comparing the incidence of hematologic malignancies in different populations (the number of cases can be referenced). Additionally, the incidence of a small country can be estimated by extrapolating data from neighboring countries with similar demographics and population characteristics, such as Monaco and San Marino, which belong to the geographical region of Western Europe, a region with a higher incidence of hematologic malignancies. Potential prevention strategies for these countries may include raising awareness and education of risk factors associated with hematologic malignancies, such as exposure to certain chemicals or radiation, as well as lifestyle factors such as smoking and drinking. Our study provides a starting point for further research and discussion, and we hope that our findings will contribute to the development of effective prevention and management strategies for hematologic malignancies.

The unique feature of this study is that we provided a comprehensive overview of the burden of hematologic malignancies according to the most recent national statistics worldwide, but there are some limitations. Similar to issues with many of the diseases from the GBD study, the accuracy of hematological malignancy models depends largely on the quality and quantity of input data. These common deficiencies have been explained in detail in previously published GBD studies [12, 51–53]. In brief, data collected from different regions and countries may vary considerably in terms of quality, comparability, accuracy, and the degree of data missing, which will inevitably lead to some deviation in the estimates, even if the data is adjusted as much as possible using multiple statistical methods. Better reporting quality and earlier diagnosis in developed countries may result in an overestimation of certain disease data. Additionally, underreporting and failure to diagnose can be sources of bias in the registration of hematologic malignancies, particularly in less developed countries with limited clinical hematology, so some estimates of hematologic malignancies may be understated. Despite these limitations, this study is the first comprehensive assessment of global trends in hematological malignancies over three decades at global, regional, and national levels. The results provide a foundation for future research in this area and help identify resources and efforts to improve the quality and comparability of data on hematological malignancies worldwide.

In summary, hematologic malignancies remain a major public health concern globally, and while ASDR is declining globally, they are still on the rise in many countries. The burden of hematologic malignancies is generally higher in men, and this gender gap decreases after peaking at a given age. In addition, the proportion of deaths attributed to high BMI continued to rise across regions over the past three decades, especially in regions with high SDI. In relatively low SDI regions, occupational exposure to benzene and formaldehyde remained a risk factor for leukemia. The demographics of different regions and countries, social and economic factors, and lifestyles all contribute to these differences. This study provides information for analyzing trends in the global burden of disease for specific hematologic malignancies and for developing appropriate policies for these modifiable risk factors.

## Supplementary information

Reproducibility_checklist_by_Springer_nature

STROBE-checklist

Supplementary Material

## Data Availability

The data are available from the Global Burden of Disease Results Tool of the Global Health Data Exchange (http://ghdx.healthdata.org/).
