# Supplementary material for: Global burden of hematologic malignancies and evolution patterns over the past 30 years
Source: Blood Cancer J. 2023 May 17;13(1):82. doi: 10.1038/s41408-023-00853-3 (PMC10188596; doi:10.1038/s41408-023-00853-3)
Supplement: Supplementary file 2 — STROBE-checklist [file 41408_2023_853_MOESM2_ESM.pdf]

STROBE Statement—checklist

"Global burden of hematologic malignancies and evolution patterns over the past 30 years"(23-BCJ-0062RR)   *by Nan Zhang et al*

	Item No.	Recommendation	Page No.	Relevant text from manuscript
**Title and abstract**	1	(*a*) Indicate the study's design with a commonly used term in the title or the abstract	1-2	Title and Abstract
		(*b*) Provide in the abstract an informative and balanced summary of what was done and what was found	2	Abstract
**Introduction**				
Background/rationale	2	Explain the scientific background and rationale for the investigation being reported	4	Paragraph 1-2 of the Introduction
Objectives	3	State specific objectives, including any prespecified hypotheses	4-5	Paragraph 3 of the Introduction
**Methods**				
Study design	4	Present key elements of study design early in the paper	5	Paragraph 1 of the Methods
Setting	5	Describe the setting, locations, and relevant dates, including periods of recruitment, exposure, follow-up, and data collection	5-8	Paragraph 2-4 of the Methods
Participants	6	(*a*) *Cohort study*—Give the eligibility criteria, and the sources and methods of selection of participants. Describe methods of follow-up<br>*Case-control study*—Give the eligibility criteria, and the sources and methods of case ascertainment and control selection. Give the rationale for the choice of cases and controls<br>*Cross-sectional study*—Give the eligibility criteria, and the sources and methods of selection of participants	9	Participants are not applicable and are explained in the last paragraph of the Method
		(*b*) *Cohort study*—For matched studies, give matching criteria and number of exposed and unexposed<br>*Case-control study*—For matched studies, give matching criteria and the number of controls per case	NA	NA
Variables	7	Clearly define all outcomes, exposures, predictors, potential confounders, and effect modifiers. Give diagnostic criteria, if applicable	5,7	Paragraph 1, 4 of the Methods
Data sources/ measurement	8*	For each variable of interest, give sources of data and details of methods of assessment (measurement). Describe comparability of assessment methods if there is more than one group	5-6	Paragraph 2 of the Methods
Bias	9	Describe any efforts to address potential sources of bias	8-9	Paragraph 5 of the Methods
Study size	10	Explain how the study size was arrived at	5	Paragraph 1 of the Methods

Quantitative variables	11	Explain how quantitative variables were handled in the analyses. If applicable, describe which groupings were chosen and why	5-6	Paragraph 2 of the Methods
Statistical methods	12	(*a*) Describe all statistical methods, including those used to control for confounding	8-9	Paragraph 4-5 of the Methods
		(*b*) Describe any methods used to examine subgroups and interactions	6	Paragraph 2 of the Methods
		(*c*) Explain how missing data were addressed	NA	
		(*d*) *Cohort study*—If applicable, explain how loss to follow-up was addressed<br>*Case-control study*—If applicable, explain how matching of cases and controls was addressed<br>*Cross-sectional study*—If applicable, describe analytical methods taking account of sampling strategy	NA	
		(*e*) Describe any sensitivity analyses	NA	
**Results**				
Participants	13*	(a) Report numbers of individuals at each stage of study—eg numbers potentially eligible, examined for eligibility, confirmed eligible, included in the study, completing follow-up, and analysed	NA	
		(b) Give reasons for non-participation at each stage	NA	
		(c) Consider use of a flow diagram	NA	
Descriptive data	14*	(a) Give characteristics of study participants (eg demographic, clinical, social) and information on exposures and potential confounders	9-17	Paragraph 1-2,5-6,8-10,12-13 of the Results
		(b) Indicate number of participants with missing data for each variable of interest	NA	
		(c) *Cohort study*—Summarise follow-up time (eg, average and total amount)	9	Paragraph 1 of the Results
Outcome data	15*	*Cohort study*—Report numbers of outcome events or summary measures over time	NA	
		Case-control study—Report numbers in each exposure category, or summary measures of exposure	NA	
		Cross-sectional study—Report numbers of outcome events or summary measures	NA	
Main results	16	(*a*) Give unadjusted estimates and, if applicable, confounder-adjusted estimates and their precision (eg, 95% confidence interval). Make clear which confounders were adjusted for and why they were included	Table 1	UI, uncertainty interval; CI, confidence interval.
		(*b*) Report category boundaries when continuous variables were categorized	11-12, Figure 2	Paragraph 3-4 of the Results
		(*c*) If relevant, consider translating estimates of relative risk into absolute risk for a meaningful time period	NA	

Continued on next page

Other analyses	17	Report other analyses done—eg analyses of subgroups and interactions, and sensitivity analyses	12-13, 16-17, Figure 3,7	Paragraph 5-6,12-13 of the Results
**Discussion**				
Key results	18	Summarise key results with reference to study objectives	20-21	Paragraph 1 of the Discussion
Limitations	19	Discuss limitations of the study, taking into account sources of potential bias or imprecision. Discuss both direction and magnitude of any potential bias	24-25	Paragraph 6 of the Discussion
Interpretation	20	Give a cautious overall interpretation of results considering objectives, limitations, multiplicity of analyses, results from similar studies, and other relevant evidence	21-24	Paragraph 3-5 of the Discussion
Generalisability	21	Discuss the generalisability (external validity) of the study results	20-21	Paragraph 2 of the Discussion
**Other information**				
Funding	22	Give the source of funding and the role of the funders for the present study and, if applicable, for the original study on which the present article is based	25-26	Data availability statement and Funding

*Give information separately for cases and controls in case-control studies and, if applicable, for exposed and unexposed groups in cohort and cross-sectional studies.

Note: An Explanation and Elaboration article discusses each checklist item and gives methodological background and published examples of transparent reporting. The STROBE checklist is best used in conjunction with this article (freely available on the Web sites of PLoS Medicine at http://www.plosmedicine.org/, Annals of Internal Medicine at http://www.annals.org/, and Epidemiology at http://www.epidem.com/). Information on the STROBE Initiative is available at www.strobe-statement.org.
