# Supplementary material for: Global burden of hematologic malignancies and evolution patterns over the past 30 years
Source: Blood Cancer J. 2023 May 17;13(1):82. doi: 10.1038/s41408-023-00853-3 (PMC10188596; doi:10.1038/s41408-023-00853-3)
Supplement: Supplementary file 3 — Supplementary Material [file 41408_2023_853_MOESM3_ESM.pdf]

Supplementary material

Global burden of hematologic malignancies and evolution patterns over the past 30 years

Catalogue **1**
Supplementary Figure S1. Global trends in incidence and death for leukemia from 1990 to 2019 by five causes. 1
Supplementary Figure S2. Global incidence and death of hematological malignancies by sex from 1990 to 2019. 2
Supplementary Figure S3. Global incidence and death of leukemia by age and sex. 3
Supplementary Figure S4. Sex differences in leukemia by age and five causes. 4
Supplementary Figure S5. The ASIR of leukemia in SDI regions from 1990 to 2019. 5
Supplementary Figure S6. The ASDR of leukemia in SDI regions from 1990 to 2019. 6
Supplementary Figure S7. Changes in the number of leukemia by countries and territories. 7
Supplementary Figure S8. The correlation between EAPC and age-standardized rate in leukemia. 8
Supplementary Figure S9. The ASIR and ASDR of multiple myeloma in SDI regions from 1990 to 2019. 9
Supplementary Figure S10. Changes in the number of multiple myeloma by countries and territories. 10
Supplementary Figure S11. The correlation between EAPC and age-standardized rate in multiple myeloma. 11
Supplementary Figure S12. The ASIR and ASDR of lymphoma in SDI regions from 1990 to 2019. 12
Supplementary Figure S13. Changes in the number of non-Hodgkin lymphoma by countries and territories. 13
Supplementary Figure S14. The global trends of Hodgkin lymphoma by countries and territories. 14
Supplementary Figure S15. The correlation between EAPC and age-standardized rate in non-Hodgkin lymphoma. 15
Supplementary Figure S16. The correlation between EAPC and age-standardized rate in Hodgkin lymphoma. 16
Supplementary Figure S17. Contribution of death causes to hematological malignancies by regions in 1990 and 2019. 17
Supplementary Figure S18. Predominant contribution of occupational carcinogens to leukemia deaths by regions. 18
Supplementary Figure S19. Predominant contribution of high body-mass index to hematological malignancies deaths by region, sex, and age groups. 19
Supplementary Figure S20. Predominant contribution of occupational carcinogens in hematological malignancies deaths by region, sex, and age groups. 20
Supplementary Figure S21. Analysis of population, number of cases and incidence rates in two countries (Qatar and the United Arab Emirates) with large fluctuations in cases from 1990 to 2019. 21
Supplementary Figure S22. Crude incidence rates for the 10 countries with the largest age-standardized incidence rates. 22
Supplementary Table S1. 21 geographic regions in 204 countries around the world. 23
Supplementary Table S2. Global age-standardised SEVs for both sexes combined in 1990 and 2019, and annualised rate of change between 1990 and 2019. 25
Supplementary Table S3. The global incident cases, deaths, and their change trends of leukemia from 1990 to 2019. 29
Supplementary Table S4. The global incident cases, deaths, and their change trends of multiple myeloma from 1990 to 2019. · 32
Supplementary Table S5. The global incident cases, deaths, and their change trends of Non-Hodgkin lymphoma from 1990 to 2019. 35
Supplementary Table S6. The global incident cases, deaths, and their change trends of Hodgkin lymphoma from 1990 to 2019.38
Supplementary Table S7. The change of hematological malignancies between 1990 and 2019 at national level, both sexes. 41
Supplementary Table S8. The percent change in death cases attributable to high body-mass index between 1990 and 2019 at national level. 122
Supplementary Table S9. The percent change in death cases attributable to occupational carcinogens between 1990 and 2019 at national level. 151

Supplementary Figure S1. Global trends in incidence and death for leukemia from 1990 to 2019 by five causes.

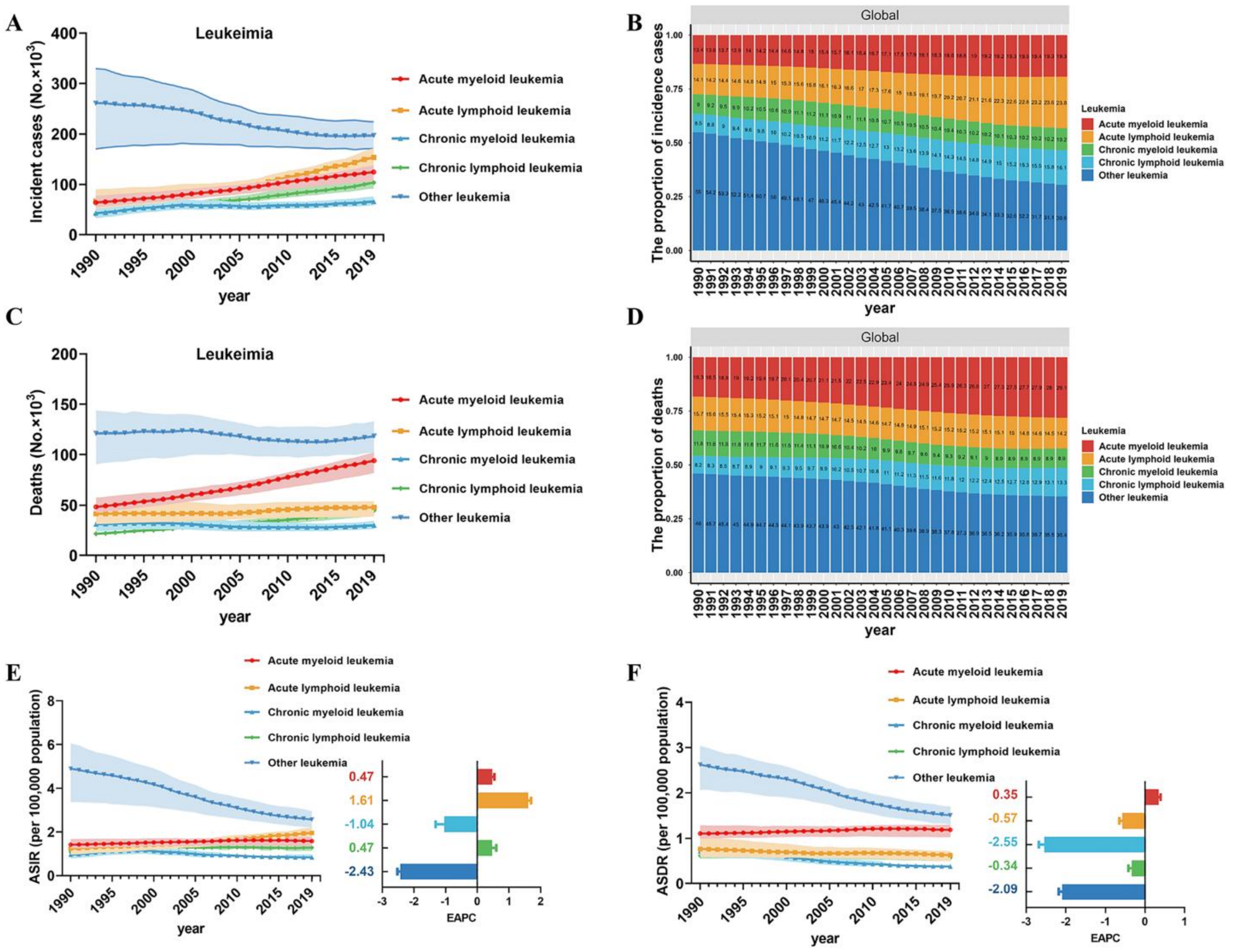

(A) The new cases of leukemia from 1990 to 2019. (B) The proportion of new cases of leukemia from 1990 to 2019. (C) The number of deaths due to leukemia from 1990 to 2019. (D) The proportion of deaths of leukemia from 1990 to 2019. (E) ASIR and EAPCs in leukemia over the last 30 years. (F) ASDR and EAPCs in leukemia over the last 30 years. ASIR, age-standardized incidence rate; ASDR, age-standardized death rate; EAPC, estimated annual percentage change.

Supplementary Figure S2. Global incidence and death of hematological malignancies by sex from 1990 to 2019.

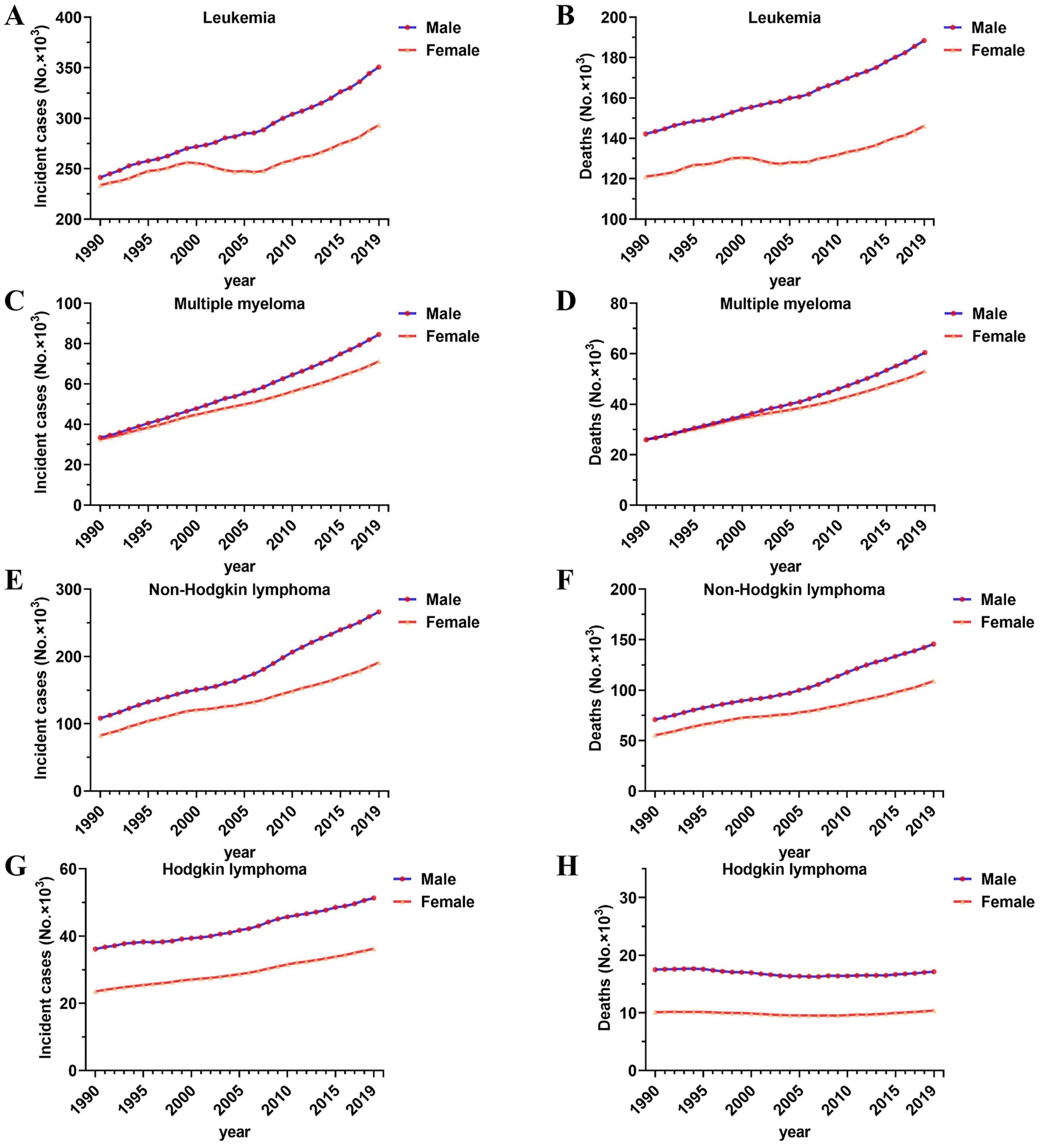

(A-B) Sex differences in leukemia. (C-D) Sex differences in multiple myeloma. (E-F) Sex differences in non-Hodgkin lymphoma. (G-H) Sex differences in Hodgkin lymphoma.

Supplementary Figure S3. Global incidence and death of leukemia by age and sex.

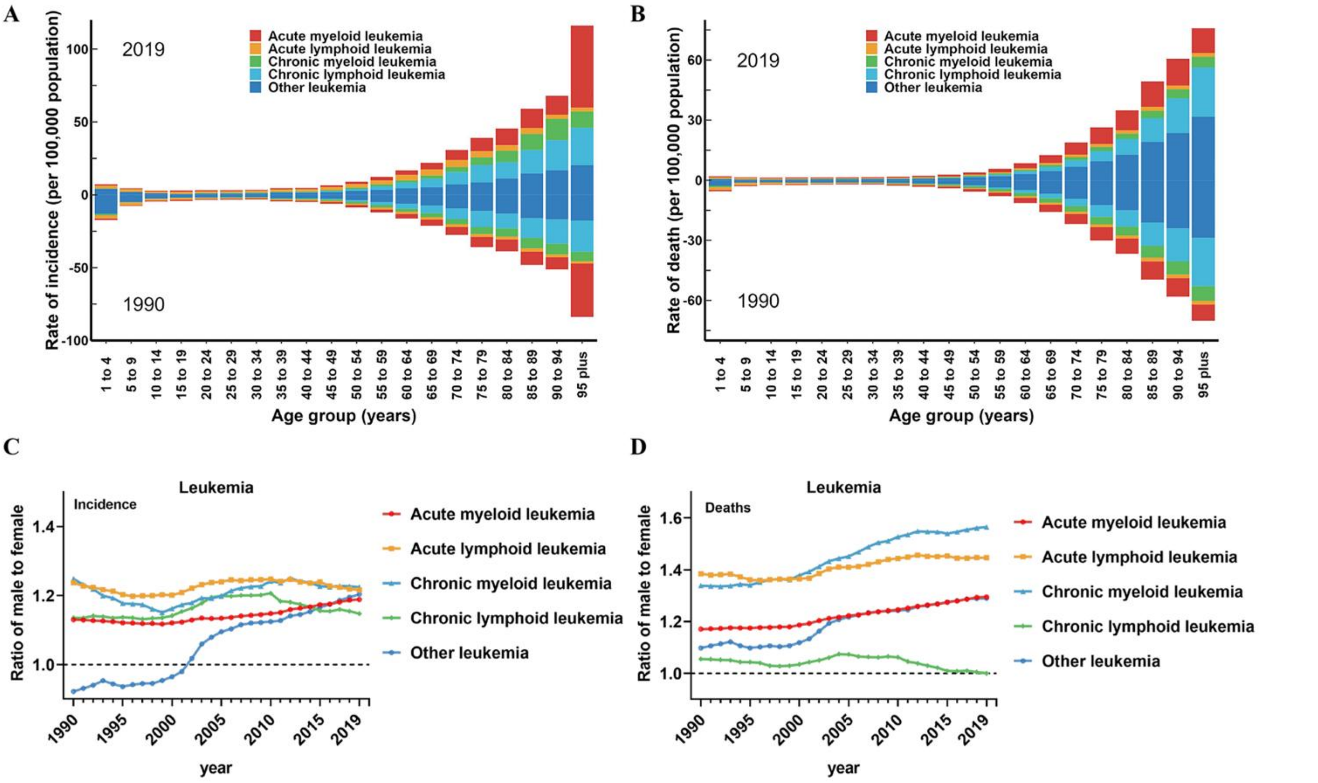

(A) Global leukemia incident cases by age for both sexes combined in 1990 and 2019. (B) Global leukemia deaths by age for both sexes combined in 1990 and 2019. For each group, the below column shows case data in 1990 and the above column shows data in 2019. (C) The sex ratio of leukemia incident cases from 1990 to 2019 by five causes. (D) The sex ratio of leukemia deaths from 1990 to 2019 by five causes.

Supplementary Figure S4. Sex differences in leukemia by age and five causes.

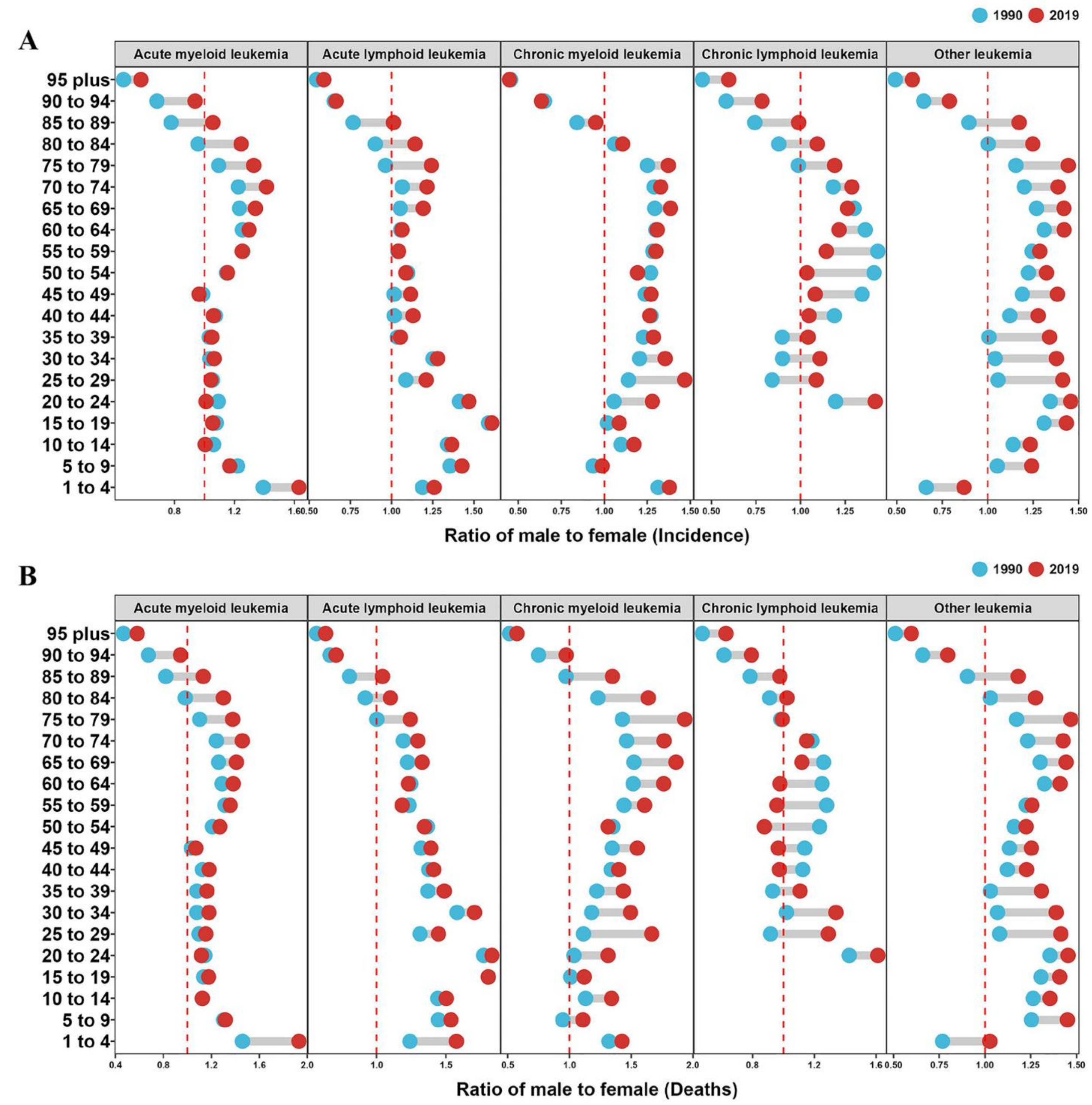

(A) The ratio of male to female in global leukemia incident cases by age in 1990 and 2019.

(B) The ratio of male to female in global leukemia deaths by age in 1990 and 2019.

Supplementary Figure S5. The ASIR of leukemia in SDI regions from 1990 to 2019.

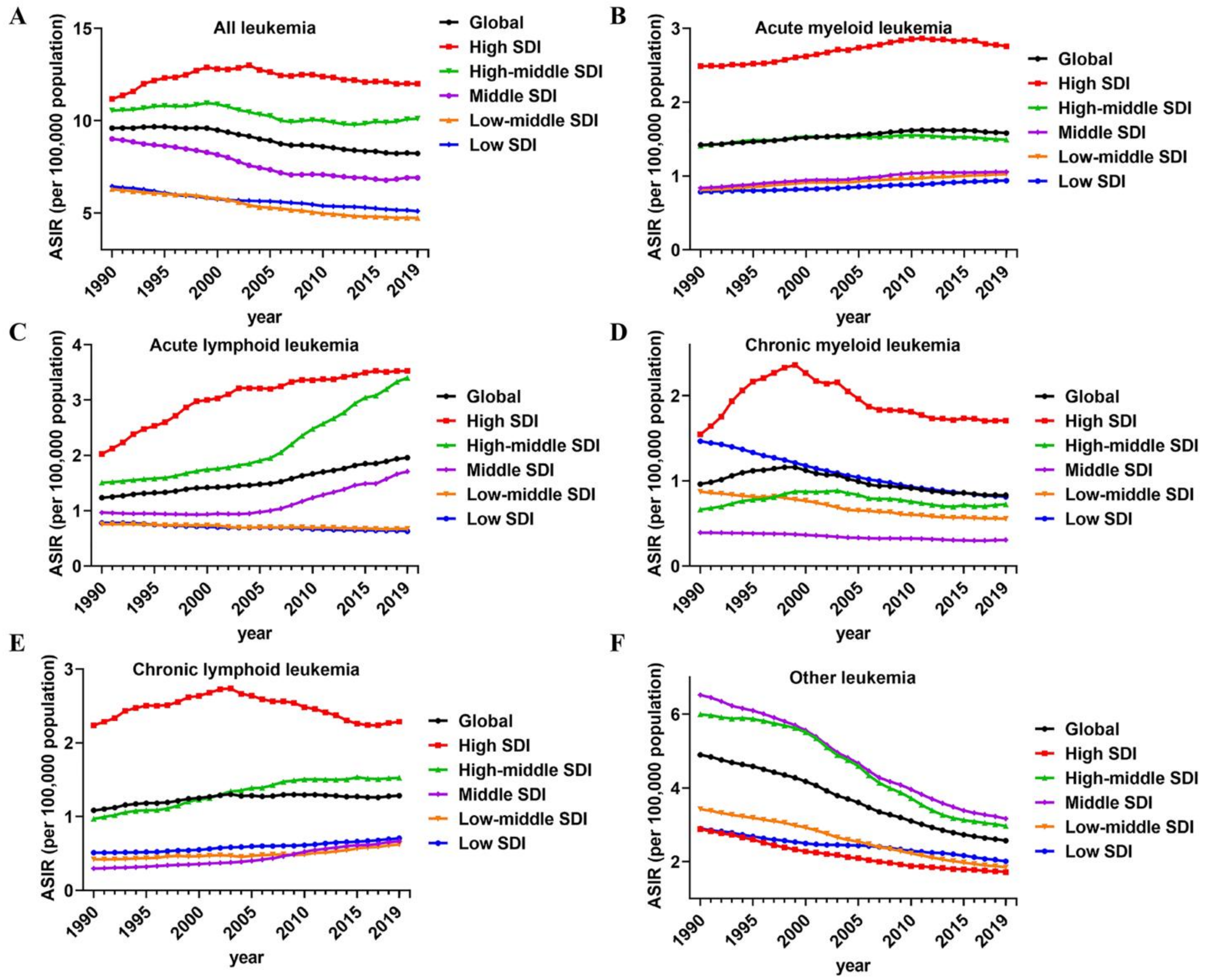

(A) All leukemia. (B) Acute myeloid leukemia. (C) Acute lymphoid leukemia. (D) Chronic myeloid leukemia. (E) Chronic lymphoid leukemia. (F) Other leukemia. ASIR, age-standardized incidence rate; SDI, Socio-demographic Index.

Supplementary Figure S6. The ASDR of leukemia in SDI regions from 1990 to 2019.

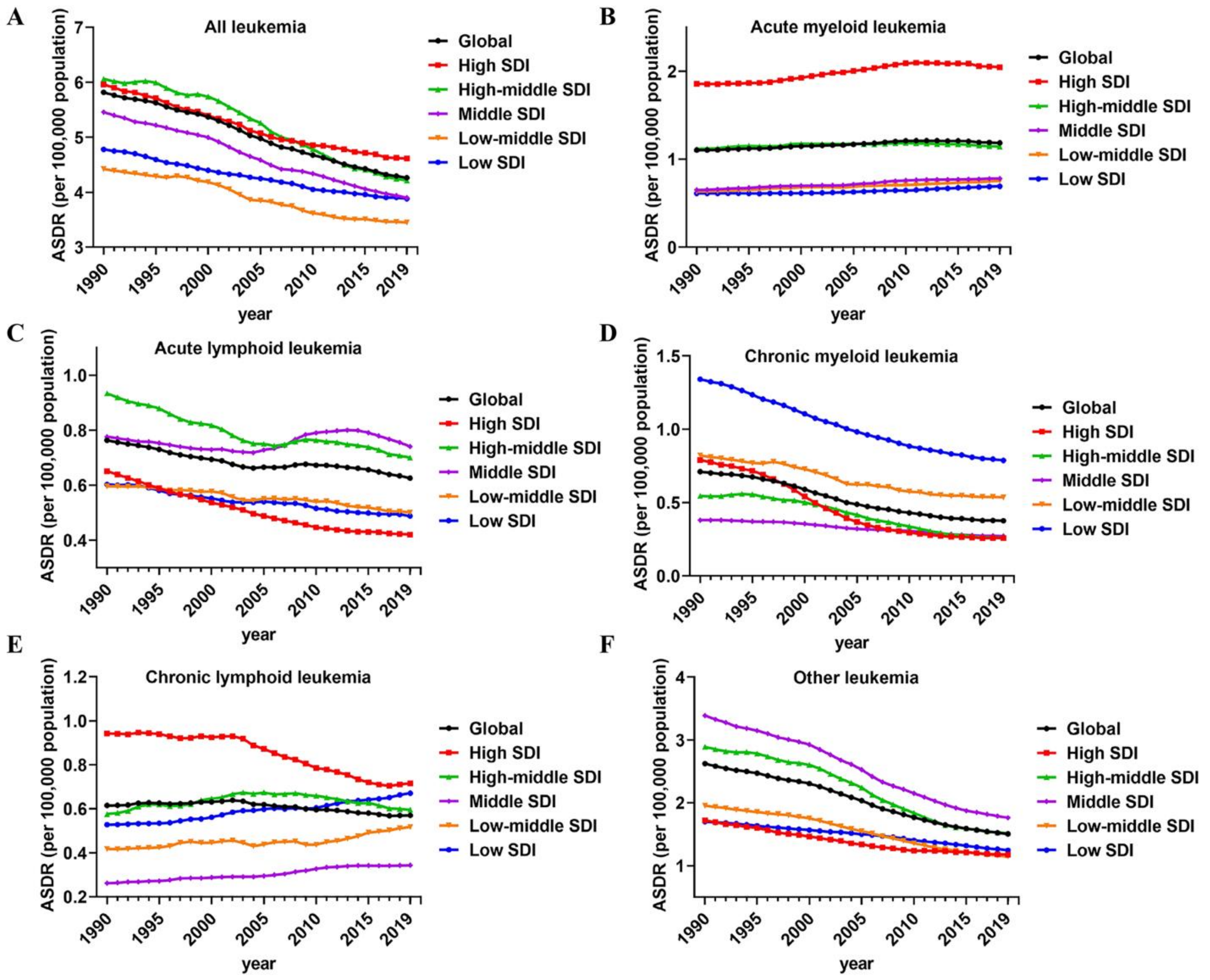

(A) All leukemia. (B) Acute myeloid leukemia. (C) Acute lymphoid leukemia. (D) Chronic myeloid leukemia. (E) Chronic lymphoid leukemia. (F) Other leukemia. ASDR, age-standardized death rate; SDI, Socio-demographic Index.

Supplementary Figure S7. Changes in the number of leukemia by countries and territories.

A

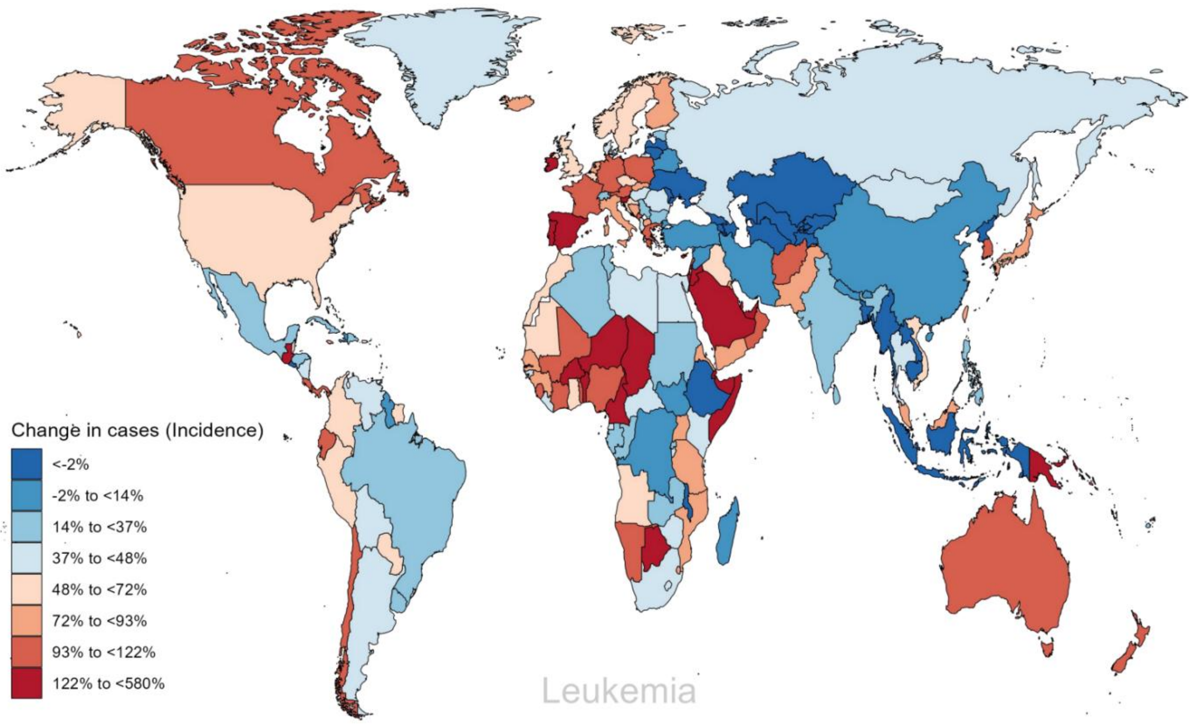

B

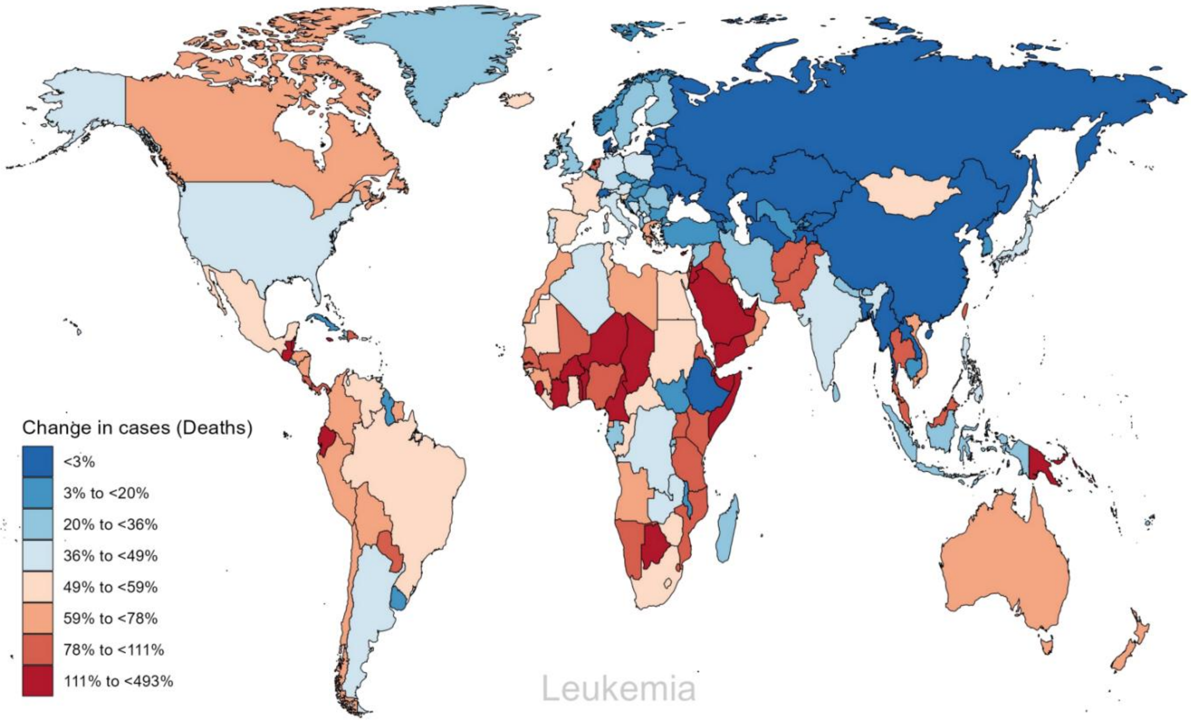

(A) The percentage change in incident cases of leukemia between 1990 and 2019. (B) The percentage change in deaths of leukemia between 1990 and 2019.

Supplementary Figure S8. The correlation between EAPC and age-standardized rate in leukemia.

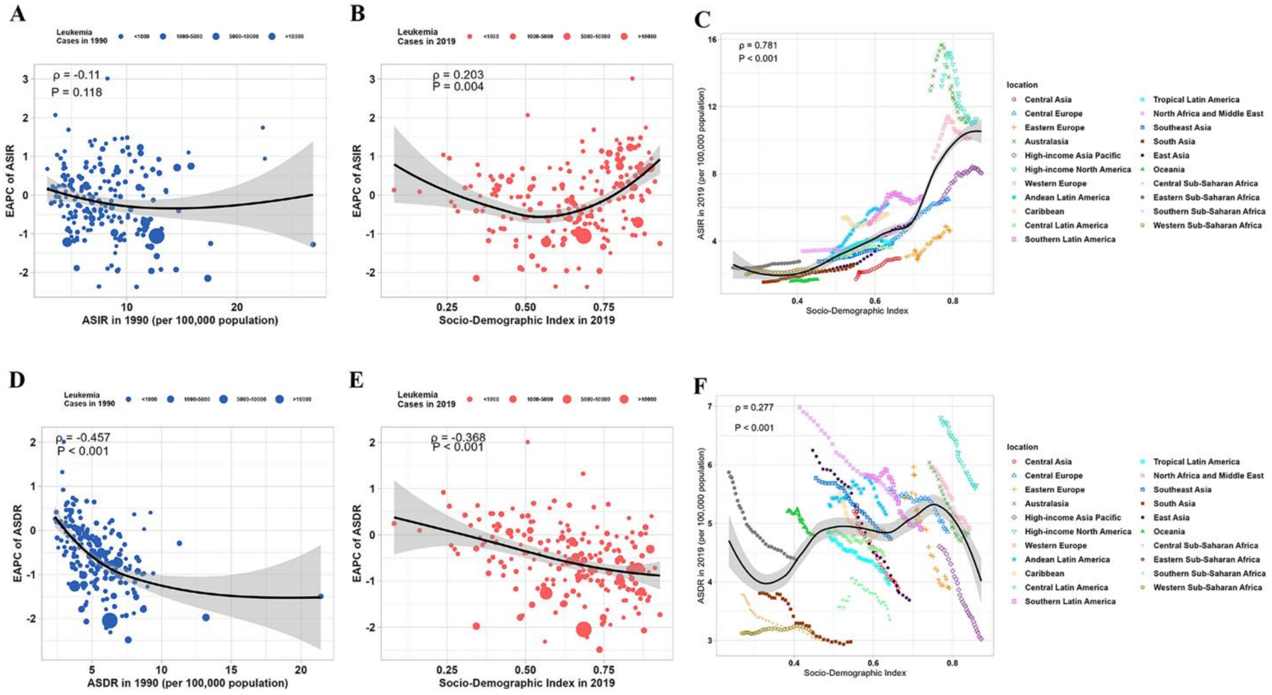

(A) The correlation between EAPC and ASIR in 1990. (B) The correlation between EAPC of ASIR and SDI in 2019. (C) ASIR of leukemia by region from 1990 to 2019 based on SDI levels. (D) The correlation between EAPC and ASDR in 1990. (R) The correlation between EAPC of ASDR and SDI in 2019. (F) ASDR of leukemia by region from 1990 to 2019 based on SDI levels. ASIR, age-standardized incidence rate; ASDR, age-standardized death rate; EAPC, estimated annual percentage change. SDI, Socio-demographic Index.

Supplementary Figure S9. The ASIR and ASDR of multiple myeloma in SDI regions from 1990 to 2019.

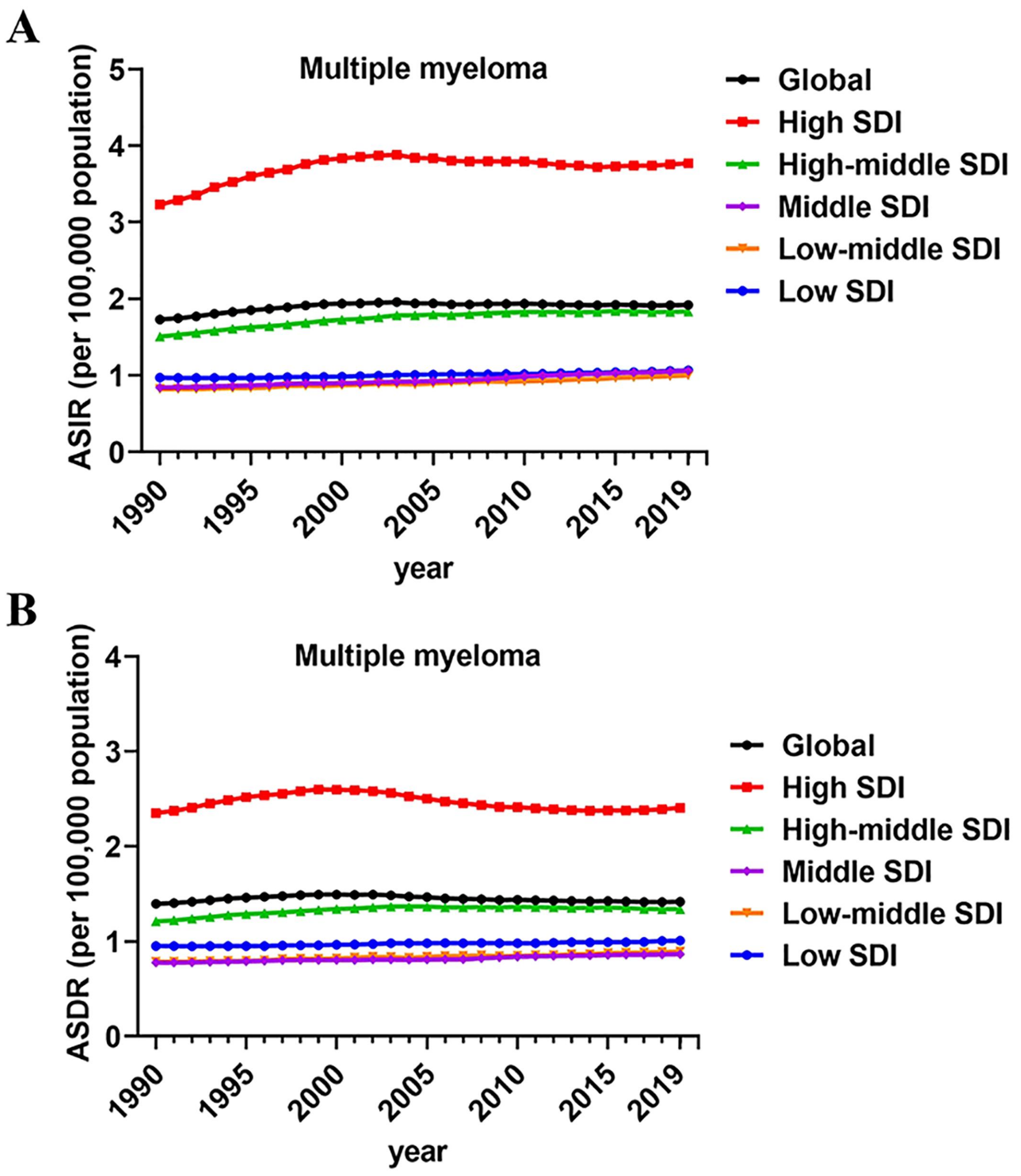

(A) The ASIR of multiple myeloma in SDI regions. (B)The ASDR of multiple myeloma in SDI regions. ASDR, age-standardized death rate; SDI, Socio-demographic Index.

Supplementary Figure S10. Changes in the number of multiple myeloma by countries and territories.

A

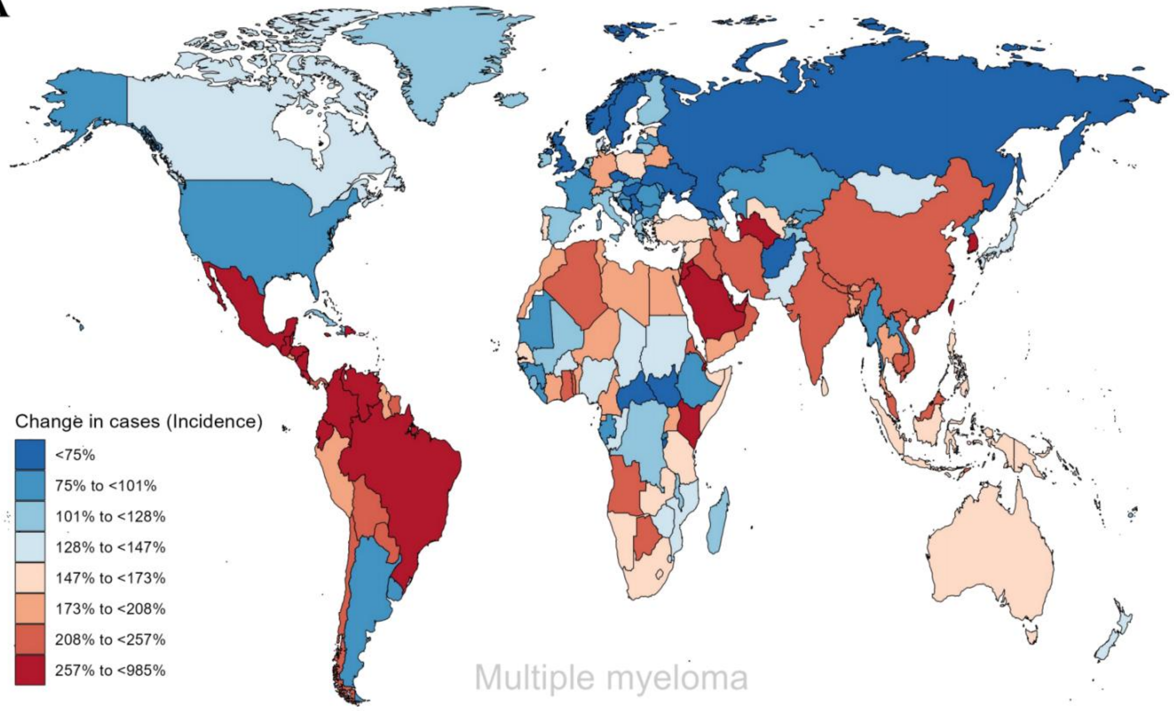

B

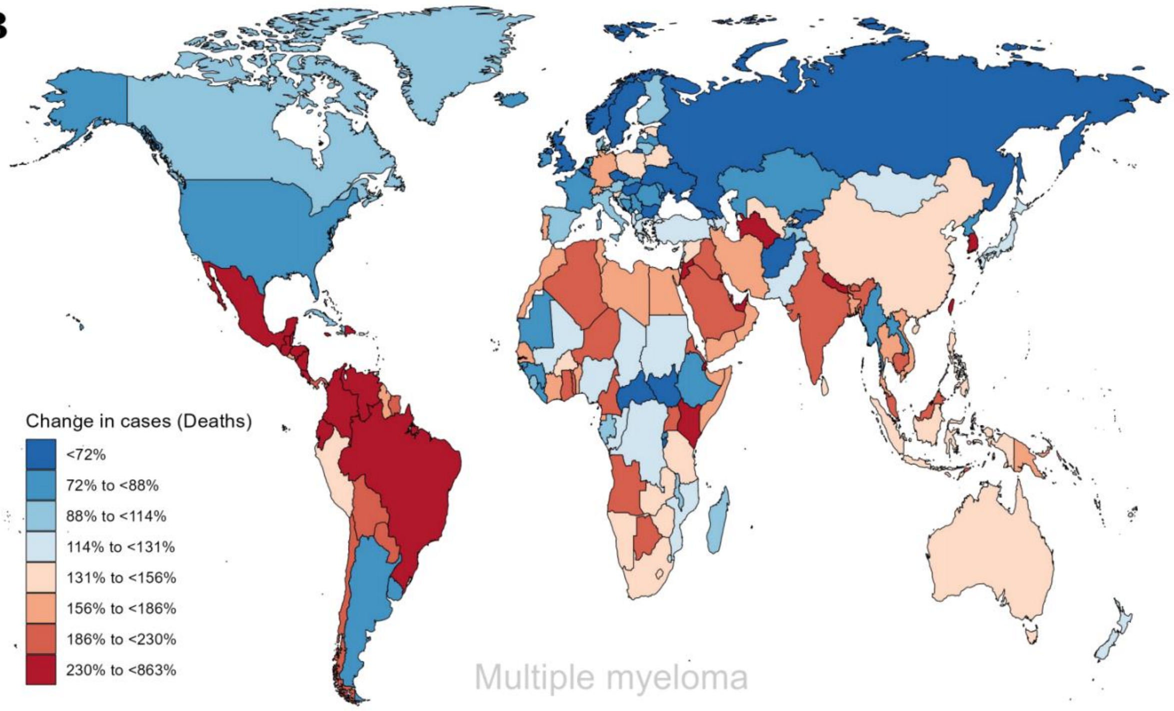

(A) The percentage change in incident cases of multiple myeloma between 1990 and 2019.

(B) The percentage change in deaths of multiple myeloma between 1990 and 2019.

Supplementary Figure S11. The correlation between EAPC and age-standardized rate in multiple myeloma.

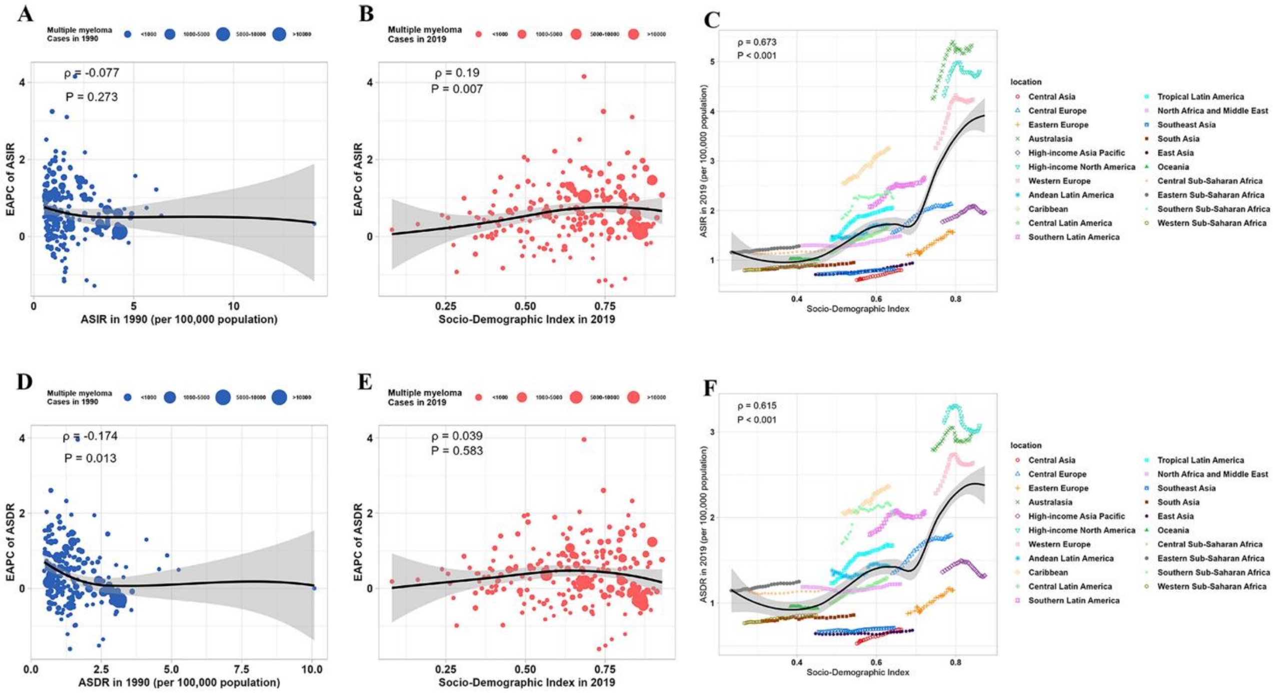

(A) The correlation between EAPC and ASIR in 1990. (B) The correlation between EAPC of ASIR and SDI in 2019. (C) ASIR of multiple myeloma by region from 1990 to 2019 based on SDI levels. (D) The correlation between EAPC and ASDR in 1990. (R) The correlation between EAPC of ASDR and SDI in 2019. (F) ASDR of multiple myeloma by region from 1990 to 2019 based on SDI levels. ASIR, age-standardized incidence rate; ASDR, age-standardized death rate; EAPC, estimated annual percentage change. SDI, Socio-demographic Index.

Supplementary Figure S12. The ASIR and ASDR of lymphoma in SDI regions from 1990 to 2019.

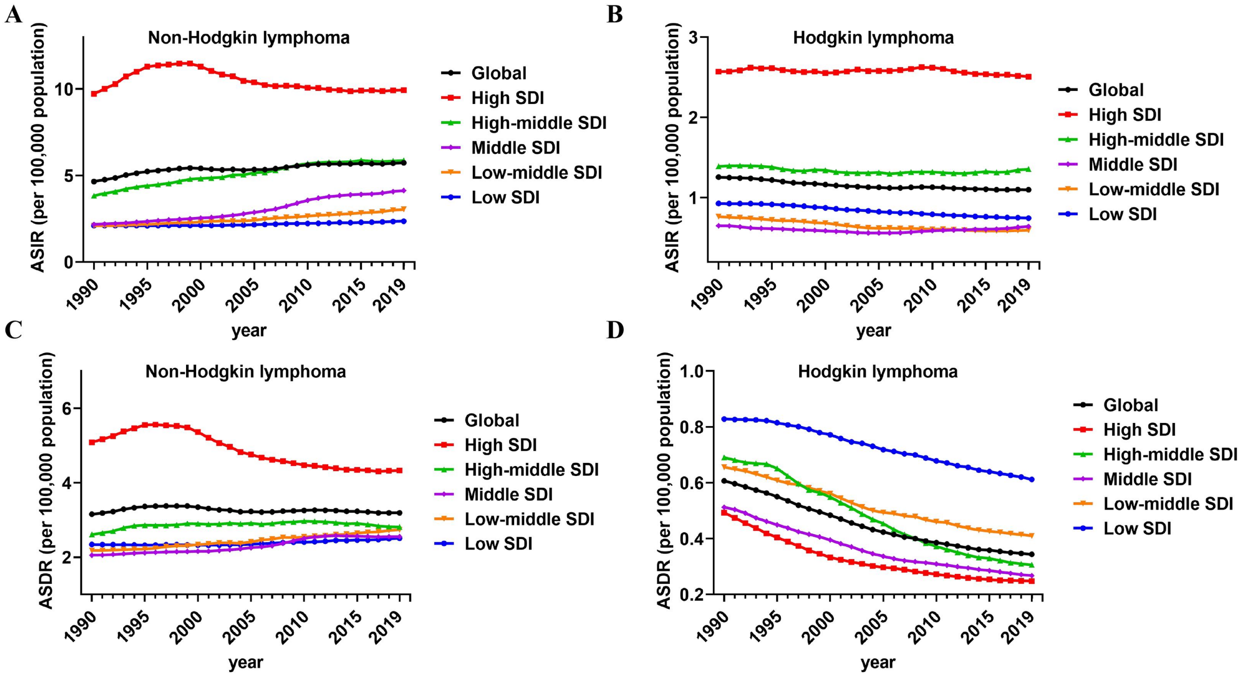

(A) The ASIR of non-Hodgkin lymphoma in SDI regions. (B) The ASIR of Hodgkin lymphoma in SDI regions. (C) The ASDR of non-Hodgkin lymphoma in SDI regions. (D) The ASDR of Hodgkin lymphoma in SDI regions. ASDR, age-standardized death rate; SDI, Socio-demographic Index.

Supplementary Figure S13. Changes in the number of non-Hodgkin lymphoma by countries and territories.

A

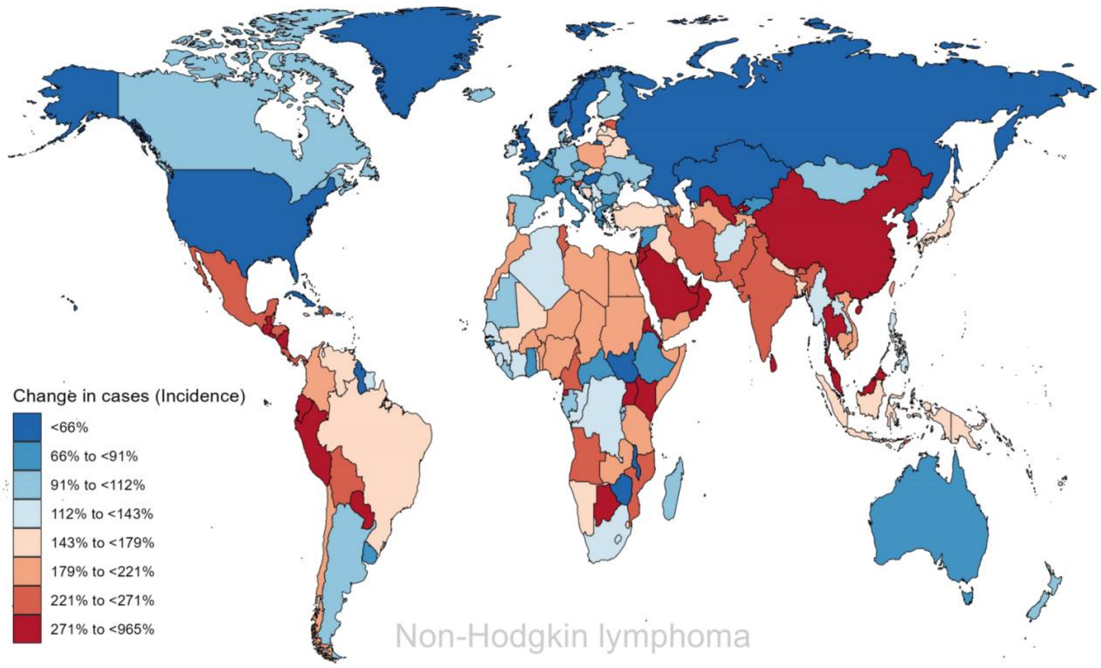

B

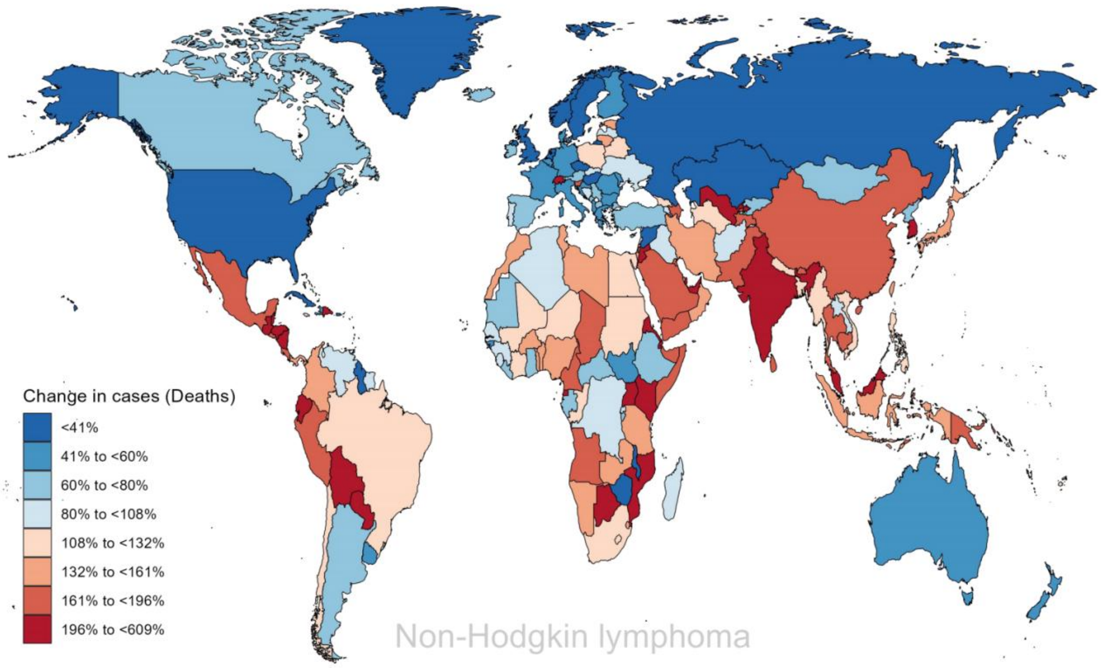

(A) The percentage change in incident cases of non-Hodgkin lymphoma between 1990 and 2019. (B) The percentage change in deaths of non-Hodgkin lymphoma between 1990 and 2019.

Supplementary Figure S14. The global trends of Hodgkin lymphoma by countries and territories.

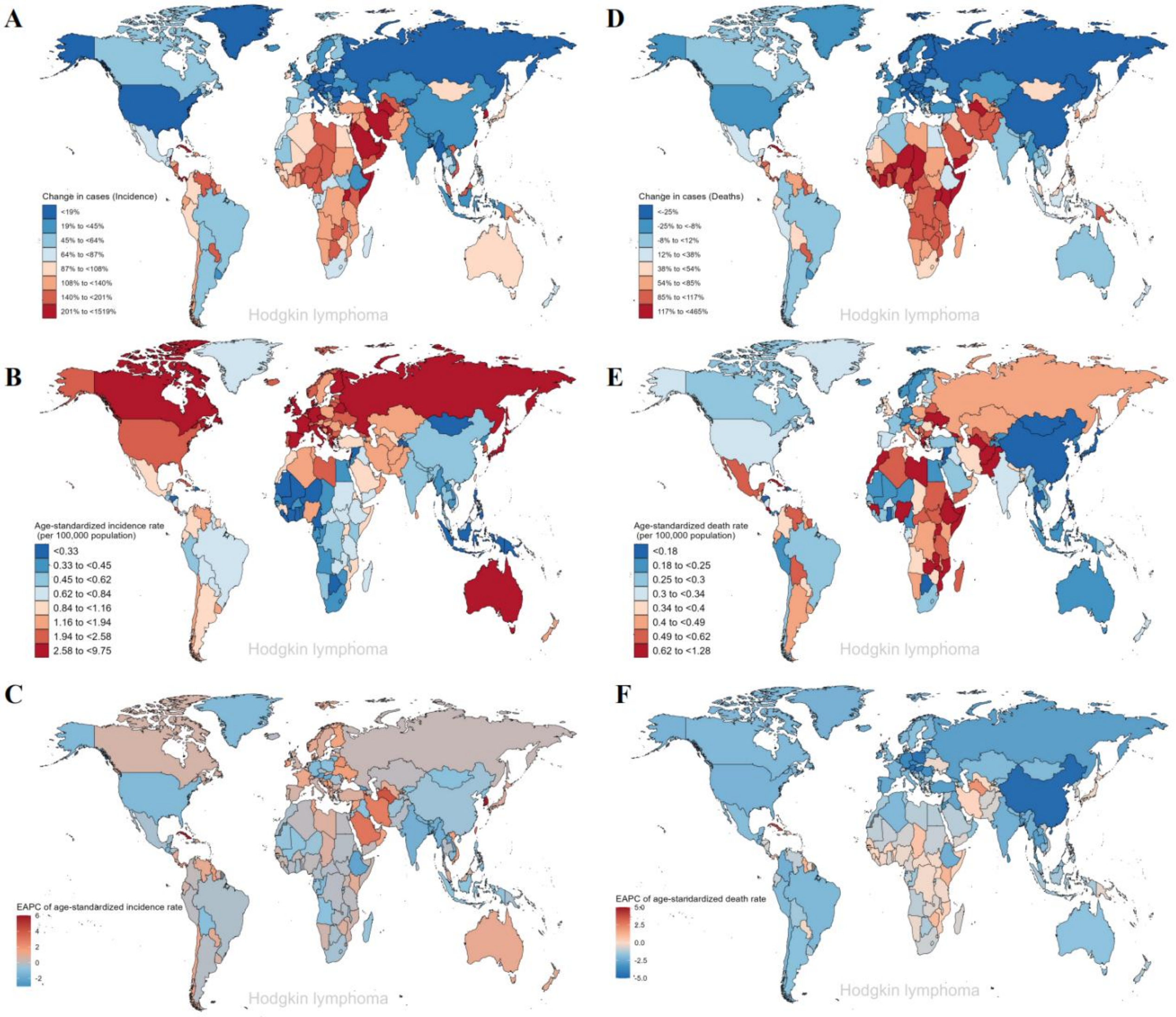

(A) The percentage change in incident cases of Hodgkin lymphoma between 1990 and 2019. (B) The ASIR of Hodgkin lymphoma in 2019. (C) The EAPC in ASIR of Hodgkin lymphoma from 1990 to 2019. (D) The percentage change in deaths of Hodgkin lymphoma between 1990 and 2019. (E) The ASDR of Hodgkin lymphoma in 2019. (F) The EAPC in ASDR of Hodgkin lymphoma from 1990 to 2019. ASIR, age-standardized incidence rate; ASDR, age-standardized death rate; EAPC, estimated annual percentage change.

Supplementary Figure S15. The correlation between EAPC and age-standardized rate in non-Hodgkin lymphoma.

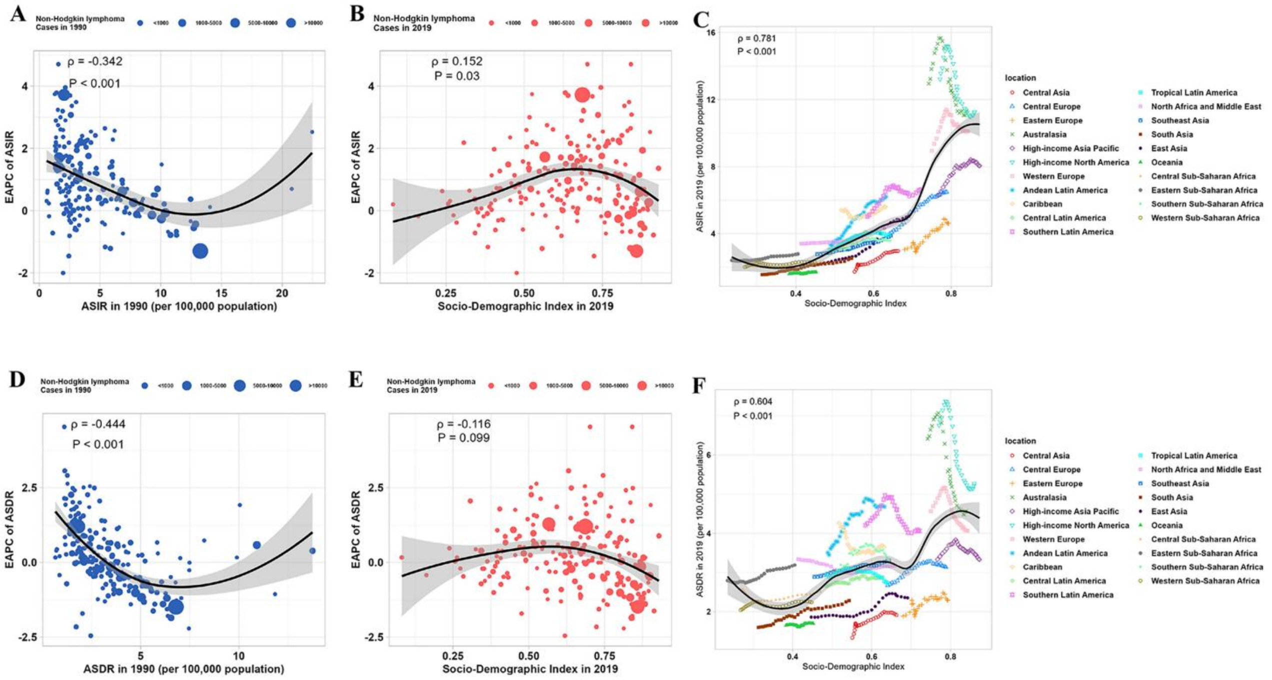

(A) The correlation between EAPC and ASIR in 1990. (B) The correlation between EAPC of ASIR and SDI in 2019. (C) ASIR of non-Hodgkin lymphoma by region from 1990 to 2019 based on SDI levels. (D) The correlation between EAPC and ASDR in 1990. (R) The correlation between EAPC of ASDR and SDI in 2019. (F) ASDR of non-Hodgkin lymphoma by region from 1990 to 2019 based on SDI levels. ASIR, age-standardized incidence rate; ASDR, age-standardized death rate; EAPC, estimated annual percentage change. SDI, Socio-demographic Index.

Supplementary Figure S16. The correlation between EAPC and age-standardized rate in Hodgkin lymphoma.

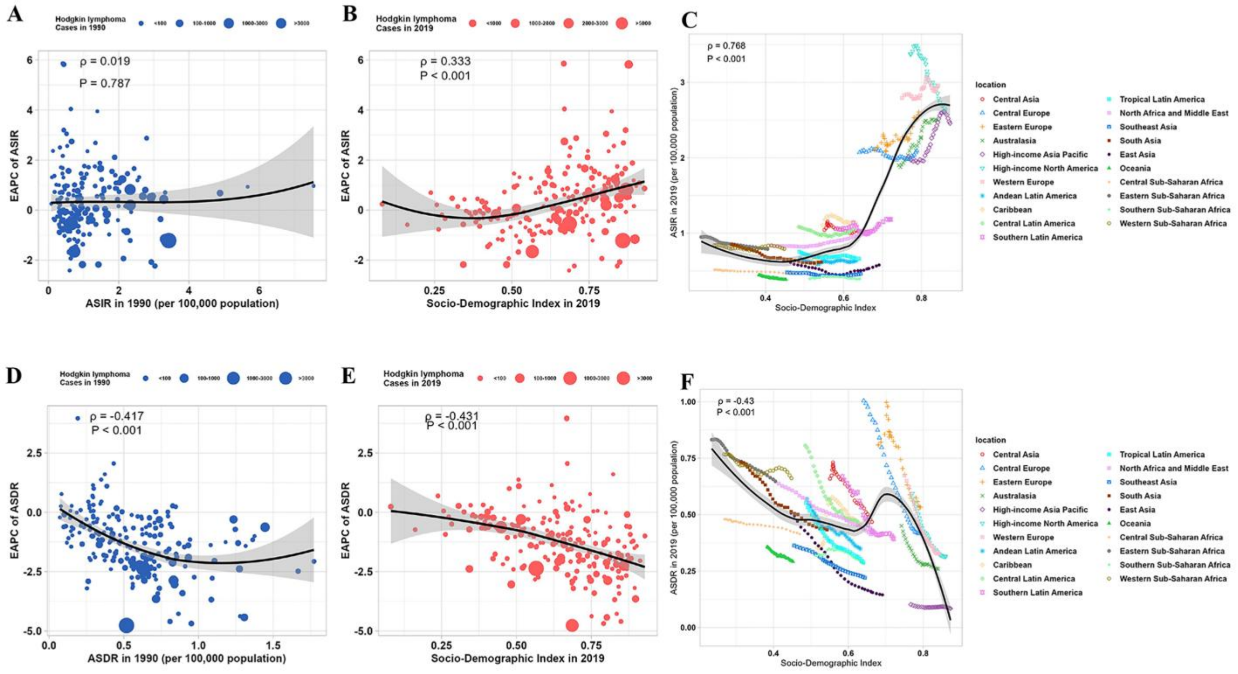

(A) The correlation between EAPC and ASIR in 1990. (B) The correlation between EAPC of ASIR and SDI in 2019. (C) ASIR of Hodgkin lymphoma by region from 1990 to 2019 based on SDI levels. (D) The correlation between EAPC and ASDR in 1990. (R) The correlation between EAPC of ASDR and SDI in 2019. (F) ASDR of Hodgkin lymphoma by region from 1990 to 2019 based on SDI levels. ASIR, age-standardized incidence rate; ASDR, age-standardized death rate; EAPC, estimated annual percentage change. SDI, Socio-demographic Index.

Supplementary Figure S17. Contribution of death causes to hematological malignancies by regions in 1990 and 2019.

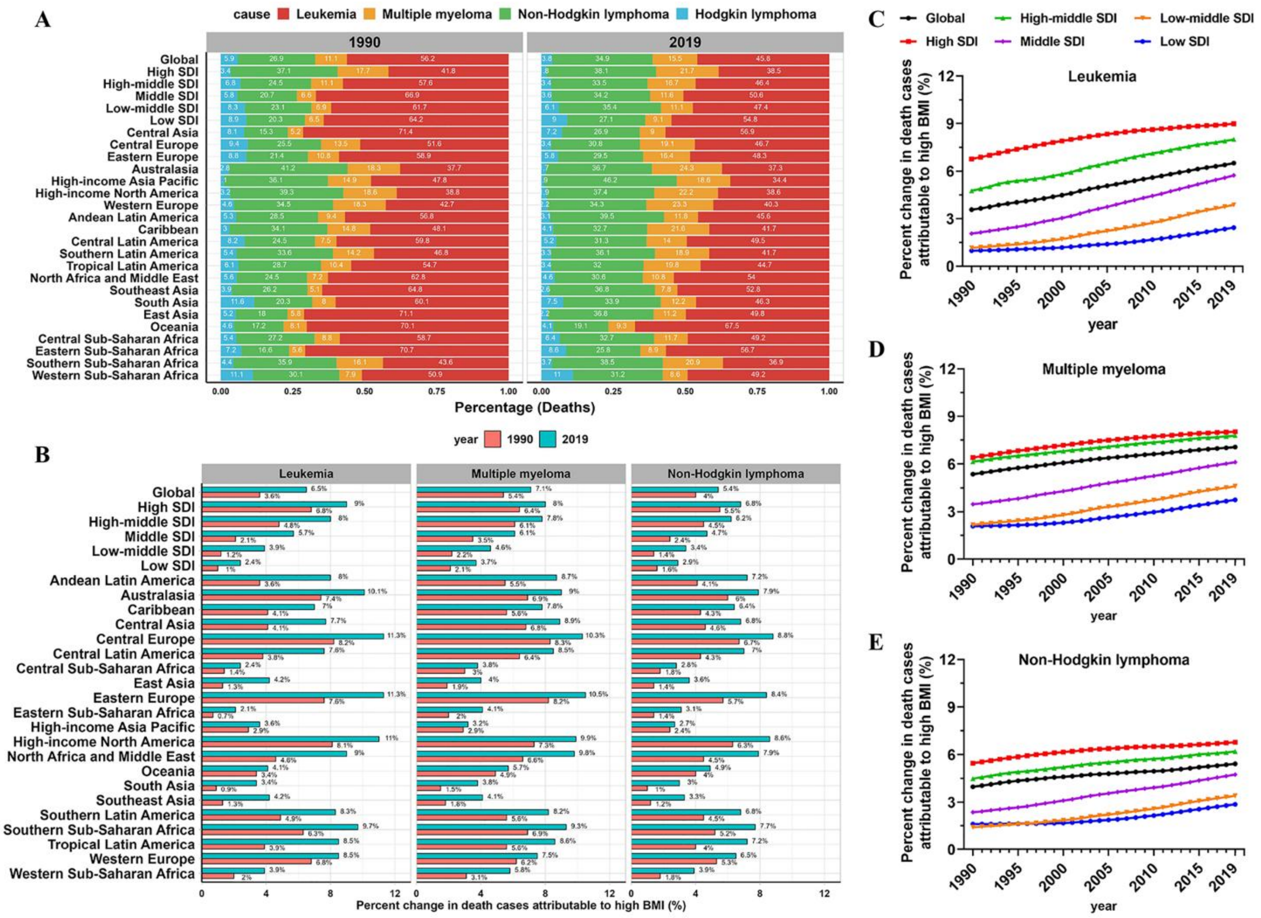

(A) Changes in the proportion of death cases from hematologic malignancies in all regions between 1990 and 2019. (B) The percent change in death cases attributable to high body-mass index in all regions between 1990 and 2019. (C) The percent change in leukemia death cases attributable to high body-mass index by SDI region from 1990 to 2019. (D) The percent change in multiple myeloma death cases attributable to high body-mass index by SDI region from 1990 to 2019. (E) The percent change in non-Hodgkin lymphoma death cases attributable to high body-mass index by SDI region from 1990 to 2019. SDI, Socio-demographic Index; BMI, Body-mass Index.

Supplementary Figure S18. Predominant contribution of occupational carcinogens to leukemia deaths by regions.

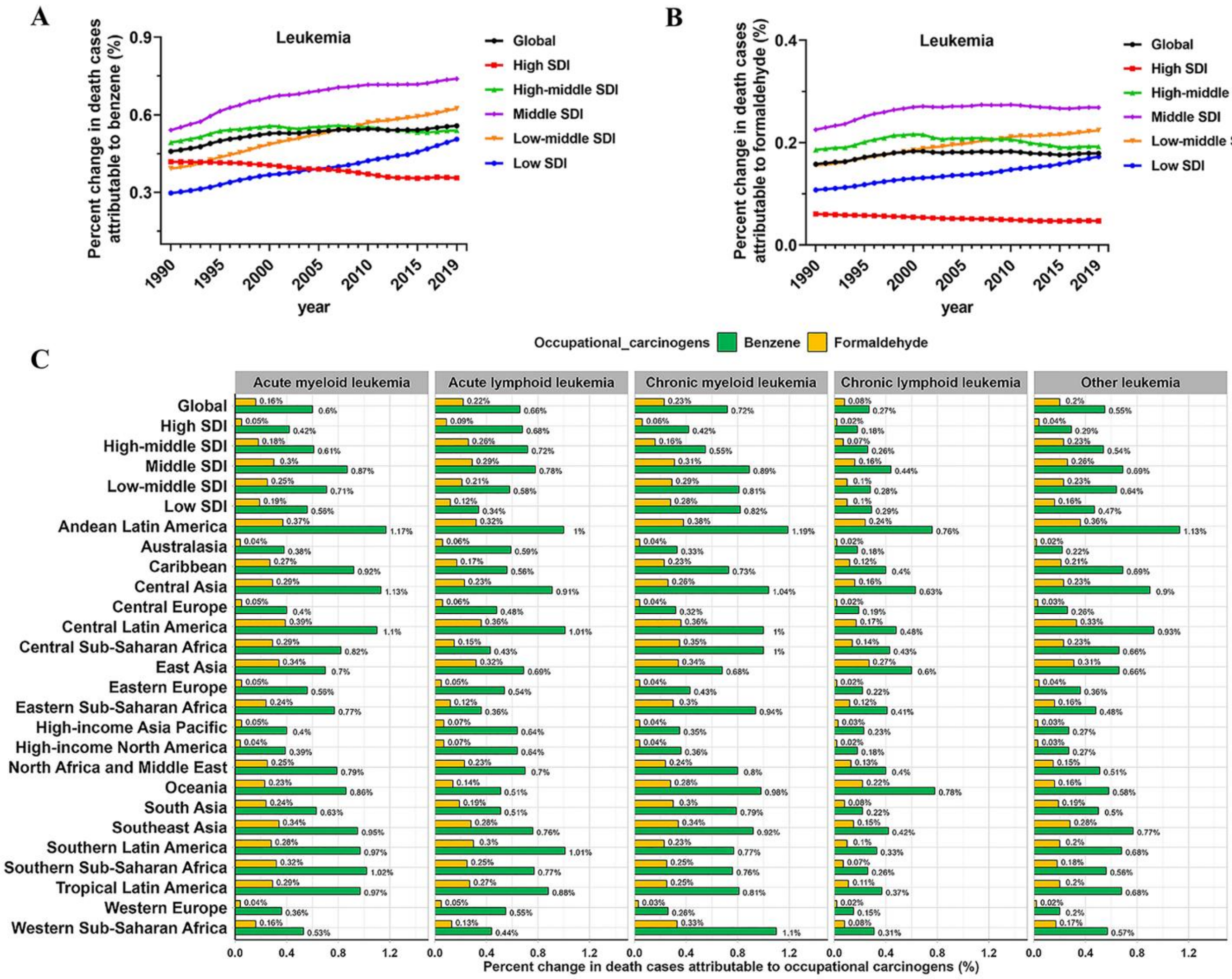

(A) The percent change in leukemia death cases attributable to occupational exposure to benzene by SDI region from 1990 to 2019. (B) The percent change in leukemia death cases attributable to occupational exposure to formaldehyde by SDI region from 1990 to 2019. (C) The percent change in death cases attributable to occupational carcinogens by all regions in 2019. SDI, Socio-demographic Index.

Supplementary Figure S19. Predominant contribution of high body-mass index to hematological malignancies deaths by region, sex, and age groups.

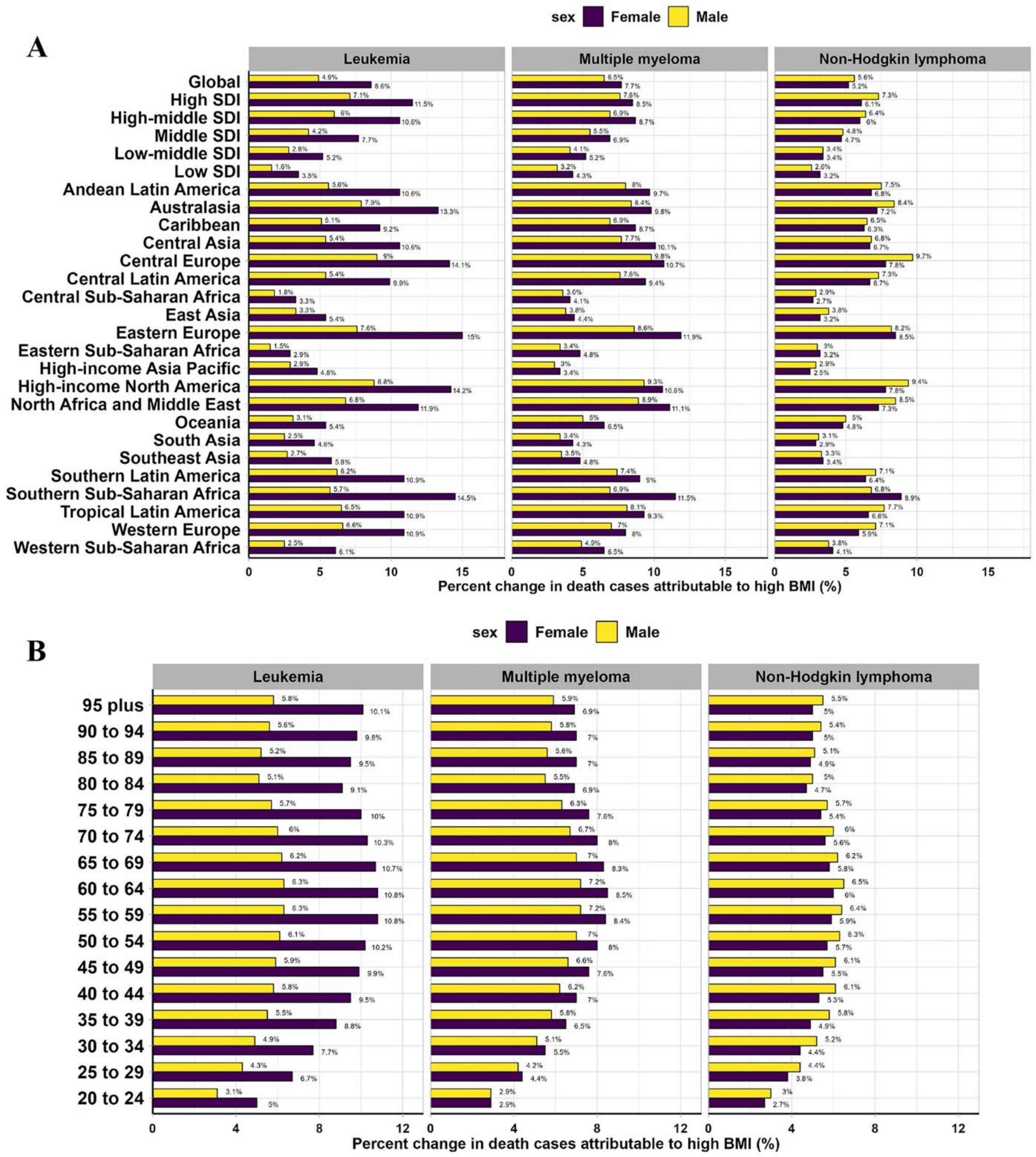

(A) The percent change in death cases attributable to high body-mass index by region and sex in 2019. (B) The percent change in death cases attributable to high body-mass index by age and sex, globally, 2019. SDI, Socio-demographic Index; BMI, Body-mass Index.

Supplementary Figure S20. Predominant contribution of occupational carcinogens in hematological malignancies deaths by region, sex, and age groups.

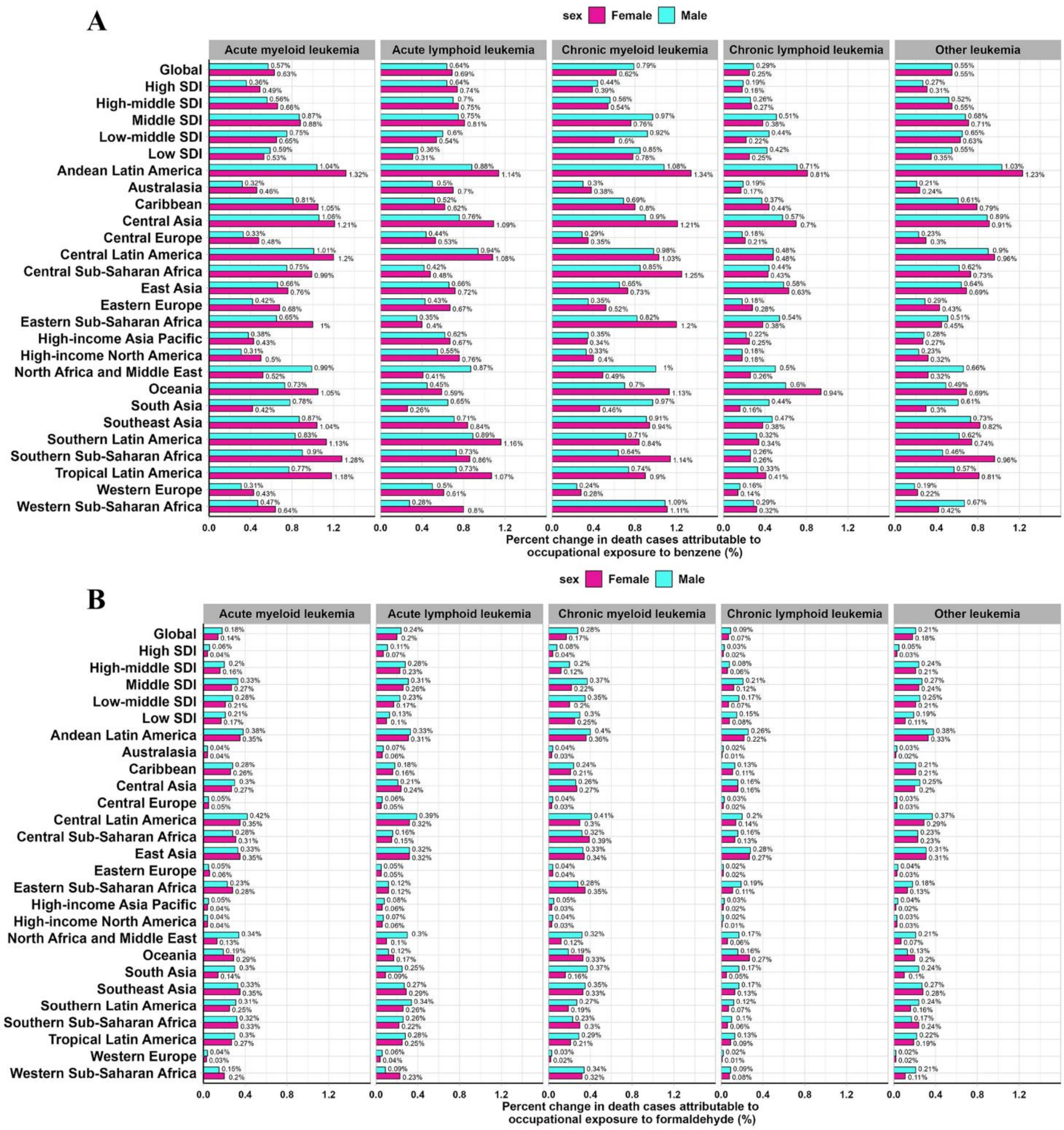

(A) The percent change in death cases attributable to occupational exposure to benzene by region and sex in 2019. (B) The percent change in death cases attributable to occupational exposure to formaldehyde by region and sex in 2019.

Supplementary Figure S21. Analysis of population, number of cases and incidence rates in two countries (Qatar and the United Arab Emirates) with large fluctuations in cases from 1990 to 2019.

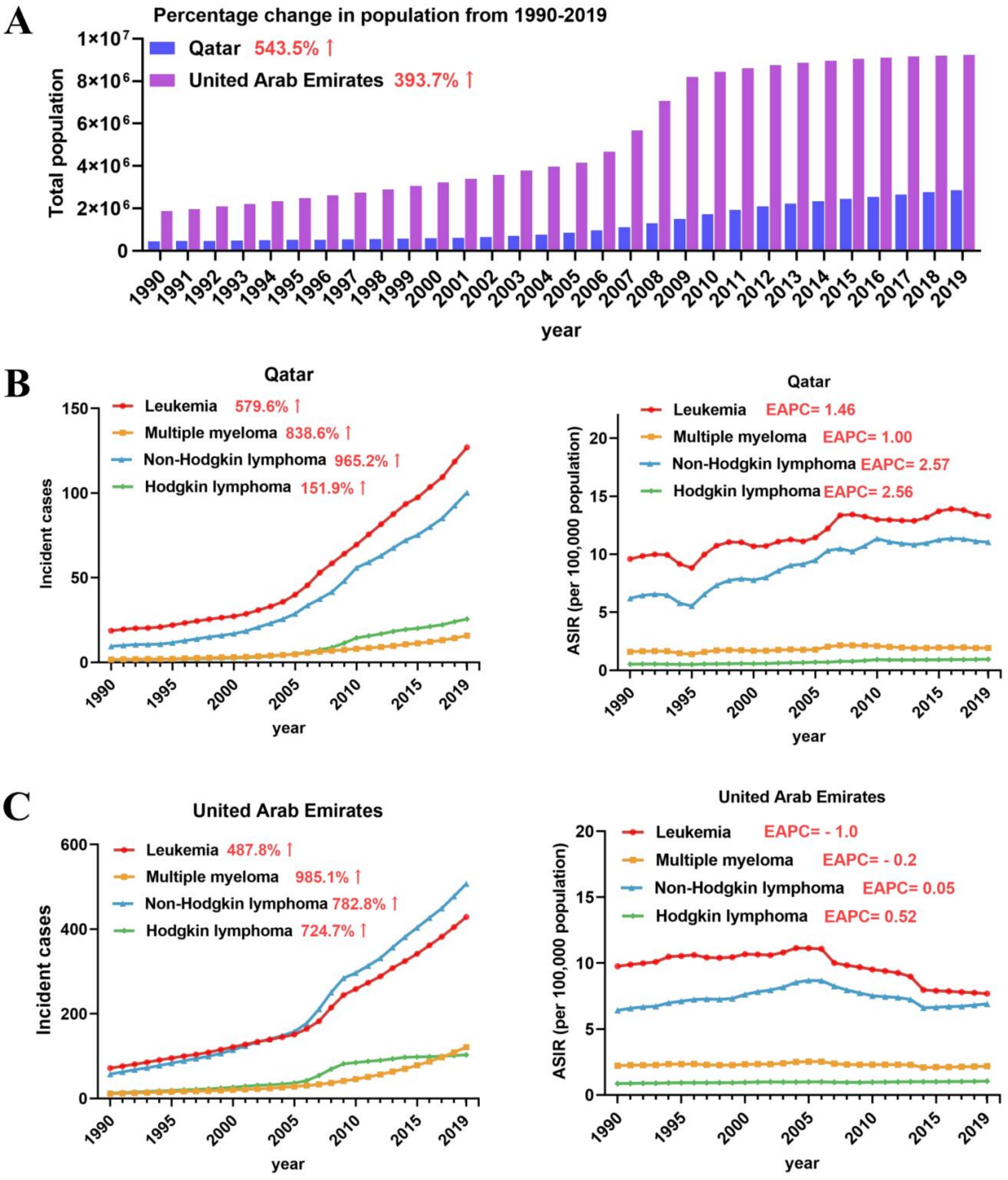

(A) Demographic changes in Qatar and the United Arab Emirates. (B) Number of cases and incidence rates of hematological malignancies in Qatar. (C) Number of cases and incidence rates of hematological malignancies in United Arab Emirates.

Supplementary Figure S22. Crude incidence rates for the 10 countries with the largest age-standardized incidence rates.

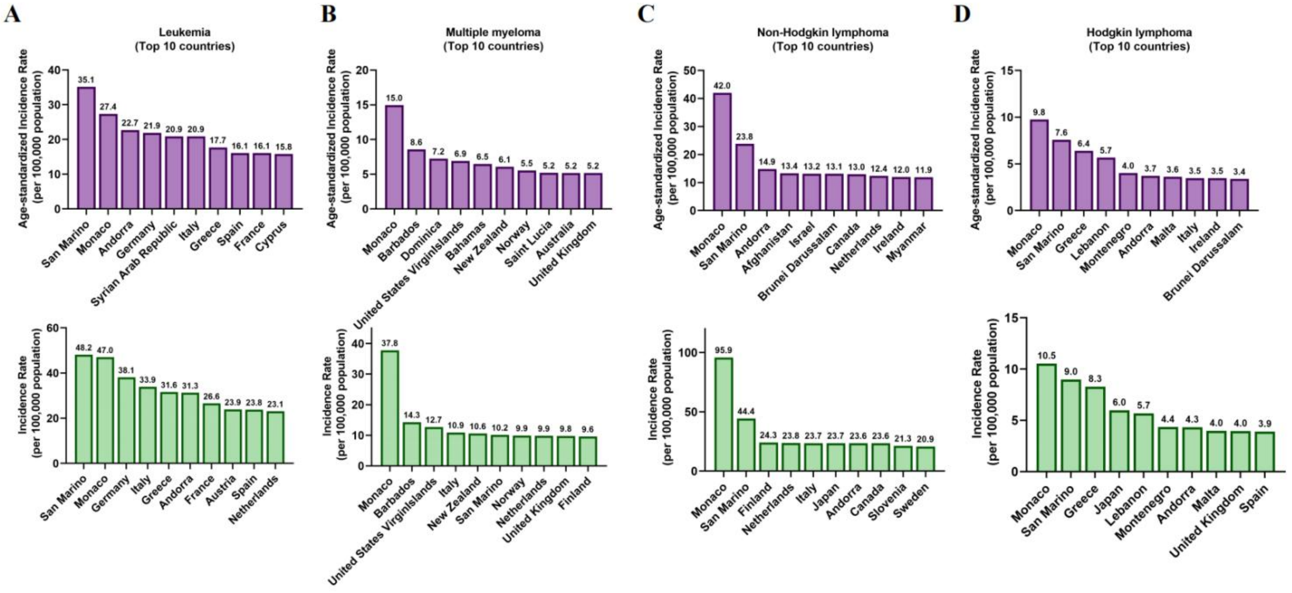

(A) Leukemia. (B) Multiple myeloma. (C) Non-Hodgkin lymphoma. (D) Hodgkin lymphoma.

Supplementary Table S1. 21 geographic regions in 204 countries around the world.

Regions	Countries	Regions	Countries	Regions	Countries
**Andean Latin America**	Bolivia	**Eastern Sub-Saharan Africa**	Burundi	**Southeast Asia**	Cambodia
	Ecuador		Comoros		Indonesia
	Peru		Djibouti		Lao People's
**Australasia**	Australia		Eritrea		Malaysia
	New Zealand		Ethiopia		Maldives
**Caribbean**	Antigua and		Kenya		Mauritius
	Bahamas		Madagascar		Myanmar
	Barbados		Malawi		Philippines
	Belize		Mozambique		Seychelles
	Bermuda		Rwanda		Sri Lanka
	Cuba		Somalia		Thailand
	Dominica		South Sudan		Timor-Leste
	Dominican		Uganda		Viet Nam
	Grenada		United Republic	**Southern Latin America**	Argentina
	Guyana		Zambia		Chile
	Haiti	**High-income Asia Pacific**	Brunei		Uruguay
	Jamaica		Japan	**Southern Sub-Saharan Africa**	Botswana
	Puerto Rico		Republic of		Eswatini
	Saint Kitts and		Singapore		Lesotho
	Saint Lucia	**High-income North America**	Canada		Namibia
	Saint Vincent		Greenland		South Africa
	Suriname		United States of		Zimbabwe
	Trinidad and	**North Africa and Middle East**	Afghanistan	**Tropical Latin America**	Brazil
	United States		Algeria		Paraguay
**Central Asia**	Armenia		Bahrain	**Western Europe**	Andorra
	Azerbaijan		Egypt		Austria
	Georgia		Iran (Islamic		Belgium
	Kazakhstan		Iraq		Cyprus
	Kyrgyzstan		Jordan		Denmark
	Mongolia		Kuwait		Finland
	Tajikistan		Lebanon		France
	Turkmenistan		Libya		Germany
	Uzbekistan		Morocco		Greece
**Central Europe**	Albania		Oman		Iceland
	Bosnia and		Palestine		Ireland
	Bulgaria		Qatar		Israel
	Croatia		Saudi Arabia		Italy
	Czechia		Sudan		Luxembourg
	Hungary		Syrian Arab		Malta
	Montenegro		Tunisia		Monaco
	North Macedonia		Turkey		Netherlands
	Poland		United Arab		Norway
	Romania		Yemen		Portugal
	Serbia	**Oceania**	American Samoa		San Marino
	Slovakia		Cook Islands		Spain
	Slovenia		Fiji		Sweden
**Central Latin**	Colombia		Guam		Switzerland

	Costa Rica		Kiribati		United Kingdom
	El Salvador		Marshall Islands	**Western Sub-Saharan Africa**	Benin
	Guatemala		Micronesia		Burkina Faso
	Honduras		Nauru		Cabo Verde
	Mexico		Niue		Cameroon
	Nicaragua		Northern		Chad
	Panama		Palau		Côte d'Ivoire
	Venezuela		Papua New		Gambia
**Central Sub-Saharan Africa**	Angola		Samoa		Ghana
	Central African		Solomon Islands		Guinea
	Congo		Tokelau		Guinea-Bissau
	Democratic		Tonga		Liberia
	Equatorial		Tuvalu		Mali
	Gabon		Vanuatu		Mauritania
**East Asia**	China	**South Asia**	Bangladesh		Niger
	Democratic		Bhutan		Nigeria
	Taiwan		India		Sao Tome and
**Eastern Europe**	Belarus		Nepal		Senegal
	Estonia		Pakistan		Sierra Leone
	Latvia				Togo
	Lithuania				
	Republic of				
	Russian				
	Ukraine				

Supplementary Table S2. Global age-standardised SEVs for both sexes combined in 1990 and 2019, and annualised rate of change between 1990 and 2019.

Regions	risk factors	SEV 1990 (95% UI)	SEV 2019 (95% UI)	ARC 1990 to 2019 (95% UI)
**Global**	High body-mass index	0.82 (0.51 to 1.45)	1.11 (0.69 to 1.97)	0.35 (0.3 to 0.41)
	Occupational exposure to benzene	0.64 (0.04 to 1.86)	0.84 (0.05 to 2.45)	0.31 (0.29 to 0.36)
	Occupational exposure to formaldehyde	0.99 (0.61 to 1.76)	1.27 (0.77 to 2.24)	0.28 (0.24 to 0.33)
**High SDI**	High body-mass index	0.92 (0.57 to 1.67)	1.05 (0.65 to 1.87)	0.13 (0.06 to 0.21)
	Occupational exposure to benzene	0.69 (0.42 to 1.22)	0.84 (0.53 to 1.52)	0.23 (0.16 to 0.3)
	Occupational exposure to formaldehyde	0.78 (0.05 to 2.25)	0.92 (0.06 to 2.62)	0.17 (0.12 to 0.24)
**High-middle SDI**	High body-mass index	0.69 (0.42 to 1.24)	0.86 (0.53 to 1.52)	0.25 (0.22 to 0.28)
	Occupational exposure to benzene	0.65 (0.4 to 1.17)	0.85 (0.52 to 1.52)	0.32 (0.29 to 0.35)
	Occupational exposure to formaldehyde	0.76 (0.47 to 1.36)	0.98 (0.61 to 1.75)	0.3 (0.27 to 0.33)
**Middle SDI**	High body-mass index	0.96 (0.59 to 1.72)	1.27 (0.79 to 2.26)	0.33 (0.22 to 0.47)
	Occupational exposure to benzene	0.82 (0.05 to 2.35)	1.08 (0.06 to 3.14)	0.32 (0.31 to 0.36)
	Occupational exposure to formaldehyde	0.85 (0.52 to 1.51)	1 (0.6 to 1.77)	0.17 (0.12 to 0.25)
**Low-middle SDI**	High body-mass index	0.8 (0.49 to 1.45)	1.13 (0.69 to 2.01)	0.41 (0.36 to 0.45)
	Occupational exposure to benzene	1.1 (0.67 to 1.97)	1.43 (0.89 to 2.54)	0.3 (0.23 to 0.39)
	Occupational exposure to formaldehyde	0.93 (0.55 to 1.65)	0.78 (0.48 to 1.39)	-0.16 (-0.21 to -0.09)
**Low SDI**	High body-mass index	0.62 (0.38 to 1.11)	0.85 (0.52 to 1.52)	0.38 (0.35 to 0.42)
	Occupational exposure to benzene	0.65 (0.39 to 1.18)	0.84 (0.52 to 1.51)	0.3 (0.19 to 0.43)
	Occupational exposure to formaldehyde	0.6 (0.37 to 1.09)	0.75 (0.46 to 1.33)	0.25 (0.2 to 0.3)
**Andean Latin America**	High body-mass index	19.52 (14.44 to 25.91)	32.07 (24.33 to 41)	0.64 (0.46 to 0.88)
	Occupational exposure to benzene	23.02 (17.33 to 30.86)	35.69 (28.32 to 44.06)	0.55 (0.39 to 0.77)
	Occupational exposure to formaldehyde	18.33 (14.19 to 23.74)	26.17 (20.51 to 33)	0.43 (0.3 to 0.58)

**Australasia**	High body-mass index	19.31<br>(14.7 to 25.34)	29.43<br>(23.69 to 37.24)	0.52<br>(0.4 to 0.69)
	Occupational exposure to benzene	20.77<br>(15.74 to 27.47)	30.15<br>(24.02 to 37.99)	0.45<br>(0.35 to 0.59)
	Occupational exposure to formaldehyde	20.26<br>(14.76 to 27.15)	30.57<br>(23.5 to 39.48)	0.51<br>(0.38 to 0.67)
**Caribbean**	High body-mass index	7.75<br>(5.33 to 10.67)	11.66<br>(8.92 to 14.94)	0.5<br>(0.34 to 0.77)
	Occupational exposure to benzene	4.81<br>(2.41 to 8.52)	13.15<br>(9.15 to 18.36)	1.73<br>(1.08 to 2.9)
	Occupational exposure to formaldehyde	20.9<br>(16.58 to 26.54)	29.27<br>(23.84 to 36.17)	0.4<br>(0.29 to 0.53)
**Central Asia**	High body-mass index	7.37<br>(5.02 to 10.54)	14.39<br>(11.42 to 18.04)	0.95<br>(0.68 to 1.33)
	Occupational exposure to benzene	11.09<br>(7.96 to 15.23)	19.45<br>(15.57 to 24.39)	0.75<br>(0.57 to 0.98)
	Occupational exposure to formaldehyde	19.27<br>(14.41 to 25.31)	30.94<br>(24.88 to 37.92)	0.61<br>(0.47 to 0.79)
**Central Europe**	High body-mass index	9.03<br>(5.69 to 13.64)	13<br>(9.26 to 17.8)	0.44<br>(0.27 to 0.71)
	Occupational exposure to benzene	26.81<br>(20.11 to 34.83)	41.05<br>(32 to 49.52)	0.53<br>(0.4 to 0.7)
	Occupational exposure to formaldehyde	14.05<br>(10.35 to 18.86)	23.2<br>(18.43 to 29.42)	0.65<br>(0.51 to 0.82)
**Central Latin America**	High body-mass index	5.76<br>(3.74 to 8.56)	11.37<br>(8.71 to 14.68)	0.97<br>(0.69 to 1.37)
	Occupational exposure to benzene	5.51<br>(3.62 to 8.33)	13.86<br>(11 to 17.39)	1.51<br>(1.06 to 2.13)
	Occupational exposure to formaldehyde	9.01<br>(6.27 to 12.76)	20.07<br>(16.21 to 24.94)	1.23<br>(0.91 to 1.63)
**Central Sub-Saharan Africa**	High body-mass index	18.93<br>(14.29 to 24.89)	33.31<br>(26.43 to 41.45)	0.76<br>(0.59 to 0.99)
	Occupational exposure to benzene	14.28<br>(9.76 to 19.9)	17.26<br>(12.66 to 23.08)	0.21<br>(0.12 to 0.33)
	Occupational exposure to formaldehyde	4.23<br>(2.69 to 6.41)	11.45<br>(8.79 to 13.99)	1.71<br>(1.14 to 2.54)
**East Asia**	High body-mass index	5.07<br>(3.11 to 7.76)	14.49<br>(11.5 to 17.85)	1.86<br>(1.22 to 2.78)
	Occupational exposure to benzene	14.29<br>(9.68 to 20.31)	26.17<br>(19.68 to 34.74)	0.83<br>(0.59 to 1.23)
	Occupational exposure to formaldehyde	20.92<br>(16.54 to 26.09)	31.34<br>(25.67 to 37.64)	0.5<br>(0.39 to 0.65)
**Eastern Europe**	High body-mass index	14.9<br>(10.46 to 20.59)	31.16<br>(25.45 to 39.39)	1.09<br>(0.78 to 1.6)
	Occupational exposure to benzene	18.35<br>(13.85 to 24.06)	26.46<br>(21.05 to 33.75)	0.44<br>(0.34 to 0.6)

	Occupational exposure to formaldehyde	8.09 (5.51 to 11.51)	18.55 (14.94 to 23.36)	1.29 (0.94 to 1.87)
**Eastern Sub-Saharan Africa**	High body-mass index	1.16 (0.72 to 2.06)	1.55 (0.96 to 2.78)	0.33 (0.26 to 0.42)
	Occupational exposure to benzene	0.81 (0.05 to 2.33)	1.04 (0.06 to 3)	0.28 (0.25 to 0.36)
	Occupational exposure to formaldehyde	0.81 (0.46 to 1.51)	1.13 (0.67 to 2.07)	0.39 (0.33 to 0.5)
**High-income Asia Pacific**	High body-mass index	0.94 (0.58 to 1.7)	1.25 (0.78 to 2.26)	0.33 (0.29 to 0.39)
	Occupational exposure to benzene	0.76 (0.35 to 1.59)	0.96 (0.49 to 1.9)	0.26 (0.19 to 0.42)
	Occupational exposure to formaldehyde	0.91 (0.09 to 2.55)	1.13 (0.17 to 3.09)	0.24 (0.21 to 0.89)
**High-income North America**	High body-mass index	0.84 (0.05 to 2.42)	1.07 (0.06 to 3.16)	0.27 (0.25 to 0.33)
	Occupational exposure to benzene	1 (0.06 to 2.87)	1.12 (0.07 to 3.19)	0.11 (0.07 to 0.18)
	Occupational exposure to formaldehyde	0.76 (0.32 to 1.65)	0.97 (0.46 to 1.99)	0.27 (0.2 to 0.47)
**North Africa and Middle East**	High body-mass index	1.28 (1.22 to 1.34)	1.47 (1.41 to 1.53)	0.15 (0.09 to 0.21)
	Occupational exposure to benzene	0.25 (0.24 to 0.26)	0.28 (0.26 to 0.3)	0.11 (0.05 to 0.19)
	Occupational exposure to formaldehyde	0.85 (0.82 to 0.89)	1.02 (0.98 to 1.06)	0.2 (0.13 to 0.26)
**Oceania**	High body-mass index	0.76 (0.73 to 0.78)	0.85 (0.83 to 0.87)	0.12 (0.08 to 0.16)
	Occupational exposure to benzene	0.27 (0.26 to 0.28)	0.26 (0.26 to 0.27)	-0.02 (-0.05 to 0.02)
	Occupational exposure to formaldehyde	1.2 (1.16 to 1.24)	1.35 (1.31 to 1.41)	0.13 (0.08 to 0.18)
**South Asia**	High body-mass index	0.99 (0.93 to 1.05)	1.1 (1.04 to 1.17)	0.12 (0.03 to 0.21)
	Occupational exposure to benzene	1.06 (1.01 to 1.09)	1.23 (1.13 to 1.34)	0.17 (0.07 to 0.29)
	Occupational exposure to formaldehyde	0.25 (0.23 to 0.26)	0.24 (0.23 to 0.25)	-0.04 (-0.1 to 0.03)
**Southeast Asia**	High body-mass index	1.06 (1.03 to 1.1)	1.2 (1.16 to 1.23)	0.13 (0.08 to 0.18)
	Occupational exposure to benzene	0.78 (0.76 to 0.79)	0.93 (0.9 to 0.95)	0.19 (0.15 to 0.23)
	Occupational exposure to formaldehyde	0.36 (0.35 to 0.37)	0.41 (0.4 to 0.42)	0.14 (0.11 to 0.17)
**Southern Latin**	High body-mass index	0.32 (0.31 to 0.34)	0.31 (0.29 to 0.32)	-0.06 (-0.11 to 0)

**America**	Occupational exposure to benzene	0.29 (0.28 to 0.3)	0.3 (0.28 to 0.31)	0.02 (-0.04 to 0.08)
	Occupational exposure to formaldehyde	0.84 (0.81 to 0.86)	1.04 (0.98 to 1.1)	0.24 (0.17 to 0.32)
**Southern Sub-Saharan Africa**	High body-mass index	0.76 (0.74 to 0.78)	0.9 (0.87 to 0.92)	0.18 (0.14 to 0.21)
	Occupational exposure to benzene	0.79 (0.77 to 0.81)	0.92 (0.89 to 0.95)	0.17 (0.13 to 0.21)
	Occupational exposure to formaldehyde	0.96 (0.94 to 0.99)	1.07 (1.03 to 1.11)	0.11 (0.07 to 0.15)
**Tropical Latin America**	High body-mass index	0.73 (0.71 to 0.75)	0.79 (0.77 to 0.8)	0.07 (0.04 to 0.11)
	Occupational exposure to benzene	0.64 (0.6 to 0.68)	0.71 (0.67 to 0.76)	0.12 (0.02 to 0.22)
	Occupational exposure to formaldehyde	0.76 (0.73 to 0.79)	0.86 (0.82 to 0.91)	0.14 (0.07 to 0.22)
**Western Europe**	High body-mass index	1.01 (0.97 to 1.04)	1.24 (1.2 to 1.28)	0.23 (0.18 to 0.29)
	Occupational exposure to benzene	1.22 (1.16 to 1.3)	1.25 (1.21 to 1.3)	0.02 (-0.04 to 0.09)
	Occupational exposure to formaldehyde	0.97 (0.91 to 1.03)	0.72 (0.7 to 0.75)	-0.25 (-0.3 to -0.19)
**Western Sub-Saharan Africa**	High body-mass index	1.05 (0.97 to 1.12)	1.13 (1.07 to 1.2)	0.08 (-0.01 to 0.18)
	Occupational exposure to benzene	0.27 (0.26 to 0.27)	0.27 (0.26 to 0.27)	0 (-0.02 to 0.02)
	Occupational exposure to formaldehyde	0.96 (0.92 to 1)	0.88 (0.85 to 0.91)	-0.08 (-0.13 to -0.04)

SEVs are presented for all levels of the risk factor hierarchy, and are measured on a scale of 0 to 100, where 100 indicates that the entire population is exposed to the maximum risk, and 0 indicates that the entire population is at minimum risk.
Abbreviations: ARC, annualised rate of change. SEVs=summary exposure values. UI, uncertainty interval.

Supplementary Table S3. The global incident cases, deaths, and their change trends of leukemia from 1990 to 2019.

Regions	Incidence (95% UI)				1990-2019	Deaths (95% UI)				1990-2019
	1990		2019			1990		2019		
	Incident cases No.×10^3	ASR per 100000	Incident cases No.×10^3	ASR per 100000	EAPC No. (95%CI)	Deaths No.×10^3	ASR per 100000	Deaths No.×10^3	ASR per 100000	EAPC No. (95%CI)
**Global**	474.92 (388.56 to 560.55)	9.6 (8.14 to 11.02)	643.58 (586.98 to 699.73)	8.22 (7.5 to 8.94)	-0.68 (-0.74 to -0.62)	263.26 (233.66 to 298.7)	5.82 (5.25 to 6.44)	334.59 (306.82 to 360.21)	4.26 (3.91 to 4.58)	-1.15 (-1.19 to -1.11)
**High SDI**	104.37 (100.13 to 106.82)	11.18 (10.74 to 11.45)	188.54 (169.16 to 208.21)	11.99 (10.87 to 13.21)	0.05 (-0.1 to 0.2)	58.95 (56.28 to 60.25)	5.96 (5.7 to 6.09)	85.26 (76.98 to 89.68)	4.61 (4.27 to 4.82)	-0.94 (-0.98 to -0.89)
**High-middle SDI**	114.95 (99.42 to 127.24)	10.54 (9.12 to 11.69)	167.72 (150.62 to 183.33)	10.11 (9.05 to 11.14)	-0.36 (-0.45 to -0.27)	65.41 (59.75 to 69.78)	6.06 (5.55 to 6.46)	75.83 (68.74 to 81.31)	4.21 (3.8 to 4.53)	-1.42 (-1.51 to -1.32)
**Middle SDI**	149.08 (113.02 to 179.04)	9.01 (7.16 to 10.62)	163.72 (144.03 to 183.74)	6.9 (6.08 to 7.74)	-1.1 (-1.21 to -0.99)	77.56 (66.82 to 87.91)	5.46 (4.79 to 6.11)	92.24 (81.5 to 103.86)	3.9 (3.45 to 4.38)	-1.22 (-1.25 to -1.18)
**Low-middle SDI**	69.81 (46.71 to 97.16)	6.28 (4.65 to 8.11)	74.17 (64.99 to 86.01)	4.72 (4.16 to 5.45)	-1.13 (-1.19 to -1.06)	40.24 (30.71 to 51.62)	4.42 (3.61 to 5.34)	51.12 (45.29 to 59.13)	3.45 (3.07 to 3.98)	-1 (-1.06 to -0.93)
**Low SDI**	36.47 (20.05 to 60.21)	6.43 (4.4 to 9.37)	49.05 (37.94 to 60.8)	5.1 (4.09 to 6.02)	-0.77 (-0.81 to -0.73)	20.96 (13.45 to 32.73)	4.78 (3.67 to 6.51)	29.92 (24.14 to 35.5)	3.88 (3.12 to 4.56)	-0.75 (-0.78 to -0.73)
**Andean Latin America**	2.72 (2.22 to 3.53)	7.44 (6.28 to 9.37)	4.42 (3.23 to 5.62)	7.3 (5.33 to 9.25)	0.01 (-0.15 to 0.17)	1.65 (1.44 to 2.06)	5.32 (4.69 to 6.5)	3.05 (2.26 to 3.81)	5.15 (3.83 to 6.42)	0.01 (-0.12 to 0.14)
**Australasia**	2.18 (2.07 to 2.28)	9.77 (9.28 to 10.21)	4.72 (3.81 to 5.8)	10.46 (8.44 to 12.78)	-0.01 (-0.14 to 0.13)	1.37 (1.31 to 1.41)	6.04 (5.76 to 6.25)	2.37 (2.12 to 2.57)	4.83 (4.39 to 5.2)	-0.92 (-1.03 to -0.81)
**Caribbean**	2.77 (2.1 to 3.83)	8.22 (6.52 to 10.88)	3.61 (2.84 to 4.45)	7.56 (5.83 to 9.49)	-0.16 (-0.27 to -0.05)	1.71 (1.43 to 2.12)	5.51 (4.77 to 6.58)	2.36 (1.95 to 2.81)	4.82 (3.95 to 5.81)	-0.35 (-0.44 to -0.27)

**Central Asia**	6.37 (5.72 to 6.84)	8.87 (8.11 to 9.41)	5.25 (4.61 to 6.05)	6.05 (5.35 to 6.93)	-1.53 (-1.64 to -1.43)	3.31 (3.18 to 3.44)	5.21 (5.02 to 5.38)	3.19 (2.85 to 3.6)	3.89 (3.5 to 4.37)	-1.14 (-1.21 to -1.07)
**Central Europe**	10.48 (10.18 to 10.87)	7.93 (7.67 to 8.23)	17.32 (15.24 to 19.6)	9.59 (8.45 to 10.87)	0.79 (0.73 to 0.85)	7.63 (7.41 to 7.92)	5.58 (5.42 to 5.81)	9.59 (8.46 to 10.79)	4.85 (4.29 to 5.45)	-0.44 (-0.51 to -0.36)
**Central Latin America**	11.1 (10.54 to 11.66)	7.03 (6.77 to 7.3)	15.98 (13.6 to 18.62)	6.58 (5.6 to 7.67)	-0.2 (-0.23 to -0.17)	6.51 (6.28 to 6.76)	4.85 (4.68 to 5.02)	10.72 (9.28 to 12.35)	4.45 (3.85 to 5.13)	-0.28 (-0.32 to -0.24)
**Central Sub-Saharan Africa**	3.3 (1.49 to 6.03)	5.45 (3.57 to 8.22)	4.09 (3.06 to 5.53)	3.89 (2.91 to 4.98)	-1.09 (-1.14 to -1.04)	1.64 (0.99 to 2.58)	3.79 (3.05 to 4.76)	2.42 (1.84 to 3.06)	2.99 (2.18 to 4.04)	-0.79 (-0.81 to -0.76)
**East Asia**	147.56 (106.05 to 178.26)	12.68 (9.23 to 15.23)	159.36 (131.9 to 185.88)	10.41 (8.69 to 12.26)	-1.04 (-1.22 to -0.87)	68.81 (56.22 to 79.72)	6.25 (5.17 to 7.2)	62.98 (52.7 to 73.47)	3.69 (3.12 to 4.28)	-1.99 (-2.1 to -1.88)
**Eastern Europe**	19.67 (18.89 to 20.41)	8.18 (7.83 to 8.53)	21.62 (19.53 to 23.85)	7.54 (6.86 to 8.29)	-0.58 (-0.8 to -0.35)	13.57 (13.2 to 13.9)	5.43 (5.27 to 5.57)	11.75 (10.64 to 12.9)	3.9 (3.55 to 4.28)	-1.55 (-1.72 to -1.38)
**Eastern Sub-Saharan Africa**	19.12 (10.21 to 33.79)	8.26 (5.3 to 13.2)	21.55 (14 to 29.46)	5.85 (4.15 to 7.71)	-1.11 (-1.21 to -1.02)	10.24 (6.15 to 17.77)	5.87 (4.19 to 8.84)	12.06 (8.4 to 15.92)	4.37 (3.19 to 5.61)	-1.03 (-1.1 to -0.96)
**High-income Asia Pacific**	15.85 (14.85 to 16.73)	9.03 (8.38 to 9.61)	29.2 (24.93 to 33.27)	10.33 (8.98 to 11.7)	0.5 (0.42 to 0.58)	8.59 (8.25 to 8.83)	4.61 (4.4 to 4.75)	12.12 (10.46 to 13.03)	3.02 (2.72 to 3.22)	-1.48 (-1.55 to -1.42)
**High-income North America**	39.06 (37.17 to 40.1)	11.82 (11.26 to 12.14)	61.55 (53.74 to 70.24)	10.69 (9.38 to 12.13)	-0.55 (-0.65 to -0.45)	23.44 (22.17 to 24.09)	6.8 (6.46 to 6.98)	34.71 (32.03 to 36.51)	5.65 (5.28 to 5.92)	-0.74 (-0.78 to -0.7)
**North Africa and Middle East**	29.19 (20.05 to 39.89)	9.51 (7.1 to 11.96)	39.3 (32.62 to 45.06)	7.76 (6.54 to 8.84)	-0.69 (-0.75 to -0.63)	16.92 (13.27 to 21.05)	6.99 (5.58 to 8.19)	25.14 (21.11 to 28.83)	5.41 (4.62 to 6.13)	-0.88 (-0.93 to -0.83)
**Oceania**	0.41 (0.27 to 0.61)	7.11 (5.19 to 9.77)	0.81 (0.53 to 1.22)	6.84 (4.8 to 9.75)	-0.16 (-0.24 to -0.09)	0.23 (0.18 to 0.32)	5.2 (4.08 to 6.71)	0.46 (0.33 to 0.64)	4.79 (3.58 to 6.48)	-0.34 (-0.38 to -0.3)

**South Asia**	46.31 (30.34 to 64.72)	4.75 (3.57 to 6.1)	59.86 (51.91 to 70.1)	3.81 (3.32 to 4.45)	-0.98 (-1.07 to -0.89)	31.17 (23.27 to 40.74)	3.81 (3.1 to 4.62)	44.55 (38.75 to 52.63)	2.98 (2.59 to 3.5)	-1.11 (-1.21 to -1.01)
**Southeast Asia**	37.67 (25.23 to 53.04)	8.64 (6.3 to 11.33)	42.26 (35.84 to 49.69)	6.81 (5.8 to 7.99)	-0.83 (-0.85 to -0.8)	20.85 (16.12 to 26.13)	5.77 (4.72 to 6.91)	28.5 (24.41 to 32.96)	4.74 (4.05 to 5.47)	-0.72 (-0.76 to -0.68)
**Southern Latin America**	3.61 (3.48 to 3.74)	7.52 (7.24 to 7.78)	5.39 (4.27 to 6.7)	7.2 (5.71 to 8.93)	-0.25 (-0.32 to -0.17)	2.72 (2.64 to 2.8)	5.82 (5.61 to 6)	3.84 (3.59 to 4.06)	4.9 (4.6 to 5.18)	-0.73 (-0.83 to -0.64)
**Southern Sub-Saharan Africa**	1.88 (1.61 to 2.14)	4.62 (4.02 to 5.11)	2.76 (2.38 to 3.14)	4.34 (3.71 to 4.86)	-0.2 (-0.31 to -0.09)	1.23 (1.09 to 1.36)	3.57 (3.08 to 4.01)	1.96 (1.65 to 2.17)	3.35 (2.78 to 3.73)	-0.19 (-0.39 to 0)
**Tropical Latin America**	9.35 (8.71 to 9.99)	6.86 (6.48 to 7.23)	12.37 (11.56 to 13.08)	5.53 (5.14 to 5.88)	-0.6 (-0.66 to -0.54)	5.8 (5.55 to 6.07)	4.85 (4.66 to 5.04)	9.07 (8.44 to 9.5)	3.97 (3.69 to 4.19)	-0.56 (-0.61 to -0.52)
**Western Europe**	60.01 (57.77 to 61.72)	12.71 (12.25 to 13.14)	118.62 (102.9 to 135.44)	16.87 (14.68 to 19.38)	0.71 (0.41 to 1.01)	31.67 (30.3 to 32.41)	5.95 (5.7 to 6.08)	45 (39.92 to 47.62)	4.91 (4.46 to 5.15)	-0.69 (-0.74 to -0.63)
**Western Sub-Saharan Africa**	6.33 (4.29 to 9.21)	3.82 (2.97 to 4.85)	13.56 (10.27 to 17.28)	3.9 (3.14 to 4.68)	0.21 (0.16 to 0.26)	4.19 (3.11 to 5.59)	3.13 (2.54 to 3.73)	8.78 (6.93 to 10.83)	3.12 (2.54 to 3.72)	0.1 (0.06 to 0.14)

Abbreviations: ASR, age-standardised rate; EAPC, estimated annual percentage change; UI, uncertainty interval. CI, confidence interval.

Supplementary Table S4. The global incident cases, deaths, and their change trends of multiple myeloma from 1990 to 2019.

Regions	Incidence (95% UI)				1990-2019	Deaths (95% UI)				1990-2019
	1990		2019			1990		2019		
	Incident cases No.×10³	ASR per 100000	Incident cases No.×10³	ASR per 100000	EAPC No. (95%CI)	Deaths No.×10³	ASR per 100000	Deaths No.×10³	ASR per 100000	EAPC No. (95%CI)
**Global**	65.94 (60.78 to 74.06)	1.73 (1.59 to 1.93)	155.69 (136.59 to 172.58)	1.92 (1.68 to 2.12)	0.25 (0.15 to 0.35)	51.86 (47.71 to 58.98)	1.4 (1.28 to 1.58)	113.47 (99.53 to 121.74)	1.42 (1.24 to 1.52)	-0.07 (-0.15 to 0.01)
**High SDI**	34.05 (30.13 to 36.15)	3.23 (2.84 to 3.43)	72.25 (62.61 to 82.52)	3.77 (3.29 to 4.33)	0.33 (0.17 to 0.49)	24.95 (22.29 to 26.3)	2.35 (2.09 to 2.48)	48.11 (41.27 to 51.24)	2.4 (2.1 to 2.59)	-0.17 (-0.29 to -0.04)
**High-middle SDI**	16.02 (14.85 to 18.2)	1.51 (1.4 to 1.71)	37.36 (30.96 to 41.84)	1.83 (1.52 to 2.05)	0.64 (0.54 to 0.75)	12.6 (11.62 to 14.4)	1.21 (1.12 to 1.39)	27.22 (22.94 to 29.56)	1.34 (1.13 to 1.45)	0.3 (0.2 to 0.4)
**Middle SDI**	8.65 (7.54 to 10.84)	0.84 (0.74 to 1.04)	26.72 (22 to 30.38)	1.06 (0.88 to 1.21)	0.83 (0.79 to 0.87)	7.65 (6.7 to 9.51)	0.77 (0.68 to 0.95)	21.14 (17.47 to 24.14)	0.86 (0.71 to 0.99)	0.39 (0.36 to 0.42)
**Low-middle SDI**	4.9 (4.13 to 6.7)	0.82 (0.7 to 1.11)	13.76 (11.92 to 15.91)	1 (0.87 to 1.16)	0.68 (0.65 to 0.71)	4.48 (3.8 to 6.04)	0.79 (0.67 to 1.04)	11.95 (10.18 to 14.02)	0.89 (0.76 to 1.05)	0.44 (0.42 to 0.47)
**Low SDI**	2.27 (1.78 to 3.07)	0.97 (0.77 to 1.29)	5.48 (4.32 to 6.39)	1.07 (0.84 to 1.23)	0.34 (0.32 to 0.36)	2.13 (1.67 to 2.87)	0.95 (0.74 to 1.26)	4.97 (3.94 to 5.77)	1.01 (0.8 to 1.17)	0.2 (0.18 to 0.22)
**Andean Latin America**	0.3 (0.24 to 0.42)	1.48 (1.19 to 2.06)	0.96 (0.75 to 1.21)	1.71 (1.34 to 2.18)	0.74 (0.62 to 0.85)	0.27 (0.22 to 0.38)	1.38 (1.12 to 1.94)	0.79 (0.62 to 1)	1.43 (1.13 to 1.8)	0.34 (0.22 to 0.46)
**Australasia**	1.01 (0.87 to 1.09)	4.26 (3.68 to 4.6)	2.66 (2.1 to 3.39)	5.33 (4.21 to 6.8)	0.61 (0.42 to 0.79)	0.66 (0.58 to 0.7)	2.8 (2.45 to 2.99)	1.54 (1.33 to 1.74)	2.97 (2.59 to 3.39)	0.08 (-0.02 to 0.17)
**Caribbean**	0.66 (0.57 to 0.78)	2.55 (2.21 to 3.02)	1.69 (1.4 to 1.99)	3.25 (2.7 to 3.84)	0.94 (0.89 to 0.99)	0.53 (0.46 to 0.63)	2.05 (1.79 to 2.43)	1.22 (1.02 to 1.44)	2.36 (1.97 to 2.79)	0.58 (0.53 to 0.64)
**Central Asia**	0.28 (0.24 to 0.32)	0.6 (0.5 to 0.68)	0.61 (0.53 to 0.69)	0.8 (0.7 to 0.89)	1.1 (1 to 1.21)	0.24 (0.2 to 0.28)	0.53 (0.44 to 0.6)	0.5 (0.44 to 0.56)	0.69 (0.61 to 0.78)	0.99 (0.93 to 1.06)

**Central Europe**	2.32 (2.2 to 2.68)	1.55 (1.47 to 1.79)	4.55 (3.6 to 5.25)	2.13 (1.69 to 2.46)	1.1 (0.93 to 1.27)	2 (1.89 to 2.33)	1.35 (1.28 to 1.59)	3.92 (3.1 to 4.5)	1.79 (1.42 to 2.06)	0.96 (0.8 to 1.12)
**Central Latin America**	0.96 (0.9 to 1.07)	1.14 (1.07 to 1.28)	3.93 (3.27 to 4.64)	1.65 (1.38 to 1.96)	1.17 (1.08 to 1.25)	0.82 (0.77 to 0.92)	1 (0.93 to 1.13)	3.04 (2.53 to 3.59)	1.3 (1.08 to 1.53)	0.82 (0.76 to 0.88)
**Central Sub-Saharan Africa**	0.26 (0.18 to 0.35)	1.16 (0.81 to 1.54)	0.63 (0.39 to 0.86)	1.2 (0.74 to 1.64)	0.12 (0.05 to 0.18)	0.25 (0.17 to 0.33)	1.15 (0.8 to 1.5)	0.57 (0.35 to 0.78)	1.16 (0.71 to 1.56)	0.02 (-0.03 to 0.08)
**East Asia**	6.37 (5.24 to 8.92)	0.71 (0.59 to 0.99)	19.71 (14.45 to 24.29)	0.94 (0.69 to 1.16)	1.05 (0.99 to 1.11)	5.58 (4.56 to 7.8)	0.64 (0.54 to 0.89)	14.11 (10.74 to 17.15)	0.68 (0.52 to 0.83)	0.21 (0.16 to 0.26)
**Eastern Europe**	3.14 (2.78 to 3.59)	1.1 (0.97 to 1.28)	5.36 (4.64 to 6.08)	1.57 (1.35 to 1.78)	1.4 (1.24 to 1.56)	2.48 (2.22 to 2.94)	0.88 (0.78 to 1.06)	3.99 (3.37 to 4.51)	1.15 (0.97 to 1.31)	1.05 (0.91 to 1.19)
**Eastern Sub-Saharan Africa**	0.85 (0.62 to 1.12)	1.16 (0.86 to 1.49)	2.03 (1.44 to 2.5)	1.28 (0.91 to 1.56)	0.38 (0.35 to 0.4)	0.81 (0.59 to 1.05)	1.15 (0.85 to 1.47)	1.89 (1.35 to 2.31)	1.25 (0.89 to 1.52)	0.34 (0.32 to 0.37)
**High-income Asia Pacific**	3.53 (3.19 to 3.89)	1.77 (1.6 to 1.96)	9.19 (7.13 to 10.89)	1.96 (1.58 to 2.3)	0.37 (0.22 to 0.52)	2.69 (2.44 to 2.98)	1.36 (1.23 to 1.51)	6.56 (5.16 to 7.28)	1.32 (1.07 to 1.45)	-0.31 (-0.46 to -0.16)
**High-income North America**	15.26 (12.82 to 15.88)	4.32 (3.62 to 4.5)	30.39 (26.02 to 36.83)	4.8 (4.12 to 5.87)	0.11 (-0.02 to 0.25)	11.2 (9.44 to 11.63)	3.11 (2.62 to 3.23)	19.94 (18.01 to 22.92)	3.07 (2.8 to 3.58)	-0.31 (-0.42 to -0.21)
**North Africa and Middle East**	2.2 (1.76 to 2.93)	1.29 (1.04 to 1.7)	6.37 (4.99 to 7.64)	1.49 (1.17 to 1.77)	0.52 (0.42 to 0.63)	1.95 (1.56 to 2.59)	1.19 (0.97 to 1.56)	5.01 (3.94 to 5.97)	1.22 (0.96 to 1.44)	0.13 (0.03 to 0.22)
**Oceania**	0.03 (0.02 to 0.05)	1.02 (0.68 to 1.68)	0.07 (0.05 to 0.11)	1.01 (0.69 to 1.55)	-0.08 (-0.12 to -0.05)	0.03 (0.02 to 0.05)	0.96 (0.64 to 1.56)	0.06 (0.04 to 0.1)	0.94 (0.64 to 1.43)	-0.12 (-0.15 to -0.09)
**South Asia**	4.52 (3.54 to 5.65)	0.82 (0.64 to 1.01)	13.37 (9.79 to 15.94)	0.95 (0.69 to 1.13)	0.49 (0.45 to 0.54)	4.15 (3.26 to 5.22)	0.79 (0.62 to 0.98)	11.73 (8.61 to 13.93)	0.86 (0.62 to 1.02)	0.24 (0.19 to 0.3)
**Southeast Asia**	1.82 (1.54 to 2.48)	0.71 (0.6 to 0.97)	5 (4.17 to 6.59)	0.82 (0.68 to 1.09)	0.48 (0.43 to 0.53)	1.63 (1.39 to 2.2)	0.66 (0.56 to 0.89)	4.19 (3.5 to 5.59)	0.71 (0.59 to 0.95)	0.23 (0.19 to 0.27)

**Southern Latin America**	0.96 (0.84 to 1.09)	2.07 (1.82 to 2.34)	2.21 (1.73 to 2.84)	2.66 (2.08 to 3.41)	0.71 (0.57 to 0.85)	0.83 (0.73 to 0.92)	1.8 (1.61 to 2.02)	1.74 (1.58 to 2)	2.07 (1.88 to 2.37)	0.32 (0.19 to 0.45)
**Southern Sub-Saharan Africa**	0.51 (0.4 to 0.58)	1.83 (1.45 to 2.1)	1.27 (0.9 to 1.47)	2.24 (1.6 to 2.59)	0.71 (0.52 to 0.91)	0.45 (0.36 to 0.52)	1.7 (1.33 to 1.95)	1.11 (0.79 to 1.28)	2.04 (1.45 to 2.35)	0.67 (0.47 to 0.88)
**Tropical Latin America**	1.3 (1.23 to 1.49)	1.41 (1.33 to 1.63)	5 (4.35 to 5.39)	2.06 (1.78 to 2.22)	1.3 (1.14 to 1.46)	1.1 (1.04 to 1.28)	1.23 (1.15 to 1.45)	4.02 (3.4 to 4.31)	1.67 (1.41 to 1.8)	1.07 (0.93 to 1.22)
**Western Europe**	18.98 (17.48 to 21.57)	3.26 (3 to 3.67)	38.98 (31.73 to 44.71)	4.24 (3.51 to 4.9)	0.68 (0.46 to 0.9)	13.56 (12.54 to 15.48)	2.28 (2.1 to 2.59)	26 (21.28 to 27.91)	2.64 (2.22 to 2.82)	0.28 (0.11 to 0.44)
**Western Sub-Saharan Africa**	0.69 (0.56 to 0.88)	0.79 (0.64 to 1.01)	1.7 (1.34 to 2.07)	0.91 (0.72 to 1.11)	0.57 (0.52 to 0.61)	0.65 (0.53 to 0.83)	0.77 (0.63 to 0.98)	1.54 (1.19 to 1.88)	0.86 (0.67 to 1.05)	0.46 (0.42 to 0.49)

Abbreviations: ASR, age-standardised rate; EAPC, estimated annual percentage change; UI, uncertainty interval. CI, confidence interval.

Supplementary Table S5. The global incident cases, deaths, and their change trends of Non-Hodgkin lymphoma from 1990 to 2019.

Regions	Incidence (95% UI)				1990-2019	Deaths (95% UI)				1990-2019
	1990		2019			1990		2019		
	Incident cases No.×10^3	ASR per 100000	Incident cases No.×10^3	ASR per 100000	EAPC No. (95%CI)	Deaths No.×10^3	ASR per 100000	Deaths No.×10^3	ASR per 100000	EAPC No. (95%CI)
**Global**	190.73 (179.03 to 203.62)	4.65 (4.37 to 4.93)	457.08 (416.89 to 498.78)	5.73 (5.21 to 6.25)	0.56 (0.45 to 0.66)	126.08 (119.77 to 131.98)	3.15 (3 to 3.29)	254.61 (237.71 to 270.35)	3.19 (2.98 to 3.39)	-0.09 (-0.17 to -0.02)
**High SDI**	98.35 (92.37 to 104.1)	9.71 (9.15 to 10.25)	184.16 (161.68 to 206.62)	9.93 (8.84 to 11.11)	-0.37 (-0.56 to -0.18)	52.44 (50.26 to 53.64)	5.09 (4.88 to 5.2)	84.47 (76.27 to 89.26)	4.33 (3.97 to 4.55)	-0.98 (-1.15 to -0.82)
**High-middle SDI**	41.73 (39.17 to 44.57)	3.83 (3.59 to 4.09)	110.05 (98.42 to 122.54)	5.86 (5.25 to 6.49)	1.48 (1.36 to 1.6)	27.84 (26.64 to 28.92)	2.61 (2.49 to 2.71)	54.72 (50.52 to 58.45)	2.81 (2.6 to 3)	0.19 (0.08 to 0.29)
**Middle SDI**	28.46 (25.4 to 32.02)	2.18 (1.99 to 2.4)	101.65 (91.21 to 113.6)	4.13 (3.71 to 4.61)	2.53 (2.37 to 2.69)	23.99 (22.13 to 25.7)	2.05 (1.91 to 2.18)	62.23 (56.93 to 68.34)	2.55 (2.33 to 2.81)	0.94 (0.84 to 1.03)
**Low-middle SDI**	15.89 (13.37 to 19.49)	2.09 (1.84 to 2.43)	45.39 (40.34 to 51.62)	3.05 (2.73 to 3.43)	1.31 (1.26 to 1.37)	15.09 (13.12 to 17.46)	2.18 (1.95 to 2.46)	38.24 (34.67 to 42.42)	2.74 (2.49 to 3.03)	0.83 (0.81 to 0.86)
**Low SDI**	6.19 (5.11 to 7.7)	2.1 (1.82 to 2.42)	15.59 (12.91 to 18.76)	2.36 (1.98 to 2.78)	0.41 (0.36 to 0.46)	6.63 (5.41 to 7.85)	2.34 (2.04 to 2.65)	14.8 (12.72 to 17.09)	2.51 (2.12 to 2.93)	0.26 (0.21 to 0.3)
**Andean Latin America**	0.86 (0.76 to 0.97)	3.34 (2.99 to 3.73)	3.59 (2.91 to 4.48)	6.21 (5.03 to 7.76)	2.41 (2.24 to 2.57)	0.83 (0.74 to 0.92)	3.51 (3.16 to 3.9)	2.64 (2.16 to 3.21)	4.68 (3.84 to 5.69)	1.14 (0.96 to 1.32)
**Australasia**	3.01 (2.79 to 3.24)	12.95 (12.03 to 13.91)	5.54 (4.38 to 6.87)	11.31 (8.97 to 14.07)	-1.12 (-1.39 to -0.85)	1.49 (1.41 to 1.57)	6.41 (6.03 to 6.73)	2.33 (2.04 to 2.58)	4.57 (4.04 to 5.05)	-1.78 (-2.01 to -1.56)
**Caribbean**	1.59 (1.47 to 1.71)	5.39 (5.02 to 5.78)	2.82 (2.37 to 3.32)	5.58 (4.69 to 6.58)	0.17 (0.02 to 0.32)	1.21 (1.12 to 1.29)	4.26 (3.98 to 4.51)	1.86 (1.58 to 2.16)	3.65 (3.11 to 4.25)	-0.47 (-0.66 to -0.28)

**Central Asia**	1 (0.87 to 1.18)	1.73 (1.54 to 1.99)	2.45 (2.12 to 2.84)	2.96 (2.58 to 3.39)	1.74 (1.55 to 1.92)	0.71 (0.68 to 0.74)	1.34 (1.28 to 1.4)	1.51 (1.35 to 1.71)	1.92 (1.71 to 2.16)	1.06 (0.89 to 1.23)
**Central Europe**	5.54 (5.18 to 5.93)	3.99 (3.73 to 4.27)	12.27 (10.74 to 14.12)	6.49 (5.71 to 7.43)	1.76 (1.55 to 1.96)	3.77 (3.65 to 3.91)	2.67 (2.58 to 2.76)	6.32 (5.57 to 7.14)	3.13 (2.77 to 3.54)	0.58 (0.42 to 0.75)
**Central Latin America**	3.23 (3.01 to 3.5)	2.82 (2.66 to 3.02)	10.4 (8.91 to 12.17)	4.32 (3.71 to 5.05)	1.46 (1.33 to 1.59)	2.67 (2.58 to 2.75)	2.64 (2.54 to 2.73)	6.79 (5.9 to 7.86)	2.86 (2.49 to 3.32)	0.28 (0.22 to 0.34)
**Central Sub-Saharan Africa**	0.66 (0.52 to 0.81)	2.26 (1.74 to 2.82)	1.67 (1.18 to 2.26)	2.34 (1.61 to 3.32)	0.17 (-0.01 to 0.35)	0.76 (0.6 to 0.96)	2.58 (2.05 to 3.1)	1.61 (1.15 to 2.17)	2.51 (1.73 to 3.58)	-0.05 (-0.22 to 0.12)
**East Asia**	21.61 (18.55 to 25.57)	2.12 (1.85 to 2.46)	95.93 (81.21 to 113.06)	5.04 (4.31 to 5.89)	3.57 (3.29 to 3.86)	17.42 (15.44 to 19.55)	1.86 (1.66 to 2.08)	46.45 (39.66 to 54)	2.35 (2.02 to 2.71)	1.16 (0.95 to 1.36)
**Eastern Europe**	7.62 (7.1 to 8.23)	3.05 (2.83 to 3.31)	13.38 (11.91 to 14.96)	4.61 (4.12 to 5.13)	1.77 (1.48 to 2.07)	4.93 (4.76 to 5.12)	1.89 (1.82 to 1.96)	7.18 (6.45 to 7.93)	2.29 (2.06 to 2.53)	0.75 (0.52 to 0.97)
**Eastern Sub-Saharan Africa**	2.16 (1.74 to 2.67)	2.41 (2.08 to 2.76)	5.41 (4.49 to 6.55)	2.78 (2.37 to 3.27)	0.58 (0.49 to 0.66)	2.4 (1.89 to 2.91)	2.79 (2.41 to 3.2)	5.5 (4.66 to 6.53)	3.19 (2.76 to 3.69)	0.57 (0.47 to 0.66)
**High-income Asia Pacific**	12.23 (11.5 to 12.94)	6.3 (5.93 to 6.66)	36.24 (30.09 to 42.22)	8.06 (6.89 to 9.34)	0.7 (0.51 to 0.89)	6.49 (6.23 to 6.65)	3.34 (3.19 to 3.43)	16.28 (13.79 to 17.64)	3.34 (2.94 to 3.57)	-0.18 (-0.33 to -0.04)
**High-income North America**	45.61 (42.72 to 48.3)	13.22 (12.42 to 14)	69.46 (59.74 to 79.49)	11.28 (9.74 to 12.9)	-1.2 (-1.43 to -0.97)	23.74 (22.64 to 24.35)	6.75 (6.46 to 6.92)	33.58 (31 to 35.37)	5.27 (4.9 to 5.53)	-1.46 (-1.67 to -1.26)
**North Africa and Middle East**	7.67 (6.32 to 9.3)	3.4 (2.87 to 3.97)	22.61 (19.76 to 25.81)	4.72 (4.15 to 5.38)	1.22 (1.08 to 1.36)	6.59 (5.53 to 7.64)	3.34 (2.83 to 3.79)	14.26 (12.44 to 16.24)	3.17 (2.75 to 3.6)	-0.18 (-0.31 to -0.05)
**Oceania**	0.06 (0.05 to 0.07)	1.6 (1.37 to 1.84)	0.14 (0.12 to 0.17)	1.71 (1.46 to 2.03)	0.18 (0.11 to 0.25)	0.06 (0.05 to 0.07)	1.65 (1.42 to 1.88)	0.13 (0.11 to 0.15)	1.7 (1.45 to 2)	0.1 (0.03 to 0.16)
**South Asia**	11.36 (9.17 to 14.82)	1.56 (1.32 to 1.88)	38.92 (32.86 to	2.53 (2.15 to 3.01)	1.68 (1.61 to 1.74)	10.51 (8.93 to 12.27)	1.6 (1.41 to 1.84)	32.62 (28.05 to	2.28 (1.97 to 2.66)	1.25 (1.18 to 1.32)

			47.02)					37.95)		
**Southeast Asia**	8.83 (7.57 to 10.28)	2.77 (2.44 to 3.17)	24.13 (20.67 to 28.16)	3.88 (3.34 to 4.49)	1.11 (1.07 to 1.16)	8.45 (7.32 to 9.78)	2.89 (2.58 to 3.28)	19.89 (17.32 to 22.88)	3.31 (2.91 to 3.79)	0.44 (0.4 to 0.47)
**Southern Latin America**	2.37 (2.18 to 2.57)	5.02 (4.61 to 5.42)	5.3 (4.15 to 6.73)	6.64 (5.19 to 8.44)	0.63 (0.34 to 0.92)	1.95 (1.88 to 2.03)	4.21 (4.03 to 4.36)	3.33 (3.07 to 3.61)	4.06 (3.76 to 4.4)	-0.43 (-0.69 to -0.17)
**Southern Sub-Saharan Africa**	1.09 (0.97 to 1.21)	3.03 (2.69 to 3.35)	2.35 (2.05 to 2.67)	3.58 (3.16 to 4.02)	0.63 (0.48 to 0.78)	1.01 (0.91 to 1.09)	3.07 (2.73 to 3.38)	2.04 (1.83 to 2.28)	3.34 (3.01 to 3.71)	0.41 (0.2 to 0.62)
**Tropical Latin America**	3.51 (3.28 to 3.8)	3.04 (2.87 to 3.25)	9.27 (8.59 to 9.99)	3.92 (3.63 to 4.22)	0.99 (0.8 to 1.18)	3.04 (2.93 to 3.14)	2.93 (2.8 to 3.03)	6.5 (6.07 to 6.86)	2.73 (2.55 to 2.89)	-0.18 (-0.31 to -0.05)
**Western Europe**	48.09 (45.12 to 50.89)	8.95 (8.44 to 9.43)	88.44 (75.34 to 101.72)	10.11 (8.65 to 11.61)	-0.01 (-0.23 to 0.22)	25.58 (24.59 to 26.21)	4.57 (4.4 to 4.68)	38.26 (34.53 to 40.64)	4.07 (3.74 to 4.29)	-0.81 (-0.99 to -0.63)
**Western Sub-Saharan Africa**	2.62 (2.01 to 3.53)	2 (1.63 to 2.44)	6.76 (5.11 to 9)	2.32 (1.88 to 2.85)	0.35 (0.24 to 0.47)	2.47 (1.95 to 3.09)	2.04 (1.71 to 2.43)	5.55 (4.54 to 6.6)	2.24 (1.88 to 2.62)	0.2 (0.1 to 0.3)

Abbreviations: ASR, age-standardised rate; EAPC, estimated annual percentage change; UI, uncertainty interval. CI, confidence interval.

Supplementary Table S6. The global incident cases, deaths, and their change trends of Hodgkin lymphoma from 1990 to 2019.

Regions	Incidence (95% UI)				1990-2019	Deaths (95% UI)				1990-2019
	1990		2019			1990		2019		
	Incident cases No.×10³	ASR per 100000	Incident cases No.×10³	ASR per 100000	EAPC No. (95%CI)	Deaths No.×10³	ASR per 100000	Deaths No.×10³	ASR per 100000	EAPC No. (95%CI)
**Global**	59.69 (48.25 to 64.24)	1.26 (1.02 to 1.35)	87.51 (77.94 to 101.43)	1.1 (0.98 to 1.27)	-0.47 (-0.53 to -0.41)	27.6 (21.66 to 30.23)	0.61 (0.48 to 0.66)	27.55 (23.68 to 31.81)	0.34 (0.29 to 0.4)	-2.08 (-2.17 to -1.98)
**High SDI**	23.7 (19.04 to 25.72)	2.57 (2.06 to 2.78)	33 (28.57 to 39.87)	2.51 (2.19 to 3.16)	-0.07 (-0.11 to -0.03)	4.82 (3.83 to 5.18)	0.49 (0.39 to 0.53)	3.88 (3.42 to 4.65)	0.25 (0.22 to 0.3)	-2.34 (-2.55 to -2.13)
**High-middle SDI**	16.15 (12.68 to 17.64)	1.39 (1.09 to 1.52)	21.99 (19.06 to 25.39)	1.36 (1.18 to 1.57)	-0.19 (-0.26 to -0.11)	7.76 (6.04 to 8.6)	0.69 (0.54 to 0.77)	5.57 (4.81 to 6.38)	0.31 (0.26 to 0.35)	-3.15 (-3.28 to -3.03)
**Middle SDI**	9.35 (6.9 to 10.61)	0.65 (0.48 to 0.74)	15.82 (13.31 to 18.54)	0.64 (0.54 to 0.75)	-0.09 (-0.27 to 0.09)	6.66 (4.86 to 7.61)	0.51 (0.38 to 0.58)	6.6 (5.53 to 7.67)	0.27 (0.22 to 0.31)	-2.29 (-2.4 to -2.17)
**Low-middle SDI**	6.91 (5.13 to 7.86)	0.76 (0.59 to 0.87)	10.02 (8.61 to 12.82)	0.6 (0.51 to 0.76)	-0.99 (-1.09 to -0.89)	5.45 (4.11 to 6.2)	0.66 (0.51 to 0.75)	6.56 (5.57 to 8.37)	0.41 (0.35 to 0.52)	-1.73 (-1.79 to -1.67)
**Low SDI**	3.55 (2.38 to 4.19)	0.93 (0.64 to 1.08)	6.63 (5.06 to 7.84)	0.75 (0.57 to 0.88)	-0.85 (-0.88 to -0.82)	2.9 (1.94 to 3.4)	0.83 (0.58 to 0.97)	4.91 (3.67 to 5.84)	0.61 (0.46 to 0.72)	-1.14 (-1.18 to -1.1)
**Andean Latin America**	0.2 (0.16 to 0.23)	0.67 (0.53 to 0.78)	0.39 (0.29 to 0.49)	0.63 (0.48 to 0.79)	-0.03 (-0.14 to 0.09)	0.15 (0.12 to 0.18)	0.57 (0.45 to 0.66)	0.2 (0.15 to 0.25)	0.35 (0.25 to 0.43)	-1.51 (-1.62 to -1.4)
**Australasia**	0.41 (0.34 to 0.48)	1.9 (1.55 to 2.2)	0.81 (0.62 to 1.06)	2.5 (1.91 to 3.34)	1.11 (1.01 to 1.22)	0.1 (0.09 to 0.12)	0.45 (0.38 to 0.52)	0.11 (0.09 to 0.13)	0.26 (0.22 to 0.31)	-1.64 (-1.91 to -1.36)
**Caribbean**	0.18 (0.16 to 0.25)	0.57 (0.49 to 0.77)	0.57 (0.35 to 0.71)	1.13 (0.7 to 1.41)	2.4 (1.62 to 3.18)	0.11 (0.09 to 0.14)	0.35 (0.31 to 0.46)	0.23 (0.15 to 0.28)	0.46 (0.3 to 0.56)	0.88 (0.29 to 1.47)
**Central Asia**	0.65 (0.54 to	1.04 (0.88 to	0.95 (0.8 to	1.02 (0.86 to	-0.38 (-0.48	0.38 (0.32 to	0.66 (0.56 to	0.41 (0.35 to	0.47 (0.4 to	-1.49 (-1.68

	0.74)	1.2)	1.11)	1.19)	to -0.27)	0.43)	0.76)	0.47)	0.54)	to -1.29)
**Central Europe**	2.72 (2.18 to 2.95)	2.07 (1.65 to 2.25)	2.68 (2.26 to 3.38)	2.09 (1.76 to 2.67)	-0.15 (-0.23 to -0.07)	1.39 (1.13 to 1.5)	1 (0.82 to 1.08)	0.71 (0.59 to 0.89)	0.42 (0.35 to 0.52)	-3.4 (-3.54 to -3.25)
**Central Latin America**	1.38 (1.22 to 1.61)	1.08 (0.95 to 1.26)	2.61 (2.18 to 3.29)	1.04 (0.87 to 1.31)	-0.15 (-0.28 to -0.01)	0.9 (0.8 to 1.04)	0.81 (0.71 to 0.93)	1.13 (0.94 to 1.37)	0.47 (0.39 to 0.57)	-1.9 (-2.1 to -1.7)
**Central Sub-Saharan Africa**	0.18 (0.13 to 0.23)	0.52 (0.39 to 0.66)	0.39 (0.29 to 0.5)	0.46 (0.33 to 0.6)	-0.34 (-0.37 to -0.31)	0.15 (0.11 to 0.19)	0.48 (0.36 to 0.61)	0.31 (0.23 to 0.4)	0.41 (0.29 to 0.53)	-0.46 (-0.51 to -0.41)
**East Asia**	6.83 (3.64 to 8.88)	0.64 (0.34 to 0.83)	9.9 (7.49 to 11.94)	0.58 (0.44 to 0.7)	-0.47 (-0.85 to -0.08)	5.02 (2.63 to 6.55)	0.51 (0.27 to 0.66)	2.81 (2.1 to 3.4)	0.15 (0.11 to 0.17)	-4.68 (-4.94 to -4.42)
**Eastern Europe**	4.96 (3.94 to 5.54)	2.1 (1.65 to 2.33)	5.71 (4.78 to 7.24)	2.61 (2.18 to 3.32)	0.55 (0.38 to 0.71)	2.04 (1.7 to 2.33)	0.81 (0.67 to 0.92)	1.41 (1.21 to 1.76)	0.53 (0.46 to 0.66)	-2.17 (-2.5 to -1.84)
**Eastern Sub-Saharan Africa**	1.31 (0.9 to 1.59)	0.95 (0.67 to 1.12)	2.52 (1.96 to 3.14)	0.79 (0.61 to 0.96)	-0.71 (-0.75 to -0.67)	1.04 (0.71 to 1.26)	0.83 (0.59 to 0.98)	1.83 (1.42 to 2.23)	0.65 (0.5 to 0.78)	-0.91 (-0.95 to -0.86)
**High-income Asia Pacific**	3.9 (3.56 to 4.78)	2.02 (1.83 to 2.48)	9.42 (6.52 to 11.24)	2.46 (1.79 to 2.89)	1.21 (1.03 to 1.38)	0.2 (0.18 to 0.25)	0.1 (0.09 to 0.13)	0.31 (0.22 to 0.34)	0.08 (0.06 to 0.09)	-0.25 (-0.38 to -0.12)
**High-income North America**	10.45 (7.96 to 11.3)	3.38 (2.57 to 3.65)	11.25 (9.15 to 15.61)	2.63 (2.12 to 3.76)	-1.04 (-1.14 to -0.94)	1.95 (1.54 to 2.1)	0.59 (0.47 to 0.64)	1.67 (1.49 to 2.1)	0.31 (0.28 to 0.41)	-2.24 (-2.38 to -2.1)
**North Africa and Middle East**	2.22 (1.87 to 2.89)	0.83 (0.69 to 1.14)	5.8 (4.66 to 6.96)	1.01 (0.83 to 1.22)	0.73 (0.62 to 0.83)	1.5 (1.24 to 2.04)	0.64 (0.52 to 0.92)	2.14 (1.78 to 2.77)	0.41 (0.35 to 0.54)	-1.45 (-1.49 to -1.41)
**Oceania**	0.02 (0.02 to 0.03)	0.45 (0.35 to 0.6)	0.04 (0.03 to 0.05)	0.39 (0.3 to 0.51)	-0.44 (-0.49 to -0.39)	0.02 (0.01 to 0.02)	0.36 (0.29 to 0.49)	0.03 (0.02 to 0.04)	0.29 (0.22 to 0.39)	-0.56 (-0.63 to -0.48)
**South Asia**	7.56 (5.08 to 8.78)	0.85 (0.59 to 0.97)	10.88 (9.05 to 13.93)	0.62 (0.51 to 0.79)	-1.27 (-1.36 to -1.19)	6 (4.12 to 6.91)	0.73 (0.53 to 0.84)	7.26 (5.94 to 9.3)	0.43 (0.35 to 0.56)	-1.97 (-2.04 to -1.91)

**Southeast Asia**	1.84 (1.57 to 2.44)	0.48 (0.41 to 0.65)	3.12 (2.44 to 4.5)	0.46 (0.36 to 0.66)	-0.25 (-0.37 to -0.14)	1.26 (1.07 to 1.7)	0.36 (0.31 to 0.5)	1.43 (1.11 to 2.12)	0.22 (0.17 to 0.32)	-1.77 (-1.8 to -1.74)
**Southern Latin America**	0.51 (0.43 to 0.59)	1.06 (0.9 to 1.23)	0.87 (0.66 to 1.14)	1.18 (0.9 to 1.55)	0.27 (0.06 to 0.48)	0.32 (0.27 to 0.37)	0.67 (0.56 to 0.78)	0.31 (0.27 to 0.37)	0.39 (0.34 to 0.48)	-1.9 (-2.14 to -1.67)
**Southern Sub-Saharan Africa**	0.17 (0.14 to 0.21)	0.4 (0.33 to 0.48)	0.29 (0.23 to 0.36)	0.4 (0.31 to 0.47)	-0.14 (-0.26 to -0.01)	0.12 (0.1 to 0.15)	0.32 (0.26 to 0.38)	0.19 (0.15 to 0.23)	0.28 (0.22 to 0.33)	-0.44 (-0.65 to -0.22)
**Tropical Latin America**	0.97 (0.84 to 1.11)	0.75 (0.65 to 0.85)	1.57 (1.34 to 1.89)	0.66 (0.57 to 0.8)	-0.24 (-0.33 to -0.15)	0.65 (0.57 to 0.74)	0.55 (0.47 to 0.63)	0.7 (0.6 to 0.84)	0.29 (0.25 to 0.35)	-2.04 (-2.16 to -1.92)
**Western Europe**	12.15 (9.43 to 13.22)	2.79 (2.16 to 3.03)	15.1 (12.84 to 18.7)	2.96 (2.51 to 3.74)	0.23 (0.13 to 0.32)	3.4 (2.73 to 3.7)	0.67 (0.54 to 0.73)	2.43 (2.08 to 2.91)	0.33 (0.29 to 0.4)	-2.42 (-2.62 to -2.22)
**Western Sub-Saharan Africa**	1.09 (0.52 to 1.55)	0.84 (0.4 to 1.21)	2.64 (1.13 to 4.14)	0.79 (0.34 to 1.21)	-0.11 (-0.21 to 0)	0.92 (0.44 to 1.31)	0.77 (0.36 to 1.1)	1.96 (0.82 to 3.02)	0.66 (0.29 to 0.99)	-0.45 (-0.53 to -0.37)

Abbreviations: ASR, age-standardised rate; EAPC, estimated annual percentage change; UI, uncertainty interval. CI, confidence interval.

Supplementary Table S7. The change of hematological malignancies between 1990 and 2019 at national level, both sexes.

Countries	Causes	Measure	Cases in 1990 No.×10^3	Cases in 2019 No.×10^3	Change in absolute number (100%)	ASR in 1990 per 100000	ASR in 2019 per 100000	EAPC from 1990 and 2019
**Afghanistan**	**Leukemia**	Incidence	1.67	3.36	1.01	14.71	12.36	-0.4
**Afghanistan**	**Leukemia**	Deaths	1.06	2.12	1.01	11.28	10.01	-0.3
**Afghanistan**	**Multiple myeloma**	Incidence	0.11	0.19	0.71	1.56	1.54	-0.03
**Afghanistan**	**Multiple myeloma**	Deaths	0.11	0.17	0.62	1.52	1.46	-0.12
**Afghanistan**	**Non-Hodgkin lymphoma**	Incidence	0.93	1.97	1.13	12.46	13.36	0.37
**Afghanistan**	**Non-Hodgkin lymphoma**	Deaths	1	2	1.01	13.75	14.84	0.39
**Afghanistan**	**Hodgkin lymphoma**	Incidence	0.12	0.28	1.4	1.35	1.17	-0.57
**Afghanistan**	**Hodgkin lymphoma**	Deaths	0.1	0.22	1.12	1.25	1.03	-0.72
**Albania**	**Leukemia**	Incidence	0.2	0.28	0.39	6.69	9.39	1.39
**Albania**	**Leukemia**	Deaths	0.12	0.15	0.23	4.47	4.19	-0.1
**Albania**	**Multiple myeloma**	Incidence	0.01	0.04	1.37	0.72	0.83	0.75
**Albania**	**Multiple myeloma**	Deaths	0.01	0.03	1.28	0.65	0.68	0.43
**Albania**	**Non-Hodgkin lymphoma**	Incidence	0.06	0.13	1.14	2.22	3.43	1.76
**Albania**	**Non-Hodgkin lymphoma**	Deaths	0.04	0.06	0.56	1.74	1.63	0.01
**Albania**	**Hodgkin lymphoma**	Incidence	0.03	0.05	0.57	1.14	1.85	1.71
**Albania**	**Hodgkin lymphoma**	Deaths	0.02	0.01	-0.24	0.7	0.4	-2.08
**Algeria**	**Leukemia**	Incidence	1.14	1.51	0.32	5.54	4.2	-0.96
**Algeria**	**Leukemia**	Deaths	0.72	1.01	0.4	4.34	3.02	-1.22
**Algeria**	**Multiple myeloma**	Incidence	0.14	0.43	2.17	1.18	1.33	0.43
**Algeria**	**Multiple myeloma**	Deaths	0.12	0.35	1.92	1.11	1.14	0.14

Countries	Causes	Measure	Cases in 1990 No.×10^3	Cases in 2019 No.×10^3	Change in absolute number (100%)	ASR in 1990 per 100000	ASR in 2019 per 100000	EAPC from 1990 and 2019
**Algeria**	**Non-Hodgkin lymphoma**	Incidence	0.92	2.15	1.34	5.27	6.31	0.68
**Algeria**	**Non-Hodgkin lymphoma**	Deaths	0.69	1.32	0.91	5.08	4.14	-0.63
**Algeria**	**Hodgkin lymphoma**	Incidence	0.35	0.67	0.92	1.61	1.63	0.05
**Algeria**	**Hodgkin lymphoma**	Deaths	0.21	0.22	0.08	1.11	0.58	-2.05
**American Samoa**	**Leukemia**	Incidence	0	0	0.22	4.3	3.77	-0.56
**American Samoa**	**Leukemia**	Deaths	0	0	0.36	3.53	2.96	-0.75
**American Samoa**	**Multiple myeloma**	Incidence	0	0	0.9	1.56	1.39	-0.57
**American Samoa**	**Multiple myeloma**	Deaths	0	0	0.88	1.41	1.22	-0.69
**American Samoa**	**Non-Hodgkin lymphoma**	Incidence	0	0	1.32	2.47	3.46	1.62
**American Samoa**	**Non-Hodgkin lymphoma**	Deaths	0	0	1.19	2.43	2.92	1.04
**American Samoa**	**Hodgkin lymphoma**	Incidence	0	0	0.85	0.22	0.28	1.02
**American Samoa**	**Hodgkin lymphoma**	Deaths	0	0	0.68	0.15	0.16	0.27
**Andorra**	**Leukemia**	Incidence	0.01	0.03	2.28	15.6	22.71	1.1
**Andorra**	**Leukemia**	Deaths	0	0.01	1.17	7.74	6.28	-0.8
**Andorra**	**Multiple myeloma**	Incidence	0	0.01	1.55	4.74	4.62	-0.17
**Andorra**	**Multiple myeloma**	Deaths	0	0	1.44	3.57	3.09	-0.57
**Andorra**	**Non-Hodgkin lymphoma**	Incidence	0.01	0.02	1.55	14.06	14.86	0.12
**Andorra**	**Non-Hodgkin lymphoma**	Deaths	0	0.01	1.19	6.78	5.66	-0.67
**Andorra**	**Hodgkin lymphoma**	Incidence	0	0	1.3	2.53	3.74	1.19
**Andorra**	**Hodgkin lymphoma**	Deaths	0	0	0.38	0.54	0.36	-1.51
**Angola**	**Leukemia**	Incidence	0.72	1.12	0.56	6.19	4.4	-1.17

Countries	Causes	Measure	Cases in 1990 No.×10^3	Cases in 2019 No.×10^3	Change in absolute number (100%)	ASR in 1990 per 100000	ASR in 2019 per 100000	EAPC from 1990 and 2019
**Angola**	**Leukemia**	Deaths	0.35	0.62	0.76	4.16	3.31	-0.79
**Angola**	**Multiple myeloma**	Incidence	0.04	0.14	2.43	1.09	1.32	0.71
**Angola**	**Multiple myeloma**	Deaths	0.04	0.13	2.27	1.08	1.26	0.58
**Angola**	**Non-Hodgkin lymphoma**	Incidence	0.13	0.47	2.53	2.51	2.97	0.76
**Angola**	**Non-Hodgkin lymphoma**	Deaths	0.16	0.45	1.81	2.88	3.18	0.51
**Angola**	**Hodgkin lymphoma**	Incidence	0.04	0.08	1.29	0.56	0.44	-0.85
**Angola**	**Hodgkin lymphoma**	Deaths	0.03	0.06	1.11	0.52	0.39	-1.06
**Antigua and Barbuda**	**Leukemia**	Incidence	0	0.01	0.57	5.9	6.07	0.1
**Antigua and Barbuda**	**Leukemia**	Deaths	0	0	0.59	4.33	4.27	-0.15
**Antigua and Barbuda**	**Multiple myeloma**	Incidence	0	0	1.6	3.02	4.17	1.11
**Antigua and Barbuda**	**Multiple myeloma**	Deaths	0	0	1.3	2.45	3.1	0.82
**Antigua and Barbuda**	**Non-Hodgkin lymphoma**	Incidence	0	0.01	0.99	4.8	5.46	0.37
**Antigua and Barbuda**	**Non-Hodgkin lymphoma**	Deaths	0	0	0.6	3.98	3.55	-0.43
**Antigua and Barbuda**	**Hodgkin lymphoma**	Incidence	0	0	0.87	0.25	0.28	0.41
**Antigua and Barbuda**	**Hodgkin lymphoma**	Deaths	0	0	0.31	0.16	0.12	-0.97
**Argentina**	**Leukemia**	Incidence	2.54	3.52	0.39	7.75	7.1	-0.53
**Argentina**	**Leukemia**	Deaths	1.94	2.66	0.37	6.02	5.16	-0.76
**Argentina**	**Multiple myeloma**	Incidence	0.59	1.16	0.96	1.83	2.17	0.37
**Argentina**	**Multiple myeloma**	Deaths	0.52	0.93	0.8	1.6	1.71	0.05
**Argentina**	**Non-Hodgkin lymphoma**	Incidence	1.61	3.33	1.08	4.93	6.41	0.41
**Argentina**	**Non-Hodgkin lymphoma**	Deaths	1.36	2.2	0.62	4.22	4.15	-0.46

Countries	Causes	Measure	Cases in 1990 No.×10^3	Cases in 2019 No.×10^3	Change in absolute number (100%)	ASR in 1990 per 100000	ASR in 2019 per 100000	EAPC from 1990 and 2019
**Argentina**	**Hodgkin lymphoma**	Incidence	0.34	0.54	0.61	1.04	1.11	-0.11
**Argentina**	**Hodgkin lymphoma**	Deaths	0.22	0.22	-0.01	0.69	0.43	-1.81
**Armenia**	**Leukemia**	Incidence	0.33	0.26	-0.21	9.79	7.62	-1.16
**Armenia**	**Leukemia**	Deaths	0.19	0.16	-0.18	6.07	4.34	-1.56
**Armenia**	**Multiple myeloma**	Incidence	0.02	0.04	1.24	0.57	0.85	1.79
**Armenia**	**Multiple myeloma**	Deaths	0.01	0.03	1.19	0.49	0.69	1.54
**Armenia**	**Non-Hodgkin lymphoma**	Incidence	0.05	0.12	1.49	1.53	3.15	3.24
**Armenia**	**Non-Hodgkin lymphoma**	Deaths	0.03	0.07	1.02	1.17	1.74	1.93
**Armenia**	**Hodgkin lymphoma**	Incidence	0.01	0.02	0.94	0.32	0.62	2.5
**Armenia**	**Hodgkin lymphoma**	Deaths	0.01	0.01	0.24	0.21	0.21	0.09
**Australia**	**Leukemia**	Incidence	1.76	3.88	1.21	9.43	10.08	-0.04
**Australia**	**Leukemia**	Deaths	1.13	1.99	0.75	6.02	4.79	-0.95
**Australia**	**Multiple myeloma**	Incidence	0.81	2.18	1.69	4.13	5.18	0.61
**Australia**	**Multiple myeloma**	Deaths	0.55	1.3	1.37	2.78	2.96	0.1
**Australia**	**Non-Hodgkin lymphoma**	Incidence	2.57	4.7	0.83	13.29	11.33	-1.21
**Australia**	**Non-Hodgkin lymphoma**	Deaths	1.27	1.97	0.56	6.55	4.57	-1.86
**Australia**	**Hodgkin lymphoma**	Incidence	0.36	0.72	0.97	2.02	2.63	1.11
**Australia**	**Hodgkin lymphoma**	Deaths	0.08	0.09	0.04	0.44	0.25	-1.72
**Austria**	**Leukemia**	Incidence	1.02	2.13	1.09	10.09	14.84	1.23
**Austria**	**Leukemia**	Deaths	0.59	0.83	0.41	5.26	4.64	-0.26
**Austria**	**Multiple myeloma**	Incidence	0.3	0.61	1.01	2.53	3.39	0.97

Countries	Causes	Measure	Cases in 1990 No.×10^3	Cases in 2019 No.×10^3	Change in absolute number (100%)	ASR in 1990 per 100000	ASR in 2019 per 100000	EAPC from 1990 and 2019
**Austria**	**Multiple myeloma**	Deaths	0.23	0.43	0.88	1.88	2.29	0.67
**Austria**	**Non-Hodgkin lymphoma**	Incidence	0.77	1.58	1.04	6.83	9.05	0.97
**Austria**	**Non-Hodgkin lymphoma**	Deaths	0.42	0.69	0.64	3.56	3.71	0.23
**Austria**	**Hodgkin lymphoma**	Incidence	0.27	0.22	-0.2	2.93	1.93	-2.08
**Austria**	**Hodgkin lymphoma**	Deaths	0.09	0.04	-0.58	0.81	0.24	-4.6
**Azerbaijan**	**Leukemia**	Incidence	0.85	0.83	-0.03	10.97	8.88	-1.01
**Azerbaijan**	**Leukemia**	Deaths	0.38	0.44	0.14	5.41	4.62	-0.89
**Azerbaijan**	**Multiple myeloma**	Incidence	0.03	0.07	1.38	0.55	0.7	0.93
**Azerbaijan**	**Multiple myeloma**	Deaths	0.02	0.06	1.3	0.49	0.63	0.92
**Azerbaijan**	**Non-Hodgkin lymphoma**	Incidence	0.09	0.26	2.05	1.37	2.72	2.9
**Azerbaijan**	**Non-Hodgkin lymphoma**	Deaths	0.06	0.17	1.65	1.11	1.8	2.14
**Azerbaijan**	**Hodgkin lymphoma**	Incidence	0.09	0.15	0.67	1.22	1.35	-0.2
**Azerbaijan**	**Hodgkin lymphoma**	Deaths	0.05	0.06	0.19	0.8	0.6	-1.55
**Bahamas**	**Leukemia**	Incidence	0.01	0.02	0.72	5.35	5.07	-0.11
**Bahamas**	**Leukemia**	Deaths	0.01	0.01	0.74	4.1	3.67	-0.32
**Bahamas**	**Multiple myeloma**	Incidence	0.01	0.03	1.94	5.64	6.49	0.74
**Bahamas**	**Multiple myeloma**	Deaths	0.01	0.02	1.75	4.56	4.91	0.51
**Bahamas**	**Non-Hodgkin lymphoma**	Incidence	0.01	0.02	1.04	5.54	5.73	0.29
**Bahamas**	**Non-Hodgkin lymphoma**	Deaths	0.01	0.02	0.82	4.72	3.95	-0.48
**Bahamas**	**Hodgkin lymphoma**	Incidence	0	0	0.69	0.75	0.74	-0.18
**Bahamas**	**Hodgkin lymphoma**	Deaths	0	0	0.38	0.47	0.34	-1.33

Countries	Causes	Measure	Cases in 1990 No.×10^3	Cases in 2019 No.×10^3	Change in absolute number (100%)	ASR in 1990 per 100000	ASR in 2019 per 100000	EAPC from 1990 and 2019
**Bahrain**	**Leukemia**	Incidence	0.02	0.07	2.11	8.98	7.67	-1.12
**Bahrain**	**Leukemia**	Deaths	0.02	0.04	1.53	8.01	5.49	-1.88
**Bahrain**	**Multiple myeloma**	Incidence	0	0.02	3.38	2.64	2.01	-1.14
**Bahrain**	**Multiple myeloma**	Deaths	0	0.01	2.67	2.42	1.65	-1.52
**Bahrain**	**Non-Hodgkin lymphoma**	Incidence	0.01	0.07	5.49	4.26	7.11	2.54
**Bahrain**	**Non-Hodgkin lymphoma**	Deaths	0.01	0.03	3.2	4.15	4.1	0.55
**Bahrain**	**Hodgkin lymphoma**	Incidence	0.01	0.02	2.33	1.73	1.45	-0.97
**Bahrain**	**Hodgkin lymphoma**	Deaths	0	0.01	0.42	1.28	0.43	-4.38
**Bangladesh**	**Leukemia**	Incidence	5.71	4.54	-0.21	5.47	3.2	-1.89
**Bangladesh**	**Leukemia**	Deaths	3.59	3.49	-0.03	4.25	2.55	-1.79
**Bangladesh**	**Multiple myeloma**	Incidence	0.34	0.98	1.85	0.73	0.75	0.16
**Bangladesh**	**Multiple myeloma**	Deaths	0.32	0.86	1.69	0.7	0.67	-0.06
**Bangladesh**	**Non-Hodgkin lymphoma**	Incidence	1.01	2.67	1.65	1.47	1.86	0.85
**Bangladesh**	**Non-Hodgkin lymphoma**	Deaths	0.93	2.14	1.3	1.51	1.61	0.3
**Bangladesh**	**Hodgkin lymphoma**	Incidence	0.83	0.83	0.01	0.96	0.53	-2.18
**Bangladesh**	**Hodgkin lymphoma**	Deaths	0.65	0.53	-0.2	0.84	0.35	-3.04
**Barbados**	**Leukemia**	Incidence	0.02	0.03	0.46	7.23	7.25	0.03
**Barbados**	**Leukemia**	Deaths	0.01	0.02	0.47	5.3	5.14	-0.16
**Barbados**	**Multiple myeloma**	Incidence	0.02	0.04	1.39	6.13	8.57	1.22
**Barbados**	**Multiple myeloma**	Deaths	0.01	0.03	1.14	4.84	6.17	0.88
**Barbados**	**Non-Hodgkin lymphoma**	Incidence	0.02	0.04	0.72	8.45	9.61	0.29

Countries	Causes	Measure	Cases in 1990 No.×10^3	Cases in 2019 No.×10^3	Change in absolute number (100%)	ASR in 1990 per 100000	ASR in 2019 per 100000	EAPC from 1990 and 2019
**Barbados**	**Non-Hodgkin lymphoma**	Deaths	0.02	0.03	0.43	6.53	5.87	-0.49
**Barbados**	**Hodgkin lymphoma**	Incidence	0	0	0.33	0.79	0.84	-0.22
**Barbados**	**Hodgkin lymphoma**	Deaths	0	0	0	0.45	0.31	-1.63
**Belarus**	**Leukemia**	Incidence	1.21	1.22	0.01	11.14	9.31	-0.89
**Belarus**	**Leukemia**	Deaths	0.85	0.64	-0.24	7.46	4.58	-1.93
**Belarus**	**Multiple myeloma**	Incidence	0.12	0.36	1.97	0.92	2.26	3.25
**Belarus**	**Multiple myeloma**	Deaths	0.09	0.23	1.54	0.71	1.46	2.61
**Belarus**	**Non-Hodgkin lymphoma**	Incidence	0.25	0.67	1.75	2.15	5.06	3.96
**Belarus**	**Non-Hodgkin lymphoma**	Deaths	0.15	0.33	1.2	1.27	2.3	2.91
**Belarus**	**Hodgkin lymphoma**	Incidence	0.21	0.32	0.54	1.88	3.34	2.11
**Belarus**	**Hodgkin lymphoma**	Deaths	0.09	0.06	-0.31	0.78	0.51	-1.66
**Belgium**	**Leukemia**	Incidence	1.52	2.63	0.72	11.73	13.97	0.5
**Belgium**	**Leukemia**	Deaths	1.03	1.24	0.21	7.06	5.26	-1.05
**Belgium**	**Multiple myeloma**	Incidence	0.58	0.95	0.63	3.69	4.11	0.3
**Belgium**	**Multiple myeloma**	Deaths	0.46	0.7	0.51	2.9	2.83	-0.2
**Belgium**	**Non-Hodgkin lymphoma**	Incidence	1.22	2.16	0.76	8.42	9.65	0.06
**Belgium**	**Non-Hodgkin lymphoma**	Deaths	0.67	0.95	0.41	4.47	3.98	-0.8
**Belgium**	**Hodgkin lymphoma**	Incidence	0.27	0.32	0.19	2.3	2.42	0.17
**Belgium**	**Hodgkin lymphoma**	Deaths	0.09	0.05	-0.38	0.63	0.28	-2.77
**Belize**	**Leukemia**	Incidence	0.01	0.02	0.67	5.35	4.79	-0.53
**Belize**	**Leukemia**	Deaths	0	0.01	1.28	3.28	3.34	-0.13

Countries	Causes	Measure	Cases in 1990 No.×10^3	Cases in 2019 No.×10^3	Change in absolute number (100%)	ASR in 1990 per 100000	ASR in 2019 per 100000	EAPC from 1990 and 2019
**Belize**	**Multiple myeloma**	Incidence	0	0	3.36	0.8	1.17	1.15
**Belize**	**Multiple myeloma**	Deaths	0	0	2.99	0.68	0.92	0.88
**Belize**	**Non-Hodgkin lymphoma**	Incidence	0	0.01	2.69	1.81	2.6	0.97
**Belize**	**Non-Hodgkin lymphoma**	Deaths	0	0.01	2.08	1.71	1.98	0.28
**Belize**	**Hodgkin lymphoma**	Incidence	0	0	2.6	0.7	0.91	0.16
**Belize**	**Hodgkin lymphoma**	Deaths	0	0	1.78	0.51	0.51	-0.76
**Benin**	**Leukemia**	Incidence	0.2	0.5	1.56	4.31	4.64	0.52
**Benin**	**Leukemia**	Deaths	0.12	0.3	1.5	3.4	3.55	0.33
**Benin**	**Multiple myeloma**	Incidence	0.02	0.04	1.77	0.76	0.86	0.48
**Benin**	**Multiple myeloma**	Deaths	0.01	0.04	1.66	0.73	0.81	0.4
**Benin**	**Non-Hodgkin lymphoma**	Incidence	0.07	0.2	1.87	2.43	2.63	-0.06
**Benin**	**Non-Hodgkin lymphoma**	Deaths	0.07	0.18	1.49	2.62	2.7	-0.21
**Benin**	**Hodgkin lymphoma**	Incidence	0.01	0.03	1.63	0.33	0.33	-0.03
**Benin**	**Hodgkin lymphoma**	Deaths	0.01	0.02	1.46	0.29	0.28	-0.15
**Bermuda**	**Leukemia**	Incidence	0	0.01	0.36	8.14	6.97	-0.42
**Bermuda**	**Leukemia**	Deaths	0	0	0.11	6.02	3.59	-1.79
**Bermuda**	**Multiple myeloma**	Incidence	0	0.01	1.24	3.99	4.18	-0.03
**Bermuda**	**Multiple myeloma**	Deaths	0	0	0.9	3	2.6	-0.69
**Bermuda**	**Non-Hodgkin lymphoma**	Incidence	0.01	0.01	0.41	10.6	8.66	-0.68
**Bermuda**	**Non-Hodgkin lymphoma**	Deaths	0	0	-0.01	7.46	3.89	-2.21
**Bermuda**	**Hodgkin lymphoma**	Incidence	0	0	1.42	1.11	2.31	2.35

Countries	Causes	Measure	Cases in 1990 No.×10^3	Cases in 2019 No.×10^3	Change in absolute number (100%)	ASR in 1990 per 100000	ASR in 2019 per 100000	EAPC from 1990 and 2019
**Bermuda**	**Hodgkin lymphoma**	Deaths	0	0	0.11	0.52	0.36	-1.75
**Bhutan**	**Leukemia**	Incidence	0.03	0.03	0.02	4.73	4.2	-0.61
**Bhutan**	**Leukemia**	Deaths	0.02	0.02	0.24	3.71	3.32	-0.52
**Bhutan**	**Multiple myeloma**	Incidence	0	0.01	2.04	0.82	1.12	1.07
**Bhutan**	**Multiple myeloma**	Deaths	0	0.01	1.92	0.8	1.02	0.85
**Bhutan**	**Non-Hodgkin lymphoma**	Incidence	0	0.02	2.51	1.38	2.68	2.35
**Bhutan**	**Non-Hodgkin lymphoma**	Deaths	0	0.01	1.9	1.47	2.39	1.76
**Bhutan**	**Hodgkin lymphoma**	Incidence	0	0	0.21	0.74	0.55	-1.16
**Bhutan**	**Hodgkin lymphoma**	Deaths	0	0	-0.08	0.65	0.36	-2.19
**Bolivia (Plurinational State of)**	**Leukemia**	Incidence	0.64	0.95	0.47	10.1	8.81	-0.57
**Bolivia (Plurinational State of)**	**Leukemia**	Deaths	0.38	0.67	0.76	7.36	6.84	-0.32
**Bolivia (Plurinational State of)**	**Multiple myeloma**	Incidence	0.06	0.19	2.48	1.76	2.22	0.78
**Bolivia (Plurinational State of)**	**Multiple myeloma**	Deaths	0.05	0.17	2.26	1.71	2.01	0.56
**Bolivia (Plurinational State of)**	**Non-Hodgkin lymphoma**	Incidence	0.13	0.48	2.7	3.41	5.23	1.37
**Bolivia (Plurinational State of)**	**Non-Hodgkin lymphoma**	Deaths	0.14	0.47	2.34	3.81	5.34	1.13
**Bolivia (Plurinational State of)**	**Hodgkin lymphoma**	Incidence	0.05	0.08	0.64	1	0.76	-0.98
**Bolivia (Plurinational State of)**	**Hodgkin lymphoma**	Deaths	0.04	0.06	0.43	0.91	0.59	-1.46

Countries	Causes	Measure	Cases in 1990 No.×10^3	Cases in 2019 No.×10^3	Change in absolute number (100%)	ASR in 1990 per 100000	ASR in 2019 per 100000	EAPC from 1990 and 2019
Bosnia and Herzegovina	Leukemia	Incidence	0.2	0.36	0.79	4.73	7.16	1.69
Bosnia and Herzegovina	Leukemia	Deaths	0.15	0.22	0.47	3.65	4.22	0.6
Bosnia and Herzegovina	Multiple myeloma	Incidence	0.05	0.09	0.76	1.19	1.4	0.57
Bosnia and Herzegovina	Multiple myeloma	Deaths	0.04	0.07	0.75	1.05	1.19	0.33
Bosnia and Herzegovina	Non-Hodgkin lymphoma	Incidence	0.09	0.24	1.67	2.02	4.76	3.67
Bosnia and Herzegovina	Non-Hodgkin lymphoma	Deaths	0.07	0.13	1.05	1.54	2.46	2.02
Bosnia and Herzegovina	Hodgkin lymphoma	Incidence	0.05	0.06	0.32	1.02	1.62	1.76
Bosnia and Herzegovina	Hodgkin lymphoma	Deaths	0.03	0.02	-0.14	0.64	0.48	-1.3
Botswana	Leukemia	Incidence	0.04	0.1	1.74	4.32	5.85	0.82
Botswana	Leukemia	Deaths	0.03	0.06	1.48	3.62	4.19	0.2
Botswana	Multiple myeloma	Incidence	0.01	0.03	2.17	1.76	2.28	0.47
Botswana	Multiple myeloma	Deaths	0.01	0.03	1.92	1.65	2.01	0.24
Botswana	Non-Hodgkin lymphoma	Incidence	0.01	0.05	2.87	1.74	2.78	0.98
Botswana	Non-Hodgkin lymphoma	Deaths	0.01	0.04	2.22	1.82	2.42	0.43
Botswana	Hodgkin lymphoma	Incidence	0	0	1.48	0.14	0.15	-0.34
Botswana	Hodgkin lymphoma	Deaths	0	0	0.85	0.12	0.09	-1.28
Brazil	Leukemia	Incidence	9.09	11.99	0.32	6.87	5.52	-0.6
Brazil	Leukemia	Deaths	5.66	8.8	0.56	4.87	3.96	-0.58
Brazil	Multiple myeloma	Incidence	1.28	4.94	2.86	1.42	2.08	1.31
Brazil	Multiple myeloma	Deaths	1.09	3.96	2.65	1.24	1.69	1.08
Brazil	Non-Hodgkin lymphoma	Incidence	3.45	9.04	1.62	3.07	3.92	0.96

Countries	Causes	Measure	Cases in 1990 No.×10^3	Cases in 2019 No.×10^3	Change in absolute number (100%)	ASR in 1990 per 100000	ASR in 2019 per 100000	EAPC from 1990 and 2019
**Brazil**	**Non-Hodgkin lymphoma**	Deaths	2.99	6.33	1.12	2.95	2.73	-0.21
**Brazil**	**Hodgkin lymphoma**	Incidence	0.95	1.52	0.59	0.76	0.66	-0.27
**Brazil**	**Hodgkin lymphoma**	Deaths	0.64	0.67	0.06	0.56	0.29	-2.08
**Brunei Darussalam**	**Leukemia**	Incidence	0.02	0.03	0.93	8.79	9.2	0.52
**Brunei Darussalam**	**Leukemia**	Deaths	0.01	0.02	0.76	6.38	5.63	-0.06
**Brunei Darussalam**	**Multiple myeloma**	Incidence	0	0.01	3.43	2.56	3.68	2.18
**Brunei Darussalam**	**Multiple myeloma**	Deaths	0	0.01	3	2.25	3.01	1.95
**Brunei Darussalam**	**Non-Hodgkin lymphoma**	Incidence	0.01	0.04	1.87	10.07	13.13	1.48
**Brunei Darussalam**	**Non-Hodgkin lymphoma**	Deaths	0.01	0.02	1.47	8.24	8.41	0.73
**Brunei Darussalam**	**Hodgkin lymphoma**	Incidence	0	0.01	4.6	1.39	3.4	3.95
**Brunei Darussalam**	**Hodgkin lymphoma**	Deaths	0	0	1.76	0.37	0.45	1.6
**Bulgaria**	**Leukemia**	Incidence	0.73	0.84	0.15	7.82	8.17	0.6
**Bulgaria**	**Leukemia**	Deaths	0.49	0.56	0.16	4.63	4.75	0.66
**Bulgaria**	**Multiple myeloma**	Incidence	0.1	0.18	0.75	0.82	1.37	1.89
**Bulgaria**	**Multiple myeloma**	Deaths	0.08	0.14	0.69	0.64	0.97	1.63
**Bulgaria**	**Non-Hodgkin lymphoma**	Incidence	0.36	0.62	0.72	3.42	5.61	2.16
**Bulgaria**	**Non-Hodgkin lymphoma**	Deaths	0.23	0.35	0.52	2.08	2.89	1.56
**Bulgaria**	**Hodgkin lymphoma**	Incidence	0.18	0.17	-0.1	1.96	2.12	0.42
**Bulgaria**	**Hodgkin lymphoma**	Deaths	0.09	0.06	-0.34	0.87	0.58	-1.31
**Burkina Faso**	**Leukemia**	Incidence	0.36	1.06	1.97	4.02	5.06	0.95
**Burkina Faso**	**Leukemia**	Deaths	0.22	0.6	1.69	3.2	3.73	0.62

Countries	Causes	Measure	Cases in 1990 No.×10^3	Cases in 2019 No.×10^3	Change in absolute number (100%)	ASR in 1990 per 100000	ASR in 2019 per 100000	EAPC from 1990 and 2019
**Burkina Faso**	**Multiple myeloma**	Incidence	0.04	0.09	1.41	0.81	0.95	0.49
**Burkina Faso**	**Multiple myeloma**	Deaths	0.03	0.08	1.32	0.79	0.89	0.39
**Burkina Faso**	**Non-Hodgkin lymphoma**	Incidence	0.12	0.35	1.84	2.13	2.56	0.22
**Burkina Faso**	**Non-Hodgkin lymphoma**	Deaths	0.13	0.33	1.5	2.36	2.67	0.04
**Burkina Faso**	**Hodgkin lymphoma**	Incidence	0.02	0.05	1.57	0.33	0.35	0.21
**Burkina Faso**	**Hodgkin lymphoma**	Deaths	0.02	0.04	1.38	0.3	0.31	0.06
**Burundi**	**Leukemia**	Incidence	0.37	0.52	0.41	5.36	4.49	-0.42
**Burundi**	**Leukemia**	Deaths	0.19	0.27	0.46	3.7	3.26	-0.41
**Burundi**	**Multiple myeloma**	Incidence	0.04	0.06	0.59	1.57	1.3	-0.92
**Burundi**	**Multiple myeloma**	Deaths	0.04	0.05	0.53	1.56	1.27	-0.98
**Burundi**	**Non-Hodgkin lymphoma**	Incidence	0.07	0.14	0.91	2.61	2.43	-0.38
**Burundi**	**Non-Hodgkin lymphoma**	Deaths	0.08	0.14	0.67	3	2.73	-0.47
**Burundi**	**Hodgkin lymphoma**	Incidence	0.04	0.08	1.01	0.93	0.87	-0.35
**Burundi**	**Hodgkin lymphoma**	Deaths	0.03	0.06	0.9	0.81	0.73	-0.5
**Cabo Verde**	**Leukemia**	Incidence	0.02	0.03	0.69	4.4	5.45	0.23
**Cabo Verde**	**Leukemia**	Deaths	0.01	0.02	0.94	3.13	4.04	0.29
**Cabo Verde**	**Multiple myeloma**	Incidence	0	0	2.25	0.54	0.93	1.33
**Cabo Verde**	**Multiple myeloma**	Deaths	0	0	1.97	0.49	0.81	1.05
**Cabo Verde**	**Non-Hodgkin lymphoma**	Incidence	0	0.02	5.23	1.28	4.45	3.74
**Cabo Verde**	**Non-Hodgkin lymphoma**	Deaths	0	0.02	3.67	1.28	3.3	2.64
**Cabo Verde**	**Hodgkin lymphoma**	Incidence	0	0	2.09	0.1	0.17	1.31

Countries	Causes	Measure	Cases in 1990 No.×10^3	Cases in 2019 No.×10^3	Change in absolute number (100%)	ASR in 1990 per 100000	ASR in 2019 per 100000	EAPC from 1990 and 2019
**Cabo Verde**	**Hodgkin lymphoma**	Deaths	0	0	1.38	0.08	0.11	0.26
**Cambodia**	**Leukemia**	Incidence	1.28	1.2	-0.06	11.91	8.24	-1.32
**Cambodia**	**Leukemia**	Deaths	0.65	0.78	0.2	8.08	5.92	-1.17
**Cambodia**	**Multiple myeloma**	Incidence	0.04	0.13	2.29	0.87	1.09	0.73
**Cambodia**	**Multiple myeloma**	Deaths	0.04	0.11	2.11	0.84	0.99	0.52
**Cambodia**	**Non-Hodgkin lymphoma**	Incidence	0.13	0.38	1.94	2.06	2.84	1.14
**Cambodia**	**Non-Hodgkin lymphoma**	Deaths	0.13	0.35	1.66	2.19	2.78	0.88
**Cambodia**	**Hodgkin lymphoma**	Incidence	0.04	0.06	0.49	0.6	0.43	-1.19
**Cambodia**	**Hodgkin lymphoma**	Deaths	0.03	0.04	0.21	0.52	0.3	-1.95
**Cameroon**	**Leukemia**	Incidence	0.35	1.1	2.15	4.28	4.96	0.69
**Cameroon**	**Leukemia**	Deaths	0.23	0.69	1.99	3.53	3.81	0.4
**Cameroon**	**Multiple myeloma**	Incidence	0.04	0.13	2.05	0.98	1.12	0.45
**Cameroon**	**Multiple myeloma**	Deaths	0.04	0.12	1.92	0.95	1.04	0.32
**Cameroon**	**Non-Hodgkin lymphoma**	Incidence	0.23	0.82	2.59	3.28	3.8	0.43
**Cameroon**	**Non-Hodgkin lymphoma**	Deaths	0.19	0.54	1.91	3.16	3.3	0.08
**Cameroon**	**Hodgkin lymphoma**	Incidence	0.02	0.06	1.94	0.3	0.29	-0.25
**Cameroon**	**Hodgkin lymphoma**	Deaths	0.02	0.05	1.59	0.27	0.23	-0.56
**Canada**	**Leukemia**	Incidence	3.86	8.44	1.19	12.95	15.1	0.57
**Canada**	**Leukemia**	Deaths	1.99	3.33	0.67	6.34	4.96	-0.75
**Canada**	**Multiple myeloma**	Incidence	1.45	3.34	1.3	4.46	4.82	0.06
**Canada**	**Multiple myeloma**	Deaths	0.95	2	1.11	2.9	2.79	-0.34

Countries	Causes	Measure	Cases in 1990 No.×10^3	Cases in 2019 No.×10^3	Change in absolute number (100%)	ASR in 1990 per 100000	ASR in 2019 per 100000	EAPC from 1990 and 2019
**Canada**	**Non-Hodgkin lymphoma**	Incidence	4.1	8.61	1.1	12.86	12.97	-0.44
**Canada**	**Non-Hodgkin lymphoma**	Deaths	2.03	3.59	0.77	6.34	5.16	-1.2
**Canada**	**Hodgkin lymphoma**	Incidence	0.88	1.37	0.55	2.96	3.25	0.56
**Canada**	**Hodgkin lymphoma**	Deaths	0.15	0.14	-0.06	0.48	0.26	-2.05
**Central African Republic**	**Leukemia**	Incidence	0.16	0.23	0.48	5.81	5.02	-0.28
**Central African Republic**	**Leukemia**	Deaths	0.09	0.14	0.56	4.26	3.78	-0.31
**Central African Republic**	**Multiple myeloma**	Incidence	0.02	0.03	0.7	1.35	1.25	-0.23
**Central African Republic**	**Multiple myeloma**	Deaths	0.02	0.03	0.68	1.35	1.25	-0.25
**Central African Republic**	**Non-Hodgkin lymphoma**	Incidence	0.04	0.07	0.8	2.47	2.29	-0.15
**Central African Republic**	**Non-Hodgkin lymphoma**	Deaths	0.04	0.07	0.72	2.76	2.53	-0.2
**Central African Republic**	**Hodgkin lymphoma**	Incidence	0.01	0.02	0.87	0.66	0.64	-0.18
**Central African Republic**	**Hodgkin lymphoma**	Deaths	0.01	0.02	0.86	0.62	0.6	-0.18
**Chad**	**Leukemia**	Incidence	0.21	0.66	2.11	3.7	4.65	1.04
**Chad**	**Leukemia**	Deaths	0.13	0.39	1.9	2.94	3.62	0.92
**Chad**	**Multiple myeloma**	Incidence	0.02	0.04	1.29	0.64	0.73	0.55
**Chad**	**Multiple myeloma**	Deaths	0.02	0.04	1.21	0.62	0.7	0.51
**Chad**	**Non-Hodgkin lymphoma**	Incidence	0.07	0.23	2.16	1.96	2.52	0.63

Countries	Causes	Measure	Cases in 1990 No.×10^3	Cases in 2019 No.×10^3	Change in absolute number (100%)	ASR in 1990 per 100000	ASR in 2019 per 100000	EAPC from 1990 and 2019
**Chad**	**Non-Hodgkin lymphoma**	Deaths	0.08	0.21	1.69	2.14	2.64	0.47
**Chad**	**Hodgkin lymphoma**	Incidence	0.01	0.04	1.89	0.32	0.38	0.7
**Chad**	**Hodgkin lymphoma**	Deaths	0.01	0.03	1.75	0.29	0.35	0.69
**Chile**	**Leukemia**	Incidence	0.76	1.5	0.97	6.35	7.29	0.85
**Chile**	**Leukemia**	Deaths	0.54	0.9	0.66	4.8	4.13	-0.3
**Chile**	**Multiple myeloma**	Incidence	0.26	0.85	2.3	2.61	3.52	0.97
**Chile**	**Multiple myeloma**	Deaths	0.22	0.65	1.98	2.23	2.67	0.47
**Chile**	**Non-Hodgkin lymphoma**	Incidence	0.52	1.52	1.92	4.74	6.65	1.19
**Chile**	**Non-Hodgkin lymphoma**	Deaths	0.4	0.83	1.09	3.86	3.56	-0.36
**Chile**	**Hodgkin lymphoma**	Incidence	0.12	0.26	1.13	1.02	1.31	1.29
**Chile**	**Hodgkin lymphoma**	Deaths	0.07	0.07	-0.02	0.6	0.3	-2.19
**China**	**Leukemia**	Incidence	142.72	154.65	0.08	12.71	10.47	-1.06
**China**	**Leukemia**	Deaths	66.82	60.38	-0.1	6.28	3.67	-2.05
**China**	**Multiple myeloma**	Incidence	6.07	18.79	2.09	0.7	0.93	1.04
**China**	**Multiple myeloma**	Deaths	5.34	13.42	1.51	0.64	0.67	0.17
**China**	**Non-Hodgkin lymphoma**	Incidence	20.08	91.95	3.58	2.04	4.99	3.72
**China**	**Non-Hodgkin lymphoma**	Deaths	16.44	44.31	1.7	1.82	2.32	1.2
**China**	**Hodgkin lymphoma**	Incidence	6.64	9.47	0.43	0.65	0.57	-0.54
**China**	**Hodgkin lymphoma**	Deaths	4.95	2.71	-0.45	0.52	0.15	-4.77
**Colombia**	**Leukemia**	Incidence	2.21	3.37	0.52	7.48	6.98	-0.15
**Colombia**	**Leukemia**	Deaths	1.37	2.19	0.6	5.32	4.39	-0.61

Countries	Causes	Measure	Cases in 1990 No.×10^3	Cases in 2019 No.×10^3	Change in absolute number (100%)	ASR in 1990 per 100000	ASR in 2019 per 100000	EAPC from 1990 and 2019
**Colombia**	**Multiple myeloma**	Incidence	0.23	0.94	3.02	1.33	1.78	0.97
**Colombia**	**Multiple myeloma**	Deaths	0.2	0.72	2.62	1.16	1.36	0.52
**Colombia**	**Non-Hodgkin lymphoma**	Incidence	0.79	2.52	2.17	3.38	4.94	1.5
**Colombia**	**Non-Hodgkin lymphoma**	Deaths	0.65	1.51	1.34	3.11	2.91	-0.04
**Colombia**	**Hodgkin lymphoma**	Incidence	0.26	0.51	0.97	0.96	1.01	0.1
**Colombia**	**Hodgkin lymphoma**	Deaths	0.16	0.18	0.1	0.68	0.35	-2.36
**Comoros**	**Leukemia**	Incidence	0.02	0.03	0.14	4.51	4.33	-0.06
**Comoros**	**Leukemia**	Deaths	0.01	0.02	0.42	3.12	3.11	-0.04
**Comoros**	**Multiple myeloma**	Incidence	0	0.01	1.45	1.11	1.24	0.28
**Comoros**	**Multiple myeloma**	Deaths	0	0.01	1.36	1.11	1.2	0.16
**Comoros**	**Non-Hodgkin lymphoma**	Incidence	0.01	0.01	1.35	2.44	2.77	0.33
**Comoros**	**Non-Hodgkin lymphoma**	Deaths	0.01	0.02	1.23	2.83	3.1	0.25
**Comoros**	**Hodgkin lymphoma**	Incidence	0	0	0.89	0.73	0.74	-0.36
**Comoros**	**Hodgkin lymphoma**	Deaths	0	0	0.77	0.65	0.6	-0.63
**Congo**	**Leukemia**	Incidence	0.12	0.16	0.33	5.51	4.04	-1.1
**Congo**	**Leukemia**	Deaths	0.07	0.11	0.53	4.24	3.27	-0.96
**Congo**	**Multiple myeloma**	Incidence	0.02	0.04	1.29	1.53	1.45	-0.2
**Congo**	**Multiple myeloma**	Deaths	0.02	0.03	1.19	1.5	1.39	-0.31
**Congo**	**Non-Hodgkin lymphoma**	Incidence	0.04	0.1	1.34	3.35	3.2	-0.16
**Congo**	**Non-Hodgkin lymphoma**	Deaths	0.05	0.1	1.11	3.72	3.43	-0.31
**Congo**	**Hodgkin lymphoma**	Incidence	0.01	0.02	0.8	0.57	0.42	-1.22

Countries	Causes	Measure	Cases in 1990 No.×10^3	Cases in 2019 No.×10^3	Change in absolute number (100%)	ASR in 1990 per 100000	ASR in 2019 per 100000	EAPC from 1990 and 2019
**Congo**	**Hodgkin lymphoma**	Deaths	0.01	0.01	0.7	0.52	0.37	-1.43
**Cook Islands**	**Leukemia**	Incidence	0	0	-0.01	4.36	3.11	-1.19
**Cook Islands**	**Leukemia**	Deaths	0	0	0.13	3.33	2.34	-1.21
**Cook Islands**	**Multiple myeloma**	Incidence	0	0	0.93	0.84	0.84	-0.02
**Cook Islands**	**Multiple myeloma**	Deaths	0	0	0.88	0.71	0.66	-0.2
**Cook Islands**	**Non-Hodgkin lymphoma**	Incidence	0	0	1.03	2.48	3.24	1.06
**Cook Islands**	**Non-Hodgkin lymphoma**	Deaths	0	0	0.73	1.93	1.93	0.16
**Cook Islands**	**Hodgkin lymphoma**	Incidence	0	0	0.47	0.44	0.52	0.95
**Cook Islands**	**Hodgkin lymphoma**	Deaths	0	0	0.02	0.2	0.14	-0.86
**Costa Rica**	**Leukemia**	Incidence	0.19	0.4	1.09	7.6	8.05	0.19
**Costa Rica**	**Leukemia**	Deaths	0.14	0.25	0.86	6.03	5.11	-0.67
**Costa Rica**	**Multiple myeloma**	Incidence	0.03	0.16	3.91	1.84	3.03	1.77
**Costa Rica**	**Multiple myeloma**	Deaths	0.03	0.12	3.58	1.53	2.33	1.47
**Costa Rica**	**Non-Hodgkin lymphoma**	Incidence	0.12	0.4	2.28	5.79	7.97	1.39
**Costa Rica**	**Non-Hodgkin lymphoma**	Deaths	0.08	0.23	1.76	4.26	4.46	0.35
**Costa Rica**	**Hodgkin lymphoma**	Incidence	0.05	0.12	1.37	1.94	2.46	0.86
**Costa Rica**	**Hodgkin lymphoma**	Deaths	0.02	0.04	0.59	0.94	0.69	-1.15
**Côte d'Ivoire**	**Leukemia**	Incidence	0.41	0.87	1.14	4.24	4.35	0.25
**Côte d'Ivoire**	**Leukemia**	Deaths	0.26	0.57	1.21	3.47	3.48	0.1
**Côte d'Ivoire**	**Multiple myeloma**	Incidence	0.03	0.09	1.81	0.82	0.88	0.12
**Côte d'Ivoire**	**Multiple myeloma**	Deaths	0.03	0.08	1.77	0.79	0.83	0.07

Countries	Causes	Measure	Cases in 1990 No.×10^3	Cases in 2019 No.×10^3	Change in absolute number (100%)	ASR in 1990 per 100000	ASR in 2019 per 100000	EAPC from 1990 and 2019
**Côte d'Ivoire**	**Non-Hodgkin lymphoma**	Incidence	0.2	0.48	1.42	3.06	2.98	-0.62
**Côte d'Ivoire**	**Non-Hodgkin lymphoma**	Deaths	0.19	0.43	1.27	3.23	3.04	-0.73
**Côte d'Ivoire**	**Hodgkin lymphoma**	Incidence	0.03	0.06	1.29	0.33	0.33	-0.14
**Côte d'Ivoire**	**Hodgkin lymphoma**	Deaths	0.02	0.05	1.25	0.3	0.28	-0.21
**Croatia**	**Leukemia**	Incidence	0.59	0.93	0.58	10.25	12.74	1.05
**Croatia**	**Leukemia**	Deaths	0.35	0.41	0.17	5.93	4.81	-0.59
**Croatia**	**Multiple myeloma**	Incidence	0.14	0.27	0.95	2.15	3.08	1.54
**Croatia**	**Multiple myeloma**	Deaths	0.11	0.2	0.91	1.66	2.2	1.28
**Croatia**	**Non-Hodgkin lymphoma**	Incidence	0.36	0.69	0.94	6	8.74	1.57
**Croatia**	**Non-Hodgkin lymphoma**	Deaths	0.2	0.32	0.58	3.33	3.76	0.66
**Croatia**	**Hodgkin lymphoma**	Incidence	0.16	0.16	0.02	2.9	2.92	0.42
**Croatia**	**Hodgkin lymphoma**	Deaths	0.04	0.02	-0.5	0.67	0.28	-2.72
**Cuba**	**Leukemia**	Incidence	0.74	0.99	0.34	7.06	6.47	-0.38
**Cuba**	**Leukemia**	Deaths	0.55	0.66	0.19	5.25	4	-0.98
**Cuba**	**Multiple myeloma**	Incidence	0.26	0.59	1.25	2.55	3.12	0.59
**Cuba**	**Multiple myeloma**	Deaths	0.2	0.4	0.99	1.93	2.07	0.14
**Cuba**	**Non-Hodgkin lymphoma**	Incidence	0.75	1.1	0.46	7.04	6.76	-0.04
**Cuba**	**Non-Hodgkin lymphoma**	Deaths	0.5	0.63	0.25	4.73	3.58	-0.84
**Cuba**	**Hodgkin lymphoma**	Incidence	0.05	0.31	5.61	0.41	2.32	5.86
**Cuba**	**Hodgkin lymphoma**	Deaths	0.02	0.1	3.81	0.19	0.64	3.96
**Cyprus**	**Leukemia**	Incidence	0.06	0.26	3.15	8.25	15.82	3.01

Countries	Causes	Measure	Cases in 1990 No.×10^3	Cases in 2019 No.×10^3	Change in absolute number (100%)	ASR in 1990 per 100000	ASR in 2019 per 100000	EAPC from 1990 and 2019
**Cyprus**	**Leukemia**	Deaths	0.04	0.1	1.26	5.95	5.61	0.05
**Cyprus**	**Multiple myeloma**	Incidence	0.02	0.07	1.73	2.97	3.32	0.45
**Cyprus**	**Multiple myeloma**	Deaths	0.02	0.04	1.3	2.51	2.29	-0.37
**Cyprus**	**Non-Hodgkin lymphoma**	Incidence	0.07	0.19	1.62	9.3	10.37	0.09
**Cyprus**	**Non-Hodgkin lymphoma**	Deaths	0.05	0.08	0.68	6.39	4.36	-1.79
**Cyprus**	**Hodgkin lymphoma**	Incidence	0.01	0.03	2.03	1.32	2.2	1.74
**Cyprus**	**Hodgkin lymphoma**	Deaths	0	0	0.04	0.56	0.27	-3.13
**Czechia**	**Leukemia**	Incidence	1.23	2.11	0.71	9.88	11.39	0.34
**Czechia**	**Leukemia**	Deaths	0.91	1.04	0.14	7.05	5.07	-1.18
**Czechia**	**Multiple myeloma**	Incidence	0.37	0.62	0.69	2.63	2.92	0.18
**Czechia**	**Multiple myeloma**	Deaths	0.31	0.49	0.57	2.24	2.26	-0.17
**Czechia**	**Non-Hodgkin lymphoma**	Incidence	0.84	1.46	0.74	6.49	7.42	0.41
**Czechia**	**Non-Hodgkin lymphoma**	Deaths	0.53	0.69	0.3	3.95	3.31	-0.55
**Czechia**	**Hodgkin lymphoma**	Incidence	0.36	0.37	0.03	3.19	3.14	-0.36
**Czechia**	**Hodgkin lymphoma**	Deaths	0.14	0.06	-0.53	1.09	0.38	-3.68
**Democratic People's Republic of Korea**	**Leukemia**	Incidence	3.45	2.04	-0.41	14.47	7.6	-1.93
**Democratic People's Republic of Korea**	**Leukemia**	Deaths	1.31	1.31	0	6.4	4.49	-1.09
**Democratic People's Republic of Korea**	**Multiple myeloma**	Incidence	0.14	0.26	0.76	0.87	0.78	-0.32

Countries	Causes	Measure	Cases in 1990 No.×10^3	Cases in 2019 No.×10^3	Change in absolute number (100%)	ASR in 1990 per 100000	ASR in 2019 per 100000	EAPC from 1990 and 2019
**Democratic People's Republic of Korea**	**Multiple myeloma**	Deaths	0.12	0.21	0.78	0.74	0.66	-0.4
**Democratic People's Republic of Korea**	**Non-Hodgkin lymphoma**	Incidence	0.47	0.79	0.69	2.56	2.58	0.18
**Democratic People's Republic of Korea**	**Non-Hodgkin lymphoma**	Deaths	0.38	0.64	0.7	2.12	2.02	-0.02
**Democratic People's Republic of Korea**	**Hodgkin lymphoma**	Incidence	0.1	0.13	0.26	0.54	0.43	-0.83
**Democratic People's Republic of Korea**	**Hodgkin lymphoma**	Deaths	0.05	0.07	0.32	0.29	0.22	-1.01
**Democratic Republic of the Congo**	**Leukemia**	Incidence	2.23	2.49	0.11	5.23	3.61	-1.18
**Democratic Republic of the Congo**	**Leukemia**	Deaths	1.09	1.5	0.37	3.62	2.81	-0.83
**Democratic Republic of the Congo**	**Multiple myeloma**	Incidence	0.18	0.4	1.26	1.12	1.13	-0.01
**Democratic Republic of the Congo**	**Multiple myeloma**	Deaths	0.16	0.36	1.21	1.11	1.09	-0.09
**Democratic Republic of the Congo**	**Non-Hodgkin lymphoma**	Incidence	0.41	0.94	1.31	2.05	2	-0.09
**Democratic Republic of the Congo**	**Non-Hodgkin lymphoma**	Deaths	0.48	0.92	0.92	2.34	2.16	-0.31
**Democratic Republic of the Congo**	**Hodgkin lymphoma**	Incidence	0.12	0.26	1.22	0.49	0.47	-0.1

Countries	Causes	Measure	Cases in 1990 No.×10^3	Cases in 2019 No.×10^3	Change in absolute number (100%)	ASR in 1990 per 100000	ASR in 2019 per 100000	EAPC from 1990 and 2019
**Democratic Republic of the Congo**	**Hodgkin lymphoma**	Deaths	0.1	0.21	1.15	0.46	0.42	-0.19
**Denmark**	**Leukemia**	Incidence	0.9	1.24	0.38	13.55	13.16	0.12
**Denmark**	**Leukemia**	Deaths	0.61	0.58	-0.05	8.07	5.06	-1.42
**Denmark**	**Multiple myeloma**	Incidence	0.21	0.51	1.42	2.58	4.37	1.42
**Denmark**	**Multiple myeloma**	Deaths	0.16	0.33	1.08	1.92	2.74	0.68
**Denmark**	**Non-Hodgkin lymphoma**	Incidence	0.5	1.01	1.03	6.58	9.2	0.53
**Denmark**	**Non-Hodgkin lymphoma**	Deaths	0.28	0.44	0.58	3.48	3.77	-0.44
**Denmark**	**Hodgkin lymphoma**	Incidence	0.09	0.17	1.01	1.46	2.6	1.88
**Denmark**	**Hodgkin lymphoma**	Deaths	0.03	0.03	0.04	0.41	0.32	-1.21
**Djibouti**	**Leukemia**	Incidence	0.03	0.06	1.25	4.68	5.38	0.51
**Djibouti**	**Leukemia**	Deaths	0.01	0.03	1.69	3.12	3.66	0.57
**Djibouti**	**Multiple myeloma**	Incidence	0	0.01	4.05	1.12	1.33	0.6
**Djibouti**	**Multiple myeloma**	Deaths	0	0.01	3.94	1.1	1.28	0.5
**Djibouti**	**Non-Hodgkin lymphoma**	Incidence	0	0.02	3.7	2.72	3.46	0.88
**Djibouti**	**Non-Hodgkin lymphoma**	Deaths	0.01	0.02	3.41	3.13	3.88	0.79
**Djibouti**	**Hodgkin lymphoma**	Incidence	0	0.01	2.18	0.71	0.76	0.06
**Djibouti**	**Hodgkin lymphoma**	Deaths	0	0.01	2	0.62	0.59	-0.27
**Dominica**	**Leukemia**	Incidence	0.01	0.01	0.09	7.98	8.55	0.36
**Dominica**	**Leukemia**	Deaths	0	0.01	0.12	6.26	6.39	0.17
**Dominica**	**Multiple myeloma**	Incidence	0	0.01	0.42	6.39	7.25	0.54
**Dominica**	**Multiple myeloma**	Deaths	0	0.01	0.39	5.26	5.89	0.5

Countries	Causes	Measure	Cases in 1990 No.×10³	Cases in 2019 No.×10³	Change in absolute number (100%)	ASR in 1990 per 100000	ASR in 2019 per 100000	EAPC from 1990 and 2019
**Dominica**	**Non-Hodgkin lymphoma**	Incidence	0.01	0.01	0.2	8.53	9.11	0.38
**Dominica**	**Non-Hodgkin lymphoma**	Deaths	0.01	0.01	0.15	7.49	7.25	-0.01
**Dominica**	**Hodgkin lymphoma**	Incidence	0	0	0.03	0.94	0.9	-0.33
**Dominica**	**Hodgkin lymphoma**	Deaths	0	0	-0.01	0.62	0.54	-0.71
**Dominican Republic**	**Leukemia**	Incidence	0.43	0.59	0.36	5.93	5.79	0.16
**Dominican Republic**	**Leukemia**	Deaths	0.21	0.4	0.87	3.64	4.05	0.73
**Dominican Republic**	**Multiple myeloma**	Incidence	0.04	0.18	3.38	1.1	1.93	2.21
**Dominican Republic**	**Multiple myeloma**	Deaths	0.04	0.14	2.99	0.97	1.53	1.89
**Dominican Republic**	**Non-Hodgkin lymphoma**	Incidence	0.09	0.34	2.65	1.75	3.38	2.61
**Dominican Republic**	**Non-Hodgkin lymphoma**	Deaths	0.08	0.25	2.06	1.73	2.6	1.86
**Dominican Republic**	**Hodgkin lymphoma**	Incidence	0.01	0.02	1.18	0.17	0.21	0.72
**Dominican Republic**	**Hodgkin lymphoma**	Deaths	0.01	0.01	0.69	0.14	0.12	-0.24
**Ecuador**	**Leukemia**	Incidence	0.62	1.29	1.08	6.81	7.81	0.62
**Ecuador**	**Leukemia**	Deaths	0.41	0.92	1.27	5.16	5.75	0.64
**Ecuador**	**Multiple myeloma**	Incidence	0.06	0.25	3.39	1.08	1.66	1.96
**Ecuador**	**Multiple myeloma**	Deaths	0.05	0.21	2.95	1.01	1.39	1.6
**Ecuador**	**Non-Hodgkin lymphoma**	Incidence	0.17	1	4.79	2.53	6.34	3.7
**Ecuador**	**Non-Hodgkin lymphoma**	Deaths	0.16	0.72	3.41	2.65	4.79	2.49
**Ecuador**	**Hodgkin lymphoma**	Incidence	0.07	0.14	1.17	0.82	0.86	0.53
**Ecuador**	**Hodgkin lymphoma**	Deaths	0.05	0.07	0.45	0.67	0.45	-0.97
**Egypt**	**Leukemia**	Incidence	2.87	4.07	0.42	5.62	4.98	-0.26

Countries	Causes	Measure	Cases in 1990 No.×10^3	Cases in 2019 No.×10^3	Change in absolute number (100%)	ASR in 1990 per 100000	ASR in 2019 per 100000	EAPC from 1990 and 2019
**Egypt**	**Leukemia**	Deaths	1.78	2.81	0.57	4.18	3.78	-0.19
**Egypt**	**Multiple myeloma**	Incidence	0.29	0.84	1.95	1.01	1.36	1.1
**Egypt**	**Multiple myeloma**	Deaths	0.26	0.7	1.69	0.96	1.2	0.86
**Egypt**	**Non-Hodgkin lymphoma**	Incidence	0.7	2.01	1.88	1.64	2.49	1.45
**Egypt**	**Non-Hodgkin lymphoma**	Deaths	0.6	1.32	1.21	1.57	1.81	0.62
**Egypt**	**Hodgkin lymphoma**	Incidence	0.17	0.35	1.03	0.42	0.41	0
**Egypt**	**Hodgkin lymphoma**	Deaths	0.13	0.18	0.34	0.35	0.24	-1.16
**El Salvador**	**Leukemia**	Incidence	0.48	0.45	-0.06	8.64	7.3	-0.67
**El Salvador**	**Leukemia**	Deaths	0.23	0.3	0.31	5.02	4.84	-0.13
**El Salvador**	**Multiple myeloma**	Incidence	0.02	0.05	1.94	0.59	0.88	1.35
**El Salvador**	**Multiple myeloma**	Deaths	0.02	0.04	1.66	0.53	0.69	0.92
**El Salvador**	**Non-Hodgkin lymphoma**	Incidence	0.06	0.22	2.77	1.55	3.64	2.75
**El Salvador**	**Non-Hodgkin lymphoma**	Deaths	0.05	0.16	1.85	1.57	2.56	1.33
**El Salvador**	**Hodgkin lymphoma**	Incidence	0.05	0.06	0.27	1.17	0.96	-0.97
**El Salvador**	**Hodgkin lymphoma**	Deaths	0.04	0.03	-0.16	0.97	0.49	-2.74
**Equatorial Guinea**	**Leukemia**	Incidence	0.02	0.03	0.45	5	3.65	-1.07
**Equatorial Guinea**	**Leukemia**	Deaths	0.01	0.02	0.64	3.7	2.92	-0.77
**Equatorial Guinea**	**Multiple myeloma**	Incidence	0	0.01	1.95	1.14	1.45	0.97
**Equatorial Guinea**	**Multiple myeloma**	Deaths	0	0.01	1.72	1.14	1.35	0.72
**Equatorial Guinea**	**Non-Hodgkin lymphoma**	Incidence	0.01	0.03	4.47	2.15	3.96	2.9
**Equatorial Guinea**	**Non-Hodgkin lymphoma**	Deaths	0.01	0.02	3	2.41	4.07	2.55

Countries	Causes	Measure	Cases in 1990 No.×10^3	Cases in 2019 No.×10^3	Change in absolute number (100%)	ASR in 1990 per 100000	ASR in 2019 per 100000	EAPC from 1990 and 2019
**Equatorial Guinea**	**Hodgkin lymphoma**	Incidence	0	0	0.86	0.6	0.33	-2.42
**Equatorial Guinea**	**Hodgkin lymphoma**	Deaths	0	0	0.41	0.56	0.26	-3.01
**Eritrea**	**Leukemia**	Incidence	0.14	0.27	0.9	4.45	4.88	0.39
**Eritrea**	**Leukemia**	Deaths	0.07	0.16	1.11	3.15	3.59	0.46
**Eritrea**	**Multiple myeloma**	Incidence	0.01	0.03	2.31	1.07	1.33	0.59
**Eritrea**	**Multiple myeloma**	Deaths	0.01	0.03	2.2	1.07	1.29	0.48
**Eritrea**	**Non-Hodgkin lymphoma**	Incidence	0.03	0.1	2.86	2.09	3.07	1.13
**Eritrea**	**Non-Hodgkin lymphoma**	Deaths	0.03	0.1	2.44	2.4	3.47	1.08
**Eritrea**	**Hodgkin lymphoma**	Incidence	0.02	0.05	1.58	0.9	0.91	-0.02
**Eritrea**	**Hodgkin lymphoma**	Deaths	0.01	0.04	1.47	0.8	0.77	-0.16
**Estonia**	**Leukemia**	Incidence	0.19	0.25	0.31	11.41	12.08	0.71
**Estonia**	**Leukemia**	Deaths	0.14	0.14	0.02	7.4	5.62	-0.85
**Estonia**	**Multiple myeloma**	Incidence	0.03	0.09	1.73	1.65	3.54	3.1
**Estonia**	**Multiple myeloma**	Deaths	0.03	0.06	1.35	1.26	2.24	2.33
**Estonia**	**Non-Hodgkin lymphoma**	Incidence	0.05	0.18	2.29	3.04	7.75	3.49
**Estonia**	**Non-Hodgkin lymphoma**	Deaths	0.04	0.09	1.5	1.89	3.54	2
**Estonia**	**Hodgkin lymphoma**	Incidence	0.04	0.03	-0.16	2.19	2.2	0.16
**Estonia**	**Hodgkin lymphoma**	Deaths	0.02	0.01	-0.6	0.87	0.31	-4.28
**Eswatini**	**Leukemia**	Incidence	0.02	0.04	0.73	4.46	5.11	0.71
**Eswatini**	**Leukemia**	Deaths	0.02	0.03	0.86	3.71	4.11	0.58
**Eswatini**	**Multiple myeloma**	Incidence	0.01	0.01	1.36	1.95	2.32	0.48

Countries	Causes	Measure	Cases in 1990 No.×10^3	Cases in 2019 No.×10^3	Change in absolute number (100%)	ASR in 1990 per 100000	ASR in 2019 per 100000	EAPC from 1990 and 2019
**Eswatini**	**Multiple myeloma**	Deaths	0.01	0.01	1.31	1.86	2.17	0.43
**Eswatini**	**Non-Hodgkin lymphoma**	Incidence	0.01	0.03	1.73	2.93	4.12	1.29
**Eswatini**	**Non-Hodgkin lymphoma**	Deaths	0.01	0.03	1.53	3.14	4.17	1.1
**Eswatini**	**Hodgkin lymphoma**	Incidence	0	0	1.07	0.4	0.46	0.69
**Eswatini**	**Hodgkin lymphoma**	Deaths	0	0	0.93	0.35	0.37	0.46
**Ethiopia**	**Leukemia**	Incidence	10.58	8.31	-0.21	17.34	9.5	-2.15
**Ethiopia**	**Leukemia**	Deaths	6.22	5.27	-0.15	13.18	7.74	-1.98
**Ethiopia**	**Multiple myeloma**	Incidence	0.2	0.35	0.81	0.99	0.89	-0.46
**Ethiopia**	**Multiple myeloma**	Deaths	0.19	0.33	0.78	0.98	0.87	-0.52
**Ethiopia**	**Non-Hodgkin lymphoma**	Incidence	0.27	0.48	0.77	1.11	0.95	-0.72
**Ethiopia**	**Non-Hodgkin lymphoma**	Deaths	0.3	0.49	0.62	1.23	1.05	-0.72
**Ethiopia**	**Hodgkin lymphoma**	Incidence	0.52	0.65	0.25	1.42	0.81	-2.18
**Ethiopia**	**Hodgkin lymphoma**	Deaths	0.42	0.47	0.12	1.27	0.68	-2.39
**Fiji**	**Leukemia**	Incidence	0.06	0.07	0.21	9.51	8.53	-0.7
**Fiji**	**Leukemia**	Deaths	0.04	0.05	0.24	7.7	6.47	-0.88
**Fiji**	**Multiple myeloma**	Incidence	0	0.01	1.23	1.03	1.1	0.3
**Fiji**	**Multiple myeloma**	Deaths	0	0.01	1.21	0.96	1.01	0.23
**Fiji**	**Non-Hodgkin lymphoma**	Incidence	0.01	0.02	1.27	1.81	2.55	1.47
**Fiji**	**Non-Hodgkin lymphoma**	Deaths	0.01	0.02	1.31	1.84	2.44	1.31
**Fiji**	**Hodgkin lymphoma**	Incidence	0	0	0.64	0.46	0.52	0.83
**Fiji**	**Hodgkin lymphoma**	Deaths	0	0	0.5	0.34	0.33	0.36

Countries	Causes	Measure	Cases in 1990 No.×10^3	Cases in 2019 No.×10^3	Change in absolute number (100%)	ASR in 1990 per 100000	ASR in 2019 per 100000	EAPC from 1990 and 2019
**Finland**	**Leukemia**	Incidence	0.56	0.97	0.73	9.11	10.63	0.73
**Finland**	**Leukemia**	Deaths	0.35	0.42	0.23	5.2	3.6	-1.26
**Finland**	**Multiple myeloma**	Incidence	0.27	0.53	1.01	3.7	4.3	0.47
**Finland**	**Multiple myeloma**	Deaths	0.19	0.36	0.89	2.63	2.76	0.02
**Finland**	**Non-Hodgkin lymphoma**	Incidence	0.7	1.34	0.92	10.29	11.41	0.21
**Finland**	**Non-Hodgkin lymphoma**	Deaths	0.39	0.57	0.48	5.51	4.55	-0.86
**Finland**	**Hodgkin lymphoma**	Incidence	0.13	0.2	0.56	2.25	3.03	1.45
**Finland**	**Hodgkin lymphoma**	Deaths	0.04	0.03	-0.28	0.59	0.29	-2.18
**France**	**Leukemia**	Incidence	7.93	17.6	1.22	11.23	16.08	1.23
**France**	**Leukemia**	Deaths	5.12	7.7	0.5	6.49	5.32	-0.75
**France**	**Multiple myeloma**	Incidence	2.77	5.36	0.94	3.31	3.83	0.34
**France**	**Multiple myeloma**	Deaths	2.23	4.05	0.82	2.58	2.66	-0.09
**France**	**Non-Hodgkin lymphoma**	Incidence	7.25	13.54	0.87	9.27	10.04	-0.14
**France**	**Non-Hodgkin lymphoma**	Deaths	4.11	5.97	0.45	5.04	4.09	-1.17
**France**	**Hodgkin lymphoma**	Incidence	1.34	2.04	0.52	2.1	2.81	1.24
**France**	**Hodgkin lymphoma**	Deaths	0.41	0.31	-0.24	0.56	0.29	-2.2
**Gabon**	**Leukemia**	Incidence	0.05	0.06	0.16	5.23	4.2	-0.71
**Gabon**	**Leukemia**	Deaths	0.03	0.04	0.35	3.93	3.33	-0.59
**Gabon**	**Multiple myeloma**	Incidence	0.01	0.02	0.99	1.63	1.76	0.16
**Gabon**	**Multiple myeloma**	Deaths	0.01	0.02	0.89	1.56	1.64	0.04
**Gabon**	**Non-Hodgkin lymphoma**	Incidence	0.03	0.05	1.01	4	4.24	0.11

Countries	Causes	Measure	Cases in 1990 No.×10^3	Cases in 2019 No.×10^3	Change in absolute number (100%)	ASR in 1990 per 100000	ASR in 2019 per 100000	EAPC from 1990 and 2019
**Gabon**	**Non-Hodgkin lymphoma**	Deaths	0.03	0.05	0.79	4.36	4.35	-0.07
**Gabon**	**Hodgkin lymphoma**	Incidence	0	0.01	0.7	0.48	0.41	-0.57
**Gabon**	**Hodgkin lymphoma**	Deaths	0	0	0.48	0.43	0.33	-0.92
**Gambia**	**Leukemia**	Incidence	0.02	0.05	1.19	2.92	3.16	0.16
**Gambia**	**Leukemia**	Deaths	0.01	0.04	1.42	2.44	2.67	0.19
**Gambia**	**Multiple myeloma**	Incidence	0	0.01	2.56	0.57	0.75	0.81
**Gambia**	**Multiple myeloma**	Deaths	0	0.01	2.51	0.55	0.71	0.74
**Gambia**	**Non-Hodgkin lymphoma**	Incidence	0	0.01	2.27	0.66	0.83	0.57
**Gambia**	**Non-Hodgkin lymphoma**	Deaths	0	0.01	1.88	0.71	0.84	0.39
**Gambia**	**Hodgkin lymphoma**	Incidence	0	0.01	1.52	0.37	0.38	-0.08
**Gambia**	**Hodgkin lymphoma**	Deaths	0	0	1.37	0.32	0.31	-0.28
**Georgia**	**Leukemia**	Incidence	0.62	0.33	-0.46	11.5	7.64	-1.03
**Georgia**	**Leukemia**	Deaths	0.33	0.23	-0.3	5.91	4.82	-0.16
**Georgia**	**Multiple myeloma**	Incidence	0.04	0.06	0.26	0.7	0.97	1.58
**Georgia**	**Multiple myeloma**	Deaths	0.04	0.05	0.3	0.59	0.81	1.55
**Georgia**	**Non-Hodgkin lymphoma**	Incidence	0.09	0.22	1.34	1.57	4.42	4.7
**Georgia**	**Non-Hodgkin lymphoma**	Deaths	0.07	0.15	1.26	1.11	2.82	4.54
**Georgia**	**Hodgkin lymphoma**	Incidence	0.18	0.09	-0.5	3.03	2.12	-2.23
**Georgia**	**Hodgkin lymphoma**	Deaths	0.1	0.05	-0.53	1.67	0.96	-2.48
**Germany**	**Leukemia**	Incidence	14.92	32.36	1.17	14.57	21.91	0.71
**Germany**	**Leukemia**	Deaths	6.3	9.34	0.48	5.45	4.88	-0.31

Countries	Causes	Measure	Cases in 1990 No.×10^3	Cases in 2019 No.×10^3	Change in absolute number (100%)	ASR in 1990 per 100000	ASR in 2019 per 100000	EAPC from 1990 and 2019
**Germany**	**Multiple myeloma**	Incidence	2.92	8	1.73	2.33	4.16	1.46
**Germany**	**Multiple myeloma**	Deaths	1.91	5.09	1.67	1.49	2.47	1.24
**Germany**	**Non-Hodgkin lymphoma**	Incidence	9.07	17.64	0.94	7.76	9.51	0.26
**Germany**	**Non-Hodgkin lymphoma**	Deaths	5.05	7.89	0.56	4.11	3.95	-0.46
**Germany**	**Hodgkin lymphoma**	Incidence	3.03	2.45	-0.19	3.32	2.58	-1.16
**Germany**	**Hodgkin lymphoma**	Deaths	0.8	0.39	-0.52	0.72	0.24	-3.65
**Ghana**	**Leukemia**	Incidence	0.56	0.85	0.51	4.56	3.58	-1.18
**Ghana**	**Leukemia**	Deaths	0.4	0.62	0.55	3.75	2.91	-1.19
**Ghana**	**Multiple myeloma**	Incidence	0.05	0.15	2.11	0.78	0.94	0.58
**Ghana**	**Multiple myeloma**	Deaths	0.05	0.14	1.99	0.74	0.87	0.49
**Ghana**	**Non-Hodgkin lymphoma**	Incidence	0.67	1.14	0.7	5.86	5.08	-1.22
**Ghana**	**Non-Hodgkin lymphoma**	Deaths	0.54	0.95	0.74	5.55	4.86	-0.96
**Ghana**	**Hodgkin lymphoma**	Incidence	0.02	0.04	1.2	0.18	0.16	-0.4
**Ghana**	**Hodgkin lymphoma**	Deaths	0.01	0.03	0.95	0.16	0.13	-0.69
**Greece**	**Leukemia**	Incidence	1.66	3.27	0.97	12.91	17.73	0.92
**Greece**	**Leukemia**	Deaths	0.99	1.63	0.64	6.99	6.59	-0.55
**Greece**	**Multiple myeloma**	Incidence	0.35	0.77	1.19	2.29	3.23	1.29
**Greece**	**Multiple myeloma**	Deaths	0.26	0.59	1.22	1.72	2.23	1.02
**Greece**	**Non-Hodgkin lymphoma**	Incidence	0.71	1.23	0.72	5.14	5.96	0.23
**Greece**	**Non-Hodgkin lymphoma**	Deaths	0.35	0.55	0.56	2.43	2.38	-0.38
**Greece**	**Hodgkin lymphoma**	Incidence	0.58	0.86	0.47	4.88	6.41	0.68

Countries	Causes	Measure	Cases in 1990 No.×10³	Cases in 2019 No.×10³	Change in absolute number (100%)	ASR in 1990 per 100000	ASR in 2019 per 100000	EAPC from 1990 and 2019
**Greece**	**Hodgkin lymphoma**	Deaths	0.18	0.19	0.08	1.26	0.94	-1.3
**Greenland**	**Leukemia**	Incidence	0	0	0.39	5.54	4.43	-1.16
**Greenland**	**Leukemia**	Deaths	0	0	0.25	4.62	3.2	-1.62
**Greenland**	**Multiple myeloma**	Incidence	0	0	1.26	2.56	2.81	-0.07
**Greenland**	**Multiple myeloma**	Deaths	0	0	1.1	2.2	2.21	-0.42
**Greenland**	**Non-Hodgkin lymphoma**	Incidence	0	0	0.57	6.89	6.42	-0.74
**Greenland**	**Non-Hodgkin lymphoma**	Deaths	0	0	0.4	6.18	4.7	-1.42
**Greenland**	**Hodgkin lymphoma**	Incidence	0	0	-0.07	0.85	0.67	-1.22
**Greenland**	**Hodgkin lymphoma**	Deaths	0	0	-0.24	0.55	0.31	-2.28
**Grenada**	**Leukemia**	Incidence	0.01	0.01	-0.01	7.59	5.99	-0.44
**Grenada**	**Leukemia**	Deaths	0	0	0.03	5.49	4.28	-0.59
**Grenada**	**Multiple myeloma**	Incidence	0	0	0.75	3.25	3.67	0.58
**Grenada**	**Multiple myeloma**	Deaths	0	0	0.53	2.77	2.84	0.29
**Grenada**	**Non-Hodgkin lymphoma**	Incidence	0.01	0.01	0.33	12.46	11.21	-0.34
**Grenada**	**Non-Hodgkin lymphoma**	Deaths	0.01	0.01	0.07	11.84	8.37	-1.06
**Grenada**	**Hodgkin lymphoma**	Incidence	0	0	0.44	0.57	0.56	-0.09
**Grenada**	**Hodgkin lymphoma**	Deaths	0	0	0.09	0.42	0.3	-0.96
**Guam**	**Leukemia**	Incidence	0.01	0.01	0.39	6.13	5.01	-0.85
**Guam**	**Leukemia**	Deaths	0	0.01	0.48	4.78	3.34	-1.42
**Guam**	**Multiple myeloma**	Incidence	0	0	1.03	1.51	1.19	-1.1
**Guam**	**Multiple myeloma**	Deaths	0	0	1.07	1.3	0.98	-1.21

Countries	Causes	Measure	Cases in 1990 No.×10^3	Cases in 2019 No.×10^3	Change in absolute number (100%)	ASR in 1990 per 100000	ASR in 2019 per 100000	EAPC from 1990 and 2019
**Guam**	**Non-Hodgkin lymphoma**	Incidence	0.01	0.01	0.78	5.41	5.06	-0.44
**Guam**	**Non-Hodgkin lymphoma**	Deaths	0	0.01	0.81	4.19	3.4	-0.77
**Guam**	**Hodgkin lymphoma**	Incidence	0	0	0.41	0.35	0.33	-0.44
**Guam**	**Hodgkin lymphoma**	Deaths	0	0	0.43	0.15	0.12	-0.62
**Guatemala**	**Leukemia**	Incidence	0.48	1.12	1.34	5.87	6.91	0.43
**Guatemala**	**Leukemia**	Deaths	0.24	0.72	1.99	3.87	4.89	0.68
**Guatemala**	**Multiple myeloma**	Incidence	0.02	0.09	2.89	0.6	0.78	0.85
**Guatemala**	**Multiple myeloma**	Deaths	0.02	0.07	2.64	0.57	0.67	0.53
**Guatemala**	**Non-Hodgkin lymphoma**	Incidence	0.07	0.3	3.39	1.37	2.23	2.2
**Guatemala**	**Non-Hodgkin lymphoma**	Deaths	0.06	0.24	2.78	1.43	1.99	1.55
**Guatemala**	**Hodgkin lymphoma**	Incidence	0.05	0.08	0.79	0.82	0.55	-1.26
**Guatemala**	**Hodgkin lymphoma**	Deaths	0.04	0.05	0.39	0.74	0.38	-2.26
**Guinea**	**Leukemia**	Incidence	0.18	0.32	0.72	3.3	3.33	0.23
**Guinea**	**Leukemia**	Deaths	0.13	0.23	0.74	2.77	2.8	0.2
**Guinea**	**Multiple myeloma**	Incidence	0.02	0.04	0.88	0.67	0.77	0.62
**Guinea**	**Multiple myeloma**	Deaths	0.02	0.04	0.83	0.65	0.74	0.55
**Guinea**	**Non-Hodgkin lymphoma**	Incidence	0.03	0.07	1.34	0.69	0.81	0.64
**Guinea**	**Non-Hodgkin lymphoma**	Deaths	0.03	0.06	0.95	0.74	0.82	0.44
**Guinea**	**Hodgkin lymphoma**	Incidence	0.05	0.09	0.99	0.9	0.9	0.14
**Guinea**	**Hodgkin lymphoma**	Deaths	0.04	0.07	0.86	0.79	0.76	0.01
**Guinea-Bissau**	**Leukemia**	Incidence	0.05	0.07	0.43	5.52	5.02	-0.03

Countries	Causes	Measure	Cases in 1990 No.×10^3	Cases in 2019 No.×10^3	Change in absolute number (100%)	ASR in 1990 per 100000	ASR in 2019 per 100000	EAPC from 1990 and 2019
**Guinea-Bissau**	**Leukemia**	Deaths	0.03	0.05	0.49	4.45	4.09	-0.06
**Guinea-Bissau**	**Multiple myeloma**	Incidence	0	0.01	0.85	0.98	1.02	0.26
**Guinea-Bissau**	**Multiple myeloma**	Deaths	0	0.01	0.79	0.93	0.95	0.18
**Guinea-Bissau**	**Non-Hodgkin lymphoma**	Incidence	0.02	0.03	0.79	3.27	3.11	-0.47
**Guinea-Bissau**	**Non-Hodgkin lymphoma**	Deaths	0.02	0.03	0.57	3.48	3.18	-0.62
**Guinea-Bissau**	**Hodgkin lymphoma**	Incidence	0	0.01	0.77	0.48	0.45	-0.05
**Guinea-Bissau**	**Hodgkin lymphoma**	Deaths	0	0	0.72	0.43	0.4	-0.08
**Guyana**	**Leukemia**	Incidence	0.03	0.03	0.09	5.03	4.9	0.17
**Guyana**	**Leukemia**	Deaths	0.02	0.03	0.19	4.01	3.87	0.06
**Guyana**	**Multiple myeloma**	Incidence	0	0.01	1.86	0.87	1.47	1.66
**Guyana**	**Multiple myeloma**	Deaths	0	0.01	1.63	0.76	1.19	1.44
**Guyana**	**Non-Hodgkin lymphoma**	Incidence	0.02	0.02	0.42	3.01	3.23	0.33
**Guyana**	**Non-Hodgkin lymphoma**	Deaths	0.01	0.02	0.35	2.99	2.85	-0.02
**Guyana**	**Hodgkin lymphoma**	Incidence	0	0.01	1.48	0.36	0.69	1.71
**Guyana**	**Hodgkin lymphoma**	Deaths	0	0	1.12	0.29	0.46	1.15
**Haiti**	**Leukemia**	Incidence	0.95	1.09	0.14	13.15	9.4	-0.87
**Haiti**	**Leukemia**	Deaths	0.49	0.64	0.3	8.37	6.4	-0.69
**Haiti**	**Multiple myeloma**	Incidence	0.1	0.21	1.15	2.99	3.02	0.16
**Haiti**	**Multiple myeloma**	Deaths	0.09	0.18	1.05	2.83	2.72	-0.01
**Haiti**	**Non-Hodgkin lymphoma**	Incidence	0.17	0.31	0.81	3.96	3.44	-0.3
**Haiti**	**Non-Hodgkin lymphoma**	Deaths	0.2	0.31	0.56	4.4	3.59	-0.5

Countries	Causes	Measure	Cases in 1990 No.×10³	Cases in 2019 No.×10³	Change in absolute number (100%)	ASR in 1990 per 100000	ASR in 2019 per 100000	EAPC from 1990 and 2019
**Haiti**	**Hodgkin lymphoma**	Incidence	0.04	0.08	0.96	0.88	0.77	-0.38
**Haiti**	**Hodgkin lymphoma**	Deaths	0.03	0.06	0.83	0.82	0.67	-0.62
**Honduras**	**Leukemia**	Incidence	0.46	0.61	0.31	9.35	7.83	-0.58
**Honduras**	**Leukemia**	Deaths	0.26	0.44	0.7	6.48	6.28	0
**Honduras**	**Multiple myeloma**	Incidence	0.02	0.11	3.81	1.09	1.81	2.06
**Honduras**	**Multiple myeloma**	Deaths	0.02	0.09	3.57	1	1.58	1.93
**Honduras**	**Non-Hodgkin lymphoma**	Incidence	0.05	0.16	2.54	1.58	2.4	1.58
**Honduras**	**Non-Hodgkin lymphoma**	Deaths	0.04	0.15	2.39	1.65	2.34	1.45
**Honduras**	**Hodgkin lymphoma**	Incidence	0.01	0.02	1.17	0.24	0.22	-0.26
**Honduras**	**Hodgkin lymphoma**	Deaths	0.01	0.01	0.93	0.21	0.17	-0.57
**Hungary**	**Leukemia**	Incidence	1.2	1.72	0.43	9.23	10.52	0.29
**Hungary**	**Leukemia**	Deaths	0.92	0.95	0.03	6.85	5.27	-1.03
**Hungary**	**Multiple myeloma**	Incidence	0.29	0.4	0.39	1.95	2.11	-0.04
**Hungary**	**Multiple myeloma**	Deaths	0.24	0.34	0.39	1.63	1.73	-0.12
**Hungary**	**Non-Hodgkin lymphoma**	Incidence	0.73	1.18	0.61	5.44	6.76	0.32
**Hungary**	**Non-Hodgkin lymphoma**	Deaths	0.5	0.61	0.21	3.62	3.27	-0.74
**Hungary**	**Hodgkin lymphoma**	Incidence	0.25	0.16	-0.37	2.13	1.43	-1.94
**Hungary**	**Hodgkin lymphoma**	Deaths	0.12	0.04	-0.65	0.95	0.28	-4.69
**Iceland**	**Leukemia**	Incidence	0.03	0.06	0.85	11.5	12.4	-0.02
**Iceland**	**Leukemia**	Deaths	0.01	0.02	0.51	4.67	3.67	-0.81
**Iceland**	**Multiple myeloma**	Incidence	0.01	0.02	1.04	4.06	4.29	0

Countries	Causes	Measure	Cases in 1990 No.×10^3	Cases in 2019 No.×10^3	Change in absolute number (100%)	ASR in 1990 per 100000	ASR in 2019 per 100000	EAPC from 1990 and 2019
**Iceland**	**Multiple myeloma**	Deaths	0.01	0.02	0.85	2.89	2.71	-0.38
**Iceland**	**Non-Hodgkin lymphoma**	Incidence	0.02	0.04	0.94	8.12	8.53	-0.02
**Iceland**	**Non-Hodgkin lymphoma**	Deaths	0.01	0.02	0.61	3.66	3.08	-0.75
**Iceland**	**Hodgkin lymphoma**	Incidence	0.01	0.01	0.32	2.35	2.19	-0.07
**Iceland**	**Hodgkin lymphoma**	Deaths	0	0	-0.18	0.41	0.2	-2.4
**India**	**Leukemia**	Incidence	34.01	43.57	0.28	4.61	3.56	-1.22
**India**	**Leukemia**	Deaths	23.65	33.83	0.43	3.77	2.87	-1.27
**India**	**Multiple myeloma**	Incidence	3.35	10.35	2.09	0.75	0.91	0.61
**India**	**Multiple myeloma**	Deaths	3.05	9.06	1.97	0.73	0.82	0.37
**India**	**Non-Hodgkin lymphoma**	Incidence	9.23	32.46	2.52	1.61	2.66	1.72
**India**	**Non-Hodgkin lymphoma**	Deaths	8.38	27.26	2.25	1.65	2.38	1.29
**India**	**Hodgkin lymphoma**	Incidence	5.18	6.61	0.28	0.73	0.48	-1.66
**India**	**Hodgkin lymphoma**	Deaths	4.1	4.36	0.06	0.63	0.33	-2.37
**Indonesia**	**Leukemia**	Incidence	15.76	15.27	-0.03	8.89	6.71	-0.98
**Indonesia**	**Leukemia**	Deaths	8.31	10.59	0.27	5.66	4.93	-0.49
**Indonesia**	**Multiple myeloma**	Incidence	0.63	1.68	1.65	0.64	0.78	0.73
**Indonesia**	**Multiple myeloma**	Deaths	0.57	1.47	1.56	0.61	0.72	0.63
**Indonesia**	**Non-Hodgkin lymphoma**	Incidence	2.49	6.48	1.61	1.86	2.85	1.37
**Indonesia**	**Non-Hodgkin lymphoma**	Deaths	2.35	5.84	1.48	1.93	2.72	1.16
**Indonesia**	**Hodgkin lymphoma**	Incidence	0.62	0.82	0.33	0.41	0.33	-0.77
**Indonesia**	**Hodgkin lymphoma**	Deaths	0.46	0.52	0.14	0.34	0.22	-1.36

Countries	Causes	Measure	Cases in 1990 No.×10^3	Cases in 2019 No.×10^3	Change in absolute number (100%)	ASR in 1990 per 100000	ASR in 2019 per 100000	EAPC from 1990 and 2019
**Iran (Islamic Republic of)**	**Leukemia**	Incidence	6.09	6.77	0.11	11.62	8.85	-0.89
**Iran (Islamic Republic of)**	**Leukemia**	Deaths	3.29	4.42	0.35	8.29	6.04	-0.93
**Iran (Islamic Republic of)**	**Multiple myeloma**	Incidence	0.28	0.89	2.12	1.09	1.22	0.32
**Iran (Islamic Republic of)**	**Multiple myeloma**	Deaths	0.24	0.68	1.86	0.97	0.96	-0.01
**Iran (Islamic Republic of)**	**Non-Hodgkin lymphoma**	Incidence	0.93	3.03	2.26	2.4	3.96	1.59
**Iran (Islamic Republic of)**	**Non-Hodgkin lymphoma**	Deaths	0.61	1.58	1.58	1.93	2.13	0.35
**Iran (Islamic Republic of)**	**Hodgkin lymphoma**	Incidence	0.28	1.1	2.92	0.66	1.32	2.74
**Iran (Islamic Republic of)**	**Hodgkin lymphoma**	Deaths	0.16	0.31	0.93	0.45	0.39	0.17
**Iraq**	**Leukemia**	Incidence	2	3.18	0.59	12.15	9.89	-0.62
**Iraq**	**Leukemia**	Deaths	0.98	1.88	0.91	8.23	6.95	-0.62
**Iraq**	**Multiple myeloma**	Incidence	0.08	0.25	2.33	0.98	1.05	0.18
**Iraq**	**Multiple myeloma**	Deaths	0.07	0.2	1.94	0.88	0.86	-0.2
**Iraq**	**Non-Hodgkin lymphoma**	Incidence	0.43	1.06	1.44	3.59	3.57	0.07
**Iraq**	**Non-Hodgkin lymphoma**	Deaths	0.33	0.64	0.92	3.35	2.46	-1.12
**Iraq**	**Hodgkin lymphoma**	Incidence	0.11	0.27	1.4	0.85	0.71	-0.86
**Iraq**	**Hodgkin lymphoma**	Deaths	0.07	0.1	0.41	0.62	0.33	-2.64
**Ireland**	**Leukemia**	Incidence	0.4	0.97	1.45	10.05	15.06	1.49
**Ireland**	**Leukemia**	Deaths	0.24	0.31	0.29	5.97	4.25	-1.38
**Ireland**	**Multiple myeloma**	Incidence	0.18	0.36	1.02	4.26	4.74	0.13
**Ireland**	**Multiple myeloma**	Deaths	0.14	0.24	0.76	3.28	3.13	-0.45
**Ireland**	**Non-Hodgkin lymphoma**	Incidence	0.38	0.87	1.29	9.38	12	0.51

Countries	Causes	Measure	Cases in 1990 No.×10^3	Cases in 2019 No.×10^3	Change in absolute number (100%)	ASR in 1990 per 100000	ASR in 2019 per 100000	EAPC from 1990 and 2019
**Ireland**	**Non-Hodgkin lymphoma**	Deaths	0.21	0.34	0.65	5.07	4.57	-0.79
**Ireland**	**Hodgkin lymphoma**	Incidence	0.09	0.18	1.01	2.44	3.47	1.66
**Ireland**	**Hodgkin lymphoma**	Deaths	0.03	0.02	-0.24	0.71	0.32	-2.67
**Israel**	**Leukemia**	Incidence	0.52	1.61	2.12	10.75	14.78	0.88
**Israel**	**Leukemia**	Deaths	0.35	0.73	1.07	7.49	6.24	-0.9
**Israel**	**Multiple myeloma**	Incidence	0.18	0.5	1.78	3.68	4.3	0.38
**Israel**	**Multiple myeloma**	Deaths	0.15	0.36	1.44	3.07	3.04	-0.16
**Israel**	**Non-Hodgkin lymphoma**	Incidence	0.46	1.51	2.26	9.57	13.2	0.5
**Israel**	**Non-Hodgkin lymphoma**	Deaths	0.29	0.7	1.43	6.07	5.98	-0.65
**Israel**	**Hodgkin lymphoma**	Incidence	0.09	0.25	1.75	1.9	2.72	0.87
**Israel**	**Hodgkin lymphoma**	Deaths	0.03	0.04	0.25	0.68	0.39	-2.3
**Italy**	**Leukemia**	Incidence	10.77	20.46	0.9	15.83	20.87	0.75
**Italy**	**Leukemia**	Deaths	5.21	7.15	0.37	6.55	5.1	-0.92
**Italy**	**Multiple myeloma**	Incidence	3.28	6.6	1.01	3.67	4.63	0.69
**Italy**	**Multiple myeloma**	Deaths	2.19	4.12	0.88	2.4	2.64	0.21
**Italy**	**Non-Hodgkin lymphoma**	Incidence	8.14	14.29	0.75	10.05	10.8	-0.26
**Italy**	**Non-Hodgkin lymphoma**	Deaths	4.01	5.9	0.47	4.74	4.07	-1.02
**Italy**	**Hodgkin lymphoma**	Incidence	2.13	2.35	0.1	3.28	3.48	0.43
**Italy**	**Hodgkin lymphoma**	Deaths	0.63	0.47	-0.26	0.83	0.42	-2.12
**Jamaica**	**Leukemia**	Incidence	0.1	0.18	0.87	4.38	6.36	1.13
**Jamaica**	**Leukemia**	Deaths	0.06	0.13	1.19	2.88	4.45	1.32

Countries	Causes	Measure	Cases in 1990 No.×10^3	Cases in 2019 No.×10^3	Change in absolute number (100%)	ASR in 1990 per 100000	ASR in 2019 per 100000	EAPC from 1990 and 2019
**Jamaica**	**Multiple myeloma**	Incidence	0.04	0.14	2.72	2.06	4.63	4.15
**Jamaica**	**Multiple myeloma**	Deaths	0.03	0.1	2.49	1.66	3.52	3.96
**Jamaica**	**Non-Hodgkin lymphoma**	Incidence	0.09	0.23	1.52	4.56	7.68	1.21
**Jamaica**	**Non-Hodgkin lymphoma**	Deaths	0.07	0.16	1.24	3.77	5.27	0.59
**Jamaica**	**Hodgkin lymphoma**	Incidence	0.01	0.01	0.73	0.36	0.44	0.51
**Jamaica**	**Hodgkin lymphoma**	Deaths	0	0.01	0.3	0.23	0.2	-0.63
**Japan**	**Leukemia**	Incidence	12.56	22.63	0.8	9.14	10.81	0.62
**Japan**	**Leukemia**	Deaths	6.71	9.86	0.47	4.43	3.09	-1.23
**Japan**	**Multiple myeloma**	Incidence	3.12	7.45	1.39	1.84	1.99	0.24
**Japan**	**Multiple myeloma**	Deaths	2.35	5.38	1.29	1.4	1.33	-0.46
**Japan**	**Non-Hodgkin lymphoma**	Incidence	10.91	30.25	1.77	6.83	8.32	0.62
**Japan**	**Non-Hodgkin lymphoma**	Deaths	5.67	13.72	1.42	3.47	3.44	-0.14
**Japan**	**Hodgkin lymphoma**	Incidence	3.71	7.66	1.07	2.32	2.59	0.82
**Japan**	**Hodgkin lymphoma**	Deaths	0.16	0.24	0.49	0.1	0.09	-0.09
**Jordan**	**Leukemia**	Incidence	0.33	0.87	1.61	11.83	9.86	-0.83
**Jordan**	**Leukemia**	Deaths	0.16	0.45	1.76	8.61	6.62	-1
**Jordan**	**Multiple myeloma**	Incidence	0.02	0.08	3.09	1.52	1.24	-1.16
**Jordan**	**Multiple myeloma**	Deaths	0.02	0.06	2.6	1.39	1	-1.61
**Jordan**	**Non-Hodgkin lymphoma**	Incidence	0.12	0.57	3.88	6.35	7.46	0.36
**Jordan**	**Non-Hodgkin lymphoma**	Deaths	0.1	0.31	2.2	6.35	4.52	-1.57
**Jordan**	**Hodgkin lymphoma**	Incidence	0.01	0.04	3.02	0.35	0.35	-0.47

Countries	Causes	Measure	Cases in 1990 No.×10^3	Cases in 2019 No.×10^3	Change in absolute number (100%)	ASR in 1990 per 100000	ASR in 2019 per 100000	EAPC from 1990 and 2019
**Jordan**	**Hodgkin lymphoma**	Deaths	0.01	0.01	0.85	0.25	0.12	-3.21
**Kazakhstan**	**Leukemia**	Incidence	1.23	0.98	-0.21	7.74	5.45	-0.74
**Kazakhstan**	**Leukemia**	Deaths	0.76	0.62	-0.19	5.09	3.56	-0.78
**Kazakhstan**	**Multiple myeloma**	Incidence	0.08	0.15	0.97	0.58	0.82	1.19
**Kazakhstan**	**Multiple myeloma**	Deaths	0.06	0.12	0.88	0.5	0.68	0.97
**Kazakhstan**	**Non-Hodgkin lymphoma**	Incidence	0.37	0.46	0.24	2.43	2.58	-1.46
**Kazakhstan**	**Non-Hodgkin lymphoma**	Deaths	0.24	0.26	0.08	1.72	1.49	-2.16
**Kazakhstan**	**Hodgkin lymphoma**	Incidence	0.18	0.24	0.32	1.11	1.27	0.04
**Kazakhstan**	**Hodgkin lymphoma**	Deaths	0.1	0.08	-0.18	0.65	0.43	-2.01
**Kenya**	**Leukemia**	Incidence	0.93	1.28	0.37	3.61	3.25	-0.03
**Kenya**	**Leukemia**	Deaths	0.45	0.8	0.8	2.45	2.53	0.41
**Kenya**	**Multiple myeloma**	Incidence	0.09	0.32	2.7	1.06	1.48	1.43
**Kenya**	**Multiple myeloma**	Deaths	0.08	0.31	2.83	1.03	1.51	1.77
**Kenya**	**Non-Hodgkin lymphoma**	Incidence	0.22	0.91	3.1	2.06	3.34	2.05
**Kenya**	**Non-Hodgkin lymphoma**	Deaths	0.23	0.9	2.97	2.3	3.82	2.27
**Kenya**	**Hodgkin lymphoma**	Incidence	0.07	0.22	2.01	0.46	0.56	0.93
**Kenya**	**Hodgkin lymphoma**	Deaths	0.05	0.16	1.99	0.39	0.46	1.1
**Kiribati**	**Leukemia**	Incidence	0	0.01	0.36	6.78	5.92	-0.56
**Kiribati**	**Leukemia**	Deaths	0	0	0.44	5.05	4.43	-0.53
**Kiribati**	**Multiple myeloma**	Incidence	0	0	0.65	1	0.9	-0.51
**Kiribati**	**Multiple myeloma**	Deaths	0	0	0.6	0.97	0.86	-0.54

Countries	Causes	Measure	Cases in 1990 No.×10^3	Cases in 2019 No.×10^3	Change in absolute number (100%)	ASR in 1990 per 100000	ASR in 2019 per 100000	EAPC from 1990 and 2019
**Kiribati**	**Non-Hodgkin lymphoma**	Incidence	0	0	0.77	2.62	2.58	-0.11
**Kiribati**	**Non-Hodgkin lymphoma**	Deaths	0	0	0.67	2.81	2.68	-0.19
**Kiribati**	**Hodgkin lymphoma**	Incidence	0	0	0.56	0.45	0.39	-0.5
**Kiribati**	**Hodgkin lymphoma**	Deaths	0	0	0.48	0.39	0.32	-0.68
**Kuwait**	**Leukemia**	Incidence	0.13	0.22	0.73	9.54	6.88	-1.04
**Kuwait**	**Leukemia**	Deaths	0.06	0.1	0.75	5.81	3.62	-1.58
**Kuwait**	**Multiple myeloma**	Incidence	0.01	0.03	3.43	1.23	1.25	0.25
**Kuwait**	**Multiple myeloma**	Deaths	0.01	0.02	2.96	1	0.93	-0.08
**Kuwait**	**Non-Hodgkin lymphoma**	Incidence	0.05	0.18	2.46	6.07	6.82	1.05
**Kuwait**	**Non-Hodgkin lymphoma**	Deaths	0.03	0.08	1.79	4.11	3.38	-0.08
**Kuwait**	**Hodgkin lymphoma**	Incidence	0.01	0.03	2.32	0.72	0.84	0.78
**Kuwait**	**Hodgkin lymphoma**	Deaths	0	0.01	0.65	0.31	0.17	-1.89
**Kyrgyzstan**	**Leukemia**	Incidence	0.35	0.23	-0.34	7.49	3.94	-2.36
**Kyrgyzstan**	**Leukemia**	Deaths	0.19	0.15	-0.19	4.56	2.85	-1.68
**Kyrgyzstan**	**Multiple myeloma**	Incidence	0.02	0.03	0.79	0.52	0.62	0.5
**Kyrgyzstan**	**Multiple myeloma**	Deaths	0.01	0.02	0.72	0.47	0.55	0.47
**Kyrgyzstan**	**Non-Hodgkin lymphoma**	Incidence	0.05	0.1	0.9	1.38	1.85	1.3
**Kyrgyzstan**	**Non-Hodgkin lymphoma**	Deaths	0.04	0.06	0.63	1.16	1.31	0.6
**Kyrgyzstan**	**Hodgkin lymphoma**	Incidence	0.02	0.03	0.14	0.61	0.45	-0.86
**Kyrgyzstan**	**Hodgkin lymphoma**	Deaths	0.02	0.01	-0.17	0.44	0.24	-1.89
**Lao People's Democratic**	**Leukemia**	Incidence	0.58	0.47	-0.19	13.11	7.64	-1.92

Countries	Causes	Measure	Cases in 1990 No.×10³	Cases in 2019 No.×10³	Change in absolute number (100%)	ASR in 1990 per 100000	ASR in 2019 per 100000	EAPC from 1990 and 2019
**Republic**								
**Lao People's Democratic Republic**	**Leukemia**	Deaths	0.3	0.29	-0.03	8.73	5.5	-1.75
**Lao People's Democratic Republic**	**Multiple myeloma**	Incidence	0.02	0.04	0.87	0.96	0.88	-0.51
**Lao People's Democratic Republic**	**Multiple myeloma**	Deaths	0.02	0.03	0.78	0.93	0.82	-0.64
**Lao People's Democratic Republic**	**Non-Hodgkin lymphoma**	Incidence	0.07	0.14	1.13	2.37	2.63	0.25
**Lao People's Democratic Republic**	**Non-Hodgkin lymphoma**	Deaths	0.07	0.13	0.9	2.54	2.67	0.05
**Lao People's Democratic Republic**	**Hodgkin lymphoma**	Incidence	0.02	0.02	0.19	0.63	0.38	-1.93
**Lao People's Democratic Republic**	**Hodgkin lymphoma**	Deaths	0.02	0.02	0.01	0.57	0.29	-2.4
**Latvia**	**Leukemia**	Incidence	0.34	0.3	-0.13	11.51	9.37	-0.61
**Latvia**	**Leukemia**	Deaths	0.24	0.18	-0.26	7.55	5.02	-1.42
**Latvia**	**Multiple myeloma**	Incidence	0.05	0.1	0.89	1.51	2.68	2.17
**Latvia**	**Multiple myeloma**	Deaths	0.04	0.07	0.72	1.17	1.81	1.61
**Latvia**	**Non-Hodgkin lymphoma**	Incidence	0.08	0.2	1.44	2.68	6.1	3.36
**Latvia**	**Non-Hodgkin lymphoma**	Deaths	0.05	0.11	1.07	1.64	3.07	2.42
**Latvia**	**Hodgkin lymphoma**	Incidence	0.05	0.04	-0.11	1.73	2.23	0.92
**Latvia**	**Hodgkin lymphoma**	Deaths	0.02	0.01	-0.5	0.71	0.4	-2.38
**Lebanon**	**Leukemia**	Incidence	0.28	0.62	1.19	10.42	12.05	0.61
**Lebanon**	**Leukemia**	Deaths	0.21	0.34	0.61	8.73	6.52	-0.98

Countries	Causes	Measure	Cases in 1990 No.×10³	Cases in 2019 No.×10³	Change in absolute number (100%)	ASR in 1990 per 100000	ASR in 2019 per 100000	EAPC from 1990 and 2019
**Lebanon**	**Multiple myeloma**	Incidence	0.08	0.18	1.38	3.39	3.43	0.04
**Lebanon**	**Multiple myeloma**	Deaths	0.06	0.13	1.08	3.05	2.59	-0.62
**Lebanon**	**Non-Hodgkin lymphoma**	Incidence	0.09	0.32	2.66	3.53	6.3	2.4
**Lebanon**	**Non-Hodgkin lymphoma**	Deaths	0.07	0.16	1.13	3.15	3.02	0.08
**Lebanon**	**Hodgkin lymphoma**	Incidence	0.08	0.29	2.76	2.79	5.67	2.88
**Lebanon**	**Hodgkin lymphoma**	Deaths	0.05	0.05	0.11	1.77	0.97	-2.07
**Lesotho**	**Leukemia**	Incidence	0.05	0.08	0.74	3.55	5.4	2.07
**Lesotho**	**Leukemia**	Deaths	0.03	0.06	0.81	2.99	4.53	2.01
**Lesotho**	**Multiple myeloma**	Incidence	0.01	0.03	1	1.49	2.32	1.98
**Lesotho**	**Multiple myeloma**	Deaths	0.01	0.03	0.97	1.44	2.22	1.96
**Lesotho**	**Non-Hodgkin lymphoma**	Incidence	0.02	0.05	1.4	1.96	3.54	2.59
**Lesotho**	**Non-Hodgkin lymphoma**	Deaths	0.02	0.05	1.34	2.1	3.72	2.51
**Lesotho**	**Hodgkin lymphoma**	Incidence	0.01	0.01	0.71	0.4	0.52	1.46
**Lesotho**	**Hodgkin lymphoma**	Deaths	0	0.01	0.66	0.35	0.44	1.38
**Liberia**	**Leukemia**	Incidence	0.09	0.13	0.43	4.68	3.86	-0.28
**Liberia**	**Leukemia**	Deaths	0.06	0.09	0.56	3.59	3.15	-0.15
**Liberia**	**Multiple myeloma**	Incidence	0.01	0.02	0.94	0.85	0.91	0.44
**Liberia**	**Multiple myeloma**	Deaths	0.01	0.02	0.83	0.83	0.86	0.31
**Liberia**	**Non-Hodgkin lymphoma**	Incidence	0.03	0.07	1.05	2.43	2.31	-0.18
**Liberia**	**Non-Hodgkin lymphoma**	Deaths	0.04	0.06	0.62	2.71	2.34	-0.47
**Liberia**	**Hodgkin lymphoma**	Incidence	0	0.01	1.21	0.33	0.31	-0.25

Countries	Causes	Measure	Cases in 1990 No.×10^3	Cases in 2019 No.×10^3	Change in absolute number (100%)	ASR in 1990 per 100000	ASR in 2019 per 100000	EAPC from 1990 and 2019
**Liberia**	**Hodgkin lymphoma**	Deaths	0	0.01	0.98	0.3	0.27	-0.44
**Libya**	**Leukemia**	Incidence	0.28	0.39	0.4	8.07	6.92	-0.28
**Libya**	**Leukemia**	Deaths	0.17	0.28	0.67	6.21	5.18	-0.49
**Libya**	**Multiple myeloma**	Incidence	0.03	0.09	2.08	1.67	1.87	0.53
**Libya**	**Multiple myeloma**	Deaths	0.03	0.07	1.84	1.49	1.55	0.24
**Libya**	**Non-Hodgkin lymphoma**	Incidence	0.07	0.22	1.92	2.93	3.76	1.3
**Libya**	**Non-Hodgkin lymphoma**	Deaths	0.06	0.14	1.34	2.74	2.58	0.07
**Libya**	**Hodgkin lymphoma**	Incidence	0.06	0.16	1.52	2.01	2.33	0.73
**Libya**	**Hodgkin lymphoma**	Deaths	0.04	0.06	0.7	1.36	1.01	-1.16
**Lithuania**	**Leukemia**	Incidence	0.43	0.41	-0.05	10.79	8.5	-0.64
**Lithuania**	**Leukemia**	Deaths	0.31	0.27	-0.13	7.43	5.13	-1.19
**Lithuania**	**Multiple myeloma**	Incidence	0.08	0.17	1.12	1.82	3.08	1.79
**Lithuania**	**Multiple myeloma**	Deaths	0.06	0.12	1	1.33	2.06	1.42
**Lithuania**	**Non-Hodgkin lymphoma**	Incidence	0.1	0.27	1.57	2.56	5.46	3.09
**Lithuania**	**Non-Hodgkin lymphoma**	Deaths	0.07	0.16	1.4	1.54	2.91	2.49
**Lithuania**	**Hodgkin lymphoma**	Incidence	0.09	0.06	-0.33	2.33	1.96	-1.22
**Lithuania**	**Hodgkin lymphoma**	Deaths	0.03	0.01	-0.53	0.73	0.34	-3.42
**Luxembourg**	**Leukemia**	Incidence	0.06	0.12	0.89	13.58	13.99	0.08
**Luxembourg**	**Leukemia**	Deaths	0.04	0.05	0.33	7.96	5.46	-1.34
**Luxembourg**	**Multiple myeloma**	Incidence	0.02	0.04	1.01	3.37	3.74	0.26
**Luxembourg**	**Multiple myeloma**	Deaths	0.01	0.03	0.85	2.62	2.61	-0.13

Countries	Causes	Measure	Cases in 1990 No.×10³	Cases in 2019 No.×10³	Change in absolute number (100%)	ASR in 1990 per 100000	ASR in 2019 per 100000	EAPC from 1990 and 2019
**Luxembourg**	**Non-Hodgkin lymphoma**	Incidence	0.04	0.09	1.04	8.23	9.09	0.02
**Luxembourg**	**Non-Hodgkin lymphoma**	Deaths	0.02	0.04	0.54	4.4	3.61	-0.96
**Luxembourg**	**Hodgkin lymphoma**	Incidence	0.01	0.02	0.78	1.99	2.25	0.16
**Luxembourg**	**Hodgkin lymphoma**	Deaths	0	0	-0.27	0.56	0.23	-3.19
**Madagascar**	**Leukemia**	Incidence	0.74	0.82	0.11	4.88	3.55	-0.94
**Madagascar**	**Leukemia**	Deaths	0.35	0.47	0.32	3.14	2.63	-0.48
**Madagascar**	**Multiple myeloma**	Incidence	0.05	0.11	1.15	0.97	0.98	-0.05
**Madagascar**	**Multiple myeloma**	Deaths	0.05	0.1	1.06	0.96	0.96	-0.1
**Madagascar**	**Non-Hodgkin lymphoma**	Incidence	0.15	0.3	0.97	2.24	2.24	0.03
**Madagascar**	**Non-Hodgkin lymphoma**	Deaths	0.16	0.3	0.86	2.53	2.52	0.01
**Madagascar**	**Hodgkin lymphoma**	Incidence	0.08	0.14	0.73	0.84	0.67	-0.65
**Madagascar**	**Hodgkin lymphoma**	Deaths	0.06	0.11	0.69	0.71	0.56	-0.66
**Malawi**	**Leukemia**	Incidence	0.55	0.53	-0.03	4.61	3.49	-0.9
**Malawi**	**Leukemia**	Deaths	0.26	0.31	0.16	3.03	2.55	-0.56
**Malawi**	**Multiple myeloma**	Incidence	0.02	0.05	1.09	0.63	0.7	0.39
**Malawi**	**Multiple myeloma**	Deaths	0.02	0.05	1.06	0.61	0.67	0.32
**Malawi**	**Non-Hodgkin lymphoma**	Incidence	0.35	0.38	0.09	5.43	4.24	-1.17
**Malawi**	**Non-Hodgkin lymphoma**	Deaths	0.35	0.35	-0.01	5.83	4.71	-1.01
**Malawi**	**Hodgkin lymphoma**	Incidence	0.05	0.09	0.84	0.67	0.62	-0.48
**Malawi**	**Hodgkin lymphoma**	Deaths	0.04	0.07	0.72	0.56	0.49	-0.66
**Malaysia**	**Leukemia**	Incidence	1.16	2	0.73	7.65	7.05	-0.37

Countries	Causes	Measure	Cases in 1990 No.×10^3	Cases in 2019 No.×10^3	Change in absolute number (100%)	ASR in 1990 per 100000	ASR in 2019 per 100000	EAPC from 1990 and 2019
**Malaysia**	**Leukemia**	Deaths	0.76	1.39	0.82	5.8	5.1	-0.55
**Malaysia**	**Multiple myeloma**	Incidence	0.08	0.28	2.51	0.88	1.04	0.55
**Malaysia**	**Multiple myeloma**	Deaths	0.07	0.22	2.21	0.79	0.86	0.21
**Malaysia**	**Non-Hodgkin lymphoma**	Incidence	0.44	1.97	3.48	3.86	7.04	2.04
**Malaysia**	**Non-Hodgkin lymphoma**	Deaths	0.39	1.26	2.22	3.85	4.71	0.54
**Malaysia**	**Hodgkin lymphoma**	Incidence	0.08	0.23	1.81	0.57	0.72	0.64
**Malaysia**	**Hodgkin lymphoma**	Deaths	0.05	0.07	0.46	0.38	0.24	-1.84
**Maldives**	**Leukemia**	Incidence	0.02	0.02	-0.02	9.27	5.51	-1.92
**Maldives**	**Leukemia**	Deaths	0.01	0.01	0.19	6.33	3.51	-2.31
**Maldives**	**Multiple myeloma**	Incidence	0	0	2.26	1.16	1.14	-0.32
**Maldives**	**Multiple myeloma**	Deaths	0	0	1.89	1.08	0.93	-0.82
**Maldives**	**Non-Hodgkin lymphoma**	Incidence	0	0.01	3.08	2.68	3.73	1.14
**Maldives**	**Non-Hodgkin lymphoma**	Deaths	0	0.01	1.33	2.82	2.21	-1.23
**Maldives**	**Hodgkin lymphoma**	Incidence	0	0.01	3.37	0.95	1.31	1.27
**Maldives**	**Hodgkin lymphoma**	Deaths	0	0	0.42	0.72	0.34	-2.83
**Mali**	**Leukemia**	Incidence	0.27	0.57	1.08	3.44	3.26	-0.17
**Mali**	**Leukemia**	Deaths	0.18	0.37	1.05	2.8	2.62	-0.23
**Mali**	**Multiple myeloma**	Incidence	0.03	0.06	1.21	0.61	0.66	0.22
**Mali**	**Multiple myeloma**	Deaths	0.02	0.05	1.14	0.6	0.62	0.13
**Mali**	**Non-Hodgkin lymphoma**	Incidence	0.03	0.09	1.73	0.61	0.68	0.31
**Mali**	**Non-Hodgkin lymphoma**	Deaths	0.04	0.08	1.32	0.66	0.7	0.16

Countries	Causes	Measure	Cases in 1990 No.×10^3	Cases in 2019 No.×10^3	Change in absolute number (100%)	ASR in 1990 per 100000	ASR in 2019 per 100000	EAPC from 1990 and 2019
**Mali**	**Hodgkin lymphoma**	Incidence	0.03	0.05	0.96	0.38	0.31	-0.77
**Mali**	**Hodgkin lymphoma**	Deaths	0.02	0.04	0.78	0.33	0.25	-0.93
**Malta**	**Leukemia**	Incidence	0.03	0.08	1.51	8.13	12.29	1.33
**Malta**	**Leukemia**	Deaths	0.02	0.03	0.53	5.24	3.86	-1.14
**Malta**	**Multiple myeloma**	Incidence	0.01	0.03	1.6	2.29	2.74	0.43
**Malta**	**Multiple myeloma**	Deaths	0.01	0.02	1.31	1.83	1.85	-0.13
**Malta**	**Non-Hodgkin lymphoma**	Incidence	0.03	0.08	1.55	7.23	9.35	0.7
**Malta**	**Non-Hodgkin lymphoma**	Deaths	0.02	0.03	0.92	4.1	3.7	-0.55
**Malta**	**Hodgkin lymphoma**	Incidence	0.01	0.02	0.74	2.58	3.63	1.32
**Malta**	**Hodgkin lymphoma**	Deaths	0	0	-0.11	0.82	0.42	-2.14
**Marshall Islands**	**Leukemia**	Incidence	0	0	0.38	7.71	7.31	-0.23
**Marshall Islands**	**Leukemia**	Deaths	0	0	0.47	6.39	5.77	-0.41
**Marshall Islands**	**Multiple myeloma**	Incidence	0	0	1.01	1.48	1.39	-0.29
**Marshall Islands**	**Multiple myeloma**	Deaths	0	0	0.91	1.4	1.29	-0.35
**Marshall Islands**	**Non-Hodgkin lymphoma**	Incidence	0	0	1.11	2.35	2.74	0.58
**Marshall Islands**	**Non-Hodgkin lymphoma**	Deaths	0	0	1.02	2.5	2.79	0.44
**Marshall Islands**	**Hodgkin lymphoma**	Incidence	0	0	0.42	0.57	0.46	-0.69
**Marshall Islands**	**Hodgkin lymphoma**	Deaths	0	0	0.35	0.47	0.36	-0.96
**Mauritania**	**Leukemia**	Incidence	0.07	0.1	0.6	4.1	3.53	-0.24
**Mauritania**	**Leukemia**	Deaths	0.05	0.07	0.58	3.49	2.89	-0.4
**Mauritania**	**Multiple myeloma**	Incidence	0.01	0.02	0.83	0.89	0.79	-0.27

Countries	Causes	Measure	Cases in 1990 No.×10^3	Cases in 2019 No.×10^3	Change in absolute number (100%)	ASR in 1990 per 100000	ASR in 2019 per 100000	EAPC from 1990 and 2019
**Mauritania**	**Multiple myeloma**	Deaths	0.01	0.01	0.74	0.85	0.73	-0.41
**Mauritania**	**Non-Hodgkin lymphoma**	Incidence	0.04	0.07	0.92	2.92	2.6	-0.61
**Mauritania**	**Non-Hodgkin lymphoma**	Deaths	0.04	0.06	0.64	3.07	2.54	-0.86
**Mauritania**	**Hodgkin lymphoma**	Incidence	0	0.01	0.62	0.32	0.25	-0.68
**Mauritania**	**Hodgkin lymphoma**	Deaths	0	0.01	0.38	0.29	0.2	-1.03
**Mauritius**	**Leukemia**	Incidence	0.07	0.08	0.1	6.8	5.84	-0.73
**Mauritius**	**Leukemia**	Deaths	0.03	0.05	0.46	3.77	3.39	-0.44
**Mauritius**	**Multiple myeloma**	Incidence	0.01	0.02	2.93	0.79	1.29	1.69
**Mauritius**	**Multiple myeloma**	Deaths	0	0.02	2.77	0.67	1.05	1.55
**Mauritius**	**Non-Hodgkin lymphoma**	Incidence	0.01	0.05	2.19	1.65	3.09	2.08
**Mauritius**	**Non-Hodgkin lymphoma**	Deaths	0.01	0.03	1.81	1.29	1.83	1.27
**Mauritius**	**Hodgkin lymphoma**	Incidence	0.01	0.01	0.71	0.67	0.84	0.21
**Mauritius**	**Hodgkin lymphoma**	Deaths	0	0	0.22	0.33	0.25	-1.14
**Mexico**	**Leukemia**	Incidence	5.8	7.79	0.34	6.87	6.44	-0.18
**Mexico**	**Leukemia**	Deaths	3.31	5.25	0.59	4.6	4.37	-0.13
**Mexico**	**Multiple myeloma**	Incidence	0.46	1.87	3.06	1.06	1.58	1.21
**Mexico**	**Multiple myeloma**	Deaths	0.39	1.44	2.7	0.92	1.24	0.89
**Mexico**	**Non-Hodgkin lymphoma**	Incidence	1.5	5.07	2.39	2.51	4.24	1.71
**Mexico**	**Non-Hodgkin lymphoma**	Deaths	1.26	3.4	1.7	2.4	2.9	0.6
**Mexico**	**Hodgkin lymphoma**	Incidence	0.81	1.38	0.7	1.24	1.11	-0.4
**Mexico**	**Hodgkin lymphoma**	Deaths	0.53	0.63	0.19	0.93	0.53	-1.91

Countries	Causes	Measure	Cases in 1990 No.×10^3	Cases in 2019 No.×10^3	Change in absolute number (100%)	ASR in 1990 per 100000	ASR in 2019 per 100000	EAPC from 1990 and 2019
**Micronesia (Federated States of)**	**Leukemia**	Incidence	0.01	0.01	-0.06	8.34	7.27	-0.57
**Micronesia (Federated States of)**	**Leukemia**	Deaths	0	0	0.02	6.76	5.78	-0.64
**Micronesia (Federated States of)**	**Multiple myeloma**	Incidence	0	0	0.47	1.62	1.54	-0.35
**Micronesia (Federated States of)**	**Multiple myeloma**	Deaths	0	0	0.38	1.53	1.4	-0.5
**Micronesia (Federated States of)**	**Non-Hodgkin lymphoma**	Incidence	0	0	0.59	2.48	3	0.63
**Micronesia (Federated States of)**	**Non-Hodgkin lymphoma**	Deaths	0	0	0.46	2.63	2.93	0.34
**Micronesia (Federated States of)**	**Hodgkin lymphoma**	Incidence	0	0	0.1	0.55	0.46	-0.65
**Micronesia (Federated States of)**	**Hodgkin lymphoma**	Deaths	0	0	-0.09	0.47	0.32	-1.28
**Monaco**	**Leukemia**	Incidence	0.01	0.02	0.85	22.51	27.38	0.94
**Monaco**	**Leukemia**	Deaths	0.01	0.01	0.52	9.2	9.66	0.4
**Monaco**	**Multiple myeloma**	Incidence	0.01	0.01	0.43	14.04	14.95	0.33
**Monaco**	**Multiple myeloma**	Deaths	0.01	0.01	0.32	10.08	9.81	0
**Monaco**	**Non-Hodgkin lymphoma**	Incidence	0.01	0.04	1.6	22.46	42.02	2.53
**Monaco**	**Non-Hodgkin lymphoma**	Deaths	0.01	0.01	1.17	10.05	15.78	1.92
**Monaco**	**Hodgkin lymphoma**	Incidence	0	0	0.46	7.54	9.75	0.97
**Monaco**	**Hodgkin lymphoma**	Deaths	0	0	-0.15	1.32	0.87	-1.42
**Mongolia**	**Leukemia**	Incidence	0.08	0.11	0.47	3.96	3.79	-0.28

Countries	Causes	Measure	Cases in 1990 No.×10³	Cases in 2019 No.×10³	Change in absolute number (100%)	ASR in 1990 per 100000	ASR in 2019 per 100000	EAPC from 1990 and 2019
**Mongolia**	**Leukemia**	Deaths	0.05	0.08	0.57	3.04	2.88	-0.38
**Mongolia**	**Multiple myeloma**	Incidence	0.01	0.02	1.44	0.63	0.67	0.02
**Mongolia**	**Multiple myeloma**	Deaths	0.01	0.01	1.28	0.58	0.6	-0.15
**Mongolia**	**Non-Hodgkin lymphoma**	Incidence	0.04	0.08	1.11	2.95	3.02	-0.55
**Mongolia**	**Non-Hodgkin lymphoma**	Deaths	0.04	0.06	0.7	3.18	2.64	-1.31
**Mongolia**	**Hodgkin lymphoma**	Incidence	0	0.01	0.95	0.32	0.28	-0.93
**Mongolia**	**Hodgkin lymphoma**	Deaths	0	0.01	0.46	0.28	0.18	-2.01
**Montenegro**	**Leukemia**	Incidence	0.04	0.09	0.99	7.13	10.03	1.43
**Montenegro**	**Leukemia**	Deaths	0.03	0.04	0.43	4.68	4.58	-0.03
**Montenegro**	**Multiple myeloma**	Incidence	0.01	0.01	0.67	1.23	1.29	0.34
**Montenegro**	**Multiple myeloma**	Deaths	0.01	0.01	0.73	1	1.06	0.33
**Montenegro**	**Non-Hodgkin lymphoma**	Incidence	0.03	0.05	0.77	4.13	5.22	1.2
**Montenegro**	**Non-Hodgkin lymphoma**	Deaths	0.01	0.02	0.6	2.29	2.45	0.53
**Montenegro**	**Hodgkin lymphoma**	Incidence	0.02	0.03	0.26	3.32	4.04	0.7
**Montenegro**	**Hodgkin lymphoma**	Deaths	0.01	0.01	-0.04	1.08	0.79	-1.38
**Morocco**	**Leukemia**	Incidence	0.64	0.99	0.54	3.16	3.06	-0.22
**Morocco**	**Leukemia**	Deaths	0.44	0.77	0.76	2.52	2.47	-0.17
**Morocco**	**Multiple myeloma**	Incidence	0.15	0.44	2.04	1.08	1.43	0.95
**Morocco**	**Multiple myeloma**	Deaths	0.13	0.37	1.84	1.01	1.25	0.73
**Morocco**	**Non-Hodgkin lymphoma**	Incidence	0.55	1.63	1.97	3.33	5.15	1.26
**Morocco**	**Non-Hodgkin lymphoma**	Deaths	0.52	1.32	1.53	3.48	4.32	0.65

Countries	Causes	Measure	Cases in 1990 No.×10^3	Cases in 2019 No.×10^3	Change in absolute number (100%)	ASR in 1990 per 100000	ASR in 2019 per 100000	EAPC from 1990 and 2019
**Morocco**	**Hodgkin lymphoma**	Incidence	0.24	0.39	0.64	1.15	1.1	-0.33
**Morocco**	**Hodgkin lymphoma**	Deaths	0.17	0.21	0.22	0.93	0.63	-1.39
**Mozambique**	**Leukemia**	Incidence	1.07	1.95	0.82	6.38	6.32	0.19
**Mozambique**	**Leukemia**	Deaths	0.5	0.93	0.85	4.11	4.32	0.43
**Mozambique**	**Multiple myeloma**	Incidence	0.07	0.16	1.35	1.17	1.49	0.99
**Mozambique**	**Multiple myeloma**	Deaths	0.07	0.15	1.26	1.18	1.46	0.86
**Mozambique**	**Non-Hodgkin lymphoma**	Incidence	0.14	0.48	2.32	2.44	4.03	2.18
**Mozambique**	**Non-Hodgkin lymphoma**	Deaths	0.17	0.5	2.05	2.91	4.68	2.06
**Mozambique**	**Hodgkin lymphoma**	Incidence	0.09	0.22	1.37	0.93	1	0.47
**Mozambique**	**Hodgkin lymphoma**	Deaths	0.07	0.16	1.17	0.83	0.84	0.27
**Myanmar**	**Leukemia**	Incidence	5.31	4.22	-0.2	12.72	8.36	-1.58
**Myanmar**	**Leukemia**	Deaths	2.81	2.66	-0.05	8.17	5.53	-1.51
**Myanmar**	**Multiple myeloma**	Incidence	0.19	0.37	0.97	0.8	0.81	-0.06
**Myanmar**	**Multiple myeloma**	Deaths	0.18	0.33	0.88	0.77	0.74	-0.23
**Myanmar**	**Non-Hodgkin lymphoma**	Incidence	2.53	5.53	1.19	9.73	11.92	0.7
**Myanmar**	**Non-Hodgkin lymphoma**	Deaths	2.7	5.64	1.09	10.91	12.73	0.58
**Myanmar**	**Hodgkin lymphoma**	Incidence	0.2	0.21	0.04	0.59	0.39	-1.68
**Myanmar**	**Hodgkin lymphoma**	Deaths	0.16	0.14	-0.14	0.51	0.27	-2.34
**Namibia**	**Leukemia**	Incidence	0.03	0.06	1.03	2.81	3.33	0.68
**Namibia**	**Leukemia**	Deaths	0.02	0.04	0.95	2.35	2.53	0.26
**Namibia**	**Multiple myeloma**	Incidence	0.01	0.02	1.52	1.04	1.35	0.77

Countries	Causes	Measure	Cases in 1990 No.×10^3	Cases in 2019 No.×10^3	Change in absolute number (100%)	ASR in 1990 per 100000	ASR in 2019 per 100000	EAPC from 1990 and 2019
**Namibia**	**Multiple myeloma**	Deaths	0.01	0.02	1.38	1	1.24	0.59
**Namibia**	**Non-Hodgkin lymphoma**	Incidence	0.02	0.06	1.73	2.74	3.66	0.84
**Namibia**	**Non-Hodgkin lymphoma**	Deaths	0.02	0.06	1.38	3.01	3.61	0.5
**Namibia**	**Hodgkin lymphoma**	Incidence	0.01	0.01	1.24	0.53	0.6	0.43
**Namibia**	**Hodgkin lymphoma**	Deaths	0	0.01	0.76	0.47	0.42	-0.32
**Nauru**	**Leukemia**	Incidence	0	0	-0.13	10.11	9.07	-0.51
**Nauru**	**Leukemia**	Deaths	0	0	-0.12	7.45	6.46	-0.65
**Nauru**	**Multiple myeloma**	Incidence	0	0	0.11	1.69	1.65	-0.36
**Nauru**	**Multiple myeloma**	Deaths	0	0	0.04	1.53	1.45	-0.43
**Nauru**	**Non-Hodgkin lymphoma**	Incidence	0	0	0.23	4.13	4.56	0.05
**Nauru**	**Non-Hodgkin lymphoma**	Deaths	0	0	0	4.04	3.85	-0.32
**Nauru**	**Hodgkin lymphoma**	Incidence	0	0	0.09	0.54	0.5	-0.4
**Nauru**	**Hodgkin lymphoma**	Deaths	0	0	-0.15	0.37	0.28	-0.84
**Nepal**	**Leukemia**	Incidence	0.87	0.89	0.02	4.7	3.59	-0.86
**Nepal**	**Leukemia**	Deaths	0.56	0.72	0.29	3.71	3.08	-0.59
**Nepal**	**Multiple myeloma**	Incidence	0.06	0.22	2.49	0.65	0.97	1.53
**Nepal**	**Multiple myeloma**	Deaths	0.06	0.19	2.38	0.63	0.91	1.39
**Nepal**	**Non-Hodgkin lymphoma**	Incidence	0.19	0.52	1.73	1.49	2.1	1.27
**Nepal**	**Non-Hodgkin lymphoma**	Deaths	0.21	0.46	1.14	1.65	2.05	0.87
**Nepal**	**Hodgkin lymphoma**	Incidence	0.12	0.14	0.23	0.77	0.51	-1.46
**Nepal**	**Hodgkin lymphoma**	Deaths	0.09	0.1	0.07	0.68	0.38	-1.95

Countries	Causes	Measure	Cases in 1990 No.×10^3	Cases in 2019 No.×10^3	Change in absolute number (100%)	ASR in 1990 per 100000	ASR in 2019 per 100000	EAPC from 1990 and 2019
**Netherlands**	**Leukemia**	Incidence	2.11	3.97	0.88	12.36	15.3	0.66
**Netherlands**	**Leukemia**	Deaths	1.01	1.83	0.82	5.33	5.43	0.16
**Netherlands**	**Multiple myeloma**	Incidence	0.84	1.69	1.01	4.2	4.88	0.29
**Netherlands**	**Multiple myeloma**	Deaths	0.63	1.17	0.86	3.08	3.25	-0.07
**Netherlands**	**Non-Hodgkin lymphoma**	Incidence	2.45	4.08	0.67	12.72	12.38	-0.55
**Netherlands**	**Non-Hodgkin lymphoma**	Deaths	1.21	1.64	0.35	6.14	4.74	-1.39
**Netherlands**	**Hodgkin lymphoma**	Incidence	0.47	0.63	0.35	2.76	3.16	0.8
**Netherlands**	**Hodgkin lymphoma**	Deaths	0.11	0.09	-0.16	0.56	0.31	-1.95
**New Zealand**	**Leukemia**	Incidence	0.42	0.83	0.97	11.43	12.46	0.17
**New Zealand**	**Leukemia**	Deaths	0.23	0.38	0.63	6.12	5.05	-0.8
**New Zealand**	**Multiple myeloma**	Incidence	0.19	0.48	1.45	4.9	6.08	0.59
**New Zealand**	**Multiple myeloma**	Deaths	0.11	0.24	1.15	2.87	3.03	-0.06
**New Zealand**	**Non-Hodgkin lymphoma**	Incidence	0.44	0.84	0.92	11.28	11.22	-0.67
**New Zealand**	**Non-Hodgkin lymphoma**	Deaths	0.22	0.36	0.6	5.72	4.57	-1.4
**New Zealand**	**Hodgkin lymphoma**	Incidence	0.05	0.09	0.84	1.33	1.74	0.96
**New Zealand**	**Hodgkin lymphoma**	Deaths	0.02	0.02	0.15	0.48	0.33	-1.21
**Nicaragua**	**Leukemia**	Incidence	0.24	0.34	0.44	5.95	6.06	0.22
**Nicaragua**	**Leukemia**	Deaths	0.14	0.23	0.69	4.19	4.41	0.28
**Nicaragua**	**Multiple myeloma**	Incidence	0.01	0.04	3.02	0.66	0.94	0.9
**Nicaragua**	**Multiple myeloma**	Deaths	0.01	0.03	2.65	0.6	0.77	0.53
**Nicaragua**	**Non-Hodgkin lymphoma**	Incidence	0.05	0.18	2.87	1.75	3.6	3

Countries	Causes	Measure	Cases in 1990 No.×10^3	Cases in 2019 No.×10^3	Change in absolute number (100%)	ASR in 1990 per 100000	ASR in 2019 per 100000	EAPC from 1990 and 2019
**Nicaragua**	**Non-Hodgkin lymphoma**	Deaths	0.04	0.11	2.04	1.69	2.51	1.58
**Nicaragua**	**Hodgkin lymphoma**	Incidence	0.01	0.04	1.74	0.5	0.64	0.99
**Nicaragua**	**Hodgkin lymphoma**	Deaths	0.01	0.02	0.8	0.41	0.34	-0.72
**Niger**	**Leukemia**	Incidence	0.37	0.94	1.52	4.48	4.43	0.09
**Niger**	**Leukemia**	Deaths	0.2	0.51	1.51	3.31	3.32	0.09
**Niger**	**Multiple myeloma**	Incidence	0.02	0.06	2.05	0.66	0.73	0.32
**Niger**	**Multiple myeloma**	Deaths	0.02	0.05	1.98	0.65	0.69	0.24
**Niger**	**Non-Hodgkin lymphoma**	Incidence	0.04	0.11	1.86	0.8	0.79	-0.18
**Niger**	**Non-Hodgkin lymphoma**	Deaths	0.04	0.09	1.29	0.87	0.8	-0.43
**Niger**	**Hodgkin lymphoma**	Incidence	0.02	0.04	1.51	0.3	0.26	-0.58
**Niger**	**Hodgkin lymphoma**	Deaths	0.01	0.03	1.36	0.27	0.23	-0.73
**Nigeria**	**Leukemia**	Incidence	2.61	5.2	1	3.48	3.46	0.17
**Nigeria**	**Leukemia**	Deaths	1.8	3.5	0.95	2.92	2.87	0.1
**Nigeria**	**Multiple myeloma**	Incidence	0.35	0.82	1.32	0.82	0.97	0.72
**Nigeria**	**Multiple myeloma**	Deaths	0.33	0.74	1.22	0.79	0.91	0.59
**Nigeria**	**Non-Hodgkin lymphoma**	Incidence	0.84	2.52	1.99	1.36	1.84	1.36
**Nigeria**	**Non-Hodgkin lymphoma**	Deaths	0.83	2	1.42	1.41	1.72	0.97
**Nigeria**	**Hodgkin lymphoma**	Incidence	0.84	2.06	1.46	1.35	1.33	0.07
**Nigeria**	**Hodgkin lymphoma**	Deaths	0.71	1.52	1.13	1.23	1.11	-0.3
**Niue**	**Leukemia**	Incidence	0	0	-0.28	7.11	6.58	-0.41
**Niue**	**Leukemia**	Deaths	0	0	-0.29	5.32	4.4	-0.84

Countries	Causes	Measure	Cases in 1990 No.×10^3	Cases in 2019 No.×10^3	Change in absolute number (100%)	ASR in 1990 per 100000	ASR in 2019 per 100000	EAPC from 1990 and 2019
**Niue**	**Multiple myeloma**	Incidence	0	0	-0.01	1.39	1.37	-0.24
**Niue**	**Multiple myeloma**	Deaths	0	0	-0.09	1.22	1.12	-0.46
**Niue**	**Non-Hodgkin lymphoma**	Incidence	0	0	0.26	2.94	4.14	1.21
**Niue**	**Non-Hodgkin lymphoma**	Deaths	0	0	-0.03	2.6	2.75	0.16
**Niue**	**Hodgkin lymphoma**	Incidence	0	0	-0.15	0.47	0.47	-0.15
**Niue**	**Hodgkin lymphoma**	Deaths	0	0	-0.44	0.27	0.16	-1.92
**North Macedonia**	**Leukemia**	Incidence	0.14	0.24	0.76	7.09	8.61	0.72
**North Macedonia**	**Leukemia**	Deaths	0.09	0.13	0.48	4.69	4.57	-0.17
**North Macedonia**	**Multiple myeloma**	Incidence	0.02	0.04	1.1	0.93	1.13	0.71
**North Macedonia**	**Multiple myeloma**	Deaths	0.02	0.03	1.04	0.81	0.94	0.53
**North Macedonia**	**Non-Hodgkin lymphoma**	Incidence	0.05	0.12	1.24	2.74	4.22	1.45
**North Macedonia**	**Non-Hodgkin lymphoma**	Deaths	0.04	0.06	0.66	2.03	2.17	0.15
**North Macedonia**	**Hodgkin lymphoma**	Incidence	0.03	0.05	0.56	1.55	2.03	0.47
**North Macedonia**	**Hodgkin lymphoma**	Deaths	0.02	0.02	-0.12	0.91	0.56	-2.27
**Northern Mariana Islands**	**Leukemia**	Incidence	0	0	0	8.96	6.29	-1.5
**Northern Mariana Islands**	**Leukemia**	Deaths	0	0	0.04	7.16	4.39	-1.94
**Northern Mariana Islands**	**Multiple myeloma**	Incidence	0	0	1.37	3.04	2.35	-1.29
**Northern Mariana Islands**	**Multiple myeloma**	Deaths	0	0	1.33	2.56	1.92	-1.35
**Northern Mariana**	**Non-Hodgkin lymphoma**	Incidence	0	0	1.75	3.23	5.46	2.16

Countries	Causes	Measure	Cases in 1990 No.×10^3	Cases in 2019 No.×10^3	Change in absolute number (100%)	ASR in 1990 per 100000	ASR in 2019 per 100000	EAPC from 1990 and 2019
**Islands**								
**Northern Mariana Islands**	**Non-Hodgkin lymphoma**	Deaths	0	0	1.75	2.38	3.41	1.85
**Northern Mariana Islands**	**Hodgkin lymphoma**	Incidence	0	0	0.55	0.34	0.41	0.48
**Northern Mariana Islands**	**Hodgkin lymphoma**	Deaths	0	0	0.45	0.14	0.13	0.06
**Norway**	**Leukemia**	Incidence	0.45	0.69	0.54	8.37	8.73	0.35
**Norway**	**Leukemia**	Deaths	0.25	0.29	0.16	3.97	3.07	-0.72
**Norway**	**Multiple myeloma**	Incidence	0.33	0.53	0.61	4.83	5.54	0.49
**Norway**	**Multiple myeloma**	Deaths	0.26	0.36	0.4	3.6	3.56	-0.08
**Norway**	**Non-Hodgkin lymphoma**	Incidence	0.72	0.92	0.27	11.3	9.78	-0.78
**Norway**	**Non-Hodgkin lymphoma**	Deaths	0.38	0.38	0	5.64	3.84	-1.63
**Norway**	**Hodgkin lymphoma**	Incidence	0.1	0.15	0.61	1.99	2.48	1.09
**Norway**	**Hodgkin lymphoma**	Deaths	0.03	0.02	-0.27	0.43	0.22	-2.04
**Oman**	**Leukemia**	Incidence	0.08	0.17	0.97	6.6	6.49	0.2
**Oman**	**Leukemia**	Deaths	0.05	0.09	0.73	5.37	4.45	-0.34
**Oman**	**Multiple myeloma**	Incidence	0.01	0.04	2.24	1.71	2.16	1.15
**Oman**	**Multiple myeloma**	Deaths	0.01	0.02	1.75	1.52	1.72	0.83
**Oman**	**Non-Hodgkin lymphoma**	Incidence	0.05	0.21	3.25	6.06	10.95	2.28
**Oman**	**Non-Hodgkin lymphoma**	Deaths	0.04	0.1	1.54	5.84	6.35	0.67
**Oman**	**Hodgkin lymphoma**	Incidence	0.01	0.07	3.56	1.08	1.56	1.45
**Oman**	**Hodgkin**	Deaths	0.01	0.01	0.49	0.73	0.4	-1.73

**Countries**	**Causes**	**Measure**	**Cases in 1990 No.×10^3**	**Cases in 2019 No.×10^3**	**Change in absolute number (100%)**	**ASR in 1990 per 100000**	**ASR in 2019 per 100000**	**EAPC from 1990 and 2019**
	**lymphoma**							
**Pakistan**	**Leukemia**	Incidence	5.69	10.83	0.91	5	5.48	0.37
**Pakistan**	**Leukemia**	Deaths	3.36	6.49	0.93	3.76	3.94	0.06
**Pakistan**	**Multiple myeloma**	Incidence	0.76	1.82	1.4	1.34	1.65	0.65
**Pakistan**	**Multiple myeloma**	Deaths	0.72	1.62	1.24	1.31	1.54	0.5
**Pakistan**	**Non-Hodgkin lymphoma**	Incidence	0.93	3.26	2.5	1.31	2.05	1.48
**Pakistan**	**Non-Hodgkin lymphoma**	Deaths	0.98	2.76	1.8	1.42	1.99	1.09
**Pakistan**	**Hodgkin lymphoma**	Incidence	1.43	3.29	1.3	1.66	1.67	-0.17
**Pakistan**	**Hodgkin lymphoma**	Deaths	1.15	2.27	0.97	1.45	1.28	-0.63
**Palau**	**Leukemia**	Incidence	0	0	0.53	3.19	2.97	-0.19
**Palau**	**Leukemia**	Deaths	0	0	0.55	2.59	2.28	-0.39
**Palau**	**Multiple myeloma**	Incidence	0	0	1.33	0.7	0.73	0.05
**Palau**	**Multiple myeloma**	Deaths	0	0	1.18	0.59	0.59	-0.07
**Palau**	**Non-Hodgkin lymphoma**	Incidence	0	0	1.17	2.21	2.8	0.71
**Palau**	**Non-Hodgkin lymphoma**	Deaths	0	0	0.87	1.82	1.83	-0.01
**Palau**	**Hodgkin lymphoma**	Incidence	0	0	0.79	0.27	0.29	0.29
**Palau**	**Hodgkin lymphoma**	Deaths	0	0	0.37	0.15	0.11	-0.8
**Palestine**	**Leukemia**	Incidence	0.21	0.32	0.5	12.51	9.52	-0.94
**Palestine**	**Leukemia**	Deaths	0.11	0.19	0.76	9.4	7.27	-0.98
**Palestine**	**Multiple myeloma**	Incidence	0.01	0.04	2	1.73	1.9	0.4
**Palestine**	**Multiple myeloma**	Deaths	0.01	0.04	1.72	1.53	1.57	0.16

Countries	Causes	Measure	Cases in 1990 No.×10^3	Cases in 2019 No.×10^3	Change in absolute number (100%)	ASR in 1990 per 100000	ASR in 2019 per 100000	EAPC from 1990 and 2019
**Palestine**	**Non-Hodgkin lymphoma**	Incidence	0.04	0.14	2.35	3.55	4.67	0.69
**Palestine**	**Non-Hodgkin lymphoma**	Deaths	0.03	0.08	1.46	3.41	3.3	-0.32
**Palestine**	**Hodgkin lymphoma**	Incidence	0.01	0.05	3.06	0.96	1.31	0.9
**Palestine**	**Hodgkin lymphoma**	Deaths	0.01	0.02	1.46	0.71	0.63	-0.45
**Panama**	**Leukemia**	Incidence	0.13	0.27	1.13	5.96	6.53	0.45
**Panama**	**Leukemia**	Deaths	0.09	0.19	1	4.69	4.46	0.02
**Panama**	**Multiple myeloma**	Incidence	0.02	0.08	2.48	1.52	1.89	0.35
**Panama**	**Multiple myeloma**	Deaths	0.02	0.06	2.2	1.32	1.49	0.06
**Panama**	**Non-Hodgkin lymphoma**	Incidence	0.06	0.2	2.3	3.42	4.78	0.99
**Panama**	**Non-Hodgkin lymphoma**	Deaths	0.05	0.12	1.54	3.02	3	0
**Panama**	**Hodgkin lymphoma**	Incidence	0.01	0.03	2.35	0.41	0.68	2.17
**Panama**	**Hodgkin lymphoma**	Deaths	0	0.01	1.08	0.26	0.25	0.45
**Papua New Guinea**	**Leukemia**	Incidence	0.26	0.59	1.29	6.63	6.59	0.02
**Papua New Guinea**	**Leukemia**	Deaths	0.14	0.32	1.29	4.57	4.5	-0.05
**Papua New Guinea**	**Multiple myeloma**	Incidence	0.02	0.04	1.61	0.89	0.91	0.06
**Papua New Guinea**	**Multiple myeloma**	Deaths	0.01	0.04	1.57	0.85	0.86	0.05
**Papua New Guinea**	**Non-Hodgkin lymphoma**	Incidence	0.02	0.05	1.73	0.79	0.86	0.23
**Papua New Guinea**	**Non-Hodgkin lymphoma**	Deaths	0.02	0.05	1.63	0.88	0.95	0.21
**Papua New Guinea**	**Hodgkin lymphoma**	Incidence	0.01	0.02	1.22	0.37	0.33	-0.35
**Papua New Guinea**	**Hodgkin lymphoma**	Deaths	0.01	0.02	1.13	0.32	0.27	-0.41
**Paraguay**	**Leukemia**	Incidence	0.25	0.38	0.49	6.41	6.06	-0.35

Countries	Causes	Measure	Cases in 1990 No.×10^3	Cases in 2019 No.×10^3	Change in absolute number (100%)	ASR in 1990 per 100000	ASR in 2019 per 100000	EAPC from 1990 and 2019
**Paraguay**	**Leukemia**	Deaths	0.14	0.27	0.92	4.37	4.52	0.1
**Paraguay**	**Multiple myeloma**	Incidence	0.02	0.07	2.25	0.94	1.22	0.72
**Paraguay**	**Multiple myeloma**	Deaths	0.02	0.05	2	0.83	0.99	0.48
**Paraguay**	**Non-Hodgkin lymphoma**	Incidence	0.06	0.23	3.12	1.97	3.89	2.37
**Paraguay**	**Non-Hodgkin lymphoma**	Deaths	0.05	0.16	2.33	1.93	2.87	1.48
**Paraguay**	**Hodgkin lymphoma**	Incidence	0.02	0.05	1.72	0.55	0.73	0.97
**Paraguay**	**Hodgkin lymphoma**	Deaths	0.01	0.02	0.89	0.4	0.36	-0.18
**Peru**	**Leukemia**	Incidence	1.46	2.18	0.5	6.93	6.59	-0.05
**Peru**	**Leukemia**	Deaths	0.87	1.45	0.68	4.81	4.4	-0.18
**Peru**	**Multiple myeloma**	Incidence	0.19	0.51	1.74	1.58	1.6	0.25
**Peru**	**Multiple myeloma**	Deaths	0.17	0.41	1.44	1.46	1.3	-0.23
**Peru**	**Non-Hodgkin lymphoma**	Incidence	0.55	2.1	2.8	3.7	6.45	2.16
**Peru**	**Non-Hodgkin lymphoma**	Deaths	0.52	1.44	1.76	3.82	4.46	0.61
**Peru**	**Hodgkin lymphoma**	Incidence	0.09	0.16	0.92	0.51	0.49	-0.04
**Peru**	**Hodgkin lymphoma**	Deaths	0.07	0.08	0.17	0.43	0.23	-2.03
**Philippines**	**Leukemia**	Incidence	5.36	6.95	0.3	9.38	6.79	-1.14
**Philippines**	**Leukemia**	Deaths	2.93	4.21	0.44	6.57	4.55	-1.49
**Philippines**	**Multiple myeloma**	Incidence	0.21	0.55	1.58	0.71	0.69	-0.27
**Philippines**	**Multiple myeloma**	Deaths	0.19	0.47	1.5	0.66	0.61	-0.38
**Philippines**	**Non-Hodgkin lymphoma**	Incidence	1.2	2.72	1.28	2.82	2.98	0.01
**Philippines**	**Non-Hodgkin lymphoma**	Deaths	1.01	2.18	1.16	2.71	2.58	-0.33

Countries	Causes	Measure	Cases in 1990 No.×10^3	Cases in 2019 No.×10^3	Change in absolute number (100%)	ASR in 1990 per 100000	ASR in 2019 per 100000	EAPC from 1990 and 2019
**Philippines**	**Hodgkin lymphoma**	Incidence	0.15	0.25	0.67	0.3	0.25	-0.89
**Philippines**	**Hodgkin lymphoma**	Deaths	0.09	0.14	0.49	0.22	0.15	-1.34
**Poland**	**Leukemia**	Incidence	3.15	6.33	1.01	7.82	10.3	1.19
**Poland**	**Leukemia**	Deaths	2.49	3.45	0.39	6	5.21	-0.51
**Poland**	**Multiple myeloma**	Incidence	0.67	1.66	1.48	1.51	2.38	1.54
**Poland**	**Multiple myeloma**	Deaths	0.64	1.62	1.52	1.46	2.27	1.47
**Poland**	**Non-Hodgkin lymphoma**	Incidence	1.44	4.21	1.93	3.42	6.71	2.24
**Poland**	**Non-Hodgkin lymphoma**	Deaths	1.05	2.21	1.09	2.47	3.31	0.81
**Poland**	**Hodgkin lymphoma**	Incidence	0.9	0.8	-0.11	2.23	1.93	-0.86
**Poland**	**Hodgkin lymphoma**	Deaths	0.55	0.24	-0.55	1.31	0.43	-4.43
**Portugal**	**Leukemia**	Incidence	1.06	2.4	1.26	9.64	13.54	1.14
**Portugal**	**Leukemia**	Deaths	0.73	1.07	0.48	5.96	4.65	-1.08
**Portugal**	**Multiple myeloma**	Incidence	0.26	0.64	1.49	1.82	2.67	1.05
**Portugal**	**Multiple myeloma**	Deaths	0.25	0.66	1.59	1.81	2.59	0.99
**Portugal**	**Non-Hodgkin lymphoma**	Incidence	0.75	2.1	1.8	5.95	9.78	1.42
**Portugal**	**Non-Hodgkin lymphoma**	Deaths	0.46	0.95	1.06	3.54	4.04	0.21
**Portugal**	**Hodgkin lymphoma**	Incidence	0.17	0.27	0.63	1.52	2.16	0.59
**Portugal**	**Hodgkin lymphoma**	Deaths	0.07	0.06	-0.23	0.59	0.31	-2.74
**Puerto Rico**	**Leukemia**	Incidence	0.25	0.37	0.52	6.88	6.76	-0.06
**Puerto Rico**	**Leukemia**	Deaths	0.19	0.27	0.39	5.38	4.06	-1.11
**Puerto Rico**	**Multiple myeloma**	Incidence	0.12	0.31	1.49	3.37	4.27	0.8

Countries	Causes	Measure	Cases in 1990 No.×10^3	Cases in 2019 No.×10^3	Change in absolute number (100%)	ASR in 1990 per 100000	ASR in 2019 per 100000	EAPC from 1990 and 2019
**Puerto Rico**	**Multiple myeloma**	Deaths	0.09	0.2	1.2	2.56	2.77	0.19
**Puerto Rico**	**Non-Hodgkin lymphoma**	Incidence	0.26	0.46	0.76	7.16	7.45	0.1
**Puerto Rico**	**Non-Hodgkin lymphoma**	Deaths	0.18	0.25	0.38	5.02	3.74	-1.15
**Puerto Rico**	**Hodgkin lymphoma**	Incidence	0.06	0.09	0.6	1.51	2.14	1.22
**Puerto Rico**	**Hodgkin lymphoma**	Deaths	0.02	0.02	-0.08	0.66	0.42	-1.89
**Qatar**	**Leukemia**	Incidence	0.02	0.13	5.8	9.6	13.28	1.46
**Qatar**	**Leukemia**	Deaths	0.01	0.06	3.67	8.04	8.54	0.36
**Qatar**	**Multiple myeloma**	Incidence	0	0.02	8.39	1.6	1.93	1
**Qatar**	**Multiple myeloma**	Deaths	0	0.01	6.63	1.42	1.54	0.56
**Qatar**	**Non-Hodgkin lymphoma**	Incidence	0.01	0.1	9.65	6.2	11.04	2.57
**Qatar**	**Non-Hodgkin lymphoma**	Deaths	0.01	0.04	4.92	5.87	5.97	0.3
**Qatar**	**Hodgkin lymphoma**	Incidence	0	0.03	15.19	0.53	0.95	2.56
**Qatar**	**Hodgkin lymphoma**	Deaths	0	0	3.25	0.37	0.21	-2.02
**Republic of Korea**	**Leukemia**	Incidence	3.1	5.98	0.93	7.8	9.19	0.64
**Republic of Korea**	**Leukemia**	Deaths	1.75	2.05	0.17	4.6	2.69	-2.07
**Republic of Korea**	**Multiple myeloma**	Incidence	0.37	1.63	3.36	1.28	1.81	1.77
**Republic of Korea**	**Multiple myeloma**	Deaths	0.31	1.11	2.58	1.1	1.23	0.91
**Republic of Korea**	**Non-Hodgkin lymphoma**	Incidence	1.16	5.46	3.69	3.09	6.7	1.94
**Republic of Korea**	**Non-Hodgkin lymphoma**	Deaths	0.73	2.33	2.19	2.16	2.74	0.16
**Republic of Korea**	**Hodgkin lymphoma**	Incidence	0.15	1.58	9.43	0.44	2.03	5.82
**Republic of Korea**	**Hodgkin lymphoma**	Deaths	0.04	0.06	0.69	0.1	0.07	-0.62

Countries	Causes	Measure	Cases in 1990 No.×10^3	Cases in 2019 No.×10^3	Change in absolute number (100%)	ASR in 1990 per 100000	ASR in 2019 per 100000	EAPC from 1990 and 2019
**Republic of Moldova**	**Leukemia**	Incidence	0.48	0.21	-0.57	10.89	5.01	-2.37
**Republic of Moldova**	**Leukemia**	Deaths	0.27	0.14	-0.48	6.03	2.9	-2.21
**Republic of Moldova**	**Multiple myeloma**	Incidence	0.03	0.07	0.95	0.76	1.16	1.74
**Republic of Moldova**	**Multiple myeloma**	Deaths	0.03	0.05	0.76	0.61	0.81	1.24
**Republic of Moldova**	**Non-Hodgkin lymphoma**	Incidence	0.12	0.2	0.68	2.65	4.2	1.71
**Republic of Moldova**	**Non-Hodgkin lymphoma**	Deaths	0.08	0.12	0.46	1.86	2.35	0.83
**Republic of Moldova**	**Hodgkin lymphoma**	Incidence	0.1	0.08	-0.2	2.15	1.95	-0.83
**Republic of Moldova**	**Hodgkin lymphoma**	Deaths	0.05	0.03	-0.45	1.09	0.57	-2.78
**Romania**	**Leukemia**	Incidence	1.53	2.13	0.4	6.3	7.36	0.72
**Romania**	**Leukemia**	Deaths	1.08	1.33	0.24	4.27	4.16	0.03
**Romania**	**Multiple myeloma**	Incidence	0.29	0.53	0.82	1.01	1.48	1.43
**Romania**	**Multiple myeloma**	Deaths	0.24	0.44	0.81	0.86	1.21	1.25
**Romania**	**Non-Hodgkin lymphoma**	Incidence	0.78	1.53	0.96	3.11	5.31	2.08
**Romania**	**Non-Hodgkin lymphoma**	Deaths	0.54	0.82	0.51	2.09	2.57	0.89
**Romania**	**Hodgkin lymphoma**	Incidence	0.41	0.33	-0.19	1.66	1.59	-0.43
**Romania**	**Hodgkin lymphoma**	Deaths	0.23	0.1	-0.54	0.89	0.38	-3.41
**Russian Federation**	**Leukemia**	Incidence	10.28	14.27	0.39	6.32	7.09	0.26
**Russian Federation**	**Leukemia**	Deaths	7.31	7.43	0.02	4.39	3.55	-1.01
**Russian Federation**	**Multiple myeloma**	Incidence	2.11	3.51	0.66	1.14	1.5	1.08
**Russian Federation**	**Multiple myeloma**	Deaths	1.72	2.72	0.59	0.94	1.15	0.8
**Russian Federation**	**Non-Hodgkin lymphoma**	Incidence	5.49	8.72	0.59	3.33	4.26	1.11

Countries	Causes	Measure	Cases in 1990 No.×10^3	Cases in 2019 No.×10^3	Change in absolute number (100%)	ASR in 1990 per 100000	ASR in 2019 per 100000	EAPC from 1990 and 2019
**Russian Federation**	**Non-Hodgkin lymphoma**	Deaths	3.62	4.7	0.3	2.11	2.15	0.04
**Russian Federation**	**Hodgkin lymphoma**	Incidence	3.65	4.07	0.11	2.33	2.65	0.2
**Russian Federation**	**Hodgkin lymphoma**	Deaths	1.38	0.85	-0.39	0.83	0.45	-2.88
**Rwanda**	**Leukemia**	Incidence	0.43	0.52	0.21	5.34	4.67	-0.56
**Rwanda**	**Leukemia**	Deaths	0.23	0.29	0.26	3.83	3.25	-0.88
**Rwanda**	**Multiple myeloma**	Incidence	0.05	0.09	0.85	1.6	1.46	-0.83
**Rwanda**	**Multiple myeloma**	Deaths	0.04	0.08	0.77	1.59	1.4	-0.97
**Rwanda**	**Non-Hodgkin lymphoma**	Incidence	0.1	0.21	1.06	2.9	2.99	-0.28
**Rwanda**	**Non-Hodgkin lymphoma**	Deaths	0.11	0.21	0.83	3.33	3.36	-0.37
**Rwanda**	**Hodgkin lymphoma**	Incidence	0.05	0.08	0.54	0.98	0.76	-1.37
**Rwanda**	**Hodgkin lymphoma**	Deaths	0.04	0.06	0.37	0.85	0.59	-1.76
**Saint Kitts and Nevis**	**Leukemia**	Incidence	0	0	0.08	8.67	6.22	-0.65
**Saint Kitts and Nevis**	**Leukemia**	Deaths	0	0	0.05	6.01	4.06	-0.99
**Saint Kitts and Nevis**	**Multiple myeloma**	Incidence	0	0	1.28	3.78	5.04	0.93
**Saint Kitts and Nevis**	**Multiple myeloma**	Deaths	0	0	0.97	3.03	3.63	0.63
**Saint Kitts and Nevis**	**Non-Hodgkin lymphoma**	Incidence	0	0	0.5	8.26	7.35	-0.18
**Saint Kitts and Nevis**	**Non-Hodgkin lymphoma**	Deaths	0	0	0.17	6.82	4.58	-1.28
**Saint Kitts and Nevis**	**Hodgkin lymphoma**	Incidence	0	0	2.18	0.13	0.25	2.37
**Saint Kitts and Nevis**	**Hodgkin lymphoma**	Deaths	0	0	1.1	0.08	0.1	0.77
**Saint Lucia**	**Leukemia**	Incidence	0.01	0.01	0.41	6.7	5.89	-0.58
**Saint Lucia**	**Leukemia**	Deaths	0.01	0.01	0.51	5.17	4.15	-0.97

Countries	Causes	Measure	Cases in 1990 No.×10^3	Cases in 2019 No.×10^3	Change in absolute number (100%)	ASR in 1990 per 100000	ASR in 2019 per 100000	EAPC from 1990 and 2019
**Saint Lucia**	**Multiple myeloma**	Incidence	0	0.01	2.3	3.91	5.21	0.65
**Saint Lucia**	**Multiple myeloma**	Deaths	0	0.01	1.99	3.29	3.97	0.3
**Saint Lucia**	**Non-Hodgkin lymphoma**	Incidence	0.01	0.02	1.01	7.43	7.66	-0.09
**Saint Lucia**	**Non-Hodgkin lymphoma**	Deaths	0.01	0.01	0.62	7.07	5.31	-1.2
**Saint Lucia**	**Hodgkin lymphoma**	Incidence	0	0	1.07	0.9	1.06	0.61
**Saint Lucia**	**Hodgkin lymphoma**	Deaths	0	0	0.52	0.65	0.5	-0.89
**Saint Vincent and the Grenadines**	**Leukemia**	Incidence	0.01	0.01	0.03	7.5	6.5	-0.61
**Saint Vincent and the Grenadines**	**Leukemia**	Deaths	0	0.01	0.27	5.18	4.72	-0.46
**Saint Vincent and the Grenadines**	**Multiple myeloma**	Incidence	0	0	1.51	1.64	2.19	1.01
**Saint Vincent and the Grenadines**	**Multiple myeloma**	Deaths	0	0	1.38	1.37	1.75	0.86
**Saint Vincent and the Grenadines**	**Non-Hodgkin lymphoma**	Incidence	0.01	0.01	0.55	8.24	8.82	0.22
**Saint Vincent and the Grenadines**	**Non-Hodgkin lymphoma**	Deaths	0.01	0.01	0.5	7.48	6.95	-0.26
**Saint Vincent and the Grenadines**	**Hodgkin lymphoma**	Incidence	0	0	1.82	0.42	0.9	1.3
**Saint Vincent and**	**Hodgkin lymphoma**	Deaths	0	0	1.62	0.3	0.52	0.63

Countries	Causes	Measure	Cases in 1990 No.×10³	Cases in 2019 No.×10³	Change in absolute number (100%)	ASR in 1990 per 100000	ASR in 2019 per 100000	EAPC from 1990 and 2019
the Grenadines								
Samoa	Leukemia	Incidence	0.01	0.01	0.21	6.74	5.92	-0.47
Samoa	Leukemia	Deaths	0.01	0.01	0.27	5.38	4.63	-0.52
Samoa	Multiple myeloma	Incidence	0	0	0.52	1.07	0.98	-0.55
Samoa	Multiple myeloma	Deaths	0	0	0.46	0.97	0.86	-0.65
Samoa	Non-Hodgkin lymphoma	Incidence	0	0	0.83	2.41	2.91	0.59
Samoa	Non-Hodgkin lymphoma	Deaths	0	0	0.64	2.45	2.56	0.2
Samoa	Hodgkin lymphoma	Incidence	0	0	0.34	1.98	1.81	-0.4
Samoa	Hodgkin lymphoma	Deaths	0	0	0.08	1.38	1	-1.01
San Marino	Leukemia	Incidence	0.01	0.02	1.66	22.33	35.14	1.74
San Marino	Leukemia	Deaths	0	0.01	0.95	8.74	8.22	0.03
San Marino	Multiple myeloma	Incidence	0	0	1.19	4.53	5.17	0.71
San Marino	Multiple myeloma	Deaths	0	0	1.2	3.38	3.62	0.57
San Marino	Non-Hodgkin lymphoma	Incidence	0.01	0.01	1.21	20.78	23.79	0.7
San Marino	Non-Hodgkin lymphoma	Deaths	0	0.01	0.97	9.9	9.56	0.17
San Marino	Hodgkin lymphoma	Incidence	0	0	0.99	5.67	7.59	0.93
San Marino	Hodgkin lymphoma	Deaths	0	0	0.28	1.18	0.84	-1.26
Sao Tome and Principe	Leukemia	Incidence	0	0.01	0.52	3.31	3.58	0.18
Sao Tome and Principe	Leukemia	Deaths	0	0	0.58	2.64	2.82	0.16
Sao Tome and Principe	Multiple myeloma	Incidence	0	0	1.42	0.69	1.04	1.39
Sao Tome and Principe	Multiple myeloma	Deaths	0	0	1.26	0.66	0.95	1.24

Countries	Causes	Measure	Cases in 1990 No.×10^3	Cases in 2019 No.×10^3	Change in absolute number (100%)	ASR in 1990 per 100000	ASR in 2019 per 100000	EAPC from 1990 and 2019
**Sao Tome and Principe**	**Non-Hodgkin lymphoma**	Incidence	0	0	1.18	2.24	3.1	0.84
**Sao Tome and Principe**	**Non-Hodgkin lymphoma**	Deaths	0	0	0.94	2.3	2.97	0.59
**Sao Tome and Principe**	**Hodgkin lymphoma**	Incidence	0	0	0.96	0.08	0.09	0.25
**Sao Tome and Principe**	**Hodgkin lymphoma**	Deaths	0	0	0.6	0.07	0.07	-0.22
**Saudi Arabia**	**Leukemia**	Incidence	0.44	1.38	2.14	4.41	5.36	0.47
**Saudi Arabia**	**Leukemia**	Deaths	0.35	0.77	1.2	4.26	3.43	-1.04
**Saudi Arabia**	**Multiple myeloma**	Incidence	0.05	0.25	3.7	0.92	1.3	1.16
**Saudi Arabia**	**Multiple myeloma**	Deaths	0.05	0.17	2.29	0.95	1.01	0
**Saudi Arabia**	**Non-Hodgkin lymphoma**	Incidence	0.31	1.77	4.62	4.25	8.33	2.19
**Saudi Arabia**	**Non-Hodgkin lymphoma**	Deaths	0.31	0.88	1.79	4.95	4.78	-0.51
**Saudi Arabia**	**Hodgkin lymphoma**	Incidence	0.06	0.42	6.23	0.51	1.11	3.05
**Saudi Arabia**	**Hodgkin lymphoma**	Deaths	0.04	0.09	1.16	0.43	0.3	-1.14
**Senegal**	**Leukemia**	Incidence	0.28	0.51	0.81	4.19	4.37	0.49
**Senegal**	**Leukemia**	Deaths	0.18	0.35	0.97	3.38	3.54	0.46
**Senegal**	**Multiple myeloma**	Incidence	0.02	0.06	1.64	0.75	0.85	0.63
**Senegal**	**Multiple myeloma**	Deaths	0.02	0.06	1.56	0.72	0.8	0.53
**Senegal**	**Non-Hodgkin lymphoma**	Incidence	0.12	0.28	1.42	2.56	2.79	0.13
**Senegal**	**Non-Hodgkin lymphoma**	Deaths	0.12	0.25	1.07	2.76	2.82	-0.13
**Senegal**	**Hodgkin lymphoma**	Incidence	0.02	0.04	1.28	0.33	0.35	0.45
**Senegal**	**Hodgkin lymphoma**	Deaths	0.01	0.03	1.17	0.3	0.3	0.31
**Serbia**	**Leukemia**	Incidence	0.83	1.07	0.29	8.61	7.92	-0.15

Countries	Causes	Measure	Cases in 1990 No.×10^3	Cases in 2019 No.×10^3	Change in absolute number (100%)	ASR in 1990 per 100000	ASR in 2019 per 100000	EAPC from 1990 and 2019
**Serbia**	**Leukemia**	Deaths	0.56	0.73	0.3	5.47	4.93	-0.22
**Serbia**	**Multiple myeloma**	Incidence	0.14	0.25	0.73	1.23	1.53	1.02
**Serbia**	**Multiple myeloma**	Deaths	0.14	0.25	0.76	1.27	1.55	0.95
**Serbia**	**Non-Hodgkin lymphoma**	Incidence	0.41	0.9	1.22	3.83	6.34	2.23
**Serbia**	**Non-Hodgkin lymphoma**	Deaths	0.3	0.48	0.63	2.73	3.23	0.98
**Serbia**	**Hodgkin lymphoma**	Incidence	0.18	0.29	0.56	1.81	2.71	1.55
**Serbia**	**Hodgkin lymphoma**	Deaths	0.09	0.07	-0.27	0.85	0.51	-1.89
**Seychelles**	**Leukemia**	Incidence	0.01	0.01	0.73	8.95	9.34	0.13
**Seychelles**	**Leukemia**	Deaths	0	0.01	0.5	7.1	6.36	-0.44
**Seychelles**	**Multiple myeloma**	Incidence	0	0	1.58	1.74	2.31	1.05
**Seychelles**	**Multiple myeloma**	Deaths	0	0	1.35	1.56	1.94	0.84
**Seychelles**	**Non-Hodgkin lymphoma**	Incidence	0	0.01	2.71	3.99	8.33	2.29
**Seychelles**	**Non-Hodgkin lymphoma**	Deaths	0	0.01	1.8	3.9	5.96	1.17
**Seychelles**	**Hodgkin lymphoma**	Incidence	0	0	0.96	0.88	1.05	0.41
**Seychelles**	**Hodgkin lymphoma**	Deaths	0	0	0.25	0.57	0.41	-1.28
**Sierra Leone**	**Leukemia**	Incidence	0.16	0.36	1.19	4.32	4.89	0.79
**Sierra Leone**	**Leukemia**	Deaths	0.1	0.21	1.12	3.27	3.6	0.63
**Sierra Leone**	**Multiple myeloma**	Incidence	0.01	0.03	1.09	0.72	0.8	0.5
**Sierra Leone**	**Multiple myeloma**	Deaths	0.01	0.03	0.99	0.7	0.75	0.4
**Sierra Leone**	**Non-Hodgkin lymphoma**	Incidence	0.05	0.13	1.34	2.21	2.38	0.06
**Sierra Leone**	**Non-Hodgkin lymphoma**	Deaths	0.06	0.12	0.92	2.45	2.44	-0.21

Countries	Causes	Measure	Cases in 1990 No.×10^3	Cases in 2019 No.×10^3	Change in absolute number (100%)	ASR in 1990 per 100000	ASR in 2019 per 100000	EAPC from 1990 and 2019
**Sierra Leone**	**Hodgkin lymphoma**	Incidence	0.01	0.02	1.39	0.3	0.32	0.43
**Sierra Leone**	**Hodgkin lymphoma**	Deaths	0.01	0.02	1.19	0.27	0.28	0.3
**Singapore**	**Leukemia**	Incidence	0.17	0.55	2.18	6.84	8.7	1.28
**Singapore**	**Leukemia**	Deaths	0.11	0.19	0.7	4.53	2.75	-1.7
**Singapore**	**Multiple myeloma**	Incidence	0.03	0.1	2.62	1.35	1.35	-0.01
**Singapore**	**Multiple myeloma**	Deaths	0.02	0.07	2.1	1.11	0.93	-0.65
**Singapore**	**Non-Hodgkin lymphoma**	Incidence	0.14	0.5	2.44	5.92	6.9	0.37
**Singapore**	**Non-Hodgkin lymphoma**	Deaths	0.08	0.21	1.55	3.62	2.83	-1.06
**Singapore**	**Hodgkin lymphoma**	Incidence	0.03	0.16	3.58	1.34	2.23	2.69
**Singapore**	**Hodgkin lymphoma**	Deaths	0	0.01	0.52	0.15	0.08	-1.45
**Slovakia**	**Leukemia**	Incidence	0.43	0.75	0.74	7.56	9.49	0.96
**Slovakia**	**Leukemia**	Deaths	0.32	0.38	0.18	5.53	4.46	-0.57
**Slovakia**	**Multiple myeloma**	Incidence	0.17	0.33	0.94	2.85	3.57	0.96
**Slovakia**	**Multiple myeloma**	Deaths	0.1	0.18	0.78	1.67	1.91	0.54
**Slovakia**	**Non-Hodgkin lymphoma**	Incidence	0.27	0.7	1.6	4.62	8.19	2.41
**Slovakia**	**Non-Hodgkin lymphoma**	Deaths	0.18	0.35	0.99	3.01	3.94	1.4
**Slovakia**	**Hodgkin lymphoma**	Incidence	0.11	0.15	0.45	1.92	2.54	1.08
**Slovakia**	**Hodgkin lymphoma**	Deaths	0.05	0.03	-0.25	0.79	0.44	-1.94
**Slovenia**	**Leukemia**	Incidence	0.2	0.47	1.32	8.93	12.92	1.45
**Slovenia**	**Leukemia**	Deaths	0.13	0.19	0.5	5.56	4.58	-0.62
**Slovenia**	**Multiple myeloma**	Incidence	0.06	0.14	1.37	2.41	3.26	1.06

Countries	Causes	Measure	Cases in 1990 No.×10^3	Cases in 2019 No.×10^3	Change in absolute number (100%)	ASR in 1990 per 100000	ASR in 2019 per 100000	EAPC from 1990 and 2019
**Slovenia**	**Multiple myeloma**	Deaths	0.05	0.12	1.34	2.02	2.55	0.79
**Slovenia**	**Non-Hodgkin lymphoma**	Incidence	0.13	0.44	2.49	5.4	10.58	2.64
**Slovenia**	**Non-Hodgkin lymphoma**	Deaths	0.08	0.2	1.65	3.18	4.48	1.38
**Slovenia**	**Hodgkin lymphoma**	Incidence	0.04	0.06	0.43	1.9	2.57	1.39
**Slovenia**	**Hodgkin lymphoma**	Deaths	0.01	0.01	-0.31	0.55	0.25	-2.75
**Solomon Islands**	**Leukemia**	Incidence	0.02	0.05	1	8.98	9.03	-0.02
**Solomon Islands**	**Leukemia**	Deaths	0.01	0.03	1.03	7.05	6.83	-0.14
**Solomon Islands**	**Multiple myeloma**	Incidence	0	0.01	1.3	1.52	1.56	0.03
**Solomon Islands**	**Multiple myeloma**	Deaths	0	0	1.22	1.41	1.41	-0.05
**Solomon Islands**	**Non-Hodgkin lymphoma**	Incidence	0.01	0.03	1.58	6.7	7.7	0.34
**Solomon Islands**	**Non-Hodgkin lymphoma**	Deaths	0.01	0.03	1.3	7.01	7.57	0.14
**Solomon Islands**	**Hodgkin lymphoma**	Incidence	0	0	1.06	0.6	0.54	-0.32
**Solomon Islands**	**Hodgkin lymphoma**	Deaths	0	0	0.8	0.51	0.4	-0.66
**Somalia**	**Leukemia**	Incidence	0.42	1.01	1.4	5.13	4.82	0.13
**Somalia**	**Leukemia**	Deaths	0.2	0.51	1.51	3.5	3.49	0.24
**Somalia**	**Multiple myeloma**	Incidence	0.03	0.08	1.64	1.18	1.19	0.17
**Somalia**	**Multiple myeloma**	Deaths	0.03	0.07	1.67	1.18	1.19	0.19
**Somalia**	**Non-Hodgkin lymphoma**	Incidence	0.06	0.16	1.95	1.87	1.89	0.2
**Somalia**	**Non-Hodgkin lymphoma**	Deaths	0.06	0.17	1.72	2.18	2.16	0.17
**Somalia**	**Hodgkin lymphoma**	Incidence	0.05	0.15	2.24	0.94	1.01	0.24
**Somalia**	**Hodgkin lymphoma**	Deaths	0.04	0.12	2.17	0.85	0.91	0.24

Countries	Causes	Measure	Cases in 1990 No.×10^3	Cases in 2019 No.×10^3	Change in absolute number (100%)	ASR in 1990 per 100000	ASR in 2019 per 100000	EAPC from 1990 and 2019
**South Africa**	**Leukemia**	Incidence	1.46	2.09	0.43	4.92	4.37	-0.46
**South Africa**	**Leukemia**	Deaths	0.96	1.49	0.56	3.72	3.34	-0.41
**South Africa**	**Multiple myeloma**	Incidence	0.35	0.9	1.55	1.68	2.01	0.6
**South Africa**	**Multiple myeloma**	Deaths	0.31	0.78	1.49	1.54	1.81	0.52
**South Africa**	**Non-Hodgkin lymphoma**	Incidence	0.91	2.04	1.23	3.35	4.02	0.72
**South Africa**	**Non-Hodgkin lymphoma**	Deaths	0.84	1.76	1.09	3.35	3.66	0.46
**South Africa**	**Hodgkin lymphoma**	Incidence	0.13	0.21	0.66	0.4	0.38	-0.43
**South Africa**	**Hodgkin lymphoma**	Deaths	0.09	0.13	0.47	0.32	0.26	-0.85
**South Sudan**	**Leukemia**	Incidence	0.47	0.51	0.1	6.17	5.05	-0.41
**South Sudan**	**Leukemia**	Deaths	0.21	0.25	0.19	3.74	3.34	-0.22
**South Sudan**	**Multiple myeloma**	Incidence	0.02	0.04	0.57	1.05	1.04	0.02
**South Sudan**	**Multiple myeloma**	Deaths	0.02	0.04	0.54	1.04	1.04	0
**South Sudan**	**Non-Hodgkin lymphoma**	Incidence	0.09	0.14	0.62	3.21	3.17	0.07
**South Sudan**	**Non-Hodgkin lymphoma**	Deaths	0.11	0.16	0.45	3.85	3.73	0.03
**South Sudan**	**Hodgkin lymphoma**	Incidence	0.03	0.04	0.62	0.66	0.65	-0.11
**South Sudan**	**Hodgkin lymphoma**	Deaths	0.02	0.04	0.58	0.59	0.58	-0.17
**Spain**	**Leukemia**	Incidence	4.84	10.96	1.27	11.27	16.1	1.08
**Spain**	**Leukemia**	Deaths	2.72	4.19	0.54	5.58	4.42	-0.81
**Spain**	**Multiple myeloma**	Incidence	1.76	3.57	1.02	3.19	3.67	0.14
**Spain**	**Multiple myeloma**	Deaths	1.15	2.37	1.06	2.07	2.27	0
**Spain**	**Non-Hodgkin lymphoma**	Incidence	3.96	7.89	0.99	8.02	8.87	-0.12

Countries	Causes	Measure	Cases in 1990 No.×10³	Cases in 2019 No.×10³	Change in absolute number (100%)	ASR in 1990 per 100000	ASR in 2019 per 100000	EAPC from 1990 and 2019
**Spain**	**Non-Hodgkin lymphoma**	Deaths	2.01	3.28	0.63	3.9	3.36	-0.99
**Spain**	**Hodgkin lymphoma**	Incidence	1.15	1.8	0.56	2.68	3.11	0.55
**Spain**	**Hodgkin lymphoma**	Deaths	0.31	0.24	-0.23	0.66	0.31	-2.6
**Sri Lanka**	**Leukemia**	Incidence	1.04	1.3	0.25	6.88	5.75	-0.74
**Sri Lanka**	**Leukemia**	Deaths	0.66	0.89	0.34	4.95	3.84	-0.91
**Sri Lanka**	**Multiple myeloma**	Incidence	0.11	0.29	1.61	1.05	1.13	0.49
**Sri Lanka**	**Multiple myeloma**	Deaths	0.1	0.23	1.36	0.95	0.91	0.11
**Sri Lanka**	**Non-Hodgkin lymphoma**	Incidence	0.25	1.01	3.04	1.92	4.14	3.2
**Sri Lanka**	**Non-Hodgkin lymphoma**	Deaths	0.21	0.61	1.86	1.83	2.5	1.64
**Sri Lanka**	**Hodgkin lymphoma**	Incidence	0.17	0.28	0.67	1.13	1.19	-0.47
**Sri Lanka**	**Hodgkin lymphoma**	Deaths	0.1	0.08	-0.16	0.74	0.34	-3.21
**Sudan**	**Leukemia**	Incidence	2.26	2.84	0.25	10.35	8.76	-0.38
**Sudan**	**Leukemia**	Deaths	1.1	1.69	0.53	6.94	6.44	-0.1
**Sudan**	**Multiple myeloma**	Incidence	0.1	0.24	1.43	1.05	1.29	0.72
**Sudan**	**Multiple myeloma**	Deaths	0.09	0.2	1.24	1	1.16	0.53
**Sudan**	**Non-Hodgkin lymphoma**	Incidence	0.2	0.62	2.16	1.64	2.32	1.3
**Sudan**	**Non-Hodgkin lymphoma**	Deaths	0.21	0.47	1.26	1.79	2.11	0.72
**Sudan**	**Hodgkin lymphoma**	Incidence	0.12	0.26	1.2	0.85	0.8	-0.14
**Sudan**	**Hodgkin lymphoma**	Deaths	0.09	0.15	0.58	0.74	0.54	-0.94
**Suriname**	**Leukemia**	Incidence	0.02	0.03	0.57	5.28	5.15	-0.12
**Suriname**	**Leukemia**	Deaths	0.01	0.02	0.78	3.71	3.65	-0.13

Countries	Causes	Measure	Cases in 1990 No.×10^3	Cases in 2019 No.×10^3	Change in absolute number (100%)	ASR in 1990 per 100000	ASR in 2019 per 100000	EAPC from 1990 and 2019
**Suriname**	**Multiple myeloma**	Incidence	0	0.01	2.1	1.88	2.49	0.98
**Suriname**	**Multiple myeloma**	Deaths	0	0.01	1.9	1.66	2.04	0.75
**Suriname**	**Non-Hodgkin lymphoma**	Incidence	0.01	0.03	1.17	3.98	4.59	0.48
**Suriname**	**Non-Hodgkin lymphoma**	Deaths	0.01	0.02	1.01	3.83	3.8	-0.03
**Suriname**	**Hodgkin lymphoma**	Incidence	0	0.01	1.09	0.71	0.85	0.59
**Suriname**	**Hodgkin lymphoma**	Deaths	0	0	0.83	0.55	0.54	-0.11
**Sweden**	**Leukemia**	Incidence	1.45	2.26	0.56	12.41	13.62	-0.13
**Sweden**	**Leukemia**	Deaths	0.7	0.93	0.33	5	4.32	-0.45
**Sweden**	**Multiple myeloma**	Incidence	0.55	0.83	0.52	3.6	3.99	0.25
**Sweden**	**Multiple myeloma**	Deaths	0.47	0.66	0.42	2.91	2.92	-0.11
**Sweden**	**Non-Hodgkin lymphoma**	Incidence	1.55	2.13	0.38	10.8	9.96	-0.78
**Sweden**	**Non-Hodgkin lymphoma**	Deaths	0.77	0.9	0.16	5.17	3.98	-1.32
**Sweden**	**Hodgkin lymphoma**	Incidence	0.16	0.22	0.34	1.58	1.88	0.77
**Sweden**	**Hodgkin lymphoma**	Deaths	0.05	0.04	-0.25	0.37	0.21	-1.8
**Switzerland**	**Leukemia**	Incidence	1.45	1.78	0.22	17.6	13.3	-1.25
**Switzerland**	**Leukemia**	Deaths	0.63	0.63	0	6.63	3.69	-1.73
**Switzerland**	**Multiple myeloma**	Incidence	0.26	0.73	1.79	2.53	4.18	1.08
**Switzerland**	**Multiple myeloma**	Deaths	0.18	0.47	1.56	1.71	2.54	0.77
**Switzerland**	**Non-Hodgkin lymphoma**	Incidence	0.45	1.59	2.51	4.69	9.27	1.36
**Switzerland**	**Non-Hodgkin lymphoma**	Deaths	0.22	0.66	2.05	2.14	3.62	0.89
**Switzerland**	**Hodgkin lymphoma**	Incidence	0.12	0.24	0.93	1.58	2.36	0.86

Countries	Causes	Measure	Cases in 1990 No.×10^3	Cases in 2019 No.×10^3	Change in absolute number (100%)	ASR in 1990 per 100000	ASR in 2019 per 100000	EAPC from 1990 and 2019
**Switzerland**	**Hodgkin lymphoma**	Deaths	0.03	0.03	0.26	0.29	0.23	-1
**Syrian Arab Republic**	**Leukemia**	Incidence	2.5	2.66	0.07	26.89	20.88	-1.28
**Syrian Arab Republic**	**Leukemia**	Deaths	1.5	1.85	0.23	21.41	15.82	-1.5
**Syrian Arab Republic**	**Multiple myeloma**	Incidence	0.04	0.1	1.65	0.72	0.8	0.24
**Syrian Arab Republic**	**Multiple myeloma**	Deaths	0.03	0.08	1.37	0.64	0.64	-0.17
**Syrian Arab Republic**	**Non-Hodgkin lymphoma**	Incidence	0.18	0.32	0.76	2.49	2.48	-0.81
**Syrian Arab Republic**	**Non-Hodgkin lymphoma**	Deaths	0.15	0.2	0.29	2.46	1.63	-2.46
**Syrian Arab Republic**	**Hodgkin lymphoma**	Incidence	0.01	0.03	1.19	0.17	0.23	1.36
**Syrian Arab Republic**	**Hodgkin lymphoma**	Deaths	0.01	0.01	0.29	0.13	0.1	-1.09
**Taiwan (Province of China)**	**Leukemia**	Incidence	1.39	2.67	0.92	7.61	9.26	0.8
**Taiwan (Province of China)**	**Leukemia**	Deaths	0.68	1.29	0.9	3.9	3.76	0.02
**Taiwan (Province of China)**	**Multiple myeloma**	Incidence	0.15	0.66	3.39	0.94	1.67	1.97
**Taiwan (Province of China)**	**Multiple myeloma**	Deaths	0.11	0.48	3.21	0.73	1.2	1.68
**Taiwan (Province of China)**	**Non-Hodgkin lymphoma**	Incidence	1.06	3.19	2	6.02	8.85	1.15
**Taiwan (Province of China)**	**Non-Hodgkin lymphoma**	Deaths	0.61	1.5	1.45	3.75	3.95	0.02
**Taiwan (Province of China)**	**Hodgkin lymphoma**	Incidence	0.09	0.3	2.5	0.43	0.97	3.2
**Taiwan (Province of China)**	**Hodgkin lymphoma**	Deaths	0.02	0.03	0.51	0.11	0.09	-0.59

Countries	Causes	Measure	Cases in 1990 No.×10³	Cases in 2019 No.×10³	Change in absolute number (100%)	ASR in 1990 per 100000	ASR in 2019 per 100000	EAPC from 1990 and 2019
**Tajikistan**	**Leukemia**	Incidence	0.51	0.42	-0.18	8.62	5.27	-1.82
**Tajikistan**	**Leukemia**	Deaths	0.26	0.26	0.02	5.23	3.82	-1.16
**Tajikistan**	**Multiple myeloma**	Incidence	0.02	0.04	1.01	0.71	0.84	0.55
**Tajikistan**	**Multiple myeloma**	Deaths	0.02	0.04	0.95	0.66	0.8	0.65
**Tajikistan**	**Non-Hodgkin lymphoma**	Incidence	0.05	0.17	2.17	1.38	2.44	2.14
**Tajikistan**	**Non-Hodgkin lymphoma**	Deaths	0.04	0.12	1.95	1.2	2.04	2.13
**Tajikistan**	**Hodgkin lymphoma**	Incidence	0.01	0.02	1.11	0.3	0.31	-0.07
**Tajikistan**	**Hodgkin lymphoma**	Deaths	0.01	0.02	0.85	0.25	0.24	-0.3
**Thailand**	**Leukemia**	Incidence	4.27	6.08	0.42	8.65	8.04	-0.32
**Thailand**	**Leukemia**	Deaths	2.29	4.14	0.81	5.35	4.69	-0.55
**Thailand**	**Multiple myeloma**	Incidence	0.26	0.79	2.07	0.72	0.76	-0.08
**Thailand**	**Multiple myeloma**	Deaths	0.22	0.62	1.8	0.65	0.61	-0.53
**Thailand**	**Non-Hodgkin lymphoma**	Incidence	0.79	2.93	2.71	1.78	3.2	1.64
**Thailand**	**Non-Hodgkin lymphoma**	Deaths	0.67	1.81	1.68	1.67	1.88	-0.03
**Thailand**	**Hodgkin lymphoma**	Incidence	0.23	0.4	0.72	0.43	0.5	-0.08
**Thailand**	**Hodgkin lymphoma**	Deaths	0.13	0.12	-0.08	0.26	0.13	-2.91
**Timor-Leste**	**Leukemia**	Incidence	0.08	0.08	0.05	9.43	7.01	-1.08
**Timor-Leste**	**Leukemia**	Deaths	0.04	0.05	0.39	6.07	5.04	-0.73
**Timor-Leste**	**Multiple myeloma**	Incidence	0	0.01	2.28	0.68	0.75	0.4
**Timor-Leste**	**Multiple myeloma**	Deaths	0	0.01	2.25	0.65	0.7	0.25
**Timor-Leste**	**Non-Hodgkin lymphoma**	Incidence	0.01	0.02	2.34	1.6	2.31	1.51

Countries	Causes	Measure	Cases in 1990 No.×10^3	Cases in 2019 No.×10^3	Change in absolute number (100%)	ASR in 1990 per 100000	ASR in 2019 per 100000	EAPC from 1990 and 2019
**Timor-Leste**	**Non-Hodgkin lymphoma**	Deaths	0.01	0.02	1.87	1.76	2.36	1.26
**Timor-Leste**	**Hodgkin lymphoma**	Incidence	0	0	0.62	0.46	0.35	-1.03
**Timor-Leste**	**Hodgkin lymphoma**	Deaths	0	0	0.39	0.41	0.27	-1.64
**Togo**	**Leukemia**	Incidence	0.11	0.24	1.11	3.86	4.02	0.24
**Togo**	**Leukemia**	Deaths	0.07	0.16	1.3	3.12	3.23	0.18
**Togo**	**Multiple myeloma**	Incidence	0.01	0.03	2.24	0.78	0.85	0.28
**Togo**	**Multiple myeloma**	Deaths	0.01	0.03	2.15	0.74	0.79	0.22
**Togo**	**Non-Hodgkin lymphoma**	Incidence	0.05	0.14	1.7	2.48	2.58	-0.31
**Togo**	**Non-Hodgkin lymphoma**	Deaths	0.05	0.12	1.52	2.59	2.58	-0.48
**Togo**	**Hodgkin lymphoma**	Incidence	0.01	0.02	1.68	0.31	0.34	0.3
**Togo**	**Hodgkin lymphoma**	Deaths	0.01	0.02	1.62	0.27	0.29	0.18
**Tokelau**	**Leukemia**	Incidence	0	0	-0.34	6.75	5.34	-0.9
**Tokelau**	**Leukemia**	Deaths	0	0	-0.3	5.26	4.09	-0.98
**Tokelau**	**Multiple myeloma**	Incidence	0	0	-0.09	1.26	1.17	-0.35
**Tokelau**	**Multiple myeloma**	Deaths	0	0	-0.15	1.15	1.01	-0.56
**Tokelau**	**Non-Hodgkin lymphoma**	Incidence	0	0	0.36	1.98	2.86	1.33
**Tokelau**	**Non-Hodgkin lymphoma**	Deaths	0	0	0.06	2.04	2.31	0.47
**Tokelau**	**Hodgkin lymphoma**	Incidence	0	0	-0.16	0.39	0.35	-0.45
**Tokelau**	**Hodgkin lymphoma**	Deaths	0	0	-0.43	0.3	0.18	-1.74
**Tonga**	**Leukemia**	Incidence	0	0	0.16	4.04	3.97	-0.15
**Tonga**	**Leukemia**	Deaths	0	0	0.25	3.23	3.09	-0.26

Countries	Causes	Measure	Cases in 1990 No.×10^3	Cases in 2019 No.×10^3	Change in absolute number (100%)	ASR in 1990 per 100000	ASR in 2019 per 100000	EAPC from 1990 and 2019
**Tonga**	**Multiple myeloma**	Incidence	0	0	0.51	1.52	1.63	0.03
**Tonga**	**Multiple myeloma**	Deaths	0	0	0.52	1.38	1.45	-0.05
**Tonga**	**Non-Hodgkin lymphoma**	Incidence	0	0	0.66	4.25	5.36	0.65
**Tonga**	**Non-Hodgkin lymphoma**	Deaths	0	0	0.57	4.37	4.93	0.33
**Tonga**	**Hodgkin lymphoma**	Incidence	0	0	0.28	0.3	0.3	0.09
**Tonga**	**Hodgkin lymphoma**	Deaths	0	0	0.11	0.22	0.19	-0.43
**Trinidad and Tobago**	**Leukemia**	Incidence	0.07	0.08	0.06	6.5	5.02	-0.91
**Trinidad and Tobago**	**Leukemia**	Deaths	0.05	0.06	0.12	4.97	3.61	-1.18
**Trinidad and Tobago**	**Multiple myeloma**	Incidence	0.02	0.06	1.92	2.61	3.36	0.99
**Trinidad and Tobago**	**Multiple myeloma**	Deaths	0.02	0.05	1.7	2.18	2.58	0.68
**Trinidad and Tobago**	**Non-Hodgkin lymphoma**	Incidence	0.05	0.08	0.47	5.19	4.81	-0.38
**Trinidad and Tobago**	**Non-Hodgkin lymphoma**	Deaths	0.04	0.06	0.31	4.5	3.25	-1.4
**Trinidad and Tobago**	**Hodgkin lymphoma**	Incidence	0	0.01	0.92	0.4	0.56	1.68
**Trinidad and Tobago**	**Hodgkin lymphoma**	Deaths	0	0	0.47	0.28	0.26	0.04
**Tunisia**	**Leukemia**	Incidence	0.39	0.52	0.33	5.09	4.38	-0.57
**Tunisia**	**Leukemia**	Deaths	0.23	0.35	0.5	3.66	2.93	-0.8
**Tunisia**	**Multiple myeloma**	Incidence	0.06	0.19	1.93	1.3	1.5	0.46
**Tunisia**	**Multiple myeloma**	Deaths	0.06	0.15	1.63	1.18	1.19	0.03
**Tunisia**	**Non-Hodgkin lymphoma**	Incidence	0.08	0.29	2.59	1.34	2.34	1.92
**Tunisia**	**Non-Hodgkin lymphoma**	Deaths	0.07	0.16	1.47	1.21	1.32	0.27
**Tunisia**	**Hodgkin lymphoma**	Incidence	0.07	0.17	1.53	0.93	1.42	1.46

Countries	Causes	Measure	Cases in 1990 No.×10^3	Cases in 2019 No.×10^3	Change in absolute number (100%)	ASR in 1990 per 100000	ASR in 2019 per 100000	EAPC from 1990 and 2019
**Tunisia**	**Hodgkin lymphoma**	Deaths	0.04	0.05	0.23	0.59	0.38	-1.58
**Turkey**	**Leukemia**	Incidence	6.64	6.88	0.04	12.21	8.45	-1.34
**Turkey**	**Leukemia**	Deaths	4.07	4.4	0.08	8.69	5.28	-1.79
**Turkey**	**Multiple myeloma**	Incidence	0.68	1.77	1.6	1.89	2	0.37
**Turkey**	**Multiple myeloma**	Deaths	0.6	1.35	1.27	1.7	1.55	-0.2
**Turkey**	**Non-Hodgkin lymphoma**	Incidence	1.82	5.09	1.79	4.06	5.92	1.76
**Turkey**	**Non-Hodgkin lymphoma**	Deaths	1.59	2.8	0.76	3.92	3.24	-0.53
**Turkey**	**Hodgkin lymphoma**	Incidence	0.42	0.89	1.13	0.83	1.02	0.77
**Turkey**	**Hodgkin lymphoma**	Deaths	0.29	0.26	-0.1	0.64	0.3	-2.88
**Turkmenistan**	**Leukemia**	Incidence	0.36	0.24	-0.34	8.27	5	-1.9
**Turkmenistan**	**Leukemia**	Deaths	0.16	0.14	-0.09	4.42	3.22	-1.3
**Turkmenistan**	**Multiple myeloma**	Incidence	0.01	0.05	2.89	0.63	1.12	2.52
**Turkmenistan**	**Multiple myeloma**	Deaths	0.01	0.04	2.53	0.56	0.9	2.03
**Turkmenistan**	**Non-Hodgkin lymphoma**	Incidence	0.03	0.1	1.83	1.31	2.23	2.08
**Turkmenistan**	**Non-Hodgkin lymphoma**	Deaths	0.03	0.06	1.32	1.14	1.48	0.85
**Turkmenistan**	**Hodgkin lymphoma**	Incidence	0.02	0.08	2.74	0.64	1.49	4.04
**Turkmenistan**	**Hodgkin lymphoma**	Deaths	0.01	0.03	1.53	0.43	0.63	2.06
**Tuvalu**	**Leukemia**	Incidence	0	0	-0.16	9.07	6.3	-1.32
**Tuvalu**	**Leukemia**	Deaths	0	0	-0.01	6.56	4.91	-1.08
**Tuvalu**	**Multiple myeloma**	Incidence	0	0	0.29	1.42	1.24	-0.64
**Tuvalu**	**Multiple myeloma**	Deaths	0	0	0.25	1.31	1.12	-0.72

Countries	Causes	Measure	Cases in 1990 No.×10^3	Cases in 2019 No.×10^3	Change in absolute number (100%)	ASR in 1990 per 100000	ASR in 2019 per 100000	EAPC from 1990 and 2019
**Tuvalu**	**Non-Hodgkin lymphoma**	Incidence	0	0	0.7	2.2	2.61	0.46
**Tuvalu**	**Non-Hodgkin lymphoma**	Deaths	0	0	0.5	2.31	2.44	0.11
**Tuvalu**	**Hodgkin lymphoma**	Incidence	0	0	0.16	0.5	0.41	-0.62
**Tuvalu**	**Hodgkin lymphoma**	Deaths	0	0	-0.06	0.4	0.27	-1.29
**Uganda**	**Leukemia**	Incidence	0.77	1.42	0.84	3.61	3.54	0.1
**Uganda**	**Leukemia**	Deaths	0.37	0.67	0.83	2.51	2.4	-0.17
**Uganda**	**Multiple myeloma**	Incidence	0.09	0.28	2.05	1.45	2.03	1.17
**Uganda**	**Multiple myeloma**	Deaths	0.09	0.25	1.91	1.43	1.95	1.07
**Uganda**	**Non-Hodgkin lymphoma**	Incidence	0.11	0.49	3.58	1.39	2.32	1.97
**Uganda**	**Non-Hodgkin lymphoma**	Deaths	0.12	0.45	2.74	1.61	2.53	1.79
**Uganda**	**Hodgkin lymphoma**	Incidence	0.1	0.3	2.07	0.82	0.96	0.34
**Uganda**	**Hodgkin lymphoma**	Deaths	0.08	0.21	1.69	0.71	0.75	0.02
**Ukraine**	**Leukemia**	Incidence	6.74	4.96	-0.26	12.24	8.6	-1.96
**Ukraine**	**Leukemia**	Deaths	4.45	2.95	-0.34	7.61	4.78	-2.48
**Ukraine**	**Multiple myeloma**	Incidence	0.7	1.05	0.5	0.96	1.43	1.64
**Ukraine**	**Multiple myeloma**	Deaths	0.52	0.73	0.4	0.72	0.98	1.24
**Ukraine**	**Non-Hodgkin lymphoma**	Incidence	1.52	3.14	1.06	2.6	5.47	3.07
**Ukraine**	**Non-Hodgkin lymphoma**	Deaths	0.92	1.66	0.8	1.46	2.6	2.27
**Ukraine**	**Hodgkin lymphoma**	Incidence	0.82	1.11	0.35	1.48	2.46	1.66
**Ukraine**	**Hodgkin lymphoma**	Deaths	0.44	0.44	-0.01	0.74	0.81	-0.31
**United Arab Emirates**	**Leukemia**	Incidence	0.07	0.43	4.98	9.75	7.69	-1

Countries	Causes	Measure	Cases in 1990 No.×10^3	Cases in 2019 No.×10^3	Change in absolute number (100%)	ASR in 1990 per 100000	ASR in 2019 per 100000	EAPC from 1990 and 2019
**United Arab Emirates**	**Leukemia**	Deaths	0.05	0.31	4.93	8.98	6.56	-1.28
**United Arab Emirates**	**Multiple myeloma**	Incidence	0.01	0.12	9.85	2.22	2.19	-0.2
**United Arab Emirates**	**Multiple myeloma**	Deaths	0.01	0.09	8.63	1.99	1.81	-0.45
**United Arab Emirates**	**Non-Hodgkin lymphoma**	Incidence	0.06	0.51	7.83	6.42	6.91	0.05
**United Arab Emirates**	**Non-Hodgkin lymphoma**	Deaths	0.04	0.31	6.09	6.27	4.81	-1.03
**United Arab Emirates**	**Hodgkin lymphoma**	Incidence	0.01	0.1	7.25	0.86	1.04	0.52
**United Arab Emirates**	**Hodgkin lymphoma**	Deaths	0.01	0.04	4.65	0.62	0.48	-0.93
**United Kingdom**	**Leukemia**	Incidence	8.19	12.65	0.54	11.1	12.55	0.2
**United Kingdom**	**Leukemia**	Deaths	4.7	5.86	0.25	5.6	4.67	-0.76
**United Kingdom**	**Multiple myeloma**	Incidence	3.85	6.6	0.71	4.22	5.17	0.6
**United Kingdom**	**Multiple myeloma**	Deaths	2.63	3.89	0.48	2.82	2.9	-0.04
**United Kingdom**	**Non-Hodgkin lymphoma**	Incidence	8.76	14.01	0.6	10.38	11.44	-0.07
**United Kingdom**	**Non-Hodgkin lymphoma**	Deaths	4.61	6.22	0.35	5.27	4.82	-0.73
**United Kingdom**	**Hodgkin lymphoma**	Incidence	1.91	2.66	0.39	2.92	3.22	0.5
**United Kingdom**	**Hodgkin lymphoma**	Deaths	0.48	0.38	-0.21	0.63	0.36	-1.85
**United Republic of Tanzania**	**Leukemia**	Incidence	1.94	3.49	0.79	5.7	5.69	0.48
**United Republic of Tanzania**	**Leukemia**	Deaths	0.86	1.65	0.91	3.54	3.65	0.45
**United Republic of Tanzania**	**Multiple myeloma**	Incidence	0.14	0.36	1.49	1.34	1.49	0.45
**United Republic of Tanzania**	**Multiple myeloma**	Deaths	0.14	0.33	1.43	1.32	1.43	0.36

Countries	Causes	Measure	Cases in 1990 No.×10^3	Cases in 2019 No.×10^3	Change in absolute number (100%)	ASR in 1990 per 100000	ASR in 2019 per 100000	EAPC from 1990 and 2019
**United Republic of Tanzania**	**Non-Hodgkin lymphoma**	Incidence	0.43	1.21	1.8	3.73	4.49	0.71
**United Republic of Tanzania**	**Non-Hodgkin lymphoma**	Deaths	0.51	1.32	1.61	4.49	5.3	0.66
**United Republic of Tanzania**	**Hodgkin lymphoma**	Incidence	0.16	0.35	1.24	0.79	0.77	0
**United Republic of Tanzania**	**Hodgkin lymphoma**	Deaths	0.12	0.24	1.06	0.67	0.61	-0.23
**United States of America**	**Leukemia**	Incidence	35.2	53.11	0.51	11.7	10.2	-0.7
**United States of America**	**Leukemia**	Deaths	21.45	31.38	0.46	6.86	5.74	-0.74
**United States of America**	**Multiple myeloma**	Incidence	13.81	27.05	0.96	4.3	4.8	0.12
**United States of America**	**Multiple myeloma**	Deaths	10.25	17.94	0.75	3.14	3.11	-0.31
**United States of America**	**Non-Hodgkin lymphoma**	Incidence	41.5	60.84	0.47	13.26	11.08	-1.3
**United States of America**	**Non-Hodgkin lymphoma**	Deaths	21.7	29.99	0.38	6.8	5.28	-1.49
**United States of America**	**Hodgkin lymphoma**	Incidence	9.56	9.88	0.03	3.42	2.56	-1.22
**United States of America**	**Hodgkin lymphoma**	Deaths	1.8	1.53	-0.15	0.61	0.32	-2.24
**United States Virgin Islands**	**Leukemia**	Incidence	0.01	0.01	0.53	6.63	6.32	-0.11
**United States Virgin Islands**	**Leukemia**	Deaths	0	0.01	0.59	4.9	4.37	-0.35

Countries	Causes	Measure	Cases in 1990 No.×10^3	Cases in 2019 No.×10^3	Change in absolute number (100%)	ASR in 1990 per 100000	ASR in 2019 per 100000	EAPC from 1990 and 2019
**United States Virgin Islands**	**Multiple myeloma**	Incidence	0	0.01	2.09	5.08	6.92	1.57
**United States Virgin Islands**	**Multiple myeloma**	Deaths	0	0.01	1.93	4.11	5.2	1.3
**United States Virgin Islands**	**Non-Hodgkin lymphoma**	Incidence	0.01	0.01	1.18	6.06	7.59	0.8
**United States Virgin Islands**	**Non-Hodgkin lymphoma**	Deaths	0	0.01	0.98	4.87	5.07	0.23
**United States Virgin Islands**	**Hodgkin lymphoma**	Incidence	0	0	0.77	0.3	0.39	1
**United States Virgin Islands**	**Hodgkin lymphoma**	Deaths	0	0	0.6	0.19	0.18	-0.02
**Uruguay**	**Leukemia**	Incidence	0.31	0.37	0.2	8.84	7.99	-0.54
**Uruguay**	**Leukemia**	Deaths	0.24	0.28	0.16	6.71	5.51	-0.88
**Uruguay**	**Multiple myeloma**	Incidence	0.11	0.2	0.81	2.78	3.69	0.97
**Uruguay**	**Multiple myeloma**	Deaths	0.09	0.16	0.75	2.38	2.94	0.7
**Uruguay**	**Non-Hodgkin lymphoma**	Incidence	0.25	0.45	0.82	6.85	9.3	0.87
**Uruguay**	**Non-Hodgkin lymphoma**	Deaths	0.19	0.29	0.49	5.22	5.6	0.05
**Uruguay**	**Hodgkin lymphoma**	Incidence	0.05	0.06	0.29	1.38	1.53	0.55
**Uruguay**	**Hodgkin lymphoma**	Deaths	0.03	0.02	-0.18	0.73	0.47	-1.32
**Uzbekistan**	**Leukemia**	Incidence	2.04	1.85	-0.1	9.17	6.3	-1.95
**Uzbekistan**	**Leukemia**	Deaths	0.99	1.1	0.11	5.34	4.24	-1.31
**Uzbekistan**	**Multiple myeloma**	Incidence	0.06	0.17	1.63	0.57	0.82	1.36
**Uzbekistan**	**Multiple myeloma**	Deaths	0.06	0.14	1.46	0.52	0.75	1.33

Countries	Causes	Measure	Cases in 1990 No.×10^3	Cases in 2019 No.×10^3	Change in absolute number (100%)	ASR in 1990 per 100000	ASR in 2019 per 100000	EAPC from 1990 and 2019
**Uzbekistan**	**Non-Hodgkin lymphoma**	Incidence	0.23	0.96	3.09	1.43	3.49	3.79
**Uzbekistan**	**Non-Hodgkin lymphoma**	Deaths	0.16	0.56	2.47	1.14	2.3	3.07
**Uzbekistan**	**Hodgkin lymphoma**	Incidence	0.13	0.32	1.41	0.74	1	1.01
**Uzbekistan**	**Hodgkin lymphoma**	Deaths	0.08	0.14	0.82	0.49	0.5	0.02
**Vanuatu**	**Leukemia**	Incidence	0.01	0.02	1.27	6.13	6.47	-0.04
**Vanuatu**	**Leukemia**	Deaths	0	0.01	1.34	5.08	5.1	-0.22
**Vanuatu**	**Multiple myeloma**	Incidence	0	0	1.76	1.17	1.2	-0.13
**Vanuatu**	**Multiple myeloma**	Deaths	0	0	1.72	1.12	1.13	-0.17
**Vanuatu**	**Non-Hodgkin lymphoma**	Incidence	0	0	2.1	1.74	2.18	0.59
**Vanuatu**	**Non-Hodgkin lymphoma**	Deaths	0	0	2.01	1.87	2.26	0.51
**Vanuatu**	**Hodgkin lymphoma**	Incidence	0	0	1.27	0.43	0.42	-0.37
**Vanuatu**	**Hodgkin lymphoma**	Deaths	0	0	1.18	0.37	0.34	-0.5
**Venezuela (Bolivarian Republic of)**	**Leukemia**	Incidence	1.11	1.63	0.47	6.51	5.84	-0.42
**Venezuela (Bolivarian Republic of)**	**Leukemia**	Deaths	0.75	1.16	0.55	4.93	4.13	-0.73
**Venezuela (Bolivarian Republic of)**	**Multiple myeloma**	Incidence	0.14	0.59	3.22	1.45	2	1.01
**Venezuela (Bolivarian Republic of)**	**Multiple myeloma**	Deaths	0.12	0.46	2.85	1.26	1.58	0.66
**Venezuela (Bolivarian Republic of)**	**Non-Hodgkin lymphoma**	Incidence	0.54	1.35	1.51	3.97	4.66	0.36
**Venezuela (Bolivarian Republic of)**	**Non-Hodgkin lymphoma**	Deaths	0.43	0.87	1.02	3.64	3.02	-0.91

Countries	Causes	Measure	Cases in 1990 No.×10^3	Cases in 2019 No.×10^3	Change in absolute number (100%)	ASR in 1990 per 100000	ASR in 2019 per 100000	EAPC from 1990 and 2019
**Venezuela (Bolivarian Republic of)**	**Hodgkin lymphoma**	Incidence	0.14	0.37	1.73	0.95	1.28	1.08
**Venezuela (Bolivarian Republic of)**	**Hodgkin lymphoma**	Deaths	0.09	0.16	0.78	0.71	0.55	-1.01
**Viet Nam**	**Leukemia**	Incidence	2.69	4.52	0.68	4.86	4.95	0.25
**Viet Nam**	**Leukemia**	Deaths	2.02	3.39	0.68	4.07	3.81	-0.06
**Viet Nam**	**Multiple myeloma**	Incidence	0.26	0.83	2.16	0.66	0.9	1.33
**Viet Nam**	**Multiple myeloma**	Deaths	0.24	0.67	1.79	0.61	0.74	0.92
**Viet Nam**	**Non-Hodgkin lymphoma**	Incidence	0.9	2.84	2.14	1.92	3.05	1.71
**Viet Nam**	**Non-Hodgkin lymphoma**	Deaths	0.89	1.98	1.24	2.03	2.17	0.23
**Viet Nam**	**Hodgkin lymphoma**	Incidence	0.32	0.82	1.59	0.57	0.82	1.57
**Viet Nam**	**Hodgkin lymphoma**	Deaths	0.22	0.29	0.36	0.42	0.3	-0.92
**Yemen**	**Leukemia**	Incidence	1.09	1.89	0.73	8.57	8.32	0.15
**Yemen**	**Leukemia**	Deaths	0.56	1.2	1.16	6.45	6.57	0.22
**Yemen**	**Multiple myeloma**	Incidence	0.05	0.15	1.91	1.02	1.09	0.36
**Yemen**	**Multiple myeloma**	Deaths	0.05	0.13	1.79	0.96	1	0.19
**Yemen**	**Non-Hodgkin lymphoma**	Incidence	0.11	0.34	2.12	1.67	1.85	0.72
**Yemen**	**Non-Hodgkin lymphoma**	Deaths	0.11	0.29	1.64	1.79	1.83	0.38
**Yemen**	**Hodgkin lymphoma**	Incidence	0.06	0.17	1.99	0.74	0.76	0.13
**Yemen**	**Hodgkin lymphoma**	Deaths	0.05	0.11	1.46	0.67	0.59	-0.54
**Zambia**	**Leukemia**	Incidence	0.64	0.82	0.28	6.31	5.08	-0.58
**Zambia**	**Leukemia**	Deaths	0.3	0.44	0.48	4.11	3.63	-0.39

Countries	Causes	Measure	Cases in 1990 No.×10^3	Cases in 2019 No.×10^3	Change in absolute number (100%)	ASR in 1990 per 100000	ASR in 2019 per 100000	EAPC from 1990 and 2019
**Zambia**	**Multiple myeloma**	Incidence	0.04	0.1	1.56	1.41	1.52	0.12
**Zambia**	**Multiple myeloma**	Deaths	0.04	0.09	1.42	1.4	1.44	-0.04
**Zambia**	**Non-Hodgkin lymphoma**	Incidence	0.13	0.37	1.85	4.17	4.78	0.38
**Zambia**	**Non-Hodgkin lymphoma**	Deaths	0.16	0.37	1.34	5.07	5.53	0.25
**Zambia**	**Hodgkin lymphoma**	Incidence	0.05	0.12	1.46	0.87	0.84	-0.18
**Zambia**	**Hodgkin lymphoma**	Deaths	0.04	0.08	1.16	0.75	0.65	-0.56
**Zimbabwe**	**Leukemia**	Incidence	0.28	0.38	0.38	3.89	3.58	-0.05
**Zimbabwe**	**Leukemia**	Deaths	0.18	0.27	0.52	3.07	2.88	0.09
**Zimbabwe**	**Multiple myeloma**	Incidence	0.11	0.27	1.38	2.92	3.93	1.29
**Zimbabwe**	**Multiple myeloma**	Deaths	0.1	0.24	1.36	2.73	3.67	1.45
**Zimbabwe**	**Non-Hodgkin lymphoma**	Incidence	0.11	0.11	0.05	1.96	1.31	-2
**Zimbabwe**	**Non-Hodgkin lymphoma**	Deaths	0.1	0.11	0.1	2.04	1.41	-1.71
**Zimbabwe**	**Hodgkin lymphoma**	Incidence	0.03	0.05	0.96	0.37	0.44	0.72
**Zimbabwe**	**Hodgkin lymphoma**	Deaths	0.02	0.04	1.03	0.3	0.36	1

Abbreviations: ASR, age-standardised rate; EAPC, estimated annual percentage change.

Supplementary Table S8. The percent change in death cases attributable to high body-mass index between 1990 and 2019 at national level.

Countries	Causes	Sex	Percent change in 1990 (95% UI)	Percent change in 2019 (95% UI)
**Afghanistan**	**Leukemia**	Female	3.9 (1.2-8.1)	8.5 (4.1-13.9)
**Afghanistan**	**Leukemia**	Male	1.7 (0.5-3.7)	9.4 (4.8-14.9)
**Afghanistan**	**Multiple myeloma**	Female	4.9 (1.4-10.2)	8 (3.8-13.1)
**Afghanistan**	**Multiple myeloma**	Male	2.7 (0.6-6.6)	2.3 (0.9-4.4)
**Afghanistan**	**Non-Hodgkin lymphoma**	Female	3.4 (0.6-7.6)	7.9 (3.7-13.2)
**Afghanistan**	**Non-Hodgkin lymphoma**	Male	2.3 (0.6-5.3)	9.7 (5-15.2)
**Albania**	**Leukemia**	Female	5.5 (2-11)	8.4 (3.9-14.3)
**Albania**	**Leukemia**	Male	3.4 (1.4-6.4)	7.8 (3.9-12.6)
**Albania**	**Multiple myeloma**	Female	7.2 (2.1-14)	11.5 (6.3-17.6)
**Albania**	**Multiple myeloma**	Male	5.4 (1.6-11.8)	2.2 (0.9-4.3)
**Albania**	**Non-Hodgkin lymphoma**	Female	4.1 (0.7-9)	10.4 (5.2-17)
**Albania**	**Non-Hodgkin lymphoma**	Male	4.5 (1.5-9.1)	8 (3.7-13.5)
**Algeria**	**Leukemia**	Female	6.1 (2.3-11.8)	3.9 (1.8-6.8)
**Algeria**	**Leukemia**	Male	2.7 (1-5.2)	10.8 (5.6-17.1)
**Algeria**	**Multiple myeloma**	Female	7.8 (2.5-14.9)	7.5 (3.6-12.7)
**Algeria**	**Multiple myeloma**	Male	4.7 (1.2-10.4)	9.8 (5-16)
**Algeria**	**Non-Hodgkin lymphoma**	Female	4.4 (0.8-9.1)	8.5 (4.2-13.7)
**Algeria**	**Non-Hodgkin lymphoma**	Male	3.4 (1.1-7.1)	4.3 (1.7-8.1)
**American Samoa**	**Leukemia**	Female	11.7 (4.9-19.3)	2.7 (1-5.5)
**American Samoa**	**Leukemia**	Male	7.9 (4.1-12.4)	1.2 (0.4-2.6)
**American Samoa**	**Multiple myeloma**	Female	12.5 (4.5-22.3)	2.5 (0.9-4.9)
**American Samoa**	**Multiple myeloma**	Male	10.4 (3.2-19.4)	5.8 (2.8-9.7)
**American Samoa**	**Non-Hodgkin lymphoma**	Female	7.9 (1.4-15.1)	1.4 (0.4-3)
**American Samoa**	**Non-Hodgkin lymphoma**	Male	9.9 (3.9-17.1)	1.6 (0.6-3.2)
**Andorra**	**Leukemia**	Female	9.8 (3.7-17.9)	4.2 (1.5-8.1)
**Andorra**	**Leukemia**	Male	6.1 (2.5-10.6)	7.1 (3.3-11.8)
**Andorra**	**Multiple myeloma**	Female	7.8 (2.5-15.1)	5.4 (2.7-8.8)
**Andorra**	**Multiple myeloma**	Male	6.8 (1.9-14.1)	11.8 (6.3-18.4)
**Andorra**	**Non-Hodgkin lymphoma**	Female	5.6 (1-11.8)	11.1 (5.7-17.9)
**Andorra**	**Non-Hodgkin lymphoma**	Male	6.7 (2.3-13.2)	9 (4.4-14.7)
**Angola**	**Leukemia**	Female	0.8 (0.1-2.8)	11.9 (6.2-18.9)
**Angola**	**Leukemia**	Male	0.5 (0.1-1.7)	4 (1.9-7.1)
**Angola**	**Multiple myeloma**	Female	1.5 (0.2-4.2)	2.1 (0.9-4.2)
**Angola**	**Multiple myeloma**	Male	1.2 (0.2-3.8)	7.4 (3.6-12.8)
**Angola**	**Non-Hodgkin lymphoma**	Female	0.8 (0.1-2.6)	9.5 (4.6-15.3)
**Angola**	**Non-Hodgkin lymphoma**	Male	0.9 (0.1-2.7)	6.6 (3.1-11.3)
**Antigua and Barbuda**	**Leukemia**	Female	5.8 (2-11.7)	10.7 (5.6-16.6)
**Antigua and Barbuda**	**Leukemia**	Male	3.1 (1.1-6.1)	8.1 (3.8-13.4)
**Antigua and Barbuda**	**Multiple myeloma**	Female	5.1 (1.4-10.9)	1.8 (0.7-3.5)
**Antigua and Barbuda**	**Multiple myeloma**	Male	3.7 (0.9-8.6)	12 (6.2-19.1)
**Antigua and Barbuda**	**Non-Hodgkin lymphoma**	Female	3.6 (0.6-8.1)	1.7 (0.7-3.5)
**Antigua and Barbuda**	**Non-Hodgkin lymphoma**	Male	3.7 (1.1-8)	8.3 (4.4-13)

Countries	Causes	Sex	Percent change in 1990 (95% UI)	Percent change in 2019 (95% UI)
**Argentina**	**Leukemia**	Female	6.2 (2-12.4)	7.8 (3.8-13.1)
**Argentina**	**Leukemia**	Male	3.5 (1.3-6.7)	7.2 (3.6-11.5)
**Argentina**	**Multiple myeloma**	Female	5.8 (1.7-11.8)	8.9 (4.4-14.6)
**Argentina**	**Multiple myeloma**	Male	4.6 (1.2-10.2)	9.1 (4.4-15.2)
**Argentina**	**Non-Hodgkin lymphoma**	Female	4 (0.7-9.1)	8.5 (3.8-14.4)
**Argentina**	**Non-Hodgkin lymphoma**	Male	4.3 (1.4-9.2)	9.5 (4.8-15.3)
**Armenia**	**Leukemia**	Female	6.9 (2.6-13)	10 (5.1-15.9)
**Armenia**	**Leukemia**	Male	3 (1.2-5.6)	4.7 (2.1-8.5)
**Armenia**	**Multiple myeloma**	Female	7.4 (2.3-14.2)	3 (1.2-5.7)
**Armenia**	**Multiple myeloma**	Male	4.6 (1.3-10.2)	7.2 (3.5-12.3)
**Armenia**	**Non-Hodgkin lymphoma**	Female	4.9 (0.8-10.3)	6.6 (2.8-11.4)
**Armenia**	**Non-Hodgkin lymphoma**	Male	4 (1.3-8.3)	11.6 (6.1-18.3)
**Australia**	**Leukemia**	Female	9.6 (3.6-17.5)	3.6 (1.7-6.4)
**Australia**	**Leukemia**	Male	5.8 (2.6-10)	4.4 (2-7.7)
**Australia**	**Multiple myeloma**	Female	7.5 (2.3-14.4)	9.2 (4.8-14.5)
**Australia**	**Multiple myeloma**	Male	6.5 (1.8-13.5)	8.7 (4.1-14.5)
**Australia**	**Non-Hodgkin lymphoma**	Female	5.5 (1-11.5)	7.7 (3.6-13.1)
**Australia**	**Non-Hodgkin lymphoma**	Male	6.6 (2.4-12.8)	9.7 (4.8-15.6)
**Austria**	**Leukemia**	Female	8.5 (3-16.1)	11.8 (6.5-18)
**Austria**	**Leukemia**	Male	4.9 (2-8.9)	10 (5.1-15.7)
**Austria**	**Multiple myeloma**	Female	6.4 (1.9-12.7)	6.7 (3-11.3)
**Austria**	**Multiple myeloma**	Male	5.4 (1.5-11.8)	12.6 (7.2-18.8)
**Austria**	**Non-Hodgkin lymphoma**	Female	4.7 (0.8-10)	3.2 (1.4-5.9)
**Austria**	**Non-Hodgkin lymphoma**	Male	5.3 (1.8-10.8)	11.9 (6-19.2)
**Azerbaijan**	**Leukemia**	Female	5.2 (2-10.1)	7.1 (3.4-11.9)
**Azerbaijan**	**Leukemia**	Male	2.3 (0.9-4.5)	5.4 (2.7-8.8)
**Azerbaijan**	**Multiple myeloma**	Female	7.6 (2.4-14.9)	11.5 (5.8-18.5)
**Azerbaijan**	**Multiple myeloma**	Male	5.2 (1.4-11.2)	7.9 (3.8-13.2)
**Azerbaijan**	**Non-Hodgkin lymphoma**	Female	5 (0.9-10.3)	2.7 (1.1-4.9)
**Azerbaijan**	**Non-Hodgkin lymphoma**	Male	4.2 (1.4-8.7)	6.4 (3.2-10.5)
**Bahamas**	**Leukemia**	Female	9.2 (3.6-16.5)	2.7 (1.1-5.1)
**Bahamas**	**Leukemia**	Male	4.7 (2.1-8.1)	7.3 (3.3-12.6)
**Bahamas**	**Multiple myeloma**	Female	8.4 (2.8-15.9)	6.9 (3.5-11.4)
**Bahamas**	**Multiple myeloma**	Male	6.3 (1.7-13.1)	7 (3.4-11.7)
**Bahamas**	**Non-Hodgkin lymphoma**	Female	5.8 (1.1-11.8)	8.9 (4.5-14.6)
**Bahamas**	**Non-Hodgkin lymphoma**	Male	5.9 (2.1-11.5)	10.2 (4.8-16.9)
**Bahrain**	**Leukemia**	Female	10.6 (4.3-18.4)	11.7 (6.2-18.2)
**Bahrain**	**Leukemia**	Male	6 (2.8-9.9)	9 (4.4-14.6)
**Bahrain**	**Multiple myeloma**	Female	11.1 (3.9-20.4)	3.3 (1.3-6.2)
**Bahrain**	**Multiple myeloma**	Male	8 (2.3-16)	6.4 (3.3-10.4)
**Bahrain**	**Non-Hodgkin lymphoma**	Female	7.3 (1.3-14.5)	3.5 (1.4-6.9)
**Bahrain**	**Non-Hodgkin lymphoma**	Male	7.1 (2.6-13.2)	7.7 (3.5-13.2)
**Bangladesh**	**Leukemia**	Female	0.5 (0.1-1.7)	6.1 (2.8-10.4)
**Bangladesh**	**Leukemia**	Male	0.4 (0.1-1.1)	1.8 (0.6-3.6)

Countries	Causes	Sex	Percent change in 1990 (95% UI)	Percent change in 2019 (95% UI)
**Bangladesh**	**Multiple myeloma**	Female	0.8 (0.1-2.5)	9.6 (4.9-15.3)
**Bangladesh**	**Multiple myeloma**	Male	0.7 (0.1-2.3)	10.4 (5.5-16.5)
**Bangladesh**	**Non-Hodgkin lymphoma**	Female	0.5 (0-1.5)	6.9 (3.2-11.9)
**Bangladesh**	**Non-Hodgkin lymphoma**	Male	0.5 (0.1-1.7)	10.4 (5.7-15.8)
**Barbados**	**Leukemia**	Female	10 (3.8-18.3)	11.1 (5.8-17.3)
**Barbados**	**Leukemia**	Male	4.8 (2-8.5)	7.4 (3.6-12.1)
**Barbados**	**Multiple myeloma**	Female	8.4 (2.6-16.7)	2.8 (1-5.6)
**Barbados**	**Multiple myeloma**	Male	5.7 (1.6-12.1)	7.3 (3.5-12.1)
**Barbados**	**Non-Hodgkin lymphoma**	Female	6.1 (1.1-12.8)	3.6 (1.6-6.5)
**Barbados**	**Non-Hodgkin lymphoma**	Male	5.6 (2-11.2)	11.6 (6-18.4)
**Belarus**	**Leukemia**	Female	9.2 (3.6-16.9)	11.6 (6.1-18.1)
**Belarus**	**Leukemia**	Male	3.9 (1.7-7.1)	13.5 (7.9-19.5)
**Belarus**	**Multiple myeloma**	Female	8.6 (2.9-16.4)	10.1 (4.9-16.1)
**Belarus**	**Multiple myeloma**	Male	5.5 (1.5-11.6)	10.7 (5.9-16.6)
**Belarus**	**Non-Hodgkin lymphoma**	Female	5.8 (1-12.1)	2.5 (0.9-4.9)
**Belarus**	**Non-Hodgkin lymphoma**	Male	4.7 (1.6-9.4)	9.2 (4.5-14.7)
**Belgium**	**Leukemia**	Female	8.1 (2.9-15.3)	8.3 (4-13.8)
**Belgium**	**Leukemia**	Male	4.5 (1.9-8.2)	9.3 (4.6-15)
**Belgium**	**Multiple myeloma**	Female	6.1 (1.8-12)	6.5 (3.1-10.7)
**Belgium**	**Multiple myeloma**	Male	4.9 (1.4-10.6)	13.4 (7.6-20)
**Belgium**	**Non-Hodgkin lymphoma**	Female	4.5 (0.8-9.7)	11.2 (5.9-17.7)
**Belgium**	**Non-Hodgkin lymphoma**	Male	5 (1.7-10)	6.7 (3.2-11.3)
**Belize**	**Leukemia**	Female	4.7 (1.7-9.2)	5.4 (2.5-9.6)
**Belize**	**Leukemia**	Male	2.2 (0.7-4.3)	11.4 (6-17.8)
**Belize**	**Multiple myeloma**	Female	7 (2.1-13.9)	5.7 (2.4-10.2)
**Belize**	**Multiple myeloma**	Male	4.1 (0.9-9.9)	0.7 (0.1-1.7)
**Belize**	**Non-Hodgkin lymphoma**	Female	4 (0.7-8.7)	2.2 (0.9-4.1)
**Belize**	**Non-Hodgkin lymphoma**	Male	3.2 (0.9-7)	9.1 (4.4-15.1)
**Benin**	**Leukemia**	Female	3.2 (1-6.9)	5.9 (2.9-9.7)
**Benin**	**Leukemia**	Male	1 (0.3-2.4)	8 (3.8-13.1)
**Benin**	**Multiple myeloma**	Female	4.2 (1.1-8.9)	7.5 (3.5-12.6)
**Benin**	**Multiple myeloma**	Male	2.3 (0.5-5.8)	9.7 (4.8-15.8)
**Benin**	**Non-Hodgkin lymphoma**	Female	2.4 (0.4-5.5)	3.2 (1.3-6)
**Benin**	**Non-Hodgkin lymphoma**	Male	1.5 (0.4-3.8)	5.8 (2.6-10.3)
**Bermuda**	**Leukemia**	Female	12 (4.8-21.3)	4 (1.7-7.1)
**Bermuda**	**Leukemia**	Male	6.9 (3.2-11.6)	8.6 (4.1-14.4)
**Bermuda**	**Multiple myeloma**	Female	9.6 (3.2-18.3)	10 (5.1-15.8)
**Bermuda**	**Multiple myeloma**	Male	7.8 (2.2-15.8)	9.8 (4.8-15.9)
**Bermuda**	**Non-Hodgkin lymphoma**	Female	7 (1.2-14.3)	7.8 (4-12.6)
**Bermuda**	**Non-Hodgkin lymphoma**	Male	7.8 (2.9-15)	7.2 (3.2-12.3)
**Bhutan**	**Leukemia**	Female	2 (0.4-5.4)	10.5 (5.3-16.9)
**Bhutan**	**Leukemia**	Male	0.9 (0.2-2.5)	15.1 (9.1-21.9)
**Bhutan**	**Multiple myeloma**	Female	2.8 (0.6-6.9)	2.4 (1.1-4.3)
**Bhutan**	**Multiple myeloma**	Male	1.7 (0.3-4.9)	11.2 (5.8-17.3)

Countries	Causes	Sex	Percent change in 1990 (95% UI)	Percent change in 2019 (95% UI)
**Bhutan**	**Non-Hodgkin lymphoma**	Female	1.8 (0.2-4.5)	8.3 (3.8-14.2)
**Bhutan**	**Non-Hodgkin lymphoma**	Male	1.3 (0.2-3.7)	7.1 (3.5-11.5)
**Bolivia (Plurinational State of)**	**Leukemia**	Female	4.1 (1.4-8.1)	7.2 (3.4-12.2)
**Bolivia (Plurinational State of)**	**Leukemia**	Male	1.9 (0.7-3.8)	2.7 (1-5.2)
**Bolivia (Plurinational State of)**	**Multiple myeloma**	Female	6 (1.8-11.7)	2.9 (1.2-5.4)
**Bolivia (Plurinational State of)**	**Multiple myeloma**	Male	4.3 (1.1-9.7)	4.7 (2.1-8.2)
**Bolivia (Plurinational State of)**	**Non-Hodgkin lymphoma**	Female	3.7 (0.6-8.1)	10.8 (4.3-19.7)
**Bolivia (Plurinational State of)**	**Non-Hodgkin lymphoma**	Male	3.4 (1.1-7.5)	12.8 (5.2-22.2)
**Bosnia and Herzegovina**	**Leukemia**	Female	8.5 (3.2-15.9)	11.4 (4.5-20.6)
**Bosnia and Herzegovina**	**Leukemia**	Male	4.1 (1.6-7.5)	3.2 (1.1-6.5)
**Bosnia and Herzegovina**	**Multiple myeloma**	Female	7.4 (2.4-14.2)	10.3 (4-18.9)
**Bosnia and Herzegovina**	**Multiple myeloma**	Male	5.2 (1.4-11.2)	13.1 (5.3-23)
**Bosnia and Herzegovina**	**Non-Hodgkin lymphoma**	Female	4.9 (0.8-10.5)	10.8 (4.2-19.7)
**Bosnia and Herzegovina**	**Non-Hodgkin lymphoma**	Male	4.6 (1.6-9.4)	10.9 (4.5-19.1)
**Botswana**	**Leukemia**	Female	5.8 (2.1-11.3)	15.9 (6.6-26.5)
**Botswana**	**Leukemia**	Male	1.4 (0.4-3.1)	2.9 (0.9-6)
**Botswana**	**Multiple myeloma**	Female	5.9 (1.9-11.7)	13.6 (5.7-23.6)
**Botswana**	**Multiple myeloma**	Male	2 (0.4-5.2)	10.3 (4-18.9)
**Botswana**	**Non-Hodgkin lymphoma**	Female	3.7 (0.6-8.2)	6.2 (2.5-11.3)
**Botswana**	**Non-Hodgkin lymphoma**	Male	1.7 (0.4-4.3)	14.5 (5.9-24.9)
**Brazil**	**Leukemia**	Female	5.1 (1.8-9.9)	10 (4-17.8)
**Brazil**	**Leukemia**	Male	2.9 (1.1-5.4)	12.7 (5.2-22.1)
**Brazil**	**Multiple myeloma**	Female	6.4 (1.9-12.6)	10.9 (4.4-19.1)
**Brazil**	**Multiple myeloma**	Male	4.9 (1.3-10.8)	5.1 (1.7-10.4)
**Brazil**	**Non-Hodgkin lymphoma**	Female	3.9 (0.7-8.5)	4.8 (1.7-9.7)
**Brazil**	**Non-Hodgkin lymphoma**	Male	4 (1.3-8.4)	1.7 (0.5-4)
**Brunei Darussalam**	**Leukemia**	Female	2.5 (0.5-6.1)	3.4 (1-7.2)
**Brunei Darussalam**	**Leukemia**	Male	1.4 (0.3-3.3)	8.6 (3.4-15.4)
**Brunei Darussalam**	**Multiple myeloma**	Female	2.8 (0.6-6.9)	2.1 (0.6-4.9)
**Brunei Darussalam**	**Multiple myeloma**	Male	2.2 (0.4-6.2)	2.7 (0.9-5.4)
**Brunei Darussalam**	**Non-Hodgkin lymphoma**	Female	1.8 (0.3-4.7)	5.4 (1.6-11.5)
**Brunei Darussalam**	**Non-Hodgkin lymphoma**	Male	1.9 (0.4-5)	9.4 (3.7-17.2)
**Bulgaria**	**Leukemia**	Female	11.5 (4.6-20.4)	7.8 (3.2-13.5)
**Bulgaria**	**Leukemia**	Male	6.8 (3.2-11.1)	14.6 (6-25)
**Bulgaria**	**Multiple myeloma**	Female	9.9 (3.3-18.6)	13.7 (5.5-23.8)
**Bulgaria**	**Multiple myeloma**	Male	8.4 (2.3-16.5)	12.4 (4.9-21.8)
**Bulgaria**	**Non-Hodgkin lymphoma**	Female	6.8 (1.2-13.8)	14.8 (5.9-25.7)
**Bulgaria**	**Non-Hodgkin lymphoma**	Male	7.7 (2.8-14.6)	6.1 (2.3-11.3)
**Burkina Faso**	**Leukemia**	Female	2 (0.4-5.1)	2.8 (0.9-5.7)
**Burkina Faso**	**Leukemia**	Male	0.6 (0.1-1.7)	9.7 (3.7-17.6)
**Burkina Faso**	**Multiple myeloma**	Female	2.5 (0.5-5.9)	12.3 (4.9-21.5)
**Burkina Faso**	**Multiple myeloma**	Male	1.3 (0.2-3.9)	8.9 (3.5-16)
**Burkina Faso**	**Non-Hodgkin lymphoma**	Female	1.4 (0.2-3.7)	15.3 (6.5-25.7)
**Burkina Faso**	**Non-Hodgkin lymphoma**	Male	0.9 (0.2-2.7)	10.9 (4.3-19.5)

Countries	Causes	Sex	Percent change in 1990 (95% UI)	Percent change in 2019 (95% UI)
**Burundi**	**Leukemia**	Female	1 (0.2-2.4)	2.9 (1-6)
**Burundi**	**Leukemia**	Male	0.5 (0.1-1.1)	15.2 (6.2-25.8)
**Burundi**	**Multiple myeloma**	Female	2 (0.4-4.7)	2.4 (0.8-5.1)
**Burundi**	**Multiple myeloma**	Male	1.2 (0.2-3.5)	10.8 (4.4-18.6)
**Burundi**	**Non-Hodgkin lymphoma**	Female	1.2 (0.2-3.1)	10.1 (3.9-18.1)
**Burundi**	**Non-Hodgkin lymphoma**	Male	0.9 (0.2-2.5)	10.9 (4.4-19)
**Cabo Verde**	**Leukemia**	Female	3.5 (1.1-7.4)	11.6 (4.8-20.2)
**Cabo Verde**	**Leukemia**	Male	1.3 (0.3-2.8)	11.6 (4.6-20.8)
**Cabo Verde**	**Multiple myeloma**	Female	4.2 (1.1-8.9)	11.1 (4.3-20.8)
**Cabo Verde**	**Multiple myeloma**	Male	2.3 (0.5-5.7)	12.8 (5-22.5)
**Cabo Verde**	**Non-Hodgkin lymphoma**	Female	2.6 (0.4-6.2)	12 (4.8-21)
**Cabo Verde**	**Non-Hodgkin lymphoma**	Male	1.9 (0.4-4.4)	6.4 (2.4-12.3)
**Cambodia**	**Leukemia**	Female	1 (0.2-2.8)	4.9 (1.8-9.7)
**Cambodia**	**Leukemia**	Male	0.4 (0.1-1.2)	10 (3.9-18)
**Cambodia**	**Multiple myeloma**	Female	1.4 (0.3-3.8)	8.5 (3.2-15.8)
**Cambodia**	**Multiple myeloma**	Male	0.8 (0.1-2.8)	14.2 (5.8-24.6)
**Cambodia**	**Non-Hodgkin lymphoma**	Female	0.8 (0.1-2.4)	4.8 (1.9-9.1)
**Cambodia**	**Non-Hodgkin lymphoma**	Male	0.6 (0.1-2)	6.3 (2.4-11.8)
**Cameroon**	**Leukemia**	Female	7.5 (2.9-13.9)	11.6 (4.6-20.3)
**Cameroon**	**Leukemia**	Male	2.4 (0.9-4.8)	11.1 (4.3-20.1)
**Cameroon**	**Multiple myeloma**	Female	8 (2.6-15.5)	10 (3.8-18.3)
**Cameroon**	**Multiple myeloma**	Male	4.3 (1.1-9.8)	13.1 (5.3-22.7)
**Cameroon**	**Non-Hodgkin lymphoma**	Female	4.7 (0.8-9.8)	15.1 (6.3-25.1)
**Cameroon**	**Non-Hodgkin lymphoma**	Male	2.9 (0.8-6.7)	13.6 (5.5-23)
**Canada**	**Leukemia**	Female	9.5 (3.6-17.4)	8.6 (3.2-15.6)
**Canada**	**Leukemia**	Male	5.5 (2.3-9.8)	17.1 (7.4-27.6)
**Canada**	**Multiple myeloma**	Female	7.3 (2.4-14.1)	4.4 (1.5-8.4)
**Canada**	**Multiple myeloma**	Male	6.1 (1.7-12.8)	15.2 (6.2-25.9)
**Canada**	**Non-Hodgkin lymphoma**	Female	5.4 (0.9-11)	12 (4.8-20.6)
**Canada**	**Non-Hodgkin lymphoma**	Male	6.2 (2.2-12.1)	7.8 (3.1-13.9)
**Central African Republic**	**Leukemia**	Female	1.3 (0.3-3.5)	14.5 (5.8-25)
**Central African Republic**	**Leukemia**	Male	0.6 (0.1-1.7)	10.2 (4-18.7)
**Central African Republic**	**Multiple myeloma**	Female	2.1 (0.5-4.9)	4 (1.3-7.8)
**Central African Republic**	**Multiple myeloma**	Male	1.3 (0.2-3.7)	8.4 (3.4-15.3)
**Central African Republic**	**Non-Hodgkin lymphoma**	Female	1.2 (0.2-3.2)	4.7 (1.6-9.3)
**Central African Republic**	**Non-Hodgkin lymphoma**	Male	1 (0.2-2.7)	10 (3.7-18.6)
**Chad**	**Leukemia**	Female	1.6 (0.4-3.8)	9.7 (4-17.1)
**Chad**	**Leukemia**	Male	0.5 (0.1-1.3)	9.4 (3.7-17)
**Chad**	**Multiple myeloma**	Female	1.9 (0.4-4.5)	11.8 (4.8-20.6)
**Chad**	**Multiple myeloma**	Male	1 (0.1-3)	12.9 (5-23.2)
**Chad**	**Non-Hodgkin lymphoma**	Female	1.1 (0.2-2.8)	14.4 (5.8-24.7)
**Chad**	**Non-Hodgkin lymphoma**	Male	0.7 (0.1-2)	12.7 (5.1-21.7)
**Chile**	**Leukemia**	Female	7.5 (2.8-14.3)	4.7 (1.7-9.2)
**Chile**	**Leukemia**	Male	4.1 (1.6-7.4)	9.6 (3.9-16.8)

Countries	Causes	Sex	Percent change in 1990 (95% UI)	Percent change in 2019 (95% UI)
**Chile**	**Multiple myeloma**	Female	7.5 (2.3-14.5)	4.7 (1.5-9.6)
**Chile**	**Multiple myeloma**	Male	6.2 (1.6-13.1)	10.1 (3.9-19.1)
**Chile**	**Non-Hodgkin lymphoma**	Female	5.2 (0.9-11)	8.5 (3.2-15.4)
**Chile**	**Non-Hodgkin lymphoma**	Male	5.6 (1.9-11.4)	3.1 (1-6.6)
**China**	**Leukemia**	Female	1.6 (0.3-4.4)	11.9 (4.8-21.1)
**China**	**Leukemia**	Male	0.9 (0.2-2.3)	13.3 (5.4-23)
**China**	**Multiple myeloma**	Female	2.1 (0.4-5.5)	9 (3.4-16.9)
**China**	**Multiple myeloma**	Male	1.7 (0.2-4.8)	13.8 (5.7-23.1)
**China**	**Non-Hodgkin lymphoma**	Female	1.3 (0.2-3.6)	13.9 (5.6-24.1)
**China**	**Non-Hodgkin lymphoma**	Male	1.4 (0.2-3.9)	10.2 (4-18.2)
**Colombia**	**Leukemia**	Female	4.6 (1.6-9.1)	3.6 (1.1-7.6)
**Colombia**	**Leukemia**	Male	2.3 (0.8-4.4)	9.2 (3.5-16.7)
**Colombia**	**Multiple myeloma**	Female	5.9 (1.8-12.2)	4.7 (1.8-9.1)
**Colombia**	**Multiple myeloma**	Male	4.2 (1.1-9.7)	14.6 (5.8-25.2)
**Colombia**	**Non-Hodgkin lymphoma**	Female	3.7 (0.6-8.1)	14.8 (6.1-25.4)
**Colombia**	**Non-Hodgkin lymphoma**	Male	3.5 (1.1-7.3)	17.6 (7.6-28.2)
**Comoros**	**Leukemia**	Female	2 (0.5-4.4)	13.5 (5.5-23.3)
**Comoros**	**Leukemia**	Male	1 (0.3-2.1)	13.5 (5.6-23.4)
**Comoros**	**Multiple myeloma**	Female	3.4 (0.9-7.6)	4 (1.3-8.3)
**Comoros**	**Multiple myeloma**	Male	2.1 (0.4-5.4)	12.1 (4.9-21.1)
**Comoros**	**Non-Hodgkin lymphoma**	Female	2.2 (0.3-5.3)	11.1 (4.4-19.4)
**Comoros**	**Non-Hodgkin lymphoma**	Male	1.8 (0.4-4.2)	11.4 (4.5-20.4)
**Congo**	**Leukemia**	Female	3.5 (1.1-7.4)	8.9 (3.5-16.2)
**Congo**	**Leukemia**	Male	1.6 (0.5-3.6)	17.9 (7.7-29.2)
**Congo**	**Multiple myeloma**	Female	4.6 (1.3-9.5)	14 (5.6-24.1)
**Congo**	**Multiple myeloma**	Male	3.1 (0.7-7.4)	9.1 (3.5-16.5)
**Congo**	**Non-Hodgkin lymphoma**	Female	2.8 (0.5-6.3)	6.9 (2.5-13.3)
**Congo**	**Non-Hodgkin lymphoma**	Male	2.4 (0.6-5.7)	14.3 (5.9-24.2)
**Cook Islands**	**Leukemia**	Female	11 (4.3-19.5)	7.4 (2.7-14.2)
**Cook Islands**	**Leukemia**	Male	6.6 (3-10.7)	1.1 (0.2-2.7)
**Cook Islands**	**Multiple myeloma**	Female	9.5 (3.2-17.8)	3 (1-6)
**Cook Islands**	**Multiple myeloma**	Male	8 (2.2-15.9)	11.9 (4.6-21.4)
**Cook Islands**	**Non-Hodgkin lymphoma**	Female	6.4 (1.2-12.8)	7.9 (3.1-14.3)
**Cook Islands**	**Non-Hodgkin lymphoma**	Male	7.7 (2.8-14.4)	10.6 (4.2-18.7)
**Costa Rica**	**Leukemia**	Female	6.4 (2.3-12.3)	9.6 (3.7-17.7)
**Costa Rica**	**Leukemia**	Male	3.6 (1.5-6.5)	13.4 (5.4-23.3)
**Costa Rica**	**Multiple myeloma**	Female	6.7 (2-13.3)	4.9 (1.7-9.6)
**Costa Rica**	**Multiple myeloma**	Male	5.4 (1.5-11.8)	7.7 (2.9-14.7)
**Costa Rica**	**Non-Hodgkin lymphoma**	Female	4.7 (0.8-10)	6.5 (2.4-11.9)
**Costa Rica**	**Non-Hodgkin lymphoma**	Male	5 (1.7-10)	10.5 (4.1-19.2)
**Croatia**	**Leukemia**	Female	10.8 (4.1-19.8)	13.4 (5.7-22.8)
**Croatia**	**Leukemia**	Male	6.4 (2.9-10.8)	12.9 (5.2-22.4)
**Croatia**	**Multiple myeloma**	Female	8.6 (2.8-16.5)	10.4 (4.2-18.2)
**Croatia**	**Multiple myeloma**	Male	7.2 (2-14.6)	9.1 (3.4-17)

Countries	Causes	Sex	Percent change in 1990 (95% UI)	Percent change in 2019 (95% UI)
**Croatia**	**Non-Hodgkin lymphoma**	Female	6 (1.1-12.5)	14.4 (5.9-24.6)
**Croatia**	**Non-Hodgkin lymphoma**	Male	7.1 (2.5-13.5)	20.2 (8.8-32.5)
**Cuba**	**Leukemia**	Female	8 (3.1-14.7)	3.5 (1.3-6.6)
**Cuba**	**Leukemia**	Male	3.6 (1.5-6.3)	14.4 (5.9-24.4)
**Cuba**	**Multiple myeloma**	Female	7 (2.2-13.5)	11 (4.4-19.6)
**Cuba**	**Multiple myeloma**	Male	4.4 (1.2-9.5)	9.7 (3.9-17.1)
**Cuba**	**Non-Hodgkin lymphoma**	Female	4.9 (0.9-10.2)	9.4 (3.6-17.2)
**Cuba**	**Non-Hodgkin lymphoma**	Male	4.3 (1.5-8.5)	3.6 (1.1-7.6)
**Cyprus**	**Leukemia**	Female	6.2 (2.1-12.4)	4.1 (1.4-8.2)
**Cyprus**	**Leukemia**	Male	3.6 (1.3-6.7)	7.8 (3-14.1)
**Cyprus**	**Multiple myeloma**	Female	4.9 (1.4-10)	6.7 (3-11.1)
**Cyprus**	**Multiple myeloma**	Male	4.1 (1.1-9.1)	6.9 (3.3-11.3)
**Cyprus**	**Non-Hodgkin lymphoma**	Female	3.6 (0.6-8.2)	7 (3.1-11.9)
**Cyprus**	**Non-Hodgkin lymphoma**	Male	4.1 (1.3-8.5)	1.6 (0.6-3.3)
**Czechia**	**Leukemia**	Female	11 (4.3-19.8)	5.8 (2.6-10)
**Czechia**	**Leukemia**	Male	7 (3.3-11.5)	7.1 (3.5-11.4)
**Czechia**	**Multiple myeloma**	Female	8.7 (2.8-16.4)	6.5 (2.8-11.1)
**Czechia**	**Multiple myeloma**	Male	7.9 (2.3-15.6)	5.5 (2.5-9.3)
**Czechia**	**Non-Hodgkin lymphoma**	Female	6.2 (1.1-12.9)	9.7 (4.9-15)
**Czechia**	**Non-Hodgkin lymphoma**	Male	7.6 (2.7-14.2)	1.8 (0.7-3.5)
**Côte d'Ivoire**	**Leukemia**	Female	3.7 (1.2-7.3)	7.3 (3.5-12)
**Côte d'Ivoire**	**Leukemia**	Male	1.5 (0.5-3.1)	6 (2.6-10.4)
**Côte d'Ivoire**	**Multiple myeloma**	Female	4.7 (1.3-9.7)	2.4 (1-4.5)
**Côte d'Ivoire**	**Multiple myeloma**	Male	3.2 (0.8-7.3)	8.7 (4-14)
**Côte d'Ivoire**	**Non-Hodgkin lymphoma**	Female	2.8 (0.5-6.1)	5 (2.3-8.6)
**Côte d'Ivoire**	**Non-Hodgkin lymphoma**	Male	2.3 (0.7-5)	7.7 (3.7-12.6)
**Democratic People's Republic of Korea**	**Leukemia**	Female	1.4 (0.2-4.3)	6.5 (3.1-10.6)
**Democratic People's Republic of Korea**	**Leukemia**	Male	0.8 (0.1-2.2)	3.4 (1.3-6.5)
**Democratic People's Republic of Korea**	**Multiple myeloma**	Female	1.7 (0.3-5)	1.3 (0.4-2.8)
**Democratic People's Republic of Korea**	**Multiple myeloma**	Male	1.4 (0.2-4.3)	0.9 (0.2-2)
**Democratic People's Republic of Korea**	**Non-Hodgkin lymphoma**	Female	1.1 (0.1-3.3)	1.6 (0.5-3.2)
**Democratic People's Republic of Korea**	**Non-Hodgkin lymphoma**	Male	1.3 (0.2-3.6)	4 (1.8-6.7)
**Democratic Republic of the Congo**	**Leukemia**	Female	2 (0.5-5.2)	0.9 (0.2-2.1)
**Democratic Republic of the Congo**	**Leukemia**	Male	1.4 (0.4-3.4)	0.9 (0.3-2.1)
**Democratic Republic of the Congo**	**Multiple myeloma**	Female	3.5 (1-7.4)	3.3 (1.2-6.5)

Countries	Causes	Sex	Percent change in 1990 (95% UI)	Percent change in 2019 (95% UI)
**Democratic Republic of the Congo**	**Multiple myeloma**	Male	3.3 (0.9-7.4)	5 (2.3-8.5)
**Democratic Republic of the Congo**	**Non-Hodgkin lymphoma**	Female	1.9 (0.3-4.6)	3.7 (1.6-6.4)
**Democratic Republic of the Congo**	**Non-Hodgkin lymphoma**	Male	2.2 (0.6-5)	9.7 (4.8-15.1)
**Denmark**	**Leukemia**	Female	7.2 (2.6-14.1)	8.9 (4.3-14.4)
**Denmark**	**Leukemia**	Male	4.5 (1.8-8.3)	6.2 (2.9-10.6)
**Denmark**	**Multiple myeloma**	Female	5.5 (1.6-11.1)	9.7 (4.8-15.2)
**Denmark**	**Multiple myeloma**	Male	4.9 (1.4-10.6)	2.8 (1.2-5.1)
**Denmark**	**Non-Hodgkin lymphoma**	Female	4 (0.7-8.8)	1.8 (0.7-3.4)
**Denmark**	**Non-Hodgkin lymphoma**	Male	4.9 (1.7-10.1)	5.8 (2.5-10.2)
**Djibouti**	**Leukemia**	Female	0.6 (0.1-1.9)	6.8 (3.2-11.2)
**Djibouti**	**Leukemia**	Male	0.5 (0.1-1.3)	5 (2.2-8.8)
**Djibouti**	**Multiple myeloma**	Female	1.7 (0.3-4.5)	7.7 (3.7-12.4)
**Djibouti**	**Multiple myeloma**	Male	1.2 (0.2-3.8)	5.4 (2.4-9.3)
**Djibouti**	**Non-Hodgkin lymphoma**	Female	1 (0.1-2.8)	1 (0.3-2.1)
**Djibouti**	**Non-Hodgkin lymphoma**	Male	0.9 (0.2-2.7)	8.6 (4.3-13.5)
**Dominica**	**Leukemia**	Female	8.3 (3.1-15.3)	1.3 (0.4-2.7)
**Dominica**	**Leukemia**	Male	3.9 (1.6-7)	6.4 (3.2-10.2)
**Dominica**	**Multiple myeloma**	Female	7.3 (2.3-14.2)	6 (2.7-10.5)
**Dominica**	**Multiple myeloma**	Male	5.1 (1.4-10.9)	5 (2.4-8.3)
**Dominica**	**Non-Hodgkin lymphoma**	Female	5.2 (0.9-10.7)	6.7 (3.1-11.3)
**Dominica**	**Non-Hodgkin lymphoma**	Male	4.8 (1.6-9.7)	7.1 (3.1-12)
**Dominican Republic**	**Leukemia**	Female	3.2 (1-6.9)	6.4 (2.7-11.2)
**Dominican Republic**	**Leukemia**	Male	1.8 (0.5-3.8)	7.9 (3.7-13)
**Dominican Republic**	**Multiple myeloma**	Female	4.8 (1.3-10.1)	8.3 (4.1-13.3)
**Dominican Republic**	**Multiple myeloma**	Male	3.5 (0.8-8.5)	3.2 (1.3-5.9)
**Dominican Republic**	**Non-Hodgkin lymphoma**	Female	2.6 (0.4-6.2)	1.7 (0.6-3.5)
**Dominican Republic**	**Non-Hodgkin lymphoma**	Male	2.8 (0.8-6.2)	5 (2.2-8.6)
**Ecuador**	**Leukemia**	Female	6.9 (2.7-12.3)	4.1 (1.7-7.6)
**Ecuador**	**Leukemia**	Male	3.5 (1.6-5.9)	9.3 (4.7-14.6)
**Ecuador**	**Multiple myeloma**	Female	7.8 (2.6-15)	2.6 (1.1-4.6)
**Ecuador**	**Multiple myeloma**	Male	6.6 (1.8-13.3)	2.6 (1.1-4.7)
**Ecuador**	**Non-Hodgkin lymphoma**	Female	5.2 (0.9-10.6)	7.3 (3.5-11.7)
**Ecuador**	**Non-Hodgkin lymphoma**	Male	5.5 (1.9-10.6)	7.1 (3.2-12.1)
**Egypt**	**Leukemia**	Female	7.8 (3.1-14)	5.9 (2.6-10.4)
**Egypt**	**Leukemia**	Male	3.4 (1.4-6.4)	6.2 (3-10.3)
**Egypt**	**Multiple myeloma**	Female	9.4 (3.2-17.6)	9.4 (4.9-14.5)
**Egypt**	**Multiple myeloma**	Male	5.8 (1.6-12.3)	6.7 (3.3-10.8)
**Egypt**	**Non-Hodgkin lymphoma**	Female	5.4 (0.9-11.2)	4.9 (2-8.4)
**Egypt**	**Non-Hodgkin lymphoma**	Male	4.5 (1.5-9)	10.5 (5.4-15.8)
**El Salvador**	**Leukemia**	Female	4.7 (1.7-9.2)	2.1 (0.8-4)
**El Salvador**	**Leukemia**	Male	2.1 (0.7-4.2)	8.1 (4-13)

Countries	Causes	Sex	Percent change in 1990 (95% UI)	Percent change in 2019 (95% UI)
**El Salvador**	**Multiple myeloma**	Female	6.3 (1.9-12.5)	3.5 (1.6-6.1)
**El Salvador**	**Multiple myeloma**	Male	4.5 (1.2-10.1)	3.8 (1.7-6.5)
**El Salvador**	**Non-Hodgkin lymphoma**	Female	3.8 (0.6-8.1)	8.4 (4-13.6)
**El Salvador**	**Non-Hodgkin lymphoma**	Male	3.6 (1.1-7.6)	6.2 (2.8-10.6)
**Equatorial Guinea**	**Leukemia**	Female	1.3 (0.2-3.7)	1.9 (0.7-3.7)
**Equatorial Guinea**	**Leukemia**	Male	0.6 (0.1-1.7)	4.8 (2.3-8.1)
**Equatorial Guinea**	**Multiple myeloma**	Female	2 (0.4-5)	1.6 (0.6-3.3)
**Equatorial Guinea**	**Multiple myeloma**	Male	1.3 (0.2-3.8)	5.5 (2.3-9.7)
**Equatorial Guinea**	**Non-Hodgkin lymphoma**	Female	1.2 (0.2-3.2)	4.5 (2.2-7.6)
**Equatorial Guinea**	**Non-Hodgkin lymphoma**	Male	1 (0.2-2.9)	4.7 (2.1-8.1)
**Eritrea**	**Leukemia**	Female	0.7 (0.2-1.9)	6.3 (3-10.5)
**Eritrea**	**Leukemia**	Male	0.3 (0.1-0.8)	8 (3.5-13.2)
**Eritrea**	**Multiple myeloma**	Female	1.7 (0.4-3.9)	9.7 (4.7-15.2)
**Eritrea**	**Multiple myeloma**	Male	0.9 (0.1-2.6)	6.5 (3-10.9)
**Eritrea**	**Non-Hodgkin lymphoma**	Female	0.9 (0.1-2.4)	1.8 (0.6-3.6)
**Eritrea**	**Non-Hodgkin lymphoma**	Male	0.7 (0.1-1.7)	3.6 (1.7-6.2)
**Estonia**	**Leukemia**	Female	10.5 (4.1-18.8)	2.5 (0.9-4.8)
**Estonia**	**Leukemia**	Male	5.1 (2.3-8.7)	5.8 (2.5-10.3)
**Estonia**	**Multiple myeloma**	Female	8.5 (2.8-16.2)	4 (1.8-7.2)
**Estonia**	**Multiple myeloma**	Male	6.3 (1.8-12.8)	0.9 (0.3-2)
**Estonia**	**Non-Hodgkin lymphoma**	Female	6.1 (1.1-12.5)	7.5 (3.6-12.1)
**Estonia**	**Non-Hodgkin lymphoma**	Male	5.6 (2-11.1)	8.5 (4.1-13.5)
**Eswatini**	**Leukemia**	Female	9.1 (3.7-16.3)	5.4 (2.3-9.6)
**Eswatini**	**Leukemia**	Male	3.4 (1.5-6.2)	8.5 (4.2-13.4)
**Eswatini**	**Multiple myeloma**	Female	10.1 (3.5-18.8)	9 (4.4-14)
**Eswatini**	**Multiple myeloma**	Male	5.7 (1.7-11.9)	5.3 (2.4-8.9)
**Eswatini**	**Non-Hodgkin lymphoma**	Female	6.3 (1.2-12.8)	2.1 (0.7-4.4)
**Eswatini**	**Non-Hodgkin lymphoma**	Male	4.7 (1.7-9.4)	5.7 (2.6-9.6)
**Ethiopia**	**Leukemia**	Female	0.7 (0.1-2.3)	2.5 (1.1-4.5)
**Ethiopia**	**Leukemia**	Male	0.4 (0-1.2)	9.2 (4.5-14.6)
**Ethiopia**	**Multiple myeloma**	Female	1.4 (0.2-3.8)	9 (4.3-14.4)
**Ethiopia**	**Multiple myeloma**	Male	1 (0.1-3.3)	12.3 (6.5-18.3)
**Ethiopia**	**Non-Hodgkin lymphoma**	Female	1 (0.1-2.8)	7.1 (3.4-11.4)
**Ethiopia**	**Non-Hodgkin lymphoma**	Male	1 (0.2-2.8)	8.6 (4.4-13.8)
**Fiji**	**Leukemia**	Female	7.6 (3-13.5)	1.4 (0.4-3)
**Fiji**	**Leukemia**	Male	3.7 (1.5-7)	6.8 (3.2-11.3)
**Fiji**	**Multiple myeloma**	Female	8.7 (2.9-16.3)	5.9 (2.6-10.1)
**Fiji**	**Multiple myeloma**	Male	5.9 (1.6-12.6)	6.9 (3.3-11.3)
**Fiji**	**Non-Hodgkin lymphoma**	Female	5.5 (1-11.1)	4.5 (2-7.9)
**Fiji**	**Non-Hodgkin lymphoma**	Male	5.2 (1.7-10.5)	10.9 (5.8-16.6)
**Finland**	**Leukemia**	Female	8.6 (3.1-16.1)	9.2 (4.5-14.7)
**Finland**	**Leukemia**	Male	5.1 (2.1-9.1)	4.9 (2.2-8.4)
**Finland**	**Multiple myeloma**	Female	6.5 (2-12.9)	4.3 (1.8-7.7)
**Finland**	**Multiple myeloma**	Male	5.7 (1.6-12.1)	8.8 (4.1-14.1)

Countries	Causes	Sex	Percent change in 1990 (95% UI)	Percent change in 2019 (95% UI)
**Finland**	**Non-Hodgkin lymphoma**	Female	4.8 (0.8-10.1)	3.9 (1.5-7.2)
**Finland**	**Non-Hodgkin lymphoma**	Male	5.7 (2-11.4)	0.4 (0.1-1.2)
**France**	**Leukemia**	Female	7.4 (2.8-14.2)	1.6 (0.6-3.2)
**France**	**Leukemia**	Male	4.7 (1.9-8.4)	6.9 (3-11.6)
**France**	**Multiple myeloma**	Female	5.7 (1.7-11.1)	4.5 (2.1-7.7)
**France**	**Multiple myeloma**	Male	5.1 (1.4-11.2)	5.5 (2.5-9.4)
**France**	**Non-Hodgkin lymphoma**	Female	4.2 (0.7-8.8)	5.8 (2.5-10.1)
**France**	**Non-Hodgkin lymphoma**	Male	5.1 (1.8-10.4)	7 (3.3-11.5)
**Gabon**	**Leukemia**	Female	4.5 (1.5-9)	2 (0.7-4.2)
**Gabon**	**Leukemia**	Male	1.6 (0.5-3.5)	4 (1.7-7.1)
**Gabon**	**Multiple myeloma**	Female	5.2 (1.5-10.6)	2.2 (0.9-4.1)
**Gabon**	**Multiple myeloma**	Male	2.8 (0.6-6.8)	6.3 (2.9-10.5)
**Gabon**	**Non-Hodgkin lymphoma**	Female	3.3 (0.6-7.5)	7.1 (3.4-11.7)
**Gabon**	**Non-Hodgkin lymphoma**	Male	2.3 (0.6-5.4)	7.1 (3.3-11.8)
**Gambia**	**Leukemia**	Female	3.4 (1.1-7.4)	5.7 (2.8-9.3)
**Gambia**	**Leukemia**	Male	1.2 (0.4-2.6)	5.4 (2.3-9.4)
**Gambia**	**Multiple myeloma**	Female	4 (1.1-8.7)	7.1 (3.5-11.5)
**Gambia**	**Multiple myeloma**	Male	2.4 (0.5-5.9)	13.3 (7.3-19.5)
**Gambia**	**Non-Hodgkin lymphoma**	Female	2.1 (0.3-5)	1.7 (0.7-3.2)
**Gambia**	**Non-Hodgkin lymphoma**	Male	1.7 (0.4-4.1)	8.9 (4.3-14.2)
**Georgia**	**Leukemia**	Female	8.1 (3.2-14.5)	5.9 (2.5-10.3)
**Georgia**	**Leukemia**	Male	4.5 (2.1-7.7)	5 (2.4-8.3)
**Georgia**	**Multiple myeloma**	Female	8.5 (2.8-15.8)	5.3 (2.4-9)
**Georgia**	**Multiple myeloma**	Male	6.8 (1.9-13.9)	1.9 (0.7-3.8)
**Georgia**	**Non-Hodgkin lymphoma**	Female	6.2 (1.1-12.5)	2 (0.8-4.1)
**Georgia**	**Non-Hodgkin lymphoma**	Male	6.5 (2.4-12.7)	1.9 (0.7-3.8)
**Germany**	**Leukemia**	Female	9.5 (3.5-17.6)	8.8 (3.8-15.3)
**Germany**	**Leukemia**	Male	5.7 (2.4-9.9)	9.6 (4.3-16.2)
**Germany**	**Multiple myeloma**	Female	7.3 (2.2-14)	7.9 (3.3-14.3)
**Germany**	**Multiple myeloma**	Male	6.3 (1.8-13.2)	3.9 (1.5-7.4)
**Germany**	**Non-Hodgkin lymphoma**	Female	5.3 (0.9-11)	7.7 (3.3-13.8)
**Germany**	**Non-Hodgkin lymphoma**	Male	6.3 (2.2-12.5)	9.9 (4.4-16.8)
**Ghana**	**Leukemia**	Female	3 (0.9-6.6)	7.4 (3.1-13.3)
**Ghana**	**Leukemia**	Male	1 (0.2-2.3)	9.7 (4.3-16.5)
**Ghana**	**Multiple myeloma**	Female	3.3 (0.8-7.2)	11.9 (5.7-19.7)
**Ghana**	**Multiple myeloma**	Male	2.1 (0.4-5.5)	2.4 (0.9-5)
**Ghana**	**Non-Hodgkin lymphoma**	Female	1.7 (0.3-4)	9.6 (4.2-16.6)
**Ghana**	**Non-Hodgkin lymphoma**	Male	1.3 (0.3-3.2)	6.9 (3-12.3)
**Greece**	**Leukemia**	Female	9.3 (3.4-17.7)	6.6 (2.8-11.9)
**Greece**	**Leukemia**	Male	5.3 (2.2-9.6)	10.3 (4.5-17.6)
**Greece**	**Multiple myeloma**	Female	7.1 (2.1-14.2)	8.6 (3.9-14.9)
**Greece**	**Multiple myeloma**	Male	5.8 (1.6-12.3)	9.1 (4.1-15.8)
**Greece**	**Non-Hodgkin lymphoma**	Female	5.2 (0.9-11.2)	8.7 (3.9-14.9)
**Greece**	**Non-Hodgkin lymphoma**	Male	5.8 (2-11.7)	4.5 (1.7-8.7)

Countries	Causes	Sex	Percent change in 1990 (95% UI)	Percent change in 2019 (95% UI)
**Greenland**	**Leukemia**	Female	9.5 (3.5-17.4)	4.8 (1.7-9.4)
**Greenland**	**Leukemia**	Male	5.3 (2.3-9.2)	2.3 (0.8-4.9)
**Greenland**	**Multiple myeloma**	Female	8.1 (2.6-15.4)	2.7 (0.9-5.6)
**Greenland**	**Multiple myeloma**	Male	6.5 (1.8-13.4)	8.3 (3.7-14.4)
**Greenland**	**Non-Hodgkin lymphoma**	Female	5.6 (1-11.8)	2.3 (0.8-5.1)
**Greenland**	**Non-Hodgkin lymphoma**	Male	6.1 (2.1-11.9)	3.1 (1.1-6.1)
**Grenada**	**Leukemia**	Female	5.1 (1.7-10.2)	4.1 (1.4-8.4)
**Grenada**	**Leukemia**	Male	2.4 (0.7-4.8)	7.7 (3.3-13.7)
**Grenada**	**Multiple myeloma**	Female	4.8 (1.4-9.8)	6.4 (2.8-11.1)
**Grenada**	**Multiple myeloma**	Male	3 (0.7-7.2)	10.7 (5-18.1)
**Grenada**	**Non-Hodgkin lymphoma**	Female	3.4 (0.5-7.5)	9.9 (4.5-16.7)
**Grenada**	**Non-Hodgkin lymphoma**	Male	2.9 (0.8-6.6)	8.2 (3.5-14.1)
**Guam**	**Leukemia**	Female	9.3 (3.6-16.7)	10.7 (4.9-18)
**Guam**	**Leukemia**	Male	5.9 (2.6-10.1)	5.7 (2.4-10.5)
**Guam**	**Multiple myeloma**	Female	8.9 (2.9-17)	3.3 (1.3-6.6)
**Guam**	**Multiple myeloma**	Male	7.6 (2-15.7)	6.6 (2.8-11.9)
**Guam**	**Non-Hodgkin lymphoma**	Female	6 (1-12.2)	9.1 (4-15.8)
**Guam**	**Non-Hodgkin lymphoma**	Male	7.4 (2.6-14.4)	7 (2.8-12.4)
**Guatemala**	**Leukemia**	Female	2.6 (0.7-6)	10.8 (5-18.4)
**Guatemala**	**Leukemia**	Male	1.3 (0.3-3)	8.2 (3.5-14.7)
**Guatemala**	**Multiple myeloma**	Female	3.9 (0.9-9)	3.2 (1.2-6.1)
**Guatemala**	**Multiple myeloma**	Male	2.8 (0.5-7.2)	10.6 (4.8-17.9)
**Guatemala**	**Non-Hodgkin lymphoma**	Female	2.3 (0.4-5.6)	2.8 (1-5.8)
**Guatemala**	**Non-Hodgkin lymphoma**	Male	2.1 (0.5-5.2)	9.5 (4.3-15.9)
**Guinea**	**Leukemia**	Female	3.7 (1.2-7.6)	6.8 (3-12.3)
**Guinea**	**Leukemia**	Male	0.9 (0.2-2.3)	8.1 (3.7-14.2)
**Guinea**	**Multiple myeloma**	Female	3.7 (1-8.1)	8.5 (3.7-14.8)
**Guinea**	**Multiple myeloma**	Male	1.7 (0.3-4.6)	8 (3.4-14)
**Guinea**	**Non-Hodgkin lymphoma**	Female	2.1 (0.3-5.1)	7.4 (3.1-13.5)
**Guinea**	**Non-Hodgkin lymphoma**	Male	1.1 (0.2-3)	8.9 (3.8-16)
**Guinea-Bissau**	**Leukemia**	Female	2.6 (0.7-6)	9.6 (4.2-16.8)
**Guinea-Bissau**	**Leukemia**	Male	0.8 (0.2-2)	6 (2.4-11.3)
**Guinea-Bissau**	**Multiple myeloma**	Female	3.2 (0.8-7.3)	4.1 (1.5-8.2)
**Guinea-Bissau**	**Multiple myeloma**	Male	1.7 (0.3-4.6)	8.1 (3.2-14.6)
**Guinea-Bissau**	**Non-Hodgkin lymphoma**	Female	1.8 (0.3-4.6)	6.9 (2.8-12.5)
**Guinea-Bissau**	**Non-Hodgkin lymphoma**	Male	1.2 (0.2-3.2)	10.3 (4.9-17.2)
**Guyana**	**Leukemia**	Female	5 (1.7-9.8)	3.8 (1.6-6.9)
**Guyana**	**Leukemia**	Male	2.4 (0.8-4.8)	4.2 (1.7-7.7)
**Guyana**	**Multiple myeloma**	Female	5.9 (1.7-11.9)	9.7 (4.5-16.7)
**Guyana**	**Multiple myeloma**	Male	3.9 (0.9-8.9)	7.8 (3.4-13.9)
**Guyana**	**Non-Hodgkin lymphoma**	Female	3.6 (0.6-7.9)	6.8 (2.9-12.4)
**Guyana**	**Non-Hodgkin lymphoma**	Male	3.3 (1-7.3)	8.4 (3.8-14.4)
**Haiti**	**Leukemia**	Female	1.5 (0.4-3.9)	12.8 (6.1-20.9)
**Haiti**	**Leukemia**	Male	0.8 (0.2-2.2)	10.6 (4.7-17.6)

Countries	Causes	Sex	Percent change in 1990 (95% UI)	Percent change in 2019 (95% UI)
**Haiti**	**Multiple myeloma**	Female	2.6 (0.6-5.9)	7.2 (2.9-13.4)
**Haiti**	**Multiple myeloma**	Male	1.8 (0.4-4.7)	13.6 (6.6-22.1)
**Haiti**	**Non-Hodgkin lymphoma**	Female	1.4 (0.2-3.5)	3.8 (1.5-7)
**Haiti**	**Non-Hodgkin lymphoma**	Male	1.3 (0.3-3.2)	10.4 (4.5-17.7)
**Honduras**	**Leukemia**	Female	2.6 (0.8-5.5)	7.7 (3.3-13.7)
**Honduras**	**Leukemia**	Male	1.4 (0.4-3.1)	7 (2.9-12.4)
**Honduras**	**Multiple myeloma**	Female	5.1 (1.4-10.8)	10.1 (4.4-17.2)
**Honduras**	**Multiple myeloma**	Male	3.4 (0.8-8.4)	7.2 (3-12.7)
**Honduras**	**Non-Hodgkin lymphoma**	Female	2.6 (0.4-6.3)	4.3 (1.6-8.2)
**Honduras**	**Non-Hodgkin lymphoma**	Male	2.8 (0.8-6.5)	6.2 (2.9-10.9)
**Hungary**	**Leukemia**	Female	12.2 (4.8-21.1)	4.5 (1.8-8.5)
**Hungary**	**Leukemia**	Male	7.3 (3.5-11.8)	6.6 (2.8-12.1)
**Hungary**	**Multiple myeloma**	Female	9.5 (3.3-17.6)	8.1 (3.5-14.2)
**Hungary**	**Multiple myeloma**	Male	8.4 (2.4-16.1)	6.7 (2.9-11.9)
**Hungary**	**Non-Hodgkin lymphoma**	Female	6.8 (1.2-13.8)	9 (4-15.4)
**Hungary**	**Non-Hodgkin lymphoma**	Male	8 (3-14.9)	8.8 (3.7-16)
**Iceland**	**Leukemia**	Female	9.8 (3.7-18.1)	10.9 (5.1-18.4)
**Iceland**	**Leukemia**	Male	5.7 (2.3-9.9)	8.7 (3.8-15.3)
**Iceland**	**Multiple myeloma**	Female	7.6 (2.4-14.6)	3.5 (1.3-6.9)
**Iceland**	**Multiple myeloma**	Male	6.5 (1.8-13.6)	6.5 (3-11.3)
**Iceland**	**Non-Hodgkin lymphoma**	Female	5.6 (0.9-11.6)	3.4 (1.2-6.8)
**Iceland**	**Non-Hodgkin lymphoma**	Male	6.3 (2.1-12.6)	6.7 (2.8-12.3)
**India**	**Leukemia**	Female	1.3 (0.3-3.1)	7.6 (3.1-13.6)
**India**	**Leukemia**	Male	0.7 (0.2-1.7)	3.5 (1.3-6.9)
**India**	**Multiple myeloma**	Female	1.8 (0.4-4.1)	9.3 (4.1-16.1)
**India**	**Multiple myeloma**	Male	1.2 (0.3-3.3)	9.8 (4.4-16.9)
**India**	**Non-Hodgkin lymphoma**	Female	1.1 (0.2-2.8)	6 (2.5-11)
**India**	**Non-Hodgkin lymphoma**	Male	1 (0.2-2.5)	11.5 (5.4-19.2)
**Indonesia**	**Leukemia**	Female	1.6 (0.4-3.9)	10.1 (4.7-16.8)
**Indonesia**	**Leukemia**	Male	0.6 (0.1-1.7)	8 (3.5-14.1)
**Indonesia**	**Multiple myeloma**	Female	1.8 (0.3-4.5)	4 (1.3-8.4)
**Indonesia**	**Multiple myeloma**	Male	1.3 (0.2-3.8)	7.8 (3.4-13.9)
**Indonesia**	**Non-Hodgkin lymphoma**	Female	1.2 (0.2-3.2)	4.3 (1.8-7.9)
**Indonesia**	**Non-Hodgkin lymphoma**	Male	1.1 (0.2-2.9)	10.4 (4.7-17.6)
**Iran (Islamic Republic of)**	**Leukemia**	Female	4.6 (1.7-8.8)	10.4 (4.8-17.1)
**Iran (Islamic Republic of)**	**Leukemia**	Male	2.3 (0.9-4.3)	14.7 (6.3-24.6)
**Iran (Islamic Republic of)**	**Multiple myeloma**	Female	6.8 (2.2-13.2)	10.1 (4.4-17.2)
**Iran (Islamic Republic of)**	**Multiple myeloma**	Male	4.1 (1.2-8.7)	9.8 (4.7-16.4)
**Iran (Islamic Republic of)**	**Non-Hodgkin lymphoma**	Female	4 (0.7-8.3)	4 (1.4-8.1)
**Iran (Islamic Republic of)**	**Non-Hodgkin lymphoma**	Male	3.3 (1.2-6.7)	8.7 (3.9-15.1)
**Iraq**	**Leukemia**	Female	7.6 (2.9-13.8)	7.8 (3.4-13.9)
**Iraq**	**Leukemia**	Male	4.5 (1.9-7.8)	8.8 (3.9-15.5)
**Iraq**	**Multiple myeloma**	Female	9.6 (3.3-18)	7.2 (3.1-12.6)
**Iraq**	**Multiple myeloma**	Male	7.8 (2.2-15.4)	13.2 (6.5-21.6)

Countries	Causes	Sex	Percent change in 1990 (95% UI)	Percent change in 2019 (95% UI)
**Iraq**	**Non-Hodgkin lymphoma**	Female	5.6 (1-11.8)	10.3 (4.7-17.2)
**Iraq**	**Non-Hodgkin lymphoma**	Male	6.1 (2.1-11.5)	6.3 (2.7-11.3)
**Ireland**	**Leukemia**	Female	8.4 (3.1-16.3)	4.9 (2-9.2)
**Ireland**	**Leukemia**	Male	5.3 (2.2-9.5)	10.3 (4.6-17)
**Ireland**	**Multiple myeloma**	Female	6.6 (2-13)	6 (2.3-11.4)
**Ireland**	**Multiple myeloma**	Male	5.9 (1.6-12.4)	1.5 (0.3-3.8)
**Ireland**	**Non-Hodgkin lymphoma**	Female	4.9 (0.8-10.5)	5.1 (2.2-9.2)
**Ireland**	**Non-Hodgkin lymphoma**	Male	6 (2-12)	8 (3.4-14.4)
**Israel**	**Leukemia**	Female	9.1 (3.4-17)	7.7 (3.5-13.6)
**Israel**	**Leukemia**	Male	4.9 (2.1-8.8)	7.6 (3.2-13.7)
**Israel**	**Multiple myeloma**	Female	7.3 (2.2-14.4)	6.6 (2.9-11.9)
**Israel**	**Multiple myeloma**	Male	5.6 (1.6-12.1)	9.6 (4.3-16.4)
**Israel**	**Non-Hodgkin lymphoma**	Female	5.3 (0.9-11.1)	4.3 (1.7-8.3)
**Israel**	**Non-Hodgkin lymphoma**	Male	5.5 (2-11.1)	5.3 (2.2-9.8)
**Italy**	**Leukemia**	Female	8.1 (3-15.4)	5.1 (2.1-9.4)
**Italy**	**Leukemia**	Male	4.7 (2-8.7)	8 (3.3-14.2)
**Italy**	**Multiple myeloma**	Female	6.4 (1.9-12.6)	9.8 (4.4-16.6)
**Italy**	**Multiple myeloma**	Male	5.4 (1.5-11.7)	9.3 (4-16)
**Italy**	**Non-Hodgkin lymphoma**	Female	4.7 (0.8-10.1)	9.8 (4.5-16.6)
**Italy**	**Non-Hodgkin lymphoma**	Male	5.4 (1.8-10.8)	6.7 (2.7-12.3)
**Jamaica**	**Leukemia**	Female	6.9 (2.6-12.5)	10.1 (4.5-17)
**Jamaica**	**Leukemia**	Male	2.6 (0.9-5)	14.6 (6-24.9)
**Jamaica**	**Multiple myeloma**	Female	7.2 (2.3-14.1)	5.1 (2.1-9.1)
**Jamaica**	**Multiple myeloma**	Male	3.9 (1-9.1)	10 (4.7-16.7)
**Jamaica**	**Non-Hodgkin lymphoma**	Female	5 (0.9-10.2)	7.6 (3.1-13.6)
**Jamaica**	**Non-Hodgkin lymphoma**	Male	3.7 (1.2-7.9)	8.5 (3.8-14.8)
**Japan**	**Leukemia**	Female	4 (1-9.2)	7.7 (3.3-13.7)
**Japan**	**Leukemia**	Male	2.3 (0.6-5.1)	2.6 (0.9-5.4)
**Japan**	**Multiple myeloma**	Female	3.2 (0.7-7.4)	5 (2.1-9.1)
**Japan**	**Multiple myeloma**	Male	2.5 (0.5-6.6)	5.7 (2.2-10.5)
**Japan**	**Non-Hodgkin lymphoma**	Female	2.3 (0.4-5.8)	9.5 (3.2-18.1)
**Japan**	**Non-Hodgkin lymphoma**	Male	2.5 (0.6-6.1)	11 (3.8-20.1)
**Jordan**	**Leukemia**	Female	9 (3.6-15.9)	8.4 (2.7-16)
**Jordan**	**Leukemia**	Male	4.6 (2.1-8)	4.5 (1.3-9.1)
**Jordan**	**Multiple myeloma**	Female	10.3 (3.5-19.3)	8.4 (2.8-16.2)
**Jordan**	**Multiple myeloma**	Male	7.6 (2.2-15.6)	10.9 (3.7-19.8)
**Jordan**	**Non-Hodgkin lymphoma**	Female	6.6 (1.2-13.2)	7.9 (2.6-15.3)
**Jordan**	**Non-Hodgkin lymphoma**	Male	6.6 (2.4-12.5)	11 (3.7-20.1)
**Kazakhstan**	**Leukemia**	Female	8.3 (3.3-14.6)	13.4 (4.8-24)
**Kazakhstan**	**Leukemia**	Male	3.4 (1.5-6)	2.6 (0.7-5.6)
**Kazakhstan**	**Multiple myeloma**	Female	9.8 (3.3-18.2)	10.7 (3.6-19.5)
**Kazakhstan**	**Multiple myeloma**	Male	6 (1.7-12.5)	7.5 (2.3-14.5)
**Kazakhstan**	**Non-Hodgkin lymphoma**	Female	5.8 (1-11.9)	7.6 (2.5-14.4)
**Kazakhstan**	**Non-Hodgkin lymphoma**	Male	4.8 (1.7-9.3)	11.2 (3.9-20.6)

Countries	Causes	Sex	Percent change in 1990 (95% UI)	Percent change in 2019 (95% UI)
**Kenya**	**Leukemia**	Female	1.7 (0.5-4)	9.6 (3.3-18.1)
**Kenya**	**Leukemia**	Male	1 (0.3-2.2)	9.8 (3.3-18.2)
**Kenya**	**Multiple myeloma**	Female	3.6 (0.9-7.7)	9.3 (3.2-17.2)
**Kenya**	**Multiple myeloma**	Male	2.3 (0.5-5.8)	4.8 (1.4-10)
**Kenya**	**Non-Hodgkin lymphoma**	Female	2.2 (0.3-5)	6.3 (1.9-12.7)
**Kenya**	**Non-Hodgkin lymphoma**	Male	1.8 (0.5-4.3)	2.9 (0.8-6.4)
**Kiribati**	**Leukemia**	Female	7 (2.6-13.1)	3 (0.8-6.6)
**Kiribati**	**Leukemia**	Male	3.9 (1.6-6.9)	9.6 (3.2-18.1)
**Kiribati**	**Multiple myeloma**	Female	6.9 (2.1-13.8)	2.9 (0.8-6.3)
**Kiribati**	**Multiple myeloma**	Male	5.9 (1.6-12.5)	4 (1.1-8.2)
**Kiribati**	**Non-Hodgkin lymphoma**	Female	4.6 (0.8-9.7)	4.4 (1.1-9.7)
**Kiribati**	**Non-Hodgkin lymphoma**	Male	5.3 (1.8-10.5)	8.6 (2.8-16.4)
**Kuwait**	**Leukemia**	Female	9.5 (4-16)	7.5 (2.6-14.3)
**Kuwait**	**Leukemia**	Male	5.9 (2.8-9.6)	11.1 (3.8-20.6)
**Kuwait**	**Multiple myeloma**	Female	12.1 (4.3-22)	10.3 (3.4-19.1)
**Kuwait**	**Multiple myeloma**	Male	9.2 (2.6-17.9)	9.6 (3.2-17.8)
**Kuwait**	**Non-Hodgkin lymphoma**	Female	7.6 (1.4-15)	11 (3.9-20.7)
**Kuwait**	**Non-Hodgkin lymphoma**	Male	7.9 (3-14.4)	6.5 (2-12.7)
**Kyrgyzstan**	**Leukemia**	Female	5.5 (2-10.3)	3.4 (0.9-7.1)
**Kyrgyzstan**	**Leukemia**	Male	2.4 (0.9-4.4)	7.1 (2.3-13.7)
**Kyrgyzstan**	**Multiple myeloma**	Female	7.2 (2.3-13.8)	10.3 (3.5-19.3)
**Kyrgyzstan**	**Multiple myeloma**	Male	4.9 (1.3-10.5)	7.5 (2.4-14.8)
**Kyrgyzstan**	**Non-Hodgkin lymphoma**	Female	4.7 (0.8-10)	13.4 (4.9-24.2)
**Kyrgyzstan**	**Non-Hodgkin lymphoma**	Male	3.9 (1.4-7.9)	9.1 (3-17.3)
**Lao People's Democratic Republic**	**Leukemia**	Female	1.2 (0.2-3.3)	4 (1.2-8.1)
**Lao People's Democratic Republic**	**Leukemia**	Male	0.5 (0.1-1.4)	11.6 (3.9-21.2)
**Lao People's Democratic Republic**	**Multiple myeloma**	Female	1.7 (0.3-4.3)	3.2 (0.8-6.9)
**Lao People's Democratic Republic**	**Multiple myeloma**	Male	1.1 (0.1-3.3)	10.5 (3.6-19.5)
**Lao People's Democratic Republic**	**Non-Hodgkin lymphoma**	Female	1 (0.1-2.7)	7.4 (2.4-14.1)
**Lao People's Democratic Republic**	**Non-Hodgkin lymphoma**	Male	0.8 (0.1-2.5)	9.8 (3.5-18.4)
**Latvia**	**Leukemia**	Female	11.6 (4.5-20.5)	9.2 (3.1-17.1)
**Latvia**	**Leukemia**	Male	5.2 (2.3-9)	8.5 (2.8-16.2)
**Latvia**	**Multiple myeloma**	Female	9.7 (3.2-18.5)	8.1 (2.7-16)
**Latvia**	**Multiple myeloma**	Male	6.4 (1.8-13.1)	9.7 (3.3-18.4)
**Latvia**	**Non-Hodgkin lymphoma**	Female	6.8 (1.2-13.8)	10.7 (3.7-19.9)
**Latvia**	**Non-Hodgkin lymphoma**	Male	5.7 (2-11.5)	6.7 (2-13.5)
**Lebanon**	**Leukemia**	Female	9.5 (3.7-17.5)	5.1 (1.6-10.7)
**Lebanon**	**Leukemia**	Male	4.7 (2-8.2)	8.9 (3-16.9)

Countries	Causes	Sex	Percent change in 1990 (95% UI)	Percent change in 2019 (95% UI)
**Lebanon**	**Multiple myeloma**	Female	8.7 (2.8-16.4)	7.9 (2.5-15.2)
**Lebanon**	**Multiple myeloma**	Male	6.1 (1.7-12.7)	10.6 (3.6-19.7)
**Lebanon**	**Non-Hodgkin lymphoma**	Female	5.9 (1-12.1)	4.3 (1.3-8.4)
**Lebanon**	**Non-Hodgkin lymphoma**	Male	5.6 (2-11.2)	5 (1.5-10)
**Lesotho**	**Leukemia**	Female	6 (2.1-11.5)	10.3 (3.6-19.1)
**Lesotho**	**Leukemia**	Male	1.5 (0.4-3.4)	8.2 (2.7-15.8)
**Lesotho**	**Multiple myeloma**	Female	5.9 (1.8-11.7)	7.4 (2.3-14.4)
**Lesotho**	**Multiple myeloma**	Male	2.2 (0.5-5.5)	10.2 (3.5-18.5)
**Lesotho**	**Non-Hodgkin lymphoma**	Female	3.9 (0.6-8.4)	13.9 (5-24.9)
**Lesotho**	**Non-Hodgkin lymphoma**	Male	1.9 (0.4-4.6)	11.9 (4.2-21.8)
**Liberia**	**Leukemia**	Female	5 (1.8-9.8)	7.5 (2.3-14.8)
**Liberia**	**Leukemia**	Male	1.6 (0.5-3.7)	15.7 (5.8-27.5)
**Liberia**	**Multiple myeloma**	Female	5.9 (1.7-11.9)	4.2 (1.3-8.4)
**Liberia**	**Multiple myeloma**	Male	3.3 (0.8-7.6)	11.6 (4-21.3)
**Liberia**	**Non-Hodgkin lymphoma**	Female	3.6 (0.6-7.7)	10.4 (3.6-19.2)
**Liberia**	**Non-Hodgkin lymphoma**	Male	2.3 (0.7-5.4)	8.1 (2.7-15.3)
**Libya**	**Leukemia**	Female	8.1 (3.1-14.8)	11 (3.9-20)
**Libya**	**Leukemia**	Male	4.5 (2-7.9)	7.6 (2.5-14.5)
**Libya**	**Multiple myeloma**	Female	10 (3.4-18.6)	4.9 (1.4-10.2)
**Libya**	**Multiple myeloma**	Male	7 (2-14)	6.8 (2.1-13)
**Libya**	**Non-Hodgkin lymphoma**	Female	6.3 (1.2-12.8)	5.6 (1.7-11)
**Libya**	**Non-Hodgkin lymphoma**	Male	6 (2.3-11.7)	7.4 (2.3-14.4)
**Lithuania**	**Leukemia**	Female	10.9 (4.3-19.8)	9.1 (3-17)
**Lithuania**	**Leukemia**	Male	5.2 (2.2-9.1)	7.8 (2.5-15.1)
**Lithuania**	**Multiple myeloma**	Female	9.2 (3.1-17.2)	10 (3.5-18.6)
**Lithuania**	**Multiple myeloma**	Male	6.3 (1.8-13.2)	9.5 (3.1-18)
**Lithuania**	**Non-Hodgkin lymphoma**	Female	6.4 (1.1-13.1)	11.2 (3.9-20.6)
**Lithuania**	**Non-Hodgkin lymphoma**	Male	5.7 (2-11.5)	10.2 (3.3-18.9)
**Luxembourg**	**Leukemia**	Female	8.7 (3.3-16.4)	4.2 (1.2-8.5)
**Luxembourg**	**Leukemia**	Male	5.2 (2.2-9.2)	8.2 (2.9-15.4)
**Luxembourg**	**Multiple myeloma**	Female	6.7 (2-13)	3.8 (1-8.1)
**Luxembourg**	**Multiple myeloma**	Male	5.8 (1.7-12.3)	7.4 (2.3-14.3)
**Luxembourg**	**Non-Hodgkin lymphoma**	Female	4.9 (0.9-10.2)	8.7 (2.9-16.6)
**Luxembourg**	**Non-Hodgkin lymphoma**	Male	5.7 (2-11.4)	4.4 (1.3-9.4)
**Madagascar**	**Leukemia**	Female	1 (0.3-2.2)	9.8 (3.3-18.2)
**Madagascar**	**Leukemia**	Male	0.5 (0.1-1.1)	10.4 (3.5-19.2)
**Madagascar**	**Multiple myeloma**	Female	2.4 (0.6-5.3)	6.6 (2-12.8)
**Madagascar**	**Multiple myeloma**	Male	1.3 (0.2-3.5)	12.9 (4.6-23.2)
**Madagascar**	**Non-Hodgkin lymphoma**	Female	1.3 (0.2-3.1)	10.4 (3.7-19.4)
**Madagascar**	**Non-Hodgkin lymphoma**	Male	0.9 (0.2-2.4)	9.4 (3.2-17.3)
**Malawi**	**Leukemia**	Female	1.1 (0.2-3.1)	4.8 (1.3-10.2)
**Malawi**	**Leukemia**	Male	0.6 (0.1-1.6)	8.3 (2.7-16)
**Malawi**	**Multiple myeloma**	Female	2.1 (0.4-5.5)	4.8 (1.4-9.3)
**Malawi**	**Multiple myeloma**	Male	1.7 (0.3-4.8)	11 (3.9-20)

Countries	Causes	Sex	Percent change in 1990 (95% UI)	Percent change in 2019 (95% UI)
**Malawi**	**Non-Hodgkin lymphoma**	Female	0.9 (0.1-2.7)	11.1 (3.9-20.4)
**Malawi**	**Non-Hodgkin lymphoma**	Male	0.9 (0.2-2.6)	16.4 (6.2-28.4)
**Malaysia**	**Leukemia**	Female	3.8 (1.2-7.8)	11.4 (3.9-21)
**Malaysia**	**Leukemia**	Male	1.8 (0.7-3.6)	10.2 (3.5-18.8)
**Malaysia**	**Multiple myeloma**	Female	4 (1.1-8.5)	5.1 (1.4-10.3)
**Malaysia**	**Multiple myeloma**	Male	3 (0.7-6.9)	9.8 (3.3-18.3)
**Malaysia**	**Non-Hodgkin lymphoma**	Female	2.8 (0.5-6.4)	9 (3-16.9)
**Malaysia**	**Non-Hodgkin lymphoma**	Male	2.8 (0.9-5.9)	9.1 (3.1-17.2)
**Maldives**	**Leukemia**	Female	1.3 (0.3-3.5)	8 (2.6-15.4)
**Maldives**	**Leukemia**	Male	0.6 (0.1-1.6)	15.3 (5.7-26.8)
**Maldives**	**Multiple myeloma**	Female	1.9 (0.4-5)	10.6 (3.7-19.4)
**Maldives**	**Multiple myeloma**	Male	1.3 (0.2-3.9)	7.1 (2.2-13.8)
**Maldives**	**Non-Hodgkin lymphoma**	Female	1.2 (0.2-3.4)	5.1 (1.5-10.3)
**Maldives**	**Non-Hodgkin lymphoma**	Male	1 (0.2-3)	10.9 (3.8-20.2)
**Mali**	**Leukemia**	Female	2.2 (0.6-5.2)	6.6 (2-13.2)
**Mali**	**Leukemia**	Male	0.7 (0.2-1.9)	1.8 (0.3-4.7)
**Mali**	**Multiple myeloma**	Female	2.7 (0.7-6.3)	6.3 (2-12.1)
**Mali**	**Multiple myeloma**	Male	1.5 (0.3-4.3)	8.8 (2.9-16.9)
**Mali**	**Non-Hodgkin lymphoma**	Female	1.4 (0.2-3.6)	9 (3.1-17.2)
**Mali**	**Non-Hodgkin lymphoma**	Male	1 (0.2-2.9)	9.1 (3-17)
**Malta**	**Leukemia**	Female	7.4 (2.7-14.2)	7.1 (2.2-13.5)
**Malta**	**Leukemia**	Male	3.9 (1.5-7.4)	10.9 (3.8-20)
**Malta**	**Multiple myeloma**	Female	5.8 (1.6-11.8)	5.6 (1.6-11.1)
**Malta**	**Multiple myeloma**	Male	4.4 (1.1-9.9)	6 (1.8-12)
**Malta**	**Non-Hodgkin lymphoma**	Female	4.2 (0.7-9.1)	6.4 (2-12.2)
**Malta**	**Non-Hodgkin lymphoma**	Male	4.4 (1.4-9)	8.6 (2.8-16.5)
**Marshall Islands**	**Leukemia**	Female	4.5 (1.4-9.6)	11.2 (3.9-20.7)
**Marshall Islands**	**Leukemia**	Male	2.6 (0.8-5.6)	10.6 (3.7-19.9)
**Marshall Islands**	**Multiple myeloma**	Female	4.9 (1.2-10.7)	10.6 (3.5-19.6)
**Marshall Islands**	**Multiple myeloma**	Male	3.8 (0.8-9)	7.3 (2.2-14.2)
**Marshall Islands**	**Non-Hodgkin lymphoma**	Female	3.1 (0.5-7.2)	11.8 (4.2-21.6)
**Marshall Islands**	**Non-Hodgkin lymphoma**	Male	3.4 (0.9-7.7)	15.6 (5.9-27.2)
**Mauritania**	**Leukemia**	Female	6.5 (2.5-12.2)	6.2 (2-12)
**Mauritania**	**Leukemia**	Male	2.1 (0.8-4.1)	10.7 (3.7-19.6)
**Mauritania**	**Multiple myeloma**	Female	6.2 (1.9-12.3)	8.3 (2.8-16.1)
**Mauritania**	**Multiple myeloma**	Male	3.5 (0.9-8)	9.5 (3.2-18)
**Mauritania**	**Non-Hodgkin lymphoma**	Female	4 (0.7-8.3)	8.6 (2.8-16.4)
**Mauritania**	**Non-Hodgkin lymphoma**	Male	2.7 (0.8-5.9)	3 (0.8-6.5)
**Mauritius**	**Leukemia**	Female	4.5 (1.7-8.8)	5.9 (1.9-11.6)
**Mauritius**	**Leukemia**	Male	1.9 (0.8-3.7)	7.5 (2.4-14)
**Mauritius**	**Multiple myeloma**	Female	5.1 (1.5-10)	8.1 (2.3-16.2)
**Mauritius**	**Multiple myeloma**	Male	3.2 (0.8-7)	8.3 (2.3-16.2)
**Mauritius**	**Non-Hodgkin lymphoma**	Female	3.2 (0.6-7.1)	7.4 (2-15)
**Mauritius**	**Non-Hodgkin lymphoma**	Male	2.8 (0.9-5.8)	3.4 (0.9-7.4)

Countries	Causes	Sex	Percent change in 1990 (95% UI)	Percent change in 2019 (95% UI)
**Mexico**	**Leukemia**	Female	5.7 (2.2-10.5)	6.9 (1.9-14.1)
**Mexico**	**Leukemia**	Male	3.1 (1.3-5.4)	8.5 (2.6-16.6)
**Mexico**	**Multiple myeloma**	Female	8.7 (2.9-16.6)	6.9 (1.8-14.1)
**Mexico**	**Multiple myeloma**	Male	6.7 (1.8-13.9)	8.4 (2.4-16.7)
**Mexico**	**Non-Hodgkin lymphoma**	Female	4.9 (0.9-10)	11.1 (3.3-20.5)
**Mexico**	**Non-Hodgkin lymphoma**	Male	5.1 (1.8-10)	2.3 (0.6-5.3)
**Micronesia (Federated States of)**	**Leukemia**	Female	8.1 (3.2-14.7)	8.1 (2.3-15.9)
**Micronesia (Federated States of)**	**Leukemia**	Male	4.1 (1.7-7.2)	6.3 (1.8-13.2)
**Micronesia (Federated States of)**	**Multiple myeloma**	Female	8 (2.5-15.5)	5.6 (1.7-11.5)
**Micronesia (Federated States of)**	**Multiple myeloma**	Male	5.9 (1.7-12.5)	9.4 (2.7-18.3)
**Micronesia (Federated States of)**	**Non-Hodgkin lymphoma**	Female	5.2 (0.9-10.8)	7.8 (2.2-15.3)
**Micronesia (Federated States of)**	**Non-Hodgkin lymphoma**	Male	5.4 (1.8-10.6)	8.4 (2.4-16.7)
**Monaco**	**Leukemia**	Female	11.4 (4.4-20.5)	8.1 (2.3-16.1)
**Monaco**	**Leukemia**	Male	6.8 (2.9-11.7)	4.3 (1.1-9.4)
**Monaco**	**Multiple myeloma**	Female	8.5 (2.8-16.4)	3 (0.7-7.1)
**Monaco**	**Multiple myeloma**	Male	7.2 (1.9-14.8)	1.8 (0.4-4.6)
**Monaco**	**Non-Hodgkin lymphoma**	Female	6.3 (1.1-13.2)	2.3 (0.6-5.4)
**Monaco**	**Non-Hodgkin lymphoma**	Male	7.3 (2.6-14.6)	7.2 (2.1-14.5)
**Mongolia**	**Leukemia**	Female	4.9 (1.7-9.5)	1.8 (0.4-4.8)
**Mongolia**	**Leukemia**	Male	2.1 (0.8-4.3)	2.2 (0.5-5.4)
**Mongolia**	**Multiple myeloma**	Female	6.2 (1.8-12.7)	3.8 (1-8.8)
**Mongolia**	**Multiple myeloma**	Male	4.4 (1.2-9.7)	6.7 (1.9-13.9)
**Mongolia**	**Non-Hodgkin lymphoma**	Female	3.6 (0.6-7.9)	5.4 (1.6-11.3)
**Mongolia**	**Non-Hodgkin lymphoma**	Male	3.8 (1.3-7.9)	10.3 (3.1-19.4)
**Montenegro**	**Leukemia**	Female	11.3 (4.6-19.9)	9.5 (2.7-18.3)
**Montenegro**	**Leukemia**	Male	7.1 (3.4-11.5)	6.9 (2.1-14)
**Montenegro**	**Multiple myeloma**	Female	10.3 (3.5-19.3)	10.3 (2.9-19.7)
**Montenegro**	**Multiple myeloma**	Male	9 (2.5-17.4)	5.1 (1.5-10.7)
**Montenegro**	**Non-Hodgkin lymphoma**	Female	6.9 (1.3-13.7)	3.3 (0.9-7.2)
**Montenegro**	**Non-Hodgkin lymphoma**	Male	8 (3-14.7)	6.2 (1.8-12.9)
**Morocco**	**Leukemia**	Female	6.2 (2.3-11.9)	8 (2.2-15.7)
**Morocco**	**Leukemia**	Male	3.2 (1.3-5.8)	6.5 (1.8-13.6)
**Morocco**	**Multiple myeloma**	Female	6.5 (2-12.9)	9.8 (2.8-18.6)
**Morocco**	**Multiple myeloma**	Male	4.6 (1.2-9.9)	7.1 (2-14.5)
**Morocco**	**Non-Hodgkin lymphoma**	Female	4 (0.7-8.7)	2.1 (0.5-4.8)
**Morocco**	**Non-Hodgkin lymphoma**	Male	4.1 (1.4-8.5)	9.2 (2.9-17.6)
**Mozambique**	**Leukemia**	Female	0.7 (0.1-2)	2.5 (0.6-5.9)
**Mozambique**	**Leukemia**	Male	0.4 (0.1-1.1)	8.6 (2.6-16.3)
**Mozambique**	**Multiple myeloma**	Female	1.6 (0.3-4.3)	6.4 (1.8-13.3)
**Mozambique**	**Multiple myeloma**	Male	1.1 (0.1-3.6)	6.9 (2-14.1)
**Mozambique**	**Non-Hodgkin lymphoma**	Female	1.1 (0.1-3.2)	7.6 (2.2-15.5)
**Mozambique**	**Non-Hodgkin lymphoma**	Male	1 (0.2-3)	7.5 (2.1-15.1)
**Myanmar**	**Leukemia**	Female	1.1 (0.2-3.3)	6.7 (1.9-14)
**Myanmar**	**Leukemia**	Male	0.5 (0.1-1.5)	8.5 (2.4-16.8)

Countries	Causes	Sex	Percent change in 1990 (95% UI)	Percent change in 2019 (95% UI)
**Myanmar**	**Multiple myeloma**	Female	1.4 (0.2-3.9)	9.2 (2.5-17.9)
**Myanmar**	**Multiple myeloma**	Male	0.9 (0.1-3)	5.1 (1.4-11.1)
**Myanmar**	**Non-Hodgkin lymphoma**	Female	0.9 (0.1-2.8)	3.1 (0.7-7.2)
**Myanmar**	**Non-Hodgkin lymphoma**	Male	0.8 (0.1-2.5)	6.5 (1.8-13.4)
**Namibia**	**Leukemia**	Female	6.1 (2.3-11.2)	5.9 (1.6-12.5)
**Namibia**	**Leukemia**	Male	2.1 (0.8-4)	10 (3.1-18.9)
**Namibia**	**Multiple myeloma**	Female	5.7 (1.9-11.1)	3.3 (0.9-6.9)
**Namibia**	**Multiple myeloma**	Male	3 (0.8-6.5)	3.4 (1-7.3)
**Namibia**	**Non-Hodgkin lymphoma**	Female	3.9 (0.7-8.2)	9.3 (2.7-17.8)
**Namibia**	**Non-Hodgkin lymphoma**	Male	2.7 (0.9-5.5)	7.5 (2.2-15.3)
**Nauru**	**Leukemia**	Female	7.5 (2.9-13.7)	6.3 (1.8-13.3)
**Nauru**	**Leukemia**	Male	4.8 (2.1-8.2)	7.1 (2.1-14.1)
**Nauru**	**Multiple myeloma**	Female	8.9 (3-16.7)	12 (3.6-22)
**Nauru**	**Multiple myeloma**	Male	7.4 (2.1-15.1)	9 (2.6-17.3)
**Nauru**	**Non-Hodgkin lymphoma**	Female	5.4 (1-10.9)	6.7 (1.9-13.9)
**Nauru**	**Non-Hodgkin lymphoma**	Male	6.5 (2.3-12.5)	12.4 (3.7-22.6)
**Nepal**	**Leukemia**	Female	0.9 (0.1-2.6)	3.3 (0.9-7)
**Nepal**	**Leukemia**	Male	0.5 (0.1-1.4)	8.8 (2.5-17.2)
**Nepal**	**Multiple myeloma**	Female	1.2 (0.2-3.1)	4.8 (1.4-9.9)
**Nepal**	**Multiple myeloma**	Male	0.9 (0.2-2.8)	6.1 (1.7-12.3)
**Nepal**	**Non-Hodgkin lymphoma**	Female	0.5 (0.1-1.5)	9 (2.5-17.5)
**Nepal**	**Non-Hodgkin lymphoma**	Male	0.7 (0.1-2)	6.7 (1.9-13.7)
**Netherlands**	**Leukemia**	Female	8.5 (3.1-16.2)	3.7 (0.9-8.2)
**Netherlands**	**Leukemia**	Male	4.3 (1.7-8)	5.9 (1.7-11.6)
**Netherlands**	**Multiple myeloma**	Female	6.6 (2-13.2)	3.4 (0.9-7.5)
**Netherlands**	**Multiple myeloma**	Male	4.8 (1.3-10.7)	5.8 (1.6-12.4)
**Netherlands**	**Non-Hodgkin lymphoma**	Female	4.8 (0.8-10.4)	6.8 (2.1-13.6)
**Netherlands**	**Non-Hodgkin lymphoma**	Male	4.8 (1.6-10)	5.7 (1.7-11.7)
**New Zealand**	**Leukemia**	Female	8.8 (3.3-16.3)	7.9 (2.3-15.6)
**New Zealand**	**Leukemia**	Male	5.4 (2.3-9.5)	8.3 (2.4-16.8)
**New Zealand**	**Multiple myeloma**	Female	6.8 (2.1-13.1)	10.6 (3.2-20.1)
**New Zealand**	**Multiple myeloma**	Male	6.2 (1.8-13.1)	7.6 (2.2-15.3)
**New Zealand**	**Non-Hodgkin lymphoma**	Female	5 (0.9-10.6)	2.6 (0.7-6.1)
**New Zealand**	**Non-Hodgkin lymphoma**	Male	6.2 (2.1-12.3)	5.1 (1.5-10.3)
**Nicaragua**	**Leukemia**	Female	3 (0.9-6.2)	3 (0.8-6.8)
**Nicaragua**	**Leukemia**	Male	1.4 (0.4-3)	6.2 (1.7-13)
**Nicaragua**	**Multiple myeloma**	Female	6 (1.8-12)	6.5 (1.8-13.5)
**Nicaragua**	**Multiple myeloma**	Male	3.6 (0.8-8.5)	2.4 (0.6-5.8)
**Nicaragua**	**Non-Hodgkin lymphoma**	Female	3 (0.5-6.8)	8.9 (2.5-17.4)
**Nicaragua**	**Non-Hodgkin lymphoma**	Male	2.3 (0.6-5.2)	9.4 (2.8-18.2)
**Niger**	**Leukemia**	Female	1.8 (0.5-4.2)	5.6 (1.6-12)
**Niger**	**Leukemia**	Male	0.5 (0.1-1.5)	10.7 (3.2-20)
**Niger**	**Multiple myeloma**	Female	3 (0.8-6.6)	9.7 (2.9-18.5)
**Niger**	**Multiple myeloma**	Male	1.5 (0.3-4)	7.1 (2-14.4)

Countries	Causes	Sex	Percent change in 1990 (95% UI)	Percent change in 2019 (95% UI)
**Niger**	**Non-Hodgkin lymphoma**	Female	1.3 (0.2-3.5)	3.4 (0.7-8.2)
**Niger**	**Non-Hodgkin lymphoma**	Male	0.8 (0.2-2.3)	7.4 (2.1-14.9)
**Nigeria**	**Leukemia**	Female	3.1 (0.9-7)	3.8 (1.1-8.1)
**Nigeria**	**Leukemia**	Male	1.4 (0.4-3.2)	9.9 (2.9-18.7)
**Nigeria**	**Multiple myeloma**	Female	3.2 (0.8-7.2)	9.7 (2.9-18.5)
**Nigeria**	**Multiple myeloma**	Male	2.6 (0.6-6.2)	14.3 (4.5-25.4)
**Nigeria**	**Non-Hodgkin lymphoma**	Female	1.8 (0.3-4.4)	8.3 (2.4-16.4)
**Nigeria**	**Non-Hodgkin lymphoma**	Male	1.6 (0.4-4.2)	9.4 (3-18)
**Niue**	**Leukemia**	Female	8.9 (3.3-16.5)	2.7 (0.6-6.6)
**Niue**	**Leukemia**	Male	5 (2.2-8.7)	7.7 (2.2-15.5)
**Niue**	**Multiple myeloma**	Female	7.7 (2.4-14.9)	6.7 (1.9-13.9)
**Niue**	**Multiple myeloma**	Male	6.4 (1.7-13.1)	8.6 (2.4-16.7)
**Niue**	**Non-Hodgkin lymphoma**	Female	5.2 (0.9-10.7)	6.3 (1.8-12.7)
**Niue**	**Non-Hodgkin lymphoma**	Male	6.2 (2.1-12)	12.2 (3.8-22.1)
**North Macedonia**	**Leukemia**	Female	9.3 (3.5-17.1)	10 (2.9-18.8)
**North Macedonia**	**Leukemia**	Male	5.2 (2.3-9)	5.5 (1.6-11.3)
**North Macedonia**	**Multiple myeloma**	Female	8.9 (2.9-16.8)	4.7 (1.3-10.1)
**North Macedonia**	**Multiple myeloma**	Male	6.8 (1.9-14)	9.5 (2.7-18.3)
**North Macedonia**	**Non-Hodgkin lymphoma**	Female	5.7 (1-11.8)	5.4 (1.4-11.8)
**North Macedonia**	**Non-Hodgkin lymphoma**	Male	6 (2.2-11.6)	1.1 (0.1-3.4)
**Northern Mariana Islands**	**Leukemia**	Female	13 (5.5-22.2)	4.1 (1.2-8.7)
**Northern Mariana Islands**	**Leukemia**	Male	9.1 (4.5-14.3)	7.3 (2.1-14.7)
**Northern Mariana Islands**	**Multiple myeloma**	Female	11.9 (4.2-21.3)	6.9 (2-14)
**Northern Mariana Islands**	**Multiple myeloma**	Male	10.9 (3.3-20.1)	6.8 (1.9-14)
**Northern Mariana Islands**	**Non-Hodgkin lymphoma**	Female	8.1 (1.4-15.7)	6.1 (1.7-12.8)
**Northern Mariana Islands**	**Non-Hodgkin lymphoma**	Male	10.6 (4.2-18.6)	8.3 (2.4-16.2)
**Norway**	**Leukemia**	Female	6.9 (2.5-13.1)	3.4 (0.8-7.7)
**Norway**	**Leukemia**	Male	4.2 (1.7-7.6)	4.5 (1.3-9.4)
**Norway**	**Multiple myeloma**	Female	5.3 (1.6-10.5)	3.6 (1-7.9)
**Norway**	**Multiple myeloma**	Male	4.5 (1.3-10)	7.2 (2-14.6)
**Norway**	**Non-Hodgkin lymphoma**	Female	3.9 (0.7-8.5)	8.3 (2.4-16.7)
**Norway**	**Non-Hodgkin lymphoma**	Male	4.6 (1.6-9.3)	8.1 (2.3-16.2)
**Oman**	**Leukemia**	Female	5.1 (1.9-10)	9.1 (2.7-17.6)
**Oman**	**Leukemia**	Male	2.5 (1-4.7)	6 (1.7-12.7)
**Oman**	**Multiple myeloma**	Female	5.8 (1.7-11.6)	8.2 (2.5-16)
**Oman**	**Multiple myeloma**	Male	3.8 (1-8.1)	14.4 (4.5-25.5)
**Oman**	**Non-Hodgkin lymphoma**	Female	4.1 (0.7-8.6)	4 (1.2-8.6)
**Oman**	**Non-Hodgkin lymphoma**	Male	3.6 (1.2-7.3)	9.5 (2.7-18.2)
**Pakistan**	**Leukemia**	Female	1.6 (0.3-4.5)	6.7 (1.9-13.7)
**Pakistan**	**Leukemia**	Male	1.1 (0.2-2.9)	7.5 (2.1-14.9)
**Pakistan**	**Multiple myeloma**	Female	2.5 (0.5-6.2)	7 (2-14.2)
**Pakistan**	**Multiple myeloma**	Male	1.9 (0.3-5.4)	2.3 (0.6-5.4)
**Pakistan**	**Non-Hodgkin lymphoma**	Female	1.5 (0.2-4.2)	4.1 (1.1-8.8)
**Pakistan**	**Non-Hodgkin lymphoma**	Male	1.5 (0.3-4.1)	2.9 (0.8-6.6)

Countries	Causes	Sex	Percent change in 1990 (95% UI)	Percent change in 2019 (95% UI)
**Palau**	**Leukemia**	Female	11.1 (4.3-19.9)	7.2 (3.1-12.8)
**Palau**	**Leukemia**	Male	6.1 (2.8-10.2)	7.5 (3.3-12.9)
**Palau**	**Multiple myeloma**	Female	8.9 (3-16.7)	7.2 (3-13)
**Palau**	**Multiple myeloma**	Male	7.5 (2.1-15)	2.6 (1-5.2)
**Palau**	**Non-Hodgkin lymphoma**	Female	6.4 (1.2-12.9)	6.5 (2.6-11.6)
**Palau**	**Non-Hodgkin lymphoma**	Male	7.1 (2.6-13.6)	7.8 (3.5-13.3)
**Palestine**	**Leukemia**	Female	6.4 (2.5-12.3)	6.4 (2.6-11.6)
**Palestine**	**Leukemia**	Male	2.8 (1-5.6)	7.7 (3.3-13.3)
**Palestine**	**Multiple myeloma**	Female	7.6 (2.4-14.5)	10.2 (4.7-16.9)
**Palestine**	**Multiple myeloma**	Male	4.5 (1.2-10.3)	2 (0.7-4.1)
**Palestine**	**Non-Hodgkin lymphoma**	Female	4.7 (0.9-10)	7.7 (3.4-13.3)
**Palestine**	**Non-Hodgkin lymphoma**	Male	3.9 (1.2-8.3)	6 (2.4-10.9)
**Panama**	**Leukemia**	Female	3.6 (1-7.9)	4.2 (1.8-7.6)
**Panama**	**Leukemia**	Male	1.8 (0.4-4.1)	9.1 (4.1-15.5)
**Panama**	**Multiple myeloma**	Female	4.3 (1-9.4)	6.9 (3.1-12.1)
**Panama**	**Multiple myeloma**	Male	2.8 (0.5-7.3)	7.6 (3.3-13.2)
**Panama**	**Non-Hodgkin lymphoma**	Female	2.9 (0.4-6.9)	7.2 (3.2-12.5)
**Panama**	**Non-Hodgkin lymphoma**	Male	2.5 (0.5-6.3)	3.7 (1.4-7.3)
**Papua New Guinea**	**Leukemia**	Female	2.7 (0.7-6.2)	2.7 (0.9-5.5)
**Papua New Guinea**	**Leukemia**	Male	1.6 (0.4-3.7)	1.5 (0.5-3.4)
**Papua New Guinea**	**Multiple myeloma**	Female	4.1 (1-9.1)	2.1 (0.7-4.4)
**Papua New Guinea**	**Multiple myeloma**	Male	2.7 (0.5-7)	5.2 (2.2-9.1)
**Papua New Guinea**	**Non-Hodgkin lymphoma**	Female	2.5 (0.4-6.3)	1.5 (0.4-3.4)
**Papua New Guinea**	**Non-Hodgkin lymphoma**	Male	2.2 (0.5-5.5)	1.7 (0.6-3.5)
**Paraguay**	**Leukemia**	Female	4.6 (1.5-9.1)	3.6 (1.2-7.3)
**Paraguay**	**Leukemia**	Male	2.7 (1.1-5)	6.3 (2.6-11.2)
**Paraguay**	**Multiple myeloma**	Female	6.3 (1.8-12.6)	4.9 (2.2-8.9)
**Paraguay**	**Multiple myeloma**	Male	5.1 (1.4-11)	9.4 (4.3-15.7)
**Paraguay**	**Non-Hodgkin lymphoma**	Female	3.8 (0.7-8.2)	8.6 (3.8-14.5)
**Paraguay**	**Non-Hodgkin lymphoma**	Male	4.2 (1.4-8.6)	7 (3-12.4)
**Peru**	**Leukemia**	Female	4.1 (1.3-8.5)	9.3 (4.2-15.7)
**Peru**	**Leukemia**	Male	2.3 (0.9-4.6)	4.1 (1.7-7.4)
**Peru**	**Multiple myeloma**	Female	5.9 (1.7-11.9)	2.4 (0.9-4.7)
**Peru**	**Multiple myeloma**	Male	4.7 (1.2-10.3)	5.8 (2.4-10.6)
**Peru**	**Non-Hodgkin lymphoma**	Female	3.7 (0.6-8.2)	7.7 (3.3-13.2)
**Peru**	**Non-Hodgkin lymphoma**	Male	3.9 (1.3-8.4)	5.8 (2.4-10.6)
**Philippines**	**Leukemia**	Female	2.1 (0.6-4.7)	9 (4.1-15.1)
**Philippines**	**Leukemia**	Male	1.2 (0.4-2.6)	6.6 (2.7-11.7)
**Philippines**	**Multiple myeloma**	Female	2.8 (0.7-6)	2 (0.7-3.9)
**Philippines**	**Multiple myeloma**	Male	2.3 (0.6-5.4)	8.7 (3.9-14.9)
**Philippines**	**Non-Hodgkin lymphoma**	Female	1.7 (0.3-4.1)	2.4 (0.9-4.9)
**Philippines**	**Non-Hodgkin lymphoma**	Male	1.9 (0.6-4.3)	7.6 (3.4-12.8)
**Poland**	**Leukemia**	Female	10.7 (4.1-19.2)	6 (2.4-10.7)
**Poland**	**Leukemia**	Male	6 (2.8-10)	6.5 (2.8-11.4)

Countries	Causes	Sex	Percent change in 1990 (95% UI)	Percent change in 2019 (95% UI)
**Poland**	**Multiple myeloma**	Female	9.2 (3.1-17.3)	7.2 (3-12.6)
**Poland**	**Multiple myeloma**	Male	7.4 (2.1-14.7)	6.9 (2.9-12.5)
**Poland**	**Non-Hodgkin lymphoma**	Female	6.3 (1.1-12.8)	6.5 (2.6-11.8)
**Poland**	**Non-Hodgkin lymphoma**	Male	6.8 (2.5-13)	8.1 (3.5-14)
**Portugal**	**Leukemia**	Female	7 (2.4-13.8)	8.6 (3.8-14.8)
**Portugal**	**Leukemia**	Male	3.8 (1.4-7.3)	4.5 (1.7-8.4)
**Portugal**	**Multiple myeloma**	Female	5.9 (1.7-12)	2.8 (1-5.5)
**Portugal**	**Multiple myeloma**	Male	4.6 (1.2-10.3)	6.2 (2.6-11)
**Portugal**	**Non-Hodgkin lymphoma**	Female	4.2 (0.7-9.3)	5.5 (2.2-10.4)
**Portugal**	**Non-Hodgkin lymphoma**	Male	4.4 (1.4-9.3)	8.8 (4.1-14.8)
**Puerto Rico**	**Leukemia**	Female	10.8 (4.2-19.3)	3.1 (1.3-5.6)
**Puerto Rico**	**Leukemia**	Male	6.3 (2.7-10.6)	3.5 (1.4-6.6)
**Puerto Rico**	**Multiple myeloma**	Female	9.1 (3.1-16.9)	7.8 (3.4-13.2)
**Puerto Rico**	**Multiple myeloma**	Male	7.3 (2-14.7)	6.9 (2.8-12.6)
**Puerto Rico**	**Non-Hodgkin lymphoma**	Female	6.6 (1.2-13.4)	5.9 (2.4-10.8)
**Puerto Rico**	**Non-Hodgkin lymphoma**	Male	7.5 (2.7-14.4)	7.5 (3.3-12.8)
**Qatar**	**Leukemia**	Female	10.9 (4.7-18.6)	10.5 (5-17.1)
**Qatar**	**Leukemia**	Male	7.1 (3.4-11.5)	8.2 (3.7-14.1)
**Qatar**	**Multiple myeloma**	Female	12.4 (4.5-22.4)	5.8 (2.3-10.5)
**Qatar**	**Multiple myeloma**	Male	9.7 (2.8-18.6)	11.5 (5.4-18.4)
**Qatar**	**Non-Hodgkin lymphoma**	Female	8.2 (1.5-16)	2.9 (1.1-5.5)
**Qatar**	**Non-Hodgkin lymphoma**	Male	8.5 (3.2-15.5)	8.5 (3.6-14.9)
**Republic of Korea**	**Leukemia**	Female	3.1 (0.7-7.3)	5.3 (2.2-9.5)
**Republic of Korea**	**Leukemia**	Male	1.7 (0.4-3.8)	5.2 (2.2-9)
**Republic of Korea**	**Multiple myeloma**	Female	3.5 (0.8-8)	8.4 (3.6-14.6)
**Republic of Korea**	**Multiple myeloma**	Male	2.5 (0.4-6.7)	6.2 (2.6-10.9)
**Republic of Korea**	**Non-Hodgkin lymphoma**	Female	2.3 (0.3-5.7)	3 (1.1-6)
**Republic of Korea**	**Non-Hodgkin lymphoma**	Male	2.2 (0.5-5.7)	5.4 (2.4-9.3)
**Republic of Moldova**	**Leukemia**	Female	7.7 (3-14)	2.5 (0.9-4.9)
**Republic of Moldova**	**Leukemia**	Male	3 (1.2-5.5)	5.7 (2.3-10.4)
**Republic of Moldova**	**Multiple myeloma**	Female	8.7 (2.9-16.7)	6 (2.6-10.4)
**Republic of Moldova**	**Multiple myeloma**	Male	5.2 (1.4-11.2)	5.5 (2.3-9.8)
**Republic of Moldova**	**Non-Hodgkin lymphoma**	Female	5.5 (0.9-11.2)	7.2 (3.1-12.3)
**Republic of Moldova**	**Non-Hodgkin lymphoma**	Male	4 (1.4-8.1)	7.8 (3.2-13.9)
**Romania**	**Leukemia**	Female	8.9 (3.5-16)	9.3 (4.3-15.5)
**Romania**	**Leukemia**	Male	5 (2.4-8.4)	7.3 (3.1-12.7)
**Romania**	**Multiple myeloma**	Female	8.5 (2.9-15.9)	2.7 (0.9-5.4)
**Romania**	**Multiple myeloma**	Male	6.9 (2.1-13.7)	5 (2.3-8.6)
**Romania**	**Non-Hodgkin lymphoma**	Female	5.4 (1-10.9)	2.7 (0.9-5.4)
**Romania**	**Non-Hodgkin lymphoma**	Male	5.7 (2.1-10.9)	5.9 (2.3-10.8)
**Russian Federation**	**Leukemia**	Female	11.2 (4.4-19.6)	5.8 (2.4-10.4)
**Russian Federation**	**Leukemia**	Male	4.4 (2-7.7)	1.7 (0.6-3.6)
**Russian Federation**	**Multiple myeloma**	Female	9.8 (3.3-18.2)	7.8 (3.5-13.2)
**Russian Federation**	**Multiple myeloma**	Male	5.7 (1.7-11.9)	8.4 (3.8-14.3)

Countries	Causes	Sex	Percent change in 1990 (95% UI)	Percent change in 2019 (95% UI)
**Russian Federation**	**Non-Hodgkin lymphoma**	Female	6.5 (1.1-13.3)	5.3 (2.1-9.8)
**Russian Federation**	**Non-Hodgkin lymphoma**	Male	5 (1.8-9.5)	9.8 (4.6-16.4)
**Rwanda**	**Leukemia**	Female	1.4 (0.3-3.3)	9.2 (4.2-15.3)
**Rwanda**	**Leukemia**	Male	0.6 (0.1-1.4)	6.4 (2.8-11.3)
**Rwanda**	**Multiple myeloma**	Female	2.5 (0.5-6.1)	2.9 (0.9-6.2)
**Rwanda**	**Multiple myeloma**	Male	1.4 (0.2-4.2)	6.5 (2.8-11.6)
**Rwanda**	**Non-Hodgkin lymphoma**	Female	1.5 (0.2-4)	3.4 (1.4-6.1)
**Rwanda**	**Non-Hodgkin lymphoma**	Male	1.1 (0.2-3)	9 (4-15.2)
**Saint Kitts and Nevis**	**Leukemia**	Female	8.1 (3.1-14.8)	9.3 (4.1-15.4)
**Saint Kitts and Nevis**	**Leukemia**	Male	4 (1.6-7.3)	12.8 (6.4-20.2)
**Saint Kitts and Nevis**	**Multiple myeloma**	Female	7.1 (2.2-13.9)	7.9 (3.5-13.5)
**Saint Kitts and Nevis**	**Multiple myeloma**	Male	5 (1.4-10.9)	8.4 (3.9-13.9)
**Saint Kitts and Nevis**	**Non-Hodgkin lymphoma**	Female	5 (0.9-10.5)	2.7 (0.9-5.5)
**Saint Kitts and Nevis**	**Non-Hodgkin lymphoma**	Male	4.9 (1.7-10.1)	7.5 (3.2-13)
**Saint Lucia**	**Leukemia**	Female	6.6 (2.3-12.6)	6.7 (2.8-11.8)
**Saint Lucia**	**Leukemia**	Male	2.9 (1-5.6)	7.3 (3.1-12.5)
**Saint Lucia**	**Multiple myeloma**	Female	6.4 (2-12.9)	5.4 (2.3-9.7)
**Saint Lucia**	**Multiple myeloma**	Male	4.1 (1.1-9.6)	11.2 (5.4-18.2)
**Saint Lucia**	**Non-Hodgkin lymphoma**	Female	4.4 (0.8-9.4)	8.9 (4-15.1)
**Saint Lucia**	**Non-Hodgkin lymphoma**	Male	3.8 (1.2-8)	5.5 (2.3-9.8)
**Saint Vincent and the Grenadines**	**Leukemia**	Female	4.8 (1.6-9.8)	4.3 (1.7-8)
**Saint Vincent and the Grenadines**	**Leukemia**	Male	2.3 (0.8-4.7)	8.7 (3.9-14.9)
**Saint Vincent and the Grenadines**	**Multiple myeloma**	Female	5.2 (1.5-10.9)	4.5 (1.7-8.5)
**Saint Vincent and the Grenadines**	**Multiple myeloma**	Male	3.5 (0.8-8.4)	0.9 (0.2-2.4)
**Saint Vincent and the Grenadines**	**Non-Hodgkin lymphoma**	Female	3.4 (0.6-7.5)	3.4 (1.3-6.4)
**Saint Vincent and the Grenadines**	**Non-Hodgkin lymphoma**	Male	3.1 (0.9-7)	7 (2.9-12.4)
**Samoa**	**Leukemia**	Female	9.8 (3.8-17.4)	5.8 (2.5-10.4)
**Samoa**	**Leukemia**	Male	5.8 (2.6-9.5)	6.6 (2.7-11.7)
**Samoa**	**Multiple myeloma**	Female	8.8 (2.9-16.8)	5.7 (2.3-10.3)
**Samoa**	**Multiple myeloma**	Male	8.4 (2.4-16.4)	7.7 (3.3-13.1)
**Samoa**	**Non-Hodgkin lymphoma**	Female	5.9 (1.1-12.1)	3.1 (1.1-6)
**Samoa**	**Non-Hodgkin lymphoma**	Male	7.5 (2.8-13.7)	4.5 (1.8-8.3)
**San Marino**	**Leukemia**	Female	10.2 (3.8-18.7)	3.5 (1.4-6.5)
**San Marino**	**Leukemia**	Male	6.2 (2.6-10.6)	6.5 (2.6-11.6)
**San Marino**	**Multiple myeloma**	Female	7.8 (2.5-15.2)	8.3 (3.7-14.1)
**San Marino**	**Multiple myeloma**	Male	6.8 (1.9-14)	8.1 (3.4-13.9)
**San Marino**	**Non-Hodgkin lymphoma**	Female	5.8 (1-12.2)	8.1 (3.6-13.9)
**San Marino**	**Non-Hodgkin lymphoma**	Male	6.7 (2.3-13.4)	5.7 (2.3-10.3)
**Sao Tome and Principe**	**Leukemia**	Female	4.8 (1.6-9.7)	7.8 (3.5-13.4)
**Sao Tome and Principe**	**Leukemia**	Male	1.5 (0.4-3.5)	13.5 (6.4-21.8)
**Sao Tome and Principe**	**Multiple myeloma**	Female	5.2 (1.4-10.7)	3.4 (1.3-6.4)
**Sao Tome and Principe**	**Multiple myeloma**	Male	3 (0.6-7.5)	8.8 (3.9-14.8)
**Sao Tome and Principe**	**Non-Hodgkin lymphoma**	Female	3.1 (0.5-7.1)	6.5 (2.6-11.6)
**Sao Tome and Principe**	**Non-Hodgkin lymphoma**	Male	1.9 (0.5-4.7)	6.4 (2.8-11.4)

Countries	Causes	Sex	Percent change in 1990 (95% UI)	Percent change in 2019 (95% UI)
**Saudi Arabia**	**Leukemia**	Female	7.8 (3-14.4)	6.4 (2.7-11.5)
**Saudi Arabia**	**Leukemia**	Male	4.5 (2-7.8)	2.2 (0.7-4.5)
**Saudi Arabia**	**Multiple myeloma**	Female	8.7 (2.8-16.4)	3.5 (1.4-6.6)
**Saudi Arabia**	**Multiple myeloma**	Male	6 (1.7-12.4)	3.6 (1.4-6.8)
**Saudi Arabia**	**Non-Hodgkin lymphoma**	Female	5.4 (1-11.1)	6.8 (1.2-13.8)
**Saudi Arabia**	**Non-Hodgkin lymphoma**	Male	5.5 (2-10.7)	7.5 (1.3-14.8)
**Senegal**	**Leukemia**	Female	3.7 (1.3-7.3)	6.2 (1.1-12.8)
**Senegal**	**Leukemia**	Male	1.2 (0.3-2.6)	2.7 (0.4-5.8)
**Senegal**	**Multiple myeloma**	Female	4.4 (1.3-9.3)	6.1 (1.1-12.6)
**Senegal**	**Multiple myeloma**	Male	2.4 (0.6-5.9)	7.6 (1.3-15.3)
**Senegal**	**Non-Hodgkin lymphoma**	Female	2.6 (0.4-5.6)	5.9 (1.1-12.3)
**Senegal**	**Non-Hodgkin lymphoma**	Male	1.7 (0.4-3.9)	7.6 (1.4-15.3)
**Serbia**	**Leukemia**	Female	10.8 (4.2-19.4)	9.5 (1.7-18.6)
**Serbia**	**Leukemia**	Male	6.4 (2.8-10.6)	1.8 (0.3-4.2)
**Serbia**	**Multiple myeloma**	Female	9.2 (3.2-17.4)	7.6 (1.4-15.4)
**Serbia**	**Multiple myeloma**	Male	7.9 (2.2-15.7)	5.6 (1-11.6)
**Serbia**	**Non-Hodgkin lymphoma**	Female	6.3 (1.1-12.9)	4.7 (0.8-9.5)
**Serbia**	**Non-Hodgkin lymphoma**	Male	7.2 (2.6-13.7)	8.3 (1.5-16.3)
**Seychelles**	**Leukemia**	Female	6.6 (2.5-12.7)	6.7 (1.2-13.6)
**Seychelles**	**Leukemia**	Male	2.5 (0.8-5.2)	7 (1.3-14)
**Seychelles**	**Multiple myeloma**	Female	5.7 (1.7-11.2)	6.6 (1.2-13.3)
**Seychelles**	**Multiple myeloma**	Male	3 (0.7-7.2)	3.2 (0.5-7.2)
**Seychelles**	**Non-Hodgkin lymphoma**	Female	4.1 (0.7-8.7)	3.6 (0.6-7.8)
**Seychelles**	**Non-Hodgkin lymphoma**	Male	3.1 (0.9-6.8)	1.6 (0.3-3.9)
**Sierra Leone**	**Leukemia**	Female	2.2 (0.5-5.4)	2.1 (0.3-4.9)
**Sierra Leone**	**Leukemia**	Male	0.7 (0.1-1.9)	5.5 (1-11.2)
**Sierra Leone**	**Multiple myeloma**	Female	3 (0.7-6.9)	1.8 (0.3-4.1)
**Sierra Leone**	**Multiple myeloma**	Male	1.6 (0.2-4.5)	2.1 (0.3-4.8)
**Sierra Leone**	**Non-Hodgkin lymphoma**	Female	1.6 (0.2-4.2)	3.2 (0.5-7.5)
**Sierra Leone**	**Non-Hodgkin lymphoma**	Male	1.1 (0.2-3.1)	6.1 (1.1-12.7)
**Singapore**	**Leukemia**	Female	3.3 (0.7-7.7)	5 (0.9-10.1)
**Singapore**	**Leukemia**	Male	2 (0.5-4.4)	8 (1.4-15.8)
**Singapore**	**Multiple myeloma**	Female	3 (0.6-7.2)	7.5 (1.3-15.2)
**Singapore**	**Multiple myeloma**	Male	2.5 (0.5-6.6)	7.1 (1.3-14.5)
**Singapore**	**Non-Hodgkin lymphoma**	Female	2.1 (0.3-5.3)	8.1 (1.4-16.5)
**Singapore**	**Non-Hodgkin lymphoma**	Male	2.5 (0.6-6.1)	4.3 (0.7-9.2)
**Slovakia**	**Leukemia**	Female	11.2 (4.5-19.6)	2.1 (0.3-4.9)
**Slovakia**	**Leukemia**	Male	6.2 (2.9-10.4)	5.3 (0.9-11)
**Slovakia**	**Multiple myeloma**	Female	9.3 (3.1-17.3)	7.4 (1.3-15)
**Slovakia**	**Multiple myeloma**	Male	7.3 (2-14.6)	5.3 (0.9-11)
**Slovakia**	**Non-Hodgkin lymphoma**	Female	6.5 (1.2-13.1)	9.1 (1.6-17.4)
**Slovakia**	**Non-Hodgkin lymphoma**	Male	7 (2.5-13.3)	6.4 (1.1-13.1)
**Slovenia**	**Leukemia**	Female	11.1 (4.3-19.9)	2.4 (0.4-5.2)
**Slovenia**	**Leukemia**	Male	6.9 (3.2-11.3)	8.4 (1.5-16.7)

Countries	Causes	Sex	Percent change in 1990 (95% UI)	Percent change in 2019 (95% UI)
**Slovenia**	**Multiple myeloma**	Female	8.7 (3-16.5)	2.3 (0.4-5.2)
**Slovenia**	**Multiple myeloma**	Male	7.8 (2.3-15.5)	6.9 (1.2-13.5)
**Slovenia**	**Non-Hodgkin lymphoma**	Female	6.3 (1.1-12.9)	5.4 (1-11)
**Slovenia**	**Non-Hodgkin lymphoma**	Male	7.4 (2.8-14.1)	6.8 (1.2-13.6)
**Solomon Islands**	**Leukemia**	Female	5 (1.6-10.3)	6.8 (1.2-13.7)
**Solomon Islands**	**Leukemia**	Male	2.5 (0.7-5.2)	6.3 (1.1-13.1)
**Solomon Islands**	**Multiple myeloma**	Female	5.2 (1.3-11.1)	6 (1.1-12.7)
**Solomon Islands**	**Multiple myeloma**	Male	3.6 (0.8-8.6)	7.1 (1.3-14.4)
**Solomon Islands**	**Non-Hodgkin lymphoma**	Female	3.1 (0.5-6.8)	7.2 (1.3-14.7)
**Solomon Islands**	**Non-Hodgkin lymphoma**	Male	3 (0.8-6.8)	4.5 (0.8-9.6)
**Somalia**	**Leukemia**	Female	0.8 (0.1-2.2)	3.2 (0.5-7.1)
**Somalia**	**Leukemia**	Male	0.4 (0.1-1.1)	6.2 (1.1-12.6)
**Somalia**	**Multiple myeloma**	Female	1.6 (0.3-4.3)	5.5 (0.9-11.5)
**Somalia**	**Multiple myeloma**	Male	1 (0.1-3.3)	7.8 (1.4-15.5)
**Somalia**	**Non-Hodgkin lymphoma**	Female	1 (0.1-2.9)	2.9 (0.5-6.3)
**Somalia**	**Non-Hodgkin lymphoma**	Male	0.8 (0.1-2.5)	3.7 (0.6-7.8)
**South Africa**	**Leukemia**	Female	10.9 (4.4-19.1)	6.9 (1.2-13.9)
**South Africa**	**Leukemia**	Male	3.6 (1.6-6.3)	6.1 (1.1-12.9)
**South Africa**	**Multiple myeloma**	Female	10.7 (3.7-19.7)	5.5 (1-11.5)
**South Africa**	**Multiple myeloma**	Male	5.3 (1.5-10.9)	7.7 (1.4-15.4)
**South Africa**	**Non-Hodgkin lymphoma**	Female	6.8 (1.3-13.7)	9.6 (1.8-18.8)
**South Africa**	**Non-Hodgkin lymphoma**	Male	4.6 (1.7-8.9)	8.2 (1.4-16.3)
**South Sudan**	**Leukemia**	Female	1.3 (0.3-3.2)	5.1 (0.9-10.7)
**South Sudan**	**Leukemia**	Male	0.8 (0.2-2)	10.8 (2-20.5)
**South Sudan**	**Multiple myeloma**	Female	3.8 (1.1-8.1)	2.8 (0.5-6.1)
**South Sudan**	**Multiple myeloma**	Male	2.4 (0.6-5.8)	8.5 (1.5-17)
**South Sudan**	**Non-Hodgkin lymphoma**	Female	2.1 (0.3-5)	7.2 (1.3-14.4)
**South Sudan**	**Non-Hodgkin lymphoma**	Male	1.9 (0.5-4.4)	5.3 (0.9-10.8)
**Spain**	**Leukemia**	Female	9.4 (3.5-17.1)	8.1 (1.4-16.5)
**Spain**	**Leukemia**	Male	5.1 (2.1-9)	5.6 (0.9-11.5)
**Spain**	**Multiple myeloma**	Female	7.7 (2.5-14.7)	2.9 (0.4-6.5)
**Spain**	**Multiple myeloma**	Male	6 (1.6-12.6)	4.9 (0.9-9.9)
**Spain**	**Non-Hodgkin lymphoma**	Female	5.6 (1-11.5)	3.1 (0.5-6.8)
**Spain**	**Non-Hodgkin lymphoma**	Male	5.8 (2-11.6)	5.5 (1-11.4)
**Sri Lanka**	**Leukemia**	Female	3.2 (1-6.6)	6.1 (1.1-12.1)
**Sri Lanka**	**Leukemia**	Male	1.3 (0.4-2.7)	5.5 (1-11.4)
**Sri Lanka**	**Multiple myeloma**	Female	3.2 (0.8-6.8)	6.6 (1.2-13.3)
**Sri Lanka**	**Multiple myeloma**	Male	1.9 (0.4-4.6)	7.1 (1.2-14.5)
**Sri Lanka**	**Non-Hodgkin lymphoma**	Female	2.2 (0.3-5)	8.1 (1.4-15.9)
**Sri Lanka**	**Non-Hodgkin lymphoma**	Male	1.8 (0.5-4.1)	7.2 (1.2-14.6)
**Sudan**	**Leukemia**	Female	3 (0.9-7.1)	2.9 (0.5-6.4)
**Sudan**	**Leukemia**	Male	1.5 (0.5-3.4)	5.7 (1-11.4)
**Sudan**	**Multiple myeloma**	Female	4.9 (1.4-10.2)	2.5 (0.4-5.8)
**Sudan**	**Multiple myeloma**	Male	2.7 (0.6-6.4)	5.5 (1-11.6)

Countries	Causes	Sex	Percent change in 1990 (95% UI)	Percent change in 2019 (95% UI)
**Sudan**	**Non-Hodgkin lymphoma**	Female	2.8 (0.5-6.4)	6 (1-12.4)
**Sudan**	**Non-Hodgkin lymphoma**	Male	2.1 (0.6-4.9)	2.2 (0.4-5.1)
**Suriname**	**Leukemia**	Female	6 (2.1-11.7)	6.8 (1.2-13.7)
**Suriname**	**Leukemia**	Male	3 (1.1-5.6)	7.4 (1.3-14.9)
**Suriname**	**Multiple myeloma**	Female	6.6 (2-13.1)	4.8 (0.8-10.1)
**Suriname**	**Multiple myeloma**	Male	4.5 (1.2-10.2)	9.2 (1.7-18.1)
**Suriname**	**Non-Hodgkin lymphoma**	Female	4.3 (0.7-9.2)	7.7 (1.4-15.3)
**Suriname**	**Non-Hodgkin lymphoma**	Male	4 (1.3-8.5)	6.3 (1.1-12.7)
**Sweden**	**Leukemia**	Female	8.1 (2.9-15.7)	3 (0.5-7)
**Sweden**	**Leukemia**	Male	4.8 (1.9-8.7)	5.8 (1-12)
**Sweden**	**Multiple myeloma**	Female	6.1 (1.8-12.3)	3.2 (0.5-6.8)
**Sweden**	**Multiple myeloma**	Male	5.2 (1.4-11.2)	8 (1.4-15.9)
**Sweden**	**Non-Hodgkin lymphoma**	Female	4.6 (0.8-9.8)	8.3 (1.5-16.5)
**Sweden**	**Non-Hodgkin lymphoma**	Male	5.2 (1.8-10.7)	11.4 (2.1-21.7)
**Switzerland**	**Leukemia**	Female	7.9 (3-14.8)	8 (1.5-15.8)
**Switzerland**	**Leukemia**	Male	4.9 (2-8.8)	7.4 (1.3-14.6)
**Switzerland**	**Multiple myeloma**	Female	6.1 (1.9-12.1)	3.2 (0.5-7.1)
**Switzerland**	**Multiple myeloma**	Male	5.6 (1.6-12)	7.1 (1.3-14.3)
**Switzerland**	**Non-Hodgkin lymphoma**	Female	4.5 (0.8-9.3)	6.5 (1.1-13.2)
**Switzerland**	**Non-Hodgkin lymphoma**	Male	5.6 (2-11.2)	6.3 (1.2-12.8)
**Syrian Arab Republic**	**Leukemia**	Female	8.3 (3.1-15.5)	5.4 (0.9-10.9)
**Syrian Arab Republic**	**Leukemia**	Male	3.6 (1.5-6.6)	10.6 (2-20.3)
**Syrian Arab Republic**	**Multiple myeloma**	Female	8.6 (2.8-16.6)	7.7 (1.4-15.3)
**Syrian Arab Republic**	**Multiple myeloma**	Male	5.6 (1.6-11.9)	5.2 (0.9-10.9)
**Syrian Arab Republic**	**Non-Hodgkin lymphoma**	Female	5.5 (1-11.4)	3.7 (0.6-8.2)
**Syrian Arab Republic**	**Non-Hodgkin lymphoma**	Male	4.8 (1.7-9.7)	8 (1.5-15.8)
**Taiwan (Province of China)**	**Leukemia**	Female	4 (1.2-8.4)	4.4 (0.7-9.4)
**Taiwan (Province of China)**	**Leukemia**	Male	2.5 (0.8-5)	1.1 (0.2-3)
**Taiwan (Province of China)**	**Multiple myeloma**	Female	4.2 (1.1-9)	3.6 (0.7-7.7)
**Taiwan (Province of China)**	**Multiple myeloma**	Male	3.3 (0.8-7.8)	6.5 (1.2-13.4)
**Taiwan (Province of China)**	**Non-Hodgkin lymphoma**	Female	2.7 (0.5-6.2)	5.7 (1-11.4)
**Taiwan (Province of China)**	**Non-Hodgkin lymphoma**	Male	3 (0.9-6.6)	6.5 (1.1-13.2)
**Tajikistan**	**Leukemia**	Female	2.5 (0.7-5.3)	5.2 (0.9-10.7)
**Tajikistan**	**Leukemia**	Male	1 (0.3-2.3)	7.5 (1.3-14.9)
**Tajikistan**	**Multiple myeloma**	Female	4.1 (1.1-8.7)	3.3 (0.6-7.3)
**Tajikistan**	**Multiple myeloma**	Male	2.3 (0.5-6)	4.4 (0.8-9.3)
**Tajikistan**	**Non-Hodgkin lymphoma**	Female	2.4 (0.4-5.6)	4.2 (0.7-8.6)
**Tajikistan**	**Non-Hodgkin lymphoma**	Male	2 (0.5-4.7)	5.9 (1-12.2)
**Thailand**	**Leukemia**	Female	2.8 (0.8-6.4)	8.2 (1.5-15.9)
**Thailand**	**Leukemia**	Male	1.3 (0.3-2.9)	7.8 (1.4-15.7)
**Thailand**	**Multiple myeloma**	Female	2.6 (0.6-6.2)	7.5 (1.3-15.1)
**Thailand**	**Multiple myeloma**	Male	1.8 (0.3-4.7)	5 (0.9-10.7)
**Thailand**	**Non-Hodgkin lymphoma**	Female	1.9 (0.3-4.6)	8.1 (1.5-16.1)
**Thailand**	**Non-Hodgkin lymphoma**	Male	1.8 (0.4-4.3)	10.8 (2-20.8)

Countries	Causes	Sex	Percent change in 1990 (95% UI)	Percent change in 2019 (95% UI)
**Timor-Leste**	**Leukemia**	Female	0.9 (0.1-2.8)	3.5 (0.6-7.3)
**Timor-Leste**	**Leukemia**	Male	0.4 (0-1.3)	7.8 (1.4-15.6)
**Timor-Leste**	**Multiple myeloma**	Female	1.4 (0.2-3.8)	6.2 (1.1-12.7)
**Timor-Leste**	**Multiple myeloma**	Male	0.9 (0.1-2.9)	6.3 (1.1-12.7)
**Timor-Leste**	**Non-Hodgkin lymphoma**	Female	0.8 (0.1-2.3)	6 (1-12.4)
**Timor-Leste**	**Non-Hodgkin lymphoma**	Male	0.7 (0.1-2.1)	2.1 (0.3-4.9)
**Togo**	**Leukemia**	Female	2.8 (0.9-6.2)	3.5 (0.6-7.7)
**Togo**	**Leukemia**	Male	0.8 (0.2-2)	5.1 (0.9-10.3)
**Togo**	**Multiple myeloma**	Female	3.6 (1-7.8)	7.6 (2.8-14.3)
**Togo**	**Multiple myeloma**	Male	1.8 (0.4-4.8)	7.6 (2.9-14.2)
**Togo**	**Non-Hodgkin lymphoma**	Female	2 (0.3-4.6)	7.5 (2.6-14.5)
**Togo**	**Non-Hodgkin lymphoma**	Male	1.2 (0.3-3.1)	2.6 (0.8-5.6)
**Tokelau**	**Leukemia**	Female	6.9 (2.4-13.5)	6.8 (2.4-13.1)
**Tokelau**	**Leukemia**	Male	3.4 (1.1-6.5)	8 (3-14.6)
**Tokelau**	**Multiple myeloma**	Female	6.1 (1.7-12.4)	6.9 (2.4-13.6)
**Tokelau**	**Multiple myeloma**	Male	4.3 (1-9.9)	7.7 (2.8-14.4)
**Tokelau**	**Non-Hodgkin lymphoma**	Female	4 (0.7-8.8)	10.6 (4.1-18.6)
**Tokelau**	**Non-Hodgkin lymphoma**	Male	4.1 (1.2-9)	2.2 (0.7-4.7)
**Tonga**	**Leukemia**	Female	10.3 (4.1-17.8)	7.8 (3-14.5)
**Tonga**	**Leukemia**	Male	4.5 (2-8)	6.4 (2.3-12.6)
**Tonga**	**Multiple myeloma**	Female	9.5 (3.3-17.8)	4 (1.4-8.2)
**Tonga**	**Multiple myeloma**	Male	6.3 (1.8-12.8)	9.7 (3.6-17.8)
**Tonga**	**Non-Hodgkin lymphoma**	Female	5.7 (1-11.7)	7.1 (2.7-13.4)
**Tonga**	**Non-Hodgkin lymphoma**	Male	6.1 (2.1-11.7)	8.2 (3-15.3)
**Trinidad and Tobago**	**Leukemia**	Female	7.8 (3-14.2)	7.8 (2.9-14.6)
**Trinidad and Tobago**	**Leukemia**	Male	4 (1.8-6.9)	4.1 (1.3-8.5)
**Trinidad and Tobago**	**Multiple myeloma**	Female	8.5 (2.8-16.2)	2.2 (0.6-5.1)
**Trinidad and Tobago**	**Multiple myeloma**	Male	5.9 (1.7-12.3)	1.5 (0.4-3.4)
**Trinidad and Tobago**	**Non-Hodgkin lymphoma**	Female	5.6 (1-11.5)	2.1 (0.6-4.6)
**Trinidad and Tobago**	**Non-Hodgkin lymphoma**	Male	5.6 (2-10.9)	5 (1.8-9.6)
**Tunisia**	**Leukemia**	Female	5.9 (2.2-11.4)	1.4 (0.4-3.4)
**Tunisia**	**Leukemia**	Male	3.4 (1.4-6.3)	1.5 (0.4-3.5)
**Tunisia**	**Multiple myeloma**	Female	7.9 (2.6-15.1)	3.8 (1.2-8.2)
**Tunisia**	**Multiple myeloma**	Male	5.1 (1.4-11.1)	6.4 (2.3-12.4)
**Tunisia**	**Non-Hodgkin lymphoma**	Female	5.4 (1-11.1)	4.9 (1.8-9.5)
**Tunisia**	**Non-Hodgkin lymphoma**	Male	4.8 (1.7-9.5)	10.4 (4-18.6)
**Turkey**	**Leukemia**	Female	7.8 (3-14.5)	9.6 (3.6-17.5)
**Turkey**	**Leukemia**	Male	3.8 (1.6-6.8)	7 (2.5-13.5)
**Turkey**	**Multiple myeloma**	Female	9.9 (3.4-18.4)	10.3 (3.9-18.7)
**Turkey**	**Multiple myeloma**	Male	6.3 (1.7-13)	4 (1.5-8)
**Turkey**	**Non-Hodgkin lymphoma**	Female	6.2 (1.1-12.8)	2.6 (0.8-5.4)
**Turkey**	**Non-Hodgkin lymphoma**	Male	5.3 (1.9-10.7)	6.3 (2.2-12.5)
**Turkmenistan**	**Leukemia**	Female	3.9 (1.4-7.6)	7.8 (2.9-14.7)
**Turkmenistan**	**Leukemia**	Male	1.8 (0.8-3.4)	6.2 (2.3-12.1)

Countries	Causes	Sex	Percent change in 1990 (95% UI)	Percent change in 2019 (95% UI)
**Turkmenistan**	**Multiple myeloma**	Female	7.5 (2.4-14.7)	9 (3.5-16.3)
**Turkmenistan**	**Multiple myeloma**	Male	5.4 (1.5-11.5)	6.7 (2.4-13.1)
**Turkmenistan**	**Non-Hodgkin lymphoma**	Female	4.7 (0.8-9.8)	1.6 (0.5-3.5)
**Turkmenistan**	**Non-Hodgkin lymphoma**	Male	4.2 (1.5-8.5)	9.1 (3.5-16.3)
**Tuvalu**	**Leukemia**	Female	5.6 (1.8-11.5)	2.4 (0.7-5.3)
**Tuvalu**	**Leukemia**	Male	2.9 (0.9-5.8)	8.1 (3-14.7)
**Tuvalu**	**Multiple myeloma**	Female	5.3 (1.4-11.2)	6.4 (2.3-12.6)
**Tuvalu**	**Multiple myeloma**	Male	4 (0.9-9.4)	6.3 (2.3-12)
**Tuvalu**	**Non-Hodgkin lymphoma**	Female	3.5 (0.5-8)	7.6 (2.8-14.5)
**Tuvalu**	**Non-Hodgkin lymphoma**	Male	3.7 (1-8.2)	7.5 (2.7-14.4)
**Uganda**	**Leukemia**	Female	0.8 (0.2-2.2)	6.9 (2.4-13.6)
**Uganda**	**Leukemia**	Male	0.5 (0.1-1.4)	8.6 (3.1-16.2)
**Uganda**	**Multiple myeloma**	Female	2 (0.4-4.9)	9.4 (3.5-17.2)
**Uganda**	**Multiple myeloma**	Male	1.4 (0.2-4.1)	4.4 (1.5-9.1)
**Uganda**	**Non-Hodgkin lymphoma**	Female	1.2 (0.2-3.2)	2.4 (0.7-5.3)
**Uganda**	**Non-Hodgkin lymphoma**	Male	1.1 (0.2-3)	6.1 (2.2-12)
**Ukraine**	**Leukemia**	Female	10.8 (4.4-19.1)	5.6 (2-11.2)
**Ukraine**	**Leukemia**	Male	4.7 (2.3-7.9)	9.9 (3.8-17.7)
**Ukraine**	**Multiple myeloma**	Female	10.4 (3.6-19.2)	3.1 (1.1-6.1)
**Ukraine**	**Multiple myeloma**	Male	6.8 (2-13.6)	3.4 (1.2-6.8)
**Ukraine**	**Non-Hodgkin lymphoma**	Female	7 (1.2-14.1)	8.4 (3.2-15.3)
**Ukraine**	**Non-Hodgkin lymphoma**	Male	5.7 (2.1-10.9)	7.6 (2.7-14.7)
**United Arab Emirates**	**Leukemia**	Female	12 (5-20.7)	6.3 (2.2-12.7)
**United Arab Emirates**	**Leukemia**	Male	6.9 (3.3-11.4)	7.3 (2.7-13.8)
**United Arab Emirates**	**Multiple myeloma**	Female	11.1 (3.9-20.3)	11.3 (4.4-19.7)
**United Arab Emirates**	**Multiple myeloma**	Male	9.3 (2.6-17.8)	8.2 (3.1-15.1)
**United Arab Emirates**	**Non-Hodgkin lymphoma**	Female	6.7 (1.1-13.5)	6.3 (2.2-12.1)
**United Arab Emirates**	**Non-Hodgkin lymphoma**	Male	8.2 (3.1-15.1)	11.8 (4.6-20.3)
**United Kingdom**	**Leukemia**	Female	9.6 (3.6-18)	3 (1-6)
**United Kingdom**	**Leukemia**	Male	6 (2.6-10.4)	8.7 (3.3-15.9)
**United Kingdom**	**Multiple myeloma**	Female	7.3 (2.2-14.3)	4.2 (1.5-8.5)
**United Kingdom**	**Multiple myeloma**	Male	6.7 (1.8-13.7)	5.1 (1.8-9.9)
**United Kingdom**	**Non-Hodgkin lymphoma**	Female	5.5 (1-11.6)	8.9 (3.3-16.7)
**United Kingdom**	**Non-Hodgkin lymphoma**	Male	6.7 (2.3-13.1)	6.7 (2.5-13)
**United Republic of Tanzania**	**Leukemia**	Female	1.5 (0.4-3.4)	3.1 (1-6.5)
**United Republic of Tanzania**	**Leukemia**	Male	0.8 (0.2-1.8)	5.7 (2.1-10.7)
**United Republic of Tanzania**	**Multiple myeloma**	Female	3.8 (1.1-8.2)	2.2 (0.7-4.9)
**United Republic of Tanzania**	**Multiple myeloma**	Male	2.3 (0.5-5.7)	5.9 (2.1-11.7)
**United Republic of Tanzania**	**Non-Hodgkin lymphoma**	Female	2.1 (0.4-5)	5.8 (2.2-11)
**United Republic of Tanzania**	**Non-Hodgkin lymphoma**	Male	1.8 (0.5-4.2)	5.5 (2-10.3)
**United States of America**	**Leukemia**	Female	10.9 (4.2-19.3)	7.6 (2.8-14.2)
**United States of America**	**Leukemia**	Male	6 (2.5-10.4)	8.5 (2.9-16)
**United States of America**	**Multiple myeloma**	Female	8.3 (2.7-15.7)	10.3 (4-18.6)
**United States of America**	**Multiple myeloma**	Male	6.6 (1.8-13.6)	7.4 (2.7-14.3)

Countries	Causes	Sex	Percent change in 1990 (95% UI)	Percent change in 2019 (95% UI)
**United States of America**	**Non-Hodgkin lymphoma**	Female	6.1 (1.1-12.4)	2.4 (0.7-5.3)
**United States of America**	**Non-Hodgkin lymphoma**	Male	6.7 (2.3-13.1)	4.6 (1.7-8.6)
**United States Virgin Islands**	**Leukemia**	Female	11.5 (4.6-19.9)	2.9 (0.9-6.2)
**United States Virgin Islands**	**Leukemia**	Male	5 (2.2-8.7)	6.2 (2.2-12.7)
**United States Virgin Islands**	**Multiple myeloma**	Female	9.5 (3.2-17.9)	5.7 (2.1-11)
**United States Virgin Islands**	**Multiple myeloma**	Male	6.7 (1.8-13.8)	1.4 (0.4-3.3)
**United States Virgin Islands**	**Non-Hodgkin lymphoma**	Female	6.9 (1.2-13.7)	8.7 (3.3-16.1)
**United States Virgin Islands**	**Non-Hodgkin lymphoma**	Male	6.2 (2.2-12)	9.1 (3.4-16.6)
**Uruguay**	**Leukemia**	Female	7 (2.4-13.4)	5.7 (2-11.2)
**Uruguay**	**Leukemia**	Male	3.8 (1.4-7.2)	10.3 (4-18.4)
**Uruguay**	**Multiple myeloma**	Female	5.9 (1.7-12)	9.8 (3.8-17.6)
**Uruguay**	**Multiple myeloma**	Male	4.6 (1.2-10.2)	6.6 (2.4-12.5)
**Uruguay**	**Non-Hodgkin lymphoma**	Female	4.3 (0.7-9.3)	2.8 (0.8-6.5)
**Uruguay**	**Non-Hodgkin lymphoma**	Male	4.5 (1.5-9.6)	7 (2.6-13.5)
**Uzbekistan**	**Leukemia**	Female	4.7 (1.7-8.8)	3.5 (1.2-6.8)
**Uzbekistan**	**Leukemia**	Male	2.4 (1-4.5)	9.8 (3.8-17.9)
**Uzbekistan**	**Multiple myeloma**	Female	6.7 (2.1-13.4)	10.1 (3.8-18.2)
**Uzbekistan**	**Multiple myeloma**	Male	5 (1.3-10.7)	13.4 (5.6-22.6)
**Uzbekistan**	**Non-Hodgkin lymphoma**	Female	4 (0.7-8.7)	7.8 (2.9-14.6)
**Uzbekistan**	**Non-Hodgkin lymphoma**	Male	3.8 (1.3-7.8)	9.3 (3.6-16.5)
**Vanuatu**	**Leukemia**	Female	5.9 (2.1-11.5)	2.2 (0.6-5)
**Vanuatu**	**Leukemia**	Male	2.7 (0.8-5.6)	7.7 (2.9-14.6)
**Vanuatu**	**Multiple myeloma**	Female	6.2 (1.7-12.6)	6.8 (2.4-13)
**Vanuatu**	**Multiple myeloma**	Male	3.7 (0.8-9)	8.2 (3-15.1)
**Vanuatu**	**Non-Hodgkin lymphoma**	Female	3.9 (0.6-8.6)	5.4 (1.9-10.6)
**Vanuatu**	**Non-Hodgkin lymphoma**	Male	3.5 (0.9-7.9)	11.7 (4.7-20.4)
**Venezuela (Bolivarian Republic of)**	**Leukemia**	Female	5.3 (1.9-10.1)	9.9 (3.8-17.9)
**Venezuela (Bolivarian Republic of)**	**Leukemia**	Male	2.6 (1-4.9)	5.7 (2-11)
**Venezuela (Bolivarian Republic of)**	**Multiple myeloma**	Female	7 (2.2-13.9)	4.7 (1.6-9.4)
**Venezuela (Bolivarian Republic of)**	**Multiple myeloma**	Male	5.3 (1.4-11.4)	9.4 (3.5-17.3)
**Venezuela (Bolivarian Republic of)**	**Non-Hodgkin lymphoma**	Female	4.4 (0.8-9.2)	4.6 (1.5-9.5)
**Venezuela (Bolivarian Republic of)**	**Non-Hodgkin lymphoma**	Male	4.3 (1.4-8.8)	0.8 (0.1-2.4)
**Viet Nam**	**Leukemia**	Female	1.1 (0.2-3.1)	3.3 (1.1-6.6)
**Viet Nam**	**Leukemia**	Male	0.4 (0.1-1.3)	7.4 (2.6-14.3)
**Viet Nam**	**Multiple myeloma**	Female	1.1 (0.2-2.9)	5.9 (2.2-11.4)
**Viet Nam**	**Multiple myeloma**	Male	0.7 (0.1-2.2)	6.6 (2.3-12.8)
**Viet Nam**	**Non-Hodgkin lymphoma**	Female	0.7 (0.1-2.2)	6.1 (2.1-12.3)
**Viet Nam**	**Non-Hodgkin lymphoma**	Male	0.6 (0.1-1.9)	7.8 (2.9-14.7)

Countries	Causes	Sex	Percent change in 1990 (95% UI)	Percent change in 2019 (95% UI)
**Yemen**	**Leukemia**	Female	2.1 (0.6-5)	2.9 (0.9-6.2)
**Yemen**	**Leukemia**	Male	1.1 (0.3-2.5)	4.6 (1.6-9)
**Yemen**	**Multiple myeloma**	Female	3.2 (0.8-7.1)	3 (1-6.2)
**Yemen**	**Multiple myeloma**	Male	1.8 (0.3-4.8)	7.2 (2.6-13.9)
**Yemen**	**Non-Hodgkin lymphoma**	Female	1.9 (0.3-4.7)	8.4 (3.2-15.6)
**Yemen**	**Non-Hodgkin lymphoma**	Male	1.5 (0.3-3.9)	8.2 (3.1-15.6)
**Zambia**	**Leukemia**	Female	1.3 (0.4-3.1)	8.6 (3.2-15.7)
**Zambia**	**Leukemia**	Male	0.7 (0.2-1.8)	6.3 (2.2-12.2)
**Zambia**	**Multiple myeloma**	Female	3.3 (0.8-7.2)	7.6 (2.9-14.3)
**Zambia**	**Multiple myeloma**	Male	2.3 (0.5-5.8)	14 (5.9-23.4)
**Zambia**	**Non-Hodgkin lymphoma**	Female	1.8 (0.3-4.4)	3.3 (1.1-6.6)
**Zambia**	**Non-Hodgkin lymphoma**	Male	1.7 (0.4-4.2)	9.5 (3.6-17.5)
**Zimbabwe**	**Leukemia**	Female	5.9 (2.3-11)	6.7 (2.4-13.2)
**Zimbabwe**	**Leukemia**	Male	1.7 (0.6-3.3)	6.6 (2.4-12.5)
**Zimbabwe**	**Multiple myeloma**	Female	6 (1.9-11.8)	6.7 (2.5-13)
**Zimbabwe**	**Multiple myeloma**	Male	2.4 (0.6-5.7)	2.3 (0.7-5)
**Zimbabwe**	**Non-Hodgkin lymphoma**	Female	4.1 (0.7-8.8)	3.5 (1.2-7.2)
**Zimbabwe**	**Non-Hodgkin lymphoma**	Male	2.2 (0.6-4.9)	2.6 (0.8-5.6)

Abbreviations: UI, uncertainty interval.

Supplementary Table S9. The percent change in death cases attributable to occupational carcinogens between 1990 and 2019 at national level.

Countries	Causes	Occupational carcinogens	Percent change in 1990 (95% UI)	Percent change in 2019 (95% UI)
**Afghanistan**	**All leukemia**	Benzene	0.33 (0.1-0.56)	0.19 (0.14-0.28)
**Afghanistan**	**All leukemia**	Formaldehyde	0.12 (0.09-0.15)	0.02 (0.01-0.02)
**Afghanistan**	**Acute myeloid leukemia**	Benzene	0.44 (0.13-0.77)	0.05 (0.04-0.06)
**Afghanistan**	**Acute myeloid leukemia**	Formaldehyde	0.16 (0.11-0.22)	0.26 (0.2-0.33)
**Afghanistan**	**Acute lymphoid leukemia**	Benzene	0.3 (0.08-0.57)	0.02 (0.01-0.03)
**Afghanistan**	**Acute lymphoid leukemia**	Formaldehyde	0.11 (0.06-0.16)	0.17 (0.13-0.22)
**Afghanistan**	**Chronic myeloid leukemia**	Benzene	0.41 (0.12-0.76)	0.16 (0.12-0.19)
**Afghanistan**	**Chronic myeloid leukemia**	Formaldehyde	0.15 (0.1-0.22)	0.11 (0.09-0.14)
**Afghanistan**	**Chronic lymphoid leukemia**	Benzene	0.38 (0.12-0.67)	0.11 (0.09-0.14)
**Afghanistan**	**Chronic lymphoid leukemia**	Formaldehyde	0.13 (0.09-0.19)	0.02 (0.01-0.03)
**Afghanistan**	**Other leukemia**	Benzene	0.29 (0.08-0.49)	0.02 (0.01-0.02)
**Afghanistan**	**Other leukemia**	Formaldehyde	0.1 (0.07-0.14)	0.15 (0.12-0.19)
**Albania**	**All leukemia**	Benzene	0.27 (0.05-0.49)	0.15 (0.12-0.18)
**Albania**	**All leukemia**	Formaldehyde	0.03 (0.02-0.03)	0.12 (0.09-0.15)
**Albania**	**Acute myeloid leukemia**	Benzene	0.32 (0.06-0.59)	0.1 (0.08-0.13)
**Albania**	**Acute myeloid leukemia**	Formaldehyde	0.03 (0.02-0.04)	0.11 (0.09-0.14)
**Albania**	**Acute lymphoid leukemia**	Benzene	0.24 (0.04-0.46)	0.03 (0.02-0.03)
**Albania**	**Acute lymphoid leukemia**	Formaldehyde	0.02 (0.01-0.03)	0.01 (0.01-0.02)
**Albania**	**Chronic myeloid leukemia**	Benzene	0.34 (0.06-0.64)	0.23 (0.18-0.28)
**Albania**	**Chronic myeloid leukemia**	Formaldehyde	0.03 (0.02-0.05)	0.12 (0.09-0.16)
**Albania**	**Chronic lymphoid leukemia**	Benzene	0.19 (0.04-0.35)	0.1 (0.07-0.13)
**Albania**	**Chronic lymphoid leukemia**	Formaldehyde	0.02 (0.01-0.02)	0.09 (0.07-0.12)
**Albania**	**Other leukemia**	Benzene	0.25 (0.05-0.47)	0.21 (0.16-0.25)
**Albania**	**Other leukemia**	Formaldehyde	0.03 (0.02-0.03)	0.02 (0.01-0.02)
**Algeria**	**All leukemia**	Benzene	0.36 (0.11-0.61)	0.1 (0.07-0.13)
**Algeria**	**All leukemia**	Formaldehyde	0.12 (0.09-0.15)	0.11 (0.09-0.14)
**Algeria**	**Acute myeloid leukemia**	Benzene	0.38 (0.11-0.68)	0.04 (0.03-0.05)
**Algeria**	**Acute myeloid leukemia**	Formaldehyde	0.13 (0.09-0.18)	0.03 (0.02-0.03)
**Algeria**	**Acute lymphoid leukemia**	Benzene	0.2 (0.06-0.4)	0.06 (0.04-0.07)
**Algeria**	**Acute lymphoid leukemia**	Formaldehyde	0.07 (0.04-0.11)	0.15 (0.12-0.19)
**Algeria**	**Chronic myeloid leukemia**	Benzene	0.51 (0.14-0.89)	0.05 (0.03-0.07)
**Algeria**	**Chronic myeloid leukemia**	Formaldehyde	0.17 (0.12-0.23)	0.2 (0.15-0.25)
**Algeria**	**Chronic lymphoid leukemia**	Benzene	0.11 (0.03-0.2)	0.1 (0.07-0.13)
**Algeria**	**Chronic lymphoid leukemia**	Formaldehyde	0.04 (0.03-0.05)	0.02 (0.01-0.03)
**Algeria**	**Other leukemia**	Benzene	0.36 (0.11-0.61)	0.12 (0.09-0.16)
**Algeria**	**Other leukemia**	Formaldehyde	0.12 (0.09-0.16)	0.07 (0.05-0.09)
**American Samoa**	**All leukemia**	Benzene	0.7 (0.21-1.16)	0.09 (0.07-0.12)
**American Samoa**	**All leukemia**	Formaldehyde	0.45 (0.34-0.57)	0.27 (0.23-0.32)
**American Samoa**	**Acute myeloid leukemia**	Benzene	0.88 (0.26-1.45)	0.16 (0.13-0.2)
**American Samoa**	**Acute myeloid leukemia**	Formaldehyde	0.54 (0.4-0.69)	0.11 (0.08-0.14)
**American Samoa**	**Acute lymphoid leukemia**	Benzene	0.63 (0.19-1.09)	0.13 (0.09-0.17)
**American Samoa**	**Acute lymphoid leukemia**	Formaldehyde	0.42 (0.29-0.58)	0.17 (0.13-0.21)

Countries	Causes	Occupational carcinogens	Percent change in 1990 (95% UI)	Percent change in 2019 (95% UI)
**American Samoa**	**Chronic myeloid leukemia**	Benzene	0.8 (0.23-1.32)	0.09 (0.07-0.12)
**American Samoa**	**Chronic myeloid leukemia**	Formaldehyde	0.51 (0.39-0.66)	0.01 (0.01-0.02)
**American Samoa**	**Chronic lymphoid leukemia**	Benzene	0.67 (0.19-1.13)	0.12 (0.1-0.15)
**American Samoa**	**Chronic lymphoid leukemia**	Formaldehyde	0.46 (0.35-0.59)	0.02 (0.01-0.02)
**American Samoa**	**Other leukemia**	Benzene	0.54 (0.16-0.9)	0.03 (0.02-0.03)
**American Samoa**	**Other leukemia**	Formaldehyde	0.35 (0.27-0.45)	0.09 (0.06-0.11)
**Andorra**	**All leukemia**	Benzene	0.46 (0.09-0.84)	0.36 (0.27-0.46)
**Andorra**	**All leukemia**	Formaldehyde	0.06 (0.05-0.07)	0.14 (0.1-0.18)
**Andorra**	**Acute myeloid leukemia**	Benzene	0.5 (0.09-0.91)	0.02 (0.01-0.03)
**Andorra**	**Acute myeloid leukemia**	Formaldehyde	0.06 (0.05-0.08)	0.1 (0.07-0.13)
**Andorra**	**Acute lymphoid leukemia**	Benzene	0.69 (0.14-1.29)	0.1 (0.08-0.13)
**Andorra**	**Acute lymphoid leukemia**	Formaldehyde	0.09 (0.06-0.11)	0.18 (0.14-0.23)
**Andorra**	**Chronic myeloid leukemia**	Benzene	0.58 (0.1-1.1)	0.19 (0.15-0.24)
**Andorra**	**Chronic myeloid leukemia**	Formaldehyde	0.07 (0.05-0.09)	0.17 (0.12-0.23)
**Andorra**	**Chronic lymphoid leukemia**	Benzene	0.22 (0.04-0.41)	0.18 (0.14-0.22)
**Andorra**	**Chronic lymphoid leukemia**	Formaldehyde	0.03 (0.02-0.04)	0.09 (0.07-0.13)
**Andorra**	**Other leukemia**	Benzene	0.38 (0.07-0.69)	0.14 (0.11-0.18)
**Andorra**	**Other leukemia**	Formaldehyde	0.05 (0.04-0.06)	0.02 (0.01-0.02)
**Angola**	**All leukemia**	Benzene	0.56 (0.15-1.15)	0.07 (0.05-0.1)
**Angola**	**All leukemia**	Formaldehyde	0.22 (0.12-0.36)	0.11 (0.09-0.14)
**Angola**	**Acute myeloid leukemia**	Benzene	0.76 (0.18-1.47)	0.16 (0.12-0.21)
**Angola**	**Acute myeloid leukemia**	Formaldehyde	0.3 (0.13-0.46)	0.02 (0.01-0.03)
**Angola**	**Acute lymphoid leukemia**	Benzene	0.36 (0.08-0.81)	0.01 (0.01-0.02)
**Angola**	**Acute lymphoid leukemia**	Formaldehyde	0.14 (0.05-0.27)	0.06 (0.04-0.08)
**Angola**	**Chronic myeloid leukemia**	Benzene	0.8 (0.18-1.67)	0.09 (0.07-0.11)
**Angola**	**Chronic myeloid leukemia**	Formaldehyde	0.31 (0.11-0.49)	0.1 (0.08-0.13)
**Angola**	**Chronic lymphoid leukemia**	Benzene	0.55 (0.16-0.94)	0.02 (0.02-0.03)
**Angola**	**Chronic lymphoid leukemia**	Formaldehyde	0.21 (0.16-0.26)	0.11 (0.08-0.15)
**Angola**	**Other leukemia**	Benzene	0.54 (0.13-1.18)	0.01 (0.01-0.01)
**Angola**	**Other leukemia**	Formaldehyde	0.21 (0.1-0.37)	0.03 (0.02-0.03)
**Antigua and Barbuda**	**All leukemia**	Benzene	0.96 (0.28-1.56)	0.18 (0.14-0.22)
**Antigua and Barbuda**	**All leukemia**	Formaldehyde	0.24 (0.2-0.29)	0.2 (0.16-0.25)
**Antigua and Barbuda**	**Acute myeloid leukemia**	Benzene	1.23 (0.36-2.04)	0.27 (0.22-0.33)
**Antigua and Barbuda**	**Acute myeloid leukemia**	Formaldehyde	0.31 (0.25-0.39)	0.07 (0.05-0.09)
**Antigua and Barbuda**	**Acute lymphoid leukemia**	Benzene	0.8 (0.23-1.34)	0.08 (0.06-0.11)
**Antigua and Barbuda**	**Acute lymphoid leukemia**	Formaldehyde	0.2 (0.15-0.27)	0.17 (0.14-0.21)
**Antigua and Barbuda**	**Chronic myeloid leukemia**	Benzene	1.04 (0.31-1.73)	0.15 (0.11-0.18)
**Antigua and Barbuda**	**Chronic myeloid leukemia**	Formaldehyde	0.26 (0.22-0.33)	0.18 (0.13-0.23)
**Antigua and Barbuda**	**Chronic lymphoid leukemia**	Benzene	0.68 (0.2-1.14)	0.02 (0.02-0.03)
**Antigua and Barbuda**	**Chronic lymphoid leukemia**	Formaldehyde	0.18 (0.13-0.22)	0.02 (0.02-0.03)
**Antigua and Barbuda**	**Other leukemia**	Benzene	0.89 (0.26-1.46)	0.07 (0.06-0.09)
**Antigua and Barbuda**	**Other leukemia**	Formaldehyde	0.22 (0.18-0.27)	0.17 (0.14-0.21)
**Argentina**	**All leukemia**	Benzene	0.73 (0.21-1.2)	0.14 (0.11-0.18)
**Argentina**	**All leukemia**	Formaldehyde	0.27 (0.23-0.32)	0.12 (0.09-0.16)

Countries	Causes	Occupational carcinogens	Percent change in 1990 (95% UI)	Percent change in 2019 (95% UI)
**Argentina**	**Acute myeloid leukemia**	Benzene	0.87 (0.26-1.46)	0.02 (0.01-0.02)
**Argentina**	**Acute myeloid leukemia**	Formaldehyde	0.31 (0.26-0.38)	0.02 (0.01-0.03)
**Argentina**	**Acute lymphoid leukemia**	Benzene	0.68 (0.2-1.13)	0.01 (0.01-0.02)
**Argentina**	**Acute lymphoid leukemia**	Formaldehyde	0.25 (0.2-0.31)	0.13 (0.1-0.16)
**Argentina**	**Chronic myeloid leukemia**	Benzene	0.84 (0.24-1.41)	0.02 (0.02-0.03)
**Argentina**	**Chronic myeloid leukemia**	Formaldehyde	0.31 (0.25-0.38)	0.13 (0.1-0.18)
**Argentina**	**Chronic lymphoid leukemia**	Benzene	0.36 (0.11-0.6)	0.16 (0.13-0.2)
**Argentina**	**Chronic lymphoid leukemia**	Formaldehyde	0.14 (0.11-0.18)	0.18 (0.15-0.22)
**Argentina**	**Other leukemia**	Benzene	0.72 (0.21-1.18)	0.15 (0.11-0.19)
**Argentina**	**Other leukemia**	Formaldehyde	0.27 (0.22-0.32)	0.17 (0.13-0.22)
**Armenia**	**All leukemia**	Benzene	0.57 (0.17-0.96)	0.2 (0.16-0.24)
**Armenia**	**All leukemia**	Formaldehyde	0.19 (0.15-0.23)	0.24 (0.18-0.32)
**Armenia**	**Acute myeloid leukemia**	Benzene	0.54 (0.15-0.95)	0.01 (0.01-0.02)
**Armenia**	**Acute myeloid leukemia**	Formaldehyde	0.18 (0.13-0.25)	0.1 (0.08-0.13)
**Armenia**	**Acute lymphoid leukemia**	Benzene	0.55 (0.15-0.94)	0.1 (0.07-0.13)
**Armenia**	**Acute lymphoid leukemia**	Formaldehyde	0.17 (0.13-0.22)	0.09 (0.07-0.12)
**Armenia**	**Chronic myeloid leukemia**	Benzene	0.66 (0.19-1.12)	0.13 (0.1-0.17)
**Armenia**	**Chronic myeloid leukemia**	Formaldehyde	0.22 (0.17-0.3)	0.02 (0.01-0.02)
**Armenia**	**Chronic lymphoid leukemia**	Benzene	0.46 (0.13-0.77)	0.01 (0.01-0.02)
**Armenia**	**Chronic lymphoid leukemia**	Formaldehyde	0.15 (0.11-0.19)	0.14 (0.11-0.18)
**Armenia**	**Other leukemia**	Benzene	0.63 (0.19-1.04)	0.19 (0.15-0.24)
**Armenia**	**Other leukemia**	Formaldehyde	0.21 (0.16-0.26)	0.13 (0.1-0.16)
**Australia**	**All leukemia**	Benzene	0.34 (0.06-0.6)	0.17 (0.13-0.23)
**Australia**	**All leukemia**	Formaldehyde	0.04 (0.03-0.05)	0.1 (0.08-0.13)
**Australia**	**Acute myeloid leukemia**	Benzene	0.38 (0.07-0.67)	0.02 (0.01-0.02)
**Australia**	**Acute myeloid leukemia**	Formaldehyde	0.05 (0.04-0.05)	0.18 (0.14-0.23)
**Australia**	**Acute lymphoid leukemia**	Benzene	0.5 (0.09-0.88)	0.04 (0.03-0.06)
**Australia**	**Acute lymphoid leukemia**	Formaldehyde	0.06 (0.05-0.07)	0.17 (0.14-0.22)
**Australia**	**Chronic myeloid leukemia**	Benzene	0.36 (0.07-0.65)	0.19 (0.16-0.23)
**Australia**	**Chronic myeloid leukemia**	Formaldehyde	0.04 (0.04-0.05)	0.24 (0.17-0.31)
**Australia**	**Chronic lymphoid leukemia**	Benzene	0.17 (0.03-0.31)	0.02 (0.01-0.03)
**Australia**	**Chronic lymphoid leukemia**	Formaldehyde	0.02 (0.02-0.03)	0.21 (0.15-0.29)
**Australia**	**Other leukemia**	Benzene	0.24 (0.04-0.44)	0.02 (0.01-0.02)
**Australia**	**Other leukemia**	Formaldehyde	0.03 (0.02-0.03)	0.14 (0.11-0.17)
**Austria**	**All leukemia**	Benzene	0.28 (0.05-0.49)	0.09 (0.07-0.11)
**Austria**	**All leukemia**	Formaldehyde	0.04 (0.03-0.05)	0.15 (0.12-0.19)
**Austria**	**Acute myeloid leukemia**	Benzene	0.35 (0.06-0.62)	0.06 (0.04-0.08)
**Austria**	**Acute myeloid leukemia**	Formaldehyde	0.05 (0.04-0.06)	0.35 (0.26-0.44)
**Austria**	**Acute lymphoid leukemia**	Benzene	0.49 (0.09-0.88)	0.11 (0.09-0.15)
**Austria**	**Acute lymphoid leukemia**	Formaldehyde	0.07 (0.06-0.09)	0.02 (0.02-0.03)
**Austria**	**Chronic myeloid leukemia**	Benzene	0.32 (0.06-0.56)	0.02 (0.02-0.03)
**Austria**	**Chronic myeloid leukemia**	Formaldehyde	0.04 (0.04-0.05)	0.23 (0.18-0.27)
**Austria**	**Chronic lymphoid leukemia**	Benzene	0.15 (0.03-0.26)	0.08 (0.06-0.11)
**Austria**	**Chronic lymphoid leukemia**	Formaldehyde	0.02 (0.02-0.03)	0.08 (0.06-0.11)

Countries	Causes	Occupational carcinogens	Percent change in 1990 (95% UI)	Percent change in 2019 (95% UI)
**Austria**	**Other leukemia**	Benzene	0.26 (0.05-0.45)	0.14 (0.1-0.18)
**Austria**	**Other leukemia**	Formaldehyde	0.04 (0.03-0.04)	0.02 (0.02-0.03)
**Azerbaijan**	**All leukemia**	Benzene	0.59 (0.17-0.98)	0.31 (0.23-0.39)
**Azerbaijan**	**All leukemia**	Formaldehyde	0.22 (0.18-0.28)	0.02 (0.01-0.02)
**Azerbaijan**	**Acute myeloid leukemia**	Benzene	0.74 (0.2-1.28)	0.17 (0.12-0.24)
**Azerbaijan**	**Acute myeloid leukemia**	Formaldehyde	0.28 (0.2-0.36)	0.1 (0.08-0.13)
**Azerbaijan**	**Acute lymphoid leukemia**	Benzene	0.48 (0.13-0.86)	0.21 (0.16-0.27)
**Azerbaijan**	**Acute lymphoid leukemia**	Formaldehyde	0.18 (0.1-0.25)	0.09 (0.07-0.13)
**Azerbaijan**	**Chronic myeloid leukemia**	Benzene	0.77 (0.21-1.38)	0.12 (0.1-0.15)
**Azerbaijan**	**Chronic myeloid leukemia**	Formaldehyde	0.29 (0.2-0.4)	0.21 (0.15-0.28)
**Azerbaijan**	**Chronic lymphoid leukemia**	Benzene	0.63 (0.17-1.06)	0.15 (0.12-0.18)
**Azerbaijan**	**Chronic lymphoid leukemia**	Formaldehyde	0.23 (0.17-0.3)	0.29 (0.23-0.36)
**Azerbaijan**	**Other leukemia**	Benzene	0.57 (0.16-0.97)	0.25 (0.2-0.29)
**Azerbaijan**	**Other leukemia**	Formaldehyde	0.22 (0.17-0.28)	0.02 (0.02-0.03)
**Bahamas**	**All leukemia**	Benzene	1.08 (0.33-1.77)	0.02 (0.01-0.02)
**Bahamas**	**All leukemia**	Formaldehyde	0.24 (0.2-0.3)	0.01 (0.01-0.02)
**Bahamas**	**Acute myeloid leukemia**	Benzene	1.31 (0.39-2.16)	0.2 (0.15-0.25)
**Bahamas**	**Acute myeloid leukemia**	Formaldehyde	0.3 (0.24-0.37)	0.05 (0.04-0.06)
**Bahamas**	**Acute lymphoid leukemia**	Benzene	0.76 (0.23-1.29)	0.01 (0.01-0.02)
**Bahamas**	**Acute lymphoid leukemia**	Formaldehyde	0.18 (0.13-0.23)	0.03 (0.02-0.03)
**Bahamas**	**Chronic myeloid leukemia**	Benzene	1.25 (0.39-2.07)	0.02 (0.02-0.03)
**Bahamas**	**Chronic myeloid leukemia**	Formaldehyde	0.29 (0.24-0.35)	0.09 (0.07-0.12)
**Bahamas**	**Chronic lymphoid leukemia**	Benzene	0.6 (0.18-1)	0.19 (0.14-0.25)
**Bahamas**	**Chronic lymphoid leukemia**	Formaldehyde	0.13 (0.11-0.16)	0.13 (0.1-0.16)
**Bahamas**	**Other leukemia**	Benzene	1.06 (0.32-1.78)	0.14 (0.11-0.16)
**Bahamas**	**Other leukemia**	Formaldehyde	0.24 (0.2-0.3)	0.12 (0.09-0.16)
**Bahrain**	**All leukemia**	Benzene	1.05 (0.31-1.75)	0.03 (0.02-0.03)
**Bahrain**	**All leukemia**	Formaldehyde	0.31 (0.24-0.4)	0.05 (0.04-0.07)
**Bahrain**	**Acute myeloid leukemia**	Benzene	1.1 (0.32-1.98)	0.2 (0.14-0.26)
**Bahrain**	**Acute myeloid leukemia**	Formaldehyde	0.32 (0.23-0.49)	0.06 (0.04-0.08)
**Bahrain**	**Acute lymphoid leukemia**	Benzene	0.8 (0.25-1.43)	0.03 (0.02-0.04)
**Bahrain**	**Acute lymphoid leukemia**	Formaldehyde	0.24 (0.16-0.36)	0.18 (0.15-0.23)
**Bahrain**	**Chronic myeloid leukemia**	Benzene	1.33 (0.38-2.25)	0.06 (0.05-0.08)
**Bahrain**	**Chronic myeloid leukemia**	Formaldehyde	0.41 (0.3-0.55)	0.03 (0.02-0.04)
**Bahrain**	**Chronic lymphoid leukemia**	Benzene	0.29 (0.08-0.48)	0.03 (0.02-0.03)
**Bahrain**	**Chronic lymphoid leukemia**	Formaldehyde	0.06 (0.05-0.08)	0.02 (0.01-0.02)
**Bahrain**	**Other leukemia**	Benzene	1.11 (0.31-1.91)	0.45 (0.32-0.64)
**Bahrain**	**Other leukemia**	Formaldehyde	0.34 (0.23-0.47)	0.12 (0.09-0.15)
**Bangladesh**	**All leukemia**	Benzene	0.31 (0.08-0.6)	0.06 (0.05-0.08)
**Bangladesh**	**All leukemia**	Formaldehyde	0.14 (0.09-0.21)	0.11 (0.08-0.14)
**Bangladesh**	**Acute myeloid leukemia**	Benzene	0.35 (0.1-0.67)	0.01 (0.01-0.02)
**Bangladesh**	**Acute myeloid leukemia**	Formaldehyde	0.16 (0.1-0.24)	0.18 (0.14-0.22)
**Bangladesh**	**Acute lymphoid leukemia**	Benzene	0.21 (0.05-0.44)	0.21 (0.16-0.27)
**Bangladesh**	**Acute lymphoid leukemia**	Formaldehyde	0.09 (0.05-0.16)	0.15 (0.12-0.19)

Countries	Causes	Occupational carcinogens	Percent change in 1990 (95% UI)	Percent change in 2019 (95% UI)
**Bangladesh**	**Chronic myeloid leukemia**	Benzene	0.47 (0.13-0.95)	0.02 (0.01-0.02)
**Bangladesh**	**Chronic myeloid leukemia**	Formaldehyde	0.21 (0.12-0.34)	0.02 (0.01-0.02)
**Bangladesh**	**Chronic lymphoid leukemia**	Benzene	0.18 (0.05-0.31)	0.1 (0.07-0.14)
**Bangladesh**	**Chronic lymphoid leukemia**	Formaldehyde	0.09 (0.07-0.11)	0.14 (0.1-0.17)
**Bangladesh**	**Other leukemia**	Benzene	0.27 (0.07-0.54)	0.12 (0.09-0.16)
**Bangladesh**	**Other leukemia**	Formaldehyde	0.12 (0.07-0.19)	0.11 (0.08-0.14)
**Barbados**	**All leukemia**	Benzene	0.75 (0.22-1.25)	0.15 (0.11-0.2)
**Barbados**	**All leukemia**	Formaldehyde	0.23 (0.19-0.27)	0.09 (0.06-0.12)
**Barbados**	**Acute myeloid leukemia**	Benzene	0.98 (0.29-1.63)	0.19 (0.14-0.25)
**Barbados**	**Acute myeloid leukemia**	Formaldehyde	0.3 (0.25-0.36)	0.18 (0.13-0.23)
**Barbados**	**Acute lymphoid leukemia**	Benzene	0.69 (0.22-1.15)	0.19 (0.14-0.23)
**Barbados**	**Acute lymphoid leukemia**	Formaldehyde	0.21 (0.17-0.27)	0.1 (0.08-0.13)
**Barbados**	**Chronic myeloid leukemia**	Benzene	0.81 (0.23-1.38)	0.11 (0.09-0.14)
**Barbados**	**Chronic myeloid leukemia**	Formaldehyde	0.25 (0.2-0.31)	0.16 (0.12-0.2)
**Barbados**	**Chronic lymphoid leukemia**	Benzene	0.38 (0.11-0.65)	0.21 (0.16-0.27)
**Barbados**	**Chronic lymphoid leukemia**	Formaldehyde	0.12 (0.09-0.14)	0.1 (0.08-0.13)
**Barbados**	**Other leukemia**	Benzene	0.65 (0.19-1.08)	0.02 (0.02-0.03)
**Barbados**	**Other leukemia**	Formaldehyde	0.2 (0.17-0.24)	0.33 (0.24-0.47)
**Belarus**	**All leukemia**	Benzene	0.38 (0.07-0.67)	0.02 (0.01-0.02)
**Belarus**	**All leukemia**	Formaldehyde	0.06 (0.05-0.07)	0.12 (0.09-0.15)
**Belarus**	**Acute myeloid leukemia**	Benzene	0.44 (0.08-0.78)	0.02 (0.02-0.03)
**Belarus**	**Acute myeloid leukemia**	Formaldehyde	0.07 (0.06-0.08)	0.13 (0.1-0.17)
**Belarus**	**Acute lymphoid leukemia**	Benzene	0.31 (0.06-0.57)	0.08 (0.06-0.11)
**Belarus**	**Acute lymphoid leukemia**	Formaldehyde	0.05 (0.04-0.07)	0.19 (0.15-0.23)
**Belarus**	**Chronic myeloid leukemia**	Benzene	0.48 (0.09-0.86)	0.26 (0.19-0.33)
**Belarus**	**Chronic myeloid leukemia**	Formaldehyde	0.08 (0.06-0.09)	0.15 (0.12-0.19)
**Belarus**	**Chronic lymphoid leukemia**	Benzene	0.21 (0.04-0.38)	0.13 (0.09-0.18)
**Belarus**	**Chronic lymphoid leukemia**	Formaldehyde	0.04 (0.03-0.05)	0.13 (0.09-0.2)
**Belarus**	**Other leukemia**	Benzene	0.33 (0.06-0.58)	0.13 (0.1-0.17)
**Belarus**	**Other leukemia**	Formaldehyde	0.05 (0.04-0.06)	0.12 (0.09-0.16)
**Belgium**	**All leukemia**	Benzene	0.24 (0.05-0.42)	0.23 (0.16-0.34)
**Belgium**	**All leukemia**	Formaldehyde	0.03 (0.02-0.03)	0.04 (0.02-0.05)
**Belgium**	**Acute myeloid leukemia**	Benzene	0.3 (0.05-0.54)	0.21 (0.16-0.26)
**Belgium**	**Acute myeloid leukemia**	Formaldehyde	0.04 (0.03-0.04)	0.31 (0.24-0.4)
**Belgium**	**Acute lymphoid leukemia**	Benzene	0.39 (0.07-0.7)	0.05 (0.03-0.06)
**Belgium**	**Acute lymphoid leukemia**	Formaldehyde	0.05 (0.04-0.06)	0.38 (0.28-0.5)
**Belgium**	**Chronic myeloid leukemia**	Benzene	0.25 (0.05-0.45)	0.23 (0.19-0.29)
**Belgium**	**Chronic myeloid leukemia**	Formaldehyde	0.03 (0.03-0.04)	0.24 (0.19-0.29)
**Belgium**	**Chronic lymphoid leukemia**	Benzene	0.12 (0.02-0.22)	0.2 (0.15-0.25)
**Belgium**	**Chronic lymphoid leukemia**	Formaldehyde	0.02 (0.01-0.02)	0.03 (0.03-0.04)
**Belgium**	**Other leukemia**	Benzene	0.22 (0.04-0.38)	0.03 (0.03-0.04)
**Belgium**	**Other leukemia**	Formaldehyde	0.03 (0.02-0.03)	0.24 (0.18-0.34)
**Belize**	**All leukemia**	Benzene	0.52 (0.15-0.87)	0.3 (0.25-0.36)
**Belize**	**All leukemia**	Formaldehyde	0.16 (0.13-0.19)	0.45 (0.32-0.6)

Countries	Causes	Occupational carcinogens	Percent change in 1990 (95% UI)	Percent change in 2019 (95% UI)
**Belize**	**Acute myeloid leukemia**	Benzene	0.76 (0.22-1.38)	0.3 (0.22-0.41)
**Belize**	**Acute myeloid leukemia**	Formaldehyde	0.23 (0.16-0.31)	0.21 (0.17-0.25)
**Belize**	**Acute lymphoid leukemia**	Benzene	0.35 (0.1-0.63)	0.06 (0.04-0.07)
**Belize**	**Acute lymphoid leukemia**	Formaldehyde	0.1 (0.07-0.15)	0.02 (0.02-0.03)
**Belize**	**Chronic myeloid leukemia**	Benzene	0.8 (0.24-1.36)	0.45 (0.35-0.58)
**Belize**	**Chronic myeloid leukemia**	Formaldehyde	0.25 (0.18-0.33)	0.52 (0.37-0.71)
**Belize**	**Chronic lymphoid leukemia**	Benzene	0.54 (0.16-0.92)	0.17 (0.13-0.21)
**Belize**	**Chronic lymphoid leukemia**	Formaldehyde	0.17 (0.13-0.21)	0.26 (0.19-0.33)
**Belize**	**Other leukemia**	Benzene	0.54 (0.16-0.91)	0.34 (0.26-0.42)
**Belize**	**Other leukemia**	Formaldehyde	0.16 (0.13-0.2)	0.03 (0.02-0.03)
**Benin**	**All leukemia**	Benzene	0.39 (0.11-0.7)	0.26 (0.17-0.37)
**Benin**	**All leukemia**	Formaldehyde	0.15 (0.11-0.21)	0.25 (0.21-0.3)
**Benin**	**Acute myeloid leukemia**	Benzene	0.35 (0.09-0.69)	0.09 (0.07-0.11)
**Benin**	**Acute myeloid leukemia**	Formaldehyde	0.14 (0.07-0.22)	0.04 (0.03-0.05)
**Benin**	**Acute lymphoid leukemia**	Benzene	0.26 (0.07-0.52)	0.24 (0.17-0.33)
**Benin**	**Acute lymphoid leukemia**	Formaldehyde	0.1 (0.05-0.18)	0.33 (0.24-0.43)
**Benin**	**Chronic myeloid leukemia**	Benzene	0.9 (0.26-1.52)	0.2 (0.12-0.3)
**Benin**	**Chronic myeloid leukemia**	Formaldehyde	0.36 (0.25-0.49)	0.34 (0.26-0.43)
**Benin**	**Chronic lymphoid leukemia**	Benzene	0.22 (0.06-0.38)	0.45 (0.31-0.65)
**Benin**	**Chronic lymphoid leukemia**	Formaldehyde	0.09 (0.06-0.12)	0.04 (0.03-0.05)
**Benin**	**Other leukemia**	Benzene	0.38 (0.11-0.65)	0.25 (0.17-0.34)
**Benin**	**Other leukemia**	Formaldehyde	0.15 (0.1-0.21)	0.28 (0.19-0.39)
**Bermuda**	**All leukemia**	Benzene	0.94 (0.27-1.56)	0.22 (0.18-0.27)
**Bermuda**	**All leukemia**	Formaldehyde	0.24 (0.2-0.28)	0.34 (0.28-0.41)
**Bermuda**	**Acute myeloid leukemia**	Benzene	1.2 (0.35-2.03)	0.32 (0.26-0.4)
**Bermuda**	**Acute myeloid leukemia**	Formaldehyde	0.3 (0.25-0.37)	0.35 (0.26-0.45)
**Bermuda**	**Acute lymphoid leukemia**	Benzene	0.99 (0.3-1.68)	0.32 (0.24-0.43)
**Bermuda**	**Acute lymphoid leukemia**	Formaldehyde	0.25 (0.19-0.31)	0.23 (0.17-0.3)
**Bermuda**	**Chronic myeloid leukemia**	Benzene	1.03 (0.31-1.73)	0.23 (0.19-0.28)
**Bermuda**	**Chronic myeloid leukemia**	Formaldehyde	0.28 (0.22-0.35)	0.03 (0.02-0.03)
**Bermuda**	**Chronic lymphoid leukemia**	Benzene	0.54 (0.16-0.91)	0.21 (0.17-0.26)
**Bermuda**	**Chronic lymphoid leukemia**	Formaldehyde	0.15 (0.11-0.18)	0.04 (0.03-0.05)
**Bermuda**	**Other leukemia**	Benzene	0.84 (0.25-1.4)	0.06 (0.04-0.07)
**Bermuda**	**Other leukemia**	Formaldehyde	0.21 (0.17-0.25)	0.41 (0.29-0.56)
**Bhutan**	**All leukemia**	Benzene	0.49 (0.13-0.91)	0.37 (0.28-0.51)
**Bhutan**	**All leukemia**	Formaldehyde	0.15 (0.09-0.21)	0.35 (0.26-0.45)
**Bhutan**	**Acute myeloid leukemia**	Benzene	0.57 (0.16-1.03)	0.04 (0.03-0.05)
**Bhutan**	**Acute myeloid leukemia**	Formaldehyde	0.17 (0.11-0.24)	0.24 (0.16-0.32)
**Bhutan**	**Acute lymphoid leukemia**	Benzene	0.36 (0.08-0.73)	0.21 (0.16-0.27)
**Bhutan**	**Acute lymphoid leukemia**	Formaldehyde	0.11 (0.04-0.19)	0.37 (0.28-0.49)
**Bhutan**	**Chronic myeloid leukemia**	Benzene	0.68 (0.18-1.25)	0.39 (0.32-0.48)
**Bhutan**	**Chronic myeloid leukemia**	Formaldehyde	0.21 (0.11-0.29)	0.28 (0.2-0.38)
**Bhutan**	**Chronic lymphoid leukemia**	Benzene	0.29 (0.08-0.49)	0.3 (0.24-0.39)
**Bhutan**	**Chronic lymphoid leukemia**	Formaldehyde	0.09 (0.07-0.12)	0.28 (0.2-0.38)

Countries	Causes	Occupational carcinogens	Percent change in 1990 (95% UI)	Percent change in 2019 (95% UI)
**Bhutan**	**Other leukemia**	Benzene	0.41 (0.1-0.8)	0.37 (0.27-0.48)
**Bhutan**	**Other leukemia**	Formaldehyde	0.12 (0.06-0.2)	0.03 (0.02-0.04)
**Bolivia (Plurinational State of)**	**All leukemia**	Benzene	0.5 (0.15-0.86)	0.22 (0.15-0.32)
**Bolivia (Plurinational State of)**	**All leukemia**	Formaldehyde	0.17 (0.13-0.21)	0.31 (0.21-0.42)
**Bolivia (Plurinational State of)**	**Acute myeloid leukemia**	Benzene	0.57 (0.17-1.01)	0.22 (0.17-0.28)
**Bolivia (Plurinational State of)**	**Acute myeloid leukemia**	Formaldehyde	0.19 (0.14-0.25)	0.04 (0.03-0.05)
**Bolivia (Plurinational State of)**	**Acute lymphoid leukemia**	Benzene	0.31 (0.09-0.58)	0.02 (0.01-0.02)
**Bolivia (Plurinational State of)**	**Acute lymphoid leukemia**	Formaldehyde	0.1 (0.07-0.15)	0.19 (0.14-0.25)
**Bolivia (Plurinational State of)**	**Chronic myeloid leukemia**	Benzene	0.74 (0.21-1.28)	0.38 (0.27-0.53)
**Bolivia (Plurinational State of)**	**Chronic myeloid leukemia**	Formaldehyde	0.25 (0.18-0.32)	0.22 (0.17-0.27)
**Bolivia (Plurinational State of)**	**Chronic lymphoid leukemia**	Benzene	0.53 (0.15-0.89)	0.03 (0.03-0.04)
**Bolivia (Plurinational State of)**	**Chronic lymphoid leukemia**	Formaldehyde	0.18 (0.14-0.22)	0.55 (0.38-0.77)
**Bolivia (Plurinational State of)**	**Other leukemia**	Benzene	0.58 (0.16-1.01)	0.01 (0.01-0.02)
**Bolivia (Plurinational State of)**	**Other leukemia**	Formaldehyde	0.19 (0.15-0.25)	0.04 (0.03-0.05)
**Bosnia and Herzegovina**	**All leukemia**	Benzene	0.23 (0.04-0.42)	0.29 (0.24-0.35)
**Bosnia and Herzegovina**	**All leukemia**	Formaldehyde	0.04 (0.03-0.04)	0.23 (0.18-0.29)
**Bosnia and Herzegovina**	**Acute myeloid leukemia**	Benzene	0.28 (0.05-0.51)	0.34 (0.28-0.42)
**Bosnia and Herzegovina**	**Acute myeloid leukemia**	Formaldehyde	0.04 (0.03-0.05)	0.26 (0.18-0.36)
**Bosnia and Herzegovina**	**Acute lymphoid leukemia**	Benzene	0.26 (0.05-0.49)	0.4 (0.28-0.55)
**Bosnia and Herzegovina**	**Acute lymphoid leukemia**	Formaldehyde	0.04 (0.03-0.05)	0.34 (0.27-0.42)
**Bosnia and Herzegovina**	**Chronic myeloid leukemia**	Benzene	0.24 (0.04-0.45)	0.23 (0.15-0.32)
**Bosnia and Herzegovina**	**Chronic myeloid leukemia**	Formaldehyde	0.04 (0.03-0.05)	0.34 (0.25-0.45)
**Bosnia and Herzegovina**	**Chronic lymphoid leukemia**	Benzene	0.13 (0.02-0.24)	0.04 (0.04-0.05)
**Bosnia and Herzegovina**	**Chronic lymphoid leukemia**	Formaldehyde	0.02 (0.01-0.03)	0.05 (0.04-0.06)
**Bosnia and Herzegovina**	**Other leukemia**	Benzene	0.19 (0.03-0.35)	0.29 (0.23-0.36)
**Bosnia and Herzegovina**	**Other leukemia**	Formaldehyde	0.03 (0.02-0.04)	0.3 (0.24-0.36)
**Botswana**	**All leukemia**	Benzene	0.58 (0.17-0.99)	0.21 (0.17-0.27)
**Botswana**	**All leukemia**	Formaldehyde	0.16 (0.13-0.2)	0.21 (0.16-0.29)
**Botswana**	**Acute myeloid leukemia**	Benzene	0.88 (0.27-1.53)	0.04 (0.03-0.05)
**Botswana**	**Acute myeloid leukemia**	Formaldehyde	0.24 (0.18-0.32)	0.04 (0.03-0.05)
**Botswana**	**Acute lymphoid leukemia**	Benzene	0.57 (0.17-1.02)	0.02 (0.02-0.02)
**Botswana**	**Acute lymphoid leukemia**	Formaldehyde	0.16 (0.11-0.22)	0.3 (0.24-0.36)
**Botswana**	**Chronic myeloid leukemia**	Benzene	0.77 (0.23-1.36)	0.03 (0.02-0.04)
**Botswana**	**Chronic myeloid leukemia**	Formaldehyde	0.21 (0.15-0.29)	0.2 (0.15-0.26)
**Botswana**	**Chronic lymphoid leukemia**	Benzene	0.38 (0.11-0.65)	0.27 (0.22-0.34)
**Botswana**	**Chronic lymphoid leukemia**	Formaldehyde	0.1 (0.08-0.14)	0.52 (0.39-0.64)
**Botswana**	**Other leukemia**	Benzene	0.6 (0.18-1.02)	0.26 (0.19-0.35)
**Botswana**	**Other leukemia**	Formaldehyde	0.17 (0.13-0.21)	0.37 (0.28-0.46)
**Brazil**	**All leukemia**	Benzene	0.69 (0.2-1.12)	0.25 (0.2-0.31)
**Brazil**	**All leukemia**	Formaldehyde	0.25 (0.21-0.3)	0.42 (0.31-0.56)
**Brazil**	**Acute myeloid leukemia**	Benzene	0.86 (0.25-1.41)	0.03 (0.02-0.04)
**Brazil**	**Acute myeloid leukemia**	Formaldehyde	0.31 (0.25-0.37)	0.2 (0.15-0.26)
**Brazil**	**Acute lymphoid leukemia**	Benzene	0.44 (0.13-0.73)	0.23 (0.15-0.34)
**Brazil**	**Acute lymphoid leukemia**	Formaldehyde	0.17 (0.13-0.21)	0.43 (0.3-0.59)

Countries	Causes	Occupational carcinogens	Percent change in 1990 (95% UI)	Percent change in 2019 (95% UI)
**Brazil**	**Chronic myeloid leukemia**	Benzene	1 (0.29-1.65)	0.21 (0.16-0.29)
**Brazil**	**Chronic myeloid leukemia**	Formaldehyde	0.37 (0.31-0.45)	0.04 (0.03-0.05)
**Brazil**	**Chronic lymphoid leukemia**	Benzene	0.37 (0.11-0.62)	0.03 (0.02-0.04)
**Brazil**	**Chronic lymphoid leukemia**	Formaldehyde	0.14 (0.12-0.17)	0.34 (0.26-0.44)
**Brazil**	**Other leukemia**	Benzene	0.64 (0.18-1.06)	0.43 (0.28-0.59)
**Brazil**	**Other leukemia**	Formaldehyde	0.23 (0.19-0.28)	0.42 (0.3-0.56)
**Brunei Darussalam**	**All leukemia**	Benzene	0.91 (0.17-1.64)	0.3 (0.23-0.39)
**Brunei Darussalam**	**All leukemia**	Formaldehyde	0.08 (0.06-0.1)	0.31 (0.23-0.43)
**Brunei Darussalam**	**Acute myeloid leukemia**	Benzene	0.89 (0.16-1.6)	0.03 (0.02-0.04)
**Brunei Darussalam**	**Acute myeloid leukemia**	Formaldehyde	0.08 (0.06-0.1)	0.23 (0.16-0.31)
**Brunei Darussalam**	**Acute lymphoid leukemia**	Benzene	0.49 (0.08-0.92)	0.21 (0.13-0.32)
**Brunei Darussalam**	**Acute lymphoid leukemia**	Formaldehyde	0.04 (0.03-0.06)	0.23 (0.18-0.28)
**Brunei Darussalam**	**Chronic myeloid leukemia**	Benzene	1.2 (0.23-2.13)	0.42 (0.35-0.51)
**Brunei Darussalam**	**Chronic myeloid leukemia**	Formaldehyde	0.1 (0.08-0.13)	0.33 (0.21-0.45)
**Brunei Darussalam**	**Chronic lymphoid leukemia**	Benzene	0.52 (0.1-0.95)	0.03 (0.02-0.04)
**Brunei Darussalam**	**Chronic lymphoid leukemia**	Formaldehyde	0.04 (0.03-0.06)	0.27 (0.2-0.39)
**Brunei Darussalam**	**Other leukemia**	Benzene	0.75 (0.14-1.39)	0.03 (0.02-0.04)
**Brunei Darussalam**	**Other leukemia**	Formaldehyde	0.06 (0.05-0.09)	0.18 (0.14-0.23)
**Bulgaria**	**All leukemia**	Benzene	0.32 (0.06-0.58)	0.24 (0.14-0.33)
**Bulgaria**	**All leukemia**	Formaldehyde	0.05 (0.05-0.06)	0.26 (0.2-0.33)
**Bulgaria**	**Acute myeloid leukemia**	Benzene	0.35 (0.07-0.63)	0.15 (0.1-0.21)
**Bulgaria**	**Acute myeloid leukemia**	Formaldehyde	0.06 (0.05-0.07)	0.47 (0.34-0.64)
**Bulgaria**	**Acute lymphoid leukemia**	Benzene	0.32 (0.06-0.58)	0.3 (0.24-0.38)
**Bulgaria**	**Acute lymphoid leukemia**	Formaldehyde	0.05 (0.04-0.07)	0.04 (0.03-0.04)
**Bulgaria**	**Chronic myeloid leukemia**	Benzene	0.33 (0.06-0.6)	0.05 (0.04-0.06)
**Bulgaria**	**Chronic myeloid leukemia**	Formaldehyde	0.06 (0.04-0.07)	0.33 (0.27-0.4)
**Bulgaria**	**Chronic lymphoid leukemia**	Benzene	0.16 (0.03-0.29)	0.32 (0.2-0.47)
**Bulgaria**	**Chronic lymphoid leukemia**	Formaldehyde	0.03 (0.02-0.04)	0.27 (0.2-0.35)
**Bulgaria**	**Other leukemia**	Benzene	0.35 (0.06-0.64)	0.19 (0.14-0.25)
**Bulgaria**	**Other leukemia**	Formaldehyde	0.06 (0.05-0.07)	0.05 (0.04-0.06)
**Burkina Faso**	**All leukemia**	Benzene	0.28 (0.08-0.5)	0.33 (0.24-0.43)
**Burkina Faso**	**All leukemia**	Formaldehyde	0.09 (0.07-0.13)	0.04 (0.03-0.05)
**Burkina Faso**	**Acute myeloid leukemia**	Benzene	0.25 (0.06-0.52)	0.27 (0.18-0.4)
**Burkina Faso**	**Acute myeloid leukemia**	Formaldehyde	0.08 (0.04-0.13)	0.38 (0.28-0.5)
**Burkina Faso**	**Acute lymphoid leukemia**	Benzene	0.19 (0.05-0.38)	0.26 (0.2-0.34)
**Burkina Faso**	**Acute lymphoid leukemia**	Formaldehyde	0.06 (0.03-0.11)	0.13 (0.1-0.18)
**Burkina Faso**	**Chronic myeloid leukemia**	Benzene	0.61 (0.19-1.05)	0.34 (0.27-0.42)
**Burkina Faso**	**Chronic myeloid leukemia**	Formaldehyde	0.21 (0.15-0.27)	0.23 (0.16-0.34)
**Burkina Faso**	**Chronic lymphoid leukemia**	Benzene	0.15 (0.04-0.25)	0.35 (0.28-0.42)
**Burkina Faso**	**Chronic lymphoid leukemia**	Formaldehyde	0.05 (0.04-0.07)	0.42 (0.33-0.53)
**Burkina Faso**	**Other leukemia**	Benzene	0.27 (0.08-0.49)	0.4 (0.33-0.48)
**Burkina Faso**	**Other leukemia**	Formaldehyde	0.09 (0.06-0.13)	0.03 (0.03-0.04)
**Burundi**	**All leukemia**	Benzene	0.33 (0.09-0.58)	0.03 (0.02-0.04)
**Burundi**	**All leukemia**	Formaldehyde	0.13 (0.09-0.18)	0.02 (0.02-0.03)

Countries	Causes	Occupational carcinogens	Percent change in 1990 (95% UI)	Percent change in 2019 (95% UI)
**Burundi**	**Acute myeloid leukemia**	Benzene	0.44 (0.12-0.85)	0.6 (0.45-0.79)
**Burundi**	**Acute myeloid leukemia**	Formaldehyde	0.17 (0.09-0.26)	0.07 (0.05-0.08)
**Burundi**	**Acute lymphoid leukemia**	Benzene	0.18 (0.04-0.39)	0.03 (0.02-0.04)
**Burundi**	**Acute lymphoid leukemia**	Formaldehyde	0.07 (0.03-0.12)	0.05 (0.04-0.06)
**Burundi**	**Chronic myeloid leukemia**	Benzene	0.55 (0.15-1.07)	0.04 (0.04-0.05)
**Burundi**	**Chronic myeloid leukemia**	Formaldehyde	0.22 (0.11-0.33)	0.25 (0.18-0.34)
**Burundi**	**Chronic lymphoid leukemia**	Benzene	0.28 (0.08-0.47)	0.27 (0.18-0.34)
**Burundi**	**Chronic lymphoid leukemia**	Formaldehyde	0.11 (0.08-0.14)	0.25 (0.21-0.31)
**Burundi**	**Other leukemia**	Benzene	0.29 (0.08-0.53)	0.26 (0.22-0.32)
**Burundi**	**Other leukemia**	Formaldehyde	0.11 (0.07-0.17)	0.17 (0.12-0.22)
**Cabo Verde**	**All leukemia**	Benzene	0.32 (0.09-0.55)	0.03 (0.02-0.04)
**Cabo Verde**	**All leukemia**	Formaldehyde	0.11 (0.08-0.15)	0.22 (0.16-0.3)
**Cabo Verde**	**Acute myeloid leukemia**	Benzene	0.3 (0.09-0.52)	0.45 (0.33-0.6)
**Cabo Verde**	**Acute myeloid leukemia**	Formaldehyde	0.1 (0.07-0.14)	0.31 (0.2-0.47)
**Cabo Verde**	**Acute lymphoid leukemia**	Benzene	0.25 (0.07-0.46)	0.04 (0.03-0.05)
**Cabo Verde**	**Acute lymphoid leukemia**	Formaldehyde	0.09 (0.05-0.13)	0.51 (0.41-0.65)
**Cabo Verde**	**Chronic myeloid leukemia**	Benzene	0.75 (0.23-1.3)	0.28 (0.2-0.39)
**Cabo Verde**	**Chronic myeloid leukemia**	Formaldehyde	0.26 (0.16-0.38)	0.07 (0.06-0.08)
**Cabo Verde**	**Chronic lymphoid leukemia**	Benzene	0.13 (0.04-0.23)	0.04 (0.03-0.06)
**Cabo Verde**	**Chronic lymphoid leukemia**	Formaldehyde	0.05 (0.03-0.06)	0.04 (0.03-0.05)
**Cabo Verde**	**Other leukemia**	Benzene	0.31 (0.09-0.52)	0.63 (0.34-0.98)
**Cabo Verde**	**Other leukemia**	Formaldehyde	0.11 (0.08-0.15)	0.25 (0.15-0.35)
**Cambodia**	**All leukemia**	Benzene	0.43 (0.12-0.76)	0.23 (0.17-0.31)
**Cambodia**	**All leukemia**	Formaldehyde	0.16 (0.11-0.21)	0.18 (0.08-0.29)
**Cambodia**	**Acute myeloid leukemia**	Benzene	0.55 (0.16-0.98)	0.02 (0.02-0.03)
**Cambodia**	**Acute myeloid leukemia**	Formaldehyde	0.2 (0.14-0.28)	0.32 (0.26-0.41)
**Cambodia**	**Acute lymphoid leukemia**	Benzene	0.27 (0.07-0.52)	0.26 (0.19-0.35)
**Cambodia**	**Acute lymphoid leukemia**	Formaldehyde	0.1 (0.06-0.16)	0.29 (0.23-0.35)
**Cambodia**	**Chronic myeloid leukemia**	Benzene	0.6 (0.18-1.06)	0.04 (0.03-0.05)
**Cambodia**	**Chronic myeloid leukemia**	Formaldehyde	0.22 (0.15-0.32)	0.04 (0.03-0.05)
**Cambodia**	**Chronic lymphoid leukemia**	Benzene	0.41 (0.13-0.71)	0.16 (0.11-0.21)
**Cambodia**	**Chronic lymphoid leukemia**	Formaldehyde	0.15 (0.11-0.2)	0.25 (0.2-0.31)
**Cambodia**	**Other leukemia**	Benzene	0.44 (0.13-0.77)	0.17 (0.13-0.23)
**Cambodia**	**Other leukemia**	Formaldehyde	0.17 (0.11-0.22)	0.34 (0.27-0.44)
**Cameroon**	**All leukemia**	Benzene	0.47 (0.13-0.8)	0.25 (0.15-0.35)
**Cameroon**	**All leukemia**	Formaldehyde	0.16 (0.13-0.2)	0.41 (0.3-0.54)
**Cameroon**	**Acute myeloid leukemia**	Benzene	0.48 (0.13-0.85)	0.24 (0.17-0.33)
**Cameroon**	**Acute myeloid leukemia**	Formaldehyde	0.17 (0.11-0.23)	0.21 (0.16-0.29)
**Cameroon**	**Acute lymphoid leukemia**	Benzene	0.35 (0.09-0.67)	0.3 (0.24-0.37)
**Cameroon**	**Acute lymphoid leukemia**	Formaldehyde	0.12 (0.07-0.19)	0.18 (0.14-0.23)
**Cameroon**	**Chronic myeloid leukemia**	Benzene	0.94 (0.28-1.59)	0.16 (0.12-0.2)
**Cameroon**	**Chronic myeloid leukemia**	Formaldehyde	0.33 (0.24-0.44)	0.25 (0.19-0.32)
**Cameroon**	**Chronic lymphoid leukemia**	Benzene	0.23 (0.07-0.4)	0.27 (0.2-0.36)
**Cameroon**	**Chronic lymphoid leukemia**	Formaldehyde	0.08 (0.06-0.11)	0.25 (0.16-0.35)

Countries	Causes	Occupational carcinogens	Percent change in 1990 (95% UI)	Percent change in 2019 (95% UI)
**Cameroon**	**Other leukemia**	Benzene	0.45 (0.13-0.77)	0.04 (0.04-0.05)
**Cameroon**	**Other leukemia**	Formaldehyde	0.16 (0.12-0.2)	0.52 (0.36-0.74)
**Canada**	**All leukemia**	Benzene	0.36 (0.07-0.64)	0.04 (0.03-0.04)
**Canada**	**All leukemia**	Formaldehyde	0.04 (0.04-0.05)	0.27 (0.16-0.39)
**Canada**	**Acute myeloid leukemia**	Benzene	0.45 (0.09-0.79)	0.04 (0.03-0.05)
**Canada**	**Acute myeloid leukemia**	Formaldehyde	0.05 (0.05-0.06)	0.17 (0.13-0.22)
**Canada**	**Acute lymphoid leukemia**	Benzene	0.57 (0.11-1.03)	0.23 (0.18-0.29)
**Canada**	**Acute lymphoid leukemia**	Formaldehyde	0.07 (0.06-0.09)	0.29 (0.23-0.36)
**Canada**	**Chronic myeloid leukemia**	Benzene	0.46 (0.09-0.82)	0.31 (0.23-0.43)
**Canada**	**Chronic myeloid leukemia**	Formaldehyde	0.06 (0.05-0.07)	0.29 (0.24-0.35)
**Canada**	**Chronic lymphoid leukemia**	Benzene	0.19 (0.04-0.33)	0.34 (0.25-0.44)
**Canada**	**Chronic lymphoid leukemia**	Formaldehyde	0.02 (0.02-0.03)	0.2 (0.13-0.3)
**Canada**	**Other leukemia**	Benzene	0.31 (0.06-0.55)	0.36 (0.26-0.47)
**Canada**	**Other leukemia**	Formaldehyde	0.04 (0.03-0.05)	0.29 (0.2-0.4)
**Central African Republic**	**All leukemia**	Benzene	0.38 (0.11-0.67)	0.54 (0.16-0.94)
**Central African Republic**	**All leukemia**	Formaldehyde	0.13 (0.09-0.17)	0.2 (0.04-0.37)
**Central African Republic**	**Acute myeloid leukemia**	Benzene	0.49 (0.13-0.87)	0.19 (0.06-0.32)
**Central African Republic**	**Acute myeloid leukemia**	Formaldehyde	0.17 (0.1-0.23)	0.78 (0.24-1.29)
**Central African Republic**	**Acute lymphoid leukemia**	Benzene	0.23 (0.06-0.46)	0.2 (0.04-0.37)
**Central African Republic**	**Acute lymphoid leukemia**	Formaldehyde	0.08 (0.04-0.13)	0.55 (0.16-0.94)
**Central African Republic**	**Chronic myeloid leukemia**	Benzene	0.53 (0.15-0.95)	0.66 (0.2-1.11)
**Central African Republic**	**Chronic myeloid leukemia**	Formaldehyde	0.18 (0.12-0.25)	0.36 (0.1-0.6)
**Central African Republic**	**Chronic lymphoid leukemia**	Benzene	0.29 (0.09-0.51)	0.4 (0.12-0.68)
**Central African Republic**	**Chronic lymphoid leukemia**	Formaldehyde	0.1 (0.07-0.13)	0.17 (0.03-0.31)
**Central African Republic**	**Other leukemia**	Benzene	0.38 (0.11-0.69)	0.16 (0.03-0.28)
**Central African Republic**	**Other leukemia**	Formaldehyde	0.13 (0.08-0.18)	0.6 (0.18-1.03)
**Chad**	**All leukemia**	Benzene	0.28 (0.08-0.51)	0.74 (0.22-1.26)
**Chad**	**All leukemia**	Formaldehyde	0.09 (0.06-0.13)	0.56 (0.17-0.94)
**Chad**	**Acute myeloid leukemia**	Benzene	0.26 (0.06-0.53)	0.26 (0.07-0.43)
**Chad**	**Acute myeloid leukemia**	Formaldehyde	0.09 (0.04-0.14)	0.44 (0.13-0.74)
**Chad**	**Acute lymphoid leukemia**	Benzene	0.19 (0.05-0.4)	0.19 (0.04-0.34)
**Chad**	**Acute lymphoid leukemia**	Formaldehyde	0.07 (0.03-0.12)	0.14 (0.03-0.25)
**Chad**	**Chronic myeloid leukemia**	Benzene	0.63 (0.18-1.07)	0.78 (0.23-1.31)
**Chad**	**Chronic myeloid leukemia**	Formaldehyde	0.21 (0.15-0.29)	0.3 (0.09-0.52)
**Chad**	**Chronic lymphoid leukemia**	Benzene	0.14 (0.04-0.24)	0.44 (0.13-0.74)
**Chad**	**Chronic lymphoid leukemia**	Formaldehyde	0.05 (0.03-0.06)	0.31 (0.09-0.52)
**Chad**	**Other leukemia**	Benzene	0.28 (0.08-0.49)	0.63 (0.18-1.06)
**Chad**	**Other leukemia**	Formaldehyde	0.09 (0.06-0.13)	0.15 (0.03-0.27)
**Chile**	**All leukemia**	Benzene	0.66 (0.19-1.09)	0.45 (0.13-0.78)
**Chile**	**All leukemia**	Formaldehyde	0.25 (0.21-0.3)	0.36 (0.1-0.6)
**Chile**	**Acute myeloid leukemia**	Benzene	0.74 (0.21-1.23)	0.69 (0.12-1.25)
**Chile**	**Acute myeloid leukemia**	Formaldehyde	0.28 (0.23-0.33)	0.21 (0.04-0.39)
**Chile**	**Acute lymphoid leukemia**	Benzene	0.57 (0.17-0.97)	0.17 (0.05-0.29)
**Chile**	**Acute lymphoid leukemia**	Formaldehyde	0.22 (0.18-0.27)	0.43 (0.12-0.72)

Countries	Causes	Occupational carcinogens	Percent change in 1990 (95% UI)	Percent change in 2019 (95% UI)
**Chile**	**Chronic myeloid leukemia**	Benzene	0.82 (0.24-1.37)	0.17 (0.05-0.29)
**Chile**	**Chronic myeloid leukemia**	Formaldehyde	0.32 (0.26-0.39)	0.46 (0.14-0.8)
**Chile**	**Chronic lymphoid leukemia**	Benzene	0.31 (0.09-0.53)	0.32 (0.09-0.55)
**Chile**	**Chronic lymphoid leukemia**	Formaldehyde	0.12 (0.1-0.15)	0.17 (0.03-0.31)
**Chile**	**Other leukemia**	Benzene	0.65 (0.19-1.06)	0.33 (0.1-0.58)
**Chile**	**Other leukemia**	Formaldehyde	0.25 (0.2-0.3)	0.2 (0.06-0.34)
**China**	**All leukemia**	Benzene	0.53 (0.16-0.88)	0.3 (0.09-0.5)
**China**	**All leukemia**	Formaldehyde	0.26 (0.21-0.32)	0.6 (0.17-1.01)
**China**	**Acute myeloid leukemia**	Benzene	0.57 (0.16-0.95)	0.49 (0.15-0.8)
**China**	**Acute myeloid leukemia**	Formaldehyde	0.29 (0.24-0.35)	0.36 (0.1-0.63)
**China**	**Acute lymphoid leukemia**	Benzene	0.43 (0.13-0.75)	0.46 (0.13-0.8)
**China**	**Acute lymphoid leukemia**	Formaldehyde	0.21 (0.16-0.26)	0.73 (0.21-1.24)
**China**	**Chronic myeloid leukemia**	Benzene	0.66 (0.19-1.09)	0.3 (0.09-0.51)
**China**	**Chronic myeloid leukemia**	Formaldehyde	0.34 (0.28-0.41)	0.13 (0.02-0.23)
**China**	**Chronic lymphoid leukemia**	Benzene	0.72 (0.21-1.2)	0.4 (0.12-0.68)
**China**	**Chronic lymphoid leukemia**	Formaldehyde	0.34 (0.28-0.41)	0.21 (0.04-0.38)
**China**	**Other leukemia**	Benzene	0.54 (0.16-0.89)	0.19 (0.04-0.34)
**China**	**Other leukemia**	Formaldehyde	0.26 (0.21-0.32)	0.27 (0.08-0.45)
**Colombia**	**All leukemia**	Benzene	0.72 (0.21-1.2)	0.75 (0.22-1.29)
**Colombia**	**All leukemia**	Formaldehyde	0.27 (0.22-0.32)	0.4 (0.12-0.71)
**Colombia**	**Acute myeloid leukemia**	Benzene	0.85 (0.25-1.41)	0.2 (0.04-0.36)
**Colombia**	**Acute myeloid leukemia**	Formaldehyde	0.32 (0.26-0.38)	0.35 (0.1-0.59)
**Colombia**	**Acute lymphoid leukemia**	Benzene	0.56 (0.17-0.94)	0.34 (0.09-0.56)
**Colombia**	**Acute lymphoid leukemia**	Formaldehyde	0.21 (0.16-0.26)	0.56 (0.16-0.95)
**Colombia**	**Chronic myeloid leukemia**	Benzene	0.94 (0.28-1.58)	0.57 (0.17-0.97)
**Colombia**	**Chronic myeloid leukemia**	Formaldehyde	0.36 (0.29-0.43)	0.57 (0.16-0.97)
**Colombia**	**Chronic lymphoid leukemia**	Benzene	0.48 (0.14-0.8)	0.51 (0.15-0.86)
**Colombia**	**Chronic lymphoid leukemia**	Formaldehyde	0.18 (0.15-0.21)	0.5 (0.15-0.89)
**Colombia**	**Other leukemia**	Benzene	0.77 (0.22-1.28)	0.42 (0.12-0.72)
**Colombia**	**Other leukemia**	Formaldehyde	0.29 (0.24-0.35)	0.16 (0.03-0.29)
**Comoros**	**All leukemia**	Benzene	0.26 (0.07-0.47)	0.23 (0.07-0.41)
**Comoros**	**All leukemia**	Formaldehyde	0.1 (0.06-0.14)	0.38 (0.11-0.64)
**Comoros**	**Acute myeloid leukemia**	Benzene	0.35 (0.1-0.74)	0.63 (0.18-1.06)
**Comoros**	**Acute myeloid leukemia**	Formaldehyde	0.14 (0.07-0.23)	0.18 (0.03-0.33)
**Comoros**	**Acute lymphoid leukemia**	Benzene	0.17 (0.04-0.36)	0.12 (0.02-0.23)
**Comoros**	**Acute lymphoid leukemia**	Formaldehyde	0.07 (0.03-0.12)	0.22 (0.06-0.38)
**Comoros**	**Chronic myeloid leukemia**	Benzene	0.46 (0.12-0.9)	0.28 (0.08-0.46)
**Comoros**	**Chronic myeloid leukemia**	Formaldehyde	0.18 (0.09-0.27)	0.39 (0.11-0.65)
**Comoros**	**Chronic lymphoid leukemia**	Benzene	0.17 (0.05-0.3)	0.18 (0.03-0.32)
**Comoros**	**Chronic lymphoid leukemia**	Formaldehyde	0.07 (0.04-0.09)	0.29 (0.09-0.49)
**Comoros**	**Other leukemia**	Benzene	0.23 (0.06-0.43)	0.11 (0.02-0.19)
**Comoros**	**Other leukemia**	Formaldehyde	0.09 (0.05-0.14)	0.31 (0.06-0.57)
**Congo**	**All leukemia**	Benzene	0.43 (0.12-0.74)	0.65 (0.18-1.09)
**Congo**	**All leukemia**	Formaldehyde	0.14 (0.11-0.19)	0.98 (0.3-1.61)

Countries	Causes	Occupational carcinogens	Percent change in 1990 (95% UI)	Percent change in 2019 (95% UI)
**Congo**	**Acute myeloid leukemia**	Benzene	0.53 (0.15-0.94)	0.72 (0.21-1.22)
**Congo**	**Acute myeloid leukemia**	Formaldehyde	0.18 (0.12-0.26)	0.21 (0.06-0.36)
**Congo**	**Acute lymphoid leukemia**	Benzene	0.28 (0.08-0.51)	0.25 (0.07-0.41)
**Congo**	**Acute lymphoid leukemia**	Formaldehyde	0.1 (0.06-0.15)	0.56 (0.17-0.94)
**Congo**	**Chronic myeloid leukemia**	Benzene	0.6 (0.19-1.07)	0.41 (0.12-0.71)
**Congo**	**Chronic myeloid leukemia**	Formaldehyde	0.2 (0.14-0.28)	0.44 (0.13-0.75)
**Congo**	**Chronic lymphoid leukemia**	Benzene	0.27 (0.08-0.46)	0.2 (0.04-0.35)
**Congo**	**Chronic lymphoid leukemia**	Formaldehyde	0.09 (0.06-0.11)	0.27 (0.05-0.5)
**Congo**	**Other leukemia**	Benzene	0.42 (0.12-0.73)	0.21 (0.06-0.34)
**Congo**	**Other leukemia**	Formaldehyde	0.14 (0.1-0.19)	0.52 (0.16-0.89)
**Cook Islands**	**All leukemia**	Benzene	0.83 (0.24-1.4)	0.41 (0.12-0.68)
**Cook Islands**	**All leukemia**	Formaldehyde	0.27 (0.22-0.34)	0.5 (0.15-0.84)
**Cook Islands**	**Acute myeloid leukemia**	Benzene	1.04 (0.31-1.76)	0.18 (0.03-0.33)
**Cook Islands**	**Acute myeloid leukemia**	Formaldehyde	0.34 (0.26-0.46)	0.21 (0.04-0.37)
**Cook Islands**	**Acute lymphoid leukemia**	Benzene	0.89 (0.26-1.53)	0.11 (0.02-0.21)
**Cook Islands**	**Acute lymphoid leukemia**	Formaldehyde	0.29 (0.21-0.39)	0.47 (0.13-0.79)
**Cook Islands**	**Chronic myeloid leukemia**	Benzene	0.91 (0.26-1.54)	0.19 (0.04-0.34)
**Cook Islands**	**Chronic myeloid leukemia**	Formaldehyde	0.31 (0.23-0.41)	0.53 (0.16-0.88)
**Cook Islands**	**Chronic lymphoid leukemia**	Benzene	0.61 (0.18-1.02)	0.6 (0.17-1)
**Cook Islands**	**Chronic lymphoid leukemia**	Formaldehyde	0.21 (0.16-0.26)	0.66 (0.19-1.13)
**Cook Islands**	**Other leukemia**	Benzene	0.66 (0.19-1.14)	0.59 (0.17-1.02)
**Cook Islands**	**Other leukemia**	Formaldehyde	0.21 (0.17-0.27)	0.77 (0.22-1.3)
**Costa Rica**	**All leukemia**	Benzene	0.62 (0.19-1.03)	0.68 (0.2-1.13)
**Costa Rica**	**All leukemia**	Formaldehyde	0.25 (0.21-0.3)	0.55 (0.16-0.95)
**Costa Rica**	**Acute myeloid leukemia**	Benzene	0.71 (0.21-1.19)	0.15 (0.03-0.28)
**Costa Rica**	**Acute myeloid leukemia**	Formaldehyde	0.28 (0.23-0.35)	0.41 (0.12-0.7)
**Costa Rica**	**Acute lymphoid leukemia**	Benzene	0.62 (0.19-1.04)	0.33 (0.09-0.56)
**Costa Rica**	**Acute lymphoid leukemia**	Formaldehyde	0.25 (0.2-0.31)	0.28 (0.08-0.49)
**Costa Rica**	**Chronic myeloid leukemia**	Benzene	0.64 (0.19-1.04)	0.56 (0.16-0.95)
**Costa Rica**	**Chronic myeloid leukemia**	Formaldehyde	0.26 (0.21-0.31)	0.15 (0.03-0.27)
**Costa Rica**	**Chronic lymphoid leukemia**	Benzene	0.27 (0.08-0.46)	0.18 (0.03-0.33)
**Costa Rica**	**Chronic lymphoid leukemia**	Formaldehyde	0.11 (0.09-0.14)	0.45 (0.13-0.76)
**Costa Rica**	**Other leukemia**	Benzene	0.59 (0.17-0.99)	0.57 (0.17-0.93)
**Costa Rica**	**Other leukemia**	Formaldehyde	0.24 (0.19-0.3)	0.37 (0.11-0.64)
**Croatia**	**All leukemia**	Benzene	0.22 (0.04-0.39)	0.58 (0.17-1.01)
**Croatia**	**All leukemia**	Formaldehyde	0.03 (0.02-0.03)	0.29 (0.08-0.48)
**Croatia**	**Acute myeloid leukemia**	Benzene	0.35 (0.06-0.65)	0.18 (0.04-0.34)
**Croatia**	**Acute myeloid leukemia**	Formaldehyde	0.05 (0.04-0.06)	0.97 (0.29-1.67)
**Croatia**	**Acute lymphoid leukemia**	Benzene	0.33 (0.06-0.62)	0.15 (0.04-0.26)
**Croatia**	**Acute lymphoid leukemia**	Formaldehyde	0.05 (0.04-0.06)	0.52 (0.15-0.86)
**Croatia**	**Chronic myeloid leukemia**	Benzene	0.3 (0.06-0.55)	0.48 (0.14-0.81)
**Croatia**	**Chronic myeloid leukemia**	Formaldehyde	0.04 (0.03-0.05)	0.84 (0.25-1.45)
**Croatia**	**Chronic lymphoid leukemia**	Benzene	0.13 (0.02-0.24)	0.17 (0.03-0.32)
**Croatia**	**Chronic lymphoid leukemia**	Formaldehyde	0.02 (0.01-0.02)	0.8 (0.23-1.38)

Countries	Causes	Occupational carcinogens	Percent change in 1990 (95% UI)	Percent change in 2019 (95% UI)
**Croatia**	**Other leukemia**	Benzene	0.21 (0.04-0.39)	0.2 (0.04-0.36)
**Croatia**	**Other leukemia**	Formaldehyde	0.03 (0.02-0.03)	0.43 (0.13-0.73)
**Cuba**	**All leukemia**	Benzene	0.63 (0.19-1.04)	0.28 (0.08-0.49)
**Cuba**	**All leukemia**	Formaldehyde	0.23 (0.19-0.28)	0.39 (0.12-0.67)
**Cuba**	**Acute myeloid leukemia**	Benzene	0.83 (0.24-1.37)	0.28 (0.08-0.48)
**Cuba**	**Acute myeloid leukemia**	Formaldehyde	0.3 (0.24-0.36)	1.31 (0.37-2.23)
**Cuba**	**Acute lymphoid leukemia**	Benzene	0.57 (0.17-0.95)	0.33 (0.09-0.57)
**Cuba**	**Acute lymphoid leukemia**	Formaldehyde	0.21 (0.16-0.26)	0.23 (0.04-0.42)
**Cuba**	**Chronic myeloid leukemia**	Benzene	0.68 (0.2-1.12)	0.22 (0.04-0.39)
**Cuba**	**Chronic myeloid leukemia**	Formaldehyde	0.25 (0.2-0.3)	0.69 (0.2-1.16)
**Cuba**	**Chronic lymphoid leukemia**	Benzene	0.32 (0.09-0.53)	0.25 (0.07-0.42)
**Cuba**	**Chronic lymphoid leukemia**	Formaldehyde	0.12 (0.1-0.14)	0.36 (0.1-0.6)
**Cuba**	**Other leukemia**	Benzene	0.57 (0.17-0.96)	0.59 (0.17-1)
**Cuba**	**Other leukemia**	Formaldehyde	0.21 (0.17-0.25)	0.2 (0.04-0.36)
**Cyprus**	**All leukemia**	Benzene	0.3 (0.06-0.53)	0.87 (0.25-1.45)
**Cyprus**	**All leukemia**	Formaldehyde	0.04 (0.03-0.05)	0.25 (0.05-0.43)
**Cyprus**	**Acute myeloid leukemia**	Benzene	0.36 (0.06-0.64)	0.74 (0.2-1.29)
**Cyprus**	**Acute myeloid leukemia**	Formaldehyde	0.04 (0.03-0.06)	0.26 (0.08-0.45)
**Cyprus**	**Acute lymphoid leukemia**	Benzene	0.43 (0.08-0.79)	0.9 (0.25-1.5)
**Cyprus**	**Acute lymphoid leukemia**	Formaldehyde	0.05 (0.04-0.07)	0.39 (0.11-0.67)
**Cyprus**	**Chronic myeloid leukemia**	Benzene	0.36 (0.07-0.66)	0.43 (0.13-0.73)
**Cyprus**	**Chronic myeloid leukemia**	Formaldehyde	0.04 (0.03-0.06)	0.77 (0.22-1.37)
**Cyprus**	**Chronic lymphoid leukemia**	Benzene	0.17 (0.03-0.31)	0.48 (0.14-0.82)
**Cyprus**	**Chronic lymphoid leukemia**	Formaldehyde	0.02 (0.02-0.03)	0.92 (0.27-1.56)
**Cyprus**	**Other leukemia**	Benzene	0.28 (0.06-0.5)	0.72 (0.22-1.2)
**Cyprus**	**Other leukemia**	Formaldehyde	0.03 (0.03-0.04)	0.2 (0.04-0.35)
**Czechia**	**All leukemia**	Benzene	0.35 (0.06-0.63)	0.15 (0.03-0.28)
**Czechia**	**All leukemia**	Formaldehyde	0.05 (0.04-0.06)	0.12 (0.02-0.22)
**Czechia**	**Acute myeloid leukemia**	Benzene	0.44 (0.08-0.79)	0.9 (0.27-1.48)
**Czechia**	**Acute myeloid leukemia**	Formaldehyde	0.07 (0.06-0.08)	0.4 (0.07-0.71)
**Czechia**	**Acute lymphoid leukemia**	Benzene	0.34 (0.06-0.65)	0.14 (0.03-0.26)
**Czechia**	**Acute lymphoid leukemia**	Formaldehyde	0.05 (0.04-0.07)	0.2 (0.04-0.37)
**Czechia**	**Chronic myeloid leukemia**	Benzene	0.52 (0.09-0.94)	0.23 (0.04-0.41)
**Czechia**	**Chronic myeloid leukemia**	Formaldehyde	0.08 (0.07-0.1)	0.32 (0.09-0.56)
**Czechia**	**Chronic lymphoid leukemia**	Benzene	0.19 (0.04-0.35)	0.61 (0.18-1.05)
**Czechia**	**Chronic lymphoid leukemia**	Formaldehyde	0.03 (0.02-0.04)	0.43 (0.12-0.71)
**Czechia**	**Other leukemia**	Benzene	0.3 (0.06-0.55)	0.45 (0.13-0.74)
**Czechia**	**Other leukemia**	Formaldehyde	0.05 (0.04-0.05)	0.42 (0.12-0.73)
**Côte d'Ivoire**	**All leukemia**	Benzene	0.48 (0.14-0.83)	0.3 (0.06-0.54)
**Côte d'Ivoire**	**All leukemia**	Formaldehyde	0.17 (0.13-0.23)	0.24 (0.07-0.41)
**Côte d'Ivoire**	**Acute myeloid leukemia**	Benzene	0.41 (0.11-0.77)	0.84 (0.25-1.42)
**Côte d'Ivoire**	**Acute myeloid leukemia**	Formaldehyde	0.15 (0.09-0.21)	0.17 (0.05-0.29)
**Côte d'Ivoire**	**Acute lymphoid leukemia**	Benzene	0.29 (0.08-0.55)	0.25 (0.05-0.47)
**Côte d'Ivoire**	**Acute lymphoid leukemia**	Formaldehyde	0.1 (0.06-0.16)	0.72 (0.21-1.2)

Countries	Causes	Occupational carcinogens	Percent change in 1990 (95% UI)	Percent change in 2019 (95% UI)
**Côte d'Ivoire**	**Chronic myeloid leukemia**	Benzene	1.04 (0.32-1.75)	0.23 (0.07-0.4)
**Côte d'Ivoire**	**Chronic myeloid leukemia**	Formaldehyde	0.37 (0.27-0.51)	0.31 (0.06-0.57)
**Côte d'Ivoire**	**Chronic lymphoid leukemia**	Benzene	0.23 (0.07-0.39)	0.2 (0.04-0.37)
**Côte d'Ivoire**	**Chronic lymphoid leukemia**	Formaldehyde	0.08 (0.06-0.11)	0.13 (0.03-0.24)
**Côte d'Ivoire**	**Other leukemia**	Benzene	0.47 (0.14-0.81)	1.27 (0.37-2.23)
**Côte d'Ivoire**	**Other leukemia**	Formaldehyde	0.17 (0.13-0.23)	0.32 (0.09-0.57)
**Democratic People's Republic of Korea**	**All leukemia**	Benzene	0.49 (0.15-0.81)	0.22 (0.06-0.39)
**Democratic People's Republic of Korea**	**All leukemia**	Formaldehyde	0.24 (0.18-0.3)	0.32 (0.09-0.54)
**Democratic People's Republic of Korea**	**Acute myeloid leukemia**	Benzene	0.52 (0.15-0.9)	0.11 (0.02-0.21)
**Democratic People's Republic of Korea**	**Acute myeloid leukemia**	Formaldehyde	0.25 (0.19-0.33)	0.45 (0.13-0.76)
**Democratic People's Republic of Korea**	**Acute lymphoid leukemia**	Benzene	0.39 (0.11-0.68)	0.64 (0.18-1.09)
**Democratic People's Republic of Korea**	**Acute lymphoid leukemia**	Formaldehyde	0.19 (0.13-0.25)	0.6 (0.18-1)
**Democratic People's Republic of Korea**	**Chronic myeloid leukemia**	Benzene	0.63 (0.19-1.1)	0.17 (0.03-0.3)
**Democratic People's Republic of Korea**	**Chronic myeloid leukemia**	Formaldehyde	0.3 (0.23-0.4)	0.17 (0.03-0.31)
**Democratic People's Republic of Korea**	**Chronic lymphoid leukemia**	Benzene	0.67 (0.2-1.14)	0.36 (0.1-0.61)
**Democratic People's Republic of Korea**	**Chronic lymphoid leukemia**	Formaldehyde	0.33 (0.25-0.42)	0.31 (0.09-0.51)
**Democratic People's Republic of Korea**	**Other leukemia**	Benzene	0.49 (0.15-0.82)	0.47 (0.14-0.83)
**Democratic People's Republic of Korea**	**Other leukemia**	Formaldehyde	0.24 (0.18-0.31)	0.26 (0.07-0.45)
**Democratic Republic of the Congo**	**All leukemia**	Benzene	0.4 (0.1-0.83)	0.41 (0.12-0.73)
**Democratic Republic of the Congo**	**All leukemia**	Formaldehyde	0.13 (0.07-0.22)	0.28 (0.08-0.49)
**Democratic Republic of the Congo**	**Acute myeloid leukemia**	Benzene	0.55 (0.14-1.05)	0.73 (0.21-1.27)
**Democratic Republic of the Congo**	**Acute myeloid leukemia**	Formaldehyde	0.18 (0.09-0.28)	0.51 (0.15-0.87)
**Democratic Republic of the Congo**	**Acute lymphoid leukemia**	Benzene	0.27 (0.06-0.64)	0.57 (0.17-0.96)
**Democratic Republic of the Congo**	**Acute lymphoid leukemia**	Formaldehyde	0.09 (0.03-0.18)	0.34 (0.1-0.57)
**Democratic Republic of the Congo**	**Chronic myeloid leukemia**	Benzene	0.56 (0.12-1.12)	0.29 (0.08-0.48)
**Democratic Republic of the Congo**	**Chronic myeloid leukemia**	Formaldehyde	0.18 (0.07-0.29)	0.59 (0.17-0.98)
**Democratic Republic of the Congo**	**Chronic lymphoid leukemia**	Benzene	0.35 (0.1-0.61)	0.74 (0.21-1.25)
**Democratic Republic of the Congo**	**Chronic lymphoid leukemia**	Formaldehyde	0.11 (0.09-0.14)	0.34 (0.1-0.57)
**Democratic Republic of the Congo**	**Other leukemia**	Benzene	0.39 (0.1-0.84)	0.22 (0.04-0.39)
**Democratic Republic of the Congo**	**Other leukemia**	Formaldehyde	0.13 (0.07-0.22)	1.35 (0.4-2.38)
**Denmark**	**All leukemia**	Benzene	0.4 (0.08-0.71)	0.18 (0.03-0.32)
**Denmark**	**All leukemia**	Formaldehyde	0.05 (0.04-0.05)	0.4 (0.12-0.67)

Countries	Causes	Occupational carcinogens	Percent change in 1990 (95% UI)	Percent change in 2019 (95% UI)
**Denmark**	**Acute myeloid leukemia**	Benzene	0.47 (0.09-0.83)	0.18 (0.03-0.32)
**Denmark**	**Acute myeloid leukemia**	Formaldehyde	0.05 (0.04-0.06)	0.5 (0.14-0.85)
**Denmark**	**Acute lymphoid leukemia**	Benzene	0.58 (0.11-1.03)	0.28 (0.08-0.47)
**Denmark**	**Acute lymphoid leukemia**	Formaldehyde	0.07 (0.06-0.09)	0.83 (0.24-1.4)
**Denmark**	**Chronic myeloid leukemia**	Benzene	0.49 (0.1-0.88)	0.99 (0.29-1.68)
**Denmark**	**Chronic myeloid leukemia**	Formaldehyde	0.06 (0.05-0.07)	0.45 (0.13-0.73)
**Denmark**	**Chronic lymphoid leukemia**	Benzene	0.2 (0.04-0.37)	0.33 (0.1-0.58)
**Denmark**	**Chronic lymphoid leukemia**	Formaldehyde	0.03 (0.02-0.03)	0.45 (0.13-0.8)
**Denmark**	**Other leukemia**	Benzene	0.33 (0.06-0.59)	0.47 (0.14-0.78)
**Denmark**	**Other leukemia**	Formaldehyde	0.04 (0.03-0.04)	0.41 (0.12-0.7)
**Djibouti**	**All leukemia**	Benzene	0.23 (0.07-0.42)	0.64 (0.18-1.17)
**Djibouti**	**All leukemia**	Formaldehyde	0.09 (0.06-0.13)	0.4 (0.07-0.73)
**Djibouti**	**Acute myeloid leukemia**	Benzene	0.32 (0.09-0.64)	0.79 (0.23-1.33)
**Djibouti**	**Acute myeloid leukemia**	Formaldehyde	0.13 (0.07-0.21)	0.95 (0.29-1.6)
**Djibouti**	**Acute lymphoid leukemia**	Benzene	0.15 (0.04-0.33)	0.46 (0.09-0.84)
**Djibouti**	**Acute lymphoid leukemia**	Formaldehyde	0.06 (0.03-0.11)	1.13 (0.32-1.91)
**Djibouti**	**Chronic myeloid leukemia**	Benzene	0.42 (0.11-0.82)	1.01 (0.31-1.71)
**Djibouti**	**Chronic myeloid leukemia**	Formaldehyde	0.17 (0.09-0.26)	0.79 (0.23-1.33)
**Djibouti**	**Chronic lymphoid leukemia**	Benzene	0.2 (0.06-0.35)	0.67 (0.19-1.1)
**Djibouti**	**Chronic lymphoid leukemia**	Formaldehyde	0.08 (0.06-0.1)	0.3 (0.06-0.55)
**Djibouti**	**Other leukemia**	Benzene	0.2 (0.05-0.37)	0.32 (0.06-0.57)
**Djibouti**	**Other leukemia**	Formaldehyde	0.08 (0.04-0.12)	0.98 (0.29-1.75)
**Dominica**	**All leukemia**	Benzene	0.46 (0.13-0.76)	1.42 (0.42-2.42)
**Dominica**	**All leukemia**	Formaldehyde	0.16 (0.13-0.19)	1.51 (0.44-2.55)
**Dominica**	**Acute myeloid leukemia**	Benzene	0.65 (0.19-1.1)	0.76 (0.22-1.34)
**Dominica**	**Acute myeloid leukemia**	Formaldehyde	0.22 (0.17-0.28)	0.79 (0.23-1.31)
**Dominica**	**Acute lymphoid leukemia**	Benzene	0.43 (0.12-0.75)	0.48 (0.08-0.87)
**Dominica**	**Acute lymphoid leukemia**	Formaldehyde	0.15 (0.11-0.19)	0.23 (0.05-0.43)
**Dominica**	**Chronic myeloid leukemia**	Benzene	0.53 (0.15-0.9)	1.54 (0.46-2.56)
**Dominica**	**Chronic myeloid leukemia**	Formaldehyde	0.18 (0.14-0.24)	1.29 (0.39-2.2)
**Dominica**	**Chronic lymphoid leukemia**	Benzene	0.25 (0.07-0.41)	0.74 (0.22-1.25)
**Dominica**	**Chronic lymphoid leukemia**	Formaldehyde	0.08 (0.07-0.1)	0.95 (0.28-1.62)
**Dominica**	**Other leukemia**	Benzene	0.42 (0.12-0.71)	1.05 (0.31-1.77)
**Dominica**	**Other leukemia**	Formaldehyde	0.14 (0.12-0.17)	0.23 (0.04-0.43)
**Dominican Republic**	**All leukemia**	Benzene	0.58 (0.17-0.97)	1.1 (0.32-2.03)
**Dominican Republic**	**All leukemia**	Formaldehyde	0.24 (0.19-0.3)	0.8 (0.23-1.31)
**Dominican Republic**	**Acute myeloid leukemia**	Benzene	0.7 (0.21-1.22)	1.27 (0.23-2.26)
**Dominican Republic**	**Acute myeloid leukemia**	Formaldehyde	0.29 (0.22-0.39)	0.32 (0.06-0.58)
**Dominican Republic**	**Acute lymphoid leukemia**	Benzene	0.35 (0.1-0.66)	0.72 (0.21-1.27)
**Dominican Republic**	**Acute lymphoid leukemia**	Formaldehyde	0.15 (0.1-0.23)	0.91 (0.25-1.56)
**Dominican Republic**	**Chronic myeloid leukemia**	Benzene	0.89 (0.26-1.55)	0.64 (0.2-1.15)
**Dominican Republic**	**Chronic myeloid leukemia**	Formaldehyde	0.37 (0.27-0.51)	0.79 (0.24-1.35)
**Dominican Republic**	**Chronic lymphoid leukemia**	Benzene	0.5 (0.14-0.85)	1.44 (0.42-2.44)
**Dominican Republic**	**Chronic lymphoid leukemia**	Formaldehyde	0.2 (0.16-0.25)	0.33 (0.06-0.59)

Countries	Causes	Occupational carcinogens	Percent change in 1990 (95% UI)	Percent change in 2019 (95% UI)
**Dominican Republic**	**Other leukemia**	Benzene	0.61 (0.17-1.01)	0.67 (0.2-1.21)
**Dominican Republic**	**Other leukemia**	Formaldehyde	0.25 (0.19-0.31)	0.83 (0.25-1.41)
**Ecuador**	**All leukemia**	Benzene	0.71 (0.22-1.18)	0.72 (0.21-1.19)
**Ecuador**	**All leukemia**	Formaldehyde	0.26 (0.22-0.31)	0.68 (0.19-1.14)
**Ecuador**	**Acute myeloid leukemia**	Benzene	0.78 (0.24-1.31)	0.99 (0.28-1.65)
**Ecuador**	**Acute myeloid leukemia**	Formaldehyde	0.29 (0.23-0.35)	1.09 (0.31-1.86)
**Ecuador**	**Acute lymphoid leukemia**	Benzene	0.51 (0.15-0.86)	1.07 (0.31-1.88)
**Ecuador**	**Acute lymphoid leukemia**	Formaldehyde	0.19 (0.15-0.24)	1.01 (0.29-1.69)
**Ecuador**	**Chronic myeloid leukemia**	Benzene	0.97 (0.27-1.63)	0.74 (0.22-1.25)
**Ecuador**	**Chronic myeloid leukemia**	Formaldehyde	0.36 (0.29-0.45)	0.23 (0.05-0.42)
**Ecuador**	**Chronic lymphoid leukemia**	Benzene	0.53 (0.16-0.89)	0.69 (0.2-1.14)
**Ecuador**	**Chronic lymphoid leukemia**	Formaldehyde	0.2 (0.16-0.25)	0.44 (0.08-0.82)
**Ecuador**	**Other leukemia**	Benzene	0.81 (0.24-1.33)	0.42 (0.08-0.75)
**Ecuador**	**Other leukemia**	Formaldehyde	0.3 (0.24-0.36)	1.24 (0.36-2.03)
**Egypt**	**All leukemia**	Benzene	0.38 (0.11-0.66)	0.78 (0.23-1.35)
**Egypt**	**All leukemia**	Formaldehyde	0.15 (0.1-0.2)	0.98 (0.29-1.68)
**Egypt**	**Acute myeloid leukemia**	Benzene	0.48 (0.14-0.85)	0.39 (0.07-0.68)
**Egypt**	**Acute myeloid leukemia**	Formaldehyde	0.18 (0.13-0.25)	0.74 (0.21-1.31)
**Egypt**	**Acute lymphoid leukemia**	Benzene	0.34 (0.1-0.61)	0.63 (0.18-1.09)
**Egypt**	**Acute lymphoid leukemia**	Formaldehyde	0.13 (0.07-0.19)	1.12 (0.33-1.9)
**Egypt**	**Chronic myeloid leukemia**	Benzene	0.51 (0.14-0.92)	1.19 (0.35-1.99)
**Egypt**	**Chronic myeloid leukemia**	Formaldehyde	0.2 (0.13-0.28)	0.9 (0.25-1.56)
**Egypt**	**Chronic lymphoid leukemia**	Benzene	0.4 (0.12-0.69)	0.86 (0.24-1.45)
**Egypt**	**Chronic lymphoid leukemia**	Formaldehyde	0.15 (0.11-0.21)	1.39 (0.43-2.47)
**Egypt**	**Other leukemia**	Benzene	0.31 (0.09-0.56)	1.08 (0.32-1.82)
**Egypt**	**Other leukemia**	Formaldehyde	0.12 (0.07-0.17)	0.28 (0.05-0.51)
**El Salvador**	**All leukemia**	Benzene	0.57 (0.17-0.93)	0.63 (0.18-1.15)
**El Salvador**	**All leukemia**	Formaldehyde	0.23 (0.19-0.28)	0.96 (0.25-1.76)
**El Salvador**	**Acute myeloid leukemia**	Benzene	0.62 (0.18-1.05)	0.85 (0.25-1.41)
**El Salvador**	**Acute myeloid leukemia**	Formaldehyde	0.25 (0.19-0.31)	0.37 (0.07-0.68)
**El Salvador**	**Acute lymphoid leukemia**	Benzene	0.36 (0.11-0.65)	0.2 (0.04-0.37)
**El Salvador**	**Acute lymphoid leukemia**	Formaldehyde	0.15 (0.1-0.2)	0.68 (0.2-1.18)
**El Salvador**	**Chronic myeloid leukemia**	Benzene	0.67 (0.2-1.14)	1.13 (0.32-1.92)
**El Salvador**	**Chronic myeloid leukemia**	Formaldehyde	0.27 (0.21-0.35)	0.86 (0.26-1.45)
**El Salvador**	**Chronic lymphoid leukemia**	Benzene	0.48 (0.14-0.8)	0.3 (0.05-0.54)
**El Salvador**	**Chronic lymphoid leukemia**	Formaldehyde	0.19 (0.16-0.24)	1.47 (0.43-2.46)
**El Salvador**	**Other leukemia**	Benzene	0.59 (0.17-0.97)	0.15 (0.03-0.28)
**El Salvador**	**Other leukemia**	Formaldehyde	0.24 (0.19-0.29)	0.39 (0.07-0.7)
**Equatorial Guinea**	**All leukemia**	Benzene	0.27 (0.08-0.49)	0.98 (0.28-1.64)
**Equatorial Guinea**	**All leukemia**	Formaldehyde	0.07 (0.05-0.1)	1.11 (0.33-1.85)
**Equatorial Guinea**	**Acute myeloid leukemia**	Benzene	0.35 (0.1-0.65)	0.94 (0.27-1.6)
**Equatorial Guinea**	**Acute myeloid leukemia**	Formaldehyde	0.1 (0.06-0.14)	0.79 (0.23-1.35)
**Equatorial Guinea**	**Acute lymphoid leukemia**	Benzene	0.17 (0.04-0.34)	1.18 (0.36-2.02)
**Equatorial Guinea**	**Acute lymphoid leukemia**	Formaldehyde	0.05 (0.02-0.08)	1.08 (0.32-1.79)

Countries	Causes	Occupational carcinogens	Percent change in 1990 (95% UI)	Percent change in 2019 (95% UI)
**Equatorial Guinea**	**Chronic myeloid leukemia**	Benzene	0.38 (0.11-0.69)	0.67 (0.19-1.18)
**Equatorial Guinea**	**Chronic myeloid leukemia**	Formaldehyde	0.1 (0.06-0.15)	0.85 (0.23-1.45)
**Equatorial Guinea**	**Chronic lymphoid leukemia**	Benzene	0.21 (0.06-0.36)	0.36 (0.07-0.64)
**Equatorial Guinea**	**Chronic lymphoid leukemia**	Formaldehyde	0.06 (0.04-0.08)	0.49 (0.09-0.91)
**Equatorial Guinea**	**Other leukemia**	Benzene	0.26 (0.07-0.49)	0.78 (0.23-1.3)
**Equatorial Guinea**	**Other leukemia**	Formaldehyde	0.07 (0.04-0.11)	0.9 (0.26-1.52)
**Eritrea**	**All leukemia**	Benzene	0.31 (0.09-0.55)	0.61 (0.18-1.03)
**Eritrea**	**All leukemia**	Formaldehyde	0.12 (0.09-0.16)	0.85 (0.24-1.46)
**Eritrea**	**Acute myeloid leukemia**	Benzene	0.43 (0.12-0.81)	0.41 (0.08-0.74)
**Eritrea**	**Acute myeloid leukemia**	Formaldehyde	0.16 (0.09-0.25)	0.4 (0.07-0.74)
**Eritrea**	**Acute lymphoid leukemia**	Benzene	0.18 (0.05-0.38)	0.17 (0.03-0.32)
**Eritrea**	**Acute lymphoid leukemia**	Formaldehyde	0.07 (0.04-0.11)	1.04 (0.3-1.72)
**Eritrea**	**Chronic myeloid leukemia**	Benzene	0.52 (0.15-0.98)	0.28 (0.05-0.49)
**Eritrea**	**Chronic myeloid leukemia**	Formaldehyde	0.2 (0.11-0.29)	0.73 (0.21-1.21)
**Eritrea**	**Chronic lymphoid leukemia**	Benzene	0.27 (0.08-0.47)	0.99 (0.29-1.65)
**Eritrea**	**Chronic lymphoid leukemia**	Formaldehyde	0.1 (0.08-0.14)	1.77 (0.53-3.03)
**Eritrea**	**Other leukemia**	Benzene	0.28 (0.08-0.52)	1.04 (0.3-1.79)
**Eritrea**	**Other leukemia**	Formaldehyde	0.11 (0.07-0.16)	1.68 (0.5-2.78)
**Estonia**	**All leukemia**	Benzene	0.42 (0.08-0.74)	0.84 (0.25-1.41)
**Estonia**	**All leukemia**	Formaldehyde	0.06 (0.05-0.07)	0.96 (0.29-1.69)
**Estonia**	**Acute myeloid leukemia**	Benzene	0.57 (0.11-1.01)	0.3 (0.05-0.55)
**Estonia**	**Acute myeloid leukemia**	Formaldehyde	0.08 (0.06-0.09)	0.74 (0.21-1.25)
**Estonia**	**Acute lymphoid leukemia**	Benzene	0.48 (0.09-0.87)	0.76 (0.22-1.41)
**Estonia**	**Acute lymphoid leukemia**	Formaldehyde	0.07 (0.05-0.08)	1.26 (0.38-2.14)
**Estonia**	**Chronic myeloid leukemia**	Benzene	0.44 (0.08-0.78)	0.9 (0.26-1.54)
**Estonia**	**Chronic myeloid leukemia**	Formaldehyde	0.06 (0.05-0.07)	0.38 (0.08-0.69)
**Estonia**	**Chronic lymphoid leukemia**	Benzene	0.31 (0.06-0.54)	0.37 (0.07-0.67)
**Estonia**	**Chronic lymphoid leukemia**	Formaldehyde	0.04 (0.03-0.05)	1.03 (0.3-1.75)
**Estonia**	**Other leukemia**	Benzene	0.34 (0.07-0.61)	1.21 (0.34-2.15)
**Estonia**	**Other leukemia**	Formaldehyde	0.05 (0.04-0.05)	1.11 (0.31-1.88)
**Eswatini**	**All leukemia**	Benzene	0.45 (0.13-0.77)	0.97 (0.27-1.62)
**Eswatini**	**All leukemia**	Formaldehyde	0.16 (0.12-0.21)	0.9 (0.26-1.53)
**Eswatini**	**Acute myeloid leukemia**	Benzene	0.67 (0.19-1.2)	0.32 (0.06-0.59)
**Eswatini**	**Acute myeloid leukemia**	Formaldehyde	0.25 (0.17-0.33)	1.23 (0.37-2.13)
**Eswatini**	**Acute lymphoid leukemia**	Benzene	0.34 (0.09-0.67)	0.68 (0.2-1.2)
**Eswatini**	**Acute lymphoid leukemia**	Formaldehyde	0.12 (0.07-0.2)	0.67 (0.19-1.1)
**Eswatini**	**Chronic myeloid leukemia**	Benzene	0.55 (0.15-1.01)	1.09 (0.33-1.8)
**Eswatini**	**Chronic myeloid leukemia**	Formaldehyde	0.2 (0.12-0.29)	1.15 (0.34-2.06)
**Eswatini**	**Chronic lymphoid leukemia**	Benzene	0.32 (0.09-0.56)	0.34 (0.07-0.64)
**Eswatini**	**Chronic lymphoid leukemia**	Formaldehyde	0.12 (0.09-0.16)	0.91 (0.27-1.58)
**Eswatini**	**Other leukemia**	Benzene	0.46 (0.14-0.8)	0.35 (0.07-0.64)
**Eswatini**	**Other leukemia**	Formaldehyde	0.17 (0.13-0.23)	0.58 (0.17-0.99)
**Ethiopia**	**All leukemia**	Benzene	0.26 (0.07-0.49)	0.68 (0.21-1.26)
**Ethiopia**	**All leukemia**	Formaldehyde	0.1 (0.06-0.14)	0.67 (0.2-1.12)

Countries	Causes	Occupational carcinogens	Percent change in 1990 (95% UI)	Percent change in 2019 (95% UI)
**Ethiopia**	**Acute myeloid leukemia**	Benzene	0.33 (0.09-0.68)	0.67 (0.2-1.21)
**Ethiopia**	**Acute myeloid leukemia**	Formaldehyde	0.13 (0.07-0.21)	1.78 (0.53-3.03)
**Ethiopia**	**Acute lymphoid leukemia**	Benzene	0.13 (0.03-0.28)	0.86 (0.25-1.46)
**Ethiopia**	**Acute lymphoid leukemia**	Formaldehyde	0.05 (0.02-0.08)	0.4 (0.08-0.71)
**Ethiopia**	**Chronic myeloid leukemia**	Benzene	0.42 (0.11-0.85)	0.51 (0.1-0.92)
**Ethiopia**	**Chronic myeloid leukemia**	Formaldehyde	0.16 (0.08-0.27)	1.05 (0.3-1.76)
**Ethiopia**	**Chronic lymphoid leukemia**	Benzene	0.24 (0.07-0.41)	0.93 (0.26-1.62)
**Ethiopia**	**Chronic lymphoid leukemia**	Formaldehyde	0.09 (0.07-0.12)	1.11 (0.31-1.89)
**Ethiopia**	**Other leukemia**	Benzene	0.18 (0.04-0.42)	0.8 (0.22-1.37)
**Ethiopia**	**Other leukemia**	Formaldehyde	0.07 (0.03-0.14)	0.39 (0.08-0.72)
**Fiji**	**All leukemia**	Benzene	0.81 (0.25-1.37)	0.92 (0.28-1.55)
**Fiji**	**All leukemia**	Formaldehyde	0.28 (0.22-0.35)	0.52 (0.1-0.92)
**Fiji**	**Acute myeloid leukemia**	Benzene	0.93 (0.28-1.57)	1.1 (0.3-1.99)
**Fiji**	**Acute myeloid leukemia**	Formaldehyde	0.32 (0.25-0.41)	0.92 (0.27-1.58)
**Fiji**	**Acute lymphoid leukemia**	Benzene	0.68 (0.2-1.24)	1.08 (0.31-1.85)
**Fiji**	**Acute lymphoid leukemia**	Formaldehyde	0.23 (0.15-0.33)	0.56 (0.16-0.95)
**Fiji**	**Chronic myeloid leukemia**	Benzene	0.91 (0.27-1.55)	1.28 (0.38-2.14)
**Fiji**	**Chronic myeloid leukemia**	Formaldehyde	0.33 (0.25-0.43)	0.87 (0.26-1.55)
**Fiji**	**Chronic lymphoid leukemia**	Benzene	0.61 (0.18-1.02)	1.14 (0.33-1.9)
**Fiji**	**Chronic lymphoid leukemia**	Formaldehyde	0.21 (0.16-0.27)	1.3 (0.37-2.2)
**Fiji**	**Other leukemia**	Benzene	0.65 (0.2-1.11)	1.18 (0.35-1.95)
**Fiji**	**Other leukemia**	Formaldehyde	0.22 (0.17-0.29)	0.29 (0.06-0.53)
**Finland**	**All leukemia**	Benzene	0.4 (0.08-0.71)	0.27 (0.05-0.5)
**Finland**	**All leukemia**	Formaldehyde	0.05 (0.04-0.05)	0.25 (0.05-0.46)
**Finland**	**Acute myeloid leukemia**	Benzene	0.46 (0.09-0.82)	2.17 (0.65-3.59)
**Finland**	**Acute myeloid leukemia**	Formaldehyde	0.05 (0.04-0.06)	0.54 (0.11-0.99)
**Finland**	**Acute lymphoid leukemia**	Benzene	0.55 (0.1-0.98)	0.32 (0.06-0.57)
**Finland**	**Acute lymphoid leukemia**	Formaldehyde	0.07 (0.05-0.08)	0.34 (0.06-0.61)
**Finland**	**Chronic myeloid leukemia**	Benzene	0.51 (0.1-0.93)	0.43 (0.08-0.77)
**Finland**	**Chronic myeloid leukemia**	Formaldehyde	0.06 (0.05-0.07)	0.83 (0.24-1.42)
**Finland**	**Chronic lymphoid leukemia**	Benzene	0.19 (0.04-0.34)	0.94 (0.27-1.66)
**Finland**	**Chronic lymphoid leukemia**	Formaldehyde	0.03 (0.02-0.03)	0.87 (0.25-1.45)
**Finland**	**Other leukemia**	Benzene	0.32 (0.06-0.57)	0.89 (0.25-1.48)
**Finland**	**Other leukemia**	Formaldehyde	0.04 (0.03-0.04)	0.57 (0.17-0.98)
**France**	**All leukemia**	Benzene	0.31 (0.06-0.56)	0.28 (0.05-0.53)
**France**	**All leukemia**	Formaldehyde	0.04 (0.03-0.04)	0.91 (0.26-1.54)
**France**	**Acute myeloid leukemia**	Benzene	0.4 (0.07-0.72)	2 (0.61-3.29)
**France**	**Acute myeloid leukemia**	Formaldehyde	0.05 (0.04-0.05)	0.8 (0.23-1.37)
**France**	**Acute lymphoid leukemia**	Benzene	0.51 (0.09-0.91)	0.34 (0.06-0.62)
**France**	**Acute lymphoid leukemia**	Formaldehyde	0.06 (0.05-0.07)	1.95 (0.59-3.25)
**France**	**Chronic myeloid leukemia**	Benzene	0.39 (0.07-0.68)	1.02 (0.29-1.74)
**France**	**Chronic myeloid leukemia**	Formaldehyde	0.04 (0.04-0.05)	0.76 (0.15-1.34)
**France**	**Chronic lymphoid leukemia**	Benzene	0.16 (0.03-0.28)	0.34 (0.06-0.61)
**France**	**Chronic lymphoid leukemia**	Formaldehyde	0.02 (0.01-0.02)	0.28 (0.06-0.51)

Countries	Causes	Occupational carcinogens	Percent change in 1990 (95% UI)	Percent change in 2019 (95% UI)
**France**	**Other leukemia**	Benzene	0.28 (0.05-0.5)	1.78 (0.49-3.3)
**France**	**Other leukemia**	Formaldehyde	0.03 (0.03-0.04)	0.71 (0.19-1.33)
**Gabon**	**All leukemia**	Benzene	0.3 (0.08-0.52)	0.68 (0.19-1.2)
**Gabon**	**All leukemia**	Formaldehyde	0.09 (0.07-0.12)	0.52 (0.13-1.07)
**Gabon**	**Acute myeloid leukemia**	Benzene	0.38 (0.11-0.7)	0.22 (0.04-0.41)
**Gabon**	**Acute myeloid leukemia**	Formaldehyde	0.11 (0.07-0.17)	0.83 (0.25-1.41)
**Gabon**	**Acute lymphoid leukemia**	Benzene	0.21 (0.05-0.41)	0.8 (0.23-1.37)
**Gabon**	**Acute lymphoid leukemia**	Formaldehyde	0.06 (0.03-0.1)	1.15 (0.35-1.91)
**Gabon**	**Chronic myeloid leukemia**	Benzene	0.4 (0.12-0.71)	0.43 (0.08-0.77)
**Gabon**	**Chronic myeloid leukemia**	Formaldehyde	0.12 (0.09-0.16)	0.36 (0.07-0.65)
**Gabon**	**Chronic lymphoid leukemia**	Benzene	0.14 (0.04-0.24)	0.55 (0.16-0.97)
**Gabon**	**Chronic lymphoid leukemia**	Formaldehyde	0.04 (0.03-0.05)	0.55 (0.16-0.94)
**Gabon**	**Other leukemia**	Benzene	0.29 (0.08-0.5)	0.69 (0.19-1.2)
**Gabon**	**Other leukemia**	Formaldehyde	0.09 (0.06-0.12)	0.86 (0.24-1.45)
**Gambia**	**All leukemia**	Benzene	0.41 (0.12-0.7)	0.66 (0.18-1.17)
**Gambia**	**All leukemia**	Formaldehyde	0.15 (0.11-0.19)	1.27 (0.4-2.15)
**Gambia**	**Acute myeloid leukemia**	Benzene	0.36 (0.1-0.68)	0.93 (0.26-1.59)
**Gambia**	**Acute myeloid leukemia**	Formaldehyde	0.13 (0.07-0.21)	0.6 (0.17-1)
**Gambia**	**Acute lymphoid leukemia**	Benzene	0.28 (0.08-0.51)	0.94 (0.27-1.58)
**Gambia**	**Acute lymphoid leukemia**	Formaldehyde	0.1 (0.06-0.15)	0.59 (0.17-1.02)
**Gambia**	**Chronic myeloid leukemia**	Benzene	0.87 (0.26-1.5)	0.41 (0.12-0.66)
**Gambia**	**Chronic myeloid leukemia**	Formaldehyde	0.31 (0.22-0.44)	0.92 (0.27-1.59)
**Gambia**	**Chronic lymphoid leukemia**	Benzene	0.21 (0.06-0.36)	0.98 (0.29-1.68)
**Gambia**	**Chronic lymphoid leukemia**	Formaldehyde	0.08 (0.06-0.1)	0.76 (0.22-1.34)
**Gambia**	**Other leukemia**	Benzene	0.41 (0.12-0.69)	0.43 (0.08-0.78)
**Gambia**	**Other leukemia**	Formaldehyde	0.15 (0.11-0.19)	2.04 (0.62-3.55)
**Georgia**	**All leukemia**	Benzene	0.79 (0.22-1.3)	0.37 (0.07-0.66)
**Georgia**	**All leukemia**	Formaldehyde	0.22 (0.18-0.27)	0.82 (0.22-1.49)
**Georgia**	**Acute myeloid leukemia**	Benzene	0.89 (0.26-1.5)	0.36 (0.07-0.65)
**Georgia**	**Acute myeloid leukemia**	Formaldehyde	0.25 (0.2-0.31)	0.77 (0.23-1.31)
**Georgia**	**Acute lymphoid leukemia**	Benzene	0.68 (0.19-1.14)	0.75 (0.22-1.27)
**Georgia**	**Acute lymphoid leukemia**	Formaldehyde	0.19 (0.15-0.25)	1.26 (0.37-2.1)
**Georgia**	**Chronic myeloid leukemia**	Benzene	0.96 (0.28-1.6)	1.21 (0.36-2.08)
**Georgia**	**Chronic myeloid leukemia**	Formaldehyde	0.27 (0.22-0.33)	0.89 (0.26-1.46)
**Georgia**	**Chronic lymphoid leukemia**	Benzene	0.59 (0.17-0.98)	0.85 (0.24-1.42)
**Georgia**	**Chronic lymphoid leukemia**	Formaldehyde	0.16 (0.13-0.19)	0.68 (0.19-1.25)
**Georgia**	**Other leukemia**	Benzene	0.78 (0.22-1.3)	1.15 (0.32-2.01)
**Georgia**	**Other leukemia**	Formaldehyde	0.22 (0.18-0.27)	0.85 (0.24-1.45)
**Germany**	**All leukemia**	Benzene	0.37 (0.07-0.65)	0.4 (0.11-0.73)
**Germany**	**All leukemia**	Formaldehyde	0.05 (0.04-0.06)	0.42 (0.07-0.78)
**Germany**	**Acute myeloid leukemia**	Benzene	0.47 (0.09-0.83)	0.54 (0.16-0.95)
**Germany**	**Acute myeloid leukemia**	Formaldehyde	0.06 (0.05-0.07)	0.98 (0.28-1.65)
**Germany**	**Acute lymphoid leukemia**	Benzene	0.62 (0.12-1.1)	0.78 (0.15-1.41)
**Germany**	**Acute lymphoid leukemia**	Formaldehyde	0.09 (0.07-0.11)	0.42 (0.11-0.8)

Countries	Causes	Occupational carcinogens	Percent change in 1990 (95% UI)	Percent change in 2019 (95% UI)
**Germany**	**Chronic myeloid leukemia**	Benzene	0.43 (0.08-0.78)	0.97 (0.3-1.6)
**Germany**	**Chronic myeloid leukemia**	Formaldehyde	0.06 (0.05-0.07)	1.03 (0.3-1.68)
**Germany**	**Chronic lymphoid leukemia**	Benzene	0.2 (0.04-0.37)	0.86 (0.26-1.42)
**Germany**	**Chronic lymphoid leukemia**	Formaldehyde	0.03 (0.02-0.03)	0.58 (0.11-1.03)
**Germany**	**Other leukemia**	Benzene	0.26 (0.05-0.48)	0.61 (0.12-1.1)
**Germany**	**Other leukemia**	Formaldehyde	0.04 (0.03-0.04)	0.92 (0.27-1.62)
**Ghana**	**All leukemia**	Benzene	0.81 (0.24-1.35)	1.2 (0.36-2)
**Ghana**	**All leukemia**	Formaldehyde	0.63 (0.49-0.8)	1.34 (0.4-2.3)
**Ghana**	**Acute myeloid leukemia**	Benzene	0.69 (0.2-1.23)	0.5 (0.13-0.93)
**Ghana**	**Acute myeloid leukemia**	Formaldehyde	0.54 (0.37-0.73)	0.91 (0.27-1.5)
**Ghana**	**Acute lymphoid leukemia**	Benzene	0.62 (0.17-1.17)	0.52 (0.1-0.93)
**Ghana**	**Acute lymphoid leukemia**	Formaldehyde	0.49 (0.29-0.78)	0.48 (0.09-0.88)
**Ghana**	**Chronic myeloid leukemia**	Benzene	1.78 (0.53-3.02)	0.97 (0.29-1.63)
**Ghana**	**Chronic myeloid leukemia**	Formaldehyde	1.37 (0.99-1.83)	0.42 (0.12-0.81)
**Ghana**	**Chronic lymphoid leukemia**	Benzene	0.33 (0.1-0.55)	0.99 (0.29-1.63)
**Ghana**	**Chronic lymphoid leukemia**	Formaldehyde	0.26 (0.19-0.35)	0.68 (0.2-1.23)
**Ghana**	**Other leukemia**	Benzene	0.86 (0.25-1.45)	0.77 (0.22-1.3)
**Ghana**	**Other leukemia**	Formaldehyde	0.66 (0.5-0.88)	0.34 (0.06-0.62)
**Greece**	**All leukemia**	Benzene	0.22 (0.04-0.4)	0.95 (0.25-1.76)
**Greece**	**All leukemia**	Formaldehyde	0.03 (0.02-0.03)	0.87 (0.25-1.43)
**Greece**	**Acute myeloid leukemia**	Benzene	0.29 (0.06-0.51)	1.05 (0.18-1.91)
**Greece**	**Acute myeloid leukemia**	Formaldehyde	0.04 (0.03-0.04)	0.49 (0.09-0.87)
**Greece**	**Acute lymphoid leukemia**	Benzene	0.37 (0.07-0.65)	0.22 (0.06-0.42)
**Greece**	**Acute lymphoid leukemia**	Formaldehyde	0.05 (0.04-0.06)	0.31 (0.09-0.58)
**Greece**	**Chronic myeloid leukemia**	Benzene	0.22 (0.04-0.4)	0.53 (0.16-0.99)
**Greece**	**Chronic myeloid leukemia**	Formaldehyde	0.03 (0.02-0.03)	0.63 (0.18-1.09)
**Greece**	**Chronic lymphoid leukemia**	Benzene	0.14 (0.03-0.26)	0.49 (0.14-0.87)
**Greece**	**Chronic lymphoid leukemia**	Formaldehyde	0.02 (0.02-0.02)	0.56 (0.1-1.01)
**Greece**	**Other leukemia**	Benzene	0.19 (0.04-0.34)	0.27 (0.07-0.51)
**Greece**	**Other leukemia**	Formaldehyde	0.02 (0.02-0.03)	0.23 (0.07-0.43)
**Greenland**	**All leukemia**	Benzene	0.47 (0.09-0.87)	0.97 (0.29-1.61)
**Greenland**	**All leukemia**	Formaldehyde	0.05 (0.04-0.07)	0.69 (0.2-1.14)
**Greenland**	**Acute myeloid leukemia**	Benzene	0.54 (0.1-1)	1.12 (0.32-1.86)
**Greenland**	**Acute myeloid leukemia**	Formaldehyde	0.06 (0.05-0.08)	0.52 (0.14-0.95)
**Greenland**	**Acute lymphoid leukemia**	Benzene	0.49 (0.09-0.91)	0.52 (0.15-0.9)
**Greenland**	**Acute lymphoid leukemia**	Formaldehyde	0.06 (0.04-0.08)	1.32 (0.4-2.22)
**Greenland**	**Chronic myeloid leukemia**	Benzene	0.63 (0.11-1.16)	1.12 (0.33-1.85)
**Greenland**	**Chronic myeloid leukemia**	Formaldehyde	0.07 (0.05-0.1)	0.39 (0.07-0.69)
**Greenland**	**Chronic lymphoid leukemia**	Benzene	0.38 (0.07-0.69)	0.87 (0.25-1.46)
**Greenland**	**Chronic lymphoid leukemia**	Formaldehyde	0.04 (0.03-0.05)	0.63 (0.11-1.18)
**Greenland**	**Other leukemia**	Benzene	0.41 (0.07-0.78)	0.42 (0.08-0.77)
**Greenland**	**Other leukemia**	Formaldehyde	0.05 (0.04-0.07)	0.46 (0.14-0.79)
**Grenada**	**All leukemia**	Benzene	0.55 (0.16-0.92)	0.71 (0.2-1.23)
**Grenada**	**All leukemia**	Formaldehyde	0.18 (0.15-0.22)	0.45 (0.13-0.82)

Countries	Causes	Occupational carcinogens	Percent change in 1990 (95% UI)	Percent change in 2019 (95% UI)
**Grenada**	**Acute myeloid leukemia**	Benzene	0.71 (0.21-1.19)	0.65 (0.12-1.16)
**Grenada**	**Acute myeloid leukemia**	Formaldehyde	0.23 (0.18-0.29)	0.28 (0.08-0.55)
**Grenada**	**Acute lymphoid leukemia**	Benzene	0.39 (0.11-0.67)	0.67 (0.19-1.14)
**Grenada**	**Acute lymphoid leukemia**	Formaldehyde	0.13 (0.1-0.17)	0.77 (0.22-1.34)
**Grenada**	**Chronic myeloid leukemia**	Benzene	0.69 (0.2-1.15)	1.03 (0.31-1.73)
**Grenada**	**Chronic myeloid leukemia**	Formaldehyde	0.23 (0.19-0.28)	0.78 (0.23-1.36)
**Grenada**	**Chronic lymphoid leukemia**	Benzene	0.3 (0.08-0.51)	0.98 (0.28-1.63)
**Grenada**	**Chronic lymphoid leukemia**	Formaldehyde	0.09 (0.08-0.12)	0.69 (0.2-1.31)
**Grenada**	**Other leukemia**	Benzene	0.57 (0.17-0.95)	0.4 (0.11-0.78)
**Grenada**	**Other leukemia**	Formaldehyde	0.19 (0.15-0.23)	0.51 (0.1-0.94)
**Guam**	**All leukemia**	Benzene	1.01 (0.3-1.69)	0.51 (0.14-0.95)
**Guam**	**All leukemia**	Formaldehyde	0.3 (0.24-0.37)	0.36 (0.09-0.71)
**Guam**	**Acute myeloid leukemia**	Benzene	1.23 (0.37-2.06)	0.79 (0.24-1.38)
**Guam**	**Acute myeloid leukemia**	Formaldehyde	0.36 (0.28-0.46)	0.58 (0.11-1.04)
**Guam**	**Acute lymphoid leukemia**	Benzene	0.96 (0.28-1.68)	0.5 (0.1-0.91)
**Guam**	**Acute lymphoid leukemia**	Formaldehyde	0.28 (0.2-0.39)	0.45 (0.13-0.8)
**Guam**	**Chronic myeloid leukemia**	Benzene	0.99 (0.29-1.71)	0.55 (0.16-0.99)
**Guam**	**Chronic myeloid leukemia**	Formaldehyde	0.29 (0.22-0.39)	0.81 (0.24-1.36)
**Guam**	**Chronic lymphoid leukemia**	Benzene	0.68 (0.2-1.19)	0.62 (0.12-1.11)
**Guam**	**Chronic lymphoid leukemia**	Formaldehyde	0.2 (0.15-0.27)	0.87 (0.26-1.5)
**Guam**	**Other leukemia**	Benzene	0.74 (0.21-1.26)	0.34 (0.07-0.6)
**Guam**	**Other leukemia**	Formaldehyde	0.22 (0.17-0.28)	0.5 (0.09-0.91)
**Guatemala**	**All leukemia**	Benzene	0.64 (0.18-1.08)	0.95 (0.29-1.62)
**Guatemala**	**All leukemia**	Formaldehyde	0.28 (0.22-0.34)	1.13 (0.33-1.87)
**Guatemala**	**Acute myeloid leukemia**	Benzene	0.71 (0.2-1.23)	0.98 (0.3-1.63)
**Guatemala**	**Acute myeloid leukemia**	Formaldehyde	0.31 (0.24-0.4)	0.3 (0.1-0.54)
**Guatemala**	**Acute lymphoid leukemia**	Benzene	0.5 (0.15-0.88)	0.46 (0.13-0.83)
**Guatemala**	**Acute lymphoid leukemia**	Formaldehyde	0.22 (0.15-0.29)	0.86 (0.25-1.41)
**Guatemala**	**Chronic myeloid leukemia**	Benzene	0.71 (0.2-1.2)	0.25 (0.07-0.46)
**Guatemala**	**Chronic myeloid leukemia**	Formaldehyde	0.31 (0.25-0.38)	0.72 (0.21-1.26)
**Guatemala**	**Chronic lymphoid leukemia**	Benzene	0.65 (0.19-1.08)	0.5 (0.09-0.9)
**Guatemala**	**Chronic lymphoid leukemia**	Formaldehyde	0.28 (0.23-0.35)	0.79 (0.16-1.43)
**Guatemala**	**Other leukemia**	Benzene	0.65 (0.18-1.09)	0.57 (0.16-0.98)
**Guatemala**	**Other leukemia**	Formaldehyde	0.28 (0.23-0.35)	0.73 (0.22-1.24)
**Guinea**	**All leukemia**	Benzene	0.26 (0.07-0.46)	0.65 (0.19-1.08)
**Guinea**	**All leukemia**	Formaldehyde	0.08 (0.06-0.1)	0.7 (0.2-1.2)
**Guinea**	**Acute myeloid leukemia**	Benzene	0.23 (0.06-0.48)	0.55 (0.11-0.99)
**Guinea**	**Acute myeloid leukemia**	Formaldehyde	0.07 (0.03-0.12)	0.76 (0.14-1.35)
**Guinea**	**Acute lymphoid leukemia**	Benzene	0.18 (0.05-0.35)	0.42 (0.08-0.76)
**Guinea**	**Acute lymphoid leukemia**	Formaldehyde	0.05 (0.03-0.08)	0.87 (0.25-1.49)
**Guinea**	**Chronic myeloid leukemia**	Benzene	0.46 (0.13-0.77)	0.6 (0.11-1.07)
**Guinea**	**Chronic myeloid leukemia**	Formaldehyde	0.14 (0.1-0.19)	0.58 (0.16-1.03)
**Guinea**	**Chronic lymphoid leukemia**	Benzene	0.13 (0.04-0.22)	0.96 (0.27-1.64)
**Guinea**	**Chronic lymphoid leukemia**	Formaldehyde	0.04 (0.03-0.05)	0.95 (0.3-1.66)

Countries	Causes	Occupational carcinogens	Percent change in 1990 (95% UI)	Percent change in 2019 (95% UI)
**Guinea**	**Other leukemia**	Benzene	0.3 (0.08-0.51)	0.93 (0.27-1.66)
**Guinea**	**Other leukemia**	Formaldehyde	0.09 (0.06-0.12)	1.44 (0.42-2.4)
**Guinea-Bissau**	**All leukemia**	Benzene	0.36 (0.11-0.61)	0.72 (0.21-1.21)
**Guinea-Bissau**	**All leukemia**	Formaldehyde	0.13 (0.09-0.16)	0.64 (0.18-1.14)
**Guinea-Bissau**	**Acute myeloid leukemia**	Benzene	0.31 (0.09-0.6)	0.48 (0.09-0.88)
**Guinea-Bissau**	**Acute myeloid leukemia**	Formaldehyde	0.11 (0.06-0.17)	0.86 (0.24-1.48)
**Guinea-Bissau**	**Acute lymphoid leukemia**	Benzene	0.23 (0.06-0.44)	0.61 (0.19-1.09)
**Guinea-Bissau**	**Acute lymphoid leukemia**	Formaldehyde	0.08 (0.05-0.13)	0.55 (0.16-0.96)
**Guinea-Bissau**	**Chronic myeloid leukemia**	Benzene	0.74 (0.21-1.23)	0.84 (0.25-1.43)
**Guinea-Bissau**	**Chronic myeloid leukemia**	Formaldehyde	0.26 (0.19-0.35)	0.46 (0.09-0.84)
**Guinea-Bissau**	**Chronic lymphoid leukemia**	Benzene	0.17 (0.05-0.29)	0.67 (0.12-1.2)
**Guinea-Bissau**	**Chronic lymphoid leukemia**	Formaldehyde	0.06 (0.04-0.08)	0.37 (0.11-0.7)
**Guinea-Bissau**	**Other leukemia**	Benzene	0.36 (0.1-0.6)	0.65 (0.19-1.19)
**Guinea-Bissau**	**Other leukemia**	Formaldehyde	0.13 (0.09-0.16)	1.1 (0.33-1.82)
**Guyana**	**All leukemia**	Benzene	0.69 (0.21-1.15)	1.23 (0.37-2.22)
**Guyana**	**All leukemia**	Formaldehyde	0.25 (0.2-0.31)	0.3 (0.08-0.54)
**Guyana**	**Acute myeloid leukemia**	Benzene	0.86 (0.25-1.44)	0.57 (0.11-1.03)
**Guyana**	**Acute myeloid leukemia**	Formaldehyde	0.31 (0.24-0.41)	1.06 (0.3-1.81)
**Guyana**	**Acute lymphoid leukemia**	Benzene	0.47 (0.14-0.83)	0.34 (0.1-0.6)
**Guyana**	**Acute lymphoid leukemia**	Formaldehyde	0.17 (0.13-0.24)	1.06 (0.31-1.76)
**Guyana**	**Chronic myeloid leukemia**	Benzene	0.95 (0.28-1.58)	1.02 (0.3-1.66)
**Guyana**	**Chronic myeloid leukemia**	Formaldehyde	0.35 (0.27-0.44)	1.09 (0.31-1.95)
**Guyana**	**Chronic lymphoid leukemia**	Benzene	0.45 (0.13-0.74)	0.58 (0.11-1.03)
**Guyana**	**Chronic lymphoid leukemia**	Formaldehyde	0.16 (0.13-0.2)	0.75 (0.22-1.29)
**Guyana**	**Other leukemia**	Benzene	0.7 (0.21-1.15)	0.51 (0.1-0.92)
**Guyana**	**Other leukemia**	Formaldehyde	0.25 (0.2-0.31)	0.61 (0.18-1.05)
**Haiti**	**All leukemia**	Benzene	0.26 (0.07-0.47)	0.2 (0.05-0.48)
**Haiti**	**All leukemia**	Formaldehyde	0.1 (0.06-0.13)	0.5 (0.15-0.86)
**Haiti**	**Acute myeloid leukemia**	Benzene	0.34 (0.09-0.65)	0.65 (0.19-1.21)
**Haiti**	**Acute myeloid leukemia**	Formaldehyde	0.13 (0.07-0.19)	1.09 (0.31-1.85)
**Haiti**	**Acute lymphoid leukemia**	Benzene	0.12 (0.03-0.25)	0.6 (0.19-1.05)
**Haiti**	**Acute lymphoid leukemia**	Formaldehyde	0.04 (0.02-0.07)	0.63 (0.12-1.14)
**Haiti**	**Chronic myeloid leukemia**	Benzene	0.36 (0.09-0.7)	0.65 (0.13-1.17)
**Haiti**	**Chronic myeloid leukemia**	Formaldehyde	0.13 (0.06-0.21)	0.89 (0.26-1.51)
**Haiti**	**Chronic lymphoid leukemia**	Benzene	0.29 (0.08-0.49)	0.24 (0.06-0.47)
**Haiti**	**Chronic lymphoid leukemia**	Formaldehyde	0.1 (0.08-0.13)	0.5 (0.13-0.9)
**Haiti**	**Other leukemia**	Benzene	0.29 (0.08-0.55)	0.87 (0.25-1.5)
**Haiti**	**Other leukemia**	Formaldehyde	0.11 (0.07-0.16)	0.45 (0.09-0.82)
**Honduras**	**All leukemia**	Benzene	0.47 (0.13-0.8)	1.06 (0.3-1.8)
**Honduras**	**All leukemia**	Formaldehyde	0.2 (0.15-0.25)	0.73 (0.14-1.29)
**Honduras**	**Acute myeloid leukemia**	Benzene	0.55 (0.15-0.95)	1.16 (0.34-2)
**Honduras**	**Acute myeloid leukemia**	Formaldehyde	0.23 (0.17-0.31)	0.32 (0.1-0.58)
**Honduras**	**Acute lymphoid leukemia**	Benzene	0.32 (0.09-0.55)	1.26 (0.36-2.15)
**Honduras**	**Acute lymphoid leukemia**	Formaldehyde	0.13 (0.09-0.18)	0.72 (0.2-1.26)

Countries	Causes	Occupational carcinogens	Percent change in 1990 (95% UI)	Percent change in 2019 (95% UI)
**Honduras**	**Chronic myeloid leukemia**	Benzene	0.82 (0.25-1.4)	1.1 (0.32-1.86)
**Honduras**	**Chronic myeloid leukemia**	Formaldehyde	0.35 (0.26-0.45)	0.38 (0.1-0.69)
**Honduras**	**Chronic lymphoid leukemia**	Benzene	0.44 (0.13-0.76)	1.13 (0.33-1.86)
**Honduras**	**Chronic lymphoid leukemia**	Formaldehyde	0.19 (0.14-0.25)	1.08 (0.32-1.8)
**Honduras**	**Other leukemia**	Benzene	0.52 (0.14-0.88)	0.68 (0.2-1.18)
**Honduras**	**Other leukemia**	Formaldehyde	0.22 (0.16-0.29)	0.56 (0.11-1)
**Hungary**	**All leukemia**	Benzene	0.3 (0.06-0.54)	0.53 (0.1-0.97)
**Hungary**	**All leukemia**	Formaldehyde	0.05 (0.04-0.06)	0.42 (0.08-0.77)
**Hungary**	**Acute myeloid leukemia**	Benzene	0.38 (0.07-0.67)	1.76 (0.54-2.97)
**Hungary**	**Acute myeloid leukemia**	Formaldehyde	0.06 (0.05-0.07)	0.73 (0.13-1.35)
**Hungary**	**Acute lymphoid leukemia**	Benzene	0.37 (0.07-0.67)	0.31 (0.06-0.57)
**Hungary**	**Acute lymphoid leukemia**	Formaldehyde	0.06 (0.05-0.07)	0.4 (0.07-0.73)
**Hungary**	**Chronic myeloid leukemia**	Benzene	0.39 (0.07-0.68)	0.58 (0.11-1.03)
**Hungary**	**Chronic myeloid leukemia**	Formaldehyde	0.06 (0.05-0.07)	0.34 (0.09-0.69)
**Hungary**	**Chronic lymphoid leukemia**	Benzene	0.16 (0.03-0.29)	0.78 (0.21-1.42)
**Hungary**	**Chronic lymphoid leukemia**	Formaldehyde	0.03 (0.02-0.03)	0.82 (0.24-1.38)
**Hungary**	**Other leukemia**	Benzene	0.28 (0.05-0.49)	0.76 (0.22-1.26)
**Hungary**	**Other leukemia**	Formaldehyde	0.04 (0.04-0.05)	0.61 (0.17-1.06)
**Iceland**	**All leukemia**	Benzene	0.54 (0.1-0.95)	0.51 (0.1-0.93)
**Iceland**	**All leukemia**	Formaldehyde	0.06 (0.05-0.07)	0.62 (0.16-1.14)
**Iceland**	**Acute myeloid leukemia**	Benzene	0.69 (0.13-1.23)	1.69 (0.53-2.89)
**Iceland**	**Acute myeloid leukemia**	Formaldehyde	0.07 (0.06-0.08)	0.35 (0.1-0.62)
**Iceland**	**Acute lymphoid leukemia**	Benzene	0.77 (0.14-1.36)	0.45 (0.08-0.82)
**Iceland**	**Acute lymphoid leukemia**	Formaldehyde	0.08 (0.07-0.1)	1.21 (0.36-2.03)
**Iceland**	**Chronic myeloid leukemia**	Benzene	0.66 (0.12-1.18)	0.31 (0.09-0.6)
**Iceland**	**Chronic myeloid leukemia**	Formaldehyde	0.07 (0.06-0.09)	0.97 (0.18-1.74)
**Iceland**	**Chronic lymphoid leukemia**	Benzene	0.31 (0.06-0.56)	0.56 (0.1-1.02)
**Iceland**	**Chronic lymphoid leukemia**	Formaldehyde	0.03 (0.02-0.04)	0.42 (0.08-0.77)
**Iceland**	**Other leukemia**	Benzene	0.47 (0.09-0.85)	1.07 (0.3-2.11)
**Iceland**	**Other leukemia**	Formaldehyde	0.05 (0.04-0.06)	0.23 (0.06-0.47)
**India**	**All leukemia**	Benzene	0.39 (0.11-0.68)	0.82 (0.23-1.46)
**India**	**All leukemia**	Formaldehyde	0.16 (0.12-0.21)	0.19 (0.05-0.42)
**India**	**Acute myeloid leukemia**	Benzene	0.46 (0.13-0.8)	0.45 (0.08-0.8)
**India**	**Acute myeloid leukemia**	Formaldehyde	0.18 (0.13-0.24)	0.81 (0.24-1.36)
**India**	**Acute lymphoid leukemia**	Benzene	0.28 (0.08-0.53)	0.55 (0.17-0.98)
**India**	**Acute lymphoid leukemia**	Formaldehyde	0.12 (0.07-0.17)	0.82 (0.25-1.38)
**India**	**Chronic myeloid leukemia**	Benzene	0.56 (0.15-1)	0.69 (0.14-1.23)
**India**	**Chronic myeloid leukemia**	Formaldehyde	0.22 (0.15-0.31)	0.68 (0.13-1.21)
**India**	**Chronic lymphoid leukemia**	Benzene	0.19 (0.06-0.33)	0.55 (0.16-0.96)
**India**	**Chronic lymphoid leukemia**	Formaldehyde	0.08 (0.06-0.09)	0.94 (0.28-1.55)
**India**	**Other leukemia**	Benzene	0.34 (0.1-0.63)	0.54 (0.15-0.92)
**India**	**Other leukemia**	Formaldehyde	0.13 (0.09-0.19)	0.82 (0.23-1.39)
**Indonesia**	**All leukemia**	Benzene	0.55 (0.16-0.94)	0.43 (0.13-0.79)
**Indonesia**	**All leukemia**	Formaldehyde	0.22 (0.17-0.28)	0.59 (0.17-1.05)

Countries	Causes	Occupational carcinogens	Percent change in 1990 (95% UI)	Percent change in 2019 (95% UI)
**Indonesia**	**Acute myeloid leukemia**	Benzene	0.69 (0.2-1.19)	1 (0.28-1.77)
**Indonesia**	**Acute myeloid leukemia**	Formaldehyde	0.28 (0.21-0.35)	0.6 (0.18-1.02)
**Indonesia**	**Acute lymphoid leukemia**	Benzene	0.37 (0.1-0.68)	0.89 (0.26-1.48)
**Indonesia**	**Acute lymphoid leukemia**	Formaldehyde	0.15 (0.1-0.22)	0.69 (0.2-1.15)
**Indonesia**	**Chronic myeloid leukemia**	Benzene	0.76 (0.22-1.28)	0.79 (0.23-1.35)
**Indonesia**	**Chronic myeloid leukemia**	Formaldehyde	0.31 (0.24-0.38)	0.65 (0.2-1.14)
**Indonesia**	**Chronic lymphoid leukemia**	Benzene	0.5 (0.15-0.82)	1.12 (0.32-1.91)
**Indonesia**	**Chronic lymphoid leukemia**	Formaldehyde	0.2 (0.16-0.24)	0.26 (0.07-0.53)
**Indonesia**	**Other leukemia**	Benzene	0.56 (0.16-0.95)	0.47 (0.09-0.86)
**Indonesia**	**Other leukemia**	Formaldehyde	0.23 (0.16-0.28)	2.23 (0.68-3.74)
**Iran (Islamic Republic of)**	**All leukemia**	Benzene	0.31 (0.09-0.53)	0.71 (0.14-1.26)
**Iran (Islamic Republic of)**	**All leukemia**	Formaldehyde	0.14 (0.11-0.17)	0.28 (0.08-0.56)
**Iran (Islamic Republic of)**	**Acute myeloid leukemia**	Benzene	0.39 (0.11-0.67)	0.65 (0.12-1.17)
**Iran (Islamic Republic of)**	**Acute myeloid leukemia**	Formaldehyde	0.17 (0.12-0.22)	0.79 (0.23-1.35)
**Iran (Islamic Republic of)**	**Acute lymphoid leukemia**	Benzene	0.27 (0.08-0.47)	1.06 (0.3-1.76)
**Iran (Islamic Republic of)**	**Acute lymphoid leukemia**	Formaldehyde	0.12 (0.08-0.16)	1.05 (0.31-1.73)
**Iran (Islamic Republic of)**	**Chronic myeloid leukemia**	Benzene	0.41 (0.12-0.69)	0.97 (0.29-1.67)
**Iran (Islamic Republic of)**	**Chronic myeloid leukemia**	Formaldehyde	0.17 (0.13-0.22)	0.86 (0.25-1.46)
**Iran (Islamic Republic of)**	**Chronic lymphoid leukemia**	Benzene	0.37 (0.11-0.63)	0.9 (0.27-1.5)
**Iran (Islamic Republic of)**	**Chronic lymphoid leukemia**	Formaldehyde	0.16 (0.12-0.2)	0.47 (0.14-0.86)
**Iran (Islamic Republic of)**	**Other leukemia**	Benzene	0.26 (0.08-0.44)	0.39 (0.11-0.81)
**Iran (Islamic Republic of)**	**Other leukemia**	Formaldehyde	0.11 (0.09-0.14)	0.68 (0.2-1.18)
**Iraq**	**All leukemia**	Benzene	0.44 (0.13-0.73)	0.61 (0.19-1.1)
**Iraq**	**All leukemia**	Formaldehyde	0.12 (0.1-0.16)	0.42 (0.08-0.77)
**Iraq**	**Acute myeloid leukemia**	Benzene	0.51 (0.15-0.91)	0.68 (0.2-1.15)
**Iraq**	**Acute myeloid leukemia**	Formaldehyde	0.15 (0.09-0.22)	1.13 (0.33-1.87)
**Iraq**	**Acute lymphoid leukemia**	Benzene	0.34 (0.1-0.67)	0.47 (0.09-0.84)
**Iraq**	**Acute lymphoid leukemia**	Formaldehyde	0.1 (0.06-0.16)	0.89 (0.26-1.55)
**Iraq**	**Chronic myeloid leukemia**	Benzene	0.66 (0.19-1.17)	1.25 (0.38-2.09)
**Iraq**	**Chronic myeloid leukemia**	Formaldehyde	0.19 (0.13-0.28)	1.01 (0.3-1.63)
**Iraq**	**Chronic lymphoid leukemia**	Benzene	0.42 (0.12-0.74)	0.79 (0.22-1.33)
**Iraq**	**Chronic lymphoid leukemia**	Formaldehyde	0.12 (0.09-0.17)	0.36 (0.07-0.65)
**Iraq**	**Other leukemia**	Benzene	0.41 (0.12-0.69)	0.37 (0.07-0.67)
**Iraq**	**Other leukemia**	Formaldehyde	0.12 (0.09-0.15)	1.19 (0.36-1.99)
**Ireland**	**All leukemia**	Benzene	0.25 (0.05-0.44)	1.7 (0.51-2.84)
**Ireland**	**All leukemia**	Formaldehyde	0.03 (0.03-0.04)	1.36 (0.39-2.35)
**Ireland**	**Acute myeloid leukemia**	Benzene	0.3 (0.06-0.53)	0.63 (0.18-1.08)
**Ireland**	**Acute myeloid leukemia**	Formaldehyde	0.04 (0.03-0.04)	1.09 (0.32-1.79)
**Ireland**	**Acute lymphoid leukemia**	Benzene	0.36 (0.07-0.64)	0.51 (0.09-0.91)
**Ireland**	**Acute lymphoid leukemia**	Formaldehyde	0.05 (0.04-0.06)	0.34 (0.06-0.61)
**Ireland**	**Chronic myeloid leukemia**	Benzene	0.31 (0.06-0.56)	1.55 (0.46-2.59)
**Ireland**	**Chronic myeloid leukemia**	Formaldehyde	0.04 (0.03-0.05)	0.49 (0.13-0.9)
**Ireland**	**Chronic lymphoid leukemia**	Benzene	0.14 (0.03-0.25)	1.02 (0.3-1.71)
**Ireland**	**Chronic lymphoid leukemia**	Formaldehyde	0.02 (0.01-0.02)	0.84 (0.24-1.42)

Countries	Causes	Occupational carcinogens	Percent change in 1990 (95% UI)	Percent change in 2019 (95% UI)
**Ireland**	**Other leukemia**	Benzene	0.17 (0.03-0.31)	1.06 (0.32-1.78)
**Ireland**	**Other leukemia**	Formaldehyde	0.02 (0.02-0.03)	0.3 (0.05-0.54)
**Israel**	**All leukemia**	Benzene	0.31 (0.06-0.55)	1.32 (0.38-2.3)
**Israel**	**All leukemia**	Formaldehyde	0.04 (0.03-0.04)	0.96 (0.28-1.61)
**Israel**	**Acute myeloid leukemia**	Benzene	0.37 (0.07-0.66)	1.29 (0.23-2.34)
**Israel**	**Acute myeloid leukemia**	Formaldehyde	0.04 (0.04-0.05)	0.49 (0.09-0.88)
**Israel**	**Acute lymphoid leukemia**	Benzene	0.48 (0.09-0.85)	0.27 (0.07-0.53)
**Israel**	**Acute lymphoid leukemia**	Formaldehyde	0.06 (0.05-0.07)	0.66 (0.19-1.21)
**Israel**	**Chronic myeloid leukemia**	Benzene	0.37 (0.07-0.66)	0.57 (0.17-0.99)
**Israel**	**Chronic myeloid leukemia**	Formaldehyde	0.04 (0.03-0.05)	0.87 (0.26-1.42)
**Israel**	**Chronic lymphoid leukemia**	Benzene	0.17 (0.03-0.3)	0.71 (0.2-1.26)
**Israel**	**Chronic lymphoid leukemia**	Formaldehyde	0.02 (0.02-0.03)	0.37 (0.07-0.66)
**Israel**	**Other leukemia**	Benzene	0.25 (0.05-0.46)	0.56 (0.16-0.98)
**Israel**	**Other leukemia**	Formaldehyde	0.03 (0.02-0.04)	0.3 (0.08-0.56)
**Italy**	**All leukemia**	Benzene	0.29 (0.05-0.52)	0.88 (0.26-1.48)
**Italy**	**All leukemia**	Formaldehyde	0.04 (0.03-0.05)	0.7 (0.2-1.15)
**Italy**	**Acute myeloid leukemia**	Benzene	0.35 (0.07-0.63)	1.14 (0.33-1.9)
**Italy**	**Acute myeloid leukemia**	Formaldehyde	0.05 (0.04-0.06)	0.86 (0.26-1.49)
**Italy**	**Acute lymphoid leukemia**	Benzene	0.43 (0.08-0.76)	0.89 (0.25-1.55)
**Italy**	**Acute lymphoid leukemia**	Formaldehyde	0.06 (0.05-0.07)	1.23 (0.36-2.03)
**Italy**	**Chronic myeloid leukemia**	Benzene	0.34 (0.06-0.62)	0.97 (0.29-1.63)
**Italy**	**Chronic myeloid leukemia**	Formaldehyde	0.05 (0.04-0.06)	0.29 (0.05-0.53)
**Italy**	**Chronic lymphoid leukemia**	Benzene	0.16 (0.03-0.28)	1.03 (0.31-1.72)
**Italy**	**Chronic lymphoid leukemia**	Formaldehyde	0.02 (0.02-0.03)	0.39 (0.07-0.77)
**Italy**	**Other leukemia**	Benzene	0.27 (0.05-0.48)	0.39 (0.07-0.7)
**Italy**	**Other leukemia**	Formaldehyde	0.04 (0.03-0.04)	0.56 (0.16-0.96)
**Jamaica**	**All leukemia**	Benzene	0.57 (0.17-0.96)	0.74 (0.21-1.29)
**Jamaica**	**All leukemia**	Formaldehyde	0.2 (0.17-0.24)	0.81 (0.22-1.34)
**Jamaica**	**Acute myeloid leukemia**	Benzene	0.59 (0.17-1.04)	0.42 (0.08-0.75)
**Jamaica**	**Acute myeloid leukemia**	Formaldehyde	0.21 (0.15-0.28)	0.57 (0.17-1)
**Jamaica**	**Acute lymphoid leukemia**	Benzene	0.27 (0.08-0.49)	0.97 (0.28-1.63)
**Jamaica**	**Acute lymphoid leukemia**	Formaldehyde	0.1 (0.06-0.14)	1.24 (0.37-2.13)
**Jamaica**	**Chronic myeloid leukemia**	Benzene	0.63 (0.19-1.07)	1.17 (0.33-1.95)
**Jamaica**	**Chronic myeloid leukemia**	Formaldehyde	0.23 (0.18-0.29)	0.91 (0.26-1.55)
**Jamaica**	**Chronic lymphoid leukemia**	Benzene	0.39 (0.11-0.66)	1.09 (0.32-1.85)
**Jamaica**	**Chronic lymphoid leukemia**	Formaldehyde	0.14 (0.11-0.17)	1.19 (0.35-2.08)
**Jamaica**	**Other leukemia**	Benzene	0.67 (0.19-1.12)	0.76 (0.22-1.35)
**Jamaica**	**Other leukemia**	Formaldehyde	0.24 (0.19-0.29)	0.38 (0.07-0.69)
**Japan**	**All leukemia**	Benzene	0.56 (0.11-0.98)	0.81 (0.24-1.42)
**Japan**	**All leukemia**	Formaldehyde	0.08 (0.07-0.09)	0.76 (0.21-1.35)
**Japan**	**Acute myeloid leukemia**	Benzene	0.59 (0.11-1.04)	1.02 (0.3-1.72)
**Japan**	**Acute myeloid leukemia**	Formaldehyde	0.08 (0.07-0.1)	0.39 (0.07-0.69)
**Japan**	**Acute lymphoid leukemia**	Benzene	0.64 (0.12-1.14)	0.32 (0.06-0.6)
**Japan**	**Acute lymphoid leukemia**	Formaldehyde	0.09 (0.08-0.11)	0.62 (0.18-1.08)

Countries	Causes	Occupational carcinogens	Percent change in 1990 (95% UI)	Percent change in 2019 (95% UI)
**Japan**	**Chronic myeloid leukemia**	Benzene	0.68 (0.13-1.21)	0.64 (0.19-1.12)
**Japan**	**Chronic myeloid leukemia**	Formaldehyde	0.1 (0.08-0.12)	0.81 (0.24-1.35)
**Japan**	**Chronic lymphoid leukemia**	Benzene	0.29 (0.05-0.51)	0.4 (0.07-0.72)
**Japan**	**Chronic lymphoid leukemia**	Formaldehyde	0.04 (0.03-0.05)	0.77 (0.21-1.28)
**Japan**	**Other leukemia**	Benzene	0.44 (0.08-0.79)	0.23 (0.05-0.42)
**Japan**	**Other leukemia**	Formaldehyde	0.06 (0.05-0.07)	0.38 (0.07-0.69)
**Jordan**	**All leukemia**	Benzene	0.39 (0.11-0.67)	1.25 (0.38-2.07)
**Jordan**	**All leukemia**	Formaldehyde	0.14 (0.11-0.17)	1.25 (0.37-2.06)
**Jordan**	**Acute myeloid leukemia**	Benzene	0.46 (0.13-0.82)	1.18 (0.34-1.96)
**Jordan**	**Acute myeloid leukemia**	Formaldehyde	0.16 (0.11-0.23)	0.38 (0.11-0.66)
**Jordan**	**Acute lymphoid leukemia**	Benzene	0.34 (0.09-0.62)	0.56 (0.16-0.97)
**Jordan**	**Acute lymphoid leukemia**	Formaldehyde	0.12 (0.07-0.18)	1.19 (0.34-1.97)
**Jordan**	**Chronic myeloid leukemia**	Benzene	0.57 (0.16-0.98)	0.65 (0.2-1.15)
**Jordan**	**Chronic myeloid leukemia**	Formaldehyde	0.2 (0.14-0.27)	0.91 (0.26-1.55)
**Jordan**	**Chronic lymphoid leukemia**	Benzene	0.41 (0.12-0.71)	0.41 (0.08-0.74)
**Jordan**	**Chronic lymphoid leukemia**	Formaldehyde	0.14 (0.1-0.2)	0.61 (0.12-1.11)
**Jordan**	**Other leukemia**	Benzene	0.39 (0.11-0.66)	0.63 (0.18-1.03)
**Jordan**	**Other leukemia**	Formaldehyde	0.13 (0.11-0.17)	1.02 (0.31-1.7)
**Kazakhstan**	**All leukemia**	Benzene	0.71 (0.21-1.18)	0.71 (0.21-1.19)
**Kazakhstan**	**All leukemia**	Formaldehyde	0.22 (0.18-0.26)	0.87 (0.25-1.43)
**Kazakhstan**	**Acute myeloid leukemia**	Benzene	0.89 (0.25-1.47)	0.4 (0.07-0.72)
**Kazakhstan**	**Acute myeloid leukemia**	Formaldehyde	0.27 (0.22-0.34)	0.51 (0.09-0.91)
**Kazakhstan**	**Acute lymphoid leukemia**	Benzene	0.63 (0.18-1.05)	0.27 (0.05-0.49)
**Kazakhstan**	**Acute lymphoid leukemia**	Formaldehyde	0.19 (0.15-0.24)	1.07 (0.3-1.79)
**Kazakhstan**	**Chronic myeloid leukemia**	Benzene	0.9 (0.27-1.5)	0.36 (0.07-0.65)
**Kazakhstan**	**Chronic myeloid leukemia**	Formaldehyde	0.28 (0.23-0.34)	0.67 (0.19-1.16)
**Kazakhstan**	**Chronic lymphoid leukemia**	Benzene	0.67 (0.2-1.12)	1.11 (0.33-1.85)
**Kazakhstan**	**Chronic lymphoid leukemia**	Formaldehyde	0.2 (0.16-0.24)	1.53 (0.47-2.57)
**Kazakhstan**	**Other leukemia**	Benzene	0.51 (0.14-0.88)	1.12 (0.32-1.9)
**Kazakhstan**	**Other leukemia**	Formaldehyde	0.15 (0.12-0.21)	1.81 (0.53-3.04)
**Kenya**	**All leukemia**	Benzene	0.46 (0.13-0.85)	1.02 (0.3-1.69)
**Kenya**	**All leukemia**	Formaldehyde	0.18 (0.12-0.26)	0.97 (0.28-1.63)
**Kenya**	**Acute myeloid leukemia**	Benzene	0.7 (0.2-1.31)	0.43 (0.08-0.81)
**Kenya**	**Acute myeloid leukemia**	Formaldehyde	0.27 (0.18-0.4)	0.87 (0.24-1.47)
**Kenya**	**Acute lymphoid leukemia**	Benzene	0.43 (0.11-0.8)	1 (0.29-1.74)
**Kenya**	**Acute lymphoid leukemia**	Formaldehyde	0.17 (0.08-0.25)	0.7 (0.2-1.17)
**Kenya**	**Chronic myeloid leukemia**	Benzene	0.71 (0.16-1.56)	0.9 (0.27-1.52)
**Kenya**	**Chronic myeloid leukemia**	Formaldehyde	0.27 (0.09-0.47)	0.41 (0.08-0.77)
**Kenya**	**Chronic lymphoid leukemia**	Benzene	0.35 (0.1-0.59)	0.47 (0.09-0.82)
**Kenya**	**Chronic lymphoid leukemia**	Formaldehyde	0.13 (0.11-0.17)	0.74 (0.22-1.3)
**Kenya**	**Other leukemia**	Benzene	0.39 (0.1-0.77)	1.1 (0.32-1.92)
**Kenya**	**Other leukemia**	Formaldehyde	0.15 (0.09-0.24)	1.09 (0.31-1.81)
**Kiribati**	**All leukemia**	Benzene	0.43 (0.12-0.73)	1.23 (0.35-2.16)
**Kiribati**	**All leukemia**	Formaldehyde	0.1 (0.08-0.13)	0.35 (0.1-0.65)

Countries	Causes	Occupational carcinogens	Percent change in 1990 (95% UI)	Percent change in 2019 (95% UI)
**Kiribati**	**Acute myeloid leukemia**	Benzene	0.53 (0.16-0.9)	0.4 (0.08-0.71)
**Kiribati**	**Acute myeloid leukemia**	Formaldehyde	0.13 (0.09-0.17)	1.29 (0.38-2.19)
**Kiribati**	**Acute lymphoid leukemia**	Benzene	0.41 (0.12-0.71)	0.41 (0.12-0.72)
**Kiribati**	**Acute lymphoid leukemia**	Formaldehyde	0.1 (0.06-0.14)	1.02 (0.29-1.68)
**Kiribati**	**Chronic myeloid leukemia**	Benzene	0.49 (0.14-0.86)	1.13 (0.33-1.85)
**Kiribati**	**Chronic myeloid leukemia**	Formaldehyde	0.12 (0.08-0.16)	1.31 (0.39-2.29)
**Kiribati**	**Chronic lymphoid leukemia**	Benzene	0.27 (0.07-0.46)	0.35 (0.07-0.66)
**Kiribati**	**Chronic lymphoid leukemia**	Formaldehyde	0.06 (0.05-0.09)	1.21 (0.36-1.97)
**Kiribati**	**Other leukemia**	Benzene	0.41 (0.12-0.71)	0.45 (0.08-0.8)
**Kiribati**	**Other leukemia**	Formaldehyde	0.1 (0.07-0.13)	0.72 (0.22-1.2)
**Kuwait**	**All leukemia**	Benzene	0.96 (0.29-1.61)	0.46 (0.12-0.92)
**Kuwait**	**All leukemia**	Formaldehyde	0.24 (0.2-0.3)	0.71 (0.21-1.19)
**Kuwait**	**Acute myeloid leukemia**	Benzene	1.13 (0.35-1.93)	0.93 (0.28-1.61)
**Kuwait**	**Acute myeloid leukemia**	Formaldehyde	0.28 (0.22-0.37)	1.59 (0.48-2.7)
**Kuwait**	**Acute lymphoid leukemia**	Benzene	0.67 (0.21-1.18)	0.8 (0.24-1.33)
**Kuwait**	**Acute lymphoid leukemia**	Formaldehyde	0.17 (0.12-0.24)	0.45 (0.09-0.81)
**Kuwait**	**Chronic myeloid leukemia**	Benzene	1.34 (0.39-2.25)	0.49 (0.09-0.87)
**Kuwait**	**Chronic myeloid leukemia**	Formaldehyde	0.36 (0.27-0.48)	1.06 (0.31-1.78)
**Kuwait**	**Chronic lymphoid leukemia**	Benzene	0.67 (0.2-1.16)	0.34 (0.08-0.68)
**Kuwait**	**Chronic lymphoid leukemia**	Formaldehyde	0.18 (0.14-0.23)	0.56 (0.15-0.97)
**Kuwait**	**Other leukemia**	Benzene	0.99 (0.3-1.64)	0.95 (0.27-1.65)
**Kuwait**	**Other leukemia**	Formaldehyde	0.24 (0.19-0.3)	0.43 (0.08-0.78)
**Kyrgyzstan**	**All leukemia**	Benzene	0.75 (0.22-1.25)	1.04 (0.3-1.74)
**Kyrgyzstan**	**All leukemia**	Formaldehyde	0.26 (0.21-0.32)	0.54 (0.1-0.95)
**Kyrgyzstan**	**Acute myeloid leukemia**	Benzene	0.81 (0.23-1.38)	1.34 (0.39-2.25)
**Kyrgyzstan**	**Acute myeloid leukemia**	Formaldehyde	0.29 (0.22-0.38)	0.61 (0.18-1.05)
**Kyrgyzstan**	**Acute lymphoid leukemia**	Benzene	0.48 (0.14-0.8)	1.39 (0.4-2.32)
**Kyrgyzstan**	**Acute lymphoid leukemia**	Formaldehyde	0.17 (0.13-0.22)	0.94 (0.26-1.65)
**Kyrgyzstan**	**Chronic myeloid leukemia**	Benzene	0.84 (0.24-1.4)	1.07 (0.31-1.8)
**Kyrgyzstan**	**Chronic myeloid leukemia**	Formaldehyde	0.29 (0.24-0.37)	0.7 (0.2-1.19)
**Kyrgyzstan**	**Chronic lymphoid leukemia**	Benzene	0.86 (0.25-1.45)	1.29 (0.39-2.13)
**Kyrgyzstan**	**Chronic lymphoid leukemia**	Formaldehyde	0.29 (0.23-0.36)	1.22 (0.36-2.03)
**Kyrgyzstan**	**Other leukemia**	Benzene	0.82 (0.23-1.37)	1.08 (0.32-1.8)
**Kyrgyzstan**	**Other leukemia**	Formaldehyde	0.28 (0.22-0.35)	0.4 (0.08-0.74)
**Lao People's Democratic Republic**	**All leukemia**	Benzene	0.43 (0.12-0.72)	0.39 (0.08-0.69)
**Lao People's Democratic Republic**	**All leukemia**	Formaldehyde	0.18 (0.13-0.24)	0.36 (0.07-0.66)
**Lao People's Democratic Republic**	**Acute myeloid leukemia**	Benzene	0.54 (0.15-0.95)	2.03 (0.6-3.4)
**Lao People's Democratic Republic**	**Acute myeloid leukemia**	Formaldehyde	0.22 (0.14-0.32)	0.58 (0.11-1.04)
**Lao People's Democratic Republic**	**Acute lymphoid leukemia**	Benzene	0.25 (0.06-0.5)	0.37 (0.07-0.66)
**Lao People's Democratic Republic**	**Acute lymphoid leukemia**	Formaldehyde	0.1 (0.06-0.17)	0.39 (0.07-0.69)
**Lao People's Democratic Republic**	**Chronic myeloid leukemia**	Benzene	0.6 (0.17-1.09)	0.63 (0.11-1.12)
**Lao People's Democratic Republic**	**Chronic myeloid leukemia**	Formaldehyde	0.25 (0.17-0.39)	0.66 (0.19-1.19)
**Lao People's Democratic Republic**	**Chronic lymphoid leukemia**	Benzene	0.43 (0.12-0.74)	1.09 (0.31-1.92)
**Lao People's Democratic Republic**	**Chronic lymphoid leukemia**	Formaldehyde	0.18 (0.13-0.26)	1.1 (0.32-1.83)

Countries	Causes	Occupational carcinogens	Percent change in 1990 (95% UI)	Percent change in 2019 (95% UI)
**Lao People's Democratic Republic**	**Other leukemia**	Benzene	0.44 (0.12-0.76)	1.03 (0.3-1.68)
**Lao People's Democratic Republic**	**Other leukemia**	Formaldehyde	0.19 (0.12-0.25)	0.68 (0.19-1.16)
**Latvia**	**All leukemia**	Benzene	0.34 (0.06-0.61)	0.34 (0.06-0.61)
**Latvia**	**All leukemia**	Formaldehyde	0.04 (0.03-0.05)	0.63 (0.19-1.09)
**Latvia**	**Acute myeloid leukemia**	Benzene	0.43 (0.08-0.77)	1.66 (0.49-2.76)
**Latvia**	**Acute myeloid leukemia**	Formaldehyde	0.05 (0.04-0.06)	0.42 (0.13-0.73)
**Latvia**	**Acute lymphoid leukemia**	Benzene	0.34 (0.06-0.62)	0.45 (0.09-0.82)
**Latvia**	**Acute lymphoid leukemia**	Formaldehyde	0.04 (0.03-0.05)	1.25 (0.35-2.13)
**Latvia**	**Chronic myeloid leukemia**	Benzene	0.36 (0.07-0.65)	0.4 (0.11-0.72)
**Latvia**	**Chronic myeloid leukemia**	Formaldehyde	0.04 (0.03-0.05)	0.63 (0.12-1.13)
**Latvia**	**Chronic lymphoid leukemia**	Benzene	0.18 (0.03-0.32)	0.47 (0.09-0.88)
**Latvia**	**Chronic lymphoid leukemia**	Formaldehyde	0.02 (0.02-0.03)	0.3 (0.06-0.56)
**Latvia**	**Other leukemia**	Benzene	0.42 (0.08-0.75)	1.55 (0.47-2.73)
**Latvia**	**Other leukemia**	Formaldehyde	0.05 (0.04-0.06)	0.52 (0.15-0.97)
**Lebanon**	**All leukemia**	Benzene	0.44 (0.13-0.75)	1.01 (0.28-1.71)
**Lebanon**	**All leukemia**	Formaldehyde	0.16 (0.12-0.2)	0.44 (0.12-0.87)
**Lebanon**	**Acute myeloid leukemia**	Benzene	0.53 (0.15-0.93)	0.3 (0.06-0.54)
**Lebanon**	**Acute myeloid leukemia**	Formaldehyde	0.19 (0.14-0.25)	0.78 (0.22-1.28)
**Lebanon**	**Acute lymphoid leukemia**	Benzene	0.45 (0.13-0.79)	0.82 (0.24-1.42)
**Lebanon**	**Acute lymphoid leukemia**	Formaldehyde	0.16 (0.11-0.22)	1.23 (0.38-2.03)
**Lebanon**	**Chronic myeloid leukemia**	Benzene	0.56 (0.15-0.96)	0.44 (0.08-0.79)
**Lebanon**	**Chronic myeloid leukemia**	Formaldehyde	0.2 (0.14-0.27)	0.47 (0.09-0.85)
**Lebanon**	**Chronic lymphoid leukemia**	Benzene	0.34 (0.09-0.58)	0.57 (0.16-0.96)
**Lebanon**	**Chronic lymphoid leukemia**	Formaldehyde	0.12 (0.09-0.16)	0.74 (0.21-1.27)
**Lebanon**	**Other leukemia**	Benzene	0.34 (0.1-0.59)	0.77 (0.22-1.32)
**Lebanon**	**Other leukemia**	Formaldehyde	0.12 (0.09-0.16)	0.78 (0.22-1.32)
**Lesotho**	**All leukemia**	Benzene	0.45 (0.13-0.76)	0.7 (0.2-1.24)
**Lesotho**	**All leukemia**	Formaldehyde	0.14 (0.11-0.18)	0.65 (0.19-1.09)
**Lesotho**	**Acute myeloid leukemia**	Benzene	0.72 (0.2-1.27)	1.06 (0.3-1.81)
**Lesotho**	**Acute myeloid leukemia**	Formaldehyde	0.23 (0.17-0.32)	0.69 (0.2-1.17)
**Lesotho**	**Acute lymphoid leukemia**	Benzene	0.41 (0.11-0.81)	1.07 (0.3-1.77)
**Lesotho**	**Acute lymphoid leukemia**	Formaldehyde	0.13 (0.08-0.2)	0.71 (0.2-1.19)
**Lesotho**	**Chronic myeloid leukemia**	Benzene	0.58 (0.16-1.03)	0.66 (0.19-1.09)
**Lesotho**	**Chronic myeloid leukemia**	Formaldehyde	0.19 (0.12-0.26)	0.9 (0.26-1.53)
**Lesotho**	**Chronic lymphoid leukemia**	Benzene	0.32 (0.09-0.54)	1.21 (0.35-2.04)
**Lesotho**	**Chronic lymphoid leukemia**	Formaldehyde	0.11 (0.08-0.14)	0.55 (0.16-1.01)
**Lesotho**	**Other leukemia**	Benzene	0.46 (0.13-0.78)	0.51 (0.1-0.92)
**Lesotho**	**Other leukemia**	Formaldehyde	0.15 (0.11-0.19)	2.05 (0.61-3.48)
**Liberia**	**All leukemia**	Benzene	0.31 (0.08-0.59)	0.4 (0.08-0.72)
**Liberia**	**All leukemia**	Formaldehyde	0.11 (0.07-0.16)	0.61 (0.18-1.16)
**Liberia**	**Acute myeloid leukemia**	Benzene	0.31 (0.07-0.67)	0.39 (0.08-0.71)
**Liberia**	**Acute myeloid leukemia**	Formaldehyde	0.11 (0.05-0.19)	1.02 (0.29-1.7)
**Liberia**	**Acute lymphoid leukemia**	Benzene	0.21 (0.05-0.46)	0.93 (0.27-1.57)
**Liberia**	**Acute lymphoid leukemia**	Formaldehyde	0.08 (0.03-0.13)	1.32 (0.39-2.18)

Countries	Causes	Occupational carcinogens	Percent change in 1990 (95% UI)	Percent change in 2019 (95% UI)
**Liberia**	**Chronic myeloid leukemia**	Benzene	0.64 (0.18-1.14)	1.21 (0.36-2.04)
**Liberia**	**Chronic myeloid leukemia**	Formaldehyde	0.23 (0.16-0.32)	1.02 (0.3-1.68)
**Liberia**	**Chronic lymphoid leukemia**	Benzene	0.17 (0.05-0.29)	0.84 (0.25-1.42)
**Liberia**	**Chronic lymphoid leukemia**	Formaldehyde	0.06 (0.04-0.08)	0.64 (0.19-1.19)
**Liberia**	**Other leukemia**	Benzene	0.3 (0.08-0.56)	0.82 (0.25-1.5)
**Liberia**	**Other leukemia**	Formaldehyde	0.11 (0.07-0.17)	1.01 (0.29-1.69)
**Libya**	**All leukemia**	Benzene	0.42 (0.12-0.7)	0.14 (0.09-0.22)
**Libya**	**All leukemia**	Formaldehyde	0.12 (0.09-0.16)	0.04 (0.03-0.05)
**Libya**	**Acute myeloid leukemia**	Benzene	0.5 (0.14-0.89)	0.14 (0.1-0.2)
**Libya**	**Acute myeloid leukemia**	Formaldehyde	0.15 (0.11-0.2)	0.32 (0.24-0.43)
**Libya**	**Acute lymphoid leukemia**	Benzene	0.36 (0.11-0.65)	0.08 (0.06-0.1)
**Libya**	**Acute lymphoid leukemia**	Formaldehyde	0.11 (0.07-0.16)	0.15 (0.09-0.23)
**Libya**	**Chronic myeloid leukemia**	Benzene	0.6 (0.17-1.03)	0.22 (0.17-0.27)
**Libya**	**Chronic myeloid leukemia**	Formaldehyde	0.18 (0.13-0.25)	0.3 (0.25-0.37)
**Libya**	**Chronic lymphoid leukemia**	Benzene	0.41 (0.12-0.71)	0.23 (0.18-0.28)
**Libya**	**Chronic lymphoid leukemia**	Formaldehyde	0.12 (0.09-0.16)	0.06 (0.05-0.08)
**Libya**	**Other leukemia**	Benzene	0.33 (0.09-0.56)	0.07 (0.05-0.08)
**Libya**	**Other leukemia**	Formaldehyde	0.1 (0.07-0.13)	0.23 (0.16-0.31)
**Lithuania**	**All leukemia**	Benzene	0.34 (0.06-0.6)	0.26 (0.21-0.32)
**Lithuania**	**All leukemia**	Formaldehyde	0.04 (0.03-0.05)	0.39 (0.28-0.53)
**Lithuania**	**Acute myeloid leukemia**	Benzene	0.45 (0.09-0.8)	0.2 (0.12-0.31)
**Lithuania**	**Acute myeloid leukemia**	Formaldehyde	0.05 (0.04-0.07)	0.23 (0.19-0.28)
**Lithuania**	**Acute lymphoid leukemia**	Benzene	0.38 (0.07-0.67)	0.07 (0.05-0.09)
**Lithuania**	**Acute lymphoid leukemia**	Formaldehyde	0.05 (0.04-0.06)	0.05 (0.04-0.06)
**Lithuania**	**Chronic myeloid leukemia**	Benzene	0.4 (0.07-0.73)	0.27 (0.21-0.35)
**Lithuania**	**Chronic myeloid leukemia**	Formaldehyde	0.05 (0.04-0.06)	0.17 (0.11-0.27)
**Lithuania**	**Chronic lymphoid leukemia**	Benzene	0.2 (0.04-0.36)	0.2 (0.16-0.24)
**Lithuania**	**Chronic lymphoid leukemia**	Formaldehyde	0.02 (0.02-0.03)	0.18 (0.12-0.25)
**Lithuania**	**Other leukemia**	Benzene	0.25 (0.05-0.46)	0.25 (0.19-0.31)
**Lithuania**	**Other leukemia**	Formaldehyde	0.03 (0.02-0.04)	0.04 (0.03-0.05)
**Luxembourg**	**All leukemia**	Benzene	0.31 (0.06-0.55)	0.23 (0.14-0.34)
**Luxembourg**	**All leukemia**	Formaldehyde	0.04 (0.03-0.04)	0.26 (0.22-0.32)
**Luxembourg**	**Acute myeloid leukemia**	Benzene	0.39 (0.07-0.69)	0.07 (0.05-0.09)
**Luxembourg**	**Acute myeloid leukemia**	Formaldehyde	0.04 (0.04-0.05)	0.06 (0.05-0.07)
**Luxembourg**	**Acute lymphoid leukemia**	Benzene	0.53 (0.1-0.94)	0.07 (0.04-0.12)
**Luxembourg**	**Acute lymphoid leukemia**	Formaldehyde	0.06 (0.05-0.08)	0.11 (0.06-0.17)
**Luxembourg**	**Chronic myeloid leukemia**	Benzene	0.38 (0.07-0.67)	0.16 (0.1-0.23)
**Luxembourg**	**Chronic myeloid leukemia**	Formaldehyde	0.05 (0.04-0.05)	0.27 (0.2-0.36)
**Luxembourg**	**Chronic lymphoid leukemia**	Benzene	0.17 (0.03-0.3)	0.15 (0.09-0.23)
**Luxembourg**	**Chronic lymphoid leukemia**	Formaldehyde	0.02 (0.01-0.02)	0.06 (0.05-0.07)
**Luxembourg**	**Other leukemia**	Benzene	0.29 (0.05-0.51)	0.1 (0.06-0.15)
**Luxembourg**	**Other leukemia**	Formaldehyde	0.03 (0.03-0.04)	0.08 (0.05-0.13)
**Madagascar**	**All leukemia**	Benzene	0.33 (0.09-0.58)	0.29 (0.24-0.35)
**Madagascar**	**All leukemia**	Formaldehyde	0.11 (0.08-0.15)	0.32 (0.26-0.38)

Countries	Causes	Occupational carcinogens	Percent change in 1990 (95% UI)	Percent change in 2019 (95% UI)
**Madagascar**	**Acute myeloid leukemia**	Benzene	0.43 (0.12-0.81)	0.36 (0.29-0.45)
**Madagascar**	**Acute myeloid leukemia**	Formaldehyde	0.14 (0.08-0.22)	0.17 (0.1-0.25)
**Madagascar**	**Acute lymphoid leukemia**	Benzene	0.2 (0.05-0.42)	0.17 (0.12-0.24)
**Madagascar**	**Acute lymphoid leukemia**	Formaldehyde	0.07 (0.03-0.12)	0.31 (0.23-0.4)
**Madagascar**	**Chronic myeloid leukemia**	Benzene	0.61 (0.14-1.18)	0.34 (0.28-0.42)
**Madagascar**	**Chronic myeloid leukemia**	Formaldehyde	0.2 (0.1-0.32)	0.05 (0.04-0.05)
**Madagascar**	**Chronic lymphoid leukemia**	Benzene	0.28 (0.08-0.48)	0.26 (0.21-0.32)
**Madagascar**	**Chronic lymphoid leukemia**	Formaldehyde	0.09 (0.07-0.12)	0.05 (0.04-0.07)
**Madagascar**	**Other leukemia**	Benzene	0.29 (0.08-0.54)	0.06 (0.05-0.07)
**Madagascar**	**Other leukemia**	Formaldehyde	0.1 (0.06-0.14)	0.15 (0.1-0.22)
**Malawi**	**All leukemia**	Benzene	0.44 (0.12-0.82)	0.34 (0.25-0.45)
**Malawi**	**All leukemia**	Formaldehyde	0.18 (0.12-0.27)	0.16 (0.1-0.24)
**Malawi**	**Acute myeloid leukemia**	Benzene	0.72 (0.2-1.39)	0.06 (0.05-0.07)
**Malawi**	**Acute myeloid leukemia**	Formaldehyde	0.29 (0.16-0.45)	0.09 (0.05-0.14)
**Malawi**	**Acute lymphoid leukemia**	Benzene	0.37 (0.09-0.74)	0.21 (0.16-0.26)
**Malawi**	**Acute lymphoid leukemia**	Formaldehyde	0.14 (0.06-0.24)	0.25 (0.17-0.35)
**Malawi**	**Chronic myeloid leukemia**	Benzene	0.57 (0.1-1.4)	0.34 (0.26-0.42)
**Malawi**	**Chronic myeloid leukemia**	Formaldehyde	0.23 (0.07-0.45)	0.25 (0.17-0.37)
**Malawi**	**Chronic lymphoid leukemia**	Benzene	0.48 (0.14-0.79)	0.35 (0.26-0.43)
**Malawi**	**Chronic lymphoid leukemia**	Formaldehyde	0.21 (0.16-0.27)	0.14 (0.09-0.22)
**Malawi**	**Other leukemia**	Benzene	0.38 (0.1-0.74)	0.14 (0.08-0.22)
**Malawi**	**Other leukemia**	Formaldehyde	0.16 (0.09-0.27)	0.06 (0.04-0.07)
**Malaysia**	**All leukemia**	Benzene	0.74 (0.22-1.24)	0.18 (0.12-0.26)
**Malaysia**	**All leukemia**	Formaldehyde	0.33 (0.27-0.41)	0.11 (0.06-0.19)
**Malaysia**	**Acute myeloid leukemia**	Benzene	0.89 (0.26-1.5)	0.2 (0.14-0.28)
**Malaysia**	**Acute myeloid leukemia**	Formaldehyde	0.4 (0.32-0.49)	0.06 (0.05-0.07)
**Malaysia**	**Acute lymphoid leukemia**	Benzene	0.61 (0.19-1.08)	0.05 (0.04-0.06)
**Malaysia**	**Acute lymphoid leukemia**	Formaldehyde	0.27 (0.19-0.36)	0.13 (0.09-0.18)
**Malaysia**	**Chronic myeloid leukemia**	Benzene	1.07 (0.31-1.83)	0.18 (0.12-0.26)
**Malaysia**	**Chronic myeloid leukemia**	Formaldehyde	0.47 (0.32-0.6)	0.2 (0.16-0.25)
**Malaysia**	**Chronic lymphoid leukemia**	Benzene	0.3 (0.09-0.52)	0.07 (0.06-0.08)
**Malaysia**	**Chronic lymphoid leukemia**	Formaldehyde	0.14 (0.1-0.18)	0.33 (0.22-0.5)
**Malaysia**	**Other leukemia**	Benzene	0.73 (0.22-1.24)	0.03 (0.03-0.04)
**Malaysia**	**Other leukemia**	Formaldehyde	0.33 (0.26-0.42)	0.05 (0.04-0.06)
**Maldives**	**All leukemia**	Benzene	0.45 (0.13-0.77)	0.27 (0.22-0.35)
**Maldives**	**All leukemia**	Formaldehyde	0.19 (0.14-0.27)	0.24 (0.18-0.32)
**Maldives**	**Acute myeloid leukemia**	Benzene	0.54 (0.14-1.09)	0.36 (0.28-0.45)
**Maldives**	**Acute myeloid leukemia**	Formaldehyde	0.19 (0.12-0.33)	0.1 (0.07-0.14)
**Maldives**	**Acute lymphoid leukemia**	Benzene	0.35 (0.1-0.71)	0.16 (0.1-0.23)
**Maldives**	**Acute lymphoid leukemia**	Formaldehyde	0.13 (0.08-0.22)	0.28 (0.22-0.36)
**Maldives**	**Chronic myeloid leukemia**	Benzene	0.7 (0.19-1.21)	0.09 (0.05-0.13)
**Maldives**	**Chronic myeloid leukemia**	Formaldehyde	0.33 (0.24-0.45)	0.29 (0.2-0.41)
**Maldives**	**Chronic lymphoid leukemia**	Benzene	0.52 (0.15-0.87)	0.06 (0.05-0.08)
**Maldives**	**Chronic lymphoid leukemia**	Formaldehyde	0.22 (0.16-0.29)	0.07 (0.06-0.09)

Countries	Causes	Occupational carcinogens	Percent change in 1990 (95% UI)	Percent change in 2019 (95% UI)
**Maldives**	**Other leukemia**	Benzene	0.38 (0.11-0.67)	0.21 (0.16-0.28)
**Maldives**	**Other leukemia**	Formaldehyde	0.17 (0.11-0.25)	0.24 (0.2-0.3)
**Mali**	**All leukemia**	Benzene	0.29 (0.08-0.53)	0.23 (0.17-0.3)
**Mali**	**All leukemia**	Formaldehyde	0.11 (0.08-0.14)	0.18 (0.12-0.25)
**Mali**	**Acute myeloid leukemia**	Benzene	0.23 (0.06-0.51)	0.05 (0.04-0.06)
**Mali**	**Acute myeloid leukemia**	Formaldehyde	0.08 (0.04-0.14)	0.07 (0.06-0.08)
**Mali**	**Acute lymphoid leukemia**	Benzene	0.19 (0.05-0.37)	0.05 (0.04-0.06)
**Mali**	**Acute lymphoid leukemia**	Formaldehyde	0.07 (0.04-0.12)	0.23 (0.18-0.3)
**Mali**	**Chronic myeloid leukemia**	Benzene	0.55 (0.15-0.95)	0.07 (0.06-0.08)
**Mali**	**Chronic myeloid leukemia**	Formaldehyde	0.21 (0.15-0.29)	0.16 (0.1-0.23)
**Mali**	**Chronic lymphoid leukemia**	Benzene	0.18 (0.05-0.31)	0.26 (0.2-0.31)
**Mali**	**Chronic lymphoid leukemia**	Formaldehyde	0.07 (0.05-0.09)	0.29 (0.2-0.39)
**Mali**	**Other leukemia**	Benzene	0.33 (0.09-0.57)	0.23 (0.15-0.32)
**Mali**	**Other leukemia**	Formaldehyde	0.12 (0.08-0.15)	0.3 (0.23-0.38)
**Malta**	**All leukemia**	Benzene	0.29 (0.06-0.52)	0.21 (0.17-0.27)
**Malta**	**All leukemia**	Formaldehyde	0.04 (0.03-0.05)	0.28 (0.2-0.39)
**Malta**	**Acute myeloid leukemia**	Benzene	0.32 (0.06-0.57)	0.05 (0.04-0.06)
**Malta**	**Acute myeloid leukemia**	Formaldehyde	0.04 (0.04-0.05)	0.24 (0.17-0.32)
**Malta**	**Acute lymphoid leukemia**	Benzene	0.43 (0.08-0.77)	0.19 (0.12-0.27)
**Malta**	**Acute lymphoid leukemia**	Formaldehyde	0.06 (0.05-0.07)	0.19 (0.12-0.28)
**Malta**	**Chronic myeloid leukemia**	Benzene	0.29 (0.06-0.53)	0.21 (0.16-0.27)
**Malta**	**Chronic myeloid leukemia**	Formaldehyde	0.04 (0.03-0.05)	0.05 (0.04-0.06)
**Malta**	**Chronic lymphoid leukemia**	Benzene	0.16 (0.03-0.28)	0.05 (0.04-0.06)
**Malta**	**Chronic lymphoid leukemia**	Formaldehyde	0.02 (0.02-0.03)	0.13 (0.08-0.19)
**Malta**	**Other leukemia**	Benzene	0.22 (0.04-0.4)	0.23 (0.14-0.34)
**Malta**	**Other leukemia**	Formaldehyde	0.03 (0.02-0.04)	0.41 (0.31-0.53)
**Marshall Islands**	**All leukemia**	Benzene	0.71 (0.2-1.2)	0.36 (0.24-0.53)
**Marshall Islands**	**All leukemia**	Formaldehyde	0.17 (0.13-0.22)	0.11 (0.07-0.15)
**Marshall Islands**	**Acute myeloid leukemia**	Benzene	0.85 (0.24-1.45)	0.05 (0.04-0.07)
**Marshall Islands**	**Acute myeloid leukemia**	Formaldehyde	0.21 (0.15-0.27)	0.2 (0.14-0.26)
**Marshall Islands**	**Acute lymphoid leukemia**	Benzene	0.64 (0.19-1.12)	0.1 (0.07-0.15)
**Marshall Islands**	**Acute lymphoid leukemia**	Formaldehyde	0.15 (0.11-0.22)	0.37 (0.29-0.48)
**Marshall Islands**	**Chronic myeloid leukemia**	Benzene	0.84 (0.24-1.48)	0.38 (0.31-0.46)
**Marshall Islands**	**Chronic myeloid leukemia**	Formaldehyde	0.21 (0.15-0.29)	0.31 (0.21-0.43)
**Marshall Islands**	**Chronic lymphoid leukemia**	Benzene	0.65 (0.19-1.1)	0.05 (0.04-0.07)
**Marshall Islands**	**Chronic lymphoid leukemia**	Formaldehyde	0.16 (0.12-0.21)	0.21 (0.14-0.3)
**Marshall Islands**	**Other leukemia**	Benzene	0.63 (0.18-1.07)	0.05 (0.04-0.06)
**Marshall Islands**	**Other leukemia**	Formaldehyde	0.15 (0.12-0.2)	0.19 (0.15-0.25)
**Mauritania**	**All leukemia**	Benzene	0.31 (0.09-0.52)	0.07 (0.03-0.13)
**Mauritania**	**All leukemia**	Formaldehyde	0.11 (0.09-0.13)	0.19 (0.14-0.26)
**Mauritania**	**Acute myeloid leukemia**	Benzene	0.3 (0.09-0.54)	0.14 (0.1-0.21)
**Mauritania**	**Acute myeloid leukemia**	Formaldehyde	0.11 (0.07-0.14)	0.29 (0.2-0.4)
**Mauritania**	**Acute lymphoid leukemia**	Benzene	0.24 (0.07-0.43)	0.21 (0.16-0.28)
**Mauritania**	**Acute lymphoid leukemia**	Formaldehyde	0.08 (0.05-0.12)	0.05 (0.04-0.07)

Countries	Causes	Occupational carcinogens	Percent change in 1990 (95% UI)	Percent change in 2019 (95% UI)
**Mauritania**	**Chronic myeloid leukemia**	Benzene	0.64 (0.19-1.08)	0.06 (0.05-0.07)
**Mauritania**	**Chronic myeloid leukemia**	Formaldehyde	0.22 (0.16-0.29)	0.29 (0.22-0.35)
**Mauritania**	**Chronic lymphoid leukemia**	Benzene	0.14 (0.04-0.24)	0.08 (0.04-0.13)
**Mauritania**	**Chronic lymphoid leukemia**	Formaldehyde	0.05 (0.03-0.06)	0.12 (0.07-0.17)
**Mauritania**	**Other leukemia**	Benzene	0.28 (0.08-0.47)	0.21 (0.15-0.28)
**Mauritania**	**Other leukemia**	Formaldehyde	0.1 (0.08-0.12)	0.06 (0.04-0.07)
**Mauritius**	**All leukemia**	Benzene	0.62 (0.18-1.02)	0.37 (0.28-0.48)
**Mauritius**	**All leukemia**	Formaldehyde	0.31 (0.26-0.38)	0.06 (0.05-0.07)
**Mauritius**	**Acute myeloid leukemia**	Benzene	0.72 (0.21-1.19)	0.29 (0.2-0.4)
**Mauritius**	**Acute myeloid leukemia**	Formaldehyde	0.37 (0.3-0.46)	0.13 (0.09-0.19)
**Mauritius**	**Acute lymphoid leukemia**	Benzene	0.59 (0.17-1)	0.3 (0.23-0.39)
**Mauritius**	**Acute lymphoid leukemia**	Formaldehyde	0.29 (0.21-0.39)	0.2 (0.13-0.28)
**Mauritius**	**Chronic myeloid leukemia**	Benzene	0.72 (0.21-1.19)	0.29 (0.23-0.35)
**Mauritius**	**Chronic myeloid leukemia**	Formaldehyde	0.36 (0.28-0.44)	0.1 (0.07-0.15)
**Mauritius**	**Chronic lymphoid leukemia**	Benzene	0.44 (0.13-0.73)	0.33 (0.26-0.41)
**Mauritius**	**Chronic lymphoid leukemia**	Formaldehyde	0.22 (0.18-0.27)	0.34 (0.26-0.43)
**Mauritius**	**Other leukemia**	Benzene	0.6 (0.18-0.99)	0.23 (0.18-0.29)
**Mauritius**	**Other leukemia**	Formaldehyde	0.3 (0.25-0.37)	0.07 (0.05-0.08)
**Mexico**	**All leukemia**	Benzene	0.72 (0.21-1.18)	0.06 (0.05-0.07)
**Mexico**	**All leukemia**	Formaldehyde	0.3 (0.25-0.36)	0.04 (0.03-0.05)
**Mexico**	**Acute myeloid leukemia**	Benzene	0.9 (0.27-1.49)	0.5 (0.35-0.7)
**Mexico**	**Acute myeloid leukemia**	Formaldehyde	0.37 (0.31-0.45)	0.09 (0.07-0.11)
**Mexico**	**Acute lymphoid leukemia**	Benzene	0.55 (0.16-0.91)	0.03 (0.02-0.04)
**Mexico**	**Acute lymphoid leukemia**	Formaldehyde	0.23 (0.18-0.29)	0.05 (0.04-0.07)
**Mexico**	**Chronic myeloid leukemia**	Benzene	1.12 (0.33-1.83)	0.06 (0.05-0.07)
**Mexico**	**Chronic myeloid leukemia**	Formaldehyde	0.47 (0.39-0.57)	0.1 (0.05-0.16)
**Mexico**	**Chronic lymphoid leukemia**	Benzene	0.55 (0.16-0.9)	0.21 (0.13-0.28)
**Mexico**	**Chronic lymphoid leukemia**	Formaldehyde	0.23 (0.19-0.27)	0.25 (0.2-0.3)
**Mexico**	**Other leukemia**	Benzene	0.74 (0.22-1.22)	0.22 (0.17-0.27)
**Mexico**	**Other leukemia**	Formaldehyde	0.31 (0.25-0.37)	0.18 (0.13-0.24)
**Micronesia (Federated States of)**	**All leukemia**	Benzene	0.72 (0.21-1.21)	0.04 (0.03-0.05)
**Micronesia (Federated States of)**	**All leukemia**	Formaldehyde	0.26 (0.19-0.33)	0.15 (0.1-0.22)
**Micronesia (Federated States of)**	**Acute myeloid leukemia**	Benzene	0.88 (0.26-1.49)	0.37 (0.27-0.54)
**Micronesia (Federated States of)**	**Acute myeloid leukemia**	Formaldehyde	0.32 (0.23-0.42)	0.13 (0.09-0.2)
**Micronesia (Federated States of)**	**Acute lymphoid leukemia**	Benzene	0.59 (0.17-1.04)	0.06 (0.05-0.07)
**Micronesia (Federated States of)**	**Acute lymphoid leukemia**	Formaldehyde	0.21 (0.14-0.29)	0.36 (0.27-0.48)
**Micronesia (Federated States of)**	**Chronic myeloid leukemia**	Benzene	0.86 (0.26-1.55)	0.08 (0.05-0.14)
**Micronesia (Federated States of)**	**Chronic myeloid leukemia**	Formaldehyde	0.32 (0.2-0.46)	0.09 (0.07-0.11)
**Micronesia (Federated States of)**	**Chronic lymphoid leukemia**	Benzene	0.58 (0.17-1.02)	0.07 (0.05-0.08)
**Micronesia (Federated States of)**	**Chronic lymphoid leukemia**	Formaldehyde	0.22 (0.16-0.29)	0.05 (0.04-0.07)
**Micronesia (Federated States of)**	**Other leukemia**	Benzene	0.64 (0.18-1.1)	0.36 (0.21-0.67)
**Micronesia (Federated States of)**	**Other leukemia**	Formaldehyde	0.23 (0.17-0.3)	0.08 (0.04-0.14)
**Monaco**	**All leukemia**	Benzene	0.27 (0.05-0.5)	0.27 (0.18-0.38)
**Monaco**	**All leukemia**	Formaldehyde	0.03 (0.03-0.04)	0.07 (0.03-0.12)

Countries	Causes	Occupational carcinogens	Percent change in 1990 (95% UI)	Percent change in 2019 (95% UI)
**Monaco**	**Acute myeloid leukemia**	Benzene	0.34 (0.06-0.62)	0.04 (0.04-0.05)
**Monaco**	**Acute myeloid leukemia**	Formaldehyde	0.04 (0.03-0.05)	0.32 (0.24-0.41)
**Monaco**	**Acute lymphoid leukemia**	Benzene	0.52 (0.09-0.96)	0.19 (0.13-0.26)
**Monaco**	**Acute lymphoid leukemia**	Formaldehyde	0.06 (0.05-0.08)	0.21 (0.17-0.26)
**Monaco**	**Chronic myeloid leukemia**	Benzene	0.35 (0.06-0.65)	0.06 (0.05-0.07)
**Monaco**	**Chronic myeloid leukemia**	Formaldehyde	0.04 (0.03-0.05)	0.07 (0.05-0.08)
**Monaco**	**Chronic lymphoid leukemia**	Benzene	0.15 (0.03-0.27)	0.16 (0.11-0.22)
**Monaco**	**Chronic lymphoid leukemia**	Formaldehyde	0.02 (0.01-0.03)	0.41 (0.34-0.5)
**Monaco**	**Other leukemia**	Benzene	0.23 (0.04-0.42)	0.14 (0.1-0.19)
**Monaco**	**Other leukemia**	Formaldehyde	0.03 (0.02-0.03)	0.33 (0.25-0.43)
**Mongolia**	**All leukemia**	Benzene	0.53 (0.15-0.89)	0.16 (0.1-0.23)
**Mongolia**	**All leukemia**	Formaldehyde	0.17 (0.13-0.21)	0.19 (0.13-0.27)
**Mongolia**	**Acute myeloid leukemia**	Benzene	0.75 (0.21-1.24)	0.26 (0.19-0.35)
**Mongolia**	**Acute myeloid leukemia**	Formaldehyde	0.24 (0.17-0.31)	0.19 (0.14-0.26)
**Mongolia**	**Acute lymphoid leukemia**	Benzene	0.31 (0.09-0.55)	0.27 (0.22-0.34)
**Mongolia**	**Acute lymphoid leukemia**	Formaldehyde	0.1 (0.06-0.14)	0.22 (0.17-0.28)
**Mongolia**	**Chronic myeloid leukemia**	Benzene	0.51 (0.14-0.92)	0.31 (0.22-0.4)
**Mongolia**	**Chronic myeloid leukemia**	Formaldehyde	0.17 (0.11-0.23)	0.18 (0.14-0.23)
**Mongolia**	**Chronic lymphoid leukemia**	Benzene	0.66 (0.18-1.15)	0.31 (0.22-0.41)
**Mongolia**	**Chronic lymphoid leukemia**	Formaldehyde	0.21 (0.15-0.29)	0.09 (0.04-0.14)
**Mongolia**	**Other leukemia**	Benzene	0.34 (0.09-0.66)	0.05 (0.04-0.06)
**Mongolia**	**Other leukemia**	Formaldehyde	0.11 (0.08-0.17)	0.59 (0.42-0.8)
**Montenegro**	**All leukemia**	Benzene	0.3 (0.06-0.53)	0.07 (0.06-0.08)
**Montenegro**	**All leukemia**	Formaldehyde	0.03 (0.03-0.04)	0.09 (0.05-0.15)
**Montenegro**	**Acute myeloid leukemia**	Benzene	0.37 (0.07-0.66)	0.07 (0.06-0.08)
**Montenegro**	**Acute myeloid leukemia**	Formaldehyde	0.04 (0.03-0.05)	0.21 (0.15-0.28)
**Montenegro**	**Acute lymphoid leukemia**	Benzene	0.33 (0.07-0.61)	0.3 (0.25-0.37)
**Montenegro**	**Acute lymphoid leukemia**	Formaldehyde	0.04 (0.03-0.05)	0.24 (0.19-0.3)
**Montenegro**	**Chronic myeloid leukemia**	Benzene	0.34 (0.06-0.61)	0.25 (0.18-0.34)
**Montenegro**	**Chronic myeloid leukemia**	Formaldehyde	0.04 (0.03-0.05)	0.27 (0.22-0.34)
**Montenegro**	**Chronic lymphoid leukemia**	Benzene	0.15 (0.03-0.27)	0.36 (0.27-0.45)
**Montenegro**	**Chronic lymphoid leukemia**	Formaldehyde	0.02 (0.01-0.02)	0.14 (0.09-0.2)
**Montenegro**	**Other leukemia**	Benzene	0.2 (0.04-0.38)	0.13 (0.06-0.2)
**Montenegro**	**Other leukemia**	Formaldehyde	0.02 (0.02-0.03)	0.23 (0.16-0.34)
**Morocco**	**All leukemia**	Benzene	0.52 (0.15-0.87)	0.22 (0.15-0.32)
**Morocco**	**All leukemia**	Formaldehyde	0.22 (0.17-0.27)	0.04 (0.03-0.05)
**Morocco**	**Acute myeloid leukemia**	Benzene	0.65 (0.18-1.09)	0.18 (0.14-0.23)
**Morocco**	**Acute myeloid leukemia**	Formaldehyde	0.27 (0.21-0.34)	0.36 (0.28-0.45)
**Morocco**	**Acute lymphoid leukemia**	Benzene	0.47 (0.14-0.83)	0.05 (0.04-0.06)
**Morocco**	**Acute lymphoid leukemia**	Formaldehyde	0.2 (0.14-0.27)	0.3 (0.21-0.41)
**Morocco**	**Chronic myeloid leukemia**	Benzene	0.64 (0.19-1.09)	0.29 (0.23-0.34)
**Morocco**	**Chronic myeloid leukemia**	Formaldehyde	0.27 (0.21-0.36)	0.3 (0.25-0.35)
**Morocco**	**Chronic lymphoid leukemia**	Benzene	0.52 (0.15-0.88)	0.22 (0.17-0.27)
**Morocco**	**Chronic lymphoid leukemia**	Formaldehyde	0.22 (0.16-0.28)	0.04 (0.03-0.05)

Countries	Causes	Occupational carcinogens	Percent change in 1990 (95% UI)	Percent change in 2019 (95% UI)
**Morocco**	**Other leukemia**	Benzene	0.42 (0.12-0.72)	0.04 (0.03-0.05)
**Morocco**	**Other leukemia**	Formaldehyde	0.18 (0.14-0.23)	0.3 (0.23-0.38)
**Mozambique**	**All leukemia**	Benzene	0.18 (0.05-0.33)	0.35 (0.29-0.42)
**Mozambique**	**All leukemia**	Formaldehyde	0.06 (0.04-0.09)	0.39 (0.29-0.53)
**Mozambique**	**Acute myeloid leukemia**	Benzene	0.29 (0.07-0.67)	0.25 (0.19-0.32)
**Mozambique**	**Acute myeloid leukemia**	Formaldehyde	0.1 (0.05-0.19)	0.27 (0.22-0.32)
**Mozambique**	**Acute lymphoid leukemia**	Benzene	0.12 (0.03-0.32)	0.06 (0.05-0.07)
**Mozambique**	**Acute lymphoid leukemia**	Formaldehyde	0.04 (0.02-0.1)	0.03 (0.03-0.04)
**Mozambique**	**Chronic myeloid leukemia**	Benzene	0.33 (0.07-0.72)	0.43 (0.35-0.55)
**Mozambique**	**Chronic myeloid leukemia**	Formaldehyde	0.11 (0.05-0.19)	0.2 (0.13-0.29)
**Mozambique**	**Chronic lymphoid leukemia**	Benzene	0.17 (0.05-0.29)	0.21 (0.18-0.25)
**Mozambique**	**Chronic lymphoid leukemia**	Formaldehyde	0.06 (0.04-0.08)	0.23 (0.17-0.29)
**Mozambique**	**Other leukemia**	Benzene	0.16 (0.04-0.33)	0.34 (0.27-0.43)
**Mozambique**	**Other leukemia**	Formaldehyde	0.05 (0.03-0.1)	0.04 (0.03-0.04)
**Myanmar**	**All leukemia**	Benzene	0.26 (0.07-0.46)	0.31 (0.22-0.42)
**Myanmar**	**All leukemia**	Formaldehyde	0.11 (0.08-0.14)	0.28 (0.24-0.34)
**Myanmar**	**Acute myeloid leukemia**	Benzene	0.33 (0.09-0.56)	0.09 (0.07-0.11)
**Myanmar**	**Acute myeloid leukemia**	Formaldehyde	0.13 (0.09-0.18)	0.06 (0.05-0.07)
**Myanmar**	**Acute lymphoid leukemia**	Benzene	0.17 (0.05-0.34)	0.09 (0.05-0.14)
**Myanmar**	**Acute lymphoid leukemia**	Formaldehyde	0.07 (0.04-0.11)	0.24 (0.14-0.33)
**Myanmar**	**Chronic myeloid leukemia**	Benzene	0.35 (0.1-0.62)	0.17 (0.13-0.24)
**Myanmar**	**Chronic myeloid leukemia**	Formaldehyde	0.14 (0.1-0.19)	0.38 (0.29-0.48)
**Myanmar**	**Chronic lymphoid leukemia**	Benzene	0.23 (0.07-0.39)	0.22 (0.16-0.29)
**Myanmar**	**Chronic lymphoid leukemia**	Formaldehyde	0.09 (0.07-0.12)	0.04 (0.03-0.05)
**Myanmar**	**Other leukemia**	Benzene	0.27 (0.07-0.47)	0.21 (0.15-0.28)
**Myanmar**	**Other leukemia**	Formaldehyde	0.11 (0.07-0.14)	0.1 (0.06-0.16)
**Namibia**	**All leukemia**	Benzene	0.45 (0.13-0.76)	0.26 (0.22-0.31)
**Namibia**	**All leukemia**	Formaldehyde	0.11 (0.09-0.14)	0.34 (0.28-0.41)
**Namibia**	**Acute myeloid leukemia**	Benzene	0.71 (0.21-1.24)	0.36 (0.3-0.44)
**Namibia**	**Acute myeloid leukemia**	Formaldehyde	0.18 (0.13-0.24)	0.28 (0.19-0.36)
**Namibia**	**Acute lymphoid leukemia**	Benzene	0.42 (0.12-0.8)	0.28 (0.21-0.36)
**Namibia**	**Acute lymphoid leukemia**	Formaldehyde	0.11 (0.06-0.16)	0.28 (0.21-0.36)
**Namibia**	**Chronic myeloid leukemia**	Benzene	0.57 (0.15-1.02)	0.29 (0.24-0.36)
**Namibia**	**Chronic myeloid leukemia**	Formaldehyde	0.14 (0.1-0.2)	0.03 (0.03-0.04)
**Namibia**	**Chronic lymphoid leukemia**	Benzene	0.29 (0.08-0.51)	0.31 (0.25-0.37)
**Namibia**	**Chronic lymphoid leukemia**	Formaldehyde	0.08 (0.06-0.1)	0.03 (0.02-0.05)
**Namibia**	**Other leukemia**	Benzene	0.47 (0.13-0.79)	0.05 (0.04-0.06)
**Namibia**	**Other leukemia**	Formaldehyde	0.12 (0.09-0.15)	0.19 (0.13-0.26)
**Nauru**	**All leukemia**	Benzene	0.92 (0.28-1.54)	0.35 (0.27-0.46)
**Nauru**	**All leukemia**	Formaldehyde	0.32 (0.24-0.41)	0.29 (0.21-0.38)
**Nauru**	**Acute myeloid leukemia**	Benzene	1.07 (0.3-1.81)	0.04 (0.03-0.05)
**Nauru**	**Acute myeloid leukemia**	Formaldehyde	0.37 (0.27-0.48)	0.18 (0.12-0.25)
**Nauru**	**Acute lymphoid leukemia**	Benzene	0.66 (0.2-1.15)	0.26 (0.2-0.33)
**Nauru**	**Acute lymphoid leukemia**	Formaldehyde	0.23 (0.15-0.33)	0.39 (0.3-0.51)

Countries	Causes	Occupational carcinogens	Percent change in 1990 (95% UI)	Percent change in 2019 (95% UI)
**Nauru**	**Chronic myeloid leukemia**	Benzene	1.21 (0.36-2.06)	0.37 (0.3-0.45)
**Nauru**	**Chronic myeloid leukemia**	Formaldehyde	0.44 (0.31-0.6)	0.28 (0.21-0.38)
**Nauru**	**Chronic lymphoid leukemia**	Benzene	0.83 (0.23-1.41)	0.38 (0.3-0.47)
**Nauru**	**Chronic lymphoid leukemia**	Formaldehyde	0.3 (0.22-0.39)	0.24 (0.17-0.33)
**Nauru**	**Other leukemia**	Benzene	0.82 (0.24-1.43)	0.26 (0.17-0.36)
**Nauru**	**Other leukemia**	Formaldehyde	0.28 (0.21-0.37)	0.04 (0.03-0.05)
**Nepal**	**All leukemia**	Benzene	0.47 (0.14-0.82)	0.28 (0.2-0.38)
**Nepal**	**All leukemia**	Formaldehyde	0.18 (0.13-0.24)	0.24 (0.16-0.35)
**Nepal**	**Acute myeloid leukemia**	Benzene	0.55 (0.16-0.94)	0.26 (0.21-0.33)
**Nepal**	**Acute myeloid leukemia**	Formaldehyde	0.21 (0.14-0.29)	0.04 (0.03-0.04)
**Nepal**	**Acute lymphoid leukemia**	Benzene	0.27 (0.08-0.5)	0.03 (0.02-0.04)
**Nepal**	**Acute lymphoid leukemia**	Formaldehyde	0.1 (0.06-0.15)	0.17 (0.13-0.23)
**Nepal**	**Chronic myeloid leukemia**	Benzene	0.69 (0.19-1.2)	0.22 (0.14-0.31)
**Nepal**	**Chronic myeloid leukemia**	Formaldehyde	0.27 (0.17-0.37)	0.2 (0.16-0.25)
**Nepal**	**Chronic lymphoid leukemia**	Benzene	0.33 (0.1-0.56)	0.04 (0.03-0.05)
**Nepal**	**Chronic lymphoid leukemia**	Formaldehyde	0.13 (0.1-0.17)	0.29 (0.21-0.37)
**Nepal**	**Other leukemia**	Benzene	0.39 (0.11-0.76)	0.02 (0.02-0.03)
**Nepal**	**Other leukemia**	Formaldehyde	0.15 (0.09-0.23)	0.04 (0.03-0.05)
**Netherlands**	**All leukemia**	Benzene	0.34 (0.06-0.61)	0.36 (0.29-0.43)
**Netherlands**	**All leukemia**	Formaldehyde	0.04 (0.03-0.04)	0.27 (0.21-0.34)
**Netherlands**	**Acute myeloid leukemia**	Benzene	0.44 (0.08-0.8)	0.42 (0.35-0.52)
**Netherlands**	**Acute myeloid leukemia**	Formaldehyde	0.05 (0.04-0.05)	0.12 (0.09-0.17)
**Netherlands**	**Acute lymphoid leukemia**	Benzene	0.54 (0.11-0.98)	0.19 (0.14-0.26)
**Netherlands**	**Acute lymphoid leukemia**	Formaldehyde	0.06 (0.05-0.07)	0.37 (0.29-0.46)
**Netherlands**	**Chronic myeloid leukemia**	Benzene	0.41 (0.07-0.73)	0.23 (0.16-0.31)
**Netherlands**	**Chronic myeloid leukemia**	Formaldehyde	0.04 (0.04-0.05)	0.37 (0.27-0.47)
**Netherlands**	**Chronic lymphoid leukemia**	Benzene	0.16 (0.03-0.29)	0.05 (0.04-0.06)
**Netherlands**	**Chronic lymphoid leukemia**	Formaldehyde	0.02 (0.01-0.02)	0.06 (0.05-0.07)
**Netherlands**	**Other leukemia**	Benzene	0.25 (0.05-0.46)	0.23 (0.19-0.28)
**Netherlands**	**Other leukemia**	Formaldehyde	0.03 (0.02-0.03)	0.34 (0.28-0.41)
**New Zealand**	**All leukemia**	Benzene	0.37 (0.07-0.67)	0.25 (0.18-0.32)
**New Zealand**	**All leukemia**	Formaldehyde	0.04 (0.04-0.05)	0.22 (0.16-0.29)
**New Zealand**	**Acute myeloid leukemia**	Benzene	0.42 (0.08-0.74)	0.04 (0.03-0.04)
**New Zealand**	**Acute myeloid leukemia**	Formaldehyde	0.05 (0.04-0.06)	0.05 (0.04-0.06)
**New Zealand**	**Acute lymphoid leukemia**	Benzene	0.51 (0.1-0.92)	0.03 (0.02-0.04)
**New Zealand**	**Acute lymphoid leukemia**	Formaldehyde	0.06 (0.05-0.08)	0.3 (0.24-0.36)
**New Zealand**	**Chronic myeloid leukemia**	Benzene	0.49 (0.1-0.87)	0.04 (0.03-0.05)
**New Zealand**	**Chronic myeloid leukemia**	Formaldehyde	0.06 (0.05-0.07)	0.18 (0.13-0.24)
**New Zealand**	**Chronic lymphoid leukemia**	Benzene	0.19 (0.04-0.34)	0.3 (0.25-0.36)
**New Zealand**	**Chronic lymphoid leukemia**	Formaldehyde	0.02 (0.02-0.03)	0.43 (0.33-0.54)
**New Zealand**	**Other leukemia**	Benzene	0.26 (0.05-0.47)	0.28 (0.2-0.37)
**New Zealand**	**Other leukemia**	Formaldehyde	0.03 (0.03-0.04)	0.37 (0.3-0.45)
**Nicaragua**	**All leukemia**	Benzene	0.42 (0.12-0.71)	0.3 (0.24-0.37)
**Nicaragua**	**All leukemia**	Formaldehyde	0.16 (0.12-0.2)	0.42 (0.31-0.55)

Countries	Causes	Occupational carcinogens	Percent change in 1990 (95% UI)	Percent change in 2019 (95% UI)
**Nicaragua**	**Acute myeloid leukemia**	Benzene	0.41 (0.12-0.74)	0.04 (0.03-0.05)
**Nicaragua**	**Acute myeloid leukemia**	Formaldehyde	0.15 (0.11-0.21)	0.22 (0.17-0.29)
**Nicaragua**	**Acute lymphoid leukemia**	Benzene	0.29 (0.08-0.5)	0.3 (0.21-0.41)
**Nicaragua**	**Acute lymphoid leukemia**	Formaldehyde	0.11 (0.08-0.15)	0.24 (0.17-0.32)
**Nicaragua**	**Chronic myeloid leukemia**	Benzene	0.74 (0.21-1.26)	0.21 (0.17-0.27)
**Nicaragua**	**Chronic myeloid leukemia**	Formaldehyde	0.27 (0.21-0.34)	0.04 (0.03-0.05)
**Nicaragua**	**Chronic lymphoid leukemia**	Benzene	0.66 (0.19-1.13)	0.03 (0.03-0.04)
**Nicaragua**	**Chronic lymphoid leukemia**	Formaldehyde	0.24 (0.19-0.3)	0.25 (0.17-0.33)
**Nicaragua**	**Other leukemia**	Benzene	0.5 (0.15-0.87)	0.38 (0.27-0.51)
**Nicaragua**	**Other leukemia**	Formaldehyde	0.19 (0.13-0.24)	0.39 (0.32-0.47)
**Niger**	**All leukemia**	Benzene	0.28 (0.07-0.57)	0.36 (0.26-0.53)
**Niger**	**All leukemia**	Formaldehyde	0.09 (0.05-0.15)	0.12 (0.07-0.19)
**Niger**	**Acute myeloid leukemia**	Benzene	0.26 (0.05-0.59)	0.04 (0.03-0.05)
**Niger**	**Acute myeloid leukemia**	Formaldehyde	0.09 (0.03-0.16)	0.24 (0.18-0.31)
**Niger**	**Acute lymphoid leukemia**	Benzene	0.18 (0.04-0.4)	0.13 (0.1-0.17)
**Niger**	**Acute lymphoid leukemia**	Formaldehyde	0.06 (0.03-0.12)	0.34 (0.28-0.41)
**Niger**	**Chronic myeloid leukemia**	Benzene	0.6 (0.17-1.17)	0.42 (0.35-0.5)
**Niger**	**Chronic myeloid leukemia**	Formaldehyde	0.2 (0.11-0.34)	0.37 (0.25-0.48)
**Niger**	**Chronic lymphoid leukemia**	Benzene	0.18 (0.05-0.31)	0.03 (0.02-0.04)
**Niger**	**Chronic lymphoid leukemia**	Formaldehyde	0.06 (0.05-0.08)	0.34 (0.27-0.41)
**Niger**	**Other leukemia**	Benzene	0.3 (0.08-0.6)	0.04 (0.03-0.05)
**Niger**	**Other leukemia**	Formaldehyde	0.1 (0.06-0.16)	0.23 (0.18-0.28)
**Nigeria**	**All leukemia**	Benzene	0.47 (0.13-0.85)	0.16 (0.07-0.25)
**Nigeria**	**All leukemia**	Formaldehyde	0.13 (0.1-0.18)	0.27 (0.21-0.34)
**Nigeria**	**Acute myeloid leukemia**	Benzene	0.44 (0.11-0.91)	0.2 (0.15-0.27)
**Nigeria**	**Acute myeloid leukemia**	Formaldehyde	0.12 (0.06-0.21)	0.42 (0.32-0.54)
**Nigeria**	**Acute lymphoid leukemia**	Benzene	0.38 (0.1-0.75)	0.28 (0.22-0.35)
**Nigeria**	**Acute lymphoid leukemia**	Formaldehyde	0.11 (0.06-0.17)	0.04 (0.03-0.05)
**Nigeria**	**Chronic myeloid leukemia**	Benzene	0.87 (0.25-1.5)	0.04 (0.04-0.05)
**Nigeria**	**Chronic myeloid leukemia**	Formaldehyde	0.25 (0.17-0.34)	0.34 (0.27-0.41)
**Nigeria**	**Chronic lymphoid leukemia**	Benzene	0.25 (0.07-0.42)	0.11 (0.06-0.18)
**Nigeria**	**Chronic lymphoid leukemia**	Formaldehyde	0.07 (0.06-0.09)	0.14 (0.09-0.19)
**Nigeria**	**Other leukemia**	Benzene	0.49 (0.14-0.87)	0.23 (0.17-0.3)
**Nigeria**	**Other leukemia**	Formaldehyde	0.14 (0.1-0.18)	0.05 (0.04-0.06)
**Niue**	**All leukemia**	Benzene	0.55 (0.16-0.94)	0.35 (0.28-0.45)
**Niue**	**All leukemia**	Formaldehyde	0.19 (0.14-0.24)	0.04 (0.03-0.05)
**Niue**	**Acute myeloid leukemia**	Benzene	0.74 (0.22-1.27)	0.33 (0.24-0.44)
**Niue**	**Acute myeloid leukemia**	Formaldehyde	0.25 (0.19-0.32)	0.25 (0.19-0.32)
**Niue**	**Acute lymphoid leukemia**	Benzene	0.64 (0.18-1.12)	0.33 (0.27-0.41)
**Niue**	**Acute lymphoid leukemia**	Formaldehyde	0.21 (0.15-0.29)	0.25 (0.17-0.35)
**Niue**	**Chronic myeloid leukemia**	Benzene	0.64 (0.18-1.1)	0.28 (0.23-0.34)
**Niue**	**Chronic myeloid leukemia**	Formaldehyde	0.22 (0.16-0.3)	0.19 (0.14-0.25)
**Niue**	**Chronic lymphoid leukemia**	Benzene	0.42 (0.12-0.72)	0.37 (0.31-0.45)
**Niue**	**Chronic lymphoid leukemia**	Formaldehyde	0.15 (0.11-0.19)	0.38 (0.31-0.45)

Countries	Causes	Occupational carcinogens	Percent change in 1990 (95% UI)	Percent change in 2019 (95% UI)
**Niue**	**Other leukemia**	Benzene	0.43 (0.13-0.75)	0.35 (0.29-0.42)
**Niue**	**Other leukemia**	Formaldehyde	0.14 (0.11-0.18)	0.05 (0.04-0.06)
**North Macedonia**	**All leukemia**	Benzene	0.26 (0.05-0.48)	0.04 (0.03-0.05)
**North Macedonia**	**All leukemia**	Formaldehyde	0.04 (0.03-0.05)	0.03 (0.03-0.04)
**North Macedonia**	**Acute myeloid leukemia**	Benzene	0.29 (0.05-0.53)	0.53 (0.4-0.71)
**North Macedonia**	**Acute myeloid leukemia**	Formaldehyde	0.04 (0.03-0.05)	0.07 (0.06-0.08)
**North Macedonia**	**Acute lymphoid leukemia**	Benzene	0.24 (0.04-0.44)	0.03 (0.03-0.04)
**North Macedonia**	**Acute lymphoid leukemia**	Formaldehyde	0.03 (0.02-0.05)	0.05 (0.04-0.06)
**North Macedonia**	**Chronic myeloid leukemia**	Benzene	0.3 (0.06-0.56)	0.06 (0.05-0.07)
**North Macedonia**	**Chronic myeloid leukemia**	Formaldehyde	0.05 (0.03-0.06)	0.2 (0.12-0.28)
**North Macedonia**	**Chronic lymphoid leukemia**	Benzene	0.14 (0.03-0.25)	0.32 (0.21-0.41)
**North Macedonia**	**Chronic lymphoid leukemia**	Formaldehyde	0.02 (0.02-0.03)	0.32 (0.26-0.38)
**North Macedonia**	**Other leukemia**	Benzene	0.28 (0.05-0.51)	0.3 (0.25-0.36)
**North Macedonia**	**Other leukemia**	Formaldehyde	0.04 (0.03-0.05)	0.2 (0.15-0.27)
**Northern Mariana Islands**	**All leukemia**	Benzene	1.37 (0.4-2.26)	0.03 (0.02-0.04)
**Northern Mariana Islands**	**All leukemia**	Formaldehyde	0.45 (0.35-0.57)	0.16 (0.12-0.22)
**Northern Mariana Islands**	**Acute myeloid leukemia**	Benzene	1.5 (0.43-2.47)	0.36 (0.27-0.49)
**Northern Mariana Islands**	**Acute myeloid leukemia**	Formaldehyde	0.48 (0.37-0.62)	0.17 (0.12-0.22)
**Northern Mariana Islands**	**Acute lymphoid leukemia**	Benzene	1.35 (0.38-2.31)	0.05 (0.04-0.07)
**Northern Mariana Islands**	**Acute lymphoid leukemia**	Formaldehyde	0.48 (0.31-0.71)	0.36 (0.28-0.46)
**Northern Mariana Islands**	**Chronic myeloid leukemia**	Benzene	1.29 (0.4-2.21)	0.11 (0.07-0.16)
**Northern Mariana Islands**	**Chronic myeloid leukemia**	Formaldehyde	0.46 (0.32-0.62)	0.06 (0.05-0.07)
**Northern Mariana Islands**	**Chronic lymphoid leukemia**	Benzene	1.09 (0.32-1.83)	0.06 (0.05-0.07)
**Northern Mariana Islands**	**Chronic lymphoid leukemia**	Formaldehyde	0.37 (0.28-0.49)	0.04 (0.03-0.05)
**Northern Mariana Islands**	**Other leukemia**	Benzene	1.21 (0.34-2.06)	0.52 (0.36-0.75)
**Northern Mariana Islands**	**Other leukemia**	Formaldehyde	0.38 (0.28-0.5)	0.19 (0.11-0.28)
**Norway**	**All leukemia**	Benzene	0.42 (0.08-0.74)	0.33 (0.25-0.43)
**Norway**	**All leukemia**	Formaldehyde	0.04 (0.04-0.05)	0.15 (0.09-0.24)
**Norway**	**Acute myeloid leukemia**	Benzene	0.51 (0.1-0.89)	0.03 (0.02-0.04)
**Norway**	**Acute myeloid leukemia**	Formaldehyde	0.05 (0.05-0.06)	0.3 (0.25-0.37)
**Norway**	**Acute lymphoid leukemia**	Benzene	0.63 (0.12-1.12)	0.27 (0.2-0.36)
**Norway**	**Acute lymphoid leukemia**	Formaldehyde	0.07 (0.06-0.08)	0.32 (0.26-0.38)
**Norway**	**Chronic myeloid leukemia**	Benzene	0.58 (0.11-1.02)	0.04 (0.03-0.04)
**Norway**	**Chronic myeloid leukemia**	Formaldehyde	0.06 (0.05-0.07)	0.05 (0.04-0.06)
**Norway**	**Chronic lymphoid leukemia**	Benzene	0.24 (0.04-0.42)	0.16 (0.12-0.2)
**Norway**	**Chronic lymphoid leukemia**	Formaldehyde	0.02 (0.02-0.03)	0.32 (0.26-0.42)
**Norway**	**Other leukemia**	Benzene	0.34 (0.06-0.59)	0.2 (0.16-0.26)
**Norway**	**Other leukemia**	Formaldehyde	0.04 (0.03-0.04)	0.31 (0.25-0.41)
**Oman**	**All leukemia**	Benzene	0.61 (0.18-1.02)	0.26 (0.16-0.35)
**Oman**	**All leukemia**	Formaldehyde	0.18 (0.13-0.23)	0.21 (0.15-0.29)
**Oman**	**Acute myeloid leukemia**	Benzene	0.76 (0.21-1.31)	0.28 (0.2-0.35)
**Oman**	**Acute myeloid leukemia**	Formaldehyde	0.22 (0.15-0.3)	0.21 (0.16-0.28)
**Oman**	**Acute lymphoid leukemia**	Benzene	0.51 (0.14-0.92)	0.33 (0.27-0.4)
**Oman**	**Acute lymphoid leukemia**	Formaldehyde	0.15 (0.1-0.22)	0.21 (0.16-0.26)

Countries	Causes	Occupational carcinogens	Percent change in 1990 (95% UI)	Percent change in 2019 (95% UI)
**Oman**	**Chronic myeloid leukemia**	Benzene	0.8 (0.23-1.38)	0.25 (0.21-0.3)
**Oman**	**Chronic myeloid leukemia**	Formaldehyde	0.23 (0.17-0.31)	0.25 (0.19-0.33)
**Oman**	**Chronic lymphoid leukemia**	Benzene	0.52 (0.15-0.89)	0.33 (0.25-0.43)
**Oman**	**Chronic lymphoid leukemia**	Formaldehyde	0.15 (0.11-0.21)	0.18 (0.11-0.26)
**Oman**	**Other leukemia**	Benzene	0.5 (0.15-0.87)	0.05 (0.04-0.06)
**Oman**	**Other leukemia**	Formaldehyde	0.14 (0.11-0.19)	0.52 (0.37-0.73)
**Pakistan**	**All leukemia**	Benzene	0.25 (0.07-0.46)	0.04 (0.03-0.05)
**Pakistan**	**All leukemia**	Formaldehyde	0.1 (0.07-0.15)	0.2 (0.12-0.3)
**Pakistan**	**Acute myeloid leukemia**	Benzene	0.31 (0.09-0.55)	0.04 (0.03-0.05)
**Pakistan**	**Acute myeloid leukemia**	Formaldehyde	0.13 (0.08-0.18)	0.24 (0.19-0.3)
**Pakistan**	**Acute lymphoid leukemia**	Benzene	0.19 (0.05-0.37)	0.26 (0.22-0.31)
**Pakistan**	**Acute lymphoid leukemia**	Formaldehyde	0.08 (0.04-0.13)	0.31 (0.25-0.38)
**Pakistan**	**Chronic myeloid leukemia**	Benzene	0.43 (0.12-0.81)	0.32 (0.24-0.41)
**Pakistan**	**Chronic myeloid leukemia**	Formaldehyde	0.18 (0.09-0.26)	0.32 (0.27-0.39)
**Pakistan**	**Chronic lymphoid leukemia**	Benzene	0.1 (0.03-0.18)	0.34 (0.26-0.42)
**Pakistan**	**Chronic lymphoid leukemia**	Formaldehyde	0.04 (0.03-0.05)	0.19 (0.12-0.28)
**Pakistan**	**Other leukemia**	Benzene	0.21 (0.06-0.41)	0.25 (0.15-0.35)
**Pakistan**	**Other leukemia**	Formaldehyde	0.09 (0.05-0.14)	0.34 (0.25-0.44)
**Palau**	**All leukemia**	Benzene	0.79 (0.23-1.37)	0.5 (0.14-0.86)
**Palau**	**All leukemia**	Formaldehyde	0.26 (0.2-0.34)	0.35 (0.07-0.63)
**Palau**	**Acute myeloid leukemia**	Benzene	1.11 (0.33-1.85)	0.57 (0.17-0.96)
**Palau**	**Acute myeloid leukemia**	Formaldehyde	0.37 (0.29-0.45)	0.88 (0.25-1.46)
**Palau**	**Acute lymphoid leukemia**	Benzene	0.91 (0.28-1.59)	0.4 (0.07-0.72)
**Palau**	**Acute lymphoid leukemia**	Formaldehyde	0.29 (0.21-0.39)	0.73 (0.22-1.25)
**Palau**	**Chronic myeloid leukemia**	Benzene	0.84 (0.24-1.48)	1 (0.31-1.69)
**Palau**	**Chronic myeloid leukemia**	Formaldehyde	0.27 (0.2-0.37)	0.82 (0.24-1.33)
**Palau**	**Chronic lymphoid leukemia**	Benzene	0.77 (0.22-1.36)	0.68 (0.2-1.12)
**Palau**	**Chronic lymphoid leukemia**	Formaldehyde	0.26 (0.2-0.36)	0.31 (0.06-0.55)
**Palau**	**Other leukemia**	Benzene	0.56 (0.15-1.04)	0.3 (0.06-0.53)
**Palau**	**Other leukemia**	Formaldehyde	0.18 (0.13-0.27)	0.99 (0.3-1.64)
**Palestine**	**All leukemia**	Benzene	0.35 (0.11-0.6)	1.36 (0.4-2.29)
**Palestine**	**All leukemia**	Formaldehyde	0.12 (0.09-0.15)	1.29 (0.39-2.14)
**Palestine**	**Acute myeloid leukemia**	Benzene	0.54 (0.15-1.01)	0.55 (0.16-0.97)
**Palestine**	**Acute myeloid leukemia**	Formaldehyde	0.18 (0.11-0.29)	0.82 (0.24-1.34)
**Palestine**	**Acute lymphoid leukemia**	Benzene	0.37 (0.1-0.7)	0.41 (0.08-0.73)
**Palestine**	**Acute lymphoid leukemia**	Formaldehyde	0.13 (0.07-0.19)	0.26 (0.05-0.46)
**Palestine**	**Chronic myeloid leukemia**	Benzene	0.3 (0.08-0.54)	1.26 (0.38-2.08)
**Palestine**	**Chronic myeloid leukemia**	Formaldehyde	0.09 (0.06-0.14)	0.58 (0.17-1.02)
**Palestine**	**Chronic lymphoid leukemia**	Benzene	0.24 (0.07-0.43)	0.74 (0.22-1.24)
**Palestine**	**Chronic lymphoid leukemia**	Formaldehyde	0.08 (0.05-0.11)	0.71 (0.2-1.2)
**Palestine**	**Other leukemia**	Benzene	0.35 (0.11-0.6)	0.93 (0.27-1.54)
**Palestine**	**Other leukemia**	Formaldehyde	0.12 (0.09-0.15)	0.24 (0.04-0.42)
**Panama**	**All leukemia**	Benzene	0.71 (0.21-1.19)	0.81 (0.24-1.41)
**Panama**	**All leukemia**	Formaldehyde	0.21 (0.18-0.26)	0.79 (0.23-1.3)

Countries	Causes	Occupational carcinogens	Percent change in 1990 (95% UI)	Percent change in 2019 (95% UI)
**Panama**	**Acute myeloid leukemia**	Benzene	0.76 (0.23-1.28)	1.1 (0.2-1.97)
**Panama**	**Acute myeloid leukemia**	Formaldehyde	0.22 (0.18-0.27)	0.4 (0.07-0.71)
**Panama**	**Acute lymphoid leukemia**	Benzene	0.62 (0.19-1.03)	0.33 (0.09-0.59)
**Panama**	**Acute lymphoid leukemia**	Formaldehyde	0.18 (0.14-0.23)	0.54 (0.15-0.92)
**Panama**	**Chronic myeloid leukemia**	Benzene	1.02 (0.3-1.7)	0.47 (0.14-0.79)
**Panama**	**Chronic myeloid leukemia**	Formaldehyde	0.3 (0.24-0.38)	0.75 (0.22-1.23)
**Panama**	**Chronic lymphoid leukemia**	Benzene	0.37 (0.11-0.61)	0.71 (0.21-1.21)
**Panama**	**Chronic lymphoid leukemia**	Formaldehyde	0.11 (0.09-0.14)	0.3 (0.06-0.53)
**Panama**	**Other leukemia**	Benzene	0.75 (0.22-1.24)	0.45 (0.13-0.77)
**Panama**	**Other leukemia**	Formaldehyde	0.22 (0.18-0.27)	0.36 (0.1-0.63)
**Papua New Guinea**	**All leukemia**	Benzene	0.39 (0.11-0.68)	0.74 (0.21-1.23)
**Papua New Guinea**	**All leukemia**	Formaldehyde	0.11 (0.08-0.15)	0.67 (0.19-1.1)
**Papua New Guinea**	**Acute myeloid leukemia**	Benzene	0.53 (0.15-0.94)	1.02 (0.3-1.68)
**Papua New Guinea**	**Acute myeloid leukemia**	Formaldehyde	0.16 (0.11-0.22)	0.66 (0.19-1.14)
**Papua New Guinea**	**Acute lymphoid leukemia**	Benzene	0.28 (0.08-0.56)	0.75 (0.22-1.27)
**Papua New Guinea**	**Acute lymphoid leukemia**	Formaldehyde	0.08 (0.05-0.13)	0.96 (0.28-1.57)
**Papua New Guinea**	**Chronic myeloid leukemia**	Benzene	0.6 (0.16-1.04)	0.88 (0.26-1.45)
**Papua New Guinea**	**Chronic myeloid leukemia**	Formaldehyde	0.18 (0.12-0.26)	0.21 (0.04-0.38)
**Papua New Guinea**	**Chronic lymphoid leukemia**	Benzene	0.52 (0.15-0.91)	0.76 (0.22-1.26)
**Papua New Guinea**	**Chronic lymphoid leukemia**	Formaldehyde	0.15 (0.11-0.21)	0.34 (0.06-0.61)
**Papua New Guinea**	**Other leukemia**	Benzene	0.34 (0.09-0.6)	0.29 (0.06-0.51)
**Papua New Guinea**	**Other leukemia**	Formaldehyde	0.1 (0.07-0.14)	0.65 (0.2-1.09)
**Paraguay**	**All leukemia**	Benzene	0.69 (0.2-1.15)	0.77 (0.23-1.31)
**Paraguay**	**All leukemia**	Formaldehyde	0.25 (0.2-0.32)	0.64 (0.19-1.07)
**Paraguay**	**Acute myeloid leukemia**	Benzene	0.89 (0.26-1.51)	0.34 (0.06-0.6)
**Paraguay**	**Acute myeloid leukemia**	Formaldehyde	0.33 (0.25-0.42)	0.46 (0.13-0.78)
**Paraguay**	**Acute lymphoid leukemia**	Benzene	0.49 (0.14-0.85)	0.65 (0.19-1.1)
**Paraguay**	**Acute lymphoid leukemia**	Formaldehyde	0.18 (0.13-0.23)	1.01 (0.3-1.69)
**Paraguay**	**Chronic myeloid leukemia**	Benzene	0.94 (0.27-1.58)	1.07 (0.31-1.79)
**Paraguay**	**Chronic myeloid leukemia**	Formaldehyde	0.35 (0.27-0.44)	0.75 (0.21-1.29)
**Paraguay**	**Chronic lymphoid leukemia**	Benzene	0.4 (0.11-0.69)	0.98 (0.29-1.6)
**Paraguay**	**Chronic lymphoid leukemia**	Formaldehyde	0.15 (0.12-0.19)	0.96 (0.29-1.68)
**Paraguay**	**Other leukemia**	Benzene	0.71 (0.2-1.19)	0.65 (0.19-1.1)
**Paraguay**	**Other leukemia**	Formaldehyde	0.26 (0.2-0.32)	0.29 (0.06-0.52)
**Peru**	**All leukemia**	Benzene	0.86 (0.25-1.4)	0.49 (0.15-0.84)
**Peru**	**All leukemia**	Formaldehyde	0.31 (0.25-0.38)	0.62 (0.17-1.09)
**Peru**	**Acute myeloid leukemia**	Benzene	0.85 (0.25-1.42)	0.89 (0.26-1.5)
**Peru**	**Acute myeloid leukemia**	Formaldehyde	0.31 (0.24-0.38)	0.32 (0.06-0.57)
**Peru**	**Acute lymphoid leukemia**	Benzene	0.56 (0.16-0.98)	0.24 (0.05-0.43)
**Peru**	**Acute lymphoid leukemia**	Formaldehyde	0.21 (0.15-0.28)	0.5 (0.15-0.84)
**Peru**	**Chronic myeloid leukemia**	Benzene	1.18 (0.37-1.97)	0.61 (0.17-1.02)
**Peru**	**Chronic myeloid leukemia**	Formaldehyde	0.43 (0.34-0.55)	0.71 (0.2-1.17)
**Peru**	**Chronic lymphoid leukemia**	Benzene	1 (0.28-1.68)	0.3 (0.05-0.56)
**Peru**	**Chronic lymphoid leukemia**	Formaldehyde	0.37 (0.29-0.46)	0.83 (0.25-1.37)

Countries	Causes	Occupational carcinogens	Percent change in 1990 (95% UI)	Percent change in 2019 (95% UI)
**Peru**	**Other leukemia**	Benzene	1 (0.29-1.64)	0.17 (0.03-0.3)
**Peru**	**Other leukemia**	Formaldehyde	0.36 (0.28-0.45)	0.35 (0.07-0.63)
**Philippines**	**All leukemia**	Benzene	0.64 (0.18-1.07)	0.92 (0.27-1.52)
**Philippines**	**All leukemia**	Formaldehyde	0.25 (0.19-0.31)	0.93 (0.28-1.51)
**Philippines**	**Acute myeloid leukemia**	Benzene	0.8 (0.23-1.33)	1.06 (0.31-1.76)
**Philippines**	**Acute myeloid leukemia**	Formaldehyde	0.31 (0.24-0.38)	0.43 (0.12-0.71)
**Philippines**	**Acute lymphoid leukemia**	Benzene	0.44 (0.12-0.79)	0.63 (0.19-1.06)
**Philippines**	**Acute lymphoid leukemia**	Formaldehyde	0.17 (0.12-0.24)	0.96 (0.28-1.59)
**Philippines**	**Chronic myeloid leukemia**	Benzene	0.97 (0.28-1.61)	0.51 (0.15-0.88)
**Philippines**	**Chronic myeloid leukemia**	Formaldehyde	0.38 (0.31-0.46)	0.78 (0.22-1.33)
**Philippines**	**Chronic lymphoid leukemia**	Benzene	0.61 (0.17-1.05)	0.32 (0.06-0.58)
**Philippines**	**Chronic lymphoid leukemia**	Formaldehyde	0.24 (0.19-0.32)	0.48 (0.09-0.87)
**Philippines**	**Other leukemia**	Benzene	0.61 (0.17-1.04)	0.56 (0.16-0.92)
**Philippines**	**Other leukemia**	Formaldehyde	0.24 (0.17-0.3)	0.85 (0.26-1.41)
**Poland**	**All leukemia**	Benzene	0.31 (0.06-0.56)	0.53 (0.15-0.88)
**Poland**	**All leukemia**	Formaldehyde	0.05 (0.04-0.06)	0.7 (0.2-1.17)
**Poland**	**Acute myeloid leukemia**	Benzene	0.43 (0.08-0.76)	0.33 (0.06-0.58)
**Poland**	**Acute myeloid leukemia**	Formaldehyde	0.07 (0.05-0.08)	0.4 (0.07-0.71)
**Poland**	**Acute lymphoid leukemia**	Benzene	0.39 (0.07-0.7)	0.21 (0.04-0.38)
**Poland**	**Acute lymphoid leukemia**	Formaldehyde	0.06 (0.05-0.08)	1.05 (0.3-1.73)
**Poland**	**Chronic myeloid leukemia**	Benzene	0.37 (0.07-0.65)	0.34 (0.06-0.6)
**Poland**	**Chronic myeloid leukemia**	Formaldehyde	0.06 (0.05-0.07)	0.54 (0.15-0.89)
**Poland**	**Chronic lymphoid leukemia**	Benzene	0.19 (0.04-0.33)	0.91 (0.26-1.49)
**Poland**	**Chronic lymphoid leukemia**	Formaldehyde	0.03 (0.02-0.03)	1.14 (0.35-1.92)
**Poland**	**Other leukemia**	Benzene	0.22 (0.04-0.39)	0.92 (0.27-1.55)
**Poland**	**Other leukemia**	Formaldehyde	0.03 (0.03-0.04)	1.41 (0.43-2.31)
**Portugal**	**All leukemia**	Benzene	0.4 (0.08-0.71)	0.86 (0.25-1.45)
**Portugal**	**All leukemia**	Formaldehyde	0.06 (0.05-0.07)	0.84 (0.26-1.42)
**Portugal**	**Acute myeloid leukemia**	Benzene	0.51 (0.1-0.91)	0.3 (0.05-0.54)
**Portugal**	**Acute myeloid leukemia**	Formaldehyde	0.08 (0.06-0.09)	0.63 (0.18-1.08)
**Portugal**	**Acute lymphoid leukemia**	Benzene	0.49 (0.1-0.88)	0.58 (0.17-0.98)
**Portugal**	**Acute lymphoid leukemia**	Formaldehyde	0.07 (0.06-0.09)	0.71 (0.21-1.17)
**Portugal**	**Chronic myeloid leukemia**	Benzene	0.42 (0.08-0.76)	0.74 (0.22-1.26)
**Portugal**	**Chronic myeloid leukemia**	Formaldehyde	0.06 (0.05-0.08)	0.29 (0.06-0.52)
**Portugal**	**Chronic lymphoid leukemia**	Benzene	0.2 (0.04-0.36)	0.35 (0.07-0.63)
**Portugal**	**Chronic lymphoid leukemia**	Formaldehyde	0.03 (0.02-0.04)	0.62 (0.18-1.05)
**Portugal**	**Other leukemia**	Benzene	0.37 (0.07-0.65)	0.82 (0.24-1.38)
**Portugal**	**Other leukemia**	Formaldehyde	0.06 (0.05-0.07)	0.98 (0.29-1.62)
**Puerto Rico**	**All leukemia**	Benzene	0.36 (0.07-0.65)	1.02 (0.29-1.76)
**Puerto Rico**	**All leukemia**	Formaldehyde	0.04 (0.03-0.05)	0.44 (0.13-0.76)
**Puerto Rico**	**Acute myeloid leukemia**	Benzene	0.49 (0.09-0.88)	0.35 (0.07-0.63)
**Puerto Rico**	**Acute myeloid leukemia**	Formaldehyde	0.05 (0.04-0.07)	1.1 (0.32-1.85)
**Puerto Rico**	**Acute lymphoid leukemia**	Benzene	0.4 (0.07-0.72)	0.4 (0.11-0.69)
**Puerto Rico**	**Acute lymphoid leukemia**	Formaldehyde	0.04 (0.04-0.06)	0.85 (0.24-1.4)

Countries	Causes	Occupational carcinogens	Percent change in 1990 (95% UI)	Percent change in 2019 (95% UI)
**Puerto Rico**	**Chronic myeloid leukemia**	Benzene	0.4 (0.08-0.74)	1 (0.3-1.65)
**Puerto Rico**	**Chronic myeloid leukemia**	Formaldehyde	0.05 (0.04-0.05)	1.1 (0.32-1.92)
**Puerto Rico**	**Chronic lymphoid leukemia**	Benzene	0.16 (0.03-0.29)	0.29 (0.05-0.53)
**Puerto Rico**	**Chronic lymphoid leukemia**	Formaldehyde	0.02 (0.01-0.02)	0.99 (0.3-1.64)
**Puerto Rico**	**Other leukemia**	Benzene	0.3 (0.06-0.53)	0.34 (0.07-0.61)
**Puerto Rico**	**Other leukemia**	Formaldehyde	0.03 (0.03-0.04)	0.56 (0.17-0.95)
**Qatar**	**All leukemia**	Benzene	1.54 (0.45-2.6)	0.35 (0.1-0.69)
**Qatar**	**All leukemia**	Formaldehyde	0.44 (0.32-0.59)	0.6 (0.18-0.99)
**Qatar**	**Acute myeloid leukemia**	Benzene	1.77 (0.51-3)	0.53 (0.16-0.91)
**Qatar**	**Acute myeloid leukemia**	Formaldehyde	0.51 (0.35-0.73)	1.42 (0.42-2.39)
**Qatar**	**Acute lymphoid leukemia**	Benzene	1.18 (0.32-2.15)	0.66 (0.2-1.1)
**Qatar**	**Acute lymphoid leukemia**	Formaldehyde	0.36 (0.23-0.53)	0.35 (0.07-0.61)
**Qatar**	**Chronic myeloid leukemia**	Benzene	2.17 (0.63-3.63)	0.4 (0.08-0.71)
**Qatar**	**Chronic myeloid leukemia**	Formaldehyde	0.64 (0.46-0.86)	0.93 (0.27-1.56)
**Qatar**	**Chronic lymphoid leukemia**	Benzene	0.75 (0.21-1.25)	0.4 (0.11-0.72)
**Qatar**	**Chronic lymphoid leukemia**	Formaldehyde	0.18 (0.14-0.23)	0.61 (0.16-1.05)
**Qatar**	**Other leukemia**	Benzene	1.4 (0.4-2.32)	0.72 (0.2-1.22)
**Qatar**	**Other leukemia**	Formaldehyde	0.4 (0.28-0.55)	0.34 (0.07-0.61)
**Republic of Korea**	**All leukemia**	Benzene	0.61 (0.12-1.09)	0.87 (0.25-1.45)
**Republic of Korea**	**All leukemia**	Formaldehyde	0.1 (0.08-0.12)	0.43 (0.08-0.76)
**Republic of Korea**	**Acute myeloid leukemia**	Benzene	0.66 (0.13-1.17)	1.08 (0.31-1.86)
**Republic of Korea**	**Acute myeloid leukemia**	Formaldehyde	0.1 (0.08-0.13)	0.52 (0.15-0.88)
**Republic of Korea**	**Acute lymphoid leukemia**	Benzene	0.47 (0.08-0.85)	1.03 (0.29-1.75)
**Republic of Korea**	**Acute lymphoid leukemia**	Formaldehyde	0.07 (0.06-0.1)	0.6 (0.18-1)
**Republic of Korea**	**Chronic myeloid leukemia**	Benzene	0.91 (0.15-1.63)	1.01 (0.29-1.67)
**Republic of Korea**	**Chronic myeloid leukemia**	Formaldehyde	0.14 (0.11-0.18)	0.55 (0.16-0.93)
**Republic of Korea**	**Chronic lymphoid leukemia**	Benzene	0.48 (0.09-0.86)	1.07 (0.32-1.75)
**Republic of Korea**	**Chronic lymphoid leukemia**	Formaldehyde	0.08 (0.06-0.09)	1.18 (0.34-1.95)
**Republic of Korea**	**Other leukemia**	Benzene	0.58 (0.11-1.04)	0.93 (0.28-1.56)
**Republic of Korea**	**Other leukemia**	Formaldehyde	0.09 (0.07-0.11)	0.3 (0.06-0.54)
**Republic of Moldova**	**All leukemia**	Benzene	0.27 (0.05-0.5)	0.29 (0.06-0.51)
**Republic of Moldova**	**All leukemia**	Formaldehyde	0.04 (0.03-0.04)	0.24 (0.04-0.44)
**Republic of Moldova**	**Acute myeloid leukemia**	Benzene	0.34 (0.06-0.63)	1.76 (0.53-2.93)
**Republic of Moldova**	**Acute myeloid leukemia**	Formaldehyde	0.04 (0.03-0.05)	0.51 (0.1-0.92)
**Republic of Moldova**	**Acute lymphoid leukemia**	Benzene	0.22 (0.04-0.4)	0.31 (0.06-0.55)
**Republic of Moldova**	**Acute lymphoid leukemia**	Formaldehyde	0.03 (0.02-0.04)	0.32 (0.06-0.58)
**Republic of Moldova**	**Chronic myeloid leukemia**	Benzene	0.34 (0.06-0.62)	0.43 (0.08-0.77)
**Republic of Moldova**	**Chronic myeloid leukemia**	Formaldehyde	0.04 (0.04-0.05)	0.52 (0.15-0.92)
**Republic of Moldova**	**Chronic lymphoid leukemia**	Benzene	0.17 (0.03-0.31)	0.85 (0.24-1.49)
**Republic of Moldova**	**Chronic lymphoid leukemia**	Formaldehyde	0.02 (0.02-0.03)	0.81 (0.24-1.35)
**Republic of Moldova**	**Other leukemia**	Benzene	0.29 (0.05-0.53)	0.78 (0.22-1.28)
**Republic of Moldova**	**Other leukemia**	Formaldehyde	0.04 (0.03-0.05)	0.53 (0.15-0.93)
**Romania**	**All leukemia**	Benzene	0.43 (0.08-0.76)	0.29 (0.05-0.52)
**Romania**	**All leukemia**	Formaldehyde	0.08 (0.07-0.1)	0.59 (0.17-0.97)

Countries	Causes	Occupational carcinogens	Percent change in 1990 (95% UI)	Percent change in 2019 (95% UI)
**Romania**	**Acute myeloid leukemia**	Benzene	0.5 (0.1-0.89)	1.61 (0.5-2.69)
**Romania**	**Acute myeloid leukemia**	Formaldehyde	0.1 (0.08-0.11)	0.44 (0.13-0.75)
**Romania**	**Acute lymphoid leukemia**	Benzene	0.35 (0.06-0.62)	0.3 (0.06-0.54)
**Romania**	**Acute lymphoid leukemia**	Formaldehyde	0.07 (0.05-0.08)	1.07 (0.31-1.76)
**Romania**	**Chronic myeloid leukemia**	Benzene	0.52 (0.09-0.92)	0.46 (0.13-0.81)
**Romania**	**Chronic myeloid leukemia**	Formaldehyde	0.1 (0.08-0.12)	0.64 (0.12-1.13)
**Romania**	**Chronic lymphoid leukemia**	Benzene	0.29 (0.05-0.52)	0.37 (0.07-0.66)
**Romania**	**Chronic lymphoid leukemia**	Formaldehyde	0.06 (0.04-0.07)	0.24 (0.05-0.43)
**Romania**	**Other leukemia**	Benzene	0.46 (0.09-0.81)	1.41 (0.43-2.49)
**Romania**	**Other leukemia**	Formaldehyde	0.09 (0.07-0.1)	0.4 (0.12-0.71)
**Russian Federation**	**All leukemia**	Benzene	0.46 (0.08-0.82)	0.48 (0.14-0.81)
**Russian Federation**	**All leukemia**	Formaldehyde	0.06 (0.05-0.07)	0.31 (0.09-0.57)
**Russian Federation**	**Acute myeloid leukemia**	Benzene	0.6 (0.11-1.07)	0.23 (0.04-0.41)
**Russian Federation**	**Acute myeloid leukemia**	Formaldehyde	0.07 (0.06-0.09)	0.68 (0.19-1.12)
**Russian Federation**	**Acute lymphoid leukemia**	Benzene	0.47 (0.09-0.83)	0.64 (0.19-1.08)
**Russian Federation**	**Acute lymphoid leukemia**	Formaldehyde	0.06 (0.05-0.07)	1.01 (0.3-1.67)
**Russian Federation**	**Chronic myeloid leukemia**	Benzene	0.49 (0.09-0.88)	0.33 (0.06-0.59)
**Russian Federation**	**Chronic myeloid leukemia**	Formaldehyde	0.06 (0.05-0.07)	0.36 (0.06-0.65)
**Russian Federation**	**Chronic lymphoid leukemia**	Benzene	0.27 (0.05-0.49)	0.48 (0.14-0.81)
**Russian Federation**	**Chronic lymphoid leukemia**	Formaldehyde	0.03 (0.03-0.04)	0.63 (0.18-1.05)
**Russian Federation**	**Other leukemia**	Benzene	0.43 (0.08-0.78)	0.65 (0.19-1.1)
**Russian Federation**	**Other leukemia**	Formaldehyde	0.05 (0.04-0.06)	0.68 (0.2-1.15)
**Rwanda**	**All leukemia**	Benzene	0.3 (0.08-0.51)	0.58 (0.17-1)
**Rwanda**	**All leukemia**	Formaldehyde	0.09 (0.07-0.12)	0.7 (0.21-1.16)
**Rwanda**	**Acute myeloid leukemia**	Benzene	0.39 (0.11-0.77)	0.85 (0.24-1.44)
**Rwanda**	**Acute myeloid leukemia**	Formaldehyde	0.12 (0.07-0.18)	0.53 (0.16-0.89)
**Rwanda**	**Acute lymphoid leukemia**	Benzene	0.16 (0.04-0.34)	0.91 (0.26-1.52)
**Rwanda**	**Acute lymphoid leukemia**	Formaldehyde	0.05 (0.03-0.08)	0.51 (0.15-0.86)
**Rwanda**	**Chronic myeloid leukemia**	Benzene	0.49 (0.13-0.92)	0.54 (0.16-0.89)
**Rwanda**	**Chronic myeloid leukemia**	Formaldehyde	0.15 (0.08-0.22)	0.8 (0.23-1.36)
**Rwanda**	**Chronic lymphoid leukemia**	Benzene	0.23 (0.07-0.39)	0.96 (0.28-1.6)
**Rwanda**	**Chronic lymphoid leukemia**	Formaldehyde	0.07 (0.05-0.09)	0.46 (0.13-0.8)
**Rwanda**	**Other leukemia**	Benzene	0.27 (0.07-0.48)	0.4 (0.07-0.72)
**Rwanda**	**Other leukemia**	Formaldehyde	0.08 (0.06-0.12)	1.94 (0.59-3.31)
**Saint Kitts and Nevis**	**All leukemia**	Benzene	0.94 (0.27-1.55)	0.34 (0.06-0.62)
**Saint Kitts and Nevis**	**All leukemia**	Formaldehyde	0.29 (0.24-0.35)	0.47 (0.14-0.82)
**Saint Kitts and Nevis**	**Acute myeloid leukemia**	Benzene	1.08 (0.32-1.82)	0.34 (0.06-0.6)
**Saint Kitts and Nevis**	**Acute myeloid leukemia**	Formaldehyde	0.35 (0.27-0.44)	0.74 (0.21-1.22)
**Saint Kitts and Nevis**	**Acute lymphoid leukemia**	Benzene	0.6 (0.16-1.07)	0.67 (0.19-1.13)
**Saint Kitts and Nevis**	**Acute lymphoid leukemia**	Formaldehyde	0.19 (0.14-0.25)	1.17 (0.35-1.93)
**Saint Kitts and Nevis**	**Chronic myeloid leukemia**	Benzene	1.13 (0.34-1.86)	1 (0.3-1.66)
**Saint Kitts and Nevis**	**Chronic myeloid leukemia**	Formaldehyde	0.36 (0.29-0.43)	0.89 (0.26-1.47)
**Saint Kitts and Nevis**	**Chronic lymphoid leukemia**	Benzene	0.53 (0.16-0.88)	0.76 (0.23-1.26)
**Saint Kitts and Nevis**	**Chronic lymphoid leukemia**	Formaldehyde	0.18 (0.13-0.22)	0.51 (0.15-0.88)

Countries	Causes	Occupational carcinogens	Percent change in 1990 (95% UI)	Percent change in 2019 (95% UI)
**Saint Kitts and Nevis**	**Other leukemia**	Benzene	1 (0.29-1.63)	0.67 (0.2-1.23)
**Saint Kitts and Nevis**	**Other leukemia**	Formaldehyde	0.3 (0.24-0.37)	0.65 (0.19-1.1)
**Saint Lucia**	**All leukemia**	Benzene	0.63 (0.18-1.04)	0.45 (0.13-0.79)
**Saint Lucia**	**All leukemia**	Formaldehyde	0.24 (0.19-0.28)	0.32 (0.06-0.58)
**Saint Lucia**	**Acute myeloid leukemia**	Benzene	0.78 (0.23-1.32)	0.53 (0.16-0.92)
**Saint Lucia**	**Acute myeloid leukemia**	Formaldehyde	0.29 (0.23-0.37)	0.71 (0.2-1.18)
**Saint Lucia**	**Acute lymphoid leukemia**	Benzene	0.43 (0.13-0.72)	0.31 (0.05-0.56)
**Saint Lucia**	**Acute lymphoid leukemia**	Formaldehyde	0.16 (0.12-0.21)	0.71 (0.2-1.27)
**Saint Lucia**	**Chronic myeloid leukemia**	Benzene	0.79 (0.23-1.31)	0.9 (0.27-1.51)
**Saint Lucia**	**Chronic myeloid leukemia**	Formaldehyde	0.3 (0.24-0.36)	0.7 (0.21-1.16)
**Saint Lucia**	**Chronic lymphoid leukemia**	Benzene	0.38 (0.11-0.64)	0.68 (0.2-1.13)
**Saint Lucia**	**Chronic lymphoid leukemia**	Formaldehyde	0.14 (0.11-0.17)	0.21 (0.04-0.38)
**Saint Lucia**	**Other leukemia**	Benzene	0.66 (0.19-1.09)	0.24 (0.04-0.44)
**Saint Lucia**	**Other leukemia**	Formaldehyde	0.25 (0.2-0.3)	0.95 (0.29-1.62)
**Saint Vincent and the Grenadines**	**All leukemia**	Benzene	0.6 (0.18-1.01)	1.27 (0.38-2.14)
**Saint Vincent and the Grenadines**	**All leukemia**	Formaldehyde	0.2 (0.16-0.24)	1.32 (0.37-2.3)
**Saint Vincent and the Grenadines**	**Acute myeloid leukemia**	Benzene	0.69 (0.2-1.21)	0.5 (0.14-0.86)
**Saint Vincent and the Grenadines**	**Acute myeloid leukemia**	Formaldehyde	0.23 (0.17-0.3)	0.68 (0.2-1.12)
**Saint Vincent and the Grenadines**	**Acute lymphoid leukemia**	Benzene	0.41 (0.12-0.72)	0.33 (0.06-0.6)
**Saint Vincent and the Grenadines**	**Acute lymphoid leukemia**	Formaldehyde	0.14 (0.09-0.19)	0.21 (0.04-0.38)
**Saint Vincent and the Grenadines**	**Chronic myeloid leukemia**	Benzene	0.72 (0.22-1.22)	1.33 (0.39-2.19)
**Saint Vincent and the Grenadines**	**Chronic myeloid leukemia**	Formaldehyde	0.23 (0.17-0.31)	0.6 (0.17-1.01)
**Saint Vincent and the Grenadines**	**Chronic lymphoid leukemia**	Benzene	0.4 (0.12-0.69)	0.6 (0.18-0.99)
**Saint Vincent and the Grenadines**	**Chronic lymphoid leukemia**	Formaldehyde	0.13 (0.1-0.17)	0.63 (0.19-1.11)
**Saint Vincent and the Grenadines**	**Other leukemia**	Benzene	0.59 (0.17-1)	0.98 (0.28-1.64)
**Saint Vincent and the Grenadines**	**Other leukemia**	Formaldehyde	0.2 (0.16-0.25)	0.21 (0.04-0.37)
**Samoa**	**All leukemia**	Benzene	0.51 (0.14-0.87)	0.87 (0.26-1.48)
**Samoa**	**All leukemia**	Formaldehyde	0.18 (0.14-0.23)	0.67 (0.19-1.1)
**Samoa**	**Acute myeloid leukemia**	Benzene	0.66 (0.19-1.14)	1.03 (0.2-1.86)
**Samoa**	**Acute myeloid leukemia**	Formaldehyde	0.24 (0.18-0.31)	0.39 (0.07-0.7)
**Samoa**	**Acute lymphoid leukemia**	Benzene	0.5 (0.14-0.9)	0.35 (0.1-0.61)
**Samoa**	**Acute lymphoid leukemia**	Formaldehyde	0.18 (0.12-0.24)	0.5 (0.14-0.88)
**Samoa**	**Chronic myeloid leukemia**	Benzene	0.55 (0.16-0.98)	0.44 (0.14-0.75)
**Samoa**	**Chronic myeloid leukemia**	Formaldehyde	0.21 (0.15-0.28)	0.75 (0.22-1.24)
**Samoa**	**Chronic lymphoid leukemia**	Benzene	0.4 (0.11-0.7)	0.75 (0.23-1.26)
**Samoa**	**Chronic lymphoid leukemia**	Formaldehyde	0.15 (0.11-0.2)	0.24 (0.04-0.44)
**Samoa**	**Other leukemia**	Benzene	0.43 (0.13-0.74)	0.45 (0.13-0.77)
**Samoa**	**Other leukemia**	Formaldehyde	0.15 (0.12-0.2)	0.38 (0.11-0.67)
**San Marino**	**All leukemia**	Benzene	0.29 (0.06-0.53)	0.59 (0.17-0.99)
**San Marino**	**All leukemia**	Formaldehyde	0.04 (0.03-0.04)	0.66 (0.19-1.08)
**San Marino**	**Acute myeloid leukemia**	Benzene	0.32 (0.06-0.59)	0.9 (0.26-1.51)
**San Marino**	**Acute myeloid leukemia**	Formaldehyde	0.04 (0.03-0.05)	0.63 (0.18-1.09)
**San Marino**	**Acute lymphoid leukemia**	Benzene	0.44 (0.09-0.81)	0.77 (0.22-1.32)
**San Marino**	**Acute lymphoid leukemia**	Formaldehyde	0.05 (0.04-0.06)	0.71 (0.2-1.18)

Countries	Causes	Occupational carcinogens	Percent change in 1990 (95% UI)	Percent change in 2019 (95% UI)
**San Marino**	**Chronic myeloid leukemia**	Benzene	0.33 (0.07-0.61)	0.7 (0.21-1.18)
**San Marino**	**Chronic myeloid leukemia**	Formaldehyde	0.04 (0.03-0.05)	0.18 (0.03-0.33)
**San Marino**	**Chronic lymphoid leukemia**	Benzene	0.26 (0.05-0.48)	0.6 (0.18-0.99)
**San Marino**	**Chronic lymphoid leukemia**	Formaldehyde	0.03 (0.02-0.04)	0.29 (0.06-0.53)
**San Marino**	**Other leukemia**	Benzene	0.24 (0.04-0.43)	0.23 (0.05-0.42)
**San Marino**	**Other leukemia**	Formaldehyde	0.03 (0.02-0.04)	0.67 (0.19-1.13)
**Sao Tome and Principe**	**All leukemia**	Benzene	0.31 (0.09-0.54)	0.79 (0.24-1.34)
**Sao Tome and Principe**	**All leukemia**	Formaldehyde	0.09 (0.06-0.12)	0.65 (0.19-1.13)
**Sao Tome and Principe**	**Acute myeloid leukemia**	Benzene	0.32 (0.08-0.6)	0.26 (0.05-0.46)
**Sao Tome and Principe**	**Acute myeloid leukemia**	Formaldehyde	0.09 (0.05-0.14)	0.41 (0.11-0.7)
**Sao Tome and Principe**	**Acute lymphoid leukemia**	Benzene	0.2 (0.06-0.38)	0.58 (0.17-0.97)
**Sao Tome and Principe**	**Acute lymphoid leukemia**	Formaldehyde	0.06 (0.03-0.09)	1.02 (0.3-1.71)
**Sao Tome and Principe**	**Chronic myeloid leukemia**	Benzene	0.61 (0.18-1.03)	1.07 (0.31-1.8)
**Sao Tome and Principe**	**Chronic myeloid leukemia**	Formaldehyde	0.17 (0.12-0.24)	0.6 (0.17-1.06)
**Sao Tome and Principe**	**Chronic lymphoid leukemia**	Benzene	0.15 (0.04-0.25)	0.99 (0.28-1.64)
**Sao Tome and Principe**	**Chronic lymphoid leukemia**	Formaldehyde	0.04 (0.03-0.05)	0.99 (0.27-1.76)
**Sao Tome and Principe**	**Other leukemia**	Benzene	0.31 (0.09-0.54)	0.62 (0.18-1.08)
**Sao Tome and Principe**	**Other leukemia**	Formaldehyde	0.1 (0.07-0.13)	0.19 (0.04-0.35)
**Saudi Arabia**	**All leukemia**	Benzene	0.68 (0.2-1.15)	0.54 (0.16-0.93)
**Saudi Arabia**	**All leukemia**	Formaldehyde	0.19 (0.14-0.25)	0.46 (0.12-0.93)
**Saudi Arabia**	**Acute myeloid leukemia**	Benzene	0.77 (0.22-1.34)	0.65 (0.19-1.11)
**Saudi Arabia**	**Acute myeloid leukemia**	Formaldehyde	0.21 (0.14-0.3)	0.21 (0.04-0.39)
**Saudi Arabia**	**Acute lymphoid leukemia**	Benzene	0.5 (0.14-0.91)	0.2 (0.04-0.36)
**Saudi Arabia**	**Acute lymphoid leukemia**	Formaldehyde	0.14 (0.08-0.22)	0.51 (0.15-0.88)
**Saudi Arabia**	**Chronic myeloid leukemia**	Benzene	0.92 (0.28-1.55)	0.57 (0.16-0.97)
**Saudi Arabia**	**Chronic myeloid leukemia**	Formaldehyde	0.26 (0.19-0.35)	0.68 (0.2-1.13)
**Saudi Arabia**	**Chronic lymphoid leukemia**	Benzene	0.39 (0.11-0.68)	0.21 (0.04-0.4)
**Saudi Arabia**	**Chronic lymphoid leukemia**	Formaldehyde	0.11 (0.07-0.15)	0.76 (0.23-1.28)
**Saudi Arabia**	**Other leukemia**	Benzene	0.69 (0.18-1.26)	0.12 (0.02-0.22)
**Saudi Arabia**	**Other leukemia**	Formaldehyde	0.19 (0.11-0.29)	0.29 (0.05-0.51)
**Senegal**	**All leukemia**	Benzene	0.33 (0.09-0.58)	0.84 (0.25-1.4)
**Senegal**	**All leukemia**	Formaldehyde	0.14 (0.1-0.18)	0.71 (0.21-1.18)
**Senegal**	**Acute myeloid leukemia**	Benzene	0.28 (0.08-0.53)	1.08 (0.33-1.81)
**Senegal**	**Acute myeloid leukemia**	Formaldehyde	0.11 (0.06-0.17)	0.49 (0.14-0.83)
**Senegal**	**Acute lymphoid leukemia**	Benzene	0.22 (0.06-0.41)	0.64 (0.19-1.08)
**Senegal**	**Acute lymphoid leukemia**	Formaldehyde	0.09 (0.05-0.13)	1 (0.29-1.67)
**Senegal**	**Chronic myeloid leukemia**	Benzene	0.73 (0.21-1.24)	0.58 (0.16-0.98)
**Senegal**	**Chronic myeloid leukemia**	Formaldehyde	0.29 (0.2-0.42)	0.79 (0.22-1.34)
**Senegal**	**Chronic lymphoid leukemia**	Benzene	0.16 (0.05-0.27)	0.28 (0.05-0.51)
**Senegal**	**Chronic lymphoid leukemia**	Formaldehyde	0.06 (0.04-0.08)	0.33 (0.06-0.6)
**Senegal**	**Other leukemia**	Benzene	0.34 (0.09-0.59)	0.49 (0.14-0.82)
**Senegal**	**Other leukemia**	Formaldehyde	0.15 (0.1-0.2)	0.84 (0.25-1.38)
**Serbia**	**All leukemia**	Benzene	0.28 (0.05-0.51)	0.34 (0.1-0.57)
**Serbia**	**All leukemia**	Formaldehyde	0.04 (0.03-0.05)	0.65 (0.19-1.11)

Countries	Causes	Occupational carcinogens	Percent change in 1990 (95% UI)	Percent change in 2019 (95% UI)
**Serbia**	**Acute myeloid leukemia**	Benzene	0.39 (0.07-0.73)	0.23 (0.04-0.42)
**Serbia**	**Acute myeloid leukemia**	Formaldehyde	0.06 (0.04-0.07)	0.32 (0.06-0.58)
**Serbia**	**Acute lymphoid leukemia**	Benzene	0.36 (0.07-0.67)	0.16 (0.03-0.29)
**Serbia**	**Acute lymphoid leukemia**	Formaldehyde	0.06 (0.04-0.07)	1.15 (0.32-1.89)
**Serbia**	**Chronic myeloid leukemia**	Benzene	0.36 (0.06-0.68)	0.25 (0.05-0.46)
**Serbia**	**Chronic myeloid leukemia**	Formaldehyde	0.05 (0.04-0.07)	0.53 (0.15-0.87)
**Serbia**	**Chronic lymphoid leukemia**	Benzene	0.27 (0.05-0.5)	0.6 (0.17-1.03)
**Serbia**	**Chronic lymphoid leukemia**	Formaldehyde	0.04 (0.03-0.05)	1.04 (0.31-1.76)
**Serbia**	**Other leukemia**	Benzene	0.23 (0.04-0.42)	0.88 (0.26-1.5)
**Serbia**	**Other leukemia**	Formaldehyde	0.03 (0.03-0.04)	1.27 (0.39-2.1)
**Seychelles**	**All leukemia**	Benzene	0.76 (0.22-1.28)	0.82 (0.24-1.39)
**Seychelles**	**All leukemia**	Formaldehyde	0.29 (0.23-0.35)	0.86 (0.26-1.45)
**Seychelles**	**Acute myeloid leukemia**	Benzene	0.91 (0.26-1.54)	0.35 (0.06-0.62)
**Seychelles**	**Acute myeloid leukemia**	Formaldehyde	0.34 (0.26-0.45)	0.42 (0.12-0.73)
**Seychelles**	**Acute lymphoid leukemia**	Benzene	0.8 (0.23-1.39)	0.65 (0.2-1.13)
**Seychelles**	**Acute lymphoid leukemia**	Formaldehyde	0.3 (0.22-0.4)	0.71 (0.21-1.2)
**Seychelles**	**Chronic myeloid leukemia**	Benzene	1.37 (0.41-2.34)	0.56 (0.17-0.99)
**Seychelles**	**Chronic myeloid leukemia**	Formaldehyde	0.52 (0.38-0.7)	0.2 (0.04-0.37)
**Seychelles**	**Chronic lymphoid leukemia**	Benzene	0.4 (0.12-0.67)	0.3 (0.06-0.53)
**Seychelles**	**Chronic lymphoid leukemia**	Formaldehyde	0.15 (0.12-0.19)	0.58 (0.17-1.01)
**Seychelles**	**Other leukemia**	Benzene	0.76 (0.22-1.3)	0.75 (0.21-1.28)
**Seychelles**	**Other leukemia**	Formaldehyde	0.29 (0.22-0.36)	0.93 (0.26-1.55)
**Sierra Leone**	**All leukemia**	Benzene	0.27 (0.08-0.51)	0.68 (0.19-1.16)
**Sierra Leone**	**All leukemia**	Formaldehyde	0.08 (0.05-0.11)	0.51 (0.15-0.86)
**Sierra Leone**	**Acute myeloid leukemia**	Benzene	0.25 (0.06-0.53)	0.25 (0.05-0.46)
**Sierra Leone**	**Acute myeloid leukemia**	Formaldehyde	0.07 (0.03-0.12)	0.99 (0.3-1.69)
**Sierra Leone**	**Acute lymphoid leukemia**	Benzene	0.17 (0.04-0.35)	0.41 (0.12-0.72)
**Sierra Leone**	**Acute lymphoid leukemia**	Formaldehyde	0.05 (0.02-0.08)	0.79 (0.22-1.32)
**Sierra Leone**	**Chronic myeloid leukemia**	Benzene	0.59 (0.17-1.05)	0.92 (0.27-1.52)
**Sierra Leone**	**Chronic myeloid leukemia**	Formaldehyde	0.16 (0.11-0.22)	0.99 (0.29-1.72)
**Sierra Leone**	**Chronic lymphoid leukemia**	Benzene	0.15 (0.04-0.25)	0.26 (0.05-0.47)
**Sierra Leone**	**Chronic lymphoid leukemia**	Formaldehyde	0.04 (0.03-0.05)	0.59 (0.15-1.35)
**Sierra Leone**	**Other leukemia**	Benzene	0.27 (0.08-0.49)	0.26 (0.05-0.47)
**Sierra Leone**	**Other leukemia**	Formaldehyde	0.08 (0.05-0.11)	0.44 (0.13-0.75)
**Singapore**	**All leukemia**	Benzene	0.59 (0.11-1.06)	0.32 (0.09-0.62)
**Singapore**	**All leukemia**	Formaldehyde	0.08 (0.07-0.09)	0.6 (0.18-1.01)
**Singapore**	**Acute myeloid leukemia**	Benzene	0.61 (0.11-1.08)	0.56 (0.17-0.97)
**Singapore**	**Acute myeloid leukemia**	Formaldehyde	0.08 (0.07-0.1)	1.32 (0.39-2.25)
**Singapore**	**Acute lymphoid leukemia**	Benzene	0.59 (0.11-1.05)	0.59 (0.17-1.01)
**Singapore**	**Acute lymphoid leukemia**	Formaldehyde	0.08 (0.06-0.1)	0.24 (0.05-0.43)
**Singapore**	**Chronic myeloid leukemia**	Benzene	0.77 (0.15-1.37)	0.29 (0.05-0.52)
**Singapore**	**Chronic myeloid leukemia**	Formaldehyde	0.1 (0.09-0.13)	0.93 (0.27-1.56)
**Singapore**	**Chronic lymphoid leukemia**	Benzene	0.31 (0.06-0.56)	0.44 (0.12-0.8)
**Singapore**	**Chronic lymphoid leukemia**	Formaldehyde	0.04 (0.03-0.05)	0.61 (0.17-1.05)

Countries	Causes	Occupational carcinogens	Percent change in 1990 (95% UI)	Percent change in 2019 (95% UI)
**Singapore**	**Other leukemia**	Benzene	0.46 (0.09-0.83)	0.55 (0.15-0.95)
**Singapore**	**Other leukemia**	Formaldehyde	0.06 (0.05-0.07)	0.39 (0.07-0.72)
**Slovakia**	**All leukemia**	Benzene	0.36 (0.07-0.64)	0.75 (0.22-1.25)
**Slovakia**	**All leukemia**	Formaldehyde	0.05 (0.04-0.06)	0.32 (0.06-0.58)
**Slovakia**	**Acute myeloid leukemia**	Benzene	0.44 (0.08-0.81)	0.77 (0.21-1.33)
**Slovakia**	**Acute myeloid leukemia**	Formaldehyde	0.06 (0.05-0.08)	0.5 (0.15-0.88)
**Slovakia**	**Acute lymphoid leukemia**	Benzene	0.44 (0.08-0.8)	0.73 (0.2-1.32)
**Slovakia**	**Acute lymphoid leukemia**	Formaldehyde	0.06 (0.05-0.08)	0.56 (0.16-0.93)
**Slovakia**	**Chronic myeloid leukemia**	Benzene	0.4 (0.07-0.71)	0.92 (0.27-1.55)
**Slovakia**	**Chronic myeloid leukemia**	Formaldehyde	0.06 (0.05-0.08)	0.48 (0.14-0.82)
**Slovakia**	**Chronic lymphoid leukemia**	Benzene	0.17 (0.03-0.31)	0.99 (0.3-1.63)
**Slovakia**	**Chronic lymphoid leukemia**	Formaldehyde	0.03 (0.02-0.03)	1.21 (0.35-1.98)
**Slovakia**	**Other leukemia**	Benzene	0.33 (0.06-0.61)	0.92 (0.27-1.54)
**Slovakia**	**Other leukemia**	Formaldehyde	0.05 (0.04-0.06)	0.22 (0.04-0.39)
**Slovenia**	**All leukemia**	Benzene	0.3 (0.06-0.54)	0.21 (0.04-0.39)
**Slovenia**	**All leukemia**	Formaldehyde	0.05 (0.04-0.06)	0.18 (0.03-0.32)
**Slovenia**	**Acute myeloid leukemia**	Benzene	0.4 (0.07-0.7)	1.66 (0.49-2.73)
**Slovenia**	**Acute myeloid leukemia**	Formaldehyde	0.06 (0.05-0.08)	0.36 (0.07-0.65)
**Slovenia**	**Acute lymphoid leukemia**	Benzene	0.49 (0.09-0.89)	0.31 (0.06-0.54)
**Slovenia**	**Acute lymphoid leukemia**	Formaldehyde	0.08 (0.06-0.1)	0.31 (0.06-0.55)
**Slovenia**	**Chronic myeloid leukemia**	Benzene	0.41 (0.08-0.74)	0.37 (0.07-0.68)
**Slovenia**	**Chronic myeloid leukemia**	Formaldehyde	0.07 (0.06-0.08)	0.49 (0.13-0.86)
**Slovenia**	**Chronic lymphoid leukemia**	Benzene	0.15 (0.03-0.26)	0.86 (0.24-1.51)
**Slovenia**	**Chronic lymphoid leukemia**	Formaldehyde	0.02 (0.02-0.03)	0.77 (0.23-1.27)
**Slovenia**	**Other leukemia**	Benzene	0.26 (0.05-0.45)	0.77 (0.22-1.28)
**Slovenia**	**Other leukemia**	Formaldehyde	0.04 (0.03-0.05)	0.45 (0.13-0.78)
**Solomon Islands**	**All leukemia**	Benzene	0.47 (0.14-0.86)	0.2 (0.04-0.38)
**Solomon Islands**	**All leukemia**	Formaldehyde	0.17 (0.11-0.24)	0.53 (0.15-0.88)
**Solomon Islands**	**Acute myeloid leukemia**	Benzene	0.55 (0.16-0.98)	1.56 (0.44-2.67)
**Solomon Islands**	**Acute myeloid leukemia**	Formaldehyde	0.2 (0.13-0.28)	0.45 (0.14-0.78)
**Solomon Islands**	**Acute lymphoid leukemia**	Benzene	0.33 (0.09-0.65)	0.23 (0.04-0.42)
**Solomon Islands**	**Acute lymphoid leukemia**	Formaldehyde	0.12 (0.07-0.22)	1.01 (0.29-1.69)
**Solomon Islands**	**Chronic myeloid leukemia**	Benzene	0.59 (0.16-1.13)	0.47 (0.14-0.81)
**Solomon Islands**	**Chronic myeloid leukemia**	Formaldehyde	0.23 (0.11-0.36)	0.46 (0.09-0.82)
**Solomon Islands**	**Chronic lymphoid leukemia**	Benzene	0.39 (0.11-0.72)	0.31 (0.06-0.57)
**Solomon Islands**	**Chronic lymphoid leukemia**	Formaldehyde	0.15 (0.1-0.21)	0.19 (0.04-0.35)
**Solomon Islands**	**Other leukemia**	Benzene	0.44 (0.13-0.81)	1.32 (0.4-2.35)
**Solomon Islands**	**Other leukemia**	Formaldehyde	0.16 (0.1-0.23)	0.38 (0.11-0.68)
**Somalia**	**All leukemia**	Benzene	0.3 (0.09-0.53)	0.54 (0.15-0.94)
**Somalia**	**All leukemia**	Formaldehyde	0.1 (0.07-0.14)	0.26 (0.07-0.51)
**Somalia**	**Acute myeloid leukemia**	Benzene	0.43 (0.12-0.87)	0.18 (0.03-0.33)
**Somalia**	**Acute myeloid leukemia**	Formaldehyde	0.15 (0.08-0.25)	0.57 (0.16-0.95)
**Somalia**	**Acute lymphoid leukemia**	Benzene	0.2 (0.05-0.44)	0.55 (0.15-0.95)
**Somalia**	**Acute lymphoid leukemia**	Formaldehyde	0.07 (0.03-0.13)	1 (0.29-1.65)

Countries	Causes	Occupational carcinogens	Percent change in 1990 (95% UI)	Percent change in 2019 (95% UI)
**Somalia**	**Chronic myeloid leukemia**	Benzene	0.51 (0.12-1.03)	0.27 (0.05-0.48)
**Somalia**	**Chronic myeloid leukemia**	Formaldehyde	0.18 (0.08-0.28)	0.27 (0.05-0.48)
**Somalia**	**Chronic lymphoid leukemia**	Benzene	0.26 (0.07-0.47)	0.45 (0.13-0.76)
**Somalia**	**Chronic lymphoid leukemia**	Formaldehyde	0.09 (0.07-0.12)	0.54 (0.15-0.91)
**Somalia**	**Other leukemia**	Benzene	0.27 (0.08-0.51)	0.65 (0.19-1.11)
**Somalia**	**Other leukemia**	Formaldehyde	0.1 (0.06-0.14)	0.67 (0.2-1.13)
**South Africa**	**All leukemia**	Benzene	0.88 (0.26-1.47)	0.58 (0.17-1.01)
**South Africa**	**All leukemia**	Formaldehyde	0.3 (0.24-0.38)	0.67 (0.2-1.13)
**South Africa**	**Acute myeloid leukemia**	Benzene	1.3 (0.38-2.19)	0.7 (0.21-1.19)
**South Africa**	**Acute myeloid leukemia**	Formaldehyde	0.46 (0.36-0.59)	0.42 (0.12-0.71)
**South Africa**	**Acute lymphoid leukemia**	Benzene	0.78 (0.22-1.37)	0.82 (0.24-1.37)
**South Africa**	**Acute lymphoid leukemia**	Formaldehyde	0.28 (0.18-0.41)	0.31 (0.08-0.53)
**South Africa**	**Chronic myeloid leukemia**	Benzene	1.15 (0.34-1.91)	0.4 (0.12-0.66)
**South Africa**	**Chronic myeloid leukemia**	Formaldehyde	0.41 (0.32-0.53)	0.81 (0.24-1.39)
**South Africa**	**Chronic lymphoid leukemia**	Benzene	0.61 (0.17-1.04)	0.81 (0.24-1.36)
**South Africa**	**Chronic lymphoid leukemia**	Formaldehyde	0.18 (0.13-0.23)	0.41 (0.11-0.71)
**South Africa**	**Other leukemia**	Benzene	0.96 (0.28-1.61)	0.37 (0.07-0.67)
**South Africa**	**Other leukemia**	Formaldehyde	0.34 (0.27-0.43)	1.62 (0.48-2.91)
**South Sudan**	**All leukemia**	Benzene	0.21 (0.05-0.43)	0.26 (0.05-0.46)
**South Sudan**	**All leukemia**	Formaldehyde	0.08 (0.04-0.13)	0.4 (0.12-0.72)
**South Sudan**	**Acute myeloid leukemia**	Benzene	0.34 (0.09-0.73)	0.27 (0.05-0.48)
**South Sudan**	**Acute myeloid leukemia**	Formaldehyde	0.13 (0.06-0.23)	0.7 (0.2-1.16)
**South Sudan**	**Acute lymphoid leukemia**	Benzene	0.15 (0.03-0.39)	0.66 (0.2-1.12)
**South Sudan**	**Acute lymphoid leukemia**	Formaldehyde	0.06 (0.02-0.13)	1.19 (0.35-1.96)
**South Sudan**	**Chronic myeloid leukemia**	Benzene	0.36 (0.07-0.86)	0.88 (0.27-1.46)
**South Sudan**	**Chronic myeloid leukemia**	Formaldehyde	0.14 (0.04-0.27)	0.85 (0.25-1.41)
**South Sudan**	**Chronic lymphoid leukemia**	Benzene	0.2 (0.06-0.34)	0.66 (0.2-1.09)
**South Sudan**	**Chronic lymphoid leukemia**	Formaldehyde	0.08 (0.06-0.1)	0.43 (0.12-0.77)
**South Sudan**	**Other leukemia**	Benzene	0.18 (0.05-0.39)	0.63 (0.18-1.15)
**South Sudan**	**Other leukemia**	Formaldehyde	0.07 (0.03-0.13)	0.6 (0.17-1.01)
**Spain**	**All leukemia**	Benzene	0.27 (0.05-0.48)	0.18 (0.13-0.24)
**Spain**	**All leukemia**	Formaldehyde	0.04 (0.03-0.05)	0.03 (0.02-0.04)
**Spain**	**Acute myeloid leukemia**	Benzene	0.32 (0.06-0.58)	0.15 (0.12-0.18)
**Spain**	**Acute myeloid leukemia**	Formaldehyde	0.05 (0.04-0.05)	0.28 (0.23-0.36)
**Spain**	**Acute lymphoid leukemia**	Benzene	0.35 (0.07-0.64)	0.04 (0.03-0.05)
**Spain**	**Acute lymphoid leukemia**	Formaldehyde	0.05 (0.04-0.06)	0.25 (0.18-0.33)
**Spain**	**Chronic myeloid leukemia**	Benzene	0.32 (0.06-0.58)	0.23 (0.19-0.27)
**Spain**	**Chronic myeloid leukemia**	Formaldehyde	0.05 (0.04-0.06)	0.24 (0.2-0.29)
**Spain**	**Chronic lymphoid leukemia**	Benzene	0.14 (0.02-0.24)	0.19 (0.16-0.22)
**Spain**	**Chronic lymphoid leukemia**	Formaldehyde	0.02 (0.02-0.02)	0.03 (0.03-0.04)
**Spain**	**Other leukemia**	Benzene	0.26 (0.05-0.46)	0.03 (0.03-0.04)
**Spain**	**Other leukemia**	Formaldehyde	0.04 (0.03-0.04)	0.25 (0.2-0.31)
**Sri Lanka**	**All leukemia**	Benzene	0.45 (0.13-0.75)	0.28 (0.24-0.33)
**Sri Lanka**	**All leukemia**	Formaldehyde	0.19 (0.15-0.23)	0.37 (0.29-0.48)

Countries	Causes	Occupational carcinogens	Percent change in 1990 (95% UI)	Percent change in 2019 (95% UI)
**Sri Lanka**	**Acute myeloid leukemia**	Benzene	0.52 (0.15-0.86)	0.22 (0.17-0.29)
**Sri Lanka**	**Acute myeloid leukemia**	Formaldehyde	0.22 (0.18-0.27)	0.21 (0.17-0.24)
**Sri Lanka**	**Acute lymphoid leukemia**	Benzene	0.42 (0.12-0.72)	0.05 (0.04-0.06)
**Sri Lanka**	**Acute lymphoid leukemia**	Formaldehyde	0.17 (0.12-0.23)	0.02 (0.02-0.03)
**Sri Lanka**	**Chronic myeloid leukemia**	Benzene	0.62 (0.18-1.05)	0.35 (0.29-0.42)
**Sri Lanka**	**Chronic myeloid leukemia**	Formaldehyde	0.26 (0.21-0.33)	0.24 (0.17-0.31)
**Sri Lanka**	**Chronic lymphoid leukemia**	Benzene	0.36 (0.1-0.6)	0.15 (0.13-0.18)
**Sri Lanka**	**Chronic lymphoid leukemia**	Formaldehyde	0.15 (0.12-0.19)	0.19 (0.15-0.24)
**Sri Lanka**	**Other leukemia**	Benzene	0.42 (0.12-0.72)	0.3 (0.24-0.36)
**Sri Lanka**	**Other leukemia**	Formaldehyde	0.18 (0.15-0.22)	0.03 (0.02-0.03)
**Sudan**	**All leukemia**	Benzene	0.34 (0.1-0.63)	0.19 (0.14-0.25)
**Sudan**	**All leukemia**	Formaldehyde	0.13 (0.08-0.19)	0.24 (0.2-0.28)
**Sudan**	**Acute myeloid leukemia**	Benzene	0.51 (0.14-0.94)	0.08 (0.06-0.09)
**Sudan**	**Acute myeloid leukemia**	Formaldehyde	0.19 (0.12-0.27)	0.05 (0.04-0.06)
**Sudan**	**Acute lymphoid leukemia**	Benzene	0.34 (0.09-0.68)	0.11 (0.08-0.15)
**Sudan**	**Acute lymphoid leukemia**	Formaldehyde	0.13 (0.07-0.21)	0.19 (0.14-0.24)
**Sudan**	**Chronic myeloid leukemia**	Benzene	0.43 (0.12-0.82)	0.14 (0.11-0.18)
**Sudan**	**Chronic myeloid leukemia**	Formaldehyde	0.16 (0.08-0.24)	0.32 (0.26-0.39)
**Sudan**	**Chronic lymphoid leukemia**	Benzene	0.47 (0.14-0.83)	0.22 (0.18-0.28)
**Sudan**	**Chronic lymphoid leukemia**	Formaldehyde	0.17 (0.13-0.24)	0.03 (0.03-0.04)
**Sudan**	**Other leukemia**	Benzene	0.29 (0.08-0.53)	0.16 (0.13-0.21)
**Sudan**	**Other leukemia**	Formaldehyde	0.11 (0.06-0.15)	0.12 (0.08-0.17)
**Suriname**	**All leukemia**	Benzene	0.72 (0.21-1.19)	0.22 (0.19-0.26)
**Suriname**	**All leukemia**	Formaldehyde	0.2 (0.16-0.24)	0.31 (0.26-0.37)
**Suriname**	**Acute myeloid leukemia**	Benzene	0.87 (0.26-1.43)	0.33 (0.27-0.39)
**Suriname**	**Acute myeloid leukemia**	Formaldehyde	0.24 (0.19-0.32)	0.21 (0.15-0.27)
**Suriname**	**Acute lymphoid leukemia**	Benzene	0.45 (0.13-0.8)	0.23 (0.18-0.29)
**Suriname**	**Acute lymphoid leukemia**	Formaldehyde	0.12 (0.09-0.18)	0.22 (0.17-0.27)
**Suriname**	**Chronic myeloid leukemia**	Benzene	0.93 (0.27-1.55)	0.27 (0.22-0.31)
**Suriname**	**Chronic myeloid leukemia**	Formaldehyde	0.25 (0.2-0.32)	0.02 (0.02-0.03)
**Suriname**	**Chronic lymphoid leukemia**	Benzene	0.48 (0.14-0.81)	0.23 (0.19-0.27)
**Suriname**	**Chronic lymphoid leukemia**	Formaldehyde	0.13 (0.1-0.16)	0.03 (0.02-0.03)
**Suriname**	**Other leukemia**	Benzene	0.73 (0.22-1.21)	0.04 (0.03-0.05)
**Suriname**	**Other leukemia**	Formaldehyde	0.2 (0.16-0.24)	0.22 (0.16-0.28)
**Sweden**	**All leukemia**	Benzene	0.41 (0.08-0.72)	0.37 (0.29-0.48)
**Sweden**	**All leukemia**	Formaldehyde	0.04 (0.04-0.05)	0.23 (0.18-0.29)
**Sweden**	**Acute myeloid leukemia**	Benzene	0.49 (0.09-0.88)	0.03 (0.03-0.04)
**Sweden**	**Acute myeloid leukemia**	Formaldehyde	0.05 (0.04-0.06)	0.14 (0.1-0.19)
**Sweden**	**Acute lymphoid leukemia**	Benzene	0.74 (0.14-1.33)	0.19 (0.16-0.23)
**Sweden**	**Acute lymphoid leukemia**	Formaldehyde	0.08 (0.07-0.1)	0.32 (0.25-0.4)
**Sweden**	**Chronic myeloid leukemia**	Benzene	0.56 (0.11-1.01)	0.34 (0.28-0.42)
**Sweden**	**Chronic myeloid leukemia**	Formaldehyde	0.06 (0.05-0.07)	0.24 (0.17-0.31)
**Sweden**	**Chronic lymphoid leukemia**	Benzene	0.2 (0.04-0.35)	0.34 (0.28-0.41)
**Sweden**	**Chronic lymphoid leukemia**	Formaldehyde	0.02 (0.02-0.03)	0.2 (0.15-0.25)

Countries	Causes	Occupational carcinogens	Percent change in 1990 (95% UI)	Percent change in 2019 (95% UI)
**Sweden**	**Other leukemia**	Benzene	0.34 (0.06-0.61)	0.22 (0.16-0.29)
**Sweden**	**Other leukemia**	Formaldehyde	0.04 (0.03-0.04)	0.03 (0.02-0.04)
**Switzerland**	**All leukemia**	Benzene	0.46 (0.09-0.82)	0.17 (0.13-0.22)
**Switzerland**	**All leukemia**	Formaldehyde	0.06 (0.05-0.07)	0.2 (0.14-0.26)
**Switzerland**	**Acute myeloid leukemia**	Benzene	0.6 (0.11-1.06)	0.23 (0.19-0.28)
**Switzerland**	**Acute myeloid leukemia**	Formaldehyde	0.08 (0.06-0.09)	0.03 (0.02-0.04)
**Switzerland**	**Acute lymphoid leukemia**	Benzene	0.77 (0.14-1.36)	0.02 (0.02-0.03)
**Switzerland**	**Acute lymphoid leukemia**	Formaldehyde	0.1 (0.08-0.13)	0.14 (0.11-0.18)
**Switzerland**	**Chronic myeloid leukemia**	Benzene	0.56 (0.11-1.02)	0.21 (0.16-0.26)
**Switzerland**	**Chronic myeloid leukemia**	Formaldehyde	0.07 (0.06-0.09)	0.18 (0.15-0.21)
**Switzerland**	**Chronic lymphoid leukemia**	Benzene	0.19 (0.03-0.33)	0.03 (0.03-0.04)
**Switzerland**	**Chronic lymphoid leukemia**	Formaldehyde	0.02 (0.02-0.03)	0.31 (0.25-0.39)
**Switzerland**	**Other leukemia**	Benzene	0.38 (0.08-0.68)	0.02 (0.01-0.02)
**Switzerland**	**Other leukemia**	Formaldehyde	0.05 (0.04-0.06)	0.03 (0.03-0.04)
**Syrian Arab Republic**	**All leukemia**	Benzene	0.42 (0.12-0.7)	0.26 (0.22-0.32)
**Syrian Arab Republic**	**All leukemia**	Formaldehyde	0.14 (0.11-0.19)	0.2 (0.16-0.24)
**Syrian Arab Republic**	**Acute myeloid leukemia**	Benzene	0.42 (0.12-0.73)	0.38 (0.32-0.46)
**Syrian Arab Republic**	**Acute myeloid leukemia**	Formaldehyde	0.14 (0.1-0.2)	0.14 (0.11-0.18)
**Syrian Arab Republic**	**Acute lymphoid leukemia**	Benzene	0.32 (0.09-0.57)	0.22 (0.17-0.28)
**Syrian Arab Republic**	**Acute lymphoid leukemia**	Formaldehyde	0.11 (0.07-0.16)	0.3 (0.24-0.36)
**Syrian Arab Republic**	**Chronic myeloid leukemia**	Benzene	0.49 (0.15-0.86)	0.18 (0.13-0.23)
**Syrian Arab Republic**	**Chronic myeloid leukemia**	Formaldehyde	0.17 (0.11-0.23)	0.31 (0.24-0.4)
**Syrian Arab Republic**	**Chronic lymphoid leukemia**	Benzene	0.33 (0.1-0.59)	0.04 (0.03-0.05)
**Syrian Arab Republic**	**Chronic lymphoid leukemia**	Formaldehyde	0.11 (0.08-0.16)	0.04 (0.04-0.05)
**Syrian Arab Republic**	**Other leukemia**	Benzene	0.43 (0.12-0.72)	0.2 (0.17-0.25)
**Syrian Arab Republic**	**Other leukemia**	Formaldehyde	0.15 (0.11-0.19)	0.28 (0.24-0.34)
**Taiwan (Province of China)**	**All leukemia**	Benzene	0.7 (0.21-1.16)	0.19 (0.15-0.23)
**Taiwan (Province of China)**	**All leukemia**	Formaldehyde	0.37 (0.31-0.45)	0.17 (0.14-0.22)
**Taiwan (Province of China)**	**Acute myeloid leukemia**	Benzene	0.86 (0.25-1.44)	0.03 (0.03-0.04)
**Taiwan (Province of China)**	**Acute myeloid leukemia**	Formaldehyde	0.46 (0.38-0.56)	0.04 (0.03-0.04)
**Taiwan (Province of China)**	**Acute lymphoid leukemia**	Benzene	0.73 (0.22-1.21)	0.02 (0.02-0.03)
**Taiwan (Province of China)**	**Acute lymphoid leukemia**	Formaldehyde	0.38 (0.31-0.48)	0.29 (0.24-0.35)
**Taiwan (Province of China)**	**Chronic myeloid leukemia**	Benzene	0.76 (0.22-1.26)	0.04 (0.03-0.04)
**Taiwan (Province of China)**	**Chronic myeloid leukemia**	Formaldehyde	0.4 (0.34-0.49)	0.14 (0.11-0.18)
**Taiwan (Province of China)**	**Chronic lymphoid leukemia**	Benzene	0.36 (0.11-0.62)	0.25 (0.21-0.29)
**Taiwan (Province of China)**	**Chronic lymphoid leukemia**	Formaldehyde	0.19 (0.16-0.24)	0.33 (0.27-0.41)
**Taiwan (Province of China)**	**Other leukemia**	Benzene	0.68 (0.2-1.12)	0.23 (0.18-0.29)
**Taiwan (Province of China)**	**Other leukemia**	Formaldehyde	0.36 (0.3-0.43)	0.29 (0.24-0.35)
**Tajikistan**	**All leukemia**	Benzene	0.39 (0.11-0.66)	0.25 (0.21-0.31)
**Tajikistan**	**All leukemia**	Formaldehyde	0.12 (0.09-0.16)	0.37 (0.29-0.46)
**Tajikistan**	**Acute myeloid leukemia**	Benzene	0.47 (0.14-0.81)	0.03 (0.02-0.03)
**Tajikistan**	**Acute myeloid leukemia**	Formaldehyde	0.15 (0.1-0.21)	0.17 (0.13-0.21)
**Tajikistan**	**Acute lymphoid leukemia**	Benzene	0.26 (0.07-0.47)	0.17 (0.13-0.22)
**Tajikistan**	**Acute lymphoid leukemia**	Formaldehyde	0.08 (0.05-0.11)	0.25 (0.19-0.3)

Countries	Causes	Occupational carcinogens	Percent change in 1990 (95% UI)	Percent change in 2019 (95% UI)
**Tajikistan**	**Chronic myeloid leukemia**	Benzene	0.5 (0.14-0.9)	0.17 (0.14-0.22)
**Tajikistan**	**Chronic myeloid leukemia**	Formaldehyde	0.15 (0.1-0.22)	0.03 (0.02-0.03)
**Tajikistan**	**Chronic lymphoid leukemia**	Benzene	0.5 (0.14-0.85)	0.03 (0.02-0.03)
**Tajikistan**	**Chronic lymphoid leukemia**	Formaldehyde	0.15 (0.11-0.2)	0.21 (0.16-0.26)
**Tajikistan**	**Other leukemia**	Benzene	0.4 (0.11-0.7)	0.29 (0.21-0.36)
**Tajikistan**	**Other leukemia**	Formaldehyde	0.12 (0.08-0.17)	0.36 (0.3-0.43)
**Thailand**	**All leukemia**	Benzene	0.68 (0.2-1.14)	0.3 (0.23-0.42)
**Thailand**	**All leukemia**	Formaldehyde	0.28 (0.22-0.35)	0.16 (0.12-0.2)
**Thailand**	**Acute myeloid leukemia**	Benzene	0.75 (0.22-1.27)	0.03 (0.03-0.04)
**Thailand**	**Acute myeloid leukemia**	Formaldehyde	0.32 (0.24-0.41)	0.2 (0.16-0.26)
**Thailand**	**Acute lymphoid leukemia**	Benzene	0.59 (0.16-1.06)	0.13 (0.09-0.16)
**Thailand**	**Acute lymphoid leukemia**	Formaldehyde	0.25 (0.16-0.36)	0.29 (0.24-0.34)
**Thailand**	**Chronic myeloid leukemia**	Benzene	0.84 (0.24-1.4)	0.38 (0.31-0.45)
**Thailand**	**Chronic myeloid leukemia**	Formaldehyde	0.35 (0.27-0.45)	0.31 (0.22-0.4)
**Thailand**	**Chronic lymphoid leukemia**	Benzene	0.31 (0.09-0.51)	0.03 (0.02-0.03)
**Thailand**	**Chronic lymphoid leukemia**	Formaldehyde	0.13 (0.1-0.16)	0.28 (0.23-0.35)
**Thailand**	**Other leukemia**	Benzene	0.68 (0.2-1.13)	0.03 (0.03-0.04)
**Thailand**	**Other leukemia**	Formaldehyde	0.28 (0.22-0.35)	0.18 (0.14-0.22)
**Timor-Leste**	**All leukemia**	Benzene	0.34 (0.09-0.65)	0.12 (0.06-0.19)
**Timor-Leste**	**All leukemia**	Formaldehyde	0.14 (0.08-0.21)	0.23 (0.19-0.28)
**Timor-Leste**	**Acute myeloid leukemia**	Benzene	0.48 (0.13-0.88)	0.11 (0.09-0.14)
**Timor-Leste**	**Acute myeloid leukemia**	Formaldehyde	0.19 (0.12-0.28)	0.38 (0.29-0.47)
**Timor-Leste**	**Acute lymphoid leukemia**	Benzene	0.24 (0.06-0.55)	0.23 (0.18-0.28)
**Timor-Leste**	**Acute lymphoid leukemia**	Formaldehyde	0.1 (0.05-0.18)	0.03 (0.02-0.04)
**Timor-Leste**	**Chronic myeloid leukemia**	Benzene	0.48 (0.13-0.92)	0.04 (0.03-0.04)
**Timor-Leste**	**Chronic myeloid leukemia**	Formaldehyde	0.2 (0.1-0.29)	0.3 (0.25-0.36)
**Timor-Leste**	**Chronic lymphoid leukemia**	Benzene	0.39 (0.11-0.67)	0.13 (0.09-0.19)
**Timor-Leste**	**Chronic lymphoid leukemia**	Formaldehyde	0.16 (0.12-0.21)	0.15 (0.11-0.19)
**Timor-Leste**	**Other leukemia**	Benzene	0.34 (0.09-0.69)	0.17 (0.13-0.22)
**Timor-Leste**	**Other leukemia**	Formaldehyde	0.14 (0.08-0.22)	0.04 (0.03-0.05)
**Togo**	**All leukemia**	Benzene	0.5 (0.15-0.86)	0.3 (0.24-0.37)
**Togo**	**All leukemia**	Formaldehyde	0.18 (0.13-0.23)	0.03 (0.03-0.04)
**Togo**	**Acute myeloid leukemia**	Benzene	0.42 (0.11-0.8)	0.26 (0.19-0.35)
**Togo**	**Acute myeloid leukemia**	Formaldehyde	0.15 (0.08-0.22)	0.21 (0.16-0.27)
**Togo**	**Acute lymphoid leukemia**	Benzene	0.35 (0.1-0.63)	0.25 (0.2-0.31)
**Togo**	**Acute lymphoid leukemia**	Formaldehyde	0.13 (0.07-0.21)	0.16 (0.13-0.2)
**Togo**	**Chronic myeloid leukemia**	Benzene	1.09 (0.31-1.82)	0.27 (0.22-0.32)
**Togo**	**Chronic myeloid leukemia**	Formaldehyde	0.39 (0.29-0.52)	0.15 (0.11-0.19)
**Togo**	**Chronic lymphoid leukemia**	Benzene	0.26 (0.07-0.44)	0.31 (0.26-0.37)
**Togo**	**Chronic lymphoid leukemia**	Formaldehyde	0.09 (0.07-0.12)	0.37 (0.31-0.43)
**Togo**	**Other leukemia**	Benzene	0.46 (0.13-0.78)	0.31 (0.25-0.38)
**Togo**	**Other leukemia**	Formaldehyde	0.16 (0.12-0.21)	0.04 (0.03-0.04)
**Tokelau**	**All leukemia**	Benzene	0.55 (0.16-0.93)	0.03 (0.03-0.04)
**Tokelau**	**All leukemia**	Formaldehyde	0.2 (0.15-0.27)	0.02 (0.02-0.03)

Countries	Causes	Occupational carcinogens	Percent change in 1990 (95% UI)	Percent change in 2019 (95% UI)
**Tokelau**	**Acute myeloid leukemia**	Benzene	0.73 (0.22-1.25)	0.47 (0.36-0.61)
**Tokelau**	**Acute myeloid leukemia**	Formaldehyde	0.27 (0.19-0.37)	0.06 (0.05-0.07)
**Tokelau**	**Acute lymphoid leukemia**	Benzene	0.63 (0.19-1.1)	0.03 (0.02-0.03)
**Tokelau**	**Acute lymphoid leukemia**	Formaldehyde	0.23 (0.15-0.34)	0.04 (0.04-0.05)
**Tokelau**	**Chronic myeloid leukemia**	Benzene	0.64 (0.18-1.11)	0.04 (0.03-0.05)
**Tokelau**	**Chronic myeloid leukemia**	Formaldehyde	0.26 (0.17-0.37)	0.16 (0.11-0.2)
**Tokelau**	**Chronic lymphoid leukemia**	Benzene	0.44 (0.13-0.76)	0.23 (0.16-0.3)
**Tokelau**	**Chronic lymphoid leukemia**	Formaldehyde	0.17 (0.12-0.23)	0.24 (0.2-0.28)
**Tokelau**	**Other leukemia**	Benzene	0.44 (0.13-0.74)	0.23 (0.19-0.27)
**Tokelau**	**Other leukemia**	Formaldehyde	0.16 (0.11-0.21)	0.16 (0.12-0.2)
**Tonga**	**All leukemia**	Benzene	0.47 (0.14-0.77)	0.03 (0.02-0.04)
**Tonga**	**All leukemia**	Formaldehyde	0.15 (0.12-0.18)	0.15 (0.12-0.19)
**Tonga**	**Acute myeloid leukemia**	Benzene	0.6 (0.17-1.01)	0.36 (0.28-0.46)
**Tonga**	**Acute myeloid leukemia**	Formaldehyde	0.19 (0.15-0.25)	0.18 (0.13-0.23)
**Tonga**	**Acute lymphoid leukemia**	Benzene	0.45 (0.13-0.8)	0.04 (0.03-0.04)
**Tonga**	**Acute lymphoid leukemia**	Formaldehyde	0.14 (0.1-0.2)	0.3 (0.25-0.36)
**Tonga**	**Chronic myeloid leukemia**	Benzene	0.53 (0.16-0.89)	0.13 (0.09-0.17)
**Tonga**	**Chronic myeloid leukemia**	Formaldehyde	0.18 (0.14-0.24)	0.06 (0.05-0.07)
**Tonga**	**Chronic lymphoid leukemia**	Benzene	0.44 (0.12-0.76)	0.04 (0.04-0.05)
**Tonga**	**Chronic lymphoid leukemia**	Formaldehyde	0.15 (0.11-0.19)	0.03 (0.03-0.04)
**Tonga**	**Other leukemia**	Benzene	0.4 (0.12-0.67)	0.48 (0.32-0.68)
**Tonga**	**Other leukemia**	Formaldehyde	0.12 (0.1-0.16)	0.14 (0.1-0.18)
**Trinidad and Tobago**	**All leukemia**	Benzene	0.64 (0.19-1.05)	0.15 (0.12-0.21)
**Trinidad and Tobago**	**All leukemia**	Formaldehyde	0.23 (0.19-0.27)	0.11 (0.07-0.15)
**Trinidad and Tobago**	**Acute myeloid leukemia**	Benzene	0.74 (0.21-1.23)	0.02 (0.02-0.03)
**Trinidad and Tobago**	**Acute myeloid leukemia**	Formaldehyde	0.26 (0.21-0.32)	0.26 (0.22-0.32)
**Trinidad and Tobago**	**Acute lymphoid leukemia**	Benzene	0.42 (0.12-0.72)	0.21 (0.16-0.27)
**Trinidad and Tobago**	**Acute lymphoid leukemia**	Formaldehyde	0.15 (0.11-0.19)	0.26 (0.21-0.3)
**Trinidad and Tobago**	**Chronic myeloid leukemia**	Benzene	0.8 (0.24-1.32)	0.03 (0.02-0.03)
**Trinidad and Tobago**	**Chronic myeloid leukemia**	Formaldehyde	0.28 (0.23-0.35)	0.04 (0.03-0.04)
**Trinidad and Tobago**	**Chronic lymphoid leukemia**	Benzene	0.46 (0.13-0.76)	0.14 (0.11-0.17)
**Trinidad and Tobago**	**Chronic lymphoid leukemia**	Formaldehyde	0.17 (0.13-0.2)	0.28 (0.23-0.32)
**Trinidad and Tobago**	**Other leukemia**	Benzene	0.68 (0.2-1.11)	0.17 (0.14-0.21)
**Trinidad and Tobago**	**Other leukemia**	Formaldehyde	0.23 (0.19-0.28)	0.27 (0.22-0.33)
**Tunisia**	**All leukemia**	Benzene	0.33 (0.1-0.56)	0.22 (0.14-0.28)
**Tunisia**	**All leukemia**	Formaldehyde	0.14 (0.11-0.18)	0.23 (0.18-0.28)
**Tunisia**	**Acute myeloid leukemia**	Benzene	0.44 (0.12-0.75)	0.22 (0.17-0.27)
**Tunisia**	**Acute myeloid leukemia**	Formaldehyde	0.2 (0.14-0.25)	0.17 (0.13-0.21)
**Tunisia**	**Acute lymphoid leukemia**	Benzene	0.3 (0.09-0.56)	0.28 (0.24-0.34)
**Tunisia**	**Acute lymphoid leukemia**	Formaldehyde	0.13 (0.09-0.19)	0.15 (0.13-0.19)
**Tunisia**	**Chronic myeloid leukemia**	Benzene	0.42 (0.12-0.74)	0.21 (0.17-0.26)
**Tunisia**	**Chronic myeloid leukemia**	Formaldehyde	0.19 (0.13-0.25)	0.22 (0.18-0.27)
**Tunisia**	**Chronic lymphoid leukemia**	Benzene	0.32 (0.09-0.55)	0.26 (0.21-0.32)
**Tunisia**	**Chronic lymphoid leukemia**	Formaldehyde	0.14 (0.11-0.18)	0.15 (0.11-0.19)

Countries	Causes	Occupational carcinogens	Percent change in 1990 (95% UI)	Percent change in 2019 (95% UI)
**Tunisia**	**Other leukemia**	Benzene	0.24 (0.07-0.42)	0.04 (0.03-0.05)
**Tunisia**	**Other leukemia**	Formaldehyde	0.1 (0.08-0.14)	0.49 (0.35-0.68)
**Turkey**	**All leukemia**	Benzene	0.46 (0.14-0.8)	0.03 (0.03-0.04)
**Turkey**	**All leukemia**	Formaldehyde	0.21 (0.16-0.26)	0.15 (0.11-0.2)
**Turkey**	**Acute myeloid leukemia**	Benzene	0.59 (0.17-1.03)	0.04 (0.03-0.04)
**Turkey**	**Acute myeloid leukemia**	Formaldehyde	0.27 (0.2-0.34)	0.18 (0.15-0.22)
**Turkey**	**Acute lymphoid leukemia**	Benzene	0.43 (0.12-0.79)	0.2 (0.16-0.23)
**Turkey**	**Acute lymphoid leukemia**	Formaldehyde	0.19 (0.13-0.27)	0.27 (0.22-0.33)
**Turkey**	**Chronic myeloid leukemia**	Benzene	0.52 (0.16-0.93)	0.26 (0.2-0.33)
**Turkey**	**Chronic myeloid leukemia**	Formaldehyde	0.23 (0.17-0.32)	0.28 (0.24-0.34)
**Turkey**	**Chronic lymphoid leukemia**	Benzene	0.38 (0.11-0.66)	0.3 (0.24-0.37)
**Turkey**	**Chronic lymphoid leukemia**	Formaldehyde	0.17 (0.12-0.23)	0.15 (0.11-0.21)
**Turkey**	**Other leukemia**	Benzene	0.37 (0.11-0.63)	0.21 (0.14-0.28)
**Turkey**	**Other leukemia**	Formaldehyde	0.17 (0.12-0.22)	0.21 (0.16-0.27)
**Turkmenistan**	**All leukemia**	Benzene	0.36 (0.1-0.61)	0.16 (0.11-0.22)
**Turkmenistan**	**All leukemia**	Formaldehyde	0.12 (0.09-0.15)	0.03 (0.02-0.04)
**Turkmenistan**	**Acute myeloid leukemia**	Benzene	0.46 (0.13-0.81)	0.14 (0.11-0.18)
**Turkmenistan**	**Acute myeloid leukemia**	Formaldehyde	0.15 (0.1-0.21)	0.23 (0.18-0.29)
**Turkmenistan**	**Acute lymphoid leukemia**	Benzene	0.27 (0.08-0.49)	0.03 (0.02-0.04)
**Turkmenistan**	**Acute lymphoid leukemia**	Formaldehyde	0.09 (0.06-0.12)	0.24 (0.17-0.34)
**Turkmenistan**	**Chronic myeloid leukemia**	Benzene	0.51 (0.14-0.88)	0.2 (0.17-0.24)
**Turkmenistan**	**Chronic myeloid leukemia**	Formaldehyde	0.17 (0.12-0.22)	0.21 (0.18-0.25)
**Turkmenistan**	**Chronic lymphoid leukemia**	Benzene	0.5 (0.15-0.85)	0.19 (0.16-0.23)
**Turkmenistan**	**Chronic lymphoid leukemia**	Formaldehyde	0.16 (0.13-0.2)	0.02 (0.02-0.03)
**Turkmenistan**	**Other leukemia**	Benzene	0.36 (0.1-0.6)	0.03 (0.02-0.03)
**Turkmenistan**	**Other leukemia**	Formaldehyde	0.12 (0.09-0.15)	0.25 (0.2-0.31)
**Tuvalu**	**All leukemia**	Benzene	0.64 (0.19-1.08)	0.26 (0.22-0.31)
**Tuvalu**	**All leukemia**	Formaldehyde	0.24 (0.19-0.31)	0.4 (0.28-0.54)
**Tuvalu**	**Acute myeloid leukemia**	Benzene	0.85 (0.24-1.42)	0.2 (0.15-0.26)
**Tuvalu**	**Acute myeloid leukemia**	Formaldehyde	0.32 (0.24-0.42)	0.17 (0.15-0.21)
**Tuvalu**	**Acute lymphoid leukemia**	Benzene	0.58 (0.17-1.02)	0.04 (0.03-0.05)
**Tuvalu**	**Acute lymphoid leukemia**	Formaldehyde	0.21 (0.14-0.3)	0.02 (0.02-0.03)
**Tuvalu**	**Chronic myeloid leukemia**	Benzene	0.77 (0.22-1.36)	0.37 (0.31-0.44)
**Tuvalu**	**Chronic myeloid leukemia**	Formaldehyde	0.31 (0.21-0.43)	0.24 (0.18-0.32)
**Tuvalu**	**Chronic lymphoid leukemia**	Benzene	0.54 (0.15-0.91)	0.12 (0.1-0.15)
**Tuvalu**	**Chronic lymphoid leukemia**	Formaldehyde	0.21 (0.16-0.28)	0.17 (0.12-0.22)
**Tuvalu**	**Other leukemia**	Benzene	0.56 (0.17-0.94)	0.31 (0.25-0.39)
**Tuvalu**	**Other leukemia**	Formaldehyde	0.21 (0.16-0.26)	0.02 (0.02-0.03)
**Uganda**	**All leukemia**	Benzene	0.25 (0.07-0.47)	0.21 (0.15-0.29)
**Uganda**	**All leukemia**	Formaldehyde	0.09 (0.06-0.12)	0.2 (0.17-0.24)
**Uganda**	**Acute myeloid leukemia**	Benzene	0.37 (0.1-0.73)	0.07 (0.06-0.09)
**Uganda**	**Acute myeloid leukemia**	Formaldehyde	0.13 (0.06-0.2)	0.05 (0.04-0.06)
**Uganda**	**Acute lymphoid leukemia**	Benzene	0.2 (0.05-0.43)	0.12 (0.08-0.16)
**Uganda**	**Acute lymphoid leukemia**	Formaldehyde	0.07 (0.03-0.12)	0.18 (0.12-0.24)

Countries	Causes	Occupational carcinogens	Percent change in 1990 (95% UI)	Percent change in 2019 (95% UI)
**Uganda**	**Chronic myeloid leukemia**	Benzene	0.39 (0.09-0.8)	0.14 (0.1-0.2)
**Uganda**	**Chronic myeloid leukemia**	Formaldehyde	0.13 (0.06-0.22)	0.32 (0.26-0.4)
**Uganda**	**Chronic lymphoid leukemia**	Benzene	0.22 (0.06-0.38)	0.24 (0.18-0.31)
**Uganda**	**Chronic lymphoid leukemia**	Formaldehyde	0.08 (0.06-0.1)	0.03 (0.02-0.03)
**Uganda**	**Other leukemia**	Benzene	0.21 (0.05-0.4)	0.17 (0.12-0.22)
**Uganda**	**Other leukemia**	Formaldehyde	0.07 (0.04-0.11)	0.13 (0.09-0.18)
**Ukraine**	**All leukemia**	Benzene	0.44 (0.08-0.8)	0.18 (0.15-0.22)
**Ukraine**	**All leukemia**	Formaldehyde	0.04 (0.03-0.05)	0.31 (0.26-0.37)
**Ukraine**	**Acute myeloid leukemia**	Benzene	0.56 (0.1-1)	0.29 (0.24-0.35)
**Ukraine**	**Acute myeloid leukemia**	Formaldehyde	0.05 (0.04-0.06)	0.2 (0.15-0.27)
**Ukraine**	**Acute lymphoid leukemia**	Benzene	0.41 (0.07-0.73)	0.24 (0.18-0.3)
**Ukraine**	**Acute lymphoid leukemia**	Formaldehyde	0.04 (0.03-0.05)	0.16 (0.12-0.2)
**Ukraine**	**Chronic myeloid leukemia**	Benzene	0.5 (0.09-0.89)	0.21 (0.17-0.27)
**Ukraine**	**Chronic myeloid leukemia**	Formaldehyde	0.05 (0.04-0.06)	0.02 (0.02-0.03)
**Ukraine**	**Chronic lymphoid leukemia**	Benzene	0.27 (0.05-0.49)	0.18 (0.15-0.22)
**Ukraine**	**Chronic lymphoid leukemia**	Formaldehyde	0.03 (0.02-0.03)	0.02 (0.02-0.03)
**Ukraine**	**Other leukemia**	Benzene	0.42 (0.07-0.77)	0.03 (0.02-0.04)
**Ukraine**	**Other leukemia**	Formaldehyde	0.04 (0.03-0.05)	0.23 (0.17-0.3)
**United Arab Emirates**	**All leukemia**	Benzene	1.24 (0.35-2.15)	0.38 (0.3-0.49)
**United Arab Emirates**	**All leukemia**	Formaldehyde	0.38 (0.28-0.55)	0.24 (0.18-0.31)
**United Arab Emirates**	**Acute myeloid leukemia**	Benzene	1.44 (0.41-2.51)	0.02 (0.02-0.03)
**United Arab Emirates**	**Acute myeloid leukemia**	Formaldehyde	0.45 (0.32-0.67)	0.13 (0.09-0.18)
**United Arab Emirates**	**Acute lymphoid leukemia**	Benzene	1.13 (0.34-2.05)	0.17 (0.13-0.2)
**United Arab Emirates**	**Acute lymphoid leukemia**	Formaldehyde	0.36 (0.23-0.52)	0.32 (0.25-0.39)
**United Arab Emirates**	**Chronic myeloid leukemia**	Benzene	1.4 (0.38-2.54)	0.34 (0.28-0.42)
**United Arab Emirates**	**Chronic myeloid leukemia**	Formaldehyde	0.43 (0.29-0.66)	0.18 (0.12-0.25)
**United Arab Emirates**	**Chronic lymphoid leukemia**	Benzene	0.98 (0.28-1.88)	0.34 (0.28-0.41)
**United Arab Emirates**	**Chronic lymphoid leukemia**	Formaldehyde	0.3 (0.19-0.5)	0.2 (0.15-0.27)
**United Arab Emirates**	**Other leukemia**	Benzene	0.95 (0.27-1.62)	0.22 (0.15-0.28)
**United Arab Emirates**	**Other leukemia**	Formaldehyde	0.29 (0.21-0.4)	0.02 (0.02-0.03)
**United Kingdom**	**All leukemia**	Benzene	0.34 (0.07-0.61)	0.19 (0.14-0.25)
**United Kingdom**	**All leukemia**	Formaldehyde	0.04 (0.03-0.05)	0.15 (0.09-0.24)
**United Kingdom**	**Acute myeloid leukemia**	Benzene	0.39 (0.07-0.71)	0.17 (0.13-0.21)
**United Kingdom**	**Acute myeloid leukemia**	Formaldehyde	0.04 (0.04-0.05)	0.02 (0.01-0.03)
**United Kingdom**	**Acute lymphoid leukemia**	Benzene	0.6 (0.11-1.06)	0.02 (0.01-0.02)
**United Kingdom**	**Acute lymphoid leukemia**	Formaldehyde	0.07 (0.06-0.08)	0.14 (0.11-0.19)
**United Kingdom**	**Chronic myeloid leukemia**	Benzene	0.41 (0.08-0.73)	0.2 (0.15-0.26)
**United Kingdom**	**Chronic myeloid leukemia**	Formaldehyde	0.05 (0.04-0.05)	0.17 (0.14-0.21)
**United Kingdom**	**Chronic lymphoid leukemia**	Benzene	0.17 (0.03-0.31)	0.02 (0.02-0.03)
**United Kingdom**	**Chronic lymphoid leukemia**	Formaldehyde	0.02 (0.02-0.03)	0.29 (0.21-0.39)
**United Kingdom**	**Other leukemia**	Benzene	0.25 (0.05-0.45)	0.01 (0.01-0.01)
**United Kingdom**	**Other leukemia**	Formaldehyde	0.03 (0.03-0.04)	0.03 (0.02-0.04)
**United Republic of Tanzania**	**All leukemia**	Benzene	0.32 (0.09-0.6)	0.24 (0.2-0.29)
**United Republic of Tanzania**	**All leukemia**	Formaldehyde	0.12 (0.08-0.18)	0.15 (0.12-0.19)

Countries	Causes	Occupational carcinogens	Percent change in 1990 (95% UI)	Percent change in 2019 (95% UI)
**United Republic of Tanzania**	**Acute myeloid leukemia**	Benzene	0.48 (0.12-0.98)	0.39 (0.32-0.48)
**United Republic of Tanzania**	**Acute myeloid leukemia**	Formaldehyde	0.19 (0.1-0.3)	0.16 (0.12-0.21)
**United Republic of Tanzania**	**Acute lymphoid leukemia**	Benzene	0.21 (0.05-0.51)	0.22 (0.16-0.29)
**United Republic of Tanzania**	**Acute lymphoid leukemia**	Formaldehyde	0.08 (0.03-0.16)	0.3 (0.24-0.36)
**United Republic of Tanzania**	**Chronic myeloid leukemia**	Benzene	0.57 (0.13-1.2)	0.2 (0.15-0.26)
**United Republic of Tanzania**	**Chronic myeloid leukemia**	Formaldehyde	0.21 (0.09-0.37)	0.31 (0.24-0.4)
**United Republic of Tanzania**	**Chronic lymphoid leukemia**	Benzene	0.33 (0.09-0.56)	0.03 (0.03-0.04)
**United Republic of Tanzania**	**Chronic lymphoid leukemia**	Formaldehyde	0.12 (0.09-0.15)	0.03 (0.02-0.04)
**United Republic of Tanzania**	**Other leukemia**	Benzene	0.28 (0.07-0.54)	0.18 (0.15-0.22)
**United Republic of Tanzania**	**Other leukemia**	Formaldehyde	0.11 (0.06-0.17)	0.28 (0.23-0.33)
**United States of America**	**All leukemia**	Benzene	0.42 (0.08-0.74)	0.12 (0.1-0.15)
**United States of America**	**All leukemia**	Formaldehyde	0.05 (0.04-0.06)	0.16 (0.12-0.2)
**United States of America**	**Acute myeloid leukemia**	Benzene	0.49 (0.09-0.86)	0.02 (0.02-0.03)
**United States of America**	**Acute myeloid leukemia**	Formaldehyde	0.05 (0.05-0.06)	0.03 (0.02-0.04)
**United States of America**	**Acute lymphoid leukemia**	Benzene	0.64 (0.12-1.13)	0.02 (0.01-0.02)
**United States of America**	**Acute lymphoid leukemia**	Formaldehyde	0.07 (0.06-0.09)	0.32 (0.26-0.39)
**United States of America**	**Chronic myeloid leukemia**	Benzene	0.57 (0.11-1.01)	0.03 (0.02-0.03)
**United States of America**	**Chronic myeloid leukemia**	Formaldehyde	0.06 (0.05-0.08)	0.14 (0.11-0.18)
**United States of America**	**Chronic lymphoid leukemia**	Benzene	0.21 (0.04-0.37)	0.16 (0.13-0.21)
**United States of America**	**Chronic lymphoid leukemia**	Formaldehyde	0.02 (0.02-0.03)	0.32 (0.25-0.4)
**United States of America**	**Other leukemia**	Benzene	0.36 (0.07-0.64)	0.22 (0.17-0.28)
**United States of America**	**Other leukemia**	Formaldehyde	0.04 (0.03-0.05)	0.26 (0.21-0.33)
**United States Virgin Islands**	**All leukemia**	Benzene	0.88 (0.26-1.45)	0.24 (0.2-0.3)
**United States Virgin Islands**	**All leukemia**	Formaldehyde	0.26 (0.21-0.31)	0.38 (0.29-0.47)
**United States Virgin Islands**	**Acute myeloid leukemia**	Benzene	1.09 (0.33-1.83)	0.03 (0.03-0.04)
**United States Virgin Islands**	**Acute myeloid leukemia**	Formaldehyde	0.3 (0.24-0.38)	0.11 (0.08-0.15)
**United States Virgin Islands**	**Acute lymphoid leukemia**	Benzene	0.67 (0.19-1.17)	0.2 (0.15-0.26)
**United States Virgin Islands**	**Acute lymphoid leukemia**	Formaldehyde	0.21 (0.15-0.29)	0.25 (0.19-0.33)
**United States Virgin Islands**	**Chronic myeloid leukemia**	Benzene	1.05 (0.32-1.79)	0.13 (0.09-0.18)
**United States Virgin Islands**	**Chronic myeloid leukemia**	Formaldehyde	0.29 (0.22-0.37)	0.02 (0.02-0.03)
**United States Virgin Islands**	**Chronic lymphoid leukemia**	Benzene	0.55 (0.16-0.93)	0.02 (0.02-0.03)
**United States Virgin Islands**	**Chronic lymphoid leukemia**	Formaldehyde	0.17 (0.13-0.2)	0.2 (0.14-0.26)
**United States Virgin Islands**	**Other leukemia**	Benzene	0.87 (0.26-1.45)	0.26 (0.17-0.35)
**United States Virgin Islands**	**Other leukemia**	Formaldehyde	0.27 (0.21-0.33)	0.33 (0.26-0.42)
**Uruguay**	**All leukemia**	Benzene	0.55 (0.16-0.91)	0.21 (0.15-0.28)
**Uruguay**	**All leukemia**	Formaldehyde	0.21 (0.18-0.24)	0.18 (0.14-0.23)
**Uruguay**	**Acute myeloid leukemia**	Benzene	0.66 (0.19-1.1)	0.02 (0.02-0.03)
**Uruguay**	**Acute myeloid leukemia**	Formaldehyde	0.24 (0.2-0.29)	0.18 (0.14-0.24)
**Uruguay**	**Acute lymphoid leukemia**	Benzene	0.59 (0.17-0.98)	0.13 (0.09-0.19)
**Uruguay**	**Acute lymphoid leukemia**	Formaldehyde	0.22 (0.18-0.28)	0.26 (0.22-0.32)
**Uruguay**	**Chronic myeloid leukemia**	Benzene	0.68 (0.2-1.15)	0.35 (0.29-0.42)
**Uruguay**	**Chronic myeloid leukemia**	Formaldehyde	0.26 (0.21-0.32)	0.28 (0.2-0.37)
**Uruguay**	**Chronic lymphoid leukemia**	Benzene	0.24 (0.07-0.4)	0.02 (0.02-0.03)
**Uruguay**	**Chronic lymphoid leukemia**	Formaldehyde	0.09 (0.07-0.12)	0.17 (0.11-0.33)

Countries	Causes	Occupational carcinogens	Percent change in 1990 (95% UI)	Percent change in 2019 (95% UI)
**Uruguay**	**Other leukemia**	Benzene	0.56 (0.17-0.94)	0.02 (0.02-0.03)
**Uruguay**	**Other leukemia**	Formaldehyde	0.21 (0.17-0.25)	0.14 (0.11-0.18)
**Uzbekistan**	**All leukemia**	Benzene	0.64 (0.19-1.07)	0.11 (0.06-0.17)
**Uzbekistan**	**All leukemia**	Formaldehyde	0.17 (0.14-0.21)	0.23 (0.18-0.28)
**Uzbekistan**	**Acute myeloid leukemia**	Benzene	0.76 (0.23-1.3)	0.12 (0.09-0.16)
**Uzbekistan**	**Acute myeloid leukemia**	Formaldehyde	0.2 (0.15-0.26)	0.35 (0.26-0.45)
**Uzbekistan**	**Acute lymphoid leukemia**	Benzene	0.51 (0.16-0.89)	0.21 (0.16-0.26)
**Uzbekistan**	**Acute lymphoid leukemia**	Formaldehyde	0.13 (0.1-0.18)	0.02 (0.02-0.03)
**Uzbekistan**	**Chronic myeloid leukemia**	Benzene	0.76 (0.22-1.29)	0.03 (0.02-0.03)
**Uzbekistan**	**Chronic myeloid leukemia**	Formaldehyde	0.2 (0.15-0.26)	0.3 (0.24-0.37)
**Uzbekistan**	**Chronic lymphoid leukemia**	Benzene	0.63 (0.18-1.04)	0.15 (0.1-0.21)
**Uzbekistan**	**Chronic lymphoid leukemia**	Formaldehyde	0.16 (0.13-0.2)	0.15 (0.12-0.19)
**Uzbekistan**	**Other leukemia**	Benzene	0.64 (0.18-1.07)	0.13 (0.1-0.17)
**Uzbekistan**	**Other leukemia**	Formaldehyde	0.17 (0.13-0.22)	0.05 (0.03-0.06)
**Vanuatu**	**All leukemia**	Benzene	0.74 (0.22-1.25)	0.26 (0.2-0.32)
**Vanuatu**	**All leukemia**	Formaldehyde	0.23 (0.18-0.3)	0.03 (0.02-0.03)
**Vanuatu**	**Acute myeloid leukemia**	Benzene	0.91 (0.27-1.58)	0.18 (0.13-0.26)
**Vanuatu**	**Acute myeloid leukemia**	Formaldehyde	0.29 (0.21-0.38)	0.21 (0.15-0.27)
**Vanuatu**	**Acute lymphoid leukemia**	Benzene	0.65 (0.19-1.2)	0.17 (0.13-0.25)
**Vanuatu**	**Acute lymphoid leukemia**	Formaldehyde	0.2 (0.14-0.29)	0.15 (0.12-0.19)
**Vanuatu**	**Chronic myeloid leukemia**	Benzene	0.92 (0.27-1.6)	0.24 (0.2-0.29)
**Vanuatu**	**Chronic myeloid leukemia**	Formaldehyde	0.29 (0.2-0.41)	0.13 (0.1-0.17)
**Vanuatu**	**Chronic lymphoid leukemia**	Benzene	0.67 (0.19-1.17)	0.28 (0.23-0.35)
**Vanuatu**	**Chronic lymphoid leukemia**	Formaldehyde	0.21 (0.15-0.29)	0.38 (0.31-0.46)
**Vanuatu**	**Other leukemia**	Benzene	0.66 (0.2-1.13)	0.31 (0.25-0.38)
**Vanuatu**	**Other leukemia**	Formaldehyde	0.21 (0.15-0.27)	0.03 (0.02-0.03)
**Venezuela (Bolivarian Republic of)**	**All leukemia**	Benzene	0.78 (0.23-1.29)	0.02 (0.02-0.03)
**Venezuela (Bolivarian Republic of)**	**All leukemia**	Formaldehyde	0.28 (0.23-0.33)	0.02 (0.01-0.02)
**Venezuela (Bolivarian Republic of)**	**Acute myeloid leukemia**	Benzene	0.97 (0.28-1.63)	0.45 (0.33-0.61)
**Venezuela (Bolivarian Republic of)**	**Acute myeloid leukemia**	Formaldehyde	0.33 (0.27-0.41)	0.04 (0.03-0.05)
**Venezuela (Bolivarian Republic of)**	**Acute lymphoid leukemia**	Benzene	0.56 (0.16-0.94)	0.03 (0.02-0.03)
**Venezuela (Bolivarian Republic of)**	**Acute lymphoid leukemia**	Formaldehyde	0.2 (0.16-0.26)	0.04 (0.03-0.05)
**Venezuela (Bolivarian Republic of)**	**Chronic myeloid leukemia**	Benzene	1.09 (0.32-1.79)	0.04 (0.03-0.04)
**Venezuela (Bolivarian Republic of)**	**Chronic myeloid leukemia**	Formaldehyde	0.38 (0.31-0.47)	0.15 (0.1-0.2)
**Venezuela (Bolivarian Republic of)**	**Chronic lymphoid leukemia**	Benzene	0.51 (0.15-0.85)	0.23 (0.16-0.29)
**Venezuela (Bolivarian Republic of)**	**Chronic lymphoid leukemia**	Formaldehyde	0.19 (0.15-0.22)	0.23 (0.19-0.26)
**Venezuela (Bolivarian Republic of)**	**Other leukemia**	Benzene	0.83 (0.24-1.4)	0.23 (0.19-0.28)
**Venezuela (Bolivarian Republic of)**	**Other leukemia**	Formaldehyde	0.29 (0.24-0.36)	0.13 (0.1-0.17)
**Viet Nam**	**All leukemia**	Benzene	0.47 (0.14-0.78)	0.02 (0.02-0.03)
**Viet Nam**	**All leukemia**	Formaldehyde	0.2 (0.15-0.25)	0.15 (0.11-0.2)
**Viet Nam**	**Acute myeloid leukemia**	Benzene	0.55 (0.16-0.92)	0.35 (0.24-0.48)
**Viet Nam**	**Acute myeloid leukemia**	Formaldehyde	0.23 (0.18-0.29)	0.19 (0.14-0.25)
**Viet Nam**	**Acute lymphoid leukemia**	Benzene	0.43 (0.13-0.75)	0.03 (0.02-0.03)
**Viet Nam**	**Acute lymphoid leukemia**	Formaldehyde	0.18 (0.12-0.25)	0.3 (0.23-0.37)

Countries	Causes	Occupational carcinogens	Percent change in 1990 (95% UI)	Percent change in 2019 (95% UI)
**Viet Nam**	**Chronic myeloid leukemia**	Benzene	0.6 (0.18-1.02)	0.14 (0.1-0.19)
**Viet Nam**	**Chronic myeloid leukemia**	Formaldehyde	0.25 (0.19-0.33)	0.04 (0.03-0.05)
**Viet Nam**	**Chronic lymphoid leukemia**	Benzene	0.23 (0.06-0.4)	0.04 (0.03-0.05)
**Viet Nam**	**Chronic lymphoid leukemia**	Formaldehyde	0.1 (0.07-0.13)	0.02 (0.02-0.03)
**Viet Nam**	**Other leukemia**	Benzene	0.45 (0.13-0.76)	0.45 (0.3-0.64)
**Viet Nam**	**Other leukemia**	Formaldehyde	0.19 (0.15-0.24)	0.13 (0.09-0.18)
**Yemen**	**All leukemia**	Benzene	0.28 (0.08-0.51)	0.18 (0.13-0.26)
**Yemen**	**All leukemia**	Formaldehyde	0.1 (0.06-0.14)	0.09 (0.05-0.15)
**Yemen**	**Acute myeloid leukemia**	Benzene	0.37 (0.1-0.72)	0.02 (0.01-0.02)
**Yemen**	**Acute myeloid leukemia**	Formaldehyde	0.13 (0.08-0.2)	0.22 (0.18-0.27)
**Yemen**	**Acute lymphoid leukemia**	Benzene	0.23 (0.06-0.47)	0.18 (0.13-0.24)
**Yemen**	**Acute lymphoid leukemia**	Formaldehyde	0.08 (0.04-0.13)	0.25 (0.2-0.3)
**Yemen**	**Chronic myeloid leukemia**	Benzene	0.42 (0.11-0.8)	0.02 (0.02-0.03)
**Yemen**	**Chronic myeloid leukemia**	Formaldehyde	0.14 (0.09-0.22)	0.03 (0.02-0.03)
**Yemen**	**Chronic lymphoid leukemia**	Benzene	0.34 (0.09-0.62)	0.13 (0.1-0.16)
**Yemen**	**Chronic lymphoid leukemia**	Formaldehyde	0.12 (0.08-0.18)	0.24 (0.19-0.28)
**Yemen**	**Other leukemia**	Benzene	0.23 (0.07-0.45)	0.17 (0.13-0.21)
**Yemen**	**Other leukemia**	Formaldehyde	0.08 (0.05-0.11)	0.27 (0.22-0.33)
**Zambia**	**All leukemia**	Benzene	0.36 (0.1-0.67)	0.22 (0.14-0.29)
**Zambia**	**All leukemia**	Formaldehyde	0.1 (0.07-0.14)	0.22 (0.17-0.29)
**Zambia**	**Acute myeloid leukemia**	Benzene	0.53 (0.14-1.06)	0.18 (0.14-0.24)
**Zambia**	**Acute myeloid leukemia**	Formaldehyde	0.15 (0.08-0.25)	0.13 (0.1-0.17)
**Zambia**	**Acute lymphoid leukemia**	Benzene	0.22 (0.05-0.51)	0.25 (0.21-0.3)
**Zambia**	**Acute lymphoid leukemia**	Formaldehyde	0.06 (0.03-0.12)	0.09 (0.07-0.12)
**Zambia**	**Chronic myeloid leukemia**	Benzene	0.63 (0.14-1.39)	0.15 (0.12-0.19)
**Zambia**	**Chronic myeloid leukemia**	Formaldehyde	0.18 (0.08-0.31)	0.22 (0.18-0.28)
**Zambia**	**Chronic lymphoid leukemia**	Benzene	0.37 (0.11-0.65)	0.22 (0.17-0.29)
**Zambia**	**Chronic lymphoid leukemia**	Formaldehyde	0.1 (0.08-0.13)	0.13 (0.09-0.18)
**Zambia**	**Other leukemia**	Benzene	0.32 (0.09-0.62)	0.04 (0.03-0.05)
**Zambia**	**Other leukemia**	Formaldehyde	0.09 (0.05-0.15)	0.39 (0.27-0.56)
**Zimbabwe**	**All leukemia**	Benzene	0.63 (0.18-1.05)	0.03 (0.02-0.03)
**Zimbabwe**	**All leukemia**	Formaldehyde	0.23 (0.18-0.28)	0.13 (0.08-0.19)
**Zimbabwe**	**Acute myeloid leukemia**	Benzene	0.98 (0.28-1.65)	0.03 (0.02-0.03)
**Zimbabwe**	**Acute myeloid leukemia**	Formaldehyde	0.35 (0.27-0.46)	0.19 (0.15-0.23)
**Zimbabwe**	**Acute lymphoid leukemia**	Benzene	0.66 (0.19-1.17)	0.19 (0.16-0.23)
**Zimbabwe**	**Acute lymphoid leukemia**	Formaldehyde	0.24 (0.15-0.35)	0.28 (0.22-0.34)
**Zimbabwe**	**Chronic myeloid leukemia**	Benzene	0.81 (0.23-1.41)	0.23 (0.17-0.3)
**Zimbabwe**	**Chronic myeloid leukemia**	Formaldehyde	0.29 (0.2-0.41)	0.27 (0.23-0.32)
**Zimbabwe**	**Chronic lymphoid leukemia**	Benzene	0.5 (0.15-0.86)	0.26 (0.2-0.34)
**Zimbabwe**	**Chronic lymphoid leukemia**	Formaldehyde	0.17 (0.13-0.22)	0.12 (0.09-0.18)
**Zimbabwe**	**Other leukemia**	Benzene	0.55 (0.15-0.9)	0.2 (0.13-0.27)
**Zimbabwe**	**Other leukemia**	Formaldehyde	0.2 (0.15-0.25)	0.2 (0.15-0.28)

Abbreviations: UI, uncertainty interval.
